# A quantitative and T‐pattern analysis of anxiety‐like behavior in male GAERS, NEC, and Wistar rats bred under the same conditions, against a commercially available Wistar control group in the hole board and elevated plus maze tests

**DOI:** 10.1111/cns.14443

**Published:** 2023-09-02

**Authors:** Maurizio Casarrubea, Manuela Radic, Tatiana P Morais, Erika Mifsud, Eleonora Cuboni, Stefania Aiello, Giuseppe Crescimanno, Vincenzo Crunelli, Giuseppe Di Giovanni

**Affiliations:** ^1^ Laboratory of Behavioral Physiology, Human Physiology Section “Giuseppe Pagano”, Department of Biomedicine, Neuroscience and Advanced Diagnostics (BIND) University of Palermo Palermo Italy; ^2^ Laboratory of Neurophysiology, Department of Physiology and Biochemistry, Faculty of Medicine and Surgery University of Malta Msida Malta; ^3^ School of Biosciences, Neuroscience Division Cardiff University Cardiff UK; ^4^ Present address: Department of Paediatrics Children's Hospital Zagreb Zagreb Croatia; ^5^ Present address: Leibniz Institute for Neurobiology Magdeburg Germany

**Keywords:** absence seizures, anxiety, comorbidities, breeding, T‐pattern analysis, spike‐and‐wave discharges

## Abstract

**Aim:**

The Genetic Absence Epilepsy Rats from Strasbourg (GAERS) are an inbred polygenic model of childhood absence epilepsy (CAE), which, as their non‐epileptic control (NEC) rats, are derived from Wistar rats. While the validity of GAERS in reproducing absence seizures is well established, its use as a model for CAE psychiatric comorbidities has been subject to conflicting findings. Differences in colonies, experimental procedures, and the use of diverse controls from different breeders may account for these disparities. Therefore, in this study, we compared GAERS, NEC, and Wistar bred in the same animal facility with commercially available Wistar (Cm Wistar) as a third control.

**Methods:**

We performed hole board (HB) and elevated plus maze (EPM) tests that were analyzed with standard quantitative and T‐pattern analysis in male, age‐matched Cm Wistar and GAERS, NEC, and Wistar, bred under the same conditions, to rule out the influence of different housing factors and provide extra information on the structure of anxiety‐like behavior of GAERS rats.

**Results:**

Quantitative analysis showed that GAERS and NEC had similar low anxiety‐like behavior when compared to Cm Wistar but not to Wistar rats, although a higher hole‐focused exploration was revealed in NEC. T‐pattern analysis showed that GAERS, NEC, and Wistar had a similar anxiety status, whereas GAERS and NEC exhibited major differences with Cm Wistar but not Wistar rats. EPM results indicated that GAERS and NEC also have similar low anxiety compared to Cm Wistar and/or Wistar rats. Nevertheless, the analysis of the T‐pattern containing open‐arm entry showed GAERS and Wistar to be less anxious than NEC and Cm Wistar rats.

**Conclusion:**

To summarize, comorbid anxiety may not be present in male GAERS rats. This study also highlighted the importance of including a control Wistar group bred under the same conditions when evaluating their behavior, as using Wistar rats from commercial breeders can lead to misleading results.

## Graphical abstract

GAERS have been suggested as a model for studying childhood absence epilepsy (CAE)‐associated psychiatric comorbidities. GAERS, non‐epileptic control (NEC), and Wistar rats were compared, along with commercially available Wistar rats (Cm Wistar) as a control. Anxiety‐like behavior was evaluated using hole board (HB) and elevated plus maze (EPM) tests. T‐pattern analysis revealed similar anxiety status in GAERS, NEC, and Wistar rats, differing from Cm Wistars. EPM results showed lower anxiety levels in GAERS and Wistar rats compared to NEC rats and Cm Wistars. The study concluded that male GAERS rats from Malta colony lacked comorbid anxiety. It also highlighted the importance of using Wistar rats from the same breeding conditions to avoid misleading results.

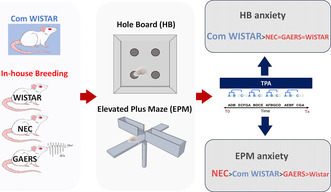

## 1 INTRODUCTION

Anxiety is a complex and multifaceted disorder that can be influenced by a wide range of factors, including gender, genetic predisposition, environmental factors, as well as neurological and neuropsychiatric disorders.^1^ Anxiety is the most prevalent psychiatric illness in the general population with a global prevalence of ~10%^2^ and up to 25% in people with epilepsy (PWE),^3^ suggesting a potential common causation for these two brain diseases. The link between the pathophysiology of epilepsy and anxiety seems to be bidirectional,^4^, ^5^ with anxiety being both induced by seizures and a risk factor for the development of epilepsy.^6^, ^7^ Unfortunately, anxiety and other psychiatric comorbidities are commonly underdiagnosed and often untreated, although they can increase the risk of suicide in PWE.^8^ Furthermore, newly diagnosed PWE with comorbid anxiety have a higher risk of recurrent seizures, despite treatment with anti‐seizure medications (ASMs), compared with those who screened negative for this condition.^9^

Although comorbid anxiety disorders are experienced by PWE of all ages, children are especially sensitive. For instance, anxiety disorders affect up to 50% of children with idiopathic childhood absence epilepsy (CAE)^10^ (see Ref. [7]), while the worldwide prevalence in children and adolescents is much lower (~10%).^11^ Anxiety also contributes to reducing the quality of life in CAE. Therefore, appropriate treatment for comorbid anxiety and epilepsy will be beneficial to CAE and their family and caregivers. Current treatment options for anxiety include antidepressants, ASMs, and benzodiazepines, although adverse effects, for example, seizure exacerbation, limit their utility in children with CAE. Therefore, an unmet need for epilepsy research is to find a treatment for comorbidities that aggravate seizures or might be also effective in halting them.^7^

Animal models of CAE have been pivotal to further our understanding of the mechanisms of epilepsy and to develop new treatments.^12^, ^13^, ^14^ In addition, it has been suggested that CAE animal models may also recapitulate anxiety‐like behaviors,^5^, ^7^ although contrasting findings were reported.^15^, ^16^, ^17^, ^18^, ^19^, ^20^, ^21^, ^22^, ^23^ This scenario is complicated by the difficulties in selecting an appropriate control group. For instance, the Genetic Absence Epilepsy Rats from Strasburg (GAERS) and the non epileptic control (NEC) were genetically developed from the original Wistar strain,^21^ raising the possibility that selected features other than absence seizures might affect the conclusions of studies of anxiety. To address this issue, a second control group of Wistar rats was included in a study of GAERS and NEC rats.^22^ However, these Wistar rats were sourced from a commercial breeder with different animal housing conditions which are known to affect the anxiety and epilepsy levels in adult animals.^24^, ^25^ Moreover, the anxiety status of CAE animal models has been evaluated with a simple ethological quantitative analysis of the behaviors. While this methodology can be informative, a more complex and sensitive analysis, such as the multivariate T‐pattern analysis (TPA), may provide additional insights through the identification of latent or easily overlooked patterns.^26^

Here, we investigated whether male GAERS, NEC, and two control groups of Wistar rats (i.e., one bred under the same conditions as GAERS and NEC and one sourced by a commercial provider, called Cm Wistar) exhibit differences in affective behavior, particularly in anxious phenotype characteristics. We used the hole board (HB) and the elevated plus maze (EPM) tests, as well as quantitative and TPA analysis. Our findings show that GAERS and NEC rats of the Maltese colony exhibit a similarly low level of anxiety. Notably, the Wistar rats, bred under the same condition as the epileptic strain and its control group, displayed lower levels of anxiety than the Cm Wistars. This difference in anxiety levels influenced the evaluation between GAERS and NEC. Moreover, our HB and EPM results indicate that multiple anxiety tests and complex analyses are key to drawing reliable conclusions in similar studies.

## 2 METHODS

### 2.1 Animals

Male GAERS, NEC, and Wistar rats (3–5 months old, 24 rats for each group) were obtained from colonies bred at the University of Malta. Additionally, 24 age‐matched Wistar rats (commercial Wistar: Cm Wistar) were purchased from Envigo RMS S.r.l. (S. Pietro al Natisone) and given 1 week to acclimate to the new environment before testing. Animals were housed in a 12:12 light cycle (lights on at 07.00 a.m. and off at 07.00 p.m.) kept in a temperature and humidity controlled (21 ± 1°C, 55 ± 5%). All animal procedures were authorized by the National Health Institute, Italy, and the University of Malta's Research Ethics Committee (UREC) (FRECMDS_1819_081), in conformity with international laws and policies (EU Directive, 2010/63/EU for animal experiments, ARRIVE guidelines, and the Basel declaration including the 3R concept). All efforts were made to minimize animal suffering and to reduce the number of animals used. All GAERS of the Maltese colony show spike‐and‐wave discharges (SWDs) in EEG recordings (not shown) as observed in other colonies.^27^

### 2.2 Behavioral analyses

#### 2.2.1 The hole board apparatus

We used a standard HB apparatus and group size, similar to our previous studies,^28^, ^29^, ^30^, ^31^, ^32^ to investigate different components of various behavioral categories.^31^, ^32^, ^33^, ^34^ Twelve animals per group were used, all of which were naive for the test and not reused in EPM. Cm Wistar, as well as Wistar, NEC, and GAERS rats bred in Malta, were tested. Eleven behavioral components, which were grouped into the following four main categories, were included in the ethogram (Figure S1): general exploration; focused exploration; grooming activity; and the last category contains only immobility (see Appendix S1 for an extensive description of the HB test).

#### 2.2.2 The elevated plus maze apparatus

A new cohort consisting of 12 Cm Wistar, 12 Wistar, 12 NEC, and 12 GAERS rats were tested using the same EPM apparatus as in^35^ and using the same ethogram as in^36^ that encompasses 24 behavioral elements occurring in the protected zones (central platform and closed arms) and the unprotected zones (open arms) (Figure S2, see Appendix S1 for an extensive description of the EPM test).

#### 2.2.3 Data analysis

Video files were analyzed using Observer XT (Noldus Information Technology) software for quantitative evaluation (frequencies and durations) of each behavioral component. Multivariate TPA of the observed pattern behaviors in the HB and EPM was performed as previously^26^, ^37^, ^38^ by using the software program Theme (Patternvision Ltd, Iceland; Noldus Information Technology bv; see Appendix S1 and Figure S3).

#### 2.2.4 Statistics

Normality of data was tested using D'Agostino & Pearson test. For data that failed normality tests, Kruskal–Wallis post‐hoc test was used and for data that passed the normality test, the frequency of occurrence and duration of the different behaviors in HB and EPM were analyzed by one‐way ANOVA (Strain × Treatment) for independent samples followed by Tukey's HSD post‐hoc test for multiple comparisons among groups, with *p* < 0.05 considered a significant value. If the data were not normally distributed, this information was included in the text of the Results section. As to TPA, the distributions of the mean number of T‐patterns of each length detected in the randomized data sets were compared with the number of patterns identified in the original data. Mean occurrences and mean length of T‐patterns detected in the raw data were assessed using two‐way ANOVA for independent samples followed by Tukey's HSD post‐hoc test for multiple comparisons among groups, with *p* < 0.05 considered a significant value. Finally, the percent distribution of T‐patterns encompassing behavioral components of hole exploration was assessed using the Pearson test, with *p* < 0.05 considered significant.

## 3 RESULTS

### 3.1 Quantitative analysis of behaviors in the hole board

#### 3.1.1 Frequency of behaviors in the HB

**FIGURE 1:**
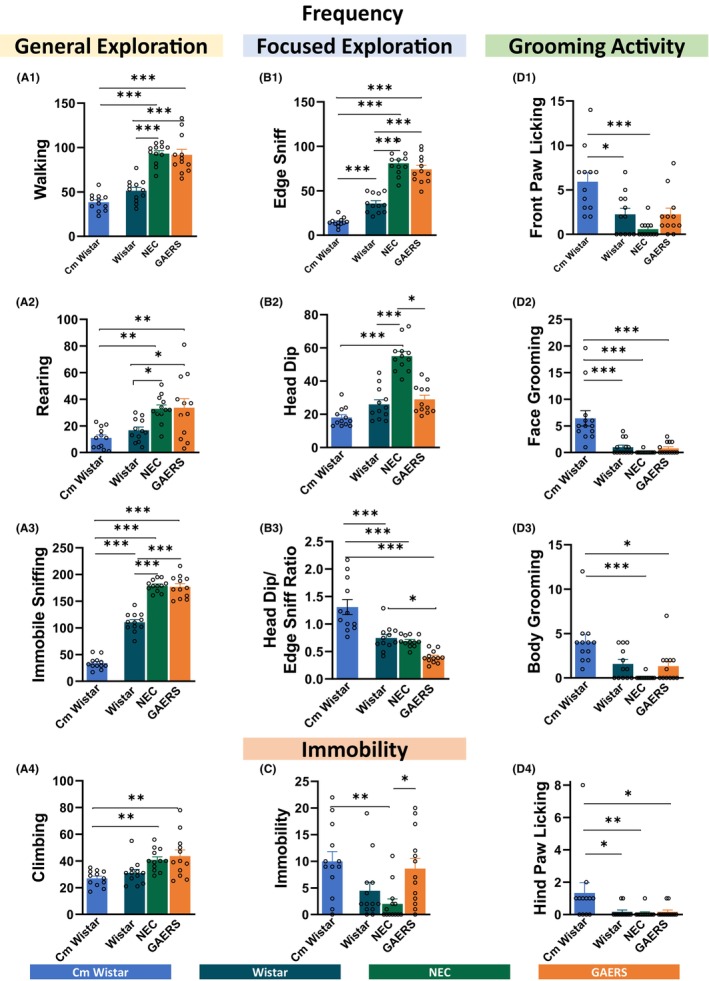
Frequency of occurrence of different behaviors in the HB test. Mean frequency ± SEM of each component of the behavioral repertoire in the HB test (see Figure S3) in Cm Wistar, Wistar, NEC, and GAERS rats. The data are divided into general exploration (A), focused exploration (B), immobility (C), and grooming activity (D). *p < 0.05, **p < 0.01, ***p < 0.001; Turkey post‐hoc test for multiple comparisons after ANOVA test, *n* = 12 rats in each group (A1–3, B1, B3), and for data that failed D'Agostino and Pearson normality test, the Kruskal–Wallis post‐hoc test was used (A4, B2, C and D1–4).

The frequencies of behaviors in the HB test for Cm Wistar, Wistar, NEC, and GAERS bred in Malta are illustrated in Figure 1. ANOVA analyses revealed significant results for all the behaviors studied (Table S1). The results from post‐hoc Tukey's test (Figure 1A1–3) and Kruskal–Wallis test (Figure 1A4) indicated that GAERS and NEC were not different in general exploration GAERS and NEC rats had equally highest frequencies of walking, rearing, immobile sniffing, and climbing compared to both Wistar (*p* < 0.05) and Cm Wistars (GAERS also for climbing, *p* < 0.05), with the exception of climbing that showed no differences between GAERS and Wistar. The only difference between Cm Wistars and Wistars was a lower frequency of immobile sniffing of the former strain (*p* < 0.001) (Figure 1A3). Regarding focused exploration, GAERS and NEC had similar highest edge sniff frequency compared to both Wistars (*p* < 0.001 for both) and Cm Wistars (*p* < 0.001 for both), with Cm Wistars being the lowest (*p* < 0.001 vs. Wistar) (Figure 1B1). In contrast, NEC showed the highest head dipping occurrence, bigger than GAERS (*p* < 0.05), Wistars (*p* < 0.001), and Cm Wistars (*p* < 0.001) (Figure 1B2, Kruskal–Wallis test). Cm Wistars had the highest head dipping/edge sniffing ratio compared to Wistar (*p* < 0.001), NEC (*p* < 0.001), and GAERS (*p* < 0.001), with GAERS showing the lowest value that was also different from that of Wistar (*p* < 0.05) (Figure 1B3). Immobility frequency was equally higher in Cm Wistar, Wistar, and GAERS, with the lowest value recorded for NEC rats compared to Cm Wistars (*p* < 0.01) and GAERS (*p* < 0.05) (Figure 1C, Kruskal–Wallis test). The occurrences of grooming activity were found to be highest in Cm Wistars while the other three strains bred in Malta had similar values (Figure 1D1–4, Kruskal–Wallis post‐hoc test). Front paw licking (Figure 1D1) was higher in Cm Wistar than in Wistar (*p* < 0.05) and NEC (*p* < 0.001). Face grooming (Figure 1D2) and body grooming (Figure 1D3) were higher in Cm Wistars than in NEC (*p* < 0.001 for both) and GAERS (*p* < 0.001 and *p* < 0.05, respectively). Differences between Cm Wistar and Wistar (*p* < 0.001) were also reported for face grooming. Differences in hind paw licking (Figure 1D4) were found between Cm Wistars and Wistar (*p* < 0.05), NEC (*p* < 0.01), and GAERS (*p* < 0.05). The cumulative occurrence of all the behaviors (Figure S4 and Table S1) performed during the 10 min session in the HB was higher in the Malta colonies, compared to Cm Wistars (*p* < 0.001), with GAERS and NEC showing similar higher values compared to Wistars.

In summary, the head dipping/edge sniffing ratio of the behavior frequency, which has been shown to be an indicator of the anxiety level,^31^ and the increased grooming activity suggests that the Maltese GAERS, NEC, and Wistar rats are more anxious than Cm Wistar. However, the frequency of most of the behaviors in the HB indicates an equally low anxiety state and neophilia for GAERS and NEC, compared to Wistar and Cm Wistar rats, with the latter group being the most anxious.

#### 3.1.2 Duration of behaviors in the HB

**FIGURE 2:**
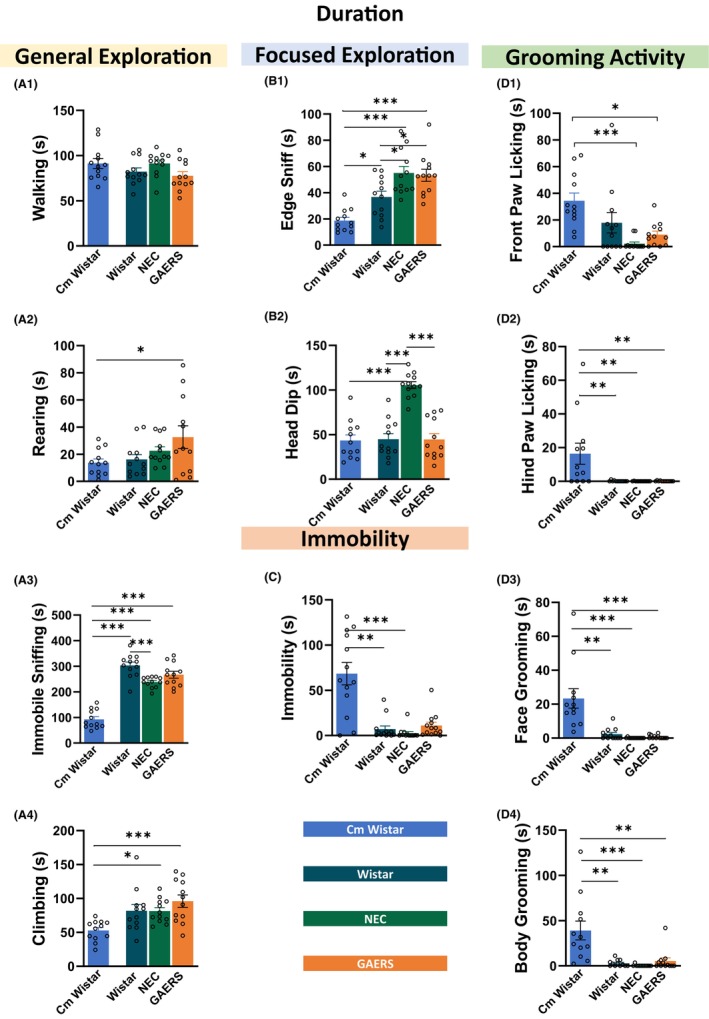
Duration of different behaviors in the HB test. Mean duration in seconds ± SEM of each component of the behavioral repertoire in the HB test (see Figure 1) in Cm Wistar, Wistar, NEC, and GAERS rats. The data are divided into general exploration (A), focused exploration (B), immobility (C), and grooming activity (D). *p < 0.05, **p < 0.01, ***p < 0.001; Turkey post‐hoc test for multiple comparisons after ANOVA test, *n* = 12 rats in each group (A1–3, C, D2–4), and for data that failed D'Agostino and Pearson normality test, the Kruskal‐Wallis post‐hoc test was used (A1, A4, B1–4, and D1).

Statistical analysis revealed no differences (Table S1) in walking time between the four groups of rats (Figure 2A1) but significant differences were found for the other general exploration behaviors. Rearing was longer in GAERS than in Cm Wistar (*p* < 0.05) but no differences were found with Wistar and NEC (Figure 2A2). Immobile sniffing was shorter in Cm Wistar than in Wistar (*p* < 0.001), NEC (*p* < 0.001), and GAERS (*p* < 0.001), and there were differences between Wistar and NEC (*p* < 0.001) (Figure 2A3). As for the climbing, Cm Wistar had a shorter climbing time than NEC and GAERS (NEC *p* < 0.05; GAERS *p* < 0.001) (Figure 2A4). Regarding the edge sniff (Figure 2B1), Wistar animals had the shortest time (NEC: *p* < 0.001 vs. Cm Wistar and *p* < 0.05 vs. Wistar; GAERS: *p* < 0.001 vs. Cm Wistar and *p* < 0.05 vs. Wistar), and differences were also present between the two Wistar groups, with Cm Wistar being those with shorter edge sniff time (*p* < 0.05). NEC spent the longest time in head dip (Figure 2B2) compared with the other three groups (Cm Wistar *p* < 0.001; Wistar *p* < 0.001; GAERS *p* < 0.001) that had a similar duration. Immobility (Figure 2C) was longer in Cm Wistar than in Wistar (*p* < 0.001) and NEC (*p* < 0.001).

**FIGURE 3:**
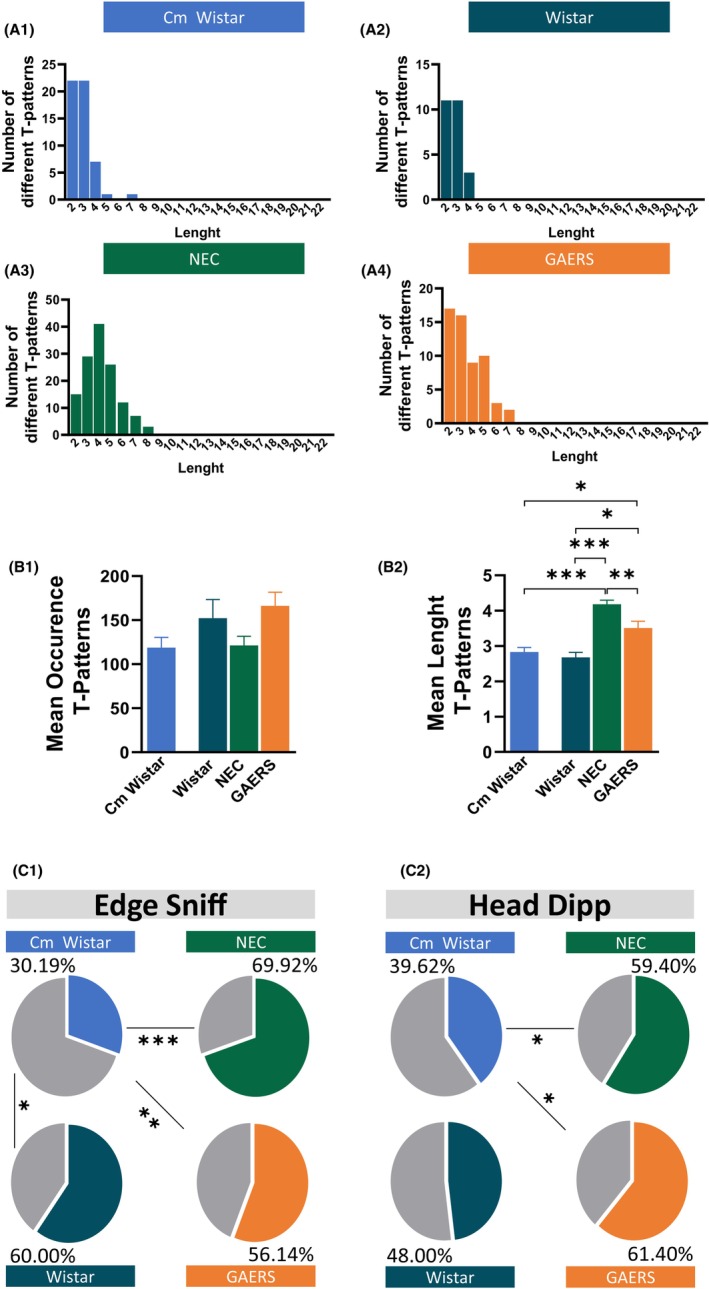
T‐pattern analysis of the HB behaviors. (A) Number of different T‐patterns in HB detected at different lengths in Cm Wistar, Wistar, NEC, and GAERS. (B) Mean occurrence ± SEM and length ± SEM of T‐patterns in HB detected in Cm Wistar, Wistar, NEC, and GAERS rats. *p < 0.05, **p < 0.01, ***p < 0.001; Turkey post‐hoc test for multiple comparisons after ANOVA test, *n* = 12 rats in each group. (C) Percentage distribution of T‐patterns in the HB test that encompassed edge sniff (C1) and head dip (C2) in Cm Wistar, Wistar, NEC, and GAERS. *p < 0.05, **p < 0.01, ***p < 0.001; Pearson test, *n* = 12 rats in each group.

**FIGURE 4:**
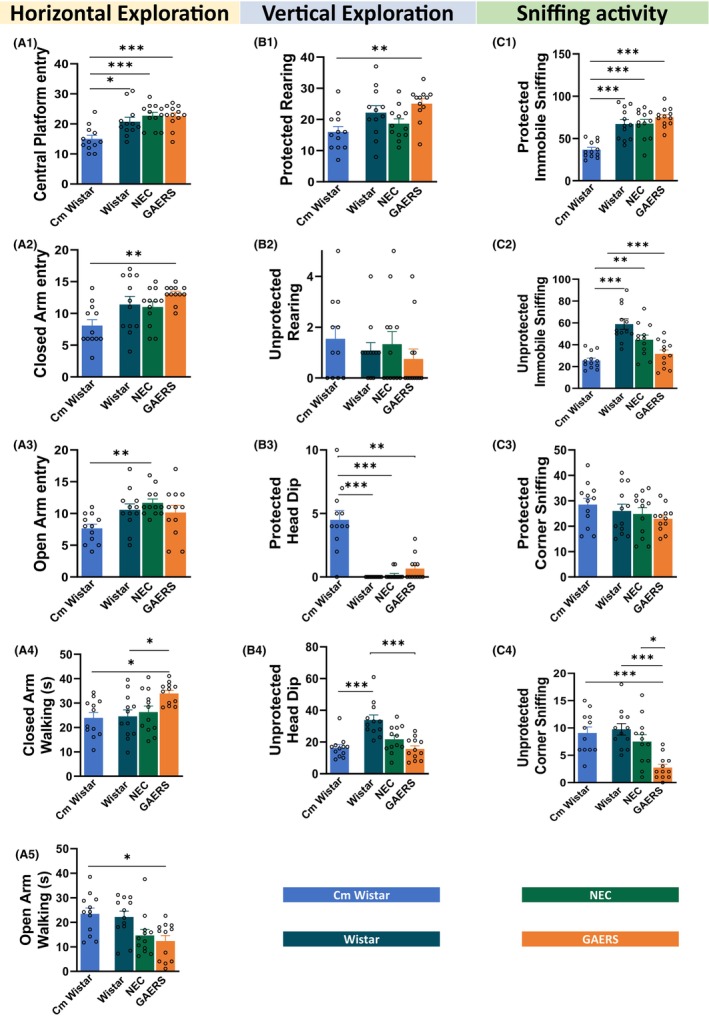
Different behaviors in the EPM test. Mean frequency ± SEM of each component of the behavioral repertoire in the EPM test (see Figure S1) in Cm Wistar, Wistar, NEC, and GAERS rats. Data are divided into horizontal exploration (A), vertical exploration (B), and sniffing activity (D). *p < 0.05, **p < 0.01, ***p < 0.001; Turkey post‐hoc test for multiple comparisons after ANOVA test, *n* = 12 rats in each group (A1–4, C, D1–4), and for data that failed D'Agostino and Pearson normality test, the Kruskal–Wallis post‐hoc test was used (A5 and B1–4).

**FIGURE 5:**
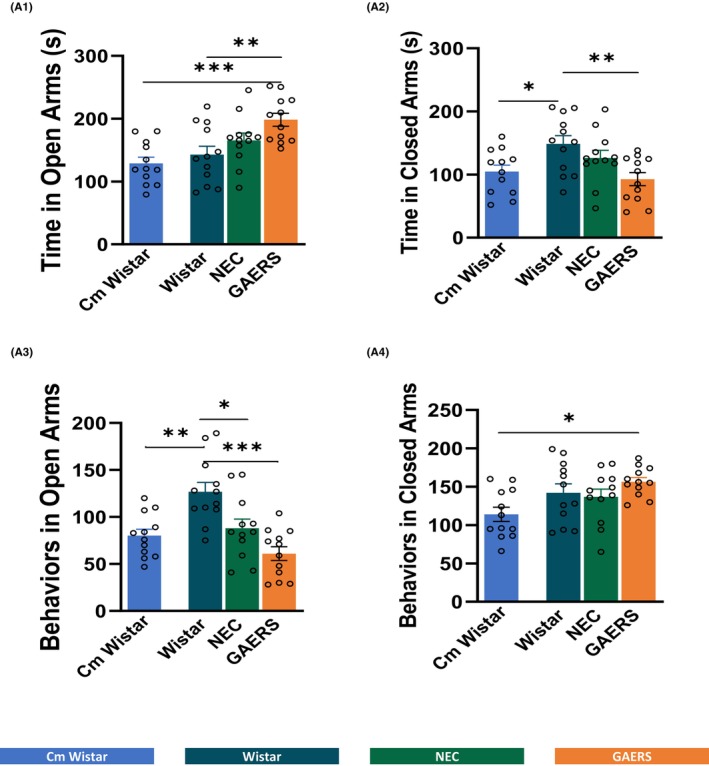
Analysis of open‐ and closed‐arm entries in the EPM. Mean duration ± SEM of time in open (A1) and closed arms (A2), and number of behaviors in open (A3) and closed arms (A4) in Cm Wistar, Wistar, NEC, and GAERS rats. *p < 0.05, **p < 0.01, ***p < 0.001; Turkey post‐hoc test for multiple comparisons after ANOVA test, *n* = 12 rats in each group.

**FIGURE 6:**
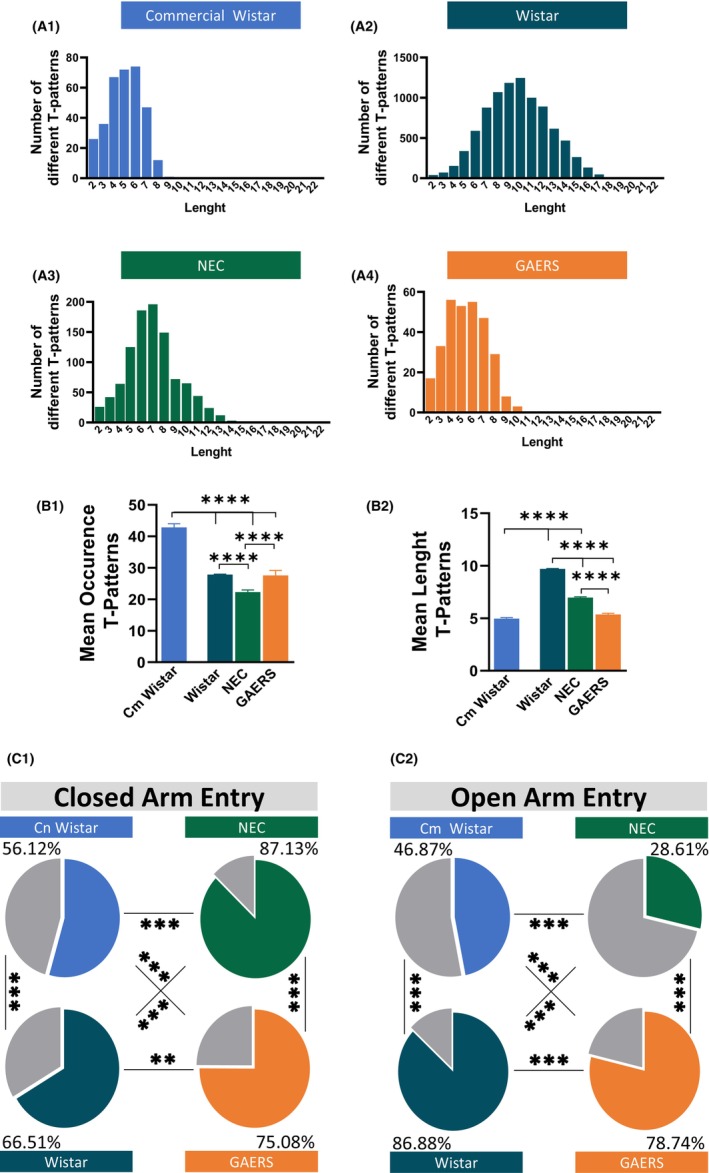
T‐pattern analysis of behavior in the EPM. (A) Number of different T‐patterns in the EPM test detected at different lengths in Cm Wistar, Wistar, NEC, and GAERS. (B) Mean occurrence ± SEM and length ± SEM of T‐patterns in EPM detected in Cm Wistar, Wistar, NEC, and GAERS rats. *p < 0.05, **p < 0.01, ***p < 0.001; Turkey post‐hoc test for multiple comparisons after ANOVA test, *n* = 12 rats in each group. (C) Percent distribution of T‐patterns in EPM that encompassed edge sniff and head dip in Cm Wistar, Wistar, NEC, and GAERS rats. *p < 0.05, **p < 0.01, ***p < 0.001; Pearson test, *n* = 12 rats in each group.

In contrast, the four parameters of grooming were also found to be different between the groups, with Cm Wistars showing the longest durations for all types of grooming (Figure 2D1–4). Front paw licking time was longer in Cm Wistar, similarly to Wistar, compared to GAERS (*p* < 0.05), and even shorter in NEC (*p* < 0.001). Hind paw licking and face and body grooming durations were longer in Cm Wistar compared to Wistar (*p* < 0.01 for all), NEC (hind paw: *p* < 0.01; face: *p* < 0.0001; body: *p* < 0.0001), and GAERS (hind paw: *p* < 0.01; face: *p* < 0.0001; body: *p* < 0.001), with no difference among the Maltese strains.

In summary, the duration of the behaviors in the HB confirms that GAERS, NEC, and Wistar show equally longer general exploration compared to Cm Wistar, indicating that the latter strain was the most anxious, although they spent longer time in grooming. Moreover, NEC are less prone to anxiety than GAERS as indicated by the hole exploration.

### 3.2 T‐patterns analysis of the temporal structure of the behaviors in the HB

To gain more insights into the anxiety levels and clarify some inconsistencies revealed in the above quantitative analysis among the different rat groups, we performed TPA. TPA is a method used to analyze the temporal structure of behavior, and to identify and characterize the recurring and significant behavioral sequences (See Methods and Appendix S1). Using TPA to study rat anxiety‐like behavior could provide valuable insights into the temporal structure of these behaviors and how they relate to each other.^39^ This analysis revealed that NEC had the most complex behavior (since they had a total of 133 T‐patterns, NEC showed T‐patterns up to 8 events, GAERS 7 events, and Cm Wistar 7 events, in turn, Wistar 4 events (Figure 3A1–4; Figure S5). ANOVA showed that the mean occurrence (Table S1; Figure 3B1) and length (Figure 3B2) of the T‐patterns were different between the four groups. NEC had the longest T‐patterns compared to GAERS (*p* < 0.01), Cm Wistar (*p* < 0.001), and Wistar (*p* < 0.001), with that of GAERS being longer than Cm Wistar (*p* < 0.05) and Wistar (*p* < 0.05). NEC, therefore, had T‐patterns with high complexity, suggesting a more adaptative and flexible behavior that requires the coordination of multiple elements in response to the exposition of a new environment.

T‐patterns encompassing behavioral components of hole exploration are better predictive of the animal anxiety level compared to their quantitative assessment.^39^ Pearson test showed that NEC had the highest percentage of T‐patterns containing edge sniff, followed by Wistar, GAERS, and Cm Wistar (*p* < 0.001) (Figure 3C1). Regarding the T‐patterns containing Head dip, GAERS had the highest number, followed by NEC, Wistar, and Cm Wistar (*p* < 0.05) (Figure 3C2).

In conclusion, TPA indicates that GAERS and NEC have a similarly low level of anxiety‐like behavior when compared to Cm Wistar but not to Wistar, with Cm Wistar being more anxious than Wistar rats.

### 3.3 Quantitative analysis of behaviors in elevated plus maze

#### 3.3.1 Frequency of behaviors in the EPM

ANOVA revealed significant results for the horizontal exploration parameters (Table S1). Cm Wistar showed the fewest central platform entries (Figure 4A1) in comparison to GAERS (*p* < 0.001), NEC (*p* < 0.001), and Wistar (*p* < 0.05). Interestingly, closed‐arm entry (Figure 4A2) was higher in GAERS compared to Cm Wistar (*p* < 0.01) and open‐arm entry (Figure 4A3) was only higher in NEC compared to Cm Wistar (*p* < 0.01). Close‐arm walking (Figure 4A4) was higher in GAERS than in the two Wistar groups (*p* < 0.05 for both). On opposite, open‐arm walking (Figure 4A5) was the smallest in GAERS, being the time spent walking different from Cm Wistar (*p* < 0.05). Regarding vertical exploration, protected rearing (Table S1; Figure 4B1) was only higher in GAERS compared to Cm Wistar (*p* < 0.01), whereas unprotected rearing was similar among the groups (Figure 4B2). Protected head dip (Figure 4B3) was higher in Cm Wistar compared to the three other groups (Wistar: *p* < 0.001; NEC: *p* < 0.001; GAERS: *p* < 0.01), whereas unprotected head dips were higher in Wistar than in Cm Wistar (*p* < 0.001) and GAERS (*p* < 0.001) (Figure 4B4).

Sniffing activity, as immobile sniffing, was higher in the in‐house breed strains compared to Cm Wistar both in protected (Wistar: *p* < 0.001; NEC: *p* < 0.001; GAERS: *p* < 0.001) (Figure 4C1) and unprotected areas (Wistar: *p* < 0.001; NEC: *p* < 0.01; GAERS: *p* < 0.001) (Figure 4C2). Protected corner sniffing was similar in the four groups, but unprotected corner sniffing was smaller in GAERS compared to Cm Wistar (*p* < 0.001), Wistar (*p* < 0.001), and NEC (*p* < 0.05) (Figure 4C4). Figure S6 and Table S2 shows that the other parameters studied in the EPM (protected paw licking, unprotected paw licking, protected head dip, unprotected head dip, and sniffing activity) also indicate greater values for Cm Wistar compared to the Wistar, GAERS, and NEC.

In conclusion, GAERS, NEC, and Wistar had similar levels of anxiety‐like behavior with GAERS being the most explorative of the novel environment in contrast to Cm Wistar, which showed the least exploratory behavior.

#### 3.3.2 Duration of behaviors in the EPM

ANOVA showed differences in time spent and the number of behaviors in open arms and closed arms among the four groups of rats (Table S1). GAERS spent more time in open arms compared to Wistar (*p* < 0.01) and Cm Wistar (*p* < 0.001) (Figure 5A1). On the other hand, GAERS spent less time in closed arms (central platform plus closed arms) compared to Wistar (*p* < 0.01), and Cm Wistar less than Wistar (*p* < 0.05). GAERS and NEC had an equally lower number of behaviors performed in open arm compared to Wistar (*p* < 0.05 NEC and *p* < 0.001 GAERS) which had a higher number than Cm Wistar (*p* < 0.01) (Figure 5A3). GAERS had a higher number of behaviors in closed arm compared to Cm Wistar (*p* < 0.05) (Figure 5A4).

### 3.4 TPA of the temporal structure of behaviors in the EPM

Cm Wistar showed a total of 335 T‐patterns, that is, sequences of behavior patterns composed of recurring simple behaviors with statistical relation^40^ (Figure 6A1), Wistar 9000 (Figure 6A2), NEC 1010 (Figure 6A3), and GAERS 301 (Figure 6A4; Figure S7). ANOVA showed differences in the occurrence and length of T‐patterns (Table S1). The mean occurrence of T‐patterns (Figure 6B1) was different between the four groups, with Cm Wistar having the highest occurrence of T‐patterns compared with Wistar (*p* < 0.001), NEC (*p* < 0.001), and GAERS (*p* < 0.001). NEC was the group with the lowest occurrence of T‐patterns compared to Wistar (*p* < 0.001) and GAERS (*p* < 0.001). The length of the T‐patterns was also different among groups, but in this case, Cm Wistar had a lower length of T‐patterns compared to Wistar (*p* < 0.001) and NEC (*p* < 0.001). Differences were also observed between Wistar and NEC (*p* < 0.001), Wistar and GAERS (*p* < 0.001), and NEC and GAERS (*p* < 0.001).

Pearson test showed that the percentage of T‐patterns containing closed‐arm entry was higher in NEC, followed by GAERS, Wistar, and Cm Wistar (all comparisons *p* < 0.001, except *p* < 0.01 for Wistar vs. GAERS) (Figure 6C1). As for the open‐arm entry‐containing T‐patterns percentage, the sequence from the lowest to the highest was NEC, Cm Wistar, GAERS, and Wistar (all *p* < 0.001) (Figure 6C2).

In conclusion, TPA indicates that GAERS are less anxious than NEC, as well as Wistar compared to Cm Wistar, a finding that was not revealed by the quantitative analysis. Differently from the TPA data of the HB, Wistar rats show a markedly more complex behavioral structure than Cm Wistar.

## 4 DISCUSSION

Three main conclusions arise from this study involving male animals: (i) GAERS and NEC rats from the Malta colony, although showing peculiar behavioral profiles, display similar anxiety‐like behavior in two tests of emotionality, namely the HB and EPM; (ii) GAERS and NEC have a low‐anxiety phenotype and neophilia traits compared to Cm Wistar, but not to the in‐house bred Wistar; and (iii) the majority of the behavioral data from HB are in agreement with those from the EPM since the TPA revealed a lower‐anxiety phenotype for NEC rats. Thus, the inclusion of a third control group, that is, in‐house‐bred Wistar rats, has allowed us to reveal that most of the anxiety‐related differences between GAERS and NEC are with the Cm Wistars, highlighting the importance of comparing rats born and raised in the same conditions (see Graphical Abstract).

Animal models have been pivotal to further our understanding of CAE.^7^ In particular, the GAERS rats have strong predictive, face, and construct validity for the pathophysiology and treatment of absence seizures presenting SWDs in the EEG and contextual behavioral arrest and sensitivity to anti‐absence drugs.^7^, ^12^, ^13^, ^41^ However, no single model does fully recapitulate the human phenotype because each one possesses its advantages and limitations. For instance, although commonly accepted,^5^ there is no definitive proof that CAE animal models recapitulate anxiety‐like behaviors. For example, Wistar Albino Glaxo from Rijswijk (WAG/Rij) rats, another widely used CAE animal model,^42^ show dysthymia^14^ and cognitive impairment^43^ both secondary to SWDs, but not anxiety.^44^

Moreover, there have been inconsistent results reported on the anxiety levels of GAERS rats from different colonies around the world and within the same colony at different times. Thus, GAERS from the original colony in Strasburg are either equally anxious as NEC^21^ or more anxious than NEC in a more recent study in the EPM and open field.^22^ Less anxiety‐related behavior than NEC was also reported in GAERS from the colonies in Melbourne and Saskatoon.^15^, ^16^, ^17^, ^18^, ^19^, ^20^ However, recent works from the Canadian colony did not observe any significant difference in anxiety‐like behavior between GAERS and NEC.^20^, ^23^ Similarly, we have recently reported that GAERS from the Malta colony are less anxious than NEC in the HB,^28^ in contrast to the present observations. Notably, however, the animals in De Deurwaerdère et al.^28^ were treated with a drug vehicle and not naive as in this current study. A maladaptive response to stress due to the restrain during the vehicle injection^45^ might have determined the higher level of anxiety observed in vehicle‐treated NEC compared to GAERS.^28^ Indeed, the head dip frequency and duration in vehicle‐treated NEC (but also in GAERS) were lower than those of naive NEC.^28^ Here, we did not observe any major differences in anxiety phenotype and neophilia between naive GAERS and NEC rats in the HB test. Both quantitative analysis of frequency and duration and TPA of the different behaviors support this conclusion, although the quantitative data seem to show an increased hole exploration, which is not supported by the more sophisticated TPA. This further underscores the importance of the latter type of analysis in interpreting behavioral data.^26^, ^29^, ^30^, ^33^, ^34^, ^37^, ^38^, ^39^

Since the study of anxiety in animals is challenging, it is always advisable to apply multiple tests.^46^ Therefore, we exposed a different cohort of rats to the EPM, a reliable test for anxiety that we had previously used in GAERS.^35^ Consistent with the HB data, GAERS and NEC did not exhibit different anxiety levels, performing similarly in walking and vertical exploration and spending equal time in the open and closed arms. TPA, however, showed a marked difference, with NEC exhibiting more structured behavior with longer T‐patterns and the lowest frequency of the T‐pattern behaviors encompassing entries in the open arms and the highest of those containing the entrances in the closed arms. This indicates that GAERS are less anxious than NEC rats.

Genetic absence epilepsy rats from Strasbourg and NEC are inbred rats derived from outbred Wistars.^21^ Thus, their level of anxiety might depend on a genetic selection of other characteristics associated with epilepsy. When GAERS were compared to Wistars as a second control, their level of anxiety was much lower than Wistars but more anxious than that of NEC in the EPM. This suggests that GAERS were mistakenly characterized as an anxious strain due to the lack of appropriate controls. These data were confirmed in the same colony in the open‐field test, where GAERS spent significantly more time in the central area and entered significantly more frequently this zone compared to Wistar.^47^ To test whether the low anxious phenotype in NEC rats might have affected our results, we included a Wistar group obtained from a commercial supplier (Cm Wistar), as in Marques‐Carneiro and colleagues' work.^22^ As we described above, quantitative analysis shows GAERS and NEC having similar anxiety‐like behavior in both HB and EPM and the inclusion of the Cm Wistars allows us to establish that they have low‐anxiety levels. However, TPA analysis of EPM data, but not of HB data, showed that NEC rats were more anxious than GAERS, which contradicts the findings of the quantitative analysis and refuted the hypothesis of genetic drifts of genes linked to the anxiety phenotype of NEC.^22^ The use of Cm Wistars leaves open the possibility that the low‐anxiety states of our GAERS and NEC colonies might depend on the different breeding and housing conditions known to affect the animal levels of anxiety and epilepsy.^24^, ^25^ Therefore, to address this issue, for the first time, we included a third control group, that is, Wistar rats bred under the same conditions as GAERS and NEC. This revealed that Wistar, GAERS, and NEC bred under the same conditions share similar explorative and anxiety‐like behavior, which was distinct from the more anxious phenotype of Cm Wistar rats. TPA analysis of the HB data showed that GAERS and NEC rats displayed a low‐anxiety state only when compared with Cm Wistars but were similar to the in‐house‐bred Wistars, while TPA analysis of EPM data showed that Cm Wistars were more anxious than Wistars. These results conclusively demonstrate that different housing conditions can lead to different levels of anxiety among animals of the same strain, as reported previously.^48^, ^49^

The current study does have certain limitations. Specifically, the data from the HB and EPM were obtained from two distinct cohorts of animals and were not subjected to EEG recordings. Given that a proportion of Wistar rats display SWDs^22^, ^50^ and the frequency and duration of seizures are directly linked to anxiety levels,^51^ it is yet to be determined whether SWD variability plays a role in our findings. Additionally, it is worth noting that our study solely utilized male rats, which may limit our understanding of potential gender differences in the manifestation of comorbid anxiety in CAE. Therefore, future research should encompass both male and female rats to comprehensively explore and elucidate if any gender‐specific variations in comorbid anxiety are associated with CAE.

In conclusion, our findings demonstrate the lack of a comorbid anxiety phenotype in male GAERS rats. This discrepancy highlights the need to consider the limitations of animal models and the unique characteristics of human conditions such as anxiety (as comorbid disorder or not) when interpreting research findings. Moreover, we showed the importance of including Wistar rats bred under the same conditions instead of the commercially available ones as control for the inbred epileptic GAERS animals and their NEC control. Finally, performing quantitative and TPA analysis in behavioral studies is of pivotal importance.

## AUTHOR CONTRIBUTIONS

G.D.G. conceived the study, designed the methodology, and performed data interpretation. M.C., E.C., E.M., and M.R. conducted all the laboratory‐based research, M.C., M.R., T.P.M., S.A., and G.D.G. performed data analysis and interpretation. M.C., T.P.M., V.C., and G.D.G. wrote the manuscript, and T.P.M. and M.C. prepared the figures. M.C., M.R., T.P.M., S.A., G.C., V.C., and G.D.G. reviewed and edited the manuscript. All authors have agreed to this manuscript submission for publication.

## CONFLICT OF INTEREST STATEMENT

The authors declare that the research work was conducted in the absence of any commercial or financial relationships that could be construed as a potential conflict of interest. G.D.G and M.C are Editorial Board members of CNS Neuroscience and Therapeutics.

## Supporting information


Appendix S1.

## Data Availability

The datasets generated during the current study are available from the corresponding author upon reasonable request.
