# Supplementary material for: A quantitative and T‐pattern analysis of anxiety‐like behavior in male GAERS, NEC, and Wistar rats bred under the same conditions, against a commercially available Wistar control group in the hole board and elevated plus maze tests
Source: CNS Neurosci Ther. 2023 Sep 2;30(3):e14443. doi: 10.1111/cns.14443 (PMC10916429; doi:10.1111/cns.14443)
Supplement: Supplementary file 1 — Appendix S1. [file CNS-30-e14443-s001.pdf]

A Quantitative and T-Pattern Analysis of Anxiety-like Behavior in Male GAERS, NEC and Wistar Rats Bred under the Same Conditions, Against a Commercially Available Wistar Control Group in the Hole Board and Elevated Plus Maze Tests

Maurizio Casarrubea, Manuela Radic, Tatiana Pinto Morais, Stefania Aiello, Giuseppe Crescimanno, Vincenzo Crunelli, Giuseppe Di Giovanni

SUPPLEMENTARY INFORMATION

Supplementary Methods

Behavioral Analyses

Hole Board apparatus

The hole-board (HB) apparatus used in the present study consisted of a square (50 × 50 cm) arena made of white opaque Plexiglas with a raised floor, containing four equidistant holes, each 4 cm in diameter. Each hole center was 10 cm distant from the two adjacent Plexiglas walls and all four holes were equidistant. The floor of the apparatus was positioned 5 cm above a white opaque Plexiglas sub-floor. The square arena was surrounded by three white opaque Plexiglas walls (50 × 50 cm) and a front transparent wall (50 × 50 cm). A digital video camera (Toshiba HDDV P10) was placed in front of the transparent wall to record the rat behavior. The behavior of each animal was recorded on the cam's SD card. At the end of each day's recording sessions, video files from the SD card were transferred and stored on a personal computer for the following analyses.

Experimental procedure

On the day of the experiment, rats were allowed to acclimatize to the testing room for 30 min. Before the start of a new test or the use of a new animal, the apparatus was carefully cleaned with ethyl alcohol to remove possible scent cues left by the preceding animal. Each rat, naïve to the test, was placed in the central area of the apparatus, allowed to explore for 10 min and its behavior was recorded using a digital camera.

Data analysis

A list of all the components of the behavioral repertoire and their formal description is shown in Suppl. Fig. 3.

Based on the ethogram shown in Suppl. Fig. 3, video files of each rat were annotated by a trained observer, who was blind to the strain, using a professional software tool (The Observer, Noldus Information Technology, Netherlands) and event log files were generated for each animal. The propaedeutic assessment of reliability was evaluated based on five video files, randomly taken from the experimental recordings, and scored by the observer in two different moments. In a second step, after such a propaedeutic step, all video files were annotated. Once event log files of all rats had been obtained, they were rearranged to contain exclusively performed behaviors and their onset. These files were then utilized to perform both quantitative and T-Pattern analyses.

T-Pattern analysis (TPA) is a multivariate technique able to reveal the temporal architecture of behavior based on the detection of statistically significant time distances among events in sequence (Magnusson, 1996). Such a procedure is divided into two different procedures: the detection step and the analysis step. TPA orbits around the utilization of a software tool known as Theme (PatternVision Ltd., Iceland). In brief, given a distribution of events occurring within the observation period Time 0 (T0) to time x (Tx) (i.e., T0-Tx), a search algorithm compares the distributions of each pair of the behavioral events, e.g. "a" and "b", searching for a time window so that "a" is followed by "b" within such a window. If this condition between event "a" and event "b" is verified, a first-level T-Pattern encompassing only two events, that is (a b), is detected; then such first level T-Pattern is considered as a potential "a" or "b" terms for the detection of higher-order patterns, e.g., ((a b) c)… and so on up to any level. When no more patterns are detected the search stops. Concepts, theories and procedures concerning the detection and analysis of T-Patterns have been extensively described previously (Casarrubea, Jonsson et al. 2015, Casarrubea, Magnusson et al. 2018). Search parameters used: significance level = 0.0001; lumping factor = 0.90; minimum percent of samples = 100%. For each group, the following behavioral responses were analyzed: mean occurrences and mean duration of each behavioral component; the structure of all the different T-Patterns (terminal strings); length distribution of different T-Patterns both in real and randomly generated data; mean length of T-Patterns; mean occurrences of T-Patterns and, finally, percent distribution of T-Patterns encompassing behavioral components of hole-exploration.

2.2. Elevated Plus Maze apparatus

The Elevated Plus Maze (EPM) apparatus was made of ivory Perspex with its arms 50 cm long and 10 cm wide. The apparatus was positioned 50 cm above the floor. The closed arms were surrounded by a 50 cm wall, the open ones presented 0.5 cm edges in order to maximize open-arm entries The maze floor was covered with grey plastic. The environmental temperature was maintained equal to the temperature measured in the housing room. The testing room was illuminated with a dim white light that provided 100 lux for the open arms and 50 lux for the closed ones.

2.3. Experimental procedure

Rats were transported from the housing to the testing room inside their home cages to minimize

the transfer effect. To avoid possible visual and/or olfactory influences, animals were allowed to acclimate for 30 min away from the observational apparatus. Each subject, experimentally naïve, was placed in the central platform of the EPM, facing an open arm, and allowed to freely explore for 5 min. After each animal experiment, the EPM was cleaned with ethyl alcohol (10%) to remove scent cues left from the preceding subject. Experiments were recorded through a video camera and video files were stored in a personal computer for the following analyses.

2.4. Ethogram and coding

The present study employed an ethogram that was based on previous research (Casarrubea, Roy et al. 2013). The result of the coding process is the event log file, that is, a sequence of behavioral events occurring at specific time points (namely, milliseconds, seconds or, even, video frames). In the present study, all video files were coded using The Observer (Noldus Information Technology, The Netherlands). To assess the temporal relationships among behavioral events, log files were processed using the software program Theme (Patternvision Ltd, Iceland; Noldus Information Technology bv, The Netherlands).

2.5. Quantitative analysis

Frequencies and percent distributions were calculated to evaluate the occurrence of each behavioral element and its impact within the comprehensive behavioral repertoire in the maze. In addition, to assess possible modifications during the observational period, the time spent by animals in the Open Arms, in the Central Platform and in the Closed Arms was calculated minute-by-minute.

2.6. Multivariate T-Pattern Analysis

Theme's detection algorithm searches for relationships between events in behavioral data, by taking into account the order, timing, and frequency of these events (Suppl. Fig. 5) (Casarrubea, Roy et al. 2013). Following T-patterns' detection the following evaluations were carried out: mean length of T-patterns; mean occurrences of T-patterns; length distribution of different T-patterns, percent distribution of T-patterns encompassing specific behavioral components.

Terminal strings, i.e. the structure of all the different T-patterns, their occurrences and related length are presented in the supplementary online material both for HB and EPM.

A more detailed description of concepts, theories and procedures behind T-pattern detection and analysis can be found in our recent monographic works (Casarrubea and Di Giovanni, 2020, Magnusson et al., 2016), in our reviews (Casarrubea et al., 2015c, Casarrubea et al., 2018, Aiello et al., 2020) and in various articles (Magnusson, 1996, Magnusson, 2000, Magnusson, 2004, Magnusson 2020, Casarrubea et al., 2013a, Casarrubea et al., 2013b, Casarrubea et al., 2014, Casarrubea et al., 2015a, Casarrubea et al., 2015b, Casarrubea et al., 2016a, Casarrubea et al., 2016b, Casarrubea et al., 2019, Santangelo et al., 2018).

Statistics

As to mean occurrences, mean durations and the ratio between Head-Dip and Edge-Sniff, possible significant differences among groups were assessed using One-Way Analysis of Variance (ANOVA) followed by Tukey post-hoc test for multiple comparisons ($p < 0.05$ was considered a significant value).

Concerning T-patterns, even if each sequence implies the existence of statistically significant constraints among the events in patterns, in data with thousands of events, an exceedingly high number of possible relationships might be possible. Such an aspect could raise an important issue, i.e. whether the T-patterns have been detected only by mere chance. Theme software deals with such an issue by repeatedly randomizing and reanalyzing the original data, using the same search parameters utilized in the detection process performed in the real data. After that, the mean number of T-patterns detected in the randomized data (+ 1SD) is compared with the number of patterns identified in the original data. Mean occurrences and mean length of T-patterns detected in real data were assessed using ANOVA followed by the Tukey post-hoc test for multiple comparisons ($p < 0.05$ was considered a significant value).

References

Aiello, S., Crescimanno, G., Di Giovanni, G. & Casarrubea, M. 2020. T-Patterns In The Study Of Movement And Behavioral Disorders. Physiol Behav, 215, 112790.

Casarrubea, M. & Di Giovanni, G. 2020. Application Of T-Pattern Analysis In The Study Of The Organization Of Behavior. Physiol Behav, 227, 113138.

Casarrubea, M., Aiello, S., Di Giovanni, G., Santangelo, A., Palacino, M. & Crescimanno, G. 2019. Combining Quantitative And Qualitative Data In The Study Of Feeding Behavior In Male Wistar Rats. Front Psychol, 10, 881.

Casarrubea, M., Cancemi, D., Cudia, A., Cardaci, M., Sorbera, F., Faulisi, F., Magnusson, M. S. & Crescimanno, G. 2015a. Application Of Multivariate T-Pattern Analysis In The Study Of Social Interaction In Rats. Journal Of Biological Research (Italy), 88, 33-35.

Casarrubea, M., Davies, C., Faulisi, F., Pierucci, M., Colangeli, R., Partridge, L., Chambers, S., Cassar, D., Valentino, M., Muscat, R., Benigno, A., Crescimanno, G. & Di Giovanni, G. 2015b. Acute Nicotine Induces Anxiety And Disrupts Temporal Pattern Organization Of Rat Exploratory Behavior In Hole-Board: A Potential Role For The Lateral Habenula. Front Cell Neurosci, 9, 197.

Casarrubea, M., Di Giovanni, G. & Crescimanno, G. 2021. Effects Of Different Anxiety Levels On The Behavioral Patternings Investigated Through T-Pattern Analysis In Wistar Rats Tested In The Hole-Board Apparatus. Brain Sciences, 11, 14.

Casarrubea, M., F. Faulisi, M. Pensabene, C. Mendola, R. Dell'utri, M. Cardaci, A. Santangelo And G. Crescimanno (2017). "Effects Of The Benzodiazepine Inverse Agonist Fg7142 On The Structure Of Anxiety-Related Behavior Of Male Wistar Rats Tested In Hole Board." Psychopharmacology (Berl) **234**(3): 381-391.

Casarrubea, M., F. Sorbera And G. Crescimanno (2009). "Structure Of Rat Behavior In Hole-Board: I) Multivariate Analysis Of Response To Anxiety." Physiol Behav **96**(1): 174-179.

Casarrubea, M., F. Sorbera And G. Crescimanno (2009). "Structure Of Rat Behavior In Hole-Board: Ii) Multivariate Analysis Of Modifications Induced By Diazepam." Physiol Behav **96**(4-5): 683-692.

Casarrubea, M., Faulisi, F., Caternicchia, F., Santangelo, A., Di Giovanni, G., Benigno, A., Magnusson, M. S. & Crescimanno, G. 2016a. Temporal Patterns Of Rat Behaviour In The Central Platform Of The Elevated Plus Maze. Comparative Analysis Between Male Subjects Of Strains With Different Basal Levels Of Emotionality. J Neurosci Methods, 268, 155-62.

Casarrubea, M., Faulisi, F., Magnusson, M. S. & Crescimanno, G. 2016b. The Effects Of Morphine On The Temporal Structure Of Wistar Rat Behavioral Response To Pain In Hot-Plate. Psychopharmacology (Berl), 233, 2891-900.

Casarrubea, M., Faulisi, F., Pensabene, M., Mendola, C., Dell'utri, R., Cardaci, M., Santangelo, A. & Crescimanno, G. 2017. Effects Of The Benzodiazepine Inverse Agonist Fg7142 On The Structure Of Anxiety-Related Behavior Of Male Wistar Rats Tested In Hole Board. Psychopharmacology (Berl), 234, 381-391.

Casarrubea, M., Jonsson, G. K., Faulisi, F., Sorbera, F., Di Giovanni, G., Benigno, A., Crescimanno, G. & Magnusson, M. S. 2015c. T-Pattern Analysis For The Study Of Temporal Structure Of Animal And Human Behavior: A Comprehensive Review. J Neurosci Methods, 239, 34-46.

Casarrubea, M., M. Pierucci, S. Aiello, D. Cassar, G. Deidda, G. Crescimanno And G. Di Giovanni (2020). "Effects Of Chronic Nicotine On The Temporal Structure Of Anxiety-Related Behavior In Rats Tested In Hole-Board." Prog Neuropsychopharmacol Biol Psychiatry **96**: 109731.

Casarrubea, M., M. S. Magnusson, M. T. Anguera, G. K. Jonsson, M. Castañer, A. Santangelo, M. Palacino, S. Aiello, F. Faulisi, G. Raso, S. Puigarnau, O. Camerino, G. Di Giovanni And G. Crescimanno (2018). "T-Pattern Detection And Analysis For The Discovery Of Hidden Features Of Behaviour." J Neurosci Methods **310**: 24-32.

Casarrubea, M., Magnusson, M. S., Roy, V., Arabo, A., Sorbera, F., Santangelo, A., Faulisi, F. & Crescimanno, G. 2014. Multivariate Temporal Pattern Analysis Applied To The Study Of Rat Behavior In The Elevated Plus Maze: Methodological And Conceptual Highlights. J Neurosci Methods, 234, 116-26.

Casarrubea, M., Roy, V., Sorbera, F., Magnusson, M. S., Santangelo, A., Arabo, A. & Crescimanno, G. 2013a. Significant Divergences Between The Temporal Structure Of The Behavior In Wistar And In The Spontaneously More Anxious Da/Han Strain Of Rats Tested In Elevated Plus Maze. Behav Brain Res, 250, 166-73.

Casarrubea, M., Roy, V., Sorbera, F., Magnusson, M. S., Santangelo, A., Arabo, A. & Crescimanno, G. 2013b. Temporal Structure Of The Rat's Behavior In Elevated Plus Maze Test. Behav Brain Res, 237, 290-9.

Casarrubea, M., Sorbera, F. & Crescimanno, G. 2009a. Structure Of Rat Behavior In Hole-Board: I) Multivariate Analysis Of Response To Anxiety. Physiol Behav, 96, 174-9.

Casarrubea, M., Sorbera, F. & Crescimanno, G. 2009b. Structure Of Rat Behavior In Hole-Board: Ii) Multivariate Analysis Of Modifications Induced By Diazepam. Physiol Behav, 96, 683-92.

Casarrubea, M., Sorbera, F., Magnusson, M. S. & Crescimanno, G. 2011. T-Pattern Analysis Of Diazepam-Induced Modifications On The Temporal Organization Of Rat Behavioral Response To Anxiety In Hole Board. Psychopharmacology (Berl), 215, 177-89.

Casarrubea, M., V. Roy, F. Sorbera, M. S. Magnusson, A. Santangelo, A. Arabo And G. Crescimanno (2013). "Temporal Structure Of The Rat's Behavior In Elevated Plus Maze Test." Behav Brain Res **237**: 290-299.

Magnusson, M. S. 1996. Hidden Real-Time Patterns In Intra- And Inter-Individual Behavior: Description And Detection. European Journal Of Psychological Assessment, 12, 112-123.

Magnusson, M. S. 2000. Discovering Hidden Time Patterns In Behavior: T-Patterns And Their Detection. Behavior Research Methods Instruments & Computers, 32, 93-110.

Magnusson, M. S. 2004. Repeated Patterns In Behavior And Other Biological Phenomena, The Mit Press, Cambridge.

Magnusson, M. S. 2020. T-Patterns, External Memory And Mass-Societies In Proteins And Humans: In An Eye-Blink The Naked Ape Became A String-Controlled Citizen. Physiol Behav, 227, 113146.

Magnusson, M. S., Burgoon, J. K. & Casarrubea, M. (Eds.) 2016. Discovering Hidden Temporal Patterns In Behavior And Interaction. T-Pattern Detection And Analysis With Theme, The Netherlands: Springer.

Santangelo, A., Bortolato, M., Mosher, L. J., Crescimanno, G., Di Giovanni, G., Cassioli, E., Ricca, V. & Casarrubea, M. 2018. Behavioral Fragmentation In The D1ct-7 Mouse Model Of Tourette's Syndrome. Cns Neurosci Ther, 24, 703-711.

Treit, D., Menard, J. & Royan, C. 1993. Anxiogenic Stimuli In The Elevated Plus-Maze. Pharmacology, Biochemistry And Behavior, 44, 463-469.

Supplementary Table 1. ANOVA/ analysis of behaviors in the Hole Board and the Elevated Plus Maze.

		**Frequency of Behaviors in Hole Board**	
	Behavior	Normal distribution	ANOVA<br>Tukey's test / Kruskal-Wallis test
General Exploration			
	Walking		F (3, 44) = 43.9, p<0.0001
	Rearing		F (3, 44) = 7.941, p=0.0002
	Immobile Sniffing		F (3, 44) = 226.4, p<0.0001
	Climbing	No	17.23, p= 0.0006
Focused Exploration			
	Edge Sniff		F (3, 44) = 85.27, p<0.0001
	Head dip	No	32.34, p<0.0001
	HD/ES ratio		F (3, 44) = 23,15, p<0,0001
Immobility			
	Immobility	No	14.70, p= 0.0021
Grooming Activity			
	Front Paw Licking	No	20.06<br>, p=0.0002
	Face Grooming	No	32.23<br>, p<0.0001
	Body Grooming	No	23.42<br>, p<0.0001
	Hind Paw Licking	No	13.62<br>, p=0.0035
		**Duration of Behaviors in Hole Board**	
General Exploration			
	Walking	No	5.982<br>, p=0.1125

	Rearing		F (3, 44) = 2.867, p=0.0472
	Immobile Sniffing		F (3, 44) = 68.15, p<0.0001
	Climbing	No	16.44<br>, p=0.0009
Focused Exploration			
	Edge Sniff		F (3, 44) = 16.27, p<0.0001
	Head dip		F (3, 44) = 27.75, p<0.0001
Immobility			
	Immobility	No	23.41<br>, p<0.0001
Grooming Activity			
	Front Paw Licking	No	20.15<br>, p=0.0002
	Face Grooming	No	16.87<br>, p=0.0008
	Body Grooming	No	33.17<br>, p<0.0001
	Hind Paw Licking	No	27.16<br>, p<0.0001
**T-Patterns in Hole Board**			
	Mean occurrence		F (3, 264) =2.681, p=0.0473
	Mean Lenght		F (3, 264)=20.13, p<0.0001)
**Number of behaviors in Hole Board**			
	Number of Behaviors		F (3, 44) = 115,0 P<0,0001
**Frequency of Behaviors in Elevated Plus Maze**			
Walking Exploration			

	Central Platform Entry		F (3, 44) = 8.633, p=0.0001
	Close Arm Entry		F (3, 44) = 5.136, p=0.0039
	Open Arm Entry		F (3, 44) = 3,958, p=0,0139
	Close Arm Walking		F (3, 44) = 4.349, p=0.0091
	Open Arm Walking	No	13.53<br>, p=0.0036
Vertical Exploration			
	Protected Rearing	No	12.24<br>, p=0.0066
	Unprotected Rearing	No	2.688<br>, p=<br>0.4423
	Protected Head Dip	No	30.54<br>, p<0.0001
	Unprotected Head Dip	No	20.69<br>, p=0.0001
Sniffing Activity			
	Protected Immobile Sniffing		F (3, 44) = 16.67, p<0.0001
	Unprotected Immobile Sniffing		F (3, 44) = 14.96, p<0.0001
	Protected Corner Sniffing		F (3, 44) = 1.009, p=0.3977
	Unprotected Corner Sniffing		F (3, 44) = 9.330, p<0.0001
**Duration of Behaviors in Elevated Plus Maze**			

	Time in Open Arms		F (3, 44) =7.108, p=0.0005
	Time in Close Arms		F (3, 44) = 4.554, p=0.0073
	Behaviors in Open Arms		F (3, 44) = 10.32, p<0.0001
	Behaviors in Close Arms		F (3, 44) = 3.612 P=0.0204
**T-Patterns in Elevated Plus Maze**			
	Mean occurrence		F (3, 10642) = 151.9, p<0.0001
	Mean Length		F (3, 10642) = 769.9, p<0.0001

Supplementary Table 2.

**Frequency of Other Behaviors in the Elevates Plus Maze**			
	Behavior	Normal distribution	ANOVA<br>Tukey's test / Kruskal-Wallis test
Other			
	Protected paw licking	No	3.420<br>, p=0.3313
	Unprotected paw licking	No	9.391<br>, p=0.0245
	Protected Head Dip		F (3, 44) = 29.45, p<0.0001
	Unprotected Head Dip		F (3, 44) = 12.02, p<0.0001
Sniffing Activity			
	Protected Immobile Sniffing		F (3, 44) = 16.67, p<0.0001

	Unprotected Immobile Sniffing		F (3, 44) = 14.96, p<0.0001
	Protected grooming	No	14.45<br>, p=0.0023
	Unprotected grooming	No	6.395<br>, p=0.0939
	Protected defecation	No	24.00<br>, p<0.0001
	Protected defecation	No	20.04<br>, p=0.0002
	Unprotected Corner Sniffing		F (3, 44) = 9.330, p<0.0001
	Protected Immobility	No	16.36<br>, p=0.0010
	Unprotected Immobility		
	Protected Stretched Attend Posture	No	45.78<br>, p<0.0001
	Unprotected Stretched Attend Posture	No	36.47<br>, p<0.0001
	Close arm return	No	27.93<br>, p<0.0001

SUPPLEMENTARY FIGURES

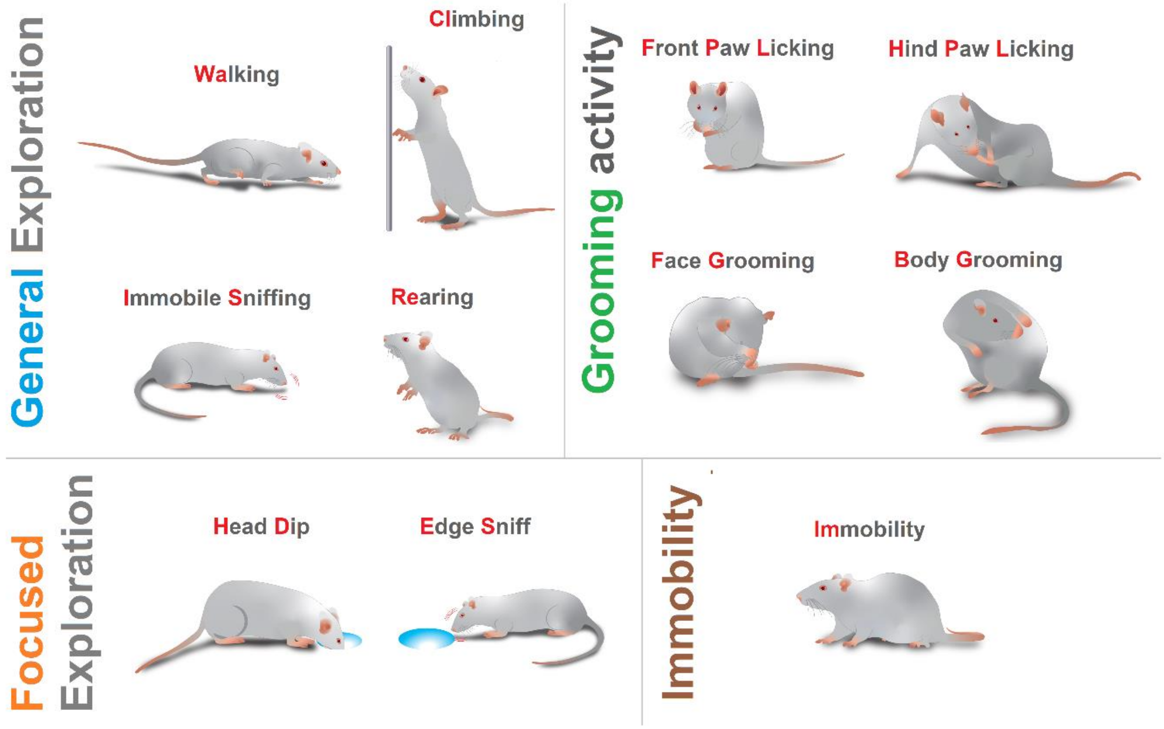

Suppl. Figure 1. Ethogram of behaviours in the Hole Board.

The ethogram is based on the different components of various behavioral categories as analyzed in our previous studies (Casarrubea, Sorbera et al. 2009, Casarrubea, Sorbera et al. 2009, Casarrubea, Faulisi et al. 2017, Casarrubea, Pierucci et al. 2020). In particular, it encompasses eleven behavioral components divided into four main categories. General Exploration encompasses all the activities of environmental exploration, excluding the holes: Walking (Wa), Climbing (Cl), Immobile Sniffing (IS) and Rearing (Re); Focused Exploration contains the components aimed at the exploration of the ground holes: Edge-Sniff (ES) and Head-Dip (HD); Grooming Activity includes Head-, Paws-, Body-, and Tail-self-cleaning behaviors: Front-Paw Licking (FPL), Hind-Paw Licking (HPL), Face Grooming (FG), Body Grooming (BG); and Immobility (Im).

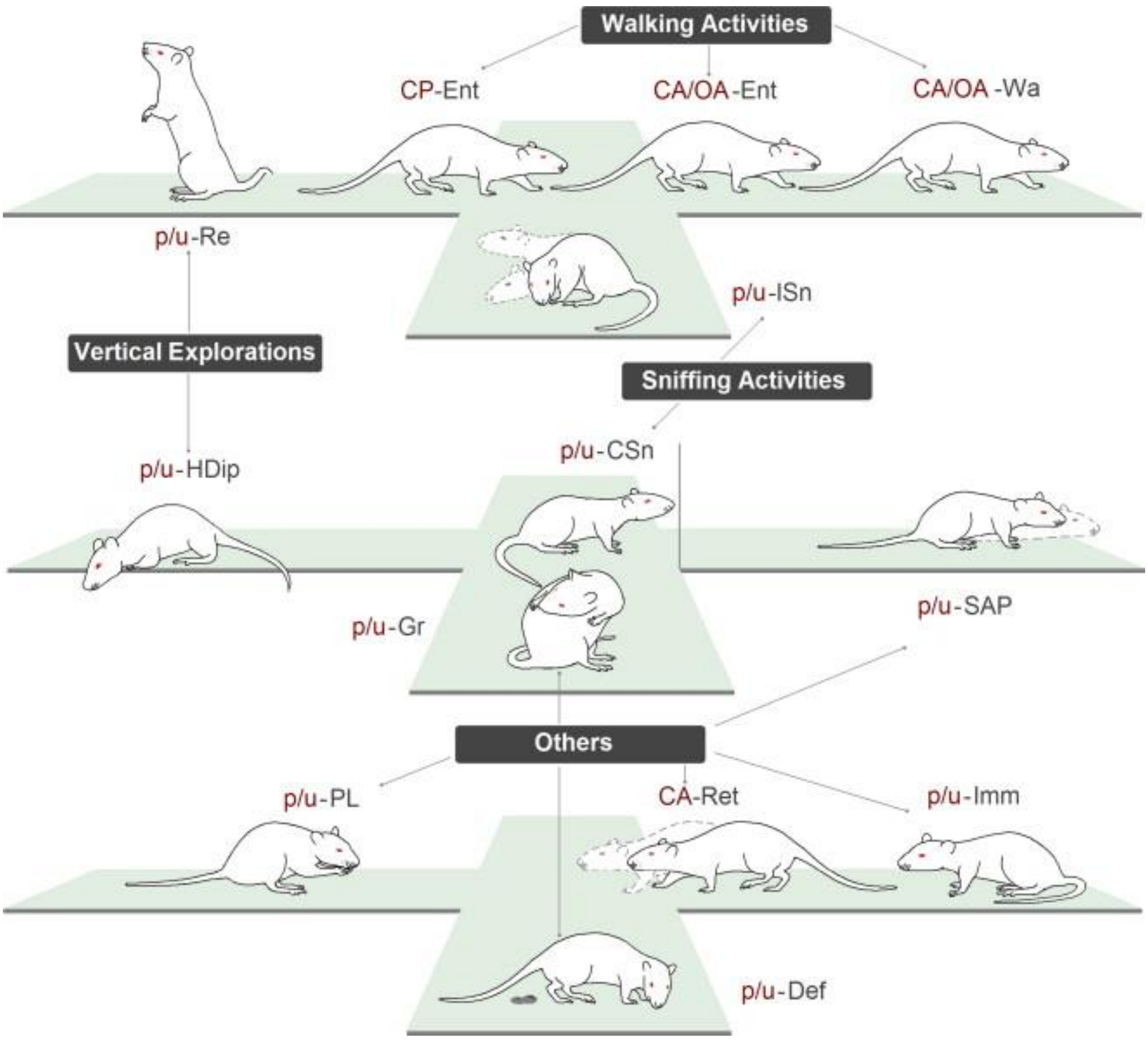

Suppl Fig. 2. Ethogram of rat behavior in the Elevated Plus Maze.

For illustrative purposes the walls of the closed arms are not represented. The behavioral element is considered "protected" (p) if performed in the central platform or in a closed arm; "unprotected" (u) if performed in an open arm. p/u-Re = Rearing: rat maintains an erect posture; CP-Ent = Central Platform Entry: rat moves from an open or closed arm to the central platform; CA/OA-Ent = Closed Arm/Open Arm-Entry: rat moves from the central platform to a closed or to an open arm (all four paws in); CA/OA-Wa = Closed Arm/Open Arm-Walking: the rat walks in a closed or in an open arm; p/u-ISn = Immobile Sniffing: the rat sniffs the surrounding area without walking activity; p/u-HDip = Head Dip: scanning head movement over the sides of the maze in the direction of the floor (specifically for head dip, it is considered "protected" (p) if performed in the central platform or "unprotected" (u) if performed in an open arm); p/u-CSn = Corner Sniffing: rat sniffs the entrance border of a closed arm; p/u-SAP = Stretched Attend Posture: the rat stretches head and shoulders forward and then returns to the original position; p/u-Gr = Grooming: rat licks/rubs its face and/or body; p/u-

PL = Paw Licking: rat licks its paws; CA-Ret = Closed Arm Return: the rat puts its head and forepaws in the central platform and then re-enters rapidly into the closed arm; p/u-Imm = Immobility: an immobile posture is maintained; p/u-Def = Defecation: excrements are released (Casarrubea, Roy et al. 2013).

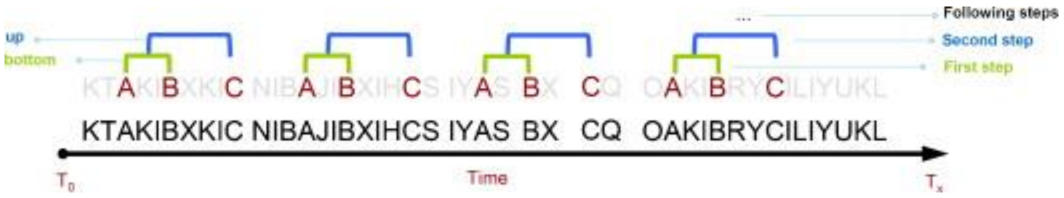

Suppl. Fig. 3. Example of T-Pattern Analysis.

Example of four occurrences of a three-element T-Pattern within a hypothetical data set encompassing 45 events. The algorithm compares the distributions of each pair of the behavioral elements A and B searching for a time window (interval) so that, more often than chance expectation, A is followed by B within that time window. In this case, A and B are by definition a T-Pattern indicated as (A B). In a second step, such first level T-Patterns are marked and considered as potential A or B terms in higher patterns, for example, ((A B) C). Thus, more complex T-Patterns may be created following this hierarchical bottom-up detection process up to any level. When no more T-Patterns are found, the search stops. The comprehensive procedure is called T-Pattern Analysis.

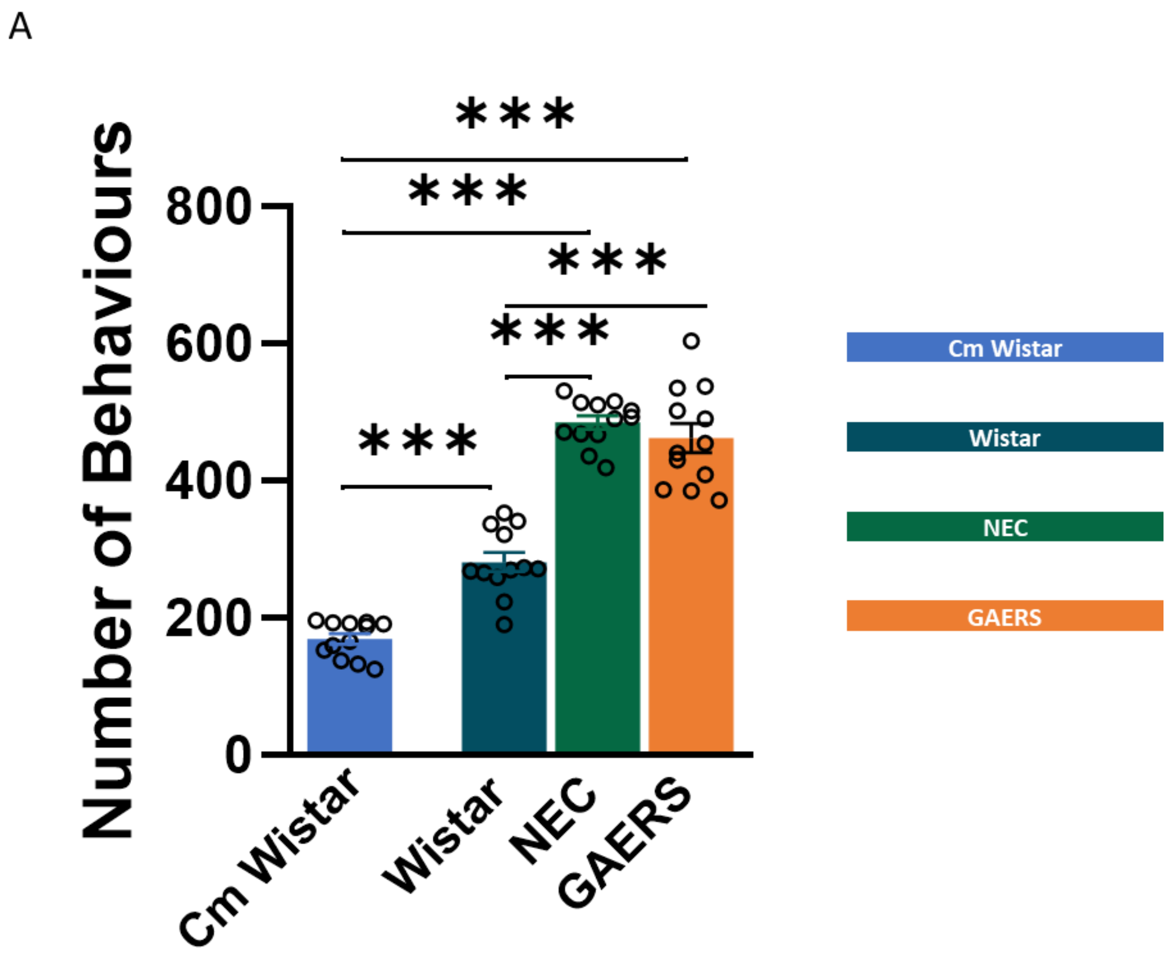

Supplementary Figure 4. The mean number of behaviors ± SEM in HB. *=p<0.05, **= p<0.01, ***= p<0.001; Turkey post-hoc test for multiple comparisons after ANOVA test, n=12 rats in each group.

Cm Wistar

#	Terminal String	Length	Occs
1	(cl cl)	2	240
2	(cl hd)	2	90
3	(cl is)	2	182
4	(cl wa)	2	259
5	(es es)	2	47
6	(es hd)	2	113
7	(es is)	2	67
8	(es wa)	2	103
9	(fg bg)	2	45
10	(fpl bg)	2	25
11	(fpl fg)	2	37
12	(hd cl)	2	89
13	(hd es)	2	87
14	(hd hd)	2	167
15	(hd wa)	2	151
16	(is cl)	2	160
17	(is is)	2	310
18	(is wa)	2	197
19	(re wa)	2	82
20	(wa cl)	2	243
21	(wa is)	2	225
22	(wa wa)	2	402
23	(cl (hd cl))	3	56
24	(cl (is cl))	3	84
25	(cl (wa cl))	3	198
26	(es (hd es))	3	59
27	(fpl (fg bg))	3	29
28	(hd (es is))	3	23
29	(hd (es wa))	3	58
30	(is (cl is))	3	123
31	(is (wa is))	3	154
32	(wa (cl wa))	3	216
33	(wa (is wa))	3	141
34	((cl hd) cl)	3	71
35	((cl is) cl)	3	116
36	((cl wa) cl)	3	235
37	((es hd) es)	3	79
38	((hd cl) hd)	3	74
39	((hd es) hd)	3	59
40	((hd es) wa)	3	59
41	((hd wa) hd)	3	122
42	((is cl) is)	3	144
43	((is wa) is)	3	171
44	((wa cl) wa)	3	236
45	((cl hd)(wa cl))	4	64
46	((es wa)(is cl))	4	46
47	((hd wa)(cl hd))	4	66
48	((is wa)(cl is))	4	97
49	((wa is)(cl wa))	4	101
50	((hd (es wa)) is)	4	22
51	(((hd es) wa) is)	4	24
52	(hd ((es wa)(is cl)))	5	25
53	(((es wa)(is cl))((is wa) is))	7	23

Wistar

#	Terminal String	Length	Occs
1	(cl is)	2	352
2	(cl wa)	2	176
3	(es es)	2	174
4	(es hd)	2	237
5	(hd es)	2	202
6	(hd hd)	2	158
7	(is re)	2	140
8	(is wa)	2	300
9	(re re)	2	97
10	(wa es)	2	153
11	(wa wa)	2	473
12	(es (hd es))	3	174
13	(es (is wa))	3	89
14	(hd (es hd))	3	129
15	(is (wa es))	3	55
16	(wa (es hd))	3	32
17	((cl is) cl)	3	113
18	((cl wa) cl)	3	144
19	((es hd) es)	3	198
20	((hd es) hd)	3	173
21	((is wa) es)	3	60
22	((is wa) hd)	3	58
23	((is wa)(es hd))	4	39
24	((is (wa es)) hd)	4	36
25	(((is wa) es) hd)	4	42

NEC

#	Terminal String	Length	Occs
1	(cl is)	2	438
2	(cl wa)	2	353
3	(es hd)	2	540
4	(es is)	2	771
5	(hd es)	2	84
6	(hd is)	2	530
7	(is es)	2	150
8	(is re)	2	216
9	(is wa)	2	202
10	(re cl)	2	78
11	(re is)	2	359
12	(re re)	2	109
13	(re wa)	2	212
14	(wa es)	2	370
15	(wa hd)	2	248
16	(cl (wa es))	3	117
17	(es (hd es))	3	72
18	(es (is re))	3	58
19	(es (is wa))	3	87
20	(hd (is re))	3	63
21	(is (es is))	3	109
22	(is (re wa))	3	114
23	(is (wa es))	3	222
24	(is (wa hd))	3	182
25	(re (cl is))	3	65
26	(re (cl wa))	3	43
27	(re (is es))	3	60
28	(wa (es hd))	3	236
29	(wa (es is))	3	301
30	(wa (hd is))	3	226
31	(wa (is wa))	3	89
32	((cl is) cl)	3	249
33	((cl is) es)	3	141
34	((cl wa) cl)	3	297
35	((cl wa) es)	3	159
36	((cl wa) hd)	3	95
37	((es hd) es)	3	410
38	((es is) re)	3	140
39	((es is) wa)	3	413
40	((hd is) re)	3	156
41	((hd is) wa)	3	345
42	((is es) hd)	3	105
43	((is re) wa)	3	115
44	((re is) es)	3	73
45	((cl is)(es is))	4	122
46	((cl is)(wa es))	4	89
47	((cl wa)(es hd))	4	108
48	((cl wa)(es is))	4	127
49	((cl wa)(hd is))	4	79
50	((es is)(re wa))	4	76
51	((es is)(wa es))	4	167
52	((hd is)(re is))	4	152
53	((hd is)(re wa))	4	93
54	((hd is)(wa hd))	4	115
55	((is es)(hd is))	4	91
56	((re is)(es is))	4	59
57	(cl (wa (es is)))	4	90
58	(es (hd (is re)))	4	48
59	(es (is (re wa)))	4	42
60	(es (is (wa es)))	4	58
61	(es ((hd is) re))	4	128
62	(es ((is re) wa))	4	43
63	(is (wa (es hd)))	4	159
64	(is (wa (es is)))	4	173
65	(is (wa (hd is)))	4	151
66	(re (is (es is)))	4	37
67	(re ((cl is) es))	4	24
68	(re ((is es) hd))	4	35
69	(wa ((es is) wa))	4	111
70	(wa ((hd is) wa))	4	115
71	((cl (wa es)) hd)	4	98
72	((es (is re)) wa)	4	49
73	((is (es is)) wa)	4	51
74	((is (wa es)) hd)	4	170
75	((re (is es)) hd)	4	41
76	((wa (es is)) cl)	4	98
77	((wa (es is)) wa)	4	204
78	((wa (hd is)) cl)	4	65
79	((wa (hd is)) re)	4	47
80	((wa (hd is)) wa)	4	216
81	(((cl is) es) hd)	4	102
82	(((cl wa) es) hd)	4	128
83	(((es is) wa) es)	4	290
84	(((hd is) wa) hd)	4	239
85	(((re is) es) hd)	4	53
86	(es ((hd is)(re is)))	5	124
87	(es ((hd is)(re wa)))	5	70
88	(re ((is es)(hd is)))	5	32
89	((cl is)(wa (es is)))	5	37
90	((cl is)((es is) wa))	5	77
91	((cl wa)(is (wa es)))	5	40
92	((hd is)(re (is es)))	5	29
93	((hd is)(wa (es hd)))	5	106
94	((hd is)((cl wa) es))	5	34
95	((re is)((hd is) wa))	5	34
96	((wa es)((hd is) re))	5	64
97	((is (wa es))(hd is))	5	153
98	((re (is es))(hd is))	5	37
99	((wa (es is))(cl wa))	5	62
100	((wa (hd is))(cl is))	5	56
101	((wa (hd is))(cl wa))	5	89
102	((wa (hd is))(re is))	5	44
103	((wa (hd is))(re wa))	5	25
104	(((cl is) es)(hd is))	5	87
105	(((hd is) re)(is es))	5	32
106	(((hd is) wa)(es hd))	5	141
107	(((re is) es)(hd is))	5	48
108	(es (is (wa (es hd))))	5	43
109	(es ((is (wa es)) hd))	5	43
110	(wa ((is (wa es)) hd))	5	74
111	((es (is (wa es))) hd)	5	45
112	((wa es)((hd is)(re is)))	6	50
113	((re (is es))((hd is) wa))	6	29
114	(((cl is) es)((hd is) wa))	6	66
115	(((es is) wa)(is (wa es)))	6	57
116	(((hd is) wa)(is (wa es)))	6	46
117	(es ((is (wa es))(hd is)))	6	36
118	(wa ((is (wa es))(hd is)))	6	63
119	((cl wa)(is (wa (es hd))))	6	18
120	((cl wa)((is (wa es)) hd))	6	31
121	((es (is (wa es)))(hd is))	6	39
122	(((cl wa)(is (wa es))) hd)	6	29
123	(((is (wa es))(hd is)) re)	6	36
124	(((hd is)(re is))((hd is) wa))	7	27
125	(es (((hd is) wa)(is (wa es))))	7	41
126	((es is)((re is)((hd is) wa)))	7	23
127	((hd is)((re is)((hd is) wa)))	7	22
128	((es ((hd is)(re is)))(hd is))	7	21
129	(((is (wa es))(hd is))(re is))	7	34
130	(((is (wa es))(hd is))(re wa))	7	24
131	(es (((hd is)(re is))((hd is) wa)))	8	21
132	((es ((hd is)(re is)))((hd is) wa))	8	23
133	(es ((hd is)((re is)((hd is) wa))))	8	18

GAERS

#	Terminal String	Length	Occs
1	(cl cl)	2	343
2	(cl is)	2	429
3	(cl wa)	2	248
4	(es es)	2	380
5	(es hd)	2	321
6	(hd es)	2	296
7	(hd hd)	2	253
8	(hd is)	2	214
9	(is wa)	2	206
10	(re cl)	2	145
11	(re is)	2	358
12	(re re)	2	320
13	(re wa)	2	188
14	(wa es)	2	385
15	(wa hd)	2	138
16	(wa is)	2	382
17	(wa wa)	2	387
18	(hd (es hd))	3	219
19	(is (wa es))	3	215
20	(wa (es hd))	3	156
21	(wa (hd is))	3	88
22	(wa (is wa))	3	113
23	((cl is) cl)	3	307
24	((cl is) es)	3	119
25	((cl wa) cl)	3	217
26	((cl wa) es)	3	101
27	((es hd) es)	3	305
28	((hd es) hd)	3	236
29	((hd is) hd)	3	58
30	((hd is) wa)	3	133
31	((is wa) is)	3	205
32	((re is) re)	3	282
33	((wa es) hd)	3	177
34	((cl is)(wa wa))	4	71
35	((hd is)(es hd))	4	32
36	((hd is)(wa hd))	4	71
37	((wa es)(hd is))	4	132
38	(is ((wa es) hd))	4	70
39	((is (wa es)) hd)	4	110
40	(((cl is) es) hd)	4	66
41	(((cl wa) es) hd)	4	53
42	(((hd is) wa) hd)	4	70
43	(is ((wa es)(hd is)))	5	45
44	((hd is)(wa (es hd)))	5	77
45	((hd is)((wa es) hd))	5	84
46	((wa es)((hd is) wa))	5	87
47	((is (wa es))(hd is))	5	81
48	(((cl is) es)(hd is))	5	61
49	(((cl is)(wa wa)) cl)	5	58
50	(((cl wa) es)(hd es))	5	47
51	(((cl wa) es)(hd is))	5	47
52	(((wa es)(hd is)) wa)	5	124
53	(is (((wa es)(hd is)) wa))	6	43
54	((is ((wa es)(hd is))) cl)	6	33
55	((((cl wa) es)(hd is)) cl)	6	35
56	((is ((wa es)(hd is)))(cl is))	7	32
57	(cl ((is ((wa es)(hd is))) cl))	7	16

Supplementary Figure 5. The length distribution of T-patterns (that is, the number of T-patterns of different lengths detected for each group) in THE Hole Board.

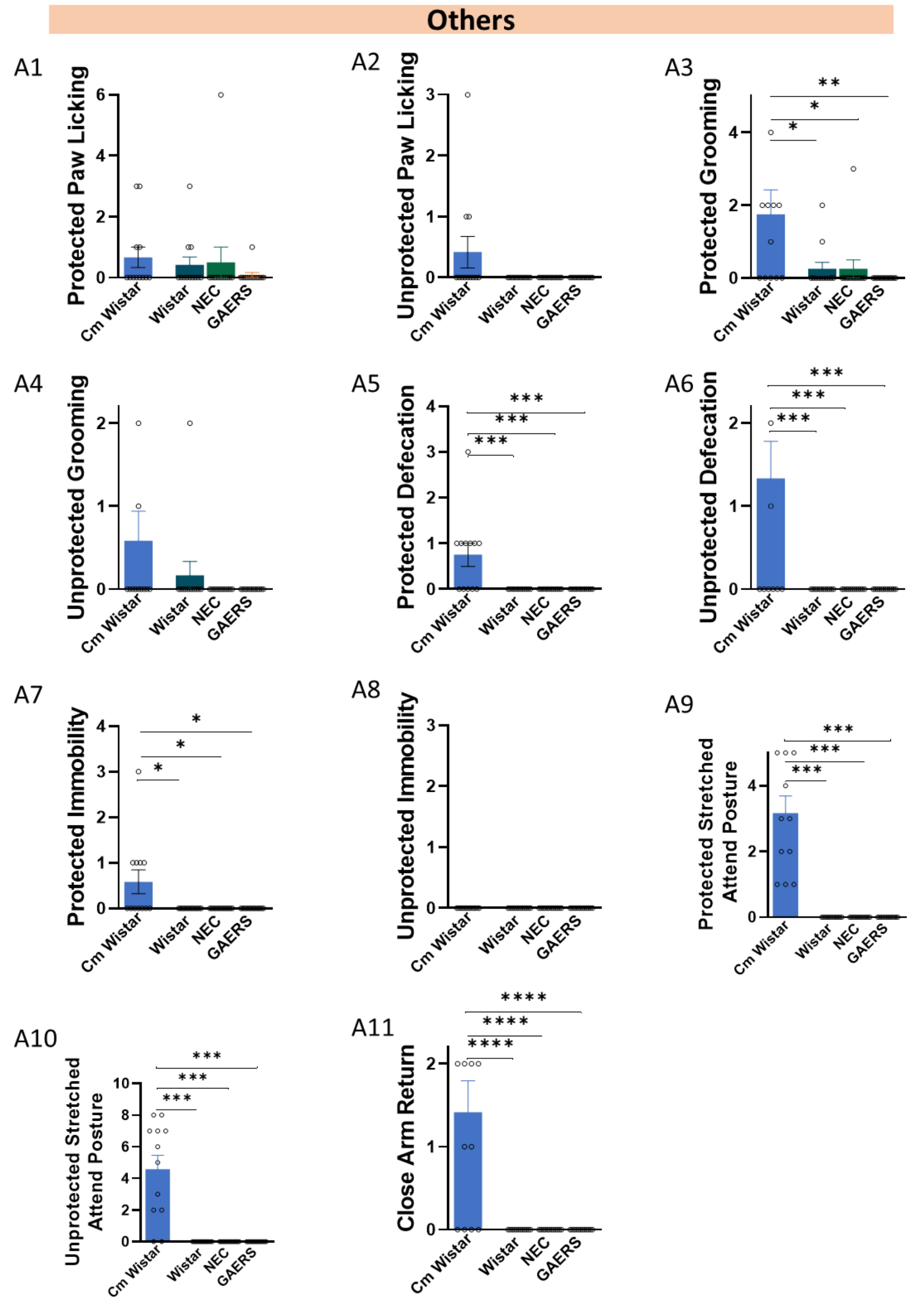

Supplementary Figure 6. Mean frequency ± SEM of each component of the behavioral repertoire in EPM classified as others in Cm Wistar, Wistar, NEC and GAERS RATS. . *=p<0.05, **= p<0.01, ***= p<0.001; Turkey post-hoc test for multiple comparisons after

404	((ca_wa (p_csn cp_ent))(oa_ent u_hdip))	33	5
405	((ca_wa (p_csn cp_ent))(oa_ent u_isn))	39	5
406	((ca_wa (p_csn cp_ent))(p_csn oa_ent))	23	5
407	((oa_ent (oa_wa u_hdip))(oa_wa cp_ent))	43	5
408	((oa_ent (oa_wa u_hdip))(oa_wa u_csn))	56	5
409	((oa_ent (oa_wa u_hdip))(u_csn cp_ent))	59	5
410	((oa_ent (u_isn u_hdip))(oa_wa u_csn))	58	5
411	((oa_wa (u_csn cp_ent))(ca_wa ca_wa))	32	5
412	((oa_wa (u_csn cp_ent))(ca_wa p_re))	35	5
413	((oa_wa (u_csn cp_ent))(oa_ent oa_wa))	66	5
414	((oa_wa (u_csn cp_ent))(p_isn ca_ent))	33	5
415	((oa_wa (u_csn cp_ent))(p_isn ca_wa))	31	5
416	((oa_wa (u_csn cp_ent))(p_isn p_re))	32	5
417	((p_csn (ca_ent ca_wa))(p_isn p_re))	85	5
418	((p_csn (ca_wa p_re))(ca_wa p_csn))	60	5
419	((p_csn (ca_wa p_re))(p_csn cp_ent))	46	5
420	((p_csn (ca_wa p_re))(p_isn p_csn))	53	5
421	((p_csn (oa_ent u_hdip))(oa_wa u_hdip))	32	5
422	((p_csn (oa_ent u_hdip))(u_isn oa_wa))	54	5
423	((p_csn (oa_ent u_isn))(u_hdip u_hdip))	59	5
424	((p_csn (p_isn ca_ent))(ca_wa p_re))	73	5
425	((p_csn (p_isn ca_ent))(p_isn p_re))	72	5
426	((p_csn (p_isn ca_wa))(p_isn p_re))	68	5
427	((p_csn (p_isn oa_ent))(oa_wa u_hdip))	46	5
428	((p_re (ca_wa p_csn))(cp_ent oa_ent))	38	5
429	((p_re (ca_wa p_csn))(oa_ent u_hdip))	32	5
430	((p_re (ca_wa p_csn))(oa_ent u_isn))	39	5
431	((p_re (ca_wa p_csn))(p_isn cp_ent))	51	5
432	((u_csn (ca_ent ca_wa))(p_isn ca_wa))	38	5
433	((u_csn (ca_ent ca_wa))(p_isn p_re))	42	5
434	((u_csn (ca_ent ca_wa))(p_re p_csn))	34	5
435	((u_csn (ca_ent ca_wa))(p_re p_re))	33	5
436	((u_csn (cp_ent ca_wa))(ca_wa p_csn))	28	5
437	((u_csn (cp_ent ca_wa))(p_isn ca_wa))	32	5
438	((u_csn (cp_ent ca_wa))(p_isn p_re))	36	5
439	((u_csn (cp_ent ca_wa))(p_re ca_wa))	37	5
440	((u_csn (cp_ent ca_wa))(p_re p_csn))	29	5
441	((u_csn (oa_ent oa_wa))(u_isn u_hdip))	34	5
442	((u_csn (oa_ent u_hdip))(u_isn u_hdip))	31	5
443	((u_csn (oa_ent u_isn))(u_hdip u_hdip))	32	5
444	((u_csn (p_csn ca_wa))(ca_wa p_csn))	19	5
445	((u_csn (p_csn ca_wa))(p_re ca_wa))	24	5
446	((u_csn (p_isn ca_ent))(ca_wa ca_wa))	36	5
447	((u_csn (p_isn ca_ent))(ca_wa p_re))	36	5
448	((u_csn (p_isn ca_ent))(p_isn ca_wa))	28	5
449	((u_csn (p_isn ca_ent))(p_isn p_re))	35	5
450	((u_csn (p_isn ca_ent))(p_re ca_wa))	36	5
451	((u_csn (p_isn ca_ent))(p_re p_csn))	27	5
452	((u_csn (p_isn ca_ent))(p_re p_re))	28	5
453	((u_csn (u_isn cp_ent))(ca_ent p_isn))	32	5
454	((u_csn (u_isn cp_ent))(ca_ent ca_wa))	34	5
455	((u_csn (u_isn cp_ent))(ca_ent p_re))	29	5
456	((u_csn (u_isn cp_ent))(ca_wa ca_wa))	23	5
457	((u_csn (u_isn cp_ent))(ca_wa p_re))	26	5
458	((u_csn (u_isn cp_ent))(oa_ent oa_wa))	43	5
459	((u_csn (u_isn cp_ent))(p_isn ca_ent))	29	5
460	((u_csn (u_isn cp_ent))(p_isn ca_wa))	24	5
461	((u_hdip (oa_wa u_csn))(cp_ent ca_ent))	30	5
462	((u_hdip (oa_wa u_csn))(ca_ent p_isn))	30	5
463	((u_hdip (oa_wa u_csn))(ca_ent ca_wa))	30	5
464	((u_hdip (oa_wa u_csn))(cp_ent ca_wa))	25	5
465	((u_hdip (oa_wa u_csn))(cp_ent oa_ent))	44	5
466	((u_hdip (oa_wa u_csn))(oa_ent u_hdip))	44	5
467	((u_hdip (oa_wa u_csn))(oa_ent u_isn))	28	5
468	((u_hdip (oa_wa u_csn))(u_isn cp_ent))	39	5
469	((u_hdip (u_csn cp_ent))(ca_ent p_isn))	34	5
470	((u_hdip (u_csn cp_ent))(ca_ent ca_wa))	34	5
471	((u_hdip (u_csn cp_ent))(oa_ent oa_wa))	51	5
472	((u_hdip (u_csn cp_ent))(oa_ent u_hdip))	61	5
473	((u_hdip (u_csn cp_ent))(oa_wa u_hdip))	27	5
474	((u_hdip (u_csn cp_ent))(p_csn ca_ent))	26	5
475	((u_hdip (u_csn cp_ent))(p_isn ca_ent))	29	5
476	((u_hdip (u_csn cp_ent))(p_isn ca_wa))	26	5
477	((u_hdip (u_isn u_csn))(cp_ent ca_ent))	33	5
478	((u_hdip (u_isn u_csn))(ca_ent p_isn))	33	5
479	((u_hdip (u_isn u_csn))(ca_ent ca_wa))	33	5
480	((u_hdip (u_isn u_csn))(cp_ent ca_wa))	28	5
481	((u_hdip (u_isn u_csn))(u_isn cp_ent))	43	5
482	(((ca_ent ca_wa) cp_ent)(oa_ent oa_wa))	48	5
483	(((ca_ent ca_wa) cp_ent)(oa_ent u_hdip))	49	5
484	(((ca_ent ca_wa) cp_ent)(oa_ent u_isn))	55	5
485	(((ca_ent ca_wa) cp_ent)(p_csn oa_ent))	34	5
486	(((ca_ent ca_wa) cp_ent)(p_isn oa_ent))	36	5
487	(((ca_ent ca_wa) p_csn)(cp_ent oa_ent))	37	5
488	(((ca_ent ca_wa) p_csn)(oa_ent oa_wa))	37	5
489	(((ca_ent ca_wa) p_csn)(oa_ent u_hdip))	39	5
490	(((ca_ent ca_wa) p_csn)(oa_ent u_isn))	47	5
491	(((ca_ent ca_wa) p_csn)(p_isn oa_ent))	31	5
492	(((ca_ent ca_wa) p_re)(cp_ent oa_ent))	45	5
493	(((ca_ent ca_wa) p_re)(p_csn cp_ent))	60	5
494	(((ca_ent ca_wa) p_re)(p_csn oa_ent))	40	5
495	(((ca_ent ca_wa) p_re)(p_isn p_csn))	84	5
496	(((ca_ent ca_wa)(p_csn cp_ent)) oa_ent)	38	5
497	(((ca_ent p_isn)(ca_wa p_csn)) oa_ent)	34	5
498	(((ca_ent p_isn)(p_csn cp_ent)) oa_ent)	35	5
499	(((ca_ent p_re) p_csn)(oa_ent oa_wa))	36	5
500	(((ca_ent p_re) p_csn)(oa_ent u_hdip))	38	5
501	(((ca_ent p_re) p_csn)(oa_ent u_isn))	46	5
502	(((ca_ent p_re) p_csn)(p_isn oa_ent))	30	5
503	(((ca_ent p_re)(cp_ent oa_ent)) oa_wa)	30	5
504	(((ca_wa ca_wa)(cp_ent oa_ent)) oa_wa)	31	5
505	(((ca_wa ca_wa)(p_csn oa_ent)) oa_wa)	30	5
506	(((ca_wa ca_wa)(p_isn cp_ent)) oa_ent)	27	5
507	(((ca_wa p_csn) oa_ent)(u_hdip u_hdip))	31	5
508	(((ca_wa p_csn) oa_ent)(u_isn u_hdip))	32	5
509	(((ca_wa p_csn)(p_isn cp_ent)) oa_ent)	24	5
510	(((ca_wa p_re) ca_wa)(cp_ent oa_ent))	55	5
511	(((ca_wa p_re) ca_wa)(oa_ent u_isn))	43	5
512	(((ca_wa p_re) ca_wa)(p_csn oa_ent))	48	5
513	(((ca_wa p_re) ca_wa)(p_isn cp_ent))	61	5
514	(((ca_wa p_re) cp_ent)(oa_ent oa_wa))	44	5
515	(((ca_wa p_re) cp_ent)(oa_ent u_hdip))	45	5
516	(((ca_wa p_re) cp_ent)(oa_ent u_isn))	50	5
517	(((ca_wa p_re) cp_ent)(p_isn oa_ent))	29	5
518	(((ca_wa p_re)(p_csn cp_ent)) oa_ent)	39	5
519	(((ca_wa p_re)(p_isn cp_ent)) oa_ent)	29	5
520	(((p_re ca_wa)(p_isn cp_ent)) oa_ent)	29	5
521	(((cp_ent ca_ent) cp_ent)(oa_ent oa_wa))	46	5
522	(((cp_ent ca_ent) cp_ent)(oa_ent u_hdip))	47	5
523	(((cp_ent ca_ent) cp_ent)(oa_ent u_isn))	53	5
524	(((cp_ent ca_ent) cp_ent)(p_isn oa_ent))	36	5
525	(((cp_ent ca_wa) cp_ent)(oa_ent oa_wa))	45	5
526	(((cp_ent ca_wa) cp_ent)(oa_ent u_hdip))	46	5
527	(((cp_ent ca_wa) cp_ent)(oa_ent u_isn))	52	5
528	(((cp_ent ca_wa) cp_ent)(p_isn oa_ent))	34	5
529	(((cp_ent oa_ent) oa_wa)(oa_wa cp_ent))	50	5
530	(((cp_ent oa_ent) oa_wa)(u_csn cp_ent))	67	5
531	(((cp_ent oa_ent) oa_wa)(u_hdip u_csn))	51	5
532	(((cp_ent oa_ent) u_hdip)(oa_wa u_csn))	62	5
533	(((cp_ent oa_ent) u_hdip)(u_csn cp_ent))	62	5
534	(((cp_ent oa_ent)(oa_wa u_hdip)) u_csn)	63	5
535	(((oa_ent oa_wa) u_csn)(cp_ent oa_ent))	44	5
536	(((oa_ent oa_wa) u_csn)(u_isn cp_ent))	50	5
537	(((oa_ent oa_wa) u_hdip)(oa_wa u_csn))	55	5
538	(((oa_ent oa_wa) u_hdip)(u_csn cp_ent))	58	5
539	(((oa_ent oa_wa)(u_csn cp_ent)) oa_ent)	54	5
540	(((oa_ent u_hdip)(oa_wa u_csn)) oa_ent)	26	5
541	(((oa_ent u_isn) u_hdip)(oa_wa u_csn))	62	5
542	(((oa_wa u_csn) p_csn)(ca_ent p_isn))	33	5
543	(((oa_wa u_csn) p_csn)(ca_ent ca_wa))	33	5
544	(((oa_wa u_csn) p_csn)(ca_wa ca_wa))	28	5
545	(((oa_wa u_csn) p_csn)(p_isn ca_ent))	28	5
546	(((oa_wa u_hdip)(oa_wa u_csn)) oa_ent)	35	5
547	(((p_csn cp_ent) oa_ent)(u_isn u_hdip))	33	5
548	(((p_csn oa_ent) u_hdip)(oa_wa u_hdip))	35	5
549	(((p_csn oa_ent) u_hdip)(u_isn oa_wa))	56	5
550	(((p_isn ca_wa) p_csn)(oa_ent oa_wa))	25	5
551	(((p_isn ca_wa) p_csn)(oa_ent u_hdip))	32	5
552	(((p_isn ca_wa) p_csn)(oa_ent u_isn))	32	5
553	(((p_isn ca_wa) p_csn)(p_isn oa_ent))	24	5
554	(((p_isn ca_wa)(p_isn cp_ent)) oa_ent)	25	5
555	(((p_isn oa_ent) oa_wa)(u_csn cp_ent))	46	5
556	(((p_isn oa_ent)(oa_wa u_hdip)) u_csn)	47	5
557	(((p_isn p_re)(p_csn cp_ent)) oa_ent)	38	5
558	(((p_isn p_re)(p_isn cp_ent)) oa_ent)	28	5
559	(((p_re ca_wa) oa_ent)(oa_wa u_hdip))	33	5
560	(((p_re ca_wa) oa_ent)(u_hdip u_hdip))	37	5
561	(((p_re ca_wa) oa_ent)(u_isn u_hdip))	37	5
562	(((p_re ca_wa) p_csn)(cp_ent oa_ent))	40	5
563	(((p_re ca_wa) p_csn)(oa_ent oa_wa))	38	5
564	(((p_re ca_wa) p_csn)(oa_ent u_hdip))	40	5
565	(((p_re ca_wa) p_csn)(oa_ent u_isn))	49	5
566	(((p_re ca_wa) p_csn)(p_isn cp_ent))	53	5
567	(((p_re ca_wa) p_csn)(p_isn oa_ent))	33	5
568	(((u_csn cp_ent) oa_ent)(oa_wa u_hdip))	62	5
569	(((u_csn cp_ent) oa_ent)(u_isn u_hdip))	60	5
570	(((u_csn cp_ent) p_csn)(ca_ent p_isn))	37	5
571	(((u_csn cp_ent) p_csn)(ca_ent ca_wa))	37	5
572	(((u_csn cp_ent) p_csn)(ca_wa ca_wa))	32	5
573	(((u_csn cp_ent) p_csn)(p_isn ca_ent))	31	5
574	(((u_csn cp_ent) p_csn)(p_isn ca_wa))	25	5
575	(((u_csn cp_ent)(oa_ent oa_wa)) u_csn)	55	5
576	(((u_hdip oa_wa) u_csn)(cp_ent ca_ent))	33	5
577	(((u_hdip oa_wa) u_csn)(ca_ent p_isn))	33	5
578	(((u_hdip oa_wa) u_csn)(ca_ent ca_wa))	33	5
579	(((u_hdip oa_wa) u_csn)(ca_ent p_re))	31	5
580	(((u_hdip oa_wa) u_csn)(cp_ent ca_wa))	28	5
581	(((u_isn cp_ent) ca_ent)(ca_wa ca_wa))	31	5
582	(((u_isn cp_ent) ca_ent)(p_isn p_re))	30	5
583	(((u_isn cp_ent) oa_ent)(oa_wa u_csn))	29	5
584	(((u_isn cp_ent) oa_ent)(oa_wa u_hdip))	29	5
585	(((u_isn cp_ent) oa_ent)(u_isn u_hdip))	29	5
586	(((u_isn cp_ent)(oa_ent oa_wa)) u_csn)	35	5
587	(((u_isn oa_wa) u_csn)(ca_ent ca_wa))	32	5
588	(((u_isn oa_wa) u_csn)(cp_ent ca_ent))	32	5
589	(((u_isn oa_wa) u_csn)(cp_ent ca_wa))	27	5
590	(((u_isn oa_wa) u_hdip)(oa_wa u_csn))	53	5
591	(((u_isn u_csn)(u_isn cp_ent)) ca_ent)	30	5
592	(((u_isn u_hdip)(oa_wa u_csn)) oa_ent)	26	5
593	(p_csn ((oa_ent (oa_wa u_hdip)) u_csn))	37	5
594	(p_csn (((ca_ent ca_wa) cp_ent) oa_ent))	36	5
595	(p_csn (((p_isn oa_ent) oa_wa) u_csn))	33	5
596	(((oa_ent (oa_wa u_hdip)) u_csn) oa_ent)	39	5
597	(((oa_wa (u_csn cp_ent)) p_csn) ca_ent)	35	5
598	(((p_csn (ca_wa p_re)) p_csn) oa_ent)	32	5
599	(((p_csn (p_isn oa_ent)) oa_wa) u_csn)	35	5
600	((((oa_ent oa_wa) u_hdip) u_csn) oa_ent)	38	5
601	(((ca_ent ca_wa) p_re)(p_csn (cp_ent oa_ent)))	32	6
602	(((ca_ent ca_wa) p_re)((ca_wa p_csn) oa_ent))	34	6
603	(((ca_ent ca_wa) p_re)((p_csn cp_ent) oa_ent))	36	6
604	((ca_ent ca_wa)((p_isn ca_wa)(p_csn cp_ent)))	53	6
605	((ca_ent ca_wa)((p_isn ca_wa)(p_csn p_csn)))	49	6
606	((ca_ent ca_wa)((p_re ca_wa)(cp_ent oa_ent)))	50	6
607	((ca_ent ca_wa)((p_re ca_wa)(p_csn cp_ent)))	58	6
608	((ca_ent ca_wa)((p_re ca_wa)(p_csn oa_ent)))	47	6
609	((ca_ent p_isn)((p_re ca_wa)(cp_ent oa_ent)))	50	6
610	((ca_ent p_isn)((p_re ca_wa)(p_csn cp_ent)))	56	6
611	((ca_ent p_isn)((p_re ca_wa)(p_csn oa_ent)))	47	6
612	(((ca_ent ca_wa) p_re)((p_csn cp_ent) p_csn))	39	6
613	((ca_wa ca_wa)((p_csn cp_ent)(oa_ent u_isn)))	39	6
614	((ca_wa ca_wa)((p_csn cp_ent)(oa_ent oa_wa)))	32	6
615	((ca_wa ca_wa)((p_csn cp_ent)(oa_ent u_hdip)))	33	6
616	((ca_wa ca_wa)((p_csn cp_ent)(p_csn oa_ent)))	23	6
617	((ca_wa ca_wa)((p_isn p_csn)(cp_ent oa_ent)))	29	6
618	((ca_wa p_csn)((p_isn cp_ent)(oa_ent u_isn)))	24	6
619	((ca_wa p_re)((ca_wa p_csn)(cp_ent oa_ent)))	38	6
620	((ca_wa p_re)((ca_wa p_csn)(oa_ent u_hdip)))	32	6
621	((ca_wa p_re)((ca_wa p_csn)(oa_ent u_isn)))	35	6
622	((ca_wa p_re)((ca_wa p_csn)(p_isn cp_ent)))	51	6
623	((ca_wa p_re)((p_csn cp_ent)(oa_ent u_isn)))	38	6
624	((ca_wa p_re)((p_csn cp_ent)(oa_ent oa_wa)))	32	6
625	((ca_wa p_re)((p_csn cp_ent)(oa_ent u_hdip)))	33	6
626	((ca_wa p_re)((p_csn oa_ent)(oa_wa u_hdip)))	30	6
627	((ca_wa p_re)((p_csn oa_ent)(u_isn u_hdip)))	34	6
628	((ca_wa p_re)((p_isn p_csn)(cp_ent oa_ent)))	29	6
629	((cp_ent ca_ent)((ca_wa ca_wa)(cp_ent oa_ent)))	42	6
630	((cp_ent ca_ent)((ca_wa ca_wa)(p_csn oa_ent)))	36	6
631	((cp_ent ca_ent)((ca_wa ca_wa)(p_isn cp_ent)))	55	6
632	((cp_ent ca_ent)((ca_wa p_csn)(p_isn cp_ent)))	47	6
633	((cp_ent ca_ent)((ca_wa p_re)(p_csn cp_ent)))	58	6
634	((cp_ent ca_ent)((ca_wa p_re)(p_csn oa_ent)))	38	6
635	((cp_ent ca_ent)((ca_wa p_re)(p_isn cp_ent)))	58	6
636	((cp_ent ca_ent)((ca_wa (p_csn cp_ent)) oa_ent))	34	6
637	((cp_ent ca_ent)((p_isn ca_wa)(p_csn cp_ent)))	49	6
638	((cp_ent ca_ent)((p_re ca_wa)(cp_ent oa_ent)))	47	6
639	((cp_ent ca_ent)((p_re ca_wa)(p_csn cp_ent)))	55	6
640	((cp_ent ca_ent)((p_re ca_wa)(p_csn oa_ent)))	44	6
641	((cp_ent ca_ent)((p_re ca_wa)(p_isn cp_ent)))	57	6
642	((cp_ent ca_wa)((ca_wa p_csn)(oa_ent u_isn)))	30	6
643	((cp_ent ca_wa)((ca_wa p_csn)(p_isn cp_ent)))	40	6
644	((cp_ent ca_wa)((p_isn ca_wa)(p_csn cp_ent)))	49	6
645	((cp_ent ca_wa)((p_re ca_wa)(cp_ent oa_ent)))	44	6
646	((cp_ent ca_wa)((p_re ca_wa)(p_csn cp_ent)))	54	6
647	((cp_ent ca_wa)((p_re ca_wa)(p_csn oa_ent)))	42	6
648	((cp_ent oa_ent)((oa_wa u_hdip)(oa_wa u_csn)))	50	6
649	((oa_ent oa_wa)((u_isn u_hdip)(oa_wa u_csn)))	41	6
650	((oa_wa oa_wa)((u_csn cp_ent)(ca_ent p_isn)))	25	6
651	((oa_wa oa_wa)((u_csn cp_ent)(p_csn ca_wa)))	19	6
652	((oa_wa oa_wa)((u_csn cp_ent)(p_isn ca_ent)))	21	6
653	((oa_wa oa_wa)((u_csn cp_ent)(ca_ent ca_wa)))	28	6
654	((oa_wa oa_wa)((u_csn cp_ent)(ca_wa ca_wa)))	24	6
655	((oa_wa oa_wa)((u_csn cp_ent)(ca_wa p_re)))	21	6
656	((oa_wa oa_wa)((u_csn cp_ent)(p_isn ca_wa)))	25	6
657	((oa_wa oa_wa)((u_csn cp_ent)(p_isn p_re)))	20	6
658	((oa_wa (u_csn cp_ent))(p_csn (ca_ent ca_wa)))	32	6
659	((oa_wa (u_csn cp_ent))((ca_ent ca_wa) p_re))	37	6
660	((oa_wa u_csn)((u_isn cp_ent)(ca_ent p_isn)))	31	6
661	((oa_wa u_csn)((u_isn cp_ent)(ca_ent ca_wa)))	31	6
662	((oa_wa u_csn)((u_isn cp_ent)(ca_wa ca_wa)))	22	6
663	((oa_wa u_csn)((u_isn cp_ent)(ca_wa p_re)))	25	6
664	((oa_wa u_hdip)((u_csn cp_ent)(p_csn ca_ent)))	26	6
665	((oa_wa u_hdip)((oa_wa u_csn)(cp_ent oa_ent)))	37	6
666	((oa_wa u_hdip)((oa_wa u_csn)(oa_ent oa_wa)))	42	6
667	((oa_wa u_hdip)((u_csn cp_ent)(oa_ent oa_wa)))	53	6
668	((oa_wa u_hdip)((u_isn u_csn)(u_isn cp_ent)))	38	6
669	((oa_wa u_hdip)((u_isn u_hdip)(oa_wa u_csn)))	38	6
670	((p_csn ca_ent)((ca_wa ca_wa)(cp_ent oa_ent)))	34	6
671	((p_csn (ca_ent ca_wa))((p_re ca_wa) oa_ent))	31	6
672	((p_csn ca_ent)((p_re ca_wa)(cp_ent oa_ent)))	35	6
673	((p_csn ca_wa)((p_re ca_wa)(p_csn cp_ent)))	40	6
674	((p_csn ca_wa)((p_re ca_wa)(p_csn oa_ent)))	33	6
675	((p_csn oa_ent)((oa_wa u_hdip)(oa_wa u_csn)))	25	6
676	(((p_isn ca_ent)(ca_wa p_re))(ca_wa p_csn))	70	6
677	((p_isn ca_ent)((ca_wa ca_wa)(cp_ent oa_ent)))	36	6
678	((p_isn ca_ent)((ca_wa ca_wa)(p_csn oa_ent)))	32	6
679	((p_isn ca_ent)((ca_wa p_re)(p_csn cp_ent)))	48	6
680	((p_isn ca_ent)((ca_wa p_re)(p_csn oa_ent)))	34	6
681	((p_isn ca_ent)((p_isn ca_wa)(p_csn cp_ent)))	40	6
682	((p_isn ca_ent)((p_isn ca_wa)(p_csn p_csn)))	39	6
683	((p_isn ca_ent)((p_re ca_wa)(cp_ent oa_ent)))	43	6
684	((p_isn ca_wa)((p_csn cp_ent)(oa_ent u_isn)))	38	6
685	((p_isn ca_wa)((p_csn cp_ent)(oa_ent oa_wa)))	31	6
686	((p_isn ca_wa)((p_csn cp_ent)(oa_ent u_hdip)))	32	6
687	((p_isn ca_wa)((p_isn p_csn)(cp_ent oa_ent)))	29	6
688	((p_isn ca_wa)((p_re ca_wa)(cp_ent oa_ent)))	48	6
689	((p_isn ca_wa)((p_re ca_wa)(p_csn oa_ent)))	43	6
690	((p_isn oa_ent)((oa_wa u_hdip)(oa_wa u_csn)))	39	6
691	((p_isn p_csn)((p_isn ca_ent)(ca_wa p_re)))	48	6
692	((p_isn p_re)((ca_wa p_csn)(cp_ent oa_ent)))	37	6
693	((p_isn p_re)((ca_wa p_csn)(oa_ent u_hdip)))	31	6
694	((p_isn p_re)((ca_wa p_csn)(oa_ent u_isn)))	34	6
695	((p_isn p_re)((ca_wa p_csn)(p_isn cp_ent)))	50	6
696	((p_isn p_re)((p_isn p_csn)(p_isn oa_ent)))	28	6
697	((p_re ca_wa)((cp_ent oa_ent)(oa_wa u_hdip)))	41	6
698	((p_re ca_wa)((cp_ent oa_ent)(u_isn u_hdip)))	38	6
699	((p_re ca_wa)((p_csn cp_ent)(oa_ent u_isn)))	39	6
700	((p_re ca_wa)((p_csn cp_ent)(oa_ent oa_wa)))	32	6
701	((p_re ca_wa)((p_csn cp_ent)(oa_ent u_hdip)))	33	6
702	((p_re ca_wa)((p_csn cp_ent)(p_csn oa_ent)))	22	6
703	((p_re ca_wa)((p_csn oa_ent)(oa_wa u_hdip)))	37	6
704	((p_re ca_wa)((p_csn oa_ent)(u_isn oa_wa)))	31	6
705	((p_re ca_wa)((p_csn oa_ent)(u_isn u_hdip)))	39	6
706	((p_re ca_wa)((p_isn cp_ent)(oa_ent u_isn)))	27	6
707	((p_re p_re)((ca_wa p_csn)(cp_ent oa_ent)))	29	6
708	((p_re p_re)((p_isn p_csn)(p_isn oa_ent)))	27	6
709	((u_csn (cp_ent ca_ent))((ca_wa p_re) ca_wa))	43	6
710	((u_csn cp_ent)((ca_ent p_isn)(ca_wa p_csn)))	33	6
711	((u_csn cp_ent)((p_isn ca_ent)(ca_wa p_csn)))	27	6
712	((u_csn cp_ent)((p_isn ca_ent)(ca_wa p_re)))	36	6
713	((u_hdip oa_wa)((u_csn cp_ent)(ca_ent p_isn)))	32	6
714	((u_hdip oa_wa)((u_csn cp_ent)(p_csn ca_ent)))	25	6
715	((u_hdip oa_wa)((u_csn cp_ent)(ca_ent ca_wa)))	34	6
716	((u_hdip oa_wa)((u_csn cp_ent)(oa_ent u_hdip)))	50	6
717	((u_hdip oa_wa)((u_csn cp_ent)(oa_wa u_hdip)))	25	6
718	((u_hdip u_csn)((cp_ent oa_ent)(oa_wa u_hdip)))	49	6
719	((u_hdip u_csn)((cp_ent oa_ent)(u_isn u_hdip)))	43	6
720	((u_hdip u_csn)((u_isn cp_ent)(oa_ent oa_wa)))	35	6
721	((u_hdip u_csn)((u_isn cp_ent)(oa_ent u_isn)))	30	6
722	((u_hdip u_hdip)((u_csn cp_ent)(p_csn ca_ent)))	22	6
723	((u_hdip u_hdip)((oa_wa (u_csn cp_ent)) ca_ent))	31	6
724	((u_hdip u_hdip)((oa_wa u_csn)(cp_ent oa_ent)))	34	6
725	((u_hdip u_hdip)((oa_wa u_csn)(u_isn cp_ent)))	36	6
726	((u_hdip u_hdip)((u_isn u_csn)(u_isn cp_ent)))	38	6
727	((u_isn cp_ent)((p_isn ca_ent)(ca_wa p_re)))	27	6
728	((u_isn oa_wa)((u_isn u_hdip)(u_csn cp_ent)))	34	6
729	(((u_isn u_csn)(ca_ent ca_wa))(ca_wa p_csn))	25	6
730	((u_isn u_csn)((u_isn cp_ent)(ca_ent p_isn)))	28	6
731	((u_isn u_csn)((u_isn cp_ent)(ca_ent ca_wa)))	28	6
732	((u_isn u_hdip)((oa_wa u_csn)(cp_ent oa_ent)))	33	6
733	((u_isn u_hdip)((oa_wa u_csn)(oa_ent oa_wa)))	31	6
734	((u_isn u_hdip)((oa_wa u_csn)(u_isn cp_ent)))	30	6
735	((u_isn u_hdip)((u_csn cp_ent)(oa_ent oa_wa)))	36	6
736	((u_isn u_hdip)((u_isn u_csn)(u_isn cp_ent)))	36	6
737	((u_isn u_hdip)((u_isn u_hdip)(oa_wa u_csn)))	48	6
738	((ca_wa (p_csn cp_ent))(oa_ent (oa_wa u_hdip)))	29	6
739	((ca_wa (p_csn cp_ent))(oa_ent (u_isn u_hdip)))	32	6
740	((ca_wa (p_csn cp_ent))(p_csn (oa_ent u_isn)))	23	6
741	((ca_wa (p_csn cp_ent))((oa_ent u_isn) u_hdip))	33	6
742	((ca_wa (p_csn cp_ent))((p_csn oa_ent) oa_wa))	21	6
743	((oa_ent (oa_wa u_hdip))(oa_wa (u_csn cp_ent)))	52	6
744	((oa_ent (oa_wa u_hdip))((u_isn oa_wa) u_csn))	49	6
745	((oa_ent (u_isn u_hdip))((u_isn oa_wa) u_csn))	53	6
746	((oa_wa (u_csn cp_ent))(p_csn (p_isn ca_ent)))	25	6
747	((oa_wa (u_csn cp_ent))((ca_wa p_re) ca_wa))	35	6
748	((oa_wa (u_csn cp_ent))((p_isn oa_ent) oa_wa))	41	6
749	((p_csn (ca_ent ca_wa))(p_re (ca_wa p_csn)))	71	6
750	((p_csn (ca_ent ca_wa))((p_re ca_wa) p_csn))	82	6
751	((p_csn (ca_wa p_re))(ca_wa (p_csn cp_ent)))	44	6
752	((p_csn (ca_wa p_re))(p_csn (cp_ent oa_ent)))	26	6
753	((p_csn (ca_wa p_re))((p_csn cp_ent) oa_ent))	30	6
754	((p_csn (oa_ent u_hdip))((u_isn oa_wa) u_hdip))	41	6
755	((p_csn (oa_ent u_isn))(u_hdip (u_isn oa_wa)))	47	6
756	((p_re (ca_wa p_csn))((oa_ent oa_wa) u_hdip))	26	6
757	((p_re (ca_wa p_csn))((p_isn cp_ent) oa_ent))	24	6
758	((u_csn (ca_ent ca_wa))((cp_ent oa_ent) u_hdip))	37	6
759	(((u_csn cp_ent)(ca_ent p_isn))(p_re ca_wa))	43	6
760	(((u_csn cp_ent)(ca_ent p_isn))(p_re p_re))	31	6
761	(((u_csn cp_ent)(ca_wa p_re))(ca_wa p_csn))	31	6
762	((u_csn (cp_ent ca_wa))((p_re ca_wa) p_csn))	31	6
763	(((u_csn cp_ent)(p_csn ca_ent))(ca_wa ca_wa))	33	6
764	(((u_csn cp_ent)(p_csn ca_ent))(ca_wa p_re))	33	6
765	(((u_csn cp_ent)(p_csn ca_ent))(p_re ca_wa))	33	6
766	((u_csn (p_isn ca_ent))((ca_wa p_re) ca_wa))	36	6
767	((u_csn (p_isn ca_ent))((p_re ca_wa) oa_ent))	22	6
768	((u_csn (u_isn cp_ent))((ca_ent ca_wa) p_re))	29	6
769	((u_csn (u_isn cp_ent))((ca_wa p_re) ca_wa))	26	6
770	((u_hdip (oa_wa u_csn))(oa_ent (u_isn u_hdip)))	40	6
771	((u_hdip (oa_wa u_csn))(p_csn (ca_ent ca_wa)))	23	6
772	((u_hdip (oa_wa u_csn))((cp_ent ca_wa) cp_ent))	24	6
773	((u_hdip (oa_wa u_csn))((cp_ent oa_ent) u_hdip))	51	6
774	((u_hdip (oa_wa u_csn))((oa_ent u_isn) u_hdip))	43	6
775	((u_hdip (u_csn cp_ent))(ca_ent (ca_wa p_csn)))	22	6
776	((u_hdip (u_csn cp_ent))(oa_ent (oa_wa u_hdip)))	48	6
777	((u_hdip (u_csn cp_ent))(oa_ent (u_isn u_hdip)))	49	6
778	((u_hdip (u_csn cp_ent))(p_csn (ca_ent ca_wa)))	26	6
779	((u_hdip (u_csn cp_ent))(p_csn (p_isn ca_ent)))	21	6
780	((u_hdip (u_csn cp_ent))(p_csn (p_isn ca_wa)))	18	6
781	((u_hdip (u_csn cp_ent))((oa_ent u_isn) u_hdip))	58	6
782	((u_hdip (u_csn cp_ent))((p_isn oa_ent) oa_wa))	35	6
783	((u_hdip (u_isn oa_wa))(u_csn (cp_ent ca_ent)))	31	6
784	((u_hdip (u_isn oa_wa))(u_csn (ca_ent ca_wa)))	31	6
785	((u_hdip (u_isn oa_wa))(u_csn (cp_ent oa_ent)))	30	6
786	((u_hdip (u_isn oa_wa))(u_csn (oa_ent u_isn)))	30	6
787	((u_hdip (u_isn oa_wa))(u_csn (oa_ent oa_wa)))	29	6
788	((u_hdip (u_isn oa_wa))(u_csn (oa_ent u_hdip)))	30	6
789	((u_hdip (u_isn oa_wa))((u_csn cp_ent) p_csn))	38	6
790	((u_hdip (u_isn u_csn))((u_isn cp_ent) ca_ent))	27	6
791	((u_hdip (u_isn u_csn))((u_isn cp_ent) p_csn))	27	6
792	(((ca_ent ca_wa) cp_ent)(oa_ent (oa_wa u_hdip)))	44	6
793	(((ca_ent ca_wa) cp_ent)(oa_ent (u_isn u_hdip)))	46	6
794	(((ca_ent ca_wa) cp_ent)(p_csn (oa_ent oa_wa)))	29	6
795	(((ca_ent ca_wa) cp_ent)(p_csn (oa_ent u_isn)))	34	6
796	(((ca_ent ca_wa) cp_ent)((oa_ent oa_wa) u_hdip))	45	6
797	(((ca_ent ca_wa) cp_ent)((oa_ent u_isn) u_hdip))	48	6
798	(((ca_ent ca_wa) cp_ent)((p_csn oa_ent) oa_wa))	32	6
799	(((ca_ent ca_wa) cp_ent)((p_isn oa_ent) u_hdip))	33	6
800	(((ca_ent ca_wa) p_csn)(oa_ent (oa_wa u_hdip)))	33	6
801	(((ca_ent ca_wa) p_csn)(oa_ent (u_isn u_hdip)))	38	6
802	(((ca_ent ca_wa) p_csn)((oa_ent oa_wa) u_hdip))	34	6
803	(((ca_ent ca_wa) p_csn)((oa_ent u_isn) u_hdip))	37	6
804	(((ca_ent ca_wa) p_csn)((p_isn oa_ent) u_hdip))	28	6
805	(((ca_ent ca_wa) p_re)(p_csn (oa_ent u_isn)))	40	6
806	(((ca_ent ca_wa)(p_csn cp_ent))(oa_ent u_isn))	38	6
807	(((ca_ent ca_wa)(p_csn cp_ent))(p_csn oa_ent))	22	6
808	(((ca_ent p_isn)(ca_wa p_csn))(oa_ent u_isn))	34	6
809	(((ca_ent p_isn)(ca_wa p_csn))(p_isn p_csn))	55	6
810	(((ca_ent p_isn)(ca_wa p_re))(p_csn cp_ent))	43	6

404	(((ca_ent p_isn)(p_re (ca_wa cp_ent))) p_csn)	57	6
405	(((ca_ent p_isn)((p_re ca_wa) cp_ent)) ca_ent)	66	6
406	(((ca_ent p_isn)((p_re ca_wa) cp_ent)) p_csn)	65	6
407	(((ca_ent p_isn)((p_re ca_wa) p_csn)) ca_ent)	32	6
408	(((p_re (ca_wa cp_ent)) oa_ent)(u_isn u_hdip))	34	6
409	(((ca_ent p_re)((ca_wa cp_ent) p_csn)) ca_ent)	58	6
410	(((p_re (ca_wa cp_ent)) p_csn)(ca_ent p_re))	49	6
411	(((p_re (ca_wa cp_ent)) p_csn)(p_isn ca_ent))	44	6
412	(((p_isn ca_ent)((p_re ca_wa) cp_ent)) p_csn)	61	6
413	(((oa_ent (u_isn cp_ent)) ca_ent)(ca_wa ca_wa))	27	6
414	(((oa_ent (u_isn cp_ent)) ca_ent)(ca_wa p_re))	24	6
415	(((oa_ent (u_isn cp_ent)) ca_ent)(p_re ca_wa))	19	6
416	(((oa_ent (u_isn cp_ent)) p_csn)(ca_ent ca_wa))	22	6
417	(((oa_ent (u_isn cp_ent)) p_csn)(ca_ent p_isn))	28	6
418	(((oa_ent (u_isn cp_ent)) p_csn)(p_isn ca_ent))	24	6
419	(((oa_ent (u_isn u_hdip))(u_csn cp_ent)) p_csn)	27	6
420	(((oa_ent (u_isn u_hdip))(u_isn cp_ent)) p_csn)	45	6
421	(((u_csn (cp_ent ca_ent))(ca_wa cp_ent)) p_csn)	28	6
422	(((u_hdip (u_isn cp_ent)) oa_ent)(u_isn u_hdip))	50	6
423	((((ca_ent ca_wa) p_re) cp_ent)(oa_ent u_hdip))	40	6
424	((((ca_ent ca_wa) p_re) cp_ent)(oa_ent u_isn))	47	6
425	((((ca_ent ca_wa) p_re) cp_ent)(p_csn ca_ent))	45	6
426	((((ca_ent ca_wa) p_re) cp_ent)(p_csn oa_ent))	33	6
427	((((ca_ent ca_wa) p_re) cp_ent)(p_isn ca_ent))	48	6
428	((((ca_ent p_isn) p_re) cp_ent)(oa_ent u_hdip))	43	6
429	((((ca_ent p_isn) p_re) cp_ent)(oa_ent u_isn))	51	6
430	((((ca_ent p_isn) p_re) cp_ent)(p_csn ca_ent))	51	6
431	((((ca_ent p_isn) p_re) cp_ent)(p_csn oa_ent))	36	6
432	((((ca_ent p_isn) p_re) cp_ent)(p_isn ca_ent))	52	6
433	((((ca_wa cp_ent) p_csn) ca_ent)(p_re ca_wa))	39	6
434	((((p_re ca_wa) cp_ent) p_csn)(ca_ent p_re))	55	6
435	((((p_re ca_wa) cp_ent) p_csn)(p_isn ca_ent))	50	6
436	((((u_csn cp_ent) p_csn) ca_ent)(ca_wa p_re))	26	6
437	((((u_isn cp_ent) p_csn) ca_ent)(ca_wa ca_wa))	32	6
438	((((u_isn cp_ent) p_csn) ca_ent)(ca_wa p_re))	30	6
439	((((u_isn u_csn) cp_ent) ca_ent)(ca_wa ca_wa))	33	6
440	((((u_isn u_csn) cp_ent) ca_ent)(ca_wa p_re))	34	6
441	((((u_isn u_csn) cp_ent) ca_ent)(p_re ca_wa))	34	6
442	((((u_isn u_csn) cp_ent) p_csn)(ca_ent p_isn))	32	6
443	((((u_isn u_csn) cp_ent) p_csn)(ca_ent p_re))	28	6
444	(((ca_ent p_isn)(p_re (ca_wa cp_ent)))(p_isn ca_ent))	45	7
445	((u_csn cp_ent)((ca_ent p_isn)(p_re (ca_wa cp_ent))))	30	7
446	(((ca_ent p_isn)(ca_wa cp_ent))(oa_ent (u_isn u_hdip)))	39	7
447	((ca_ent ca_wa)((ca_wa cp_ent)(p_csn (p_isn ca_ent))))	37	7
448	((oa_ent (u_isn u_hdip))((u_isn u_hdip)(u_isn cp_ent)))	35	7
449	(((ca_ent p_isn)(p_re (ca_wa cp_ent)))(p_csn ca_ent))	32	7
450	(((ca_ent p_re)(ca_wa cp_ent))(oa_ent (u_isn u_hdip)))	36	7
451	(((ca_ent p_re)(ca_wa cp_ent))(p_csn (p_isn ca_ent)))	29	7
452	(((ca_ent ca_wa) p_re)((ca_wa cp_ent)(oa_ent u_isn)))	42	7
453	(((ca_ent ca_wa) p_re)((ca_wa cp_ent)(oa_ent u_hdip)))	37	7
454	(((ca_ent p_isn) p_re)((ca_wa cp_ent)(oa_ent u_isn)))	43	7
455	(((ca_ent p_isn) p_re)((ca_wa cp_ent)(oa_ent u_hdip)))	38	7
456	(((ca_ent p_isn)(ca_wa p_re))((ca_wa cp_ent) oa_ent))	31	7
457	(((cp_ent ca_ent)(ca_wa cp_ent))(oa_ent (u_isn u_hdip)))	38	7
458	(((oa_ent u_hdip)(u_csn cp_ent))((ca_ent p_isn) p_re))	29	7
459	((((oa_ent u_hdip)(u_isn u_csn)) cp_ent)(ca_ent p_isn))	26	7
460	(((p_csn ca_ent)(ca_wa p_re))((ca_wa cp_ent) oa_ent))	37	7
461	(((p_csn ca_ent)(ca_wa p_re))((ca_wa cp_ent) p_csn))	50	7
462	(((p_isn ca_ent) p_re)((ca_wa cp_ent)(oa_ent u_hdip)))	35	7
463	(((p_isn ca_ent)(ca_wa cp_ent))(oa_ent (u_isn u_hdip)))	36	7
464	(((p_isn ca_ent)(ca_wa p_re))((ca_wa cp_ent) oa_ent))	38	7
465	(((u_csn cp_ent)((ca_ent p_isn) p_re))(ca_wa cp_ent))	34	7
466	(((u_csn cp_ent)(ca_ent p_isn))((p_re ca_wa) cp_ent))	36	7
467	(((u_csn cp_ent) p_csn)((ca_ent p_isn)(ca_wa cp_ent)))	27	7
468	(((u_csn cp_ent)(ca_ent ca_wa))(p_re (ca_wa cp_ent)))	31	7
469	(((u_csn cp_ent)(ca_ent ca_wa))((p_re ca_wa) cp_ent))	32	7
470	(((u_csn cp_ent)(ca_ent ca_wa))((p_re ca_wa) p_csn))	21	7
471	(((u_csn cp_ent)(ca_ent ca_wa))((p_re ca_wa) p_re))	23	7
472	(((u_csn cp_ent)(ca_ent p_isn))((p_re ca_wa) p_re))	26	7
473	(((u_isn cp_ent) p_csn)((p_isn ca_ent)(ca_wa p_re)))	24	7
474	(((u_isn cp_ent)(ca_ent ca_wa))(p_re (ca_wa cp_ent)))	28	7
475	(((u_isn cp_ent)(ca_ent ca_wa))((p_re ca_wa) cp_ent))	28	7
476	(((u_isn cp_ent)(ca_ent p_isn))((p_re ca_wa) cp_ent))	29	7
477	(((u_isn cp_ent)(ca_ent p_re))((ca_wa cp_ent) ca_ent))	26	7
478	(((u_isn cp_ent)(p_csn ca_ent))(ca_wa (p_re ca_wa)))	27	7
479	(((u_isn cp_ent)(p_csn ca_ent))((ca_wa p_re) ca_wa))	30	7
480	(((u_isn cp_ent)(p_isn ca_ent))(ca_wa (p_re ca_wa)))	29	7
481	(((u_isn cp_ent)(p_isn ca_ent))((ca_wa p_re) ca_wa))	31	7
482	(((u_isn cp_ent)(p_isn ca_ent))((p_re ca_wa) cp_ent))	21	7
483	(((u_isn u_csn)(cp_ent ca_ent))(p_re (ca_wa cp_ent)))	31	7
484	(((u_isn u_csn)(ca_ent p_isn))((p_re ca_wa) cp_ent))	29	7
485	(((u_isn u_csn) cp_ent)((ca_ent p_isn)(ca_wa cp_ent)))	30	7
486	(((u_isn u_csn)(cp_ent ca_ent))((ca_wa p_re) ca_wa))	27	7
487	(((u_isn u_csn)(cp_ent ca_ent))((p_re ca_wa) cp_ent))	32	7
488	(ca_wa (((p_re ca_wa) cp_ent)(p_csn (ca_ent p_re))))	25	7
489	((ca_ent ca_wa)(((ca_wa cp_ent) p_csn)(p_isn ca_ent)))	47	7
490	((ca_ent ca_wa)(((p_re ca_wa) cp_ent)(oa_ent u_hdip)))	41	7
491	((ca_ent ca_wa)(((p_re ca_wa) cp_ent)(oa_ent u_isn)))	49	7
492	((ca_ent ca_wa)(((p_re ca_wa) cp_ent)(p_csn ca_ent)))	48	7
493	((ca_ent ca_wa)(((p_re ca_wa) cp_ent)(p_isn ca_ent)))	50	7
494	((ca_ent ca_wa)(((p_re ca_wa) p_csn)(oa_ent u_isn)))	26	7
495	((ca_ent p_isn)((ca_wa cp_ent)(oa_ent (u_isn u_hdip))))	36	7
496	((ca_ent p_isn)((p_re (ca_wa cp_ent))(oa_ent u_isn)))	42	7
497	((ca_ent p_isn)((p_re (ca_wa cp_ent))(oa_ent u_hdip)))	37	7
498	((ca_ent p_isn)((p_re (ca_wa cp_ent))(p_isn ca_ent)))	43	7
499	((ca_ent p_isn)(((ca_wa cp_ent) p_csn)(p_isn ca_ent)))	46	7
500	((ca_ent p_isn)(((p_re ca_wa) cp_ent)(oa_ent u_hdip)))	42	7
501	((ca_ent p_isn)(((p_re ca_wa) cp_ent)(oa_ent u_isn)))	49	7
502	((ca_ent p_isn)(((p_re ca_wa) cp_ent)(p_csn ca_ent)))	46	7
503	((ca_ent p_isn)(((p_re ca_wa) cp_ent)(p_isn ca_ent)))	49	7
504	((ca_ent p_isn)(((p_re ca_wa) p_csn)(oa_ent u_isn)))	23	7
505	((ca_ent p_re)(((ca_wa cp_ent) oa_ent)(u_isn u_hdip)))	34	7
506	(((ca_ent p_isn) p_re)(((ca_wa cp_ent) p_csn) ca_ent))	46	7
507	((ca_wa p_re)((ca_wa cp_ent)(oa_ent (u_isn u_hdip))))	34	7
508	((ca_wa p_re)((ca_wa cp_ent)(p_csn (p_isn ca_ent))))	33	7
509	((ca_wa p_re)(((ca_wa cp_ent) oa_ent)(u_isn u_hdip)))	32	7
510	((ca_wa p_re)(((ca_wa cp_ent) p_csn)(p_isn ca_ent)))	43	7
511	((cp_ent ca_ent)((ca_wa cp_ent)(oa_ent (u_isn u_hdip))))	34	7
512	((cp_ent ca_ent)((ca_wa p_re)((ca_wa cp_ent) oa_ent)))	39	7
513	((cp_ent ca_ent)((p_re (ca_wa cp_ent))(oa_ent u_isn)))	41	7
514	((cp_ent ca_ent)((p_re (ca_wa cp_ent))(oa_ent u_hdip)))	36	7
515	((cp_ent ca_ent)(((p_re ca_wa) cp_ent)(oa_ent u_hdip)))	41	7
516	((cp_ent ca_ent)(((p_re ca_wa) cp_ent)(oa_ent u_isn)))	48	7
517	((cp_ent ca_ent)(((p_re ca_wa) p_csn)(oa_ent u_isn)))	24	7
518	((oa_ent u_hdip)((u_isn u_hdip)((u_isn cp_ent) p_csn)))	28	7
519	((oa_ent u_hdip)(((u_isn u_csn) cp_ent)(p_isn ca_ent)))	27	7
520	((oa_ent u_hdip)(((u_isn u_hdip)(u_isn cp_ent)) p_csn))	30	7
521	((p_csn ca_ent)((ca_wa p_re)((ca_wa cp_ent) oa_ent)))	35	7
522	((p_csn ca_ent)(((p_re ca_wa) p_csn)(oa_ent u_isn)))	24	7
523	((p_isn ca_ent)((ca_wa p_re)((ca_wa cp_ent) oa_ent)))	36	7
524	((p_isn ca_ent)((p_re (ca_wa cp_ent))(oa_ent u_hdip)))	36	7
525	((p_isn ca_ent)(((ca_wa cp_ent) oa_ent)(u_isn u_hdip)))	34	7
526	((p_isn ca_ent)(((p_re ca_wa) cp_ent)(oa_ent u_hdip)))	42	7
527	((p_isn ca_ent)(((p_re ca_wa) p_csn)(oa_ent u_isn)))	25	7
528	((u_csn cp_ent)((ca_ent ca_wa)(p_re (ca_wa cp_ent))))	30	7
529	((u_csn cp_ent)((ca_ent ca_wa)((p_re ca_wa) cp_ent)))	27	7
530	((u_csn cp_ent)((ca_ent p_isn)((p_re ca_wa) cp_ent)))	32	7
531	((u_csn cp_ent)((p_isn ca_ent)(p_re (ca_wa cp_ent))))	33	7
532	((u_csn cp_ent)(((p_isn ca_ent) p_re)(ca_wa cp_ent)))	34	7
533	((u_isn cp_ent)(p_csn ((p_isn ca_ent)(ca_wa p_re))))	21	7
534	((u_isn cp_ent)((ca_ent p_isn)((p_re ca_wa) cp_ent)))	23	7
535	((u_isn cp_ent)((p_isn ca_ent)((p_re ca_wa) cp_ent)))	23	7
536	((u_isn cp_ent)((p_csn (p_isn ca_ent))(ca_wa p_re)))	24	7
537	((u_isn u_hdip)((u_isn u_hdip)((u_isn cp_ent) p_csn)))	25	7
538	((u_isn u_hdip)(((u_isn u_hdip)(u_isn cp_ent)) p_csn))	31	7
539	((p_csn (ca_ent ca_wa))((p_re (ca_wa cp_ent)) p_csn))	48	7
540	((p_csn (ca_ent ca_wa))(((p_re ca_wa) cp_ent) p_csn))	55	7
541	((p_csn (p_isn ca_ent))((p_re (ca_wa cp_ent)) p_csn))	47	7
542	((p_csn (p_isn ca_ent))(((p_re ca_wa) cp_ent) p_csn))	52	7
543	((p_re (ca_wa cp_ent))(p_csn ((ca_ent p_isn) p_re)))	25	7
544	((p_re (ca_wa cp_ent))(p_csn ((p_isn ca_ent) p_re)))	25	7
545	(((p_re ca_wa) cp_ent)(p_csn ((ca_ent p_isn) p_re)))	32	7
546	(((p_re ca_wa) cp_ent)(p_csn ((p_isn ca_ent) p_re)))	34	7
547	(((u_isn cp_ent) ca_ent)((p_re (ca_wa cp_ent)) ca_ent))	27	7
548	(((u_isn cp_ent) ca_ent)(((p_re ca_wa) cp_ent) ca_ent))	26	7
549	(((u_isn cp_ent) p_csn)(((ca_ent p_isn) p_re) cp_ent))	18	7
550	((p_csn ((ca_ent ca_wa) p_re))((ca_wa cp_ent) p_csn))	49	7
551	((p_csn ((ca_ent p_isn) p_re))((ca_wa cp_ent) p_csn))	49	7
552	((p_csn ((p_isn ca_ent) p_re))((ca_wa cp_ent) p_csn))	47	7
553	(((ca_ent ca_wa)(p_re (ca_wa cp_ent)))(p_csn ca_ent))	41	7
554	(((ca_ent ca_wa)(p_re (ca_wa cp_ent)))(p_isn ca_ent))	57	7
555	(((ca_ent ca_wa)((p_re ca_wa) cp_ent))(oa_ent u_hdip))	42	7
556	(((ca_ent ca_wa)((p_re ca_wa) cp_ent))(p_csn oa_ent))	37	7
557	(((ca_ent ca_wa)((p_re ca_wa) cp_ent))(p_isn ca_ent))	62	7
558	(((ca_ent ca_wa)((p_re ca_wa) p_csn))(oa_ent u_isn))	25	7
559	(((ca_ent ca_wa)((p_re ca_wa) p_csn))(p_isn ca_ent))	34	7
560	(((ca_ent p_isn)(p_re (ca_wa cp_ent)))(oa_ent u_isn))	44	7
561	(((ca_ent p_isn)((p_re ca_wa) cp_ent))(oa_ent u_hdip))	44	7
562	(((ca_ent p_isn)((p_re ca_wa) cp_ent))(oa_ent u_isn))	51	7
563	(((ca_ent p_isn)((p_re ca_wa) p_csn))(oa_ent u_isn))	25	7
564	(((ca_ent p_isn)((p_re ca_wa) p_csn))(p_isn ca_ent))	35	7
565	(((p_re (ca_wa cp_ent)) p_csn)((ca_ent p_isn) p_re))	26	7
566	(((p_re (ca_wa cp_ent)) p_csn)((p_isn ca_ent) p_re))	47	7
567	(((ca_wa cp_ent)(p_csn (p_isn ca_ent)))(p_re ca_wa))	30	7
568	(((cp_ent ca_ent)(p_re (ca_wa cp_ent)))(oa_ent u_hdip))	37	7
569	(((cp_ent ca_ent)(p_re (ca_wa cp_ent)))(oa_ent u_isn))	42	7
570	(((cp_ent ca_ent)((p_re ca_wa) cp_ent))(oa_ent u_hdip))	42	7
571	(((cp_ent ca_ent)((p_re ca_wa) cp_ent))(oa_ent u_isn))	49	7
572	(((p_csn ca_ent)((p_re ca_wa) p_csn))(oa_ent u_isn))	25	7
573	(((p_isn ca_ent)((p_re ca_wa) p_csn))(oa_ent u_hdip))	32	7
574	(((p_isn ca_ent)((p_re ca_wa) p_csn))(oa_ent u_isn))	29	7
575	(((u_csn cp_ent)((ca_ent p_isn)(ca_wa cp_ent))) p_csn)	27	7
576	(((u_isn cp_ent)(p_csn (ca_ent ca_wa)))(ca_wa cp_ent))	24	7
577	(((u_isn cp_ent)(p_csn (ca_ent ca_wa)))(p_re ca_wa))	27	7
578	(((u_isn cp_ent)(p_csn (p_isn ca_ent)))(ca_wa ca_wa))	28	7
579	(((u_isn cp_ent)(p_csn (p_isn ca_ent)))(ca_wa p_re))	25	7
580	(((u_isn cp_ent)((ca_ent ca_wa) p_re))(ca_wa cp_ent))	23	7
581	(((u_isn cp_ent)((ca_ent p_isn) p_re))(ca_wa cp_ent))	27	7
582	((((ca_ent p_isn)(ca_wa cp_ent)) oa_ent)(u_isn u_hdip))	37	7
583	(((oa_ent (u_isn cp_ent)) ca_ent)(ca_wa (p_re ca_wa)))	24	7
584	(((oa_ent (u_isn cp_ent)) ca_ent)((ca_wa p_re) ca_wa))	25	7
585	(((oa_ent (u_isn cp_ent)) p_csn)((ca_ent p_isn) cp_ent))	17	7
586	(((oa_ent (u_isn cp_ent))(ca_ent ca_wa))(p_re ca_wa))	22	7
587	(((oa_ent (u_isn cp_ent))(ca_ent p_isn))(p_re ca_wa))	20	7
588	(((oa_ent (u_isn cp_ent))(p_csn ca_ent))(ca_wa ca_wa))	24	7
589	(((oa_ent (u_isn cp_ent))(p_isn ca_ent))(ca_wa ca_wa))	24	7
590	(((oa_ent (u_isn cp_ent))(p_isn ca_ent))(ca_wa p_re))	21	7
591	(((oa_ent (u_isn u_hdip))(u_csn cp_ent))(ca_ent p_isn))	26	7
592	(((oa_ent (u_isn u_hdip))(u_isn u_csn))(cp_ent ca_ent))	24	7
593	(((u_csn (cp_ent ca_ent))(ca_wa p_re))(ca_wa cp_ent))	29	7
594	(((u_csn (cp_ent ca_ent))(p_re (ca_wa cp_ent))) p_csn)	27	7
595	(((u_csn (cp_ent ca_ent))((ca_wa p_re) ca_wa)) p_csn)	24	7
596	(((u_csn (cp_ent ca_ent))((p_re ca_wa) cp_ent)) p_csn)	27	7
597	((((ca_ent ca_wa) p_re) cp_ent)(oa_ent (u_isn u_hdip)))	37	7
598	((((ca_ent ca_wa) p_re) cp_ent)(p_csn (oa_ent u_isn)))	33	7
599	((((ca_ent p_isn) cp_ent)(oa_ent u_hdip))(u_isn u_hdip))	26	7
600	((((ca_ent p_isn) p_re) cp_ent)(oa_ent (u_isn u_hdip)))	40	7
601	((((ca_ent p_isn) p_re) cp_ent)(p_csn (oa_ent u_isn)))	36	7
602	((((ca_wa cp_ent) p_csn)(p_isn ca_ent))(p_re ca_wa))	37	7
603	((((p_isn ca_ent) p_re)(ca_wa cp_ent))(oa_ent u_hdip))	34	7
604	((((p_isn ca_ent)(ca_wa p_re))(ca_wa cp_ent)) p_csn)	53	7
605	((((p_re ca_wa) cp_ent) p_csn)((ca_ent p_isn) p_re))	34	7
606	((((p_re ca_wa) cp_ent) p_csn)((p_isn ca_ent) p_re))	38	7
607	((((p_re ca_wa) cp_ent)(oa_ent u_isn))(u_hdip u_hdip))	27	7
608	((((u_csn cp_ent) ca_ent)(ca_wa ca_wa))(p_re p_re))	21	7
609	((((u_csn cp_ent)(ca_ent p_isn))(ca_wa cp_ent)) p_csn)	29	7
610	((((u_csn cp_ent) ca_ent)(ca_wa p_re))(ca_wa cp_ent))	31	7
611	((((u_csn cp_ent) ca_ent)((ca_wa p_re) ca_wa)) p_csn)	26	7
612	((((u_csn cp_ent) p_csn)(ca_ent p_isn))(ca_wa cp_ent))	27	7
613	((((u_csn cp_ent) p_csn) ca_ent)((ca_wa p_re) ca_wa))	25	7
614	((((u_csn cp_ent) p_csn)(ca_ent p_re))(ca_wa cp_ent))	26	7
615	((((u_csn cp_ent)(ca_ent p_re))(ca_wa cp_ent)) p_csn)	29	7
616	((((u_isn cp_ent) ca_ent)(ca_wa p_re))(ca_wa cp_ent))	30	7
617	((((u_isn cp_ent) p_csn) ca_ent)(ca_wa (p_re ca_wa)))	28	7
618	((((u_isn cp_ent) p_csn) ca_ent)((ca_wa p_re) ca_wa))	31	7
619	((((u_isn cp_ent) p_csn)(ca_ent ca_wa))(ca_wa cp_ent))	27	7
620	((((u_isn cp_ent) p_csn)(ca_ent ca_wa))(p_re ca_wa))	30	7
621	((((u_isn cp_ent) p_csn)(ca_ent p_isn))(p_re ca_wa))	31	7
622	((((u_isn cp_ent) p_csn)(p_isn ca_ent))(ca_wa ca_wa))	29	7
623	((((u_isn cp_ent) p_csn)(p_isn ca_ent))(ca_wa p_re))	27	7
624	((((u_isn u_csn)(cp_ent ca_ent))(ca_wa cp_ent)) p_csn)	26	7
625	((((u_isn u_csn) ca_ent)((ca_wa p_re) ca_wa)) p_csn)	22	7
626	((((u_isn u_csn) cp_ent)(ca_ent p_isn))(ca_wa cp_ent))	34	7
627	((((u_isn u_csn) cp_ent) ca_ent)((ca_wa p_re) ca_wa))	31	7
628	((((u_isn u_csn) cp_ent) p_csn)((ca_ent p_isn) p_re))	25	7
629	((((u_isn u_csn) cp_ent)(ca_ent ca_wa))(p_re ca_wa))	31	7
630	((((u_isn u_csn) cp_ent)(ca_ent p_isn))(p_re ca_wa))	34	7
631	((((u_isn u_csn) cp_ent)(p_isn ca_ent))(ca_wa ca_wa))	31	7
632	((((u_isn u_csn) cp_ent)(p_isn ca_ent))(ca_wa cp_ent))	32	7
633	((((u_isn u_csn) cp_ent)(p_isn ca_ent))(ca_wa p_re))	33	7
634	((((u_isn u_csn) cp_ent)(p_isn ca_ent))(p_re ca_wa))	33	7
635	(ca_wa ((((p_re ca_wa) cp_ent) p_csn)(p_isn ca_ent)))	36	7
636	(((((ca_ent ca_wa) p_re) cp_ent) p_csn)(oa_ent u_isn))	33	7
637	(((((ca_ent ca_wa) p_re) cp_ent) p_csn)(p_isn ca_ent))	47	7
638	(((((ca_ent p_isn) p_re) cp_ent) p_csn)(oa_ent u_isn))	37	7
639	(((((ca_ent p_isn) p_re) cp_ent) p_csn)(p_isn ca_ent))	56	7
640	(((ca_ent p_isn)(ca_wa p_re))((ca_wa cp_ent)(oa_ent u_isn)))	31	8
641	(((ca_ent p_isn)(ca_wa p_re))((ca_wa cp_ent)(oa_ent u_hdip)))	28	8
642	(((p_csn ca_ent)(ca_wa p_re))((ca_wa cp_ent)(oa_ent u_isn)))	37	8
643	(((p_isn ca_ent)(ca_wa p_re))((ca_wa cp_ent)(oa_ent u_isn)))	38	8
644	(((p_isn ca_ent)(ca_wa p_re))((ca_wa cp_ent)(oa_ent u_hdip)))	34	8
645	(((u_csn cp_ent) p_csn)((ca_ent p_isn)(p_re (ca_wa cp_ent))))	26	8
646	(((u_isn cp_ent)(ca_ent ca_wa))((ca_wa cp_ent)(ca_ent p_isn)))	22	8
647	(((u_isn cp_ent)(ca_ent ca_wa))((ca_wa cp_ent)(p_csn ca_ent)))	20	8
648	(((u_isn cp_ent)(ca_ent p_re))((ca_wa cp_ent)(p_isn ca_ent)))	26	8
649	(((u_isn u_csn) cp_ent)((ca_ent p_isn)(p_re (ca_wa cp_ent))))	29	8
650	((ca_ent ca_wa)((p_re p_re)((ca_wa cp_ent)(oa_ent u_hdip))))	23	8
651	((ca_ent ca_wa)((p_re (ca_wa cp_ent))(oa_ent (u_isn u_hdip))))	35	8
652	((ca_ent ca_wa)(((p_re ca_wa) cp_ent)(oa_ent (u_isn u_hdip))))	38	8
653	((ca_ent ca_wa)(((p_re ca_wa) cp_ent)(p_csn (p_isn ca_ent))))	41	8
654	((ca_ent p_isn)((p_re (ca_wa cp_ent))(oa_ent (u_isn u_hdip))))	35	8
655	((ca_ent p_isn)(((p_re ca_wa) cp_ent)(oa_ent (u_isn u_hdip))))	39	8
656	((ca_ent p_isn)(((p_re ca_wa) cp_ent)(p_csn (p_isn ca_ent))))	39	8
657	((ca_ent p_isn)((p_re (ca_wa cp_ent))(p_csn (p_isn ca_ent))))	34	8
658	((ca_ent ca_wa)(((p_re (ca_wa cp_ent)) p_csn)(p_isn ca_ent)))	44	8
659	((cp_ent ca_ent)((ca_wa p_re)((ca_wa cp_ent)(oa_ent u_isn))))	39	8
660	((cp_ent ca_ent)((ca_wa p_re)((ca_wa cp_ent)(oa_ent u_hdip))))	34	8
661	((cp_ent ca_ent)((p_re (ca_wa cp_ent))(oa_ent (u_isn u_hdip))))	33	8
662	((cp_ent ca_ent)(((p_re ca_wa) cp_ent)(oa_ent (u_isn u_hdip))))	38	8
663	((p_csn ca_ent)((ca_wa p_re)((ca_wa cp_ent)(oa_ent u_isn))))	35	8
664	((p_isn ca_ent)((ca_wa p_re)((ca_wa cp_ent)(oa_ent u_isn))))	36	8
665	((p_isn ca_ent)((ca_wa p_re)((ca_wa cp_ent)(oa_ent u_hdip))))	30	8
666	((p_isn ca_ent)((p_re p_re)((ca_wa cp_ent)(oa_ent u_hdip))))	22	8
667	((p_isn ca_ent)((p_re (ca_wa cp_ent))(oa_ent (u_isn u_hdip))))	34	8
668	((p_isn ca_ent)(((p_re ca_wa) cp_ent)(oa_ent (u_isn u_hdip))))	39	8
669	((u_csn cp_ent)(((p_isn ca_ent)(ca_wa p_re))(ca_wa cp_ent)))	32	8
670	((oa_ent (u_isn u_hdip))((u_isn u_hdip)((u_isn cp_ent) p_csn)))	28	8
671	((oa_ent (u_isn u_hdip))(((u_isn u_hdip)(u_isn cp_ent)) p_csn))	30	8
672	((p_csn (ca_ent ca_wa))(((p_re ca_wa) p_csn)(oa_ent u_isn)))	26	8
673	((p_csn (p_isn ca_ent))(((p_re ca_wa) p_csn)(oa_ent u_isn)))	25	8
674	(((ca_ent ca_wa) p_re)((ca_wa cp_ent)(oa_ent (u_isn u_hdip))))	33	8
675	(((ca_ent p_isn) p_re)((ca_wa cp_ent)(oa_ent (u_isn u_hdip))))	36	8
676	(((ca_ent p_isn) p_re)(((ca_wa cp_ent) oa_ent)(u_isn u_hdip)))	34	8
677	(((p_isn ca_ent) p_re)((ca_wa cp_ent)(oa_ent (u_isn u_hdip))))	33	8
678	(((p_isn ca_ent) p_re)(((ca_wa cp_ent) oa_ent)(u_isn u_hdip)))	31	8
679	(((u_csn cp_ent)(ca_ent ca_wa))(((p_re ca_wa) cp_ent) ca_ent))	24	8
680	(((u_csn cp_ent) p_csn)((ca_ent ca_wa)(p_re (ca_wa cp_ent))))	28	8
681	(((u_csn cp_ent)(ca_ent ca_wa))(((p_re ca_wa) p_csn) ca_ent))	16	8
682	(((u_isn cp_ent) ca_ent)(((p_re ca_wa) cp_ent)(p_isn ca_ent)))	26	8
683	(((u_isn cp_ent) p_csn)((ca_ent ca_wa)((p_re ca_wa) cp_ent)))	19	8
684	(((u_isn cp_ent) p_csn)((p_isn ca_ent)((p_re ca_wa) cp_ent)))	18	8
685	(((u_isn cp_ent)(ca_ent ca_wa))((p_re (ca_wa cp_ent)) ca_ent))	24	8
686	(((u_isn cp_ent)(ca_ent ca_wa))(((p_re ca_wa) cp_ent) ca_ent))	25	8
687	(((u_isn cp_ent)(ca_ent p_isn))(((p_re ca_wa) cp_ent) ca_ent))	25	8
688	(((u_isn u_csn) cp_ent)((ca_ent ca_wa)(p_re (ca_wa cp_ent))))	29	8
689	(((u_isn u_csn) cp_ent)((ca_ent ca_wa)((p_re ca_wa) cp_ent)))	27	8
690	(((u_isn u_csn) cp_ent)((ca_ent p_isn)((p_re ca_wa) cp_ent)))	31	8
691	(((u_isn u_csn) cp_ent)((p_isn ca_ent)(p_re (ca_wa cp_ent))))	32	8
692	(((u_isn u_csn) cp_ent)(((p_isn ca_ent) p_re)(ca_wa cp_ent)))	33	8
693	((p_csn ((p_isn ca_ent)(ca_wa p_re)))((ca_wa cp_ent) oa_ent))	34	8
694	((p_csn ((p_isn ca_ent)(ca_wa p_re)))((ca_wa cp_ent) p_csn))	46	8
695	(((ca_ent ca_wa)(p_re (ca_wa cp_ent)))(p_csn (p_isn ca_ent)))	36	8
696	(((ca_ent ca_wa)((p_re ca_wa) cp_ent))(oa_ent (u_isn u_hdip)))	39	8
697	(((ca_ent ca_wa)((p_re ca_wa) cp_ent))(p_csn (oa_ent u_isn)))	37	8
698	(((ca_ent ca_wa)((p_re ca_wa) cp_ent))(p_csn (p_isn ca_ent)))	44	8
699	(((ca_ent p_isn)((p_re ca_wa) cp_ent))(oa_ent (u_isn u_hdip)))	41	8
700	(((cp_ent ca_ent)(p_re (ca_wa cp_ent)))(oa_ent (u_isn u_hdip)))	35	8
701	(((cp_ent ca_ent)((p_re ca_wa) cp_ent))(oa_ent (u_isn u_hdip)))	39	8
702	(((u_isn cp_ent)(p_csn (ca_ent ca_wa)))((p_re ca_wa) cp_ent))	23	8
703	(((u_isn cp_ent)(p_csn (ca_ent ca_wa)))((p_re ca_wa) p_csn))	18	8
704	(((u_isn cp_ent)(p_csn (p_isn ca_ent)))(ca_wa (p_re ca_wa)))	23	8
705	(((u_isn cp_ent)(p_csn (p_isn ca_ent)))((ca_wa p_re) ca_wa))	26	8
706	(((u_isn cp_ent)((ca_ent ca_wa) p_re))((ca_wa cp_ent) ca_ent))	20	8
707	(((u_isn cp_ent)((ca_ent p_isn) cp_ent))((p_isn ca_ent) p_re))	19	8
708	(((u_isn cp_ent)((ca_ent p_isn) p_re))((ca_wa cp_ent) ca_ent))	25	8
709	(((u_isn cp_ent)(p_isn ca_ent))((ca_wa p_re)(ca_wa cp_ent)))	23	8
710	(((oa_ent (u_isn cp_ent))(ca_ent ca_wa))((p_re ca_wa) cp_ent))	19	8
711	(((oa_ent (u_isn cp_ent))(p_isn ca_ent))(ca_wa (p_re ca_wa)))	20	8
712	(((oa_ent (u_isn cp_ent))(p_isn ca_ent))((ca_wa p_re) ca_wa))	21	8
713	(((oa_ent (u_isn cp_ent))(p_csn (p_isn ca_ent)))(ca_wa ca_wa))	19	8
714	((((oa_ent (u_isn u_hdip))(u_isn u_csn)) cp_ent)(ca_ent p_isn))	24	8
715	(((p_csn (ca_ent ca_wa))((p_re ca_wa) p_csn))(oa_ent u_isn))	34	8
716	(((p_csn (p_isn ca_ent))(ca_wa p_re))((ca_wa cp_ent) p_csn))	47	8
717	(((p_csn (p_isn ca_ent))((p_re ca_wa) p_csn))(oa_ent u_isn))	32	8
718	((((ca_ent p_isn) cp_ent)(oa_ent (u_isn u_hdip)))(u_isn u_hdip))	26	8
719	((((cp_ent ca_ent) cp_ent)(oa_ent (u_isn u_hdip)))(u_isn u_hdip))	25	8
720	((((p_isn ca_ent)(ca_wa p_re))(ca_wa cp_ent))(oa_ent u_hdip))	32	8
721	((((p_re ca_wa) cp_ent)(oa_ent u_isn))(u_hdip (u_isn u_hdip)))	27	8
722	((((u_csn cp_ent)(ca_ent p_isn))((p_re ca_wa) cp_ent)) p_csn)	30	8
723	((((u_csn cp_ent) p_csn)(ca_ent p_isn))(p_re (ca_wa cp_ent)))	27	8
724	((((u_csn cp_ent) p_csn)(ca_ent p_re))((ca_wa cp_ent) p_csn))	23	8
725	(((((u_csn cp_ent) p_csn)(ca_ent p_isn))(ca_wa cp_ent)) p_csn)	24	8
726	((((u_csn cp_ent) p_csn)((ca_ent p_isn) p_re))(ca_wa cp_ent))	25	8
727	((((u_csn cp_ent)(ca_ent ca_wa))(p_re ca_wa))(ca_ent p_isn))	23	8
728	((((u_csn cp_ent)(ca_ent ca_wa))(p_re ca_wa))(cp_ent ca_ent))	24	8
729	((((u_csn cp_ent)(ca_ent ca_wa))(p_re (ca_wa cp_ent))) p_csn)	26	8
730	((((u_csn cp_ent)(ca_ent ca_wa))((p_re ca_wa) cp_ent)) p_csn)	28	8
731	((((u_isn cp_ent) ca_ent)(ca_wa p_re))((ca_wa cp_ent) p_csn))	26	8
732	((((u_isn cp_ent) ca_ent)((ca_wa p_re) ca_wa))(p_isn ca_ent))	24	8
733	((((u_isn cp_ent) p_csn)(ca_ent ca_wa))(p_re (ca_wa cp_ent)))	25	8
734	((((u_isn cp_ent) p_csn)(ca_ent ca_wa))((p_re ca_wa) cp_ent))	26	8
735	((((u_isn cp_ent) p_csn)(ca_ent ca_wa))((p_re ca_wa) p_csn))	21	8
736	((((u_isn cp_ent) p_csn)(p_isn ca_ent))(ca_wa (p_re ca_wa)))	24	8
737	((((u_isn cp_ent) p_csn)(p_isn ca_ent))((ca_wa p_re) ca_wa))	27	8
738	((((u_isn cp_ent) p_csn)((ca_ent ca_wa) p_re))(ca_wa cp_ent))	22	8
739	((((u_isn cp_ent) p_csn)((ca_ent p_isn) p_re))(ca_wa cp_ent))	25	8
740	((((u_isn cp_ent) p_csn)((ca_ent p_re) cp_ent))(ca_ent p_isn))	19	8
741	((((u_isn cp_ent)(ca_ent ca_wa))(p_re ca_wa))(ca_ent p_isn))	23	8
742	((((u_isn cp_ent)(ca_ent ca_wa))(p_re ca_wa))(p_isn ca_ent))	23	8
743	((((u_isn cp_ent)(ca_ent ca_wa))(p_re (ca_wa cp_ent))) p_csn)	24	8
744	((((u_isn cp_ent)(p_csn ca_ent))(ca_wa p_re))(ca_wa cp_ent))	24	8
745	((((u_isn cp_ent)(p_isn ca_ent))(ca_wa p_re))(ca_wa cp_ent))	27	8
746	((((u_isn u_csn)(cp_ent ca_ent))(ca_wa p_re))(ca_wa cp_ent))	27	8
747	((((u_isn u_csn)(cp_ent ca_ent))(p_re (ca_wa cp_ent))) p_csn)	25	8
748	((((u_isn u_csn)(ca_ent p_isn))((p_re ca_wa) cp_ent)) p_csn)	23	8
749	((((u_isn u_csn) cp_ent)((ca_ent p_isn) p_re))(ca_wa cp_ent))	33	8
750	((((u_isn u_csn) cp_ent)(ca_ent p_isn))((p_re ca_wa) cp_ent))	35	8
751	((((u_isn u_csn) cp_ent) p_csn)((ca_ent p_isn)(ca_wa cp_ent)))	24	8
752	((((u_isn u_csn) cp_ent)(ca_ent ca_wa))((p_re ca_wa) cp_ent))	30	8
753	((((u_isn u_csn) cp_ent)(ca_ent ca_wa))((p_re ca_wa) p_csn))	20	8
754	((((u_isn u_csn) cp_ent)(ca_ent ca_wa))((p_re ca_wa) p_re))	22	8
755	((((u_isn u_csn) cp_ent)(p_isn ca_ent))(ca_wa (p_re ca_wa)))	31	8
756	((((u_isn u_csn) cp_ent)(p_isn ca_ent))(p_re (ca_wa cp_ent)))	32	8
757	((((u_isn u_csn) cp_ent)(p_isn ca_ent))((ca_wa p_re) ca_wa))	32	8
758	((((u_isn u_csn) cp_ent)(p_isn ca_ent))((p_re ca_wa) cp_ent))	34	8
759	((((u_isn u_csn) cp_ent)(p_isn ca_ent))((p_re ca_wa) p_re))	26	8
760	((((u_isn u_csn) cp_ent)((ca_ent p_isn)(ca_wa cp_ent))) p_csn)	25	8
761	((((u_isn u_csn)(cp_ent ca_ent))((ca_wa p_re) ca_wa)) p_csn)	23	8
762	((((u_isn u_csn)(cp_ent ca_ent))((p_re ca_wa) cp_ent)) p_csn)	26	8
763	((ca_ent ca_wa)((((p_re ca_wa) cp_ent) p_csn)(p_isn ca_ent)))	49	8
764	((ca_ent p_isn)(((p_re (ca_wa cp_ent)) p_csn)(p_isn ca_ent)))	42	8
765	((ca_ent p_isn)((((p_re ca_wa) cp_ent) p_csn)(p_isn ca_ent)))	48	8
766	((cp_ent ca_ent)(ca_wa (((p_re ca_wa) cp_ent)(oa_ent u_isn))))	35	8
767	((p_csn ca_ent)(ca_wa (((p_re ca_wa) cp_ent)(oa_ent u_isn))))	31	8
768	((p_isn ca_ent)(ca_wa (((p_re ca_wa) cp_ent)(oa_ent u_isn))))	35	8
769	((p_isn ca_ent)(((p_re (ca_wa cp_ent)) oa_ent)(u_isn u_hdip)))	32	8
770	((u_isn cp_ent)((ca_ent ca_wa)(((p_re ca_wa) cp_ent) ca_ent)))	20	8
771	((u_isn cp_ent)((ca_ent p_isn)(((p_re ca_wa) cp_ent) ca_ent)))	19	8
772	((p_csn ((ca_ent ca_wa)((p_re ca_wa) p_csn)))(oa_ent u_isn))	33	8
773	((p_csn ((ca_ent p_isn)((p_re ca_wa) p_csn)))(oa_ent u_isn))	29	8
774	((((ca_ent ca_wa)(p_re (ca_wa cp_ent))) p_csn)(p_isn ca_ent))	50	8
775	((((ca_ent p_isn)(p_re (ca_wa cp_ent))) p_csn)(p_isn ca_ent))	48	8
776	((((ca_ent p_isn)((p_re ca_wa) cp_ent)) p_csn)(p_isn ca_ent))	53	8
777	(((u_csn cp_ent)((ca_ent ca_wa)(p_re (ca_wa cp_ent)))) p_csn)	25	8
778	(((u_csn cp_ent)((ca_ent ca_wa)((p_re ca_wa) cp_ent))) p_csn)	23	8
779	(((u_csn cp_ent)((ca_ent p_isn)((p_re ca_wa) cp_ent))) p_csn)	26	8
780	(((u_csn cp_ent)(((p_isn ca_ent) p_re)(ca_wa cp_ent))) p_csn)	29	8
781	((((ca_ent p_isn)(p_re (ca_wa cp_ent))) p_csn)(p_isn ca_ent))	48	8
782	((((u_isn cp_ent)(p_csn (ca_ent ca_wa)))(ca_wa cp_ent)) p_csn)	21	8
783	((((oa_ent (u_isn cp_ent)) p_csn)(p_isn ca_ent))(ca_wa ca_wa))	20	8
784	(((((u_csn cp_ent) p_csn) ca_ent)(ca_wa p_re))(ca_wa cp_ent))	25	8
785	(((((u_isn u_csn) cp_ent) ca_ent)(ca_wa ca_wa))(p_re p_re))	21	8
786	(((((u_isn u_csn) cp_ent) ca_ent)(ca_wa p_re))(ca_wa cp_ent))	30	8
787	(((((u_isn u_csn) cp_ent) p_csn)(ca_ent p_isn))(ca_wa cp_ent))	24	8
788	(((((u_isn u_csn) cp_ent) p_csn)(ca_ent p_re))(ca_wa cp_ent))	23	8
789	(((ca_ent p_isn)(ca_wa p_re))(((ca_wa cp_ent)(oa_ent (u_isn u_hdip)))))	27	9
790	(((ca_ent p_isn)(ca_wa p_re))((((ca_wa cp_ent) oa_ent)(u_isn u_hdip))))	25	9
791	(((p_isn ca_ent)(ca_wa cp_ent))((((ca_ent p_re) cp_ent)(oa_ent u_isn))))	21	9
792	(((p_isn ca_ent)(ca_wa p_re))(((ca_wa cp_ent)(oa_ent (u_isn u_hdip)))))	32	9
793	(((p_isn ca_ent)(ca_wa p_re))((((ca_wa cp_ent) oa_ent)(u_isn u_hdip))))	30	9
794	(((u_csn cp_ent)(ca_ent p_isn))((((p_re ca_wa) cp_ent)(ca_ent p_isn))))	24	9
795	(((u_csn cp_ent)(ca_ent p_isn))((((p_re ca_wa) cp_ent)(p_isn ca_ent))))	25	9
796	(((u_csn cp_ent)(ca_ent ca_wa))((((p_re ca_wa) p_csn)(ca_ent p_isn))))	16	9
797	(((u_isn cp_ent)(ca_ent p_isn))((((p_re ca_wa) cp_ent)(ca_ent p_isn))))	23	9
798	(((u_isn cp_ent)(ca_ent ca_wa))(((ca_wa cp_ent)(p_csn (p_isn ca_ent)))))	22	9
799	(((u_isn cp_ent)(ca_ent ca_wa))(((p_re (ca_wa cp_ent))(ca_ent p_isn))))	24	9
800	(((u_isn cp_ent)(ca_ent ca_wa))(((p_re (ca_wa cp_ent))(p_isn ca_ent))))	24	9
801	(((u_isn cp_ent)(ca_ent ca_wa))((((p_re ca_wa) cp_ent)(ca_ent p_isn))))	25	9
802	(((u_isn cp_ent)(ca_ent ca_wa))((((p_re ca_wa) cp_ent)(p_isn ca_ent))))	25	9
803	(((u_isn cp_ent)(ca_ent p_isn))((((p_re ca_wa) cp_ent)(p_isn ca_ent))))	25	9
804	(((u_isn u_csn) cp_ent)(((p_isn ca_ent)(ca_wa p_re))(ca_wa cp_ent)))	31	9
805	((p_csn ((p_isn ca_ent)(ca_wa p_re)))(((ca_wa cp_ent)(oa_ent u_isn))))	34	9
806	(((u_isn cp_ent)((ca_ent ca_wa) p_re))(((ca_wa cp_ent)(p_isn ca_ent))))	20	9
807	(((u_isn cp_ent)((ca_ent p_isn) p_re))(((ca_wa cp_ent)(ca_ent p_isn))))	20	9
808	(((u_isn cp_ent)((ca_ent p_isn) p_re))(((ca_wa cp_ent)(p_isn ca_ent))))	26	9
809	(((u_isn cp_ent)((p_csn ca_ent) p_re))(((ca_wa cp_ent)(p_isn ca_ent))))	24	9
810	(((u_isn cp_ent)((p_isn ca_ent) p_re))(((ca_wa cp_ent)(p_isn ca_ent))))	26	9

811	(((ca_ent p_isn)(p_csn cp_ent))(oa_ent u_isn))	35	6
812	(((ca_ent p_isn)(p_re p_csn))(p_isn p_csn))	48	6
813	(((ca_ent p_re)p_csn)(oa_ent(oa_wa u_hdip)))	32	6
814	(((ca_ent p_re)p_csn)(oa_ent(u_isn u_hdip)))	36	6
815	(((ca_ent p_re)p_csn)((oa_ent oa_wa)u_hdip))	33	6
816	(((ca_ent p_re)p_csn)((oa_ent u_isn)u_hdip))	35	6
817	(((ca_ent p_re)(cp_ent oa_ent))(oa_wa u_hdip))	32	6
818	(((ca_ent p_re)(cp_ent oa_ent))(u_hdip u_hdip))	34	6
819	(((ca_ent p_re)(p_csn cp_ent))(oa_ent oa_wa))	33	6
820	(((ca_ent p_re)(p_csn oa_ent))(u_isn u_hdip))	24	6
821	(((ca_wa ca_wa)(cp_ent oa_ent))(oa_wa u_hdip))	28	6
822	(((ca_wa ca_wa)(cp_ent oa_ent))(u_hdip u_hdip))	37	6
823	(((ca_wa ca_wa)(cp_ent oa_ent))(u_isn u_hdip))	37	6
824	(((ca_wa ca_wa)(p_csn oa_ent))(oa_wa u_hdip))	28	6
825	(((ca_wa ca_wa)(p_csn oa_ent))(u_hdip u_hdip))	36	6
826	(((ca_wa ca_wa)(p_csn oa_ent))(u_isn u_hdip))	36	6
827	(((ca_wa ca_wa)(p_isn cp_ent))(oa_ent u_isn))	27	6
828	(((ca_wa p_csn)(cp_ent oa_ent))(u_isn u_hdip))	35	6
829	(((ca_wa p_csn)(oa_ent u_hdip))(u_isn u_hdip))	30	6
830	(((ca_wa p_csn)(oa_ent u_isn))(u_hdip u_hdip))	31	6
831	(((ca_wa p_csn)(p_isn cp_ent))(oa_ent u_isn))	24	6
832	(((ca_wa p_re)ca_wa)(p_csn(cp_ent oa_ent)))	35	6
833	(((ca_wa p_re)ca_wa)(p_csn(oa_ent oa_wa)))	36	6
834	(((ca_wa p_re)ca_wa)(p_csn(oa_ent u_hdip)))	38	6
835	(((ca_wa p_re)ca_wa)(p_csn(oa_ent u_isn)))	48	6
836	(((ca_wa p_re)ca_wa)(p_csn(p_isn oa_ent)))	32	6
837	(((ca_wa p_re)ca_wa)((cp_ent oa_ent)oa_wa))	44	6
838	(((ca_wa p_re)ca_wa)((cp_ent oa_ent)u_hdip))	46	6
839	(((ca_wa p_re)ca_wa)((p_csn cp_ent)oa_ent))	39	6
840	(((ca_wa p_re)ca_wa)((p_csn cp_ent)p_csn))	39	6
841	(((ca_wa p_re)ca_wa)((p_csn oa_ent)oa_wa))	39	6
842	(((ca_wa p_re)ca_wa)((p_csn oa_ent)u_hdip))	40	6
843	(((ca_wa p_re)ca_wa)((p_isn cp_ent)oa_ent))	27	6
844	(((ca_wa p_re)cp_ent)(oa_ent(oa_wa u_hdip)))	41	6
845	(((ca_wa p_re)cp_ent)(oa_ent(u_isn u_hdip)))	42	6
846	(((ca_wa p_re)cp_ent)((oa_ent oa_wa)u_hdip))	42	6
847	(((ca_wa p_re)cp_ent)((oa_ent u_isn)u_hdip))	44	6
848	(((ca_wa p_re)cp_ent)((p_csn oa_ent)oa_wa))	27	6
849	(((ca_wa p_re)cp_ent)((p_isn oa_ent)u_hdip))	27	6
850	(((ca_wa p_re)(p_csn cp_ent))(oa_ent u_isn))	39	6
851	(((ca_wa p_re)(p_csn cp_ent))(p_csn oa_ent))	22	6
852	(((ca_wa p_re)(p_csn oa_ent))(oa_wa oa_wa))	28	6
853	(((ca_wa p_re)(p_csn oa_ent))(oa_wa u_hdip))	31	6
854	(((ca_wa p_re)(p_csn oa_ent))(u_hdip u_hdip))	34	6
855	(((ca_wa p_re)(p_csn oa_ent))(u_isn u_hdip))	37	6
856	(((ca_wa p_re)(p_isn cp_ent))(oa_ent u_isn))	29	6
857	(((p_re ca_wa)(p_isn cp_ent))(oa_ent u_isn))	29	6
858	(((cp_ent ca_ent)cp_ent)(oa_ent(oa_wa u_hdip)))	42	6
859	(((cp_ent ca_ent)cp_ent)(oa_ent(u_isn u_hdip)))	44	6
860	(((cp_ent ca_ent)cp_ent)((oa_ent oa_wa)u_hdip))	43	6
861	(((cp_ent ca_ent)cp_ent)((oa_ent u_isn)u_hdip))	46	6
862	(((cp_ent ca_ent)cp_ent)((p_isn oa_ent)u_hdip))	33	6
863	(((cp_ent ca_ent)(ca_wa p_re))(cp_ent oa_ent))	44	6
864	(((cp_ent ca_ent)(ca_wa p_re))(p_csn oa_ent))	39	6
865	(((cp_ent ca_ent)(ca_wa p_re))(p_isn cp_ent))	57	6
866	(((cp_ent ca_wa)cp_ent)(oa_ent(oa_wa u_hdip)))	41	6
867	(((cp_ent ca_wa)cp_ent)(oa_ent(u_isn u_hdip)))	43	6
868	(((cp_ent ca_wa)cp_ent)((oa_ent oa_wa)u_hdip))	42	6
869	(((cp_ent ca_wa)cp_ent)((oa_ent u_isn)u_hdip))	45	6
870	(((cp_ent ca_wa)cp_ent)((p_isn oa_ent)u_hdip))	31	6
871	(((cp_ent ca_wa)(p_csn oa_ent))(u_isn u_hdip))	27	6
872	(((cp_ent oa_ent)oa_wa)(u_hdip(oa_wa u_csn)))	41	6
873	(((cp_ent oa_ent)oa_wa)(u_hdip(u_csn cp_ent)))	53	6
874	(((cp_ent oa_ent)oa_wa)((u_hdip oa_wa)u_csn))	46	6
875	(((cp_ent oa_ent)oa_wa)((u_isn oa_wa)u_csn))	47	6
876	(((cp_ent oa_ent)u_hdip)((u_isn oa_wa)u_csn))	54	6
877	(((cp_ent oa_ent)(oa_wa u_hdip))(oa_wa cp_ent))	43	6
878	(((cp_ent oa_ent)(oa_wa u_hdip))(oa_wa u_csn))	56	6
879	(((cp_ent oa_ent)(oa_wa u_hdip))(u_csn cp_ent))	59	6
880	(((cp_ent oa_ent)(oa_wa u_hdip))(u_isn u_csn))	39	6
881	(((cp_ent oa_ent)(u_isn u_hdip))(oa_wa u_csn))	57	6
882	(((oa_ent oa_wa)u_hdip)(oa_wa(u_csn cp_ent)))	51	6
883	(((oa_ent oa_wa)u_hdip)((u_isn oa_wa)u_csn))	48	6
884	(((oa_ent oa_wa)(u_hdip u_csn))(cp_ent oa_ent))	40	6
885	(((oa_ent u_hdip)(oa_wa u_csn))(cp_ent oa_ent))	27	6
886	(((oa_ent u_hdip)(oa_wa u_csn))(u_isn cp_ent))	43	6
887	(((oa_ent u_isn)u_hdip)(oa_wa(u_csn cp_ent)))	56	6
888	(((oa_ent u_isn)u_hdip)((u_isn oa_wa)u_csn))	55	6
889	(((oa_wa oa_wa)(cp_ent ca_wa))(p_isn p_re))	25	6
890	(((oa_wa oa_wa)(cp_ent ca_wa))(p_re ca_wa))	25	6
891	(((oa_wa oa_wa)(cp_ent ca_wa))(p_re p_re))	19	6
892	(((oa_wa u_csn)(cp_ent ca_wa))(ca_wa p_csn))	25	6
893	(((oa_wa u_csn)(cp_ent ca_wa))(p_isn p_re))	32	6
894	(((oa_wa u_csn)(cp_ent ca_wa))(p_re ca_wa))	33	6
895	(((oa_wa u_csn)(cp_ent ca_wa))(p_re p_csn))	26	6
896	(((oa_wa u_csn)(p_csn ca_wa))(p_re ca_wa))	22	6
897	(((oa_wa u_csn)(p_isn ca_ent))(ca_wa ca_wa))	30	6
898	(((oa_wa u_csn)(p_isn ca_ent))(ca_wa p_re))	29	6
899	(((oa_wa u_csn)(p_isn ca_ent))(p_isn ca_wa))	22	6
900	(((oa_wa u_csn)(p_isn ca_ent))(p_isn p_re))	29	6
901	(((oa_wa u_csn)(p_isn ca_ent))(p_re ca_wa))	29	6
902	(((oa_wa u_csn)(u_isn cp_ent))(p_isn p_re))	24	6
903	(((oa_wa u_hdip)oa_wa)(u_csn(cp_ent ca_ent)))	30	6
904	(((oa_wa u_hdip)oa_wa)(u_csn(ca_ent ca_wa)))	30	6
905	(((oa_wa u_hdip)oa_wa)(u_csn(cp_ent ca_wa)))	27	6
906	(((oa_wa u_hdip)oa_wa)((u_csn cp_ent)p_csn))	34	6
907	(((oa_wa u_hdip)(oa_wa u_csn))(cp_ent ca_ent))	26	6
908	(((oa_wa u_hdip)(oa_wa u_csn))(cp_ent oa_ent))	44	6
909	(((p_csn ca_ent)(ca_wa p_re))(ca_wa p_csn))	73	6
910	(((p_csn ca_ent)(p_isn p_re))(ca_wa p_csn))	72	6
911	(((p_csn ca_wa)(p_isn p_re))(ca_wa p_csn))	50	6
912	(((p_csn ca_wa)(p_isn p_re))(p_isn p_csn))	44	6
913	(((p_csn cp_ent)(oa_ent oa_wa))(u_isn u_hdip))	29	6
914	(((p_csn cp_ent)(oa_ent u_isn))(oa_wa u_hdip))	30	6
915	(((p_csn oa_ent)u_hdip)((u_isn oa_wa)u_hdip))	44	6
916	(((p_csn oa_ent)(oa_wa u_hdip))(oa_wa u_csn))	38	6
917	(((p_csn oa_ent)(u_hdip oa_wa))(u_csn cp_ent))	31	6
918	(((p_csn oa_ent)(u_hdip oa_wa))(u_isn u_hdip))	34	6
919	(((p_csn oa_ent)(u_isn u_hdip))(oa_wa oa_wa))	27	6
920	(((p_csn oa_ent)(u_isn u_hdip))(oa_wa u_hdip))	45	6
921	(((p_csn oa_ent)(u_isn u_hdip))(u_isn oa_wa))	53	6
922	(((p_csn oa_ent)(u_isn u_hdip))(u_isn u_hdip))	57	6
923	(((p_isn ca_ent)(ca_wa p_re))(cp_ent oa_ent))	41	6
924	(((p_isn ca_ent)(ca_wa p_re))(p_csn oa_ent))	36	6
925	(((p_isn ca_ent)(ca_wa p_re))(p_isn p_csn))	74	6
926	(((p_isn ca_wa)p_csn)(oa_ent(oa_wa u_hdip)))	25	6
927	(((p_isn ca_wa)p_csn)(oa_ent(u_isn u_hdip)))	31	6
928	(((p_isn ca_wa)p_csn)((p_isn oa_ent)u_hdip))	23	6
929	(((p_isn ca_wa)(p_isn cp_ent))(oa_ent u_isn))	25	6
930	(((p_isn oa_ent)oa_wa)(u_hdip(oa_wa u_csn)))	29	6
931	(((p_isn oa_ent)oa_wa)((u_csn cp_ent)p_csn))	32	6
932	(((p_isn oa_ent)oa_wa)((u_hdip oa_wa)u_csn))	31	6
933	(((p_isn oa_ent)(oa_wa u_hdip))(oa_wa u_csn))	42	6
934	(((p_isn oa_ent)(oa_wa u_hdip))(u_csn cp_ent))	44	6
935	(((p_isn oa_ent)(u_isn u_hdip))(oa_wa u_csn))	47	6
936	(((p_isn p_csn)(ca_ent ca_wa))(p_isn p_re))	50	6
937	(((p_isn p_csn)(ca_ent ca_wa))(p_re ca_wa))	52	6
938	(((p_isn p_csn)(p_isn ca_wa))(p_isn p_re))	42	6
939	(((p_isn p_re)(p_csn cp_ent))(oa_ent oa_wa))	31	6
940	(((p_isn p_re)(p_csn cp_ent))(oa_ent u_hdip))	32	6
941	(((p_isn p_re)(p_csn cp_ent))(oa_ent u_isn))	38	6
942	(((p_isn p_re)(p_csn cp_ent))(p_csn oa_ent))	22	6
943	(((p_isn p_re)(p_isn cp_ent))(oa_ent u_isn))	28	6
944	(((p_re ca_wa)p_csn)(oa_ent(oa_wa u_hdip)))	32	6
945	(((p_re ca_wa)p_csn)(oa_ent(u_isn u_hdip)))	39	6
946	(((p_re ca_wa)p_csn)((oa_ent oa_wa)u_hdip))	35	6
947	(((p_re ca_wa)p_csn)((p_isn oa_ent)u_hdip))	28	6
948	(((p_re ca_wa)(cp_ent oa_ent))(oa_wa u_hdip))	39	6
949	(((p_re ca_wa)(cp_ent oa_ent))(u_hdip u_hdip))	43	6
950	(((p_re ca_wa)(cp_ent oa_ent))(u_isn u_hdip))	43	6
951	(((p_re ca_wa)(p_csn cp_ent))(oa_ent oa_wa))	33	6
952	(((p_re ca_wa)(p_csn cp_ent))(oa_ent u_hdip))	34	6
953	(((p_re ca_wa)(p_csn oa_ent))(oa_wa u_hdip))	36	6
954	(((p_re ca_wa)(p_csn oa_ent))(u_hdip u_hdip))	39	6
955	(((p_re ca_wa)(p_csn oa_ent))(u_isn u_hdip))	41	6
956	(((u_csn cp_ent)(ca_ent ca_wa))(ca_wa p_csn))	34	6
957	(((u_csn cp_ent)(ca_ent ca_wa))(p_isn ca_wa))	39	6
958	(((u_csn cp_ent)(ca_ent ca_wa))(p_isn p_re))	43	6
959	(((u_csn cp_ent)(ca_ent ca_wa))(p_re ca_wa))	44	6
960	(((u_csn cp_ent)(ca_ent ca_wa))(p_re p_csn))	35	6
961	(((u_csn cp_ent)(ca_ent ca_wa))(p_re p_re))	34	6
962	(((u_csn cp_ent)(ca_wa p_re))(p_isn ca_wa))	28	6
963	(((u_csn cp_ent)(oa_wa u_hdip))(u_isn u_hdip))	28	6
964	(((u_csn cp_ent)(p_csn ca_wa))(ca_wa p_csn))	20	6
965	(((u_csn cp_ent)(p_csn ca_wa))(p_re ca_wa))	25	6
966	(((u_csn cp_ent)(p_isn ca_ent))(ca_wa ca_wa))	37	6
967	(((u_csn cp_ent)(p_isn ca_ent))(ca_wa p_csn))	28	6
968	(((u_csn cp_ent)(p_isn ca_ent))(ca_wa p_re))	37	6
969	(((u_csn cp_ent)(p_isn ca_ent))(p_isn ca_wa))	29	6
970	(((u_csn cp_ent)(p_isn ca_ent))(p_isn p_re))	36	6
971	(((u_csn cp_ent)(p_isn ca_ent))(p_re ca_wa))	37	6
972	(((u_csn cp_ent)(p_isn ca_ent))(p_re p_csn))	28	6
973	(((u_csn cp_ent)(p_isn ca_ent))(p_re p_re))	29	6
974	(((u_csn cp_ent)(p_isn ca_wa))(p_isn ca_wa))	28	6
975	(((u_csn cp_ent)(p_isn ca_wa))(p_isn p_re))	33	6
976	(((u_csn cp_ent)(p_isn ca_wa))(p_re ca_wa))	34	6
977	(((u_csn cp_ent)(p_isn ca_wa))(p_re p_csn))	26	6
978	(((u_csn cp_ent)(p_isn p_re))(ca_wa p_csn))	28	6
979	(((u_hdip oa_wa)u_csn)(oa_ent(u_isn u_hdip)))	28	6
980	(((u_hdip oa_wa)u_csn)((cp_ent ca_wa)cp_ent))	27	6
981	(((u_hdip oa_wa)u_csn)((oa_ent u_isn)u_hdip))	49	6
982	(((u_hdip oa_wa)u_hdip)(oa_wa(u_csn cp_ent)))	22	6
983	(((u_hdip u_csn)(cp_ent oa_ent))(oa_wa u_hdip))	32	6
984	(((u_hdip u_csn)(cp_ent oa_ent))(u_hdip u_hdip))	28	6
985	(((u_hdip u_csn)(ca_ent ca_wa))(ca_wa p_csn))	26	6
986	(((u_hdip u_csn)(ca_ent ca_wa))(p_isn ca_wa))	25	6
987	(((u_hdip u_csn)(cp_ent ca_ent))(ca_wa ca_wa))	34	6
988	(((u_hdip u_csn)(cp_ent ca_ent))(ca_wa p_re))	34	6
989	(((u_hdip u_csn)(cp_ent ca_ent))(p_isn ca_wa))	27	6
990	(((u_hdip u_csn)(cp_ent ca_ent))(p_re ca_wa))	34	6
991	(((u_hdip u_csn)(cp_ent ca_wa))(ca_wa p_csn))	22	6
992	(((u_hdip u_csn)(cp_ent ca_wa))(p_re ca_wa))	30	6
993	(((u_hdip u_csn)(oa_ent u_hdip))(u_isn u_hdip))	27	6
994	(((u_hdip u_csn)(u_isn cp_ent))(oa_ent u_hdip))	36	6
995	(((u_isn cp_ent)(ca_ent ca_wa))(ca_wa p_csn))	26	6
996	(((u_isn cp_ent)(ca_ent p_isn))(p_re ca_wa))	30	6
997	(((u_isn cp_ent)(ca_ent ca_wa))(p_re p_csn))	26	6
998	(((u_isn cp_ent)ca_ent)((ca_wa p_re)ca_wa))	30	6
999	(((u_isn cp_ent)ca_ent)((ca_wa p_re)cp_ent))	28	6
1000	(((u_isn cp_ent)(ca_ent ca_wa))(p_isn p_re))	30	6
1001	(((u_isn cp_ent)(ca_ent ca_wa))(p_re ca_wa))	31	6
1002	(((u_isn cp_ent)(ca_wa p_re))(ca_wa p_csn))	23	6
1003	(((u_isn cp_ent)(oa_ent oa_wa))(u_hdip u_hdip))	34	6
1004	(((u_isn cp_ent)(oa_ent oa_wa))(u_isn u_hdip))	38	6
1005	(((u_isn cp_ent)(oa_ent u_isn))(oa_wa u_csn))	27	6
1006	(((u_isn cp_ent)(p_isn ca_ent))(ca_wa ca_wa))	28	6
1007	(((u_isn cp_ent)(p_isn ca_ent))(ca_wa p_csn))	23	6
1008	(((u_isn cp_ent)(p_isn ca_ent))(p_isn p_re))	27	6
1009	(((u_isn cp_ent)(p_isn ca_ent))(p_re ca_wa))	27	6
1010	(((u_isn cp_ent)(p_isn ca_wa))(p_isn p_re))	21	6
1011	(((u_isn cp_ent)(p_isn ca_wa))(p_re ca_wa))	21	6
1012	(((u_isn oa_wa)u_csn)(p_csn(ca_ent ca_wa)))	26	6
1013	(((u_isn oa_wa)u_csn)((cp_ent ca_wa)cp_ent))	26	6
1014	(((u_isn oa_wa)u_hdip)(u_csn(cp_ent ca_ent)))	26	6
1015	(((u_isn oa_wa)u_hdip)(u_csn(cp_ent oa_ent)))	20	6
1016	(((u_isn oa_wa)u_hdip)(u_csn(oa_ent u_isn)))	20	6
1017	(((u_isn oa_wa)u_hdip)(u_csn(oa_ent oa_wa)))	20	6
1018	(((u_isn u_csn)(cp_ent ca_ent))(ca_wa ca_wa))	32	6
1019	(((u_isn u_csn)(cp_ent ca_ent))(p_isn ca_wa))	23	6
1020	(((u_isn u_csn)(ca_ent p_isn))(p_re ca_wa))	32	6
1021	(((u_isn u_csn)(ca_ent ca_wa))(p_isn p_csn))	26	6
1022	(((u_isn u_csn)(cp_ent ca_wa))(ca_wa p_csn))	21	6
1023	(((u_isn u_csn)(cp_ent ca_wa))(p_isn ca_wa))	21	6
1024	(((u_isn u_csn)(u_isn cp_ent))(ca_ent p_isn))	30	6
1025	(((u_isn u_csn)(u_isn cp_ent))(ca_ent ca_wa))	31	6
1026	(((u_isn u_hdip)(oa_wa(u_csn cp_ent)))ca_ent)	27	6
1027	(((u_isn u_hdip)(oa_wa u_csn))(cp_ent ca_wa))	23	6
1028	(((u_isn u_hdip)(oa_wa u_csn))(cp_ent oa_ent))	34	6
1029	(((u_isn u_hdip)(oa_wa u_csn))(oa_ent u_isn))	26	6
1030	(((u_isn u_hdip)(oa_wa u_csn))(u_isn cp_ent))	37	6
1031	(((u_isn u_hdip)(u_csn cp_ent))(oa_ent oa_wa))	34	6
1032	(((u_isn u_hdip)(u_csn cp_ent))(oa_ent u_hdip))	32	6
1033	(p_csn((ca_ent ca_wa)((p_re ca_wa)oa_ent)))	29	6
1034	(p_csn((ca_ent p_isn)((p_re ca_wa)oa_ent)))	28	6
1035	(p_csn((ca_wa p_re)(ca_wa(p_csn cp_ent))))	37	6
1036	(p_csn((p_isn ca_ent)((p_re ca_wa)oa_ent)))	25	6
1037	(p_csn(((ca_ent ca_wa)cp_ent)(oa_ent u_isn)))	36	6
1038	(p_csn(((ca_ent ca_wa)cp_ent)(oa_ent u_hdip)))	31	6
1039	(p_csn(((ca_ent ca_wa)cp_ent)(p_isn oa_ent)))	25	6
1040	(p_csn(((ca_ent ca_wa)p_re)(cp_ent oa_ent)))	33	6
1041	(p_csn(((p_isn oa_ent)oa_wa)(u_csn cp_ent)))	26	6
1042	(p_csn(((p_isn oa_ent)(oa_wa u_hdip))u_csn))	27	6
1043	((ca_ent ca_wa)((p_re(ca_wa p_csn))oa_ent))	38	6
1044	((ca_ent ca_wa)(((p_re ca_wa)p_csn)oa_ent))	48	6
1045	((ca_ent p_isn)((ca_wa(p_csn cp_ent))oa_ent))	36	6
1046	((ca_ent p_isn)((ca_wa(p_csn cp_ent))p_csn))	37	6
1047	((ca_ent p_isn)((p_re(ca_wa p_csn))oa_ent))	37	6
1048	((ca_ent p_isn)(((p_re ca_wa)p_csn)oa_ent))	48	6
1049	((ca_ent p_re)((ca_wa(p_csn cp_ent))oa_ent))	36	6
1050	((ca_wa p_re)(p_csn(oa_ent(u_isn u_hdip))))	28	6
1051	((ca_wa p_re)((p_csn(oa_ent u_isn))u_hdip))	31	6
1052	((ca_wa p_re)((p_csn(p_isn oa_ent))u_hdip))	26	6
1053	((cp_ent ca_ent)((p_re(ca_wa p_csn))oa_ent))	36	6
1054	((cp_ent ca_ent)(((ca_wa p_re)ca_wa)oa_ent))	42	6
1055	((cp_ent ca_ent)(((ca_wa p_re)cp_ent)oa_ent))	47	6
1056	((cp_ent ca_ent)(((p_isn ca_wa)p_csn)oa_ent))	29	6
1057	((cp_ent ca_ent)(((p_re ca_wa)p_csn)oa_ent))	45	6
1058	((cp_ent ca_wa)((p_re(ca_wa p_csn))oa_ent))	34	6
1059	((cp_ent ca_wa)(((p_re ca_wa)p_csn)oa_ent))	43	6
1060	((oa_ent oa_wa)((u_hdip(u_isn oa_wa))u_csn))	39	6
1061	((oa_wa oa_wa)(((u_csn cp_ent)p_csn)ca_ent))	23	6
1062	((oa_wa u_hdip)(((u_csn cp_ent)p_csn)ca_ent))	27	6
1063	((p_csn ca_ent)(((ca_wa p_re)ca_wa)oa_ent))	32	6
1064	((p_csn ca_ent)(((ca_wa p_re)cp_ent)oa_ent))	37	6
1065	((p_isn ca_ent)((p_re(ca_wa p_csn))oa_ent))	29	6
1066	((p_isn ca_ent)(((ca_wa p_re)ca_wa)oa_ent))	37	6
1067	((p_isn ca_ent)(((ca_wa p_re)cp_ent)oa_ent))	43	6
1068	((p_isn ca_ent)(((p_isn ca_wa)p_csn)oa_ent))	25	6
1069	((p_isn ca_ent)(((p_re ca_wa)p_csn)oa_ent))	38	6
1070	((p_isn ca_wa)((p_re(ca_wa p_csn))oa_ent))	36	6
1071	((p_isn ca_wa)(((p_re ca_wa)p_csn)oa_ent))	44	6
1072	((p_isn p_re)((ca_wa(p_csn cp_ent))oa_ent))	38	6
1073	((p_isn p_re)((ca_wa(p_csn cp_ent))p_csn))	39	6
1074	((p_re ca_wa)(p_csn(oa_ent(oa_wa u_hdip))))	31	6
1075	((p_re ca_wa)(p_csn(oa_ent(u_isn u_hdip))))	36	6
1076	((p_re ca_wa)(p_csn((oa_ent oa_wa)u_hdip)))	34	6
1077	((p_re ca_wa)(p_csn((p_isn oa_ent)u_hdip)))	27	6
1078	((p_re p_re)((ca_wa(p_csn cp_ent))oa_ent))	30	6
1079	((u_hdip oa_wa)(((u_csn cp_ent)p_csn)ca_ent))	28	6
1080	((u_hdip u_hdip)((oa_wa(u_csn cp_ent))oa_ent))	45	6
1081	((u_hdip u_hdip)(((oa_wa u_csn)oa_ent)oa_wa))	26	6
1082	((u_isn u_hdip)((oa_wa(u_csn cp_ent))oa_ent))	29	6
1083	((u_isn u_hdip)(((oa_wa u_csn)oa_ent)oa_wa))	22	6
1084	((oa_ent((oa_wa u_hdip)(oa_wa u_csn)))oa_ent)	23	6
1085	((p_csn(oa_ent(oa_wa u_hdip)))(oa_wa u_csn))	35	6
1086	((p_csn(oa_ent(u_isn u_hdip)))(oa_wa u_hdip))	41	6
1087	((p_csn(oa_ent(u_isn u_hdip)))(u_isn oa_wa))	50	6
1088	((p_csn(oa_ent(u_isn u_hdip)))(u_isn u_hdip))	54	6
1089	((p_csn((oa_ent oa_wa)u_hdip))(oa_wa u_csn))	32	6
1090	((p_csn((oa_ent u_isn)u_hdip))(oa_wa u_csn))	40	6
1091	((p_csn((oa_ent u_isn)u_hdip))(oa_wa u_hdip))	37	6
1092	((p_csn((oa_ent u_isn)u_hdip))(u_isn oa_wa))	52	6
1093	((p_csn((p_isn oa_ent)u_hdip))(u_isn oa_wa))	35	6
1094	(((ca_ent ca_wa)((p_isn ca_wa)p_csn))oa_ent)	32	6
1095	(((ca_ent ca_wa)((p_re ca_wa)p_csn))oa_ent)	47	6
1096	(((ca_ent p_isn)(p_re(ca_wa p_csn)))oa_ent)	37	6
1097	(((ca_ent p_isn)((p_re ca_wa)p_csn))oa_ent)	46	6
1098	(((ca_wa(p_csn cp_ent))oa_ent)(u_hdip u_hdip))	33	6
1099	(((ca_wa(p_csn cp_ent))oa_ent)(u_isn u_hdip))	33	6
1100	(((p_re(ca_wa p_csn))(p_isn cp_ent))oa_ent)	23	6
1101	(((cp_ent ca_ent)((ca_wa p_re)cp_ent))oa_ent)	48	6
1102	(((oa_ent oa_wa)(u_hdip(u_csn cp_ent)))oa_ent)	44	6
1103	(((oa_wa u_csn)((cp_ent ca_wa)cp_ent))oa_ent)	26	6
1104	(((p_csn ca_wa)((p_re ca_wa)p_csn))oa_ent)	31	6
1105	(((p_csn oa_ent)((u_isn oa_wa)u_hdip))u_csn)	35	6
1106	(((p_isn ca_ent)((p_re ca_wa)p_csn))oa_ent)	37	6
1107	(((u_hdip u_hdip)(oa_wa(u_csn cp_ent)))ca_ent)	30	6
1108	(((u_isn oa_wa)((u_csn cp_ent)p_csn))ca_ent)	29	6
1109	(((u_isn u_hdip)(oa_wa(u_csn cp_ent)))oa_ent)	36	6
1110	(((oa_ent(oa_wa u_hdip))u_csn)(cp_ent oa_ent))	48	6
1111	(((oa_ent(oa_wa u_hdip))u_csn)(u_isn cp_ent))	40	6
1112	(((oa_ent(oa_wa u_hdip))(u_csn cp_ent))oa_ent)	49	6
1113	(((oa_ent(u_isn u_hdip))(oa_wa u_csn))oa_ent)	25	6
1114	(((oa_wa(u_csn cp_ent))p_csn)(ca_ent p_isn))	35	6
1115	(((oa_wa(u_csn cp_ent))p_csn)(ca_ent ca_wa))	35	6
1116	(((oa_wa(u_csn cp_ent))p_csn)(ca_wa ca_wa))	30	6
1117	(((oa_wa(u_csn cp_ent))p_csn)(p_isn ca_ent))	29	6
1118	(((p_csn(ca_wa p_re))p_csn)(cp_ent oa_ent))	26	6
1119	(((p_csn(ca_wa p_re))p_csn)(oa_ent u_isn))	31	6
1120	(((p_csn(ca_wa p_re))(ca_wa p_csn))oa_ent)	26	6
1121	(((p_csn(oa_ent u_hdip))(u_isn oa_wa))u_csn)	39	6
1122	(((p_csn(oa_ent u_isn))u_hdip)(oa_wa oa_wa))	30	6
1123	(((p_csn(oa_ent u_isn))u_hdip)(oa_wa u_csn))	41	6
1124	(((p_csn(oa_ent u_isn))u_hdip)(oa_wa u_hdip))	44	6
1125	(((p_csn(oa_ent u_isn))u_hdip)(u_isn oa_wa))	55	6
1126	(((p_csn(p_isn oa_ent))u_hdip)(u_isn oa_wa))	31	6
1127	(((p_re(ca_wa p_csn))oa_ent)(u_hdip u_hdip))	31	6
1128	(((p_re(ca_wa p_csn))oa_ent)(u_isn u_hdip))	33	6
1129	(((u_csn(u_isn cp_ent))ca_ent)(ca_wa ca_wa))	30	6
1130	(((u_csn(u_isn cp_ent))ca_ent)(p_isn p_re))	29	6
1131	(((u_hdip(oa_wa u_csn))oa_ent)(oa_wa u_hdip))	26	6
1132	(((u_hdip(oa_wa u_csn))oa_ent)(u_isn u_hdip))	23	6
1133	(((u_hdip(u_csn cp_ent))ca_ent)(ca_wa ca_wa))	32	6
1134	(((u_hdip(u_csn cp_ent))ca_ent)(p_isn ca_wa))	25	6
1135	(((u_hdip(u_csn cp_ent))oa_ent)(oa_wa u_hdip))	50	6
1136	(((u_hdip(u_csn cp_ent))oa_ent)(u_isn u_hdip))	47	6
1137	(((u_hdip(u_isn oa_wa))u_csn)(cp_ent ca_ent))	32	6
1138	(((u_hdip(u_isn oa_wa))u_csn)(ca_ent p_isn))	32	6
1139	(((u_hdip(u_isn oa_wa))u_csn)(ca_ent ca_wa))	32	6
1140	(((u_hdip(u_isn oa_wa))u_csn)(ca_ent p_re))	30	6
1141	(((u_hdip(u_isn oa_wa))u_csn)(cp_ent ca_wa))	27	6
1142	(((u_hdip(u_isn u_csn))(u_isn cp_ent))ca_ent)	29	6
1143	((((ca_ent ca_wa)cp_ent)oa_ent)(oa_wa oa_wa))	37	6
1144	((((ca_ent ca_wa)cp_ent)oa_ent)(oa_wa u_hdip))	45	6
1145	((((ca_ent ca_wa)cp_ent)oa_ent)(u_hdip oa_wa))	33	6
1146	((((ca_ent ca_wa)cp_ent)oa_ent)(u_hdip u_hdip))	45	6
1147	((((ca_ent ca_wa)cp_ent)oa_ent)(u_isn oa_wa))	41	6
1148	((((ca_ent ca_wa)cp_ent)oa_ent)(u_isn u_hdip))	50	6
1149	((((ca_ent ca_wa)p_csn)oa_ent)(oa_wa u_hdip))	36	6
1150	((((ca_ent ca_wa)p_csn)oa_ent)(u_hdip u_hdip))	35	6
1151	((((ca_ent ca_wa)p_csn)oa_ent)(u_isn u_hdip))	41	6
1152	((((ca_ent ca_wa)p_re)(p_csn cp_ent))oa_ent)	39	6
1153	((((ca_ent p_re)p_csn)oa_ent)(oa_wa u_hdip))	33	6
1154	((((ca_ent p_re)p_csn)oa_ent)(u_hdip u_hdip))	36	6
1155	((((ca_ent p_re)p_csn)oa_ent)(u_isn u_hdip))	38	6
1156	((((ca_wa p_re)ca_wa)oa_ent)(oa_wa u_hdip))	35	6
1157	((((ca_wa p_re)ca_wa)oa_ent)(u_hdip u_hdip))	39	6
1158	((((ca_wa p_re)ca_wa)oa_ent)(u_isn u_hdip))	39	6
1159	((((ca_wa p_re)ca_wa)(p_isn cp_ent))oa_ent)	29	6
1160	((((ca_wa p_re)cp_ent)oa_ent)(oa_wa oa_wa))	40	6
1161	((((ca_wa p_re)cp_ent)oa_ent)(oa_wa u_hdip))	42	6
1162	((((ca_wa p_re)cp_ent)oa_ent)(u_hdip u_hdip))	43	6
1163	((((ca_wa p_re)cp_ent)oa_ent)(u_isn oa_wa))	41	6
1164	((((ca_wa p_re)cp_ent)oa_ent)(u_isn u_hdip))	46	6
1165	((((cp_ent ca_ent)cp_ent)oa_ent)(oa_wa oa_wa))	41	6
1166	((((cp_ent ca_ent)cp_ent)oa_ent)(oa_wa u_hdip))	43	6
1167	((((cp_ent ca_ent)cp_ent)oa_ent)(u_hdip oa_wa))	31	6
1168	((((cp_ent ca_ent)cp_ent)oa_ent)(u_hdip u_hdip))	43	6
1169	((((cp_ent ca_ent)cp_ent)oa_ent)(u_isn oa_wa))	39	6
1170	((((cp_ent ca_wa)cp_ent)oa_ent)(oa_wa oa_wa))	41	6
1171	((((cp_ent ca_wa)cp_ent)oa_ent)(oa_wa u_hdip))	42	6
1172	((((cp_ent ca_wa)cp_ent)oa_ent)(u_hdip u_hdip))	43	6
1173	((((cp_ent oa_ent)oa_wa)u_csn)(u_isn cp_ent))	52	6
1174	((((oa_ent oa_wa)u_csn)(u_isn cp_ent))ca_ent)	24	6
1175	((((oa_ent oa_wa)u_hdip)u_csn)(cp_ent oa_ent))	47	6
1176	((((oa_ent oa_wa)u_hdip)u_csn)(u_isn cp_ent))	39	6
1177	((((oa_ent oa_wa)u_hdip)(u_csn cp_ent))oa_ent)	48	6
1178	((((oa_ent u_isn)u_hdip)(oa_wa u_csn))oa_ent)	27	6
1179	((((oa_wa u_csn)p_csn)ca_ent)(ca_wa ca_wa))	31	6
1180	((((p_isn ca_wa)p_csn)oa_ent)(u_hdip u_hdip))	31	6
1181	((((p_isn ca_wa)p_csn)oa_ent)(u_isn u_hdip))	32	6
1182	((((p_re ca_wa)p_csn)oa_ent)(oa_wa u_hdip))	37	6
1183	((((p_re ca_wa)p_csn)oa_ent)(u_hdip u_hdip))	40	6
1184	((((p_re ca_wa)p_csn)oa_ent)(u_isn u_hdip))	42	6
1185	((((u_csn cp_ent)p_csn)ca_ent)(ca_wa ca_wa))	35	6
1186	((((u_csn cp_ent)p_csn)ca_ent)(ca_wa p_re))	35	6
1187	((((u_csn cp_ent)p_csn)ca_ent)(p_re ca_wa))	35	6
1188	((((u_isn oa_wa)u_hdip)(oa_wa u_csn))oa_ent)	26	6
1189	(((ca_ent ca_wa)p_re)((ca_wa p_csn)(cp_ent oa_ent)))	36	7
1190	((ca_ent p_isn)((p_re(ca_wa p_csn))(oa_ent u_hdip)))	31	7
1191	(((ca_ent ca_wa)p_re)((ca_wa p_csn)(oa_ent u_isn)))	34	7
1192	(((ca_ent ca_wa)p_re)((p_csn cp_ent)(oa_ent u_isn)))	36	7
1193	(((ca_ent ca_wa)p_re)((p_csn cp_ent)(oa_ent oa_wa)))	30	7
1194	((ca_ent ca_wa)((p_isn p_re)(ca_wa(p_csn cp_ent))))	55	7
1195	((ca_ent ca_wa)((p_isn p_re)((ca_wa p_csn)oa_ent)))	34	7
1196	((ca_ent p_isn)((p_re(ca_wa p_csn))(cp_ent oa_ent)))	36	7
1197	(((cp_ent ca_ent)(ca_wa p_re))((ca_wa p_csn)oa_ent))	32	7
1198	((cp_ent ca_ent)((p_re(ca_wa p_csn))(cp_ent oa_ent)))	34	7
1199	((cp_ent ca_ent)(((p_isn ca_wa)p_csn)(oa_ent u_hdip)))	29	7
1200	((cp_ent ca_ent)((ca_wa(p_csn cp_ent))(oa_ent u_isn)))	34	7
1201	((cp_ent ca_ent)((p_isn p_re)(ca_wa(p_csn cp_ent))))	53	7
1202	((cp_ent ca_wa)(((p_isn ca_wa)p_csn)(oa_ent u_hdip)))	27	7
1203	((cp_ent ca_wa)((p_isn p_re)(ca_wa(p_csn cp_ent))))	52	7
1204	(((oa_ent u_isn)u_hdip)((u_isn u_hdip)(oa_wa u_csn)))	42	7
1205	(((oa_wa oa_wa)(u_csn(cp_ent ca_ent)))(ca_wa p_re))	23	7
1206	(((oa_wa(u_csn cp_ent))(p_isn ca_ent))(ca_wa p_re))	30	7
1207	(((oa_wa u_csn)((u_isn cp_ent)ca_ent))(p_isn p_re))	28	7
1208	((p_csn ca_wa)((p_isn p_re)(ca_wa(p_csn cp_ent))))	38	7
1209	((p_isn ca_ent)((ca_wa p_re)((ca_wa p_csn)oa_ent)))	28	7
1210	((p_isn ca_ent)((p_re(ca_wa p_csn))(cp_ent oa_ent)))	28	7
1211	(((p_isn ca_ent)((p_isn ca_wa)p_csn))(oa_ent u_hdip))	25	7
1212	((p_isn ca_ent)((p_isn p_re)(ca_wa(p_csn cp_ent))))	44	7
1213	((p_isn ca_ent)((ca_wa p_re)(ca_wa(p_csn cp_ent))))	46	7
1214	((p_isn ca_ent)(((ca_wa p_re)ca_wa)(p_csn oa_ent)))	37	7
1215	(((u_csn(ca_ent ca_wa))(p_isn p_re))(ca_wa p_csn))	32	7
1216	((u_csn(cp_ent ca_ent))((ca_wa p_re)(p_csn oa_ent)))	24	7
1217	((u_csn cp_ent)((ca_ent ca_wa)((p_isn ca_wa)p_csn)))	27	7

811	((((p_csn ca_ent)(ca_wa p_re))((ca_wa cp_ent)oa_ent))(u_isn u_hdip))	29	9
812	((((p_csn ca_ent)(ca_wa p_re))((ca_wa cp_ent)p_csn))(oa_ent u_hdip))	30	9
813	((((p_csn ca_ent)(ca_wa p_re))((ca_wa cp_ent)p_csn))(oa_ent u_isn))	34	9
814	((((u_csn cp_ent)ca_ent)((ca_wa p_re)ca_wa))(p_csn(p_isn ca_ent)))	21	9
815	((((u_csn cp_ent)(ca_ent p_isn))((p_re ca_wa)cp_ent))(ca_ent p_isn))	26	9
816	((((u_csn cp_ent)(ca_ent p_isn))((p_re ca_wa)cp_ent))(ca_ent p_re))	24	9
817	((((u_csn cp_ent)(ca_ent p_isn))((p_re ca_wa)cp_ent))(p_isn ca_ent))	27	9
818	((((u_csn cp_ent)p_csn)((ca_ent p_isn)p_re))((ca_wa cp_ent)p_csn))	23	9
819	((((u_csn cp_ent)(ca_ent ca_wa))((p_re ca_wa)p_re))(ca_ent p_isn))	16	9
820	((((u_csn cp_ent)(ca_ent ca_wa))((p_re ca_wa)p_re))(p_isn ca_ent))	16	9
821	((((u_isn cp_ent)ca_ent)((ca_wa p_re)((ca_wa cp_ent)(p_csn ca_ent))))	20	9
822	((((u_isn cp_ent)p_csn)((p_isn ca_ent)(ca_wa p_re)))(ca_wa cp_ent))	20	9
823	((((u_isn cp_ent)(p_isn ca_ent))(ca_wa p_re))((ca_wa cp_ent)p_csn))	24	9
824	((((u_isn cp_ent)(p_isn ca_ent))((p_re ca_wa)cp_ent))(ca_ent p_isn))	20	9
825	((((u_isn u_csn)cp_ent)p_csn)((ca_ent p_isn)(p_re(ca_wa cp_ent))))	23	9
826	((((u_isn u_csn)cp_ent)(ca_ent ca_wa))(((p_re ca_wa)cp_ent)ca_ent))	23	9
827	((((u_isn u_csn)cp_ent)(p_isn ca_ent))(((p_re ca_wa)cp_ent)ca_ent))	23	9
828	((cp_ent ca_ent)((ca_wa p_re)((ca_wa cp_ent)(oa_ent(u_isn u_hdip)))))	32	9
829	((p_isn ca_ent)((ca_wa p_re)((ca_wa cp_ent)(oa_ent(u_isn u_hdip)))))	27	9
830	((u_isn cp_ent)((ca_ent ca_wa)(((p_re ca_wa)cp_ent)(p_isn ca_ent))))	20	9
831	((u_isn cp_ent)((ca_ent p_isn)(((p_re ca_wa)cp_ent)(p_isn ca_ent))))	19	9
832	((u_isn cp_ent)((p_csn(ca_ent ca_wa))(((p_re ca_wa)cp_ent)p_csn)))	18	9
833	((u_isn cp_ent)(((ca_ent ca_wa)((p_re ca_wa)cp_ent))(p_isn ca_ent)))	22	9
834	((p_csn(p_isn ca_ent))(ca_wa(((p_re ca_wa)cp_ent)(oa_ent u_isn))))	29	9
835	(((u_csn cp_ent)p_csn)((ca_ent p_isn)((p_re(ca_wa cp_ent))p_csn)))	22	9
836	(((u_csn cp_ent)((p_isn ca_ent)(ca_wa p_re)))((ca_wa cp_ent)p_csn))	28	9
837	(((u_isn cp_ent)(p_csn((ca_ent p_isn)p_re)))((ca_wa cp_ent)ca_ent))	22	9
838	(((u_isn cp_ent)(p_csn(p_isn ca_ent)))((ca_wa p_re)(ca_wa cp_ent)))	20	9
839	(((p_csn(ca_ent ca_wa))((p_re(ca_wa cp_ent))p_csn))(oa_ent u_isn))	33	9
840	(((p_csn(p_isn ca_ent))((p_re(ca_wa cp_ent))p_csn))(oa_ent u_isn))	32	9
841	((((u_csn cp_ent)p_csn)((ca_ent ca_wa)(p_re(ca_wa cp_ent))))p_csn)	25	9
842	((((u_isn cp_ent)p_csn)(((ca_ent p_isn)p_re)cp_ent))(ca_ent p_isn))	18	9
843	((((u_isn cp_ent)p_csn)(((ca_ent p_isn)p_re)cp_ent))(ca_ent p_re))	18	9
844	((((u_isn u_csn)cp_ent)((ca_ent ca_wa)(p_re(ca_wa cp_ent))))p_csn)	24	9
845	((((u_isn u_csn)cp_ent)((ca_ent ca_wa)((p_re ca_wa)cp_ent)))p_csn)	23	9
846	((((u_isn u_csn)cp_ent)((ca_ent p_isn)((p_re ca_wa)cp_ent)))p_csn)	25	9
847	((((u_isn u_csn)cp_ent)(((p_isn ca_ent)p_re)(ca_wa cp_ent)))p_csn)	28	9
848	(((p_csn((ca_ent p_isn)p_re))((ca_wa cp_ent)p_csn))(oa_ent u_hdip))	30	9
849	(((p_csn((ca_ent p_isn)p_re))((ca_wa cp_ent)p_csn))(oa_ent u_isn))	34	9
850	((((u_isn cp_ent)(p_csn(p_isn ca_ent)))((ca_wa p_re)(ca_wa cp_ent)))	21	9
851	((((u_isn cp_ent)(p_csn(p_isn ca_ent)))((ca_wa p_re)ca_wa))p_csn)	24	9
852	((((u_isn cp_ent)((ca_ent ca_wa)p_re))(ca_wa cp_ent))(p_isn ca_ent))	22	9
853	((((u_isn cp_ent)((ca_ent p_isn)p_re))(ca_wa cp_ent))(p_isn ca_ent))	26	9
854	((((u_isn cp_ent)p_csn)(p_isn ca_ent))((ca_wa p_re)(ca_wa cp_ent)))	23	9
855	(((((u_isn u_csn)cp_ent)ca_ent)((ca_wa p_re)ca_wa))(cp_ent ca_ent))	23	9
856	(((((u_isn u_csn)cp_ent)p_csn)(ca_ent p_isn))(p_re(ca_wa cp_ent)))	23	9
857	(((((u_isn u_csn)cp_ent)p_csn)(ca_ent p_re))((ca_wa cp_ent)p_csn))	23	9
858	(((((u_isn u_csn)cp_ent)p_csn)((ca_ent p_isn)p_re))(ca_wa cp_ent))	23	9
859	(((((u_isn u_csn)cp_ent)(p_isn ca_ent))(ca_wa ca_wa))(p_re p_re))	21	9
860	(((((u_isn u_csn)cp_ent)(p_isn ca_ent))(ca_wa p_re))(ca_wa cp_ent))	30	9
861	((u_isn cp_ent)(((p_csn(p_isn ca_ent))(ca_wa p_re))((ca_wa cp_ent)p_csn)))	17	10
862	(((u_csn cp_ent)(ca_ent p_isn))(((p_re ca_wa)cp_ent)(p_csn(p_isn ca_ent))))	22	10
863	(((u_csn cp_ent)(ca_ent p_isn))(((p_re ca_wa)cp_ent)((ca_ent p_isn)p_re)))	19	10
864	(((u_csn cp_ent)(ca_ent p_isn))(((p_re ca_wa)cp_ent)((p_csn ca_ent)p_re)))	21	10
865	(((u_csn cp_ent)(ca_ent ca_wa))(((p_re ca_wa)cp_ent)((p_isn ca_ent)p_re)))	22	10
866	(((u_isn cp_ent)(ca_ent p_isn))(((p_re ca_wa)cp_ent)((p_isn ca_ent)p_re)))	26	10
867	(((u_isn cp_ent)(ca_ent ca_wa))(((p_re ca_wa)cp_ent)(p_csn(p_isn ca_ent))))	23	10
868	(((u_isn cp_ent)(ca_ent ca_wa))(((p_re ca_wa)cp_ent)((p_isn ca_ent)p_re)))	23	10
869	((p_csn((p_isn ca_ent)(ca_wa p_re)))(((ca_wa cp_ent)p_csn)(ca_ent p_re)))	25	10
870	((u_isn cp_ent)((p_csn(p_isn ca_ent))(((p_re ca_wa)cp_ent)(p_csn ca_ent))))	24	10
871	(((u_isn cp_ent)((ca_ent ca_wa)p_re))((ca_wa cp_ent)(p_csn(p_isn ca_ent))))	17	10
872	(((u_isn cp_ent)((ca_ent ca_wa)p_re))(((ca_wa cp_ent)p_csn)(p_isn ca_ent)))	19	10
873	(((u_isn cp_ent)((ca_ent p_isn)p_re))((ca_wa cp_ent)(p_csn(p_isn ca_ent))))	20	10
874	(((u_isn cp_ent)((ca_ent p_isn)p_re))(((ca_wa cp_ent)p_csn)(p_isn ca_ent)))	22	10
875	(((u_isn cp_ent)((p_csn ca_ent)(ca_wa p_re)))((ca_wa cp_ent)(p_isn ca_ent)))	21	10
876	(((u_isn cp_ent)((p_csn ca_ent)(ca_wa p_re)))(((ca_wa cp_ent)p_csn)ca_ent))	19	10
877	(((u_isn cp_ent)((p_isn ca_ent)(ca_wa p_re)))((ca_wa cp_ent)(p_isn ca_ent)))	22	10
878	(((u_isn cp_ent)((p_isn ca_ent)(ca_wa p_re)))(((ca_wa cp_ent)p_csn)ca_ent))	20	10
879	(((u_csn cp_ent)(ca_ent p_isn))(((p_re ca_wa)cp_ent)(p_csn(p_isn ca_ent))))	25	10
880	((((u_csn cp_ent)(ca_ent p_isn))((p_re ca_wa)cp_ent))((ca_ent p_isn)p_re))	22	10
881	((((u_csn cp_ent)(ca_ent p_isn))((p_re ca_wa)cp_ent))((p_isn ca_ent)p_re))	24	10
882	((((u_csn cp_ent)(ca_ent ca_wa))((p_re ca_wa)cp_ent))(p_csn(p_isn ca_ent)))	23	10
883	((((u_csn cp_ent)(ca_ent ca_wa))((p_re ca_wa)cp_ent))((ca_ent p_isn)p_re))	21	10
884	((((u_isn cp_ent)p_csn)((ca_ent ca_wa)p_re))((ca_wa cp_ent)(p_isn ca_ent)))	22	10
885	((((u_isn cp_ent)p_csn)((ca_ent ca_wa)p_re))(((ca_wa cp_ent)p_csn)ca_ent))	20	10
886	((((u_isn cp_ent)p_csn)((p_isn ca_ent)(ca_wa p_re)))((ca_wa cp_ent)p_csn))	19	10
887	((((u_isn cp_ent)(ca_ent ca_wa))(p_re(ca_wa cp_ent)))(p_csn(p_isn ca_ent)))	21	10
888	((((u_isn cp_ent)(ca_ent ca_wa))((p_re ca_wa)cp_ent))(p_csn(p_isn ca_ent)))	24	10
889	((((u_isn u_csn)cp_ent)(ca_ent ca_wa))(((p_re ca_wa)cp_ent)(ca_ent p_isn)))	23	10
890	((((u_isn u_csn)cp_ent)(ca_ent ca_wa))(((p_re ca_wa)cp_ent)(p_isn ca_ent)))	24	10
891	((((u_isn u_csn)cp_ent)(p_isn ca_ent))(((p_re ca_wa)cp_ent)(ca_ent p_isn)))	23	10
892	((((u_isn u_csn)cp_ent)(p_isn ca_ent))(((p_re ca_wa)cp_ent)(p_isn ca_ent)))	24	10
893	((u_isn cp_ent)((p_csn(p_isn ca_ent))(ca_wa p_re)))((ca_wa cp_ent)p_csn))	16	10
894	((((u_isn u_csn)cp_ent)ca_ent)(ca_wa(((p_re ca_wa)cp_ent)(p_isn ca_ent))))	21	10
895	((((u_isn cp_ent)(p_csn((ca_ent ca_wa)p_re)))((ca_wa cp_ent)(p_isn ca_ent)))	20	10
896	((((u_isn cp_ent)(p_csn((ca_ent ca_wa)p_re)))(((ca_wa cp_ent)p_csn)ca_ent))	18	10
897	((((u_isn cp_ent)(p_csn((ca_ent p_isn)p_re)))((ca_wa cp_ent)(p_isn ca_ent)))	23	10
898	((((u_isn cp_ent)ca_ent)(((p_re ca_wa)cp_ent)(p_isn ca_ent)))(p_re ca_wa))	21	10
899	((((u_isn cp_ent)p_csn)(((ca_ent p_isn)p_re)cp_ent))((ca_ent ca_wa)p_re))	17	10
900	((((u_isn cp_ent)p_csn)(((ca_ent p_isn)p_re)cp_ent))((ca_ent p_re)cp_ent))	18	10
901	((((u_isn u_csn)cp_ent)((p_isn ca_ent)(ca_wa p_re)))((ca_wa cp_ent)p_csn))	27	10
902	(((p_csn((p_isn ca_ent)(ca_wa p_re)))((ca_wa cp_ent)p_csn))(oa_ent u_isn))	32	10
903	((((u_isn cp_ent)(p_csn(p_isn ca_ent)))(ca_wa p_re))((ca_wa cp_ent)p_csn))	20	10
904	((((u_isn cp_ent)((ca_ent ca_wa)p_re))(ca_wa cp_ent))(p_csn(p_isn ca_ent)))	17	10
905	((((u_isn cp_ent)((ca_ent p_isn)p_re))(ca_wa cp_ent))(p_csn(p_isn ca_ent)))	20	10
906	((((u_isn cp_ent)((p_isn ca_ent)(ca_wa p_re)))(ca_wa cp_ent))(p_csn ca_ent))	18	10
907	((((u_isn cp_ent)((p_isn ca_ent)(ca_wa p_re)))(ca_wa cp_ent))(p_isn ca_ent))	22	10
908	(((((u_csn cp_ent)(ca_ent ca_wa))((p_re ca_wa)cp_ent))p_csn)(p_isn ca_ent))	22	10
909	(((((u_isn cp_ent)ca_ent)(ca_wa p_re))(ca_wa cp_ent))(p_csn(p_isn ca_ent)))	22	10
910	(((((u_isn cp_ent)p_csn)(p_isn ca_ent))(ca_wa p_re))((ca_wa cp_ent)p_csn))	22	10
911	(((((u_isn cp_ent)p_csn)((ca_ent ca_wa)p_re))(ca_wa cp_ent))(p_csn ca_ent))	18	10
912	(((((u_isn cp_ent)p_csn)((ca_ent ca_wa)p_re))(ca_wa cp_ent))(p_isn ca_ent))	21	10
913	(((((u_isn cp_ent)p_csn)((ca_ent p_isn)p_re))(ca_wa cp_ent))(p_csn ca_ent))	19	10
914	(((((u_isn cp_ent)(p_isn ca_ent))(ca_wa p_re))(ca_wa cp_ent))(p_csn ca_ent))	21	10
915	(((((u_isn u_csn)cp_ent)(ca_ent p_isn))((p_re ca_wa)cp_ent))(ca_ent p_isn))	25	10
916	(((((u_isn u_csn)cp_ent)(ca_ent p_isn))((p_re ca_wa)cp_ent))(ca_ent p_re))	23	10
917	(((((u_isn u_csn)cp_ent)(ca_ent p_isn))((p_re ca_wa)cp_ent))(p_isn ca_ent))	25	10
918	(((((u_isn u_csn)cp_ent)p_csn)((ca_ent p_isn)p_re))((ca_wa cp_ent)p_csn))	22	10
919	(((((u_isn u_csn)cp_ent)(ca_ent ca_wa))((p_re ca_wa)cp_ent))(ca_ent p_re))	21	10
920	(((((u_isn u_csn)cp_ent)(ca_ent ca_wa))((p_re ca_wa)p_re))(ca_ent p_isn))	16	10
921	(((((u_isn u_csn)cp_ent)(ca_ent ca_wa))((p_re ca_wa)p_re))(p_isn ca_ent))	16	10
922	(((((u_isn u_csn)cp_ent)(p_isn ca_ent))(ca_wa(p_re ca_wa)))(cp_ent ca_ent))	20	10
923	(((((u_isn u_csn)cp_ent)(p_isn ca_ent))((p_re ca_wa)cp_ent))(ca_ent p_isn))	25	10
924	(((((u_isn u_csn)cp_ent)(p_isn ca_ent))((p_re ca_wa)cp_ent))(ca_ent p_re))	23	10
925	(((((u_isn u_csn)cp_ent)(p_isn ca_ent))((p_re ca_wa)cp_ent))(p_isn ca_ent))	26	10
926	((u_isn cp_ent)(((p_isn ca_ent)(ca_wa p_re))(((ca_wa cp_ent)p_csn)(ca_ent p_isn))))	20	11
927	(((((u_csn cp_ent)(ca_ent ca_wa))((p_re ca_wa)cp_ent))p_csn)((p_isn ca_ent)p_re))	19	11
928	((((u_csn cp_ent)(ca_ent p_isn))((p_re ca_wa)cp_ent))(p_csn((p_isn ca_ent)p_re)))	22	11
929	((((u_csn cp_ent)(ca_ent ca_wa))((p_re ca_wa)cp_ent))(p_csn((p_isn ca_ent)p_re)))	20	11
930	((((u_isn cp_ent)p_csn)((ca_ent ca_wa)p_re))(((ca_wa cp_ent)p_csn)(ca_ent p_isn)))	20	11
931	((((u_isn cp_ent)p_csn)((ca_ent ca_wa)p_re))(((ca_wa cp_ent)p_csn)(p_isn ca_ent)))	21	11
932	((((u_isn cp_ent)p_csn)((p_isn ca_ent)(ca_wa p_re)))((ca_wa cp_ent)(p_isn ca_ent)))	20	11
933	((((u_isn cp_ent)p_csn)((p_isn ca_ent)(ca_wa p_re)))(((ca_wa cp_ent)p_csn)ca_ent))	20	11
934	((((u_isn u_csn)cp_ent)(ca_ent ca_wa))(((p_re ca_wa)cp_ent)((ca_ent p_isn)p_re)))	18	11
935	((((u_isn u_csn)cp_ent)(ca_ent p_isn))(((p_re ca_wa)cp_ent)((p_csn ca_ent)p_re)))	20	11
936	((((u_isn u_csn)cp_ent)(ca_ent ca_wa))(((p_re ca_wa)cp_ent)((p_isn ca_ent)p_re)))	21	11
937	((((u_isn u_csn)cp_ent)(ca_ent ca_wa))(((p_re ca_wa)cp_ent)(p_csn(p_isn ca_ent))))	21	11
938	((((u_isn u_csn)cp_ent)(ca_ent p_isn))(((p_re ca_wa)cp_ent)((ca_ent p_isn)p_re)))	17	11
939	((((u_isn u_csn)cp_ent)(p_isn ca_ent))(((p_re ca_wa)cp_ent)(p_csn(p_isn ca_ent))))	21	11
940	((((u_isn u_csn)cp_ent)(p_isn ca_ent))(((p_re ca_wa)cp_ent)((ca_ent p_isn)p_re)))	18	11
941	((((u_isn u_csn)cp_ent)(p_isn ca_ent))(((p_re ca_wa)cp_ent)((p_csn ca_ent)p_re)))	20	11
942	((((u_isn u_csn)cp_ent)(p_isn ca_ent))(((p_re ca_wa)cp_ent)((p_isn ca_ent)p_re)))	21	11
943	(((((u_csn cp_ent)(ca_ent ca_wa))((p_re ca_wa)cp_ent))p_csn)((ca_ent p_isn)p_re))	19	11
944	((((u_csn cp_ent)(ca_ent p_isn))((p_re ca_wa)cp_ent)p_csn)((ca_ent p_isn)p_re))	20	11
945	((((u_isn u_csn)cp_ent)(p_isn ca_ent))(ca_wa(((p_re ca_wa)cp_ent)(p_csn ca_ent))))	20	11
946	(((u_isn cp_ent)(p_csn((ca_ent ca_wa)p_re)))(((ca_wa cp_ent)p_csn)(ca_ent p_isn)))	18	11
947	(((u_isn cp_ent)(p_csn((p_isn ca_ent)(ca_wa p_re))))(((ca_wa cp_ent)p_csn)ca_ent))	16	11
948	(((u_isn cp_ent)((p_csn(p_isn ca_ent))(ca_wa p_re)))(((ca_wa cp_ent)p_csn)ca_ent))	20	11
949	((((u_isn cp_ent)p_csn)((ca_ent ca_wa)((p_re ca_wa)cp_ent)))((p_isn ca_ent)p_re))	18	11
950	((((u_isn cp_ent)p_csn)((p_isn ca_ent)((p_re ca_wa)cp_ent)))(p_csn(p_isn ca_ent)))	17	11
951	((((u_isn cp_ent)(ca_ent ca_wa))(((p_re ca_wa)cp_ent)(p_isn ca_ent)))(p_re ca_wa))	21	11
952	(((((u_csn cp_ent)(ca_ent p_isn))((p_re ca_wa)cp_ent))p_csn)((ca_ent p_isn)p_re))	20	11
953	(((((u_csn cp_ent)(ca_ent p_isn))((p_re ca_wa)cp_ent))p_csn)((p_isn ca_ent)p_re))	21	11
954	(((((u_isn cp_ent)p_csn)(p_isn ca_ent))(ca_wa p_re))((ca_wa cp_ent)(p_csn ca_ent)))	19	11
955	(((((u_isn cp_ent)p_csn)((ca_ent ca_wa)p_re))(ca_wa cp_ent))((p_isn ca_ent)p_re))	21	11
956	(((((u_isn cp_ent)p_csn)(p_isn ca_ent))(ca_wa p_re))((ca_wa cp_ent)(p_csn ca_ent)))	17	11
957	(((((u_isn u_csn)cp_ent)(ca_ent p_isn))((p_re ca_wa)cp_ent))(p_csn(p_isn ca_ent)))	24	11
958	(((((u_isn u_csn)cp_ent)(ca_ent p_isn))((p_re ca_wa)cp_ent))((ca_ent p_isn)p_re))	21	11
959	(((((u_isn u_csn)cp_ent)(ca_ent p_isn))((p_re ca_wa)cp_ent))((p_isn ca_ent)p_re))	23	11
960	(((((u_isn u_csn)cp_ent)(ca_ent ca_wa))((p_re ca_wa)cp_ent))(p_csn(p_isn ca_ent)))	22	11
961	(((((u_isn u_csn)cp_ent)(ca_ent ca_wa))((p_re ca_wa)cp_ent))((ca_ent p_isn)p_re))	20	11
962	(((((u_isn u_csn)cp_ent)(p_isn ca_ent))((ca_wa p_re)ca_wa))((cp_ent ca_ent)cp_ent))	21	11
963	(((((u_isn u_csn)cp_ent)(p_isn ca_ent))((p_re ca_wa)cp_ent))(p_csn(p_isn ca_ent)))	24	11
964	(((((u_isn u_csn)cp_ent)(p_isn ca_ent))((p_re ca_wa)cp_ent))((ca_ent p_isn)cp_ent))	20	11
965	(((((u_isn u_csn)cp_ent)(p_isn ca_ent))((p_re ca_wa)cp_ent))((ca_ent p_isn)p_re))	21	11
966	(((((u_isn u_csn)cp_ent)(p_isn ca_ent))((p_re ca_wa)cp_ent))((p_isn ca_ent)p_re))	23	11
967	(((u_isn cp_ent)(p_csn(ca_ent ca_wa)))((((p_re ca_wa)cp_ent)p_csn)(ca_ent p_isn)))	18	11
968	(((u_isn cp_ent)(p_csn(ca_ent ca_wa)))((((p_re ca_wa)cp_ent)p_csn)(ca_ent p_re)))	18	11
969	((((u_isn cp_ent)p_csn)(((ca_ent p_isn)p_re)cp_ent))((ca_ent ca_wa)(p_re ca_wa)))	17	11
970	((((u_isn cp_ent)(p_csn(p_isn ca_ent)))(ca_wa p_re))(((ca_wa cp_ent)p_csn)(ca_ent p_isn)))	19	12
971	((((u_isn cp_ent)(ca_ent p_isn))((p_re ca_wa)cp_ent))(((ca_ent p_re)cp_ent)(oa_ent u_isn)))	19	12
972	((((u_isn cp_ent)p_csn)((p_isn ca_ent)(ca_wa p_re)))((ca_wa cp_ent)(p_csn(p_isn ca_ent))))	18	12
973	((((u_isn cp_ent)p_csn)((p_isn ca_ent)(ca_wa p_re)))(((ca_wa cp_ent)p_csn)(ca_ent p_isn)))	20	12
974	((((u_isn cp_ent)p_csn)((p_isn ca_ent)(ca_wa p_re)))(((ca_wa cp_ent)p_csn)(p_isn ca_ent)))	20	12
975	((((u_isn u_csn)cp_ent)(ca_ent ca_wa))(((p_re ca_wa)cp_ent)(p_csn((ca_ent p_isn)p_re))))	18	12
976	((((u_isn u_csn)cp_ent)(ca_ent ca_wa))(((p_re ca_wa)cp_ent)(p_csn((p_isn ca_ent)p_re))))	19	12
977	((((u_isn u_csn)cp_ent)(ca_ent ca_wa))((((p_re ca_wa)cp_ent)p_csn)((ca_ent p_isn)p_re)))	19	12
978	((((u_isn u_csn)cp_ent)(p_isn ca_ent))(((p_re ca_wa)cp_ent)(p_csn((ca_ent p_isn)p_re))))	18	12
979	((((u_isn u_csn)cp_ent)(p_isn ca_ent))(((p_re ca_wa)cp_ent)(p_csn((p_isn ca_ent)p_re))))	19	12
980	((((u_isn u_csn)cp_ent)(p_isn ca_ent))((((p_re ca_wa)cp_ent)p_csn)((ca_ent p_isn)p_re)))	19	12
981	(((u_isn cp_ent)(p_csn((p_isn ca_ent)(ca_wa p_re))))(((ca_wa cp_ent)p_csn)(ca_ent p_isn)))	16	12
982	(((u_isn cp_ent)((p_isn ca_ent)((p_re ca_wa)cp_ent)))(((ca_ent p_re)cp_ent)(oa_ent u_isn)))	18	12
983	(((u_isn cp_ent)((p_csn(p_isn ca_ent))(ca_wa p_re)))(((ca_wa cp_ent)p_csn)(p_isn ca_ent)))	20	12
984	(((u_isn cp_ent)((p_csn(p_isn ca_ent))(ca_wa p_re)))((ca_wa cp_ent)(p_csn(p_isn ca_ent))))	18	12
985	(((u_isn cp_ent)((p_csn(p_isn ca_ent))(ca_wa p_re)))(((ca_wa cp_ent)p_csn)(ca_ent p_isn)))	20	12
986	(((((u_isn cp_ent)p_csn)((p_isn ca_ent)(ca_wa p_re)))(ca_wa cp_ent))((p_isn ca_ent)p_re))	19	12
987	(((((u_isn cp_ent)p_csn)((p_isn ca_ent)(ca_wa p_re)))((ca_wa cp_ent)p_csn))(ca_ent p_isn))	19	12
988	(((((u_isn cp_ent)p_csn)((p_isn ca_ent)(ca_wa p_re)))((ca_wa cp_ent)p_csn))(ca_ent p_re))	19	12
989	(((((u_isn u_csn)cp_ent)(ca_ent p_isn))((p_re ca_wa)cp_ent))(p_csn((p_isn ca_ent)p_re)))	21	12
990	(((((u_isn u_csn)cp_ent)(p_isn ca_ent))((p_re ca_wa)cp_ent))(p_csn((p_isn ca_ent)p_re)))	21	12
991	(((u_isn cp_ent)((p_csn(ca_ent ca_wa))(((p_re ca_wa)cp_ent)p_csn)))((ca_ent p_re)cp_ent))	17	12
992	(((u_isn cp_ent)((p_csn(p_isn ca_ent))(ca_wa p_re)))((ca_wa cp_ent)((p_isn ca_ent)p_re)))	19	12
993	(((u_isn cp_ent)((p_csn(p_isn ca_ent))(ca_wa p_re)))(((ca_wa cp_ent)p_csn)(ca_ent p_re)))	19	12
994	((((u_isn cp_ent)p_csn)((ca_ent ca_wa)((p_re ca_wa)cp_ent)))(((ca_ent p_re)cp_ent)(oa_ent u_isn)))	17	13
995	((((u_isn cp_ent)p_csn)((p_isn ca_ent)((p_re ca_wa)cp_ent)))(((ca_ent p_re)cp_ent)(oa_ent u_isn)))	16	13
996	((((u_isn cp_ent)((ca_ent p_isn)p_re))((ca_wa cp_ent)(p_csn(p_isn ca_ent))))((p_re ca_wa)cp_ent))	18	13
997	((((u_isn cp_ent)((ca_ent p_isn)p_re))(((ca_wa cp_ent)p_csn)(p_isn ca_ent)))((p_re ca_wa)cp_ent))	20	13
998	(((((u_isn cp_ent)p_csn)((ca_ent ca_wa)((p_re ca_wa)cp_ent)))((ca_ent p_re)cp_ent))(oa_ent u_isn))	18	13
999	(((((u_isn cp_ent)p_csn)((p_isn ca_ent)(ca_wa p_re)))((ca_wa cp_ent)p_csn))((ca_ent p_re)cp_ent))	18	13
1000	(((((u_isn cp_ent)p_csn)((ca_ent ca_wa)((p_re ca_wa)cp_ent)))((p_isn ca_ent)p_re))(oa_ent u_hdip))	16	13
1001	(((((u_isn cp_ent)p_csn)((ca_ent ca_wa)((p_re ca_wa)cp_ent)))((p_isn ca_ent)p_re))(oa_ent u_isn))	18	13
1002	(((((u_isn cp_ent)p_csn)((ca_ent ca_wa)((p_re ca_wa)cp_ent)))((p_isn ca_ent)p_re))(p_csn oa_ent))	18	13
1003	(((((u_isn cp_ent)p_csn)((p_isn ca_ent)((p_re ca_wa)cp_ent)))((p_isn ca_ent)p_re))(oa_ent u_isn))	17	13
1004	(((((u_isn cp_ent)p_csn)((p_isn ca_ent)((p_re ca_wa)cp_ent)))((p_isn ca_ent)p_re))(p_csn oa_ent))	17	13
1005	(((((u_isn cp_ent)p_csn)((ca_ent p_isn)p_re))((ca_wa cp_ent)(p_csn ca_ent)))((p_re ca_wa)cp_ent))	17	13
1006	(((((u_isn cp_ent)p_csn)((p_isn ca_ent)(ca_wa p_re)))(((ca_wa cp_ent)p_csn)(ca_ent ca_wa)))(p_re ca_wa))	19	14
1007	(((((u_isn cp_ent)p_csn)((ca_ent ca_wa)((p_re ca_wa)cp_ent)))((p_isn ca_ent)p_re))(p_csn(oa_ent u_isn)))	18	14
1008	(((((u_isn cp_ent)p_csn)((p_isn ca_ent)((p_re ca_wa)cp_ent)))((p_isn ca_ent)p_re))(p_csn(oa_ent u_isn)))	17	14
1009	((((((u_isn cp_ent)p_csn)((p_isn ca_ent)(ca_wa p_re)))((ca_wa cp_ent)p_csn))((ca_ent ca_wa)(p_re ca_wa)))cp_ent)	19	15
1010	((((((u_isn cp_ent)p_csn)((p_isn ca_ent)(ca_wa p_re)))((ca_wa cp_ent)p_csn))((p_isn ca_ent)(p_re ca_wa)))cp_ent)	19	15

1218	(((u_csn cp_ent)(ca_ent ca_wa))((p_re ca_wa)oa_ent))	26	7
1219	((u_csn cp_ent)((ca_ent p_isn)((p_re ca_wa)p_csn)))	36	7
1220	((u_hdip u_hdip)((oa_wa (u_csn cp_ent))(ca_ent p_isn)))	31	7
1221	((u_hdip u_hdip)((oa_wa (u_csn cp_ent))(ca_ent ca_wa)))	31	7
1222	((u_hdip u_hdip)((oa_wa u_csn)((cp_ent oa_ent)oa_wa)))	38	7
1223	((u_hdip u_hdip)((oa_wa (u_csn cp_ent))(p_csn ca_ent)))	25	7
1224	((ca_ent ca_wa)((p_re (ca_wa p_csn))(cp_ent oa_ent)))	37	7
1225	(((ca_ent ca_wa)((p_isn ca_wa)p_csn))(oa_ent u_hdip))	32	7
1226	((ca_wa (p_csn cp_ent))((p_csn oa_ent)(u_isn oa_wa)))	19	7
1227	((oa_ent (oa_wa u_hdip))((u_isn u_hdip)(oa_wa u_csn)))	39	7
1228	((oa_ent (u_isn u_hdip))((u_isn u_hdip)(oa_wa u_csn)))	40	7
1229	((p_csn (ca_ent ca_wa))((p_re ca_wa)(cp_ent oa_ent)))	37	7
1230	((p_csn (ca_wa p_re))((ca_wa p_csn)(cp_ent oa_ent)))	26	7
1231	((p_csn (ca_wa p_re))((p_csn cp_ent)(oa_ent u_isn)))	30	7
1232	((p_csn (p_isn ca_ent))((ca_wa ca_wa)(cp_ent oa_ent)))	26	7
1233	((p_csn (p_isn ca_ent))((p_re ca_wa)(cp_ent oa_ent)))	25	7
1234	((p_csn (p_isn ca_wa))((p_re ca_wa)(cp_ent oa_ent)))	33	7
1235	((p_csn (p_isn oa_ent))((oa_wa u_hdip)(oa_wa u_csn)))	28	7
1236	((p_re (ca_wa p_csn))((p_isn cp_ent)(oa_ent u_isn)))	24	7
1237	((u_csn (ca_ent ca_wa))((cp_ent oa_ent)(u_isn u_hdip)))	35	7
1238	((u_csn (ca_ent ca_wa))((p_csn oa_ent)(u_isn u_hdip)))	30	7
1239	(((u_csn (cp_ent ca_wa))(p_isn p_re))(ca_wa p_csn))	27	7
1240	((u_csn (cp_ent ca_wa))((p_re ca_wa)(cp_ent oa_ent)))	26	7
1241	(((u_csn cp_ent)(p_csn (ca_ent ca_wa)))(p_re ca_wa))	33	7
1242	(((u_csn (p_isn ca_ent))(p_isn p_re))(ca_wa p_csn))	26	7
1243	((u_csn (p_isn ca_ent))((ca_wa p_re)(p_csn oa_ent)))	20	7
1244	((u_csn cp_ent)((p_isn ca_ent)((p_isn ca_wa)p_csn)))	22	7
1245	((u_csn cp_ent)((p_isn ca_ent)((p_re ca_wa)p_csn)))	29	7
1246	((u_csn (p_isn ca_ent))((p_re ca_wa)(cp_ent oa_ent)))	27	7
1247	((u_csn (u_isn cp_ent))((p_isn ca_ent)(ca_wa p_re)))	26	7
1248	((u_hdip (oa_wa u_csn))((cp_ent oa_ent)(u_isn u_hdip)))	41	7
1249	((u_hdip (u_csn cp_ent))((ca_ent p_isn)(ca_wa p_csn)))	24	7
1250	((u_hdip (u_csn cp_ent))((p_isn ca_ent)(ca_wa p_csn)))	18	7
1251	((u_hdip (u_csn cp_ent))((p_isn oa_ent)(oa_wa u_hdip)))	33	7
1252	((u_hdip (u_isn oa_wa))((u_csn cp_ent)(ca_ent p_isn)))	31	7
1253	((u_hdip (u_isn oa_wa))((u_csn cp_ent)(p_csn ca_ent)))	25	7
1254	((u_hdip (u_isn oa_wa))((u_csn cp_ent)(ca_ent ca_wa)))	33	7
1255	((u_hdip (u_isn oa_wa))((u_csn cp_ent)(oa_ent u_hdip)))	48	7
1256	((u_hdip (u_isn oa_wa))((u_csn cp_ent)(oa_wa u_hdip)))	24	7
1257	((u_hdip (u_isn u_csn))((u_isn cp_ent)(ca_ent p_isn)))	27	7
1258	((u_hdip (u_isn u_csn))((u_isn cp_ent)(ca_ent ca_wa)))	27	7
1259	(((ca_ent ca_wa)cp_ent)((p_csn oa_ent)(oa_wa u_hdip)))	29	7
1260	(((ca_ent ca_wa)cp_ent)((p_csn oa_ent)(u_isn oa_wa)))	28	7
1261	(((ca_ent ca_wa)cp_ent)((p_csn oa_ent)(u_isn u_hdip)))	28	7
1262	(((ca_ent ca_wa)cp_ent)((p_isn oa_ent)(u_isn u_hdip)))	34	7
1263	(((ca_ent ca_wa)p_csn)((p_isn oa_ent)(u_isn u_hdip)))	30	7
1264	((((ca_ent ca_wa)p_re)(p_csn cp_ent))(oa_ent oa_wa))	32	7
1265	((((ca_ent ca_wa)p_re)(p_csn cp_ent))(oa_ent u_hdip))	33	7
1266	(((ca_ent ca_wa)(p_csn cp_ent))(p_csn (oa_ent u_isn)))	22	7
1267	(((ca_ent ca_wa)(p_csn cp_ent))((p_csn oa_ent)oa_wa))	21	7
1268	(((ca_ent p_isn)(p_re (ca_wa p_csn)))(cp_ent oa_ent))	37	7
1269	(((ca_ent p_isn)(p_re (ca_wa p_csn)))(oa_ent u_hdip))	33	7
1270	(((ca_ent p_isn)(p_csn cp_ent))((p_csn oa_ent)oa_wa))	20	7
1271	((((ca_ent p_re)p_csn)(p_isn oa_ent))(u_isn u_hdip))	28	7
1272	(((ca_ent p_re)(p_csn cp_ent))((p_csn oa_ent)oa_wa))	20	7
1273	((((ca_wa p_re)ca_wa)(cp_ent oa_ent))(oa_wa u_hdip))	41	7
1274	((((ca_wa p_re)ca_wa)(cp_ent oa_ent))(u_isn u_hdip))	41	7
1275	((((ca_wa p_re)ca_wa)(p_csn cp_ent))(oa_ent u_isn))	39	7
1276	((((ca_wa p_re)ca_wa)(p_csn cp_ent))(oa_ent oa_wa))	32	7
1277	((((ca_wa p_re)ca_wa)(p_csn cp_ent))(oa_ent u_hdip))	33	7
1278	((((ca_wa p_re)ca_wa)(p_csn cp_ent))(p_csn oa_ent))	22	7
1279	((((ca_wa p_re)ca_wa)(p_csn oa_ent))(oa_wa u_hdip))	37	7
1280	((((ca_wa p_re)ca_wa)(p_csn oa_ent))(u_isn oa_wa))	32	7
1281	((((ca_wa p_re)ca_wa)(p_csn oa_ent))(u_isn u_hdip))	40	7
1282	((((ca_wa p_re)ca_wa)(p_isn cp_ent))(oa_ent u_isn))	27	7
1283	((((ca_wa p_re)cp_ent)(p_isn oa_ent))(u_isn u_hdip))	29	7
1284	(((ca_wa p_re)(p_csn cp_ent))(oa_ent (oa_wa u_hdip)))	29	7
1285	(((ca_wa p_re)(p_csn cp_ent))(oa_ent (u_isn u_hdip)))	33	7
1286	(((ca_wa p_re)(p_csn cp_ent))(p_csn (oa_ent u_isn)))	22	7
1287	(((ca_wa p_re)(p_csn cp_ent))((oa_ent u_isn)u_hdip))	34	7
1288	((((cp_ent ca_ent)cp_ent)(p_isn oa_ent))(u_isn u_hdip))	34	7
1289	(((cp_ent ca_ent)(ca_wa p_re))(ca_wa (p_csn cp_ent)))	56	7
1290	(((cp_ent ca_ent)(ca_wa p_re))(p_csn (cp_ent oa_ent)))	30	7
1291	(((cp_ent ca_ent)(ca_wa p_re))(p_csn (oa_ent u_isn)))	39	7
1292	(((cp_ent ca_ent)(ca_wa p_re))((p_csn oa_ent)u_hdip))	30	7
1293	((((cp_ent ca_wa)cp_ent)(p_isn oa_ent))(u_isn u_hdip))	32	7
1294	((((cp_ent oa_ent)oa_wa)(u_isn u_hdip))(oa_wa u_csn))	41	7
1295	((((cp_ent oa_ent)u_hdip)(u_isn u_hdip))(oa_wa u_csn))	42	7
1296	(((cp_ent oa_ent)(oa_wa u_hdip))(oa_wa (u_csn cp_ent)))	52	7
1297	(((cp_ent oa_ent)(oa_wa u_hdip))((u_isn oa_wa)u_csn))	49	7
1298	(((cp_ent oa_ent)(u_isn u_hdip))(oa_wa (u_csn cp_ent)))	52	7
1299	(((cp_ent oa_ent)(u_isn u_hdip))((u_isn oa_wa)u_csn))	52	7
1300	((((oa_wa u_csn)p_csn)(p_isn ca_ent))(ca_wa p_re))	27	7
1301	(((oa_wa u_csn)(cp_ent ca_wa))((p_re ca_wa)p_csn))	28	7
1302	(((oa_wa u_csn)(p_isn ca_ent))((ca_wa p_re)ca_wa))	30	7
1303	(((oa_wa u_csn)(u_isn cp_ent))((ca_ent ca_wa)p_re))	28	7
1304	(((oa_wa u_csn)(u_isn cp_ent))((ca_wa p_re)ca_wa))	25	7
1305	((((oa_wa u_hdip)oa_wa)(u_csn cp_ent))(ca_ent p_isn))	30	7
1306	((((oa_wa u_hdip)oa_wa)(u_csn cp_ent))(p_csn ca_ent))	23	7
1307	((((oa_wa u_hdip)oa_wa)(u_csn cp_ent))(ca_ent ca_wa))	32	7
1308	((((oa_wa u_hdip)oa_wa)(u_csn cp_ent))(ca_wa ca_wa))	26	7
1309	((((oa_wa u_hdip)oa_wa)(u_csn cp_ent))(p_isn ca_wa))	26	7
1310	((((oa_wa u_hdip)oa_wa)(u_isn u_hdip))(u_csn cp_ent))	23	7
1311	((oa_wa u_hdip)((oa_wa u_csn)((cp_ent oa_ent)oa_wa)))	43	7
1312	(((oa_wa u_hdip)(oa_wa u_csn))((cp_ent oa_ent)oa_wa))	45	7
1313	(((oa_wa u_hdip)(u_isn oa_wa))(u_csn (cp_ent ca_ent)))	26	7
1314	(((oa_wa u_hdip)(u_isn oa_wa))(u_csn (ca_ent ca_wa)))	26	7
1315	(((oa_wa u_hdip)(u_isn oa_wa))(u_csn (cp_ent ca_wa)))	23	7
1316	(((oa_wa u_hdip)(u_isn oa_wa))((u_csn cp_ent)p_csn))	33	7
1317	(((p_csn ca_wa)(p_isn p_re))(ca_wa (p_csn cp_ent)))	39	7
1318	(((p_csn oa_ent)(oa_wa u_hdip))((u_csn cp_ent)p_csn))	32	7
1319	(((p_csn oa_ent)(oa_wa u_hdip))((u_isn oa_wa)u_csn))	28	7
1320	(((p_csn oa_ent)(u_hdip oa_wa))((u_csn cp_ent)p_csn))	29	7
1321	(((p_csn oa_ent)(u_isn oa_wa))((u_isn oa_wa)u_csn))	24	7
1322	(((p_csn oa_ent)(u_isn u_hdip))((u_isn oa_wa)u_csn))	30	7
1323	(((p_csn oa_ent)(u_isn u_hdip))((u_isn oa_wa)u_hdip))	44	7
1324	((((p_isn ca_ent)(ca_wa p_re))(ca_wa p_csn))oa_ent)	29	7
1325	(((p_isn ca_ent)(ca_wa p_re))(p_csn (cp_ent oa_ent)))	26	7
1326	(((p_isn ca_ent)(ca_wa p_re))(p_csn (oa_ent u_isn)))	36	7
1327	(((p_isn ca_ent)(ca_wa p_re))(p_csn (p_isn oa_ent)))	28	7
1328	(((p_isn ca_ent)(ca_wa p_re))((p_csn cp_ent)oa_ent))	30	7
1329	(((p_isn ca_ent)(ca_wa p_re))((p_csn cp_ent)p_csn))	32	7
1330	((((p_isn ca_wa)p_csn)(p_isn oa_ent))(u_isn u_hdip))	22	7
1331	(((p_isn ca_wa)(p_csn cp_ent))(oa_ent (oa_wa u_hdip)))	29	7
1332	(((p_isn ca_wa)(p_csn cp_ent))(oa_ent (u_isn u_hdip)))	32	7
1333	(((p_isn ca_wa)(p_csn cp_ent))((oa_ent u_isn)u_hdip))	33	7
1334	((((p_isn oa_ent)oa_wa)(u_isn u_hdip))(oa_wa u_csn))	30	7
1335	(((p_isn oa_ent)(oa_wa u_hdip))(oa_wa (u_csn cp_ent)))	39	7
1336	(((p_isn oa_ent)(oa_wa u_hdip))((u_csn cp_ent)p_csn))	35	7
1337	(((p_isn oa_ent)(oa_wa u_hdip))((u_isn oa_wa)u_csn))	37	7
1338	(((p_isn oa_ent)(u_isn u_hdip))((u_isn oa_wa)u_csn))	42	7
1339	(((p_isn p_re)(p_csn cp_ent))(oa_ent (oa_wa u_hdip)))	28	7
1340	(((p_isn p_re)(p_csn cp_ent))(oa_ent (u_isn u_hdip)))	31	7
1341	(((p_isn p_re)(p_csn cp_ent))(p_csn (oa_ent u_isn)))	22	7
1342	(((p_isn p_re)(p_csn cp_ent))((oa_ent u_isn)u_hdip))	32	7
1343	(((p_isn p_re)(p_csn cp_ent))((p_csn oa_ent)oa_wa))	20	7
1344	((((p_re ca_wa)oa_ent)(oa_wa u_hdip))(u_isn oa_wa))	25	7
1345	((((p_re ca_wa)p_csn)(p_isn oa_ent))(u_isn u_hdip))	30	7
1346	(((p_re ca_wa)(p_csn cp_ent))(oa_ent (oa_wa u_hdip)))	29	7
1347	(((p_re ca_wa)(p_csn cp_ent))(oa_ent (u_isn u_hdip)))	33	7
1348	(((p_re ca_wa)(p_csn cp_ent))(p_csn (oa_ent u_isn)))	22	7
1349	(((p_re ca_wa)(p_csn cp_ent))((oa_ent u_isn)u_hdip))	34	7
1350	(((p_re ca_wa)(p_csn cp_ent))((p_csn oa_ent)oa_wa))	20	7
1351	(((p_re ca_wa)(p_isn p_re))(p_csn (cp_ent oa_ent)))	19	7
1352	(((p_re ca_wa)(p_isn p_re))((p_csn cp_ent)oa_ent))	21	7
1353	((((u_csn cp_ent)p_csn)(p_isn ca_ent))(ca_wa p_re))	30	7
1354	(((u_csn cp_ent)(ca_ent ca_wa))((p_isn ca_wa)p_csn))	28	7
1355	(((u_csn cp_ent)(ca_ent ca_wa))((p_re ca_wa)p_csn))	37	7
1356	(((u_csn cp_ent)(ca_wa ca_wa))((cp_ent oa_ent)u_hdip))	29	7
1357	(((u_csn cp_ent)(ca_wa p_re))((cp_ent oa_ent)u_hdip))	33	7
1358	(((u_csn cp_ent)(ca_wa p_re))((p_csn oa_ent)u_hdip))	28	7
1359	(((u_csn cp_ent)(oa_ent oa_wa))(u_hdip (oa_wa u_csn)))	31	7
1360	(((u_csn cp_ent)(oa_ent oa_wa))((u_hdip oa_wa)u_csn))	40	7
1361	(((u_csn cp_ent)(oa_ent oa_wa))((u_isn oa_wa)u_csn))	37	7
1362	(((u_csn cp_ent)(p_isn ca_ent))((ca_wa p_re)ca_wa))	37	7
1363	(((u_csn cp_ent)(p_isn ca_ent))((p_isn ca_wa)p_csn))	23	7
1364	(((u_csn cp_ent)(p_isn ca_ent))((p_re ca_wa)oa_ent))	23	7
1365	(((u_csn cp_ent)(p_isn ca_ent))((p_re ca_wa)p_csn))	30	7
1366	(((u_csn cp_ent)(p_isn ca_wa))((p_re ca_wa)p_csn))	28	7
1367	((((u_hdip oa_wa)u_csn)(cp_ent ca_ent))(ca_wa p_re))	31	7
1368	((((u_hdip oa_wa)u_csn)(cp_ent oa_ent))(u_isn u_hdip))	47	7
1369	(((u_hdip u_csn)(cp_ent ca_ent))((ca_wa p_re)ca_wa))	34	7
1370	(((u_hdip u_csn)(ca_ent ca_wa))((p_isn ca_wa)p_csn))	22	7
1371	(((u_hdip u_csn)(cp_ent ca_ent))((ca_wa p_re)cp_ent))	31	7
1372	(((u_hdip u_csn)((oa_ent u_isn)u_hdip))(u_isn u_hdip))	27	7
1373	(((u_hdip u_csn)(u_isn cp_ent))(oa_ent (oa_wa u_hdip)))	33	7
1374	(((u_hdip u_csn)(u_isn cp_ent))((oa_ent u_isn)u_hdip))	35	7
1375	(((u_isn cp_ent)(ca_ent ca_wa))((p_re ca_wa)p_csn))	26	7
1376	(((u_isn cp_ent)(p_isn ca_ent))((ca_wa p_re)ca_wa))	27	7
1377	((((u_isn oa_wa)u_hdip)(u_csn cp_ent))(ca_ent ca_wa))	26	7
1378	((((u_isn oa_wa)u_hdip)(u_csn cp_ent))(ca_ent p_isn))	26	7
1379	((((u_isn oa_wa)u_hdip)(u_csn cp_ent))(p_csn ca_ent))	25	7
1380	((((u_isn oa_wa)u_hdip)(u_isn u_hdip))(oa_wa u_csn))	40	7
1381	(((u_isn u_csn)(cp_ent ca_ent))((ca_wa p_re)ca_wa))	32	7
1382	(((u_isn u_csn)(cp_ent ca_ent))((ca_wa p_re)cp_ent))	29	7
1383	(((u_isn u_hdip)(oa_wa (u_csn cp_ent)))(ca_ent p_isn))	27	7
1384	(((u_isn u_hdip)(oa_wa (u_csn cp_ent)))(ca_ent ca_wa))	27	7
1385	(((u_isn u_hdip)(oa_wa (u_csn cp_ent)))(p_csn ca_ent))	22	7
1386	(((u_isn u_hdip)(oa_wa u_csn))(oa_ent (oa_wa u_hdip)))	21	7
1387	(((u_isn u_hdip)(oa_wa u_csn))(oa_ent (u_isn u_hdip)))	21	7
1388	(((u_isn u_hdip)(oa_wa u_csn))((cp_ent ca_wa)cp_ent))	22	7
1389	(((u_isn u_hdip)(u_csn cp_ent))(oa_ent (u_isn u_hdip)))	29	7
1390	(((u_isn u_hdip)(u_csn cp_ent))((oa_ent u_isn)u_hdip))	34	7
1391	(oa_ent ((oa_wa u_hdip)((u_isn u_hdip)(oa_wa u_csn))))	35	7
1392	(p_csn ((ca_ent ca_wa)((p_re ca_wa)(cp_ent oa_ent))))	33	7
1393	(p_csn ((ca_ent p_isn)((p_re ca_wa)(cp_ent oa_ent))))	33	7
1394	(p_csn ((p_isn ca_ent)((ca_wa ca_wa)(cp_ent oa_ent))))	26	7
1395	(p_csn ((p_isn ca_ent)((p_re ca_wa)(cp_ent oa_ent))))	29	7
1396	(p_csn ((p_isn ca_wa)((p_re ca_wa)(cp_ent oa_ent))))	28	7
1397	(p_csn ((p_isn oa_ent)((oa_wa u_hdip)(oa_wa u_csn))))	24	7
1398	(p_csn (((ca_ent ca_wa)cp_ent)(oa_ent (u_isn u_hdip))))	29	7
1399	(p_csn (((ca_ent ca_wa)cp_ent)((oa_ent u_isn)u_hdip)))	30	7
1400	(p_csn (((ca_ent ca_wa)cp_ent)((p_isn oa_ent)u_hdip)))	22	7
1401	(p_csn (((p_isn ca_ent)(ca_wa p_re))(cp_ent oa_ent)))	30	7
1402	(p_csn (((p_isn oa_ent)oa_wa)(u_hdip (oa_wa u_csn))))	19	7
1403	(p_csn (((p_isn oa_ent)oa_wa)((u_hdip oa_wa)u_csn)))	21	7
1404	(p_csn (((p_isn oa_ent)(oa_wa u_hdip))(oa_wa u_csn)))	25	7
1405	(p_csn (((p_isn oa_ent)(oa_wa u_hdip))(u_csn cp_ent)))	24	7
1406	(p_csn (((p_isn oa_ent)(u_isn u_hdip))(oa_wa u_csn)))	30	7
1407	((ca_ent ca_wa)((p_isn ca_wa)(p_csn (cp_ent oa_ent))))	31	7
1408	((ca_ent ca_wa)((p_isn ca_wa)((p_csn cp_ent)oa_ent)))	35	7
1409	((ca_ent ca_wa)((p_isn p_re)((p_csn cp_ent)p_csn)))	34	7
1410	((ca_ent ca_wa)((p_re ca_wa)(p_csn (cp_ent oa_ent))))	34	7
1411	((ca_ent ca_wa)((p_re ca_wa)(p_csn (oa_ent oa_wa))))	36	7
1412	((ca_ent ca_wa)((p_re ca_wa)(p_csn (oa_ent u_hdip))))	38	7
1413	((ca_ent ca_wa)((p_re ca_wa)(p_csn (oa_ent u_isn))))	47	7
1414	((ca_ent ca_wa)((p_re ca_wa)(p_csn (p_isn oa_ent))))	30	7
1415	((ca_ent ca_wa)((p_re ca_wa)((cp_ent oa_ent)oa_wa)))	42	7
1416	((ca_ent ca_wa)((p_re ca_wa)((cp_ent oa_ent)u_hdip)))	46	7
1417	((ca_ent ca_wa)((p_re ca_wa)((p_csn cp_ent)oa_ent)))	38	7
1418	((ca_ent ca_wa)((p_re ca_wa)((p_csn cp_ent)p_csn)))	38	7
1419	((ca_ent ca_wa)((p_re ca_wa)((p_csn oa_ent)oa_wa)))	35	7
1420	((ca_ent ca_wa)((p_re ca_wa)((p_csn oa_ent)u_hdip)))	40	7
1421	((ca_ent ca_wa)((p_re (ca_wa p_csn))(oa_ent u_isn)))	38	7
1422	((ca_ent ca_wa)(((p_isn ca_wa)p_csn)(oa_ent oa_wa)))	24	7
1423	((ca_ent ca_wa)(((p_isn ca_wa)p_csn)(oa_ent u_isn)))	31	7
1424	((ca_ent ca_wa)(((p_isn ca_wa)p_csn)(p_isn oa_ent)))	22	7
1425	((ca_ent ca_wa)(((p_re ca_wa)oa_ent)(oa_wa u_hdip)))	33	7
1426	((ca_ent ca_wa)(((p_re ca_wa)oa_ent)(u_hdip u_hdip)))	37	7
1427	((ca_ent ca_wa)(((p_re ca_wa)oa_ent)(u_isn u_hdip)))	37	7
1428	((ca_ent ca_wa)(((p_re ca_wa)p_csn)(oa_ent u_isn)))	48	7
1429	((ca_ent ca_wa)(((p_re ca_wa)p_csn)(cp_ent oa_ent)))	39	7
1430	((ca_ent ca_wa)(((p_re ca_wa)p_csn)(oa_ent oa_wa)))	38	7
1431	((ca_ent ca_wa)(((p_re ca_wa)p_csn)(oa_ent u_hdip)))	40	7
1432	((ca_ent ca_wa)(((p_re ca_wa)p_csn)(p_isn oa_ent)))	31	7
1433	((ca_ent p_isn)((p_re ca_wa)(p_csn (cp_ent oa_ent))))	32	7
1434	((ca_ent p_isn)((p_re ca_wa)(p_csn (oa_ent oa_wa))))	35	7
1435	((ca_ent p_isn)((p_re ca_wa)(p_csn (oa_ent u_hdip))))	37	7
1436	((ca_ent p_isn)((p_re ca_wa)(p_csn (oa_ent u_isn))))	47	7
1437	((ca_ent p_isn)((p_re ca_wa)(p_csn (p_isn oa_ent))))	28	7
1438	((ca_ent p_isn)((p_re ca_wa)((cp_ent oa_ent)oa_wa)))	42	7
1439	((ca_ent p_isn)((p_re ca_wa)((p_csn oa_ent)oa_wa)))	33	7
1440	((ca_ent p_isn)((p_re ca_wa)((p_csn oa_ent)u_hdip)))	39	7
1441	((ca_ent p_isn)((ca_wa (p_csn cp_ent))(oa_ent u_isn)))	36	7
1442	((ca_ent p_isn)((ca_wa (p_csn cp_ent))(oa_ent oa_wa)))	28	7
1443	((ca_ent p_isn)((ca_wa (p_csn cp_ent))(oa_ent u_hdip)))	29	7
1444	((ca_ent p_isn)((p_re (ca_wa p_csn))(oa_ent u_isn)))	37	7
1445	((ca_ent p_isn)(((p_re ca_wa)oa_ent)(u_hdip u_hdip)))	36	7
1446	((ca_ent p_isn)(((p_re ca_wa)oa_ent)(u_isn u_hdip)))	36	7
1447	((ca_ent p_isn)(((p_re ca_wa)p_csn)(oa_ent u_isn)))	48	7
1448	((ca_ent p_isn)(((p_re ca_wa)p_csn)(cp_ent oa_ent)))	38	7
1449	((ca_ent p_isn)(((p_re ca_wa)p_csn)(oa_ent oa_wa)))	37	7
1450	((ca_ent p_isn)(((p_re ca_wa)p_csn)(oa_ent u_hdip)))	39	7
1451	((ca_ent p_isn)(((p_re ca_wa)p_csn)(p_isn oa_ent)))	29	7
1452	((ca_ent p_re)((ca_wa (p_csn cp_ent))(oa_ent u_isn)))	36	7
1453	(((ca_ent ca_wa)p_re)((ca_wa (p_csn cp_ent))p_csn))	37	7
1454	((ca_wa ca_wa)((p_csn cp_ent)(oa_ent (oa_wa u_hdip))))	29	7
1455	((ca_wa ca_wa)((p_csn cp_ent)(oa_ent (u_isn u_hdip))))	32	7
1456	((ca_wa ca_wa)((p_csn cp_ent)(p_csn (oa_ent u_isn))))	23	7
1457	((ca_wa ca_wa)((p_csn cp_ent)((oa_ent u_isn)u_hdip)))	33	7
1458	((ca_wa ca_wa)((p_csn (oa_ent u_isn))(u_hdip u_hdip)))	35	7
1459	((ca_wa ca_wa)(((p_csn cp_ent)oa_ent)(u_hdip u_hdip)))	33	7
1460	((ca_wa ca_wa)(((p_csn cp_ent)oa_ent)(u_isn u_hdip)))	33	7
1461	((ca_wa p_re)(p_csn ((p_isn oa_ent)(u_isn u_hdip))))	25	7
1462	((ca_wa p_re)((p_csn cp_ent)(oa_ent (u_isn u_hdip))))	32	7
1463	((ca_wa p_re)((p_csn cp_ent)((oa_ent u_isn)u_hdip)))	33	7
1464	((ca_wa p_re)((p_csn (oa_ent u_isn))(u_hdip u_hdip)))	34	7
1465	((ca_wa p_re)(((ca_wa p_csn)oa_ent)(u_hdip u_hdip)))	31	7
1466	((ca_wa p_re)(((ca_wa p_csn)oa_ent)(u_isn u_hdip)))	32	7
1467	((ca_wa p_re)(((p_csn cp_ent)oa_ent)(u_hdip u_hdip)))	33	7
1468	((cp_ent ca_ent)((ca_wa ca_wa)(p_csn (oa_ent u_isn))))	33	7
1469	((cp_ent ca_ent)((ca_wa p_re)(p_csn (oa_ent u_isn))))	40	7
1470	((cp_ent ca_ent)((ca_wa p_re)((ca_wa p_csn)oa_ent)))	33	7
1471	((cp_ent ca_ent)((p_isn ca_wa)(p_csn (cp_ent oa_ent))))	29	7
1472	((cp_ent ca_ent)((p_isn ca_wa)((p_csn cp_ent)oa_ent)))	33	7
1473	((cp_ent ca_ent)((p_isn p_re)((ca_wa p_csn)oa_ent)))	32	7
1474	((cp_ent ca_ent)((p_re ca_wa)(p_csn (oa_ent u_hdip))))	35	7
1475	((cp_ent ca_ent)((p_re ca_wa)(p_csn (oa_ent u_isn))))	44	7
1476	((cp_ent ca_ent)((p_re ca_wa)((cp_ent oa_ent)oa_wa)))	39	7
1477	((cp_ent ca_ent)((p_re ca_wa)((p_csn cp_ent)oa_ent)))	36	7
1478	((cp_ent ca_ent)((p_re ca_wa)((p_csn oa_ent)oa_wa)))	29	7
1479	((cp_ent ca_ent)((p_re ca_wa)((p_csn oa_ent)u_hdip)))	37	7
1480	((cp_ent ca_ent)((p_re (ca_wa p_csn))(oa_ent u_isn)))	36	7
1481	((cp_ent ca_ent)(((ca_wa p_re)ca_wa)(cp_ent oa_ent)))	52	7
1482	((cp_ent ca_ent)(((ca_wa p_re)ca_wa)(oa_ent u_isn)))	40	7
1483	((cp_ent ca_ent)(((ca_wa p_re)ca_wa)(p_csn oa_ent)))	45	7
1484	((cp_ent ca_ent)(((ca_wa p_re)ca_wa)(p_isn cp_ent)))	58	7
1485	((cp_ent ca_ent)(((ca_wa p_re)cp_ent)(oa_ent u_isn)))	47	7
1486	((cp_ent ca_ent)(((ca_wa p_re)cp_ent)(oa_ent oa_wa)))	41	7
1487	((cp_ent ca_ent)(((ca_wa p_re)cp_ent)(oa_ent u_hdip)))	42	7
1488	((cp_ent ca_ent)(((ca_wa p_re)(p_csn cp_ent))oa_ent))	36	7
1489	((cp_ent ca_ent)(((p_isn ca_wa)p_csn)(oa_ent oa_wa)))	22	7
1490	((cp_ent ca_ent)(((p_isn ca_wa)p_csn)(oa_ent u_isn)))	29	7
1491	((cp_ent ca_ent)(((p_isn ca_wa)p_csn)(p_isn oa_ent)))	22	7
1492	((cp_ent ca_ent)(((p_isn p_re)(p_csn cp_ent))oa_ent))	35	7
1493	((cp_ent ca_ent)(((p_re ca_wa)oa_ent)(u_isn u_hdip)))	32	7
1494	((cp_ent ca_ent)(((p_re ca_wa)p_csn)(oa_ent u_isn)))	45	7
1495	((cp_ent ca_ent)(((p_re ca_wa)p_csn)(cp_ent oa_ent)))	37	7
1496	((cp_ent ca_ent)(((p_re ca_wa)p_csn)(oa_ent oa_wa)))	32	7
1497	((cp_ent ca_ent)(((p_re ca_wa)p_csn)(oa_ent u_hdip)))	37	7
1498	((cp_ent ca_ent)(((p_re ca_wa)p_csn)(p_isn oa_ent)))	29	7
1499	((cp_ent ca_wa)((p_isn p_re)((ca_wa p_csn)oa_ent)))	30	7
1500	((cp_ent ca_wa)((p_re ca_wa)(p_csn (oa_ent oa_wa))))	31	7
1501	((cp_ent ca_wa)((p_re ca_wa)(p_csn (oa_ent u_hdip))))	33	7
1502	((cp_ent ca_wa)((p_re ca_wa)(p_csn (oa_ent u_isn))))	42	7
1503	((cp_ent ca_wa)((p_re ca_wa)(p_csn (p_isn oa_ent))))	26	7
1504	((cp_ent ca_wa)((p_re ca_wa)((cp_ent oa_ent)oa_wa)))	36	7
1505	((cp_ent ca_wa)((p_re ca_wa)((cp_ent oa_ent)u_hdip)))	40	7
1506	((cp_ent ca_wa)((p_re ca_wa)((p_csn oa_ent)u_hdip)))	35	7
1507	((cp_ent ca_wa)((p_re (ca_wa p_csn))(oa_ent u_isn)))	34	7
1508	((cp_ent ca_wa)((p_re (ca_wa p_csn))(p_isn cp_ent)))	40	7
1509	((cp_ent ca_wa)(((p_isn ca_wa)p_csn)(oa_ent u_isn)))	27	7
1510	((cp_ent ca_wa)(((p_isn ca_wa)p_csn)(p_isn oa_ent)))	20	7
1511	((cp_ent ca_wa)(((p_re ca_wa)oa_ent)(u_hdip u_hdip)))	33	7
1512	((cp_ent ca_wa)(((p_re ca_wa)oa_ent)(u_isn u_hdip)))	32	7
1513	((cp_ent ca_wa)(((p_re ca_wa)p_csn)(oa_ent u_isn)))	43	7
1514	((cp_ent ca_wa)(((p_re ca_wa)p_csn)(oa_ent oa_wa)))	33	7
1515	((cp_ent ca_wa)(((p_re ca_wa)p_csn)(oa_ent u_hdip)))	35	7
1516	((cp_ent ca_wa)(((p_re ca_wa)p_csn)(p_isn oa_ent)))	29	7
1517	((cp_ent oa_ent)((u_hdip u_hdip)(oa_wa (u_csn cp_ent))))	44	7
1518	((cp_ent oa_ent)(((u_isn oa_wa)u_hdip)(oa_wa u_csn)))	28	7
1519	((oa_ent oa_wa)((u_hdip u_hdip)(oa_wa (u_csn cp_ent))))	44	7
1520	((oa_ent oa_wa)((u_isn u_hdip)(oa_wa (u_csn cp_ent))))	39	7
1521	((oa_ent oa_wa)((u_isn u_hdip)((u_isn oa_wa)u_csn)))	44	7
1522	((oa_ent u_hdip)((u_isn u_hdip)((u_isn oa_wa)u_csn)))	45	7
1523	((oa_wa oa_wa)((u_csn cp_ent)(p_csn (ca_ent ca_wa))))	20	7
1524	((oa_wa oa_wa)((u_csn cp_ent)(p_csn (p_isn ca_wa))))	18	7
1525	((oa_wa oa_wa)((u_csn (ca_ent ca_wa))(p_isn p_re)))	23	7
1526	((oa_wa oa_wa)((u_csn cp_ent)((p_isn oa_ent)oa_wa)))	35	7
1527	((oa_wa oa_wa)((u_csn (cp_ent ca_wa))(p_isn p_re)))	23	7
1528	((oa_wa oa_wa)((u_csn (cp_ent ca_wa))(p_re ca_wa)))	24	7
1529	((oa_wa oa_wa)((u_csn (p_isn ca_ent))(p_isn p_re)))	19	7
1530	((oa_wa oa_wa)(((u_csn cp_ent)p_csn)(ca_ent p_isn)))	23	7
1531	((oa_wa oa_wa)(((u_csn cp_ent)p_csn)(ca_ent ca_wa)))	23	7
1532	((oa_wa oa_wa)(((u_csn cp_ent)p_csn)(ca_wa ca_wa)))	19	7
1533	((oa_wa oa_wa)(((u_csn cp_ent)p_csn)(p_isn ca_ent)))	19	7
1534	((oa_wa oa_wa)(((u_csn cp_ent)p_csn)(p_isn ca_wa)))	18	7
1535	((oa_wa u_csn)(((u_isn cp_ent)ca_ent)(ca_wa ca_wa)))	29	7
1536	((oa_wa u_hdip)((u_csn cp_ent)(p_csn (ca_ent ca_wa))))	26	7
1537	((oa_wa u_hdip)((u_isn oa_wa)((u_csn cp_ent)p_csn)))	21	7
1538	((oa_wa u_hdip)((u_isn u_hdip)(oa_wa (u_csn cp_ent))))	36	7
1539	((oa_wa u_hdip)((u_isn u_hdip)((oa_wa u_csn)oa_ent)))	18	7
1540	((oa_wa u_hdip)((oa_wa (u_csn cp_ent))(oa_ent oa_wa)))	46	7
1541	((oa_wa u_hdip)(((u_csn cp_ent)p_csn)(ca_ent p_isn)))	27	7
1542	((oa_wa u_hdip)(((u_csn cp_ent)p_csn)(ca_ent ca_wa)))	27	7
1543	((oa_wa u_hdip)(((u_csn cp_ent)p_csn)(ca_wa ca_wa)))	24	7
1544	((oa_wa u_hdip)(((u_isn oa_wa)u_csn)(ca_ent ca_wa)))	19	7
1545	((oa_wa u_hdip)(((u_isn oa_wa)u_csn)(cp_ent ca_ent)))	19	7
1546	((oa_wa u_hdip)(((u_isn oa_wa)u_csn)(cp_ent ca_wa)))	18	7
1547	((oa_wa u_hdip)(((u_isn u_hdip)(oa_wa u_csn))oa_ent))	19	7
1548	((p_csn ca_ent)(((ca_wa p_re)ca_wa)(cp_ent oa_ent)))	42	7
1549	((p_csn ca_ent)(((ca_wa p_re)ca_wa)(oa_ent u_isn)))	32	7
1550	((p_csn ca_ent)(((ca_wa p_re)cp_ent)(oa_ent u_isn)))	37	7
1551	((p_csn ca_ent)(((ca_wa p_re)cp_ent)(oa_ent u_hdip)))	32	7
1552	((p_csn ca_wa)((p_re ca_wa)(p_csn (oa_ent u_isn))))	33	7
1553	((p_csn oa_ent)(((u_isn oa_wa)u_hdip)(oa_wa u_csn)))	20	7
1554	((p_isn ca_ent)((ca_wa ca_wa)(p_csn (oa_ent u_isn))))	30	7
1555	((p_isn ca_ent)((ca_wa p_re)(p_csn (oa_ent u_hdip))))	26	7
1556	((p_isn ca_ent)((ca_wa p_re)(p_csn (oa_ent u_isn))))	37	7
1557	((p_isn ca_ent)((ca_wa p_re)((p_csn oa_ent)oa_wa)))	27	7
1558	((p_isn ca_ent)((ca_wa p_re)((p_csn oa_ent)u_hdip)))	28	7
1559	((p_isn ca_ent)((p_isn ca_wa)(p_csn (cp_ent oa_ent))))	23	7
1560	((p_isn ca_ent)((p_isn ca_wa)((p_csn cp_ent)oa_ent)))	27	7
1561	((p_isn ca_ent)((p_isn p_re)((ca_wa p_csn)oa_ent)))	28	7
1562	((p_isn ca_ent)((p_re ca_wa)(p_csn (oa_ent oa_wa))))	24	7
1563	((p_isn ca_ent)((p_re ca_wa)((cp_ent oa_ent)u_hdip)))	39	7
1564	((p_isn ca_ent)((p_re ca_wa)((p_csn oa_ent)oa_wa)))	27	7
1565	((p_isn ca_ent)((p_re (ca_wa p_csn))(oa_ent u_isn)))	29	7
1566	((p_isn ca_ent)(((ca_wa p_re)ca_wa)(cp_ent oa_ent)))	45	7
1567	((p_isn ca_ent)(((ca_wa p_re)ca_wa)(oa_ent u_isn)))	36	7
1568	((p_isn ca_ent)(((ca_wa p_re)cp_ent)(oa_ent u_isn)))	43	7
1569	((p_isn ca_ent)(((ca_wa p_re)cp_ent)(oa_ent oa_wa)))	36	7
1570	((p_isn ca_ent)(((ca_wa p_re)cp_ent)(oa_ent u_hdip)))	37	7
1571	((p_isn ca_ent)(((ca_wa p_re)cp_ent)(p_isn oa_ent)))	24	7
1572	((p_isn ca_ent)(((p_isn ca_wa)p_csn)(oa_ent u_isn)))	25	7
1573	((p_isn ca_ent)(((p_isn ca_wa)p_csn)(p_isn oa_ent)))	21	7
1574	((p_isn ca_ent)(((p_re ca_wa)oa_ent)(u_hdip u_hdip)))	30	7
1575	((p_isn ca_ent)(((p_re ca_wa)oa_ent)(u_isn u_hdip)))	30	7
1576	((p_isn ca_ent)(((p_re ca_wa)p_csn)(oa_ent u_isn)))	38	7
1577	((p_isn ca_ent)(((p_re ca_wa)p_csn)(cp_ent oa_ent)))	29	7
1578	((p_isn ca_ent)(((p_re ca_wa)p_csn)(oa_ent oa_wa)))	26	7
1579	((p_isn ca_ent)(((p_re ca_wa)p_csn)(oa_ent u_hdip)))	32	7
1580	((p_isn ca_ent)(((p_re ca_wa)p_csn)(p_isn oa_ent)))	28	7
1581	((p_isn ca_wa)((p_csn cp_ent)(oa_ent (u_isn u_hdip))))	31	7
1582	((p_isn ca_wa)((p_csn cp_ent)((oa_ent u_isn)u_hdip)))	32	7
1583	((p_isn ca_wa)((p_isn p_re)((ca_wa p_csn)oa_ent)))	33	7
1584	((p_isn ca_wa)((p_re ca_wa)(p_csn (oa_ent oa_wa))))	34	7
1585	((p_isn ca_wa)((p_re ca_wa)(p_csn (oa_ent u_hdip))))	36	7
1586	((p_isn ca_wa)((p_re ca_wa)(p_csn (oa_ent u_isn))))	43	7
1587	((p_isn ca_wa)((p_re ca_wa)(p_csn (p_isn oa_ent))))	30	7
1588	((p_isn ca_wa)((p_re ca_wa)((cp_ent oa_ent)oa_wa)))	40	7
1589	((p_isn ca_wa)((p_re ca_wa)((cp_ent oa_ent)u_hdip)))	44	7
1590	((p_isn ca_wa)((p_re ca_wa)((p_csn oa_ent)oa_wa)))	33	7
1591	((p_isn ca_wa)((p_re p_re)((p_isn p_csn)oa_ent)))	28	7
1592	((p_isn ca_wa)((p_re (ca_wa p_csn))(oa_ent u_isn)))	36	7
1593	((p_isn ca_wa)(((p_csn cp_ent)oa_ent)(u_hdip u_hdip)))	33	7
1594	((p_isn ca_wa)(((p_isn p_re)(p_csn cp_ent))oa_ent))	36	7
1595	((p_isn ca_wa)(((p_re ca_wa)oa_ent)(oa_wa u_hdip)))	30	7
1596	((p_isn ca_wa)(((p_re ca_wa)oa_ent)(u_hdip u_hdip)))	35	7
1597	((p_isn ca_wa)(((p_re ca_wa)oa_ent)(u_isn u_hdip)))	35	7
1598	((p_isn ca_wa)(((p_re ca_wa)p_csn)(oa_ent u_isn)))	44	7
1599	((p_isn ca_wa)(((p_re ca_wa)p_csn)(oa_ent oa_wa)))	36	7
1600	((p_isn ca_wa)(((p_re ca_wa)p_csn)(oa_ent u_hdip)))	38	7
1601	((p_isn ca_wa)(((p_re ca_wa)p_csn)(p_isn oa_ent)))	31	7
1602	((p_isn oa_ent)((oa_wa oa_wa)((u_csn cp_ent)p_csn)))	27	7
1603	((p_isn p_re)((ca_wa (p_csn cp_ent))(oa_ent u_isn)))	38	7
1604	((p_isn p_re)((ca_wa (p_csn cp_ent))(oa_ent oa_wa)))	31	7
1605	((p_isn p_re)((ca_wa (p_csn cp_ent))(oa_ent u_hdip)))	32	7
1606	((p_isn p_re)(((ca_wa p_csn)oa_ent)(u_hdip u_hdip)))	30	7
1607	((p_isn p_re)(((ca_wa p_csn)oa_ent)(u_isn u_hdip)))	31	7
1608	((p_re ca_wa)(p_csn ((p_isn oa_ent)(u_isn u_hdip))))	26	7
1609	((p_re ca_wa)((p_csn cp_ent)(oa_ent (u_isn u_hdip))))	32	7
1610	((p_re ca_wa)((p_csn cp_ent)((oa_ent u_isn)u_hdip)))	33	7
1611	((p_re ca_wa)((p_csn (oa_ent u_hdip))(u_isn oa_wa)))	29	7
1612	((p_re ca_wa)((p_csn (oa_ent u_isn))(u_hdip u_hdip)))	38	7
1613	((p_re ca_wa)(((p_csn cp_ent)oa_ent)(u_hdip u_hdip)))	33	7
1614	((p_re p_re)((ca_wa (p_csn cp_ent))(oa_ent u_isn)))	30	7
1615	((u_csn (cp_ent ca_ent))(((ca_wa p_re)ca_wa)oa_ent))	28	7
1616	((u_csn (cp_ent ca_ent))(((ca_wa p_re)cp_ent)oa_ent))	34	7
1617	((u_csn cp_ent)(((ca_ent ca_wa)cp_ent)(oa_ent u_hdip)))	32	7
1618	((u_csn cp_ent)(((ca_ent ca_wa)p_re)(p_isn p_csn)))	29	7
1619	((u_hdip oa_wa)((u_csn cp_ent)(p_csn (ca_ent ca_wa))))	25	7
1620	((u_hdip oa_wa)((u_csn cp_ent)(p_csn (p_isn ca_wa))))	16	7
1621	((u_hdip oa_wa)((u_csn cp_ent)((oa_ent u_isn)u_hdip)))	50	7
1622	((u_hdip oa_wa)((u_csn cp_ent)((p_isn oa_ent)oa_wa)))	32	7
1623	((u_hdip oa_wa)((u_csn (oa_ent oa_wa))(u_isn u_hdip)))	26	7
1624	((u_hdip oa_wa)((u_csn (oa_ent u_hdip))(u_isn u_hdip)))	24	7

1625	((u_hdip oa_wa)(((u_csn cp_ent)p_csn)(ca_ent p_isn)))	28	7
1626	((u_hdip oa_wa)(((u_csn cp_ent)p_csn)(ca_ent ca_wa)))	28	7
1627	((u_hdip oa_wa)(((u_csn cp_ent)p_csn)(ca_wa ca_wa)))	24	7
1628	((u_hdip oa_wa)(((u_csn cp_ent)p_csn)(p_isn ca_wa)))	17	7
1629	((u_hdip u_csn)(((u_isn cp_ent)oa_ent)(oa_wa u_hdip)))	26	7
1630	((u_hdip u_hdip)((u_csn cp_ent)(oa_ent(u_isn u_hdip))))	43	7
1631	((u_hdip u_hdip)((u_csn cp_ent)(p_csn(ca_ent ca_wa))))	22	7
1632	((u_hdip u_hdip)((u_csn cp_ent)((oa_ent u_isn)u_hdip)))	44	7
1633	((u_hdip u_hdip)((u_isn oa_wa)((u_csn cp_ent)p_csn)))	35	7
1634	((u_hdip u_hdip)((oa_wa(u_csn cp_ent))(oa_ent oa_wa)))	47	7
1635	((u_hdip u_hdip)(((u_csn cp_ent)oa_ent)(u_isn u_hdip)))	41	7
1636	((u_hdip u_hdip)(((u_isn oa_wa)u_csn)(ca_ent ca_wa)))	28	7
1637	((u_hdip u_hdip)(((u_isn oa_wa)u_csn)(cp_ent ca_ent)))	28	7
1638	((u_hdip u_hdip)(((u_isn oa_wa)u_csn)(cp_ent ca_wa)))	22	7
1639	((u_isn oa_wa)((u_csn cp_ent)(p_csn(p_isn ca_wa))))	17	7
1640	((u_isn oa_wa)((u_csn cp_ent)((p_isn oa_ent)oa_wa)))	35	7
1641	((u_isn oa_wa)((u_hdip(u_csn cp_ent))(p_csn ca_ent)))	24	7
1642	((u_isn oa_wa)(((u_csn cp_ent)p_csn)(p_isn ca_wa)))	18	7
1643	((u_isn u_csn)(((u_isn cp_ent)ca_ent)(ca_wa ca_wa)))	26	7
1644	((u_isn u_hdip)((oa_wa u_csn)((cp_ent oa_ent)oa_wa)))	29	7
1645	((u_isn u_hdip)((u_isn oa_wa)((u_csn cp_ent)p_csn)))	37	7
1646	((u_isn u_hdip)((u_isn u_hdip)(oa_wa(u_csn cp_ent))))	46	7
1647	((u_isn u_hdip)((u_isn u_hdip)((oa_wa u_csn)oa_ent)))	21	7
1648	((u_isn u_hdip)((u_isn u_hdip)((u_isn oa_wa)u_csn)))	52	7
1649	((u_isn u_hdip)(((u_isn oa_wa)u_csn)(ca_ent ca_wa)))	27	7
1650	((u_isn u_hdip)(((u_isn oa_wa)u_csn)(cp_ent ca_ent)))	27	7
1651	((u_isn u_hdip)(((u_isn oa_wa)u_csn)(cp_ent ca_wa)))	22	7
1652	((u_isn u_hdip)(((u_isn u_hdip)(oa_wa u_csn))oa_ent))	21	7
1653	((oa_ent(oa_wa u_hdip))((oa_wa(u_csn cp_ent))oa_ent))	38	7
1654	((p_csn(ca_wa p_re))((ca_wa(p_csn cp_ent))oa_ent))	30	7
1655	((p_csn(p_isn ca_ent))(((ca_wa p_re)ca_wa)oa_ent))	22	7
1656	((p_csn(p_isn ca_ent))(((ca_wa p_re)cp_ent)oa_ent))	31	7
1657	((u_csn(p_isn ca_ent))(((ca_wa p_re)ca_wa)oa_ent))	24	7
1658	((u_csn(p_isn ca_ent))(((ca_wa p_re)cp_ent)oa_ent))	28	7
1659	((u_hdip(u_isn oa_wa))(((u_csn cp_ent)p_csn)ca_ent))	28	7
1660	(((ca_ent ca_wa)cp_ent)(p_csn((oa_ent oa_wa)u_hdip)))	26	7
1661	(((ca_ent ca_wa)cp_ent)((p_csn(oa_ent u_isn))oa_wa))	29	7
1662	(((ca_ent ca_wa)p_re)(p_csn(oa_ent(u_isn u_hdip))))	24	7
1663	(((ca_ent ca_wa)p_re)((p_csn(oa_ent u_isn))u_hdip))	25	7
1664	(((ca_wa p_re)ca_wa)(p_csn(oa_ent(oa_wa u_hdip))))	33	7
1665	(((ca_wa p_re)ca_wa)(p_csn(oa_ent(u_isn u_hdip))))	36	7
1666	(((ca_wa p_re)ca_wa)(p_csn((oa_ent oa_wa)u_hdip)))	34	7
1667	(((ca_wa p_re)ca_wa)(p_csn((p_isn oa_ent)u_hdip)))	27	7
1668	(((ca_wa p_re)ca_wa)((p_csn(oa_ent u_isn))u_hdip))	39	7
1669	(((ca_wa p_re)ca_wa)((p_csn(p_isn oa_ent))u_hdip))	30	7
1670	(((ca_wa p_re)cp_ent)((p_csn(oa_ent u_isn))oa_wa))	25	7
1671	(((cp_ent oa_ent)oa_wa)((u_hdip(u_isn oa_wa))u_csn))	39	7
1672	(((oa_ent u_isn)u_hdip)((oa_wa(u_csn cp_ent))oa_ent))	37	7
1673	(((oa_wa u_hdip)oa_wa)(((u_csn cp_ent)p_csn)ca_ent))	25	7
1674	(((u_isn cp_ent)ca_ent)(((ca_wa p_re)cp_ent)oa_ent))	23	7
1675	(((u_isn oa_wa)u_hdip)(((u_csn cp_ent)p_csn)ca_ent))	29	7
1676	((oa_ent((oa_wa u_hdip)(oa_wa u_csn)))(cp_ent oa_ent))	33	7
1677	((p_csn(oa_ent(u_isn u_hdip)))((u_isn oa_wa)u_csn))	29	7
1678	((p_csn(oa_ent(u_isn u_hdip)))((u_isn oa_wa)u_hdip))	41	7
1679	((p_csn((p_isn ca_ent)(ca_wa p_re)))(ca_wa p_csn))	54	7
1680	((p_csn((p_isn oa_ent)(u_isn u_hdip)))(u_isn oa_wa))	31	7
1681	((p_csn((p_isn oa_ent)(u_isn u_hdip)))(u_isn u_hdip))	44	7
1682	(((ca_ent ca_wa)((p_isn ca_wa)p_csn))(oa_ent oa_wa))	25	7
1683	(((ca_ent ca_wa)((p_isn ca_wa)p_csn))(oa_ent u_isn))	32	7
1684	(((ca_ent ca_wa)((p_isn ca_wa)p_csn))(p_isn oa_ent))	23	7
1685	(((ca_ent ca_wa)((p_isn ca_wa)(p_csn cp_ent)))oa_ent)	37	7
1686	(((ca_ent ca_wa)((p_re ca_wa)oa_ent))(u_isn u_hdip))	39	7
1687	(((ca_ent ca_wa)((p_re ca_wa)p_csn))(cp_ent oa_ent))	38	7
1688	(((ca_ent ca_wa)((p_re ca_wa)p_csn))(oa_ent oa_wa))	37	7
1689	(((ca_ent ca_wa)((p_re ca_wa)p_csn))(oa_ent u_hdip))	39	7
1690	(((ca_ent ca_wa)((p_re ca_wa)p_csn))(oa_ent u_isn))	47	7
1691	(((ca_ent ca_wa)((p_re ca_wa)p_csn))(p_isn oa_ent))	32	7
1692	(((ca_ent ca_wa)((p_re ca_wa)(p_csn cp_ent)))oa_ent)	39	7
1693	(((ca_wa p_re)(ca_wa(p_csn cp_ent)))(oa_ent oa_wa))	33	7
1694	(((ca_wa p_re)(ca_wa(p_csn cp_ent)))(oa_ent u_hdip))	34	7
1695	(((ca_ent p_isn)(ca_wa(p_csn cp_ent)))(oa_ent oa_wa))	32	7
1696	(((ca_ent p_isn)(ca_wa(p_csn cp_ent)))(oa_ent u_hdip))	33	7
1697	(((ca_ent p_isn)(ca_wa(p_csn cp_ent)))(p_csn oa_ent))	22	7
1698	(((ca_ent p_isn)(p_re(ca_wa p_csn)))(oa_ent u_isn))	37	7
1699	(((ca_ent p_isn)(p_re(ca_wa p_csn)))(p_isn p_csn))	54	7
1700	(((ca_ent p_isn)((p_re ca_wa)oa_ent))(oa_wa u_hdip))	32	7
1701	(((ca_ent p_isn)((p_re ca_wa)p_csn))(cp_ent oa_ent))	37	7
1702	(((ca_ent p_isn)((p_re ca_wa)p_csn))(oa_ent oa_wa))	36	7
1703	(((ca_ent p_isn)((p_re ca_wa)p_csn))(oa_ent u_isn))	46	7
1704	(((ca_ent p_isn)((p_re ca_wa)(p_csn cp_ent)))oa_ent)	38	7
1705	(((ca_wa ca_wa)(p_csn(oa_ent u_isn)))(u_hdip u_hdip))	33	7
1706	(((ca_wa(p_csn cp_ent))oa_ent)((u_isn oa_wa)u_hdip))	28	7
1707	(((ca_wa(p_csn cp_ent))(oa_ent u_isn))(oa_wa u_hdip))	30	7
1708	(((ca_wa(p_csn cp_ent))(oa_ent u_isn))(u_hdip u_hdip))	33	7
1709	(((ca_wa(p_csn cp_ent))(oa_ent oa_wa))(u_isn u_hdip))	29	7
1710	(((ca_wa(p_csn cp_ent))(oa_ent u_hdip))(u_isn u_hdip))	31	7
1711	(((ca_wa p_re)(p_csn(oa_ent u_hdip)))(u_isn u_hdip))	29	7
1712	(((ca_wa p_re)(p_csn(oa_ent u_isn)))(oa_wa oa_wa))	28	7
1713	(((ca_wa p_re)(p_csn(oa_ent u_isn)))(oa_wa u_hdip))	31	7
1714	(((ca_wa p_re)(p_csn(oa_ent u_isn)))(u_hdip u_hdip))	35	7
1715	(((p_re(ca_wa p_csn))(cp_ent oa_ent))(u_isn u_hdip))	35	7
1716	(((p_re(ca_wa p_csn))(oa_ent u_hdip))(u_isn u_hdip))	30	7
1717	((((p_isn ca_wa)p_csn)(oa_ent u_hdip))(u_isn u_hdip))	30	7
1718	(((ca_wa p_re)((ca_wa p_csn)(p_isn cp_ent)))oa_ent)	23	7
1719	(((p_re(ca_wa p_csn))(p_isn cp_ent))(oa_ent u_isn))	23	7
1720	(((ca_wa p_re)((p_csn cp_ent)oa_ent))(oa_wa u_hdip))	30	7
1721	((((ca_ent ca_wa)(p_csn cp_ent))oa_ent)(oa_wa u_hdip))	30	7
1722	(((ca_wa p_re)((p_csn oa_ent)oa_wa))(u_hdip u_hdip))	23	7
1723	(((ca_wa p_re)((p_csn oa_ent)oa_wa))(u_isn oa_wa))	24	7
1724	(((ca_wa p_re)((p_csn oa_ent)oa_wa))(u_isn u_hdip))	28	7
1725	(((ca_wa p_re)((p_csn oa_ent)u_hdip))(oa_wa u_hdip))	26	7
1726	(((ca_wa p_re)((p_csn oa_ent)u_hdip))(u_isn u_hdip))	31	7
1727	(((cp_ent ca_ent)((ca_wa ca_wa)(p_isn cp_ent)))oa_ent)	25	7
1728	(((cp_ent ca_ent)((ca_wa p_re)cp_ent))(oa_ent oa_wa))	42	7
1729	(((cp_ent ca_ent)((ca_wa p_re)cp_ent))(oa_ent u_hdip))	43	7
1730	(((cp_ent ca_ent)((ca_wa p_re)cp_ent))(oa_ent u_isn))	48	7
1731	(((cp_ent ca_ent)((ca_wa p_re)cp_ent))(p_isn oa_ent))	29	7
1732	(((cp_ent ca_ent)((ca_wa p_re)(p_csn cp_ent)))oa_ent)	37	7
1733	(((cp_ent ca_ent)((ca_wa p_re)(p_isn cp_ent)))oa_ent)	27	7
1734	(((cp_ent ca_ent)((p_re ca_wa)oa_ent))(oa_wa u_hdip))	24	7
1735	(((cp_ent ca_ent)((p_re ca_wa)oa_ent))(u_hdip u_hdip))	34	7
1736	(((cp_ent ca_ent)((p_re ca_wa)oa_ent))(u_isn u_hdip))	34	7
1737	(((cp_ent ca_wa)((p_re ca_wa)oa_ent))(oa_wa u_hdip))	22	7
1738	(((cp_ent ca_wa)((p_re ca_wa)(p_csn cp_ent)))oa_ent)	35	7
1739	(((oa_ent oa_wa)(u_hdip(oa_wa u_csn)))(cp_ent oa_ent))	18	7
1740	(((oa_ent oa_wa)(u_hdip(oa_wa u_csn)))(u_isn cp_ent))	30	7
1741	(((oa_ent oa_wa)((u_hdip oa_wa)u_csn))(cp_ent oa_ent))	20	7
1742	(((oa_ent oa_wa)((u_hdip oa_wa)u_csn))(u_isn cp_ent))	33	7
1743	(((oa_ent oa_wa)((u_isn oa_wa)u_csn))(cp_ent oa_ent))	21	7
1744	(((oa_ent oa_wa)((u_isn oa_wa)u_csn))(oa_ent u_hdip))	19	7
1745	(((oa_ent oa_wa)((u_isn oa_wa)u_csn))(u_isn cp_ent))	33	7
1746	(((oa_ent u_hdip)((u_isn oa_wa)u_csn))(u_isn cp_ent))	32	7
1747	(((oa_wa oa_wa)(u_csn(cp_ent ca_ent)))(ca_wa ca_wa))	24	7
1748	(((oa_wa oa_wa)(u_csn(cp_ent ca_ent)))(p_isn p_re))	23	7
1749	(((oa_wa oa_wa)(u_csn(cp_ent ca_wa)))(p_re ca_wa))	23	7
1750	(((oa_wa oa_wa)(u_csn(cp_ent ca_wa)))(p_re p_csn))	20	7
1751	(((oa_wa oa_wa)((u_csn cp_ent)p_csn))(ca_wa ca_wa))	18	7
1752	(((oa_wa oa_wa)((u_csn cp_ent)p_csn))(p_isn ca_wa))	17	7
1753	(((oa_wa u_csn)(p_csn(p_isn ca_ent)))(ca_wa ca_wa))	23	7
1754	(((oa_wa u_csn)((cp_ent ca_wa)cp_ent))(oa_ent oa_wa))	25	7
1755	(((oa_wa u_csn)((cp_ent ca_wa)cp_ent))(oa_ent u_hdip))	26	7
1756	(((oa_wa u_csn)((cp_ent ca_wa)cp_ent))(oa_ent u_isn))	26	7
1757	(((oa_wa u_hdip)(oa_wa(u_csn cp_ent)))(ca_ent p_isn))	26	7
1758	(((oa_wa u_hdip)(oa_wa(u_csn cp_ent)))(ca_ent ca_wa))	26	7
1759	(((oa_wa u_hdip)(oa_wa(u_csn cp_ent)))(oa_wa u_hdip))	23	7
1760	(((oa_wa u_hdip)((u_isn u_hdip)(oa_wa u_csn)))oa_ent)	20	7
1761	(((p_csn ca_ent)((p_re ca_wa)p_csn))(cp_ent oa_ent))	28	7
1762	(((p_csn ca_wa)((p_re ca_wa)p_csn))(oa_ent u_isn))	31	7
1763	(((p_csn oa_ent)(u_hdip(u_isn oa_wa)))(u_isn u_hdip))	32	7
1764	(((p_csn oa_ent)((u_isn oa_wa)u_hdip))(oa_wa u_csn))	32	7
1765	(((p_csn oa_ent)((u_isn oa_wa)u_hdip))(u_csn cp_ent))	31	7
1766	(((p_csn oa_ent)((u_isn oa_wa)u_hdip))(u_isn oa_wa))	35	7
1767	(((p_csn oa_ent)((u_isn oa_wa)u_hdip))(u_isn u_csn))	25	7
1768	(((p_isn ca_ent)((ca_wa p_re)(p_csn cp_ent)))oa_ent)	30	7
1769	(((p_isn ca_ent)((p_isn ca_wa)(p_csn cp_ent)))oa_ent)	26	7
1770	(((p_isn ca_ent)((p_re ca_wa)oa_ent))(u_isn u_hdip))	26	7
1771	(((p_isn ca_ent)((p_re ca_wa)p_csn))(cp_ent oa_ent))	30	7
1772	(((p_isn ca_ent)((p_re ca_wa)p_csn))(oa_ent oa_wa))	28	7
1773	(((p_isn ca_ent)((p_re ca_wa)p_csn))(oa_ent u_hdip))	33	7
1774	(((p_isn ca_ent)((p_re ca_wa)p_csn))(oa_ent u_isn))	39	7
1775	(((p_isn ca_ent)((p_re ca_wa)p_csn))(p_isn oa_ent))	29	7
1776	(((p_isn ca_wa)((p_csn cp_ent)oa_ent))(oa_wa u_hdip))	30	7
1777	(((p_isn ca_wa)((p_re ca_wa)oa_ent))(oa_wa u_hdip))	31	7
1778	(((p_isn p_re)(ca_wa(p_csn cp_ent)))(p_csn oa_ent))	22	7
1779	(((p_isn p_re)((ca_wa p_csn)(p_isn cp_ent)))oa_ent)	22	7
1780	(((p_re ca_wa)(p_csn(oa_ent oa_wa)))(u_hdip u_hdip))	29	7
1781	(((p_re ca_wa)(p_csn(oa_ent oa_wa)))(u_isn u_hdip))	33	7
1782	(((p_re ca_wa)(p_csn(oa_ent u_hdip)))(u_isn u_hdip))	35	7
1783	(((p_re ca_wa)(p_csn(oa_ent u_isn)))(oa_wa u_hdip))	35	7
1784	(((p_re ca_wa)(p_csn(oa_ent u_isn)))(u_hdip u_hdip))	39	7
1785	(((p_re ca_wa)(p_csn(p_isn oa_ent)))(u_hdip u_hdip))	27	7
1786	(((p_re ca_wa)(p_csn(p_isn oa_ent)))(u_isn u_hdip))	30	7
1787	(((p_re ca_wa)((cp_ent oa_ent)oa_wa))(u_hdip u_hdip))	36	7
1788	(((p_re ca_wa)((cp_ent oa_ent)oa_wa))(u_isn oa_wa))	37	7
1789	(((p_re ca_wa)((cp_ent oa_ent)oa_wa))(u_isn u_hdip))	38	7
1790	(((p_re ca_wa)((cp_ent oa_ent)u_hdip))(u_isn oa_wa))	39	7
1791	(((p_re ca_wa)((cp_ent oa_ent)u_hdip))(u_isn u_hdip))	41	7
1792	(((p_re ca_wa)((p_csn oa_ent)oa_wa))(u_hdip u_hdip))	31	7
1793	(((p_re ca_wa)((p_csn oa_ent)oa_wa))(u_isn u_hdip))	32	7
1794	(((p_re ca_wa)((p_csn oa_ent)u_hdip))(oa_wa u_hdip))	32	7
1795	(((p_re ca_wa)((p_csn oa_ent)u_hdip))(u_isn u_hdip))	37	7
1796	(((u_csn cp_ent)(oa_ent(u_isn u_hdip)))(u_isn u_hdip))	54	7
1797	(((u_csn cp_ent)(p_csn(p_isn ca_ent)))(ca_wa ca_wa))	26	7
1798	(((u_csn cp_ent)((ca_ent ca_wa)p_csn))(oa_ent u_hdip))	32	7
1799	(((u_csn cp_ent)((ca_ent ca_wa)p_re))(p_isn ca_wa))	30	7
1800	(((u_csn cp_ent)((ca_wa p_re)ca_wa))(cp_ent oa_ent))	35	7
1801	(((u_hdip oa_wa)(u_csn(cp_ent ca_ent)))(ca_wa ca_wa))	31	7
1802	(((u_hdip oa_wa)(u_csn(cp_ent ca_ent)))(ca_wa p_csn))	23	7
1803	(((u_hdip oa_wa)(u_csn(cp_ent ca_ent)))(ca_wa p_re))	31	7
1804	(((u_hdip oa_wa)(u_csn(cp_ent ca_ent)))(p_isn ca_wa))	23	7
1805	(((u_hdip oa_wa)(u_csn(oa_ent oa_wa)))(u_hdip u_hdip))	24	7
1806	(((u_hdip oa_wa)(u_csn(oa_ent u_hdip)))(u_isn u_hdip))	26	7
1807	(((u_hdip oa_wa)(u_csn(cp_ent oa_ent)))(oa_wa u_hdip))	29	7
1808	(((u_hdip oa_wa)(u_csn(cp_ent oa_ent)))(u_isn u_hdip))	26	7
1809	(((u_hdip u_csn)(oa_ent(oa_wa u_hdip)))(u_isn u_hdip))	27	7
1810	(((u_hdip u_hdip)(oa_wa(u_csn cp_ent)))(ca_ent p_isn))	30	7
1811	(((u_hdip u_hdip)(oa_wa(u_csn cp_ent)))(ca_ent ca_wa))	30	7
1812	(((u_hdip u_hdip)(oa_wa(u_csn cp_ent)))(oa_ent oa_wa))	44	7
1813	(((u_hdip u_hdip)(oa_wa(u_csn cp_ent)))(oa_ent u_hdip))	52	7
1814	(((u_hdip u_hdip)(oa_wa(u_csn cp_ent)))(oa_wa u_hdip))	23	7
1815	(((u_hdip u_hdip)(oa_wa(u_csn cp_ent)))(p_csn ca_ent))	24	7
1816	(((u_hdip u_hdip)(u_csn(oa_ent oa_wa)))(u_isn u_hdip))	23	7
1817	(((u_hdip u_hdip)(u_csn(cp_ent oa_ent)))(oa_wa u_hdip))	30	7
1818	(((u_hdip u_hdip)(u_csn(cp_ent oa_ent)))(u_isn u_hdip))	27	7
1819	(((u_hdip u_hdip)((oa_wa u_csn)oa_ent))(oa_wa u_hdip))	24	7
1820	(((u_hdip u_hdip)((oa_wa u_csn)oa_ent))(u_isn oa_wa))	21	7
1821	(((u_hdip u_hdip)((oa_wa u_csn)oa_ent))(u_isn u_hdip))	21	7
1822	(((u_isn cp_ent)((ca_wa p_re)ca_wa))(p_csn cp_ent))	20	7
1823	(((u_isn oa_wa)((u_csn cp_ent)p_csn))(ca_ent p_isn))	29	7
1824	(((u_isn oa_wa)((u_csn cp_ent)p_csn))(ca_ent ca_wa))	29	7
1825	(((u_isn oa_wa)((u_csn cp_ent)p_csn))(ca_wa ca_wa))	25	7
1826	(((u_isn oa_wa)((u_csn cp_ent)p_csn))(p_isn ca_ent))	20	7
1827	(((u_isn u_hdip)(oa_wa(u_csn cp_ent)))(oa_ent u_isn))	36	7
1828	(((u_isn u_hdip)(oa_wa(u_csn cp_ent)))(oa_wa u_hdip))	20	7
1829	(((u_isn u_hdip)((oa_wa u_csn)oa_ent))(oa_wa u_hdip))	19	7
1830	(((u_isn u_hdip)((u_isn oa_wa)u_csn))(ca_ent ca_wa))	28	7
1831	(((u_isn u_hdip)((u_isn oa_wa)u_csn))(cp_ent ca_ent))	28	7
1832	(((u_isn u_hdip)((u_isn u_hdip)(oa_wa u_csn)))oa_ent)	33	7
1833	(((oa_ent(oa_wa u_hdip))(oa_wa u_csn))(cp_ent oa_ent))	23	7
1834	(((oa_ent(oa_wa u_hdip))(oa_wa u_csn))(u_isn cp_ent))	40	7
1835	(((oa_ent(oa_wa u_hdip))(oa_wa(u_csn cp_ent)))oa_ent)	41	7
1836	(((oa_ent(oa_wa u_hdip))((u_isn oa_wa)u_csn))oa_ent)	21	7
1837	(((oa_ent(u_isn u_hdip))(oa_wa u_csn))(cp_ent oa_ent))	26	7
1838	(((oa_ent(u_isn u_hdip))(oa_wa u_csn))(u_isn cp_ent))	39	7
1839	(((oa_ent(u_isn u_hdip))((u_isn oa_wa)u_csn))oa_ent)	24	7
1840	(((oa_wa(u_csn cp_ent))(ca_wa p_re))(ca_wa p_csn))	27	7
1841	(((oa_wa(u_csn cp_ent))(p_csn ca_wa))(p_re ca_wa))	23	7
1842	(((oa_wa(u_csn cp_ent))(p_isn ca_ent))(ca_wa ca_wa))	31	7
1843	(((oa_wa(u_csn cp_ent))(p_isn ca_ent))(p_isn ca_wa))	23	7
1844	(((oa_wa(u_csn cp_ent))(p_isn ca_ent))(p_isn p_re))	30	7
1845	(((oa_wa(u_csn cp_ent))(p_isn ca_ent))(p_re ca_wa))	30	7
1846	(((oa_wa(u_csn cp_ent))(p_isn ca_wa))(p_isn p_re))	28	7
1847	(((oa_wa(u_csn cp_ent))(p_isn ca_wa))(p_re ca_wa))	29	7
1848	(((oa_wa(u_csn cp_ent))(p_isn p_re))(ca_wa p_csn))	24	7
1849	(((p_csn(ca_ent ca_wa))(p_isn p_re))(ca_wa p_csn))	71	7
1850	(((p_csn(ca_ent ca_wa))((p_re ca_wa)p_csn))oa_ent)	37	7
1851	(((p_csn(ca_wa p_re))(ca_wa p_csn))(cp_ent oa_ent))	28	7
1852	(((p_csn(ca_wa p_re))(ca_wa p_csn))(oa_ent u_hdip))	23	7
1853	(((p_csn(ca_wa p_re))(ca_wa p_csn))(oa_ent u_isn))	25	7
1854	(((p_csn(ca_wa p_re))(ca_wa p_csn))(p_isn oa_ent))	23	7
1855	(((p_csn(oa_ent u_hdip))(u_isn oa_wa))(u_csn cp_ent))	37	7
1856	(((p_csn(oa_ent u_hdip))(u_isn oa_wa))(u_isn u_hdip))	34	7
1857	(((p_csn(oa_ent u_isn))(u_hdip u_hdip))(oa_wa u_hdip))	29	7
1858	(((p_csn(oa_ent u_isn))(u_hdip u_hdip))(u_isn oa_wa))	43	7
1859	(((p_csn(oa_ent u_isn))(u_hdip u_hdip))(u_isn u_hdip))	42	7
1860	(((p_csn(p_isn ca_ent))(ca_wa p_re))(ca_wa p_csn))	56	7
1861	(((p_csn(p_isn ca_ent))(p_isn p_re))(ca_wa p_csn))	55	7
1862	(((p_csn(p_isn ca_wa))(p_isn p_re))(cp_ent oa_ent))	32	7
1863	(((p_csn(p_isn oa_ent))(oa_wa u_hdip))(oa_wa u_csn))	31	7
1864	(((p_re(ca_wa p_csn))(oa_ent u_isn))(u_hdip u_hdip))	31	7
1865	(((u_csn(ca_ent ca_wa))(p_isn ca_wa))(oa_ent u_isn))	34	7
1866	(((u_csn(ca_ent ca_wa))(p_re p_re))(p_isn p_csn))	27	7
1867	(((u_csn(oa_ent oa_wa))(u_isn u_hdip))(u_isn u_hdip))	29	7
1868	(((u_csn(p_isn ca_ent))(ca_wa p_re))(p_isn ca_wa))	26	7
1869	(((u_csn(u_isn cp_ent))(ca_ent p_isn))(ca_wa p_csn))	25	7
1870	(((u_csn(u_isn cp_ent))(ca_ent p_isn))(p_re ca_wa))	29	7
1871	(((u_csn(u_isn cp_ent))(ca_ent p_isn))(p_re p_csn))	25	7
1872	(((u_csn(u_isn cp_ent))ca_ent)((ca_wa p_re)ca_wa))	30	7
1873	(((u_csn(u_isn cp_ent))ca_ent)((ca_wa p_re)cp_ent))	28	7
1874	(((u_csn(u_isn cp_ent))ca_ent)((p_re ca_wa)p_csn))	25	7
1875	(((u_csn(u_isn cp_ent))(ca_ent ca_wa))(ca_wa p_csn))	26	7
1876	(((u_csn(u_isn cp_ent))(ca_ent ca_wa))(p_isn p_re))	30	7
1877	(((u_csn(u_isn cp_ent))(ca_ent ca_wa))(p_re ca_wa))	30	7
1878	(((u_csn(u_isn cp_ent))(ca_ent ca_wa))(p_re p_csn))	26	7
1879	(((u_csn(u_isn cp_ent))(ca_wa p_re))(ca_wa p_csn))	22	7
1880	(((u_csn(u_isn cp_ent))(p_isn ca_ent))(ca_wa ca_wa))	27	7
1881	(((u_csn(u_isn cp_ent))(p_isn ca_ent))(ca_wa p_csn))	22	7
1882	(((u_csn(u_isn cp_ent))(p_isn ca_ent))(p_isn p_re))	26	7
1883	(((u_csn(u_isn cp_ent))(p_isn ca_ent))(p_re ca_wa))	26	7
1884	(((u_csn(u_isn cp_ent))(p_isn ca_wa))(p_isn p_re))	20	7
1885	(((u_csn(u_isn cp_ent))(p_isn ca_wa))(p_re ca_wa))	20	7
1886	(((u_hdip(oa_wa u_csn))(cp_ent ca_ent))(ca_wa ca_wa))	29	7
1887	(((u_hdip(oa_wa u_csn))(ca_ent ca_wa))(ca_wa p_csn))	22	7
1888	(((u_hdip(oa_wa u_csn))(cp_ent ca_ent))(ca_wa p_re))	29	7
1889	(((u_hdip(oa_wa u_csn))(cp_ent ca_ent))(p_isn ca_wa))	21	7
1890	(((u_hdip(oa_wa u_csn))(ca_ent p_isn))(p_re ca_wa))	29	7
1891	(((u_hdip(oa_wa u_csn))(cp_ent oa_ent))(u_isn u_hdip))	38	7
1892	(((u_hdip(u_csn cp_ent))(ca_ent p_isn))(p_re ca_wa))	32	7
1893	(((u_hdip(u_csn cp_ent))ca_ent)((ca_wa p_re)ca_wa))	32	7
1894	(((u_hdip(u_csn cp_ent))(oa_wa u_hdip))(u_isn u_hdip))	25	7
1895	(((u_hdip(u_csn cp_ent))(p_csn ca_ent))(ca_wa ca_wa))	25	7
1896	(((u_hdip(u_csn cp_ent))(p_isn ca_ent))(ca_wa ca_wa))	27	7
1897	(((u_hdip(u_csn cp_ent))(p_isn ca_ent))(ca_wa p_csn))	20	7
1898	(((u_hdip(u_csn cp_ent))(p_isn ca_ent))(p_isn ca_wa))	21	7
1899	(((u_hdip(u_csn cp_ent))(p_isn ca_wa))(p_isn ca_wa))	19	7
1900	(((u_hdip(u_isn oa_wa))u_csn)(oa_ent(oa_wa u_hdip)))	27	7
1901	(((u_hdip(u_isn oa_wa))u_csn)(oa_ent(u_isn u_hdip)))	27	7
1902	(((u_hdip(u_isn oa_wa))u_csn)((cp_ent ca_wa)cp_ent))	26	7
1903	(((u_hdip(u_isn oa_wa))u_csn)((oa_ent u_isn)u_hdip))	29	7
1904	(((u_hdip(u_isn u_csn))(cp_ent ca_ent))(ca_wa ca_wa))	31	7
1905	(((u_hdip(u_isn u_csn))(ca_ent ca_wa))(ca_wa p_csn))	24	7
1906	(((u_hdip(u_isn u_csn))(cp_ent ca_ent))(p_isn ca_wa))	23	7
1907	(((u_hdip(u_isn u_csn))(ca_ent p_isn))(p_re ca_wa))	31	7
1908	(((u_hdip(u_isn u_csn))(cp_ent ca_wa))(ca_wa p_csn))	20	7
1909	(((u_hdip(u_isn u_csn))(cp_ent ca_wa))(p_isn ca_wa))	21	7
1910	(((u_hdip(u_isn u_csn))(u_isn cp_ent))(ca_ent p_isn))	29	7
1911	(((u_hdip(u_isn u_csn))(u_isn cp_ent))(ca_ent ca_wa))	30	7
1912	((((ca_ent ca_wa)cp_ent)oa_ent)(u_hdip(u_isn oa_wa)))	40	7
1913	((((ca_ent ca_wa)cp_ent)oa_ent)((u_hdip oa_wa)u_hdip))	29	7
1914	((((ca_ent ca_wa)cp_ent)oa_ent)((u_isn oa_wa)u_hdip))	39	7
1915	((((ca_ent ca_wa)cp_ent)(oa_ent oa_wa))(u_hdip u_hdip))	36	7
1916	((((ca_ent ca_wa)cp_ent)(oa_ent oa_wa))(u_isn oa_wa))	40	7
1917	((((ca_ent ca_wa)cp_ent)(oa_ent oa_wa))(u_isn u_hdip))	44	7
1918	((((ca_ent ca_wa)cp_ent)(oa_ent u_hdip))(u_isn oa_wa))	41	7
1919	((((ca_ent ca_wa)cp_ent)(oa_ent u_hdip))(u_isn u_hdip))	44	7
1920	((((ca_ent ca_wa)cp_ent)(oa_ent u_isn))(u_hdip u_hdip))	45	7
1921	((((ca_ent ca_wa)cp_ent)(p_csn oa_ent))(oa_wa u_hdip))	28	7
1922	((((ca_ent ca_wa)p_csn)oa_ent)((oa_wa u_hdip)oa_wa))	29	7
1923	((((ca_ent ca_wa)p_csn)(cp_ent oa_ent))(oa_wa u_hdip))	29	7
1924	((((ca_ent ca_wa)p_csn)(cp_ent oa_ent))(u_isn oa_wa))	30	7
1925	((((ca_ent ca_wa)p_csn)(cp_ent oa_ent))(u_isn u_hdip))	35	7
1926	((((ca_ent ca_wa)p_csn)(oa_ent oa_wa))(u_isn u_hdip))	32	7
1927	((((ca_ent ca_wa)p_csn)(oa_ent u_isn))(oa_wa u_hdip))	34	7
1928	((((ca_ent ca_wa)p_csn)(oa_ent u_isn))(u_hdip u_hdip))	35	7
1929	((((ca_ent ca_wa)p_csn)(p_isn oa_ent))(u_isn u_hdip))	30	7
1930	((((ca_ent ca_wa)p_re)(cp_ent oa_ent))(oa_wa u_hdip))	35	7
1931	((((ca_ent ca_wa)p_re)(cp_ent oa_ent))(u_hdip u_hdip))	37	7
1932	((((ca_ent ca_wa)p_re)(cp_ent oa_ent))(u_isn u_hdip))	28	7
1933	((((ca_ent ca_wa)p_re)(p_csn cp_ent))(oa_ent u_isn))	39	7
1934	((((ca_ent ca_wa)p_re)(p_csn cp_ent))(p_csn oa_ent))	22	7
1935	((((ca_ent ca_wa)p_re)(p_csn oa_ent))(oa_wa u_hdip))	27	7
1936	((((ca_ent ca_wa)p_re)(p_csn oa_ent))(u_hdip u_hdip))	33	7
1937	((((ca_ent ca_wa)p_re)(p_csn oa_ent))(u_isn u_hdip))	34	7
1938	((((ca_ent ca_wa)p_re)(p_isn p_csn))(p_isn cp_ent))	45	7
1939	((((p_csn cp_ent)oa_ent)(u_isn u_hdip))(u_isn oa_wa))	22	7
1940	((((ca_ent p_re)p_csn)(oa_ent oa_wa))(u_isn u_hdip))	31	7
1941	((((ca_ent p_re)p_csn)(oa_ent u_hdip))(u_isn oa_wa))	31	7
1942	((((ca_ent p_re)p_csn)(oa_ent u_isn))(u_hdip u_hdip))	38	7
1943	((((ca_ent p_re)p_csn)(p_isn oa_ent))(u_isn u_hdip))	29	7
1944	((((ca_ent p_re)(cp_ent oa_ent))oa_wa)(u_hdip u_hdip))	23	7
1945	((((ca_ent p_re)(cp_ent oa_ent))oa_wa)(u_isn u_hdip))	28	7
1946	((((ca_wa ca_wa)(cp_ent oa_ent))oa_wa)(u_hdip u_hdip))	25	7
1947	((((ca_wa ca_wa)(cp_ent oa_ent))oa_wa)(u_isn u_hdip))	27	7
1948	((((ca_wa ca_wa)(p_csn oa_ent))oa_wa)(u_hdip u_hdip))	27	7
1949	((((ca_wa ca_wa)(p_csn oa_ent))oa_wa)(u_isn u_hdip))	28	7
1950	((((ca_wa p_csn)oa_ent)(u_hdip u_hdip))(u_isn u_hdip))	28	7
1951	((((ca_wa p_csn)oa_ent)(u_isn u_hdip))(u_isn u_hdip))	30	7
1952	((((ca_wa p_re)ca_wa)(cp_ent oa_ent))(oa_wa u_hdip))	44	7
1953	((((ca_wa p_re)ca_wa)(cp_ent oa_ent))(u_hdip u_hdip))	45	7
1954	((((ca_wa p_re)ca_wa)(cp_ent oa_ent))(u_isn oa_wa))	40	7
1955	((((ca_wa p_re)ca_wa)(cp_ent oa_ent))(u_isn u_hdip))	45	7
1956	((((ca_wa p_re)ca_wa)(oa_ent u_isn))(u_hdip u_hdip))	38	7
1957	((((ca_wa p_re)ca_wa)(p_csn oa_ent))(oa_wa u_hdip))	36	7
1958	((((ca_wa p_re)ca_wa)(p_csn oa_ent))(u_hdip u_hdip))	39	7
1959	((((ca_wa p_re)ca_wa)(p_csn oa_ent))(u_isn u_hdip))	41	7
1960	((((ca_wa p_re)ca_wa)(p_isn cp_ent))(oa_ent u_isn))	29	7
1961	((((ca_wa p_re)cp_ent)oa_ent)((u_hdip oa_wa)u_hdip))	35	7
1962	((((ca_wa p_re)cp_ent)oa_ent)((u_isn oa_wa)u_hdip))	39	7
1963	((((ca_wa p_re)cp_ent)(oa_ent oa_wa))(u_hdip u_hdip))	35	7
1964	((((ca_wa p_re)cp_ent)(oa_ent oa_wa))(u_isn oa_wa))	38	7
1965	((((ca_wa p_re)cp_ent)(oa_ent oa_wa))(u_isn u_hdip))	41	7
1966	((((ca_wa p_re)cp_ent)(oa_ent u_hdip))(u_isn oa_wa))	39	7
1967	((((ca_wa p_re)cp_ent)(oa_ent u_hdip))(u_isn u_hdip))	43	7
1968	((((ca_wa p_re)cp_ent)(oa_ent u_isn))(u_hdip u_hdip))	43	7
1969	((((ca_wa p_re)cp_ent)(p_isn oa_ent))(u_hdip u_hdip))	22	7
1970	((((cp_ent ca_ent)cp_ent)oa_ent)(u_hdip(u_isn oa_wa)))	35	7
1971	((((cp_ent ca_ent)cp_ent)oa_ent)((u_hdip oa_wa)u_hdip))	28	7
1972	((((cp_ent ca_ent)cp_ent)oa_ent)((u_isn oa_wa)u_hdip))	37	7
1973	((((cp_ent ca_ent)cp_ent)(oa_ent oa_wa))(u_hdip u_hdip))	34	7
1974	((((cp_ent ca_ent)cp_ent)(oa_ent oa_wa))(u_isn oa_wa))	40	7
1975	((((cp_ent ca_ent)cp_ent)(oa_ent oa_wa))(u_isn u_hdip))	42	7
1976	((((cp_ent ca_ent)cp_ent)(oa_ent u_hdip))(u_isn oa_wa))	39	7
1977	((((cp_ent ca_ent)cp_ent)(oa_ent u_hdip))(u_isn u_hdip))	42	7
1978	((((cp_ent ca_ent)cp_ent)(oa_ent u_isn))(u_hdip oa_wa))	32	7
1979	((((cp_ent ca_ent)cp_ent)(oa_ent u_isn))(u_hdip u_hdip))	43	7
1980	((((cp_ent ca_wa)cp_ent)oa_ent)((u_isn oa_wa)u_hdip))	36	7
1981	((((cp_ent ca_wa)cp_ent)(oa_ent oa_wa))(u_hdip u_hdip))	34	7
1982	((((cp_ent ca_wa)cp_ent)(oa_ent oa_wa))(u_isn oa_wa))	39	7
1983	((((cp_ent ca_wa)cp_ent)(oa_ent oa_wa))(u_isn u_hdip))	41	7
1984	((((cp_ent ca_wa)cp_ent)(oa_ent u_hdip))(u_isn oa_wa))	38	7
1985	((((cp_ent ca_wa)cp_ent)(oa_ent u_hdip))(u_isn u_hdip))	41	7
1986	((((cp_ent ca_wa)cp_ent)(oa_ent u_isn))(u_hdip u_hdip))	43	7
1987	((((cp_ent oa_ent)u_hdip)(oa_wa u_csn))(u_isn cp_ent))	43	7
1988	((((cp_ent oa_ent)(oa_wa u_hdip))u_csn)(u_isn cp_ent))	44	7
1989	((((oa_ent oa_wa)u_csn)(u_isn cp_ent))(ca_ent p_isn))	24	7
1990	((((oa_ent oa_wa)u_csn)(u_isn cp_ent))(ca_ent ca_wa))	25	7
1991	((((oa_ent oa_wa)u_csn)(u_isn cp_ent))(ca_wa ca_wa))	19	7
1992	((((oa_ent oa_wa)u_hdip)(oa_wa u_csn))(cp_ent oa_ent))	22	7
1993	((((oa_ent oa_wa)u_hdip)(oa_wa(u_csn cp_ent)))oa_ent)	40	7
1994	((((oa_ent u_isn)u_hdip)(oa_wa u_csn))(cp_ent oa_ent))	28	7
1995	((((oa_ent u_isn)u_hdip)(oa_wa u_csn))(u_isn cp_ent))	42	7
1996	((((oa_ent u_isn)u_hdip)(oa_wa(u_csn cp_ent)))oa_ent)	46	7
1997	((((oa_ent u_isn)u_hdip)((u_isn oa_wa)u_csn))oa_ent)	27	7
1998	((((oa_wa u_csn)p_csn)ca_ent)((ca_wa p_re)ca_wa))	31	7
1999	((((oa_wa u_csn)p_csn)(ca_ent p_isn))(p_re ca_wa))	31	7
2000	((((oa_wa u_csn)p_csn)(p_isn ca_ent))(ca_wa ca_wa))	26	7
2001	((((p_csn oa_ent)u_hdip)(oa_wa u_hdip))(u_isn u_hdip))	25	7
2002	((((p_csn oa_ent)u_hdip)(u_isn oa_wa))(u_csn cp_ent))	34	7
2003	((((p_csn oa_ent)(u_isn u_hdip))(u_isn oa_wa))u_csn)	34	7
2004	((((p_isn ca_wa)p_csn)oa_ent)((oa_wa u_hdip)oa_wa))	26	7
2005	((((p_isn ca_wa)p_csn)(oa_ent oa_wa))(u_hdip u_hdip))	24	7
2006	((((p_isn ca_wa)p_csn)(oa_ent oa_wa))(u_isn u_hdip))	24	7
2007	((((p_isn ca_wa)p_csn)(oa_ent u_isn))(u_hdip u_hdip))	31	7
2008	((((p_isn ca_wa)p_csn)(p_isn oa_ent))(u_hdip u_hdip))	22	7
2009	((((p_isn ca_wa)p_csn)(p_isn oa_ent))(u_isn u_hdip))	23	7
2010	((((p_isn oa_ent)oa_wa)u_csn)((cp_ent oa_ent)u_hdip))	18	7
2011	((((p_isn oa_ent)oa_wa)u_csn)(oa_ent(oa_wa u_hdip)))	17	7
2012	((((p_isn oa_ent)oa_wa)u_csn)(oa_ent(u_isn u_hdip)))	18	7
2013	((((p_isn oa_ent)oa_wa)u_csn)((cp_ent oa_ent)oa_wa))	17	7
2014	((((p_re ca_wa)oa_ent)(oa_wa u_hdip))(u_isn oa_wa))	28	7
2015	((((p_re ca_wa)oa_ent)(oa_wa u_hdip))(u_isn u_hdip))	30	7
2016	((((p_re ca_wa)oa_ent)(u_hdip u_hdip))(u_isn u_hdip))	33	7
2017	((((p_re ca_wa)oa_ent)(u_isn u_hdip))(u_isn oa_wa))	31	7
2018	((((p_re ca_wa)oa_ent)(u_isn u_hdip))(u_isn u_hdip))	34	7
2019	((((p_re ca_wa)p_csn)oa_ent)((u_isn oa_wa)u_hdip))	33	7
2020	((((p_re ca_wa)p_csn)(cp_ent oa_ent))(oa_wa u_hdip))	31	7
2021	((((p_re ca_wa)p_csn)(cp_ent oa_ent))(u_hdip u_hdip))	34	7
2022	((((p_re ca_wa)p_csn)(cp_ent oa_ent))(u_isn u_hdip))	37	7
2023	((((p_re ca_wa)p_csn)(oa_ent oa_wa))(u_hdip u_hdip))	30	7
2024	((((p_re ca_wa)p_csn)(oa_ent oa_wa))(u_isn u_hdip))	34	7
2025	((((p_re ca_wa)p_csn)(oa_ent u_hdip))(u_isn u_hdip))	36	7
2026	((((p_re ca_wa)p_csn)(oa_ent u_isn))(oa_wa u_hdip))	36	7
2027	((((p_re ca_wa)p_csn)(oa_ent u_isn))(u_hdip u_hdip))	40	7
2028	((((p_re ca_wa)p_csn)(p_isn oa_ent))(u_isn u_hdip))	31	7
2029	((((u_csn cp_ent)oa_ent)(u_isn u_hdip))(u_isn u_hdip))	56	7
2030	((((u_csn cp_ent)p_csn)ca_ent)((ca_wa p_re)ca_wa))	35	7
2031	((((u_csn cp_ent)p_csn)(ca_ent p_isn))(p_re ca_wa))	35	7

2032	((((u_csn cp_ent) p_csn)(p_isn ca_ent))(ca_wa ca_wa))	29	7
2033	((((u_hdip oa_wa) u_csn)(cp_ent ca_ent))(ca_wa ca_wa))	32	7
2034	((((u_hdip oa_wa) u_csn)(ca_ent ca_wa))(ca_wa p_csn))	24	7
2035	((((u_hdip oa_wa) u_csn)(cp_ent ca_ent))(ca_wa p_re))	32	7
2036	((((u_hdip oa_wa) u_csn)(cp_ent ca_ent))(p_isn ca_wa))	24	7
2037	((((u_hdip oa_wa) u_csn)(cp_ent ca_ent))(p_isn p_re))	31	7
2038	((((u_hdip oa_wa) u_csn)(ca_ent p_isn))(p_re ca_wa))	32	7
2039	((((u_hdip oa_wa) u_csn)(ca_ent ca_wa))(p_isn p_re))	31	7
2040	((((u_hdip oa_wa) u_csn)(cp_ent ca_wa))(p_isn p_re))	26	7
2041	((((u_hdip oa_wa) u_csn)(cp_ent ca_wa))(p_re ca_wa))	27	7
2042	((((u_hdip oa_wa) u_csn)((cp_ent ca_wa) cp_ent)) oa_ent)	21	7
2043	((((u_isn oa_wa) u_csn)(ca_ent ca_wa))(ca_wa p_csn))	24	7
2044	((((u_isn oa_wa) u_csn)(cp_ent ca_ent))(ca_wa ca_wa))	31	7
2045	((((u_isn oa_wa) u_csn)(cp_ent ca_ent))(ca_wa p_re))	31	7
2046	((((u_isn oa_wa) u_csn)(cp_ent ca_ent))(p_isn ca_wa))	23	7
2047	((((u_isn oa_wa) u_csn)(cp_ent ca_ent))(p_re ca_wa))	31	7
2048	((((u_isn oa_wa) u_csn)(cp_ent ca_wa))(p_re ca_wa))	26	7
2049	((((u_isn oa_wa) u_hdip)(oa_wa u_csn))(ca_ent ca_wa))	26	7
2050	((((u_isn oa_wa) u_hdip)(oa_wa u_csn))(cp_ent ca_ent))	26	7
2051	((((u_isn oa_wa) u_hdip)(oa_wa u_csn))(cp_ent ca_wa))	23	7
2052	((((u_isn oa_wa) u_hdip)(oa_wa u_csn))(oa_ent u_isn))	26	7
2053	((((u_isn u_csn)(u_isn cp_ent)) ca_ent)(ca_wa ca_wa))	27	7
2054	((((u_isn u_hdip)(oa_wa u_csn)) oa_ent)(oa_wa u_hdip))	24	7
2055	(p_csn ((p_isn ca_ent)((ca_wa p_re) ca_wa) oa_ent)))	26	7
2056	(p_csn ((p_isn ca_ent)((ca_wa p_re) cp_ent) oa_ent)))	31	7
2057	(p_csn ((((ca_ent ca_wa) cp_ent) oa_ent)(u_hdip u_hdip)))	28	7
2058	(p_csn ((((ca_ent ca_wa) cp_ent) oa_ent)(u_isn u_hdip)))	32	7
2059	((u_hdip u_hdip)((oa_wa (u_csn cp_ent)) p_csn) ca_ent))	28	7
2060	((p_csn ((ca_wa p_re)(ca_wa (p_csn cp_ent)))) oa_ent)	24	7
2061	(((p_csn (oa_ent (u_isn u_hdip)))(u_isn oa_wa)) u_csn)	36	7
2062	(((p_csn ((oa_ent u_isn) u_hdip))(u_isn oa_wa)) u_csn)	38	7
2063	((((oa_wa (u_csn cp_ent)) p_csn) ca_ent)(ca_wa ca_wa))	33	7
2064	((((oa_wa (u_csn cp_ent)) p_csn) ca_ent)(ca_wa p_re))	33	7
2065	(((p_csn (ca_wa p_re)) p_csn) oa_ent)(u_isn u_hdip))	21	7
2066	((((cp_ent oa_ent) oa_wa) u_csn)(u_isn cp_ent)) ca_ent)	27	7
2067	(((ca_ent ca_wa) p_re)((ca_wa (p_csn cp_ent))(oa_ent u_hdip)))	31	8
2068	((ca_ent ca_wa)((p_isn p_re)((ca_wa p_csn)(oa_ent u_isn))))	34	8
2069	((cp_ent ca_ent)((ca_wa p_re)((ca_wa p_csn)(oa_ent u_isn))))	32	8
2070	((p_isn ca_ent)((ca_wa p_re)((ca_wa p_csn)(oa_ent u_isn))))	28	8
2071	(((u_csn cp_ent)(ca_ent ca_wa))((p_re ca_wa)(cp_ent oa_ent)))	33	8
2072	((u_csn (cp_ent ca_ent))(((ca_wa p_re) ca_wa)(p_csn oa_ent)))	27	8
2073	(((ca_ent p_isn)(p_re (ca_wa p_csn)))((oa_ent oa_wa) u_hdip))	26	8
2074	(((u_csn cp_ent)(p_csn (ca_ent ca_wa)))((p_re ca_wa) oa_ent))	21	8
2075	((((ca_ent ca_wa) cp_ent)((p_csn oa_ent)(u_hdip (u_isn oa_wa))))	25	8
2076	((((ca_ent ca_wa) p_re)(p_csn cp_ent))((oa_ent oa_wa) u_hdip))	29	8
2077	((((ca_ent ca_wa) p_re)(p_csn cp_ent)) oa_ent)(u_isn u_hdip))	33	8
2078	((((ca_ent ca_wa) p_re)(p_csn cp_ent))((oa_ent u_isn) u_hdip))	34	8
2079	(((ca_ent ca_wa)(p_csn cp_ent))((p_csn oa_ent)(u_isn oa_wa)))	19	8
2080	(((ca_ent p_isn)(p_re (ca_wa p_csn)))(oa_ent (oa_wa u_hdip)))	25	8
2081	((ca_ent ca_wa)(((p_re ca_wa) p_csn)((p_isn oa_ent) u_hdip)))	27	8
2082	(((ca_ent p_re)(cp_ent oa_ent))((oa_wa u_hdip)(u_isn oa_wa)))	24	8
2083	(((ca_wa ca_wa)(cp_ent oa_ent))((oa_wa u_hdip)(u_isn oa_wa)))	25	8
2084	(((ca_wa ca_wa)(p_csn oa_ent))((oa_wa u_hdip)(u_isn oa_wa)))	24	8
2085	(((ca_wa p_re)(p_csn oa_ent))((oa_wa u_hdip)(u_isn oa_wa)))	23	8
2086	(((cp_ent ca_ent)(ca_wa p_re))((ca_wa p_csn)(p_isn cp_ent)))	47	8
2087	(((cp_ent ca_ent)(ca_wa p_re))((p_csn cp_ent)(oa_ent oa_wa)))	28	8
2088	(((cp_ent oa_ent)(oa_wa u_hdip))((u_isn u_hdip)(oa_wa u_csn)))	39	8
2089	(((cp_ent oa_ent)(u_isn u_hdip))((u_isn u_hdip)(oa_wa u_csn)))	40	8
2090	(((oa_wa oa_wa)(cp_ent ca_wa))((p_re ca_wa)(p_csn p_csn)))	18	8
2091	((((oa_wa u_csn)(cp_ent ca_wa))(p_isn p_re))(ca_wa p_csn))	24	8
2092	(((oa_wa u_csn)(cp_ent ca_wa))((p_re ca_wa)(cp_ent oa_ent)))	24	8
2093	(((oa_wa u_hdip)(u_isn oa_wa))((u_csn cp_ent)(ca_ent p_isn)))	26	8
2094	(((oa_wa u_hdip)(u_isn oa_wa))((u_csn cp_ent)(ca_ent ca_wa)))	28	8
2095	(((oa_wa u_hdip)(u_isn oa_wa))((u_csn cp_ent)(ca_wa ca_wa)))	22	8
2096	(((oa_wa u_hdip)(u_isn oa_wa))((u_csn cp_ent)(p_isn ca_wa)))	22	8
2097	((((p_isn ca_ent)(ca_wa p_re))(ca_wa p_csn))(cp_ent oa_ent))	29	8
2098	((((p_isn ca_ent)(ca_wa p_re))(ca_wa p_csn))(oa_ent u_hdip))	26	8
2099	((((p_isn ca_ent)(ca_wa p_re))(ca_wa p_csn))(oa_ent u_isn))	29	8
2100	(((p_isn ca_ent)(ca_wa p_re))((p_csn cp_ent)(oa_ent u_isn)))	30	8
2101	((p_isn oa_ent)((u_isn u_hdip)((u_isn u_hdip)(oa_wa u_csn))))	33	8
2102	(((p_isn oa_ent)(oa_wa u_hdip))((u_isn u_hdip)(oa_wa u_csn)))	30	8
2103	(((p_re ca_wa)(cp_ent oa_ent))((oa_wa u_hdip)(u_isn oa_wa)))	33	8
2104	(((p_re ca_wa)(p_csn oa_ent))((oa_wa u_hdip)(u_isn oa_wa)))	22	8
2105	(((p_re ca_wa)(p_isn p_re))((p_csn cp_ent)(oa_ent u_isn)))	21	8
2106	(((p_re ca_wa)(p_isn p_re))((p_isn p_csn)(cp_ent oa_ent)))	20	8
2107	((((u_csn cp_ent)(ca_ent ca_wa))(p_isn p_re))(ca_wa p_csn))	33	8
2108	(((u_csn cp_ent)(ca_wa ca_wa))((cp_ent oa_ent)(oa_wa u_hdip)))	27	8
2109	(((u_csn cp_ent)(ca_wa p_re))((cp_ent oa_ent)(u_isn u_hdip)))	32	8
2110	(((u_csn cp_ent)(ca_wa p_re))((p_csn oa_ent)(u_isn u_hdip)))	28	8
2111	(((u_csn cp_ent)(oa_ent oa_wa))((u_isn u_hdip)(oa_wa u_csn)))	30	8
2112	((((u_csn cp_ent)(p_isn ca_ent))(p_isn p_re))(ca_wa p_csn))	27	8
2113	(((u_csn cp_ent)(p_isn ca_ent))((p_re ca_wa)(cp_ent oa_ent)))	28	8
2114	((((u_csn cp_ent)(p_isn ca_wa))(p_isn p_re))(ca_wa p_csn))	24	8
2115	(((u_csn cp_ent)(p_isn ca_wa))((p_re ca_wa)(cp_ent oa_ent)))	25	8
2116	(((u_hdip (u_csn cp_ent))(ca_ent ca_wa))((p_re ca_wa) oa_ent))	21	8
2117	(((u_hdip u_csn)(cp_ent ca_wa))((p_re ca_wa)(cp_ent oa_ent)))	23	8
2118	((((u_isn cp_ent)(ca_ent ca_wa))(p_isn p_re))(ca_wa p_csn))	25	8
2119	(((u_isn cp_ent)(ca_wa ca_wa))((cp_ent oa_ent)(oa_wa u_hdip)))	21	8
2120	(((u_isn u_csn)(ca_ent ca_wa))((p_re ca_wa)(cp_ent oa_ent)))	23	8
2121	(((u_isn u_hdip)(oa_wa (u_csn cp_ent)))(p_csn (ca_ent ca_wa)))	22	8
2122	(p_csn (((p_isn oa_ent)(oa_wa u_hdip))((u_isn oa_wa) u_csn)))	21	8
2123	(p_csn (((p_isn oa_ent)(u_isn u_hdip))((u_isn oa_wa) u_csn)))	24	8
2124	((ca_ent ca_wa)((p_isn ca_wa)((p_csn cp_ent)(oa_ent u_isn))))	35	8
2125	((ca_ent ca_wa)((p_isn ca_wa)((p_csn cp_ent)(oa_ent oa_wa))))	29	8
2126	((ca_ent ca_wa)((p_isn ca_wa)((p_csn cp_ent)(oa_ent u_hdip))))	30	8
2127	((ca_ent ca_wa)((p_re ca_wa)((cp_ent oa_ent)(oa_wa u_hdip))))	41	8
2128	((ca_ent ca_wa)((p_re ca_wa)((cp_ent oa_ent)(u_isn u_hdip))))	38	8
2129	((ca_ent ca_wa)((p_re ca_wa)((p_csn cp_ent)(oa_ent u_isn))))	38	8
2130	((ca_ent ca_wa)((p_re ca_wa)((p_csn cp_ent)(oa_ent oa_wa))))	32	8
2131	((ca_ent ca_wa)((p_re ca_wa)((p_csn cp_ent)(p_csn oa_ent))))	20	8
2132	((ca_ent ca_wa)((p_re ca_wa)((p_csn oa_ent)(oa_wa u_hdip))))	37	8
2133	((ca_ent ca_wa)((p_re ca_wa)((p_csn oa_ent)(u_isn oa_wa))))	29	8
2134	((ca_ent ca_wa)((p_re ca_wa)((p_csn oa_ent)(u_isn u_hdip))))	39	8
2135	((ca_ent ca_wa)((p_re (ca_wa p_csn))((oa_ent oa_wa) u_hdip)))	25	8
2136	(((ca_ent p_isn)((p_re (ca_wa p_csn)) oa_ent))(u_hdip u_hdip))	30	8
2137	(((ca_ent ca_wa)(((p_isn ca_wa) p_csn) oa_ent))(u_isn u_hdip))	31	8
2138	(((ca_ent p_re)((ca_wa (p_csn cp_ent)) oa_ent))(u_hdip u_hdip))	31	8
2139	(((ca_ent p_re)((ca_wa (p_csn cp_ent)) oa_ent))(u_isn u_hdip))	31	8
2140	((ca_ent ca_wa)(((p_isn ca_wa) p_csn)(oa_ent (oa_wa u_hdip))))	24	8
2141	((ca_ent ca_wa)(((p_isn ca_wa) p_csn)(oa_ent (u_isn u_hdip))))	30	8
2142	((ca_ent ca_wa)(((p_isn ca_wa) p_csn)((p_isn oa_ent) u_hdip)))	22	8
2143	((ca_ent ca_wa)((p_isn p_re)((ca_wa (p_csn cp_ent)) oa_ent)))	37	8
2144	((ca_ent ca_wa)(((p_re ca_wa) p_csn)(oa_ent (oa_wa u_hdip))))	32	8
2145	((ca_ent ca_wa)(((p_re ca_wa) p_csn)(oa_ent (u_isn u_hdip))))	39	8
2146	((ca_ent ca_wa)(((p_re ca_wa) p_csn)((oa_ent oa_wa) u_hdip)))	35	8
2147	((ca_ent ca_wa)(((p_re ca_wa)(cp_ent oa_ent))(oa_wa u_hdip)))	39	8
2148	((ca_ent ca_wa)(((p_re ca_wa)(cp_ent oa_ent))(u_hdip u_hdip)))	43	8
2149	((ca_ent ca_wa)(((p_re ca_wa)(cp_ent oa_ent))(u_isn u_hdip)))	43	8
2150	((ca_ent ca_wa)(((p_re ca_wa)(p_csn cp_ent))(oa_ent oa_wa)))	33	8
2151	((ca_ent ca_wa)(((p_re ca_wa)(p_csn cp_ent))(oa_ent u_hdip)))	34	8
2152	((ca_ent ca_wa)(((p_re ca_wa)(p_csn oa_ent))(oa_wa u_hdip)))	36	8
2153	((ca_ent ca_wa)(((p_re ca_wa)(p_csn oa_ent))(u_hdip u_hdip)))	39	8
2154	((ca_ent ca_wa)(((p_re ca_wa)(p_csn oa_ent))(u_isn u_hdip)))	41	8
2155	((ca_ent p_isn)((p_re ca_wa)((cp_ent oa_ent)(oa_wa u_hdip))))	40	8
2156	((ca_ent p_isn)((p_re ca_wa)((cp_ent oa_ent)(u_isn u_hdip))))	37	8
2157	((ca_ent ca_wa)((p_isn p_re)((ca_wa (p_csn cp_ent)) p_csn)))	37	8
2158	((ca_ent p_isn)((p_re ca_wa)((p_csn oa_ent)(oa_wa u_hdip))))	36	8
2159	((ca_ent p_isn)((p_re ca_wa)((p_csn oa_ent)(u_isn oa_wa))))	27	8
2160	((ca_ent p_isn)((p_re ca_wa)((p_csn oa_ent)(u_isn u_hdip))))	38	8
2161	((ca_ent p_isn)((ca_wa (p_csn cp_ent))((oa_ent u_isn) u_hdip)))	29	8
2162	((ca_ent p_isn)((ca_wa (p_csn cp_ent))(oa_ent (oa_wa u_hdip))))	25	8
2163	((ca_ent p_isn)((ca_wa (p_csn cp_ent))(oa_ent (u_isn u_hdip))))	28	8
2164	((ca_ent p_isn)(((ca_wa p_csn)(oa_ent u_hdip))(u_isn u_hdip)))	27	8
2165	((ca_ent p_isn)(((p_re ca_wa) p_csn)(oa_ent (oa_wa u_hdip))))	28	8
2166	((ca_ent p_isn)(((p_re ca_wa) p_csn)(oa_ent (u_isn u_hdip))))	38	8
2167	((ca_ent p_isn)(((p_re ca_wa) p_csn)((oa_ent oa_wa) u_hdip)))	33	8
2168	((ca_ent p_isn)(((p_re ca_wa) p_csn)((p_isn oa_ent) u_hdip)))	25	8
2169	((ca_ent p_isn)(((p_re ca_wa)(cp_ent oa_ent))(oa_wa u_hdip)))	38	8
2170	((ca_ent p_isn)(((p_re ca_wa)(p_csn cp_ent))(oa_ent oa_wa)))	31	8
2171	((ca_ent p_isn)(((p_re ca_wa)(p_csn cp_ent))(oa_ent u_hdip)))	32	8
2172	((ca_ent p_isn)(((p_re ca_wa)(p_csn oa_ent))(oa_wa u_hdip)))	35	8
2173	((ca_ent p_isn)(((p_re ca_wa)(p_csn oa_ent))(u_hdip u_hdip)))	38	8
2174	((ca_ent p_isn)(((p_re ca_wa)(p_csn oa_ent))(u_isn u_hdip)))	40	8
2175	(((ca_ent ca_wa) p_re)((ca_wa (p_csn cp_ent))(oa_ent oa_wa)))	30	8
2176	((ca_wa ca_wa)(((p_csn cp_ent)(oa_ent oa_wa))(u_isn u_hdip)))	29	8
2177	((ca_wa ca_wa)((p_re p_re)((ca_wa p_csn)(cp_ent oa_ent))))	24	8
2178	((ca_wa ca_wa)(((p_csn cp_ent)(oa_ent u_isn))(u_hdip u_hdip)))	33	8
2179	((ca_wa ca_wa)(((p_csn cp_ent) oa_ent)((u_isn oa_wa) u_hdip)))	28	8
2180	((ca_wa ca_wa)(((p_csn cp_ent)(oa_ent u_hdip))(u_isn u_hdip)))	31	8
2181	((ca_wa ca_wa)(((p_csn cp_ent)(oa_ent u_isn))(oa_wa u_hdip)))	30	8
2182	((ca_wa ca_wa)(((p_csn oa_ent)(u_isn u_hdip))(u_isn u_hdip)))	33	8
2183	((ca_wa ca_wa)((p_isn p_re)((p_csn cp_ent)(oa_ent oa_wa))))	25	8
2184	((ca_wa p_re)(((p_csn cp_ent)(oa_ent oa_wa))(u_isn u_hdip)))	29	8
2185	((ca_wa p_re)((ca_wa (p_csn cp_ent))((oa_ent u_isn) u_hdip)))	33	8
2186	((ca_wa p_re)((ca_wa (p_csn cp_ent))(oa_ent (oa_wa u_hdip))))	29	8
2187	((ca_wa p_re)((ca_wa (p_csn cp_ent))(oa_ent (u_isn u_hdip))))	32	8
2188	((ca_wa p_re)(((ca_wa p_csn)(oa_ent u_isn))(u_hdip u_hdip)))	31	8
2189	((ca_wa p_re)(((ca_wa p_csn)(cp_ent oa_ent))(u_isn u_hdip)))	35	8
2190	((ca_wa p_re)(((ca_wa p_csn)(oa_ent u_hdip))(u_isn u_hdip)))	30	8
2191	((ca_wa p_re)(((p_csn cp_ent)(oa_ent u_isn))(u_hdip u_hdip)))	33	8
2192	((ca_wa p_re)(((p_csn cp_ent) oa_ent)((u_isn oa_wa) u_hdip)))	28	8
2193	((ca_wa p_re)(((p_csn cp_ent)(oa_ent u_hdip))(u_isn u_hdip)))	31	8
2194	((ca_wa p_re)(((p_csn cp_ent)(oa_ent u_isn))(oa_wa u_hdip)))	30	8
2195	((ca_wa p_re)(((p_csn oa_ent) u_hdip)((u_isn oa_wa) u_hdip)))	27	8
2196	((ca_wa p_re)(((p_csn oa_ent)(u_isn u_hdip))(oa_wa u_hdip)))	28	8
2197	((ca_wa p_re)(((p_csn oa_ent)(u_isn u_hdip))(u_isn oa_wa)))	26	8
2198	((ca_wa p_re)(((p_csn oa_ent)(u_isn u_hdip))(u_isn u_hdip)))	33	8
2199	((cp_ent ca_ent)(((ca_wa p_re) ca_wa)(p_csn (cp_ent oa_ent))))	30	8
2200	((cp_ent ca_ent)(((ca_wa p_re) ca_wa)((p_csn cp_ent) oa_ent)))	36	8
2201	((cp_ent ca_ent)((ca_wa p_re)((ca_wa p_csn)(cp_ent oa_ent))))	35	8
2202	((cp_ent ca_ent)((ca_wa p_re)((ca_wa p_csn)(p_isn cp_ent))))	48	8
2203	((cp_ent ca_ent)((p_isn ca_wa)((p_csn cp_ent)(oa_ent u_isn))))	33	8
2204	((cp_ent ca_ent)((p_isn ca_wa)((p_csn cp_ent)(oa_ent oa_wa))))	27	8
2205	((cp_ent ca_ent)((p_isn ca_wa)((p_csn cp_ent)(oa_ent u_hdip))))	28	8
2206	((cp_ent ca_ent)((p_isn p_re)((ca_wa p_csn)(cp_ent oa_ent))))	34	8
2207	((cp_ent ca_ent)((p_isn p_re)((ca_wa p_csn)(oa_ent u_isn))))	32	8
2208	((cp_ent ca_ent)((p_isn p_re)((ca_wa p_csn)(p_isn cp_ent))))	45	8
2209	((cp_ent ca_ent)(((ca_wa p_re) ca_wa)(p_csn (oa_ent oa_wa))))	30	8
2210	((cp_ent ca_ent)(((ca_wa p_re) ca_wa)(p_csn (p_isn oa_ent))))	28	8
2211	((cp_ent ca_ent)(((ca_wa p_re) ca_wa)((cp_ent oa_ent) u_hdip)))	43	8
2212	((cp_ent ca_ent)((p_re ca_wa)((cp_ent oa_ent)(oa_wa u_hdip))))	38	8
2213	((cp_ent ca_ent)((p_re ca_wa)((cp_ent oa_ent)(u_isn u_hdip))))	35	8
2214	((cp_ent ca_ent)((p_re ca_wa)((p_csn cp_ent)(oa_ent u_isn))))	36	8
2215	((cp_ent ca_ent)((p_re ca_wa)((p_csn oa_ent)(u_isn oa_wa))))	25	8
2216	((cp_ent ca_ent)((p_re ca_wa)((p_csn oa_ent)(u_isn u_hdip))))	36	8
2217	((cp_ent ca_ent)((ca_wa (p_csn cp_ent))(oa_ent (oa_wa u_hdip))))	25	8
2218	((cp_ent ca_ent)((p_re (ca_wa p_csn))((oa_ent oa_wa) u_hdip)))	23	8
2219	((cp_ent ca_ent)(((ca_wa ca_wa)(cp_ent oa_ent))(oa_wa u_hdip)))	21	8
2220	((cp_ent ca_ent)(((ca_wa ca_wa)(cp_ent oa_ent))(u_hdip u_hdip)))	31	8
2221	((cp_ent ca_ent)(((ca_wa ca_wa)(cp_ent oa_ent))(u_isn u_hdip)))	31	8
2222	((cp_ent ca_ent)(((ca_wa ca_wa)(p_csn oa_ent))(oa_wa u_hdip)))	22	8
2223	((cp_ent ca_ent)(((ca_wa ca_wa)(p_csn oa_ent))(u_hdip u_hdip)))	30	8
2224	((cp_ent ca_ent)(((ca_wa ca_wa)(p_csn oa_ent))(u_isn u_hdip)))	30	8
2225	(((cp_ent ca_ent)(((p_isn ca_wa) p_csn) oa_ent))(u_hdip u_hdip))	28	8
2226	(((cp_ent ca_ent)(((p_isn ca_wa) p_csn) oa_ent))(u_isn u_hdip))	29	8
2227	((cp_ent ca_ent)(((ca_wa p_re) ca_wa)(p_csn (oa_ent u_isn))))	45	8
2228	((cp_ent ca_ent)(((ca_wa p_re) ca_wa)(p_csn (oa_ent u_hdip))))	32	8
2229	((cp_ent ca_ent)(((ca_wa p_re) ca_wa)((cp_ent oa_ent) oa_wa)))	41	8
2230	((cp_ent ca_ent)(((ca_wa p_re) ca_wa)((p_csn oa_ent) oa_wa)))	34	8
2231	((cp_ent ca_ent)(((ca_wa p_re) ca_wa)((p_csn oa_ent) u_hdip)))	35	8
2232	((cp_ent ca_ent)(((ca_wa p_re) cp_ent)(oa_ent (oa_wa u_hdip))))	36	8
2233	((cp_ent ca_ent)(((ca_wa p_re) cp_ent)(oa_ent (u_isn u_hdip))))	39	8
2234	((cp_ent ca_ent)(((ca_wa p_re) cp_ent)((oa_ent oa_wa) u_hdip)))	39	8
2235	((cp_ent ca_ent)(((ca_wa p_re) cp_ent)((oa_ent u_isn) u_hdip)))	41	8
2236	((cp_ent ca_ent)(((ca_wa p_re)(p_csn cp_ent))(oa_ent u_isn)))	36	8
2237	((cp_ent ca_ent)(((ca_wa p_re)(p_csn oa_ent))(u_hdip u_hdip)))	32	8
2238	((cp_ent ca_ent)(((ca_wa p_re)(p_csn oa_ent))(u_isn u_hdip)))	35	8
2239	((cp_ent ca_ent)(((p_isn ca_wa) p_csn)(oa_ent (oa_wa u_hdip))))	22	8
2240	((cp_ent ca_ent)(((p_isn ca_wa) p_csn)(oa_ent (u_isn u_hdip))))	28	8
2241	((cp_ent ca_ent)(((p_isn ca_wa) p_csn)((p_isn oa_ent) u_hdip)))	22	8
2242	((cp_ent ca_ent)(((p_isn p_re)(p_csn cp_ent))(oa_ent u_isn)))	35	8
2243	((cp_ent ca_ent)(((p_re ca_wa) p_csn)(oa_ent (oa_wa u_hdip))))	26	8
2244	((cp_ent ca_ent)(((p_re ca_wa) p_csn)(oa_ent (u_isn u_hdip))))	36	8
2245	((cp_ent ca_ent)(((p_re ca_wa) p_csn)((oa_ent oa_wa) u_hdip)))	29	8
2246	((cp_ent ca_ent)(((p_re ca_wa) p_csn)((p_isn oa_ent) u_hdip)))	25	8
2247	((cp_ent ca_ent)(((p_re ca_wa)(cp_ent oa_ent))(oa_wa u_hdip)))	36	8
2248	((cp_ent ca_ent)(((p_re ca_wa)(cp_ent oa_ent))(u_hdip u_hdip)))	40	8
2249	((cp_ent ca_ent)(((p_re ca_wa)(cp_ent oa_ent))(u_isn u_hdip)))	40	8
2250	((cp_ent ca_ent)(((p_re ca_wa)(p_csn oa_ent))(u_hdip u_hdip)))	36	8
2251	((cp_ent ca_wa)((p_isn p_re)((ca_wa p_csn)(oa_ent u_isn))))	30	8
2252	((cp_ent ca_wa)((p_isn p_re)((ca_wa p_csn)(p_isn cp_ent))))	37	8
2253	((cp_ent ca_wa)((p_re ca_wa)((cp_ent oa_ent)(oa_wa u_hdip))))	35	8
2254	((cp_ent ca_wa)((p_re ca_wa)((cp_ent oa_ent)(u_isn u_hdip))))	33	8
2255	((cp_ent ca_wa)((p_re ca_wa)((p_csn oa_ent)(oa_wa u_hdip))))	32	8
2256	((cp_ent ca_wa)((p_re ca_wa)((p_csn oa_ent)(u_isn u_hdip))))	35	8
2257	(((cp_ent ca_wa)(((p_isn ca_wa) p_csn) oa_ent))(u_isn u_hdip))	27	8
2258	((cp_ent ca_wa)(((p_isn ca_wa) p_csn)((p_isn oa_ent) u_hdip)))	20	8
2259	((cp_ent ca_wa)(((p_re ca_wa) p_csn)(oa_ent (u_isn u_hdip))))	35	8
2260	((cp_ent ca_wa)(((p_re ca_wa) p_csn)((oa_ent oa_wa) u_hdip)))	30	8
2261	((cp_ent ca_wa)(((p_re ca_wa) p_csn)((p_isn oa_ent) u_hdip)))	23	8
2262	((cp_ent ca_wa)(((p_re ca_wa)(cp_ent oa_ent))(oa_wa u_hdip)))	33	8
2263	((cp_ent ca_wa)(((p_re ca_wa)(cp_ent oa_ent))(u_hdip u_hdip)))	38	8
2264	((cp_ent ca_wa)(((p_re ca_wa)(cp_ent oa_ent))(u_isn u_hdip)))	37	8
2265	((cp_ent ca_wa)(((p_re ca_wa)(p_csn oa_ent))(oa_wa u_hdip)))	31	8
2266	((cp_ent ca_wa)(((p_re ca_wa)(p_csn oa_ent))(u_hdip u_hdip)))	34	8
2267	((cp_ent ca_wa)(((p_re ca_wa)(p_csn oa_ent))(u_isn u_hdip)))	36	8
2268	((cp_ent oa_ent)((oa_wa u_hdip)((u_isn u_hdip)(oa_wa u_csn))))	35	8
2269	((cp_ent oa_ent)((u_isn u_hdip)((u_isn u_hdip)(oa_wa u_csn))))	41	8
2270	((oa_ent oa_wa)((u_hdip u_hdip)((oa_wa u_csn)(cp_ent oa_ent))))	23	8
2271	((oa_ent oa_wa)((u_hdip u_hdip)((oa_wa u_csn)(u_isn cp_ent))))	30	8
2272	((oa_ent oa_wa)((u_isn u_hdip)((u_isn u_hdip)(oa_wa u_csn))))	36	8
2273	(((oa_ent u_isn) u_hdip)((u_isn u_hdip)(oa_wa (u_csn cp_ent))))	40	8
2274	((oa_ent u_hdip)(((u_isn u_hdip)(oa_wa u_csn))(cp_ent oa_ent)))	29	8
2275	((oa_wa oa_wa)((u_csn (cp_ent ca_ent))((ca_wa p_re) ca_wa)))	24	8
2276	(((oa_wa oa_wa)(u_csn (cp_ent ca_ent)))((ca_wa p_re) ca_wa))	23	8
2277	((oa_wa oa_wa)(((u_csn cp_ent)(p_csn ca_ent))(ca_wa ca_wa)))	20	8
2278	((oa_wa oa_wa)(((u_csn cp_ent)(p_csn ca_ent))(ca_wa p_re)))	20	8
2279	((oa_wa oa_wa)(((u_csn cp_ent)(p_csn ca_wa))(p_re ca_wa)))	19	8
2280	((oa_wa oa_wa)(((u_csn cp_ent)(p_isn ca_ent))(ca_wa ca_wa)))	20	8
2281	((oa_wa oa_wa)(((u_csn cp_ent)(p_isn ca_ent))(ca_wa p_re)))	20	8
2282	((oa_wa oa_wa)(((u_csn cp_ent)(ca_ent ca_wa))(p_isn p_re)))	24	8
2283	((oa_wa oa_wa)(((u_csn cp_ent)(ca_ent ca_wa))(p_re ca_wa)))	26	8
2284	((oa_wa oa_wa)(((u_csn cp_ent)(p_isn ca_wa))(p_isn p_re)))	22	8
2285	((oa_wa oa_wa)(((u_csn cp_ent)(p_isn ca_wa))(p_re ca_wa)))	23	8
2286	(((oa_wa u_csn)((u_isn cp_ent)(ca_ent p_isn)))(p_re ca_wa))	28	8
2287	((oa_wa u_csn)(((u_isn cp_ent) ca_ent)((ca_wa p_re) ca_wa)))	28	8
2288	((oa_wa u_csn)(((u_isn cp_ent)(ca_ent ca_wa))(p_isn p_re)))	28	8
2289	((oa_wa u_csn)(((u_isn cp_ent)(ca_ent ca_wa))(p_re ca_wa)))	29	8
2290	(((oa_wa u_csn)((u_isn cp_ent)(ca_ent ca_wa)))(ca_wa p_csn))	24	8
2291	(((oa_wa u_csn)((u_isn cp_ent)(ca_ent ca_wa)))(p_re p_csn))	24	8
2292	((oa_wa u_csn)(((u_isn cp_ent) ca_ent)((ca_wa p_re) cp_ent)))	27	8
2293	((oa_wa u_csn)(((u_isn cp_ent)(ca_wa p_re))(ca_wa p_csn)))	21	8
2294	((oa_wa u_hdip)((u_isn u_hdip)((oa_wa u_csn)(cp_ent oa_ent))))	24	8
2295	((oa_wa u_hdip)((u_isn u_hdip)((oa_wa u_csn)(oa_ent oa_wa))))	25	8
2296	((oa_wa u_hdip)(((u_csn cp_ent)(p_csn ca_ent))(ca_wa ca_wa)))	25	8
2297	((oa_wa u_hdip)(((u_csn cp_ent)(p_csn ca_ent))(ca_wa p_re)))	25	8
2298	((oa_wa u_hdip)(((u_csn cp_ent)(p_csn ca_ent))(p_re ca_wa)))	25	8
2299	((oa_wa u_hdip)(((u_isn oa_wa) u_csn)((cp_ent ca_wa) cp_ent)))	17	8
2300	((oa_wa u_hdip)(((u_isn u_hdip)(oa_wa u_csn))(oa_ent u_isn)))	19	8
2301	((p_csn ca_ent)(((ca_wa p_re) ca_wa)((cp_ent oa_ent) u_hdip)))	30	8
2302	((p_csn ca_ent)(((ca_wa p_re) ca_wa)((cp_ent oa_ent) oa_wa)))	29	8
2303	((p_csn ca_ent)(((p_re ca_wa)(cp_ent oa_ent))(u_isn u_hdip)))	25	8
2304	((p_isn ca_ent)(((ca_wa p_re) ca_wa)(p_csn (cp_ent oa_ent))))	24	8
2305	(((p_isn ca_ent)(ca_wa p_re))((ca_wa (p_csn cp_ent)) oa_ent))	28	8
2306	((p_isn ca_ent)((ca_wa p_re)((p_csn oa_ent)(oa_wa u_hdip))))	26	8
2307	((p_isn ca_ent)((p_isn ca_wa)((p_csn cp_ent)(oa_ent u_isn))))	27	8
2308	((p_isn ca_ent)((p_isn ca_wa)((p_csn cp_ent)(oa_ent oa_wa))))	21	8
2309	((p_isn ca_ent)((p_isn ca_wa)((p_csn cp_ent)(oa_ent u_hdip))))	24	8
2310	((p_isn ca_ent)((p_isn p_re)((ca_wa p_csn)(cp_ent oa_ent))))	28	8
2311	((p_isn ca_ent)((p_isn p_re)((ca_wa p_csn)(oa_ent u_isn))))	28	8
2312	((p_isn ca_ent)(((ca_wa p_re) ca_wa)(p_csn (oa_ent u_hdip))))	30	8
2313	((p_isn ca_ent)(((ca_wa p_re) ca_wa)(p_csn (oa_ent u_isn))))	37	8
2314	((p_isn ca_ent)(((ca_wa p_re) ca_wa)(p_csn (p_isn oa_ent))))	27	8
2315	((p_isn ca_ent)(((ca_wa p_re) ca_wa)((cp_ent oa_ent) oa_wa)))	34	8
2316	(((p_isn ca_ent)(ca_wa p_re))((ca_wa (p_csn cp_ent)) p_csn))	30	8
2317	((p_isn ca_ent)(((ca_wa p_re) ca_wa)((p_csn oa_ent) u_hdip)))	32	8
2318	((p_isn ca_ent)((p_re ca_wa)((p_csn oa_ent)(oa_wa u_hdip))))	25	8
2319	((p_isn ca_ent)((p_re ca_wa)((p_csn oa_ent)(u_isn oa_wa))))	21	8
2320	((p_isn ca_ent)((p_re (ca_wa p_csn))((oa_ent oa_wa) u_hdip)))	18	8
2321	((p_isn ca_ent)(((ca_wa ca_wa)(cp_ent oa_ent))(u_hdip u_hdip)))	29	8
2322	((p_isn ca_ent)(((ca_wa ca_wa)(cp_ent oa_ent))(u_isn u_hdip)))	28	8
2323	((p_isn ca_ent)(((ca_wa ca_wa)(p_csn oa_ent))(u_hdip u_hdip)))	28	8
2324	((p_isn ca_ent)(((ca_wa ca_wa)(p_csn oa_ent))(u_isn u_hdip)))	28	8
2325	(((p_isn ca_ent)(((p_isn ca_wa) p_csn) oa_ent))(u_hdip u_hdip))	24	8
2326	(((p_isn ca_ent)(((p_isn ca_wa) p_csn) oa_ent))(u_isn u_hdip))	25	8
2327	((p_isn ca_ent)(((ca_wa p_re) ca_wa)(p_csn (oa_ent oa_wa))))	26	8
2328	((p_isn ca_ent)(((ca_wa p_re) ca_wa)((cp_ent oa_ent) u_hdip)))	38	8
2329	((p_isn ca_ent)(((ca_wa p_re) ca_wa)((p_csn oa_ent) oa_wa)))	29	8
2330	((p_isn ca_ent)(((ca_wa p_re) cp_ent)(oa_ent (oa_wa u_hdip))))	33	8
2331	((p_isn ca_ent)(((ca_wa p_re) cp_ent)(oa_ent (u_isn u_hdip))))	34	8
2332	((p_isn ca_ent)(((ca_wa p_re) cp_ent)((oa_ent oa_wa) u_hdip)))	34	8
2333	((p_isn ca_ent)(((ca_wa p_re) cp_ent)((oa_ent u_isn) u_hdip)))	36	8
2334	((p_isn ca_ent)(((ca_wa p_re) cp_ent)((p_isn oa_ent) u_hdip)))	25	8
2335	((p_isn ca_ent)(((ca_wa p_re)(p_csn oa_ent))(oa_wa u_hdip)))	26	8
2336	((p_isn ca_ent)(((ca_wa p_re)(p_csn oa_ent))(u_hdip u_hdip)))	29	8
2337	((p_isn ca_ent)(((ca_wa p_re)(p_csn oa_ent))(u_isn u_hdip)))	32	8
2338	((p_isn ca_ent)(((p_isn ca_wa) p_csn)(oa_ent (u_isn u_hdip))))	24	8
2339	((p_isn ca_ent)(((p_isn ca_wa) p_csn)((p_isn oa_ent) u_hdip)))	21	8
2340	((p_isn ca_ent)((p_isn p_re)((ca_wa (p_csn cp_ent)) oa_ent)))	28	8
2341	((p_isn ca_ent)(((p_re ca_wa) p_csn)(oa_ent (oa_wa u_hdip))))	21	8
2342	((p_isn ca_ent)(((p_re ca_wa) p_csn)(oa_ent (u_isn u_hdip))))	31	8
2343	((p_isn ca_ent)(((p_re ca_wa) p_csn)((oa_ent oa_wa) u_hdip)))	22	8
2344	((p_isn ca_ent)(((p_re ca_wa)(cp_ent oa_ent))(oa_wa u_hdip)))	31	8
2345	((p_isn ca_ent)(((p_re ca_wa)(cp_ent oa_ent))(u_hdip u_hdip)))	35	8
2346	((p_isn ca_ent)(((p_re ca_wa)(cp_ent oa_ent))(u_isn u_hdip)))	35	8
2347	((p_isn ca_ent)(((p_re ca_wa)(p_csn oa_ent))(oa_wa u_hdip)))	24	8
2348	((p_isn ca_wa)(((p_csn cp_ent)(oa_ent oa_wa))(u_isn u_hdip)))	29	8
2349	((p_isn ca_wa)((p_isn p_re)((ca_wa p_csn)(oa_ent u_hdip))))	30	8
2350	((p_isn ca_wa)((p_isn p_re)((ca_wa p_csn)(oa_ent u_isn))))	33	8
2351	((p_isn ca_wa)((p_re ca_wa)((cp_ent oa_ent)(oa_wa u_hdip))))	39	8
2352	((p_isn ca_wa)((p_re ca_wa)((cp_ent oa_ent)(u_isn u_hdip))))	36	8
2353	((p_isn ca_wa)((p_re ca_wa)((p_csn oa_ent)(oa_wa u_hdip))))	33	8
2354	((p_isn ca_wa)((p_re ca_wa)((p_csn oa_ent)(u_isn oa_wa))))	29	8
2355	((p_isn ca_wa)((p_re ca_wa)((p_csn oa_ent)(u_isn u_hdip))))	35	8
2356	((p_isn ca_wa)((p_re p_re)((p_isn p_csn)(p_isn oa_ent))))	26	8
2357	((p_isn ca_wa)((p_re (ca_wa p_csn))((oa_ent oa_wa) u_hdip)))	24	8
2358	((p_isn ca_wa)(((p_csn cp_ent)(oa_ent u_isn))(u_hdip u_hdip)))	33	8
2359	((p_isn ca_wa)(((p_csn cp_ent) oa_ent)((u_isn oa_wa) u_hdip)))	28	8
2360	((p_isn ca_wa)(((p_csn cp_ent)(oa_ent u_hdip))(u_isn u_hdip)))	31	8
2361	((p_isn ca_wa)(((p_csn cp_ent)(oa_ent u_isn))(oa_wa u_hdip)))	30	8
2362	((p_isn ca_wa)(((p_isn p_re)(p_csn cp_ent))(oa_ent u_isn)))	36	8
2363	((p_isn ca_wa)(((p_isn p_re)(p_csn cp_ent))(oa_ent oa_wa)))	30	8
2364	((p_isn ca_wa)(((p_isn p_re)(p_csn cp_ent))(oa_ent u_hdip)))	31	8
2365	((p_isn ca_wa)(((p_re ca_wa) p_csn)(oa_ent (oa_wa u_hdip))))	30	8
2366	((p_isn ca_wa)(((p_re ca_wa) p_csn)(oa_ent (u_isn u_hdip))))	37	8
2367	((p_isn ca_wa)(((p_re ca_wa) p_csn)((oa_ent oa_wa) u_hdip)))	33	8
2368	((p_isn ca_wa)(((p_re ca_wa) p_csn)((p_isn oa_ent) u_hdip)))	27	8
2369	((p_isn ca_wa)(((p_re ca_wa)(cp_ent oa_ent))(oa_wa u_hdip)))	37	8
2370	((p_isn ca_wa)(((p_re ca_wa)(cp_ent oa_ent))(u_hdip u_hdip)))	41	8
2371	((p_isn ca_wa)(((p_re ca_wa)(cp_ent oa_ent))(u_isn u_hdip)))	41	8
2372	((p_isn ca_wa)(((p_re ca_wa)(p_csn oa_ent))(oa_wa u_hdip)))	32	8
2373	((p_isn ca_wa)(((p_re ca_wa)(p_csn oa_ent))(u_hdip u_hdip)))	35	8
2374	((p_isn ca_wa)(((p_re ca_wa)(p_csn oa_ent))(u_isn u_hdip)))	37	8
2375	((p_isn oa_ent)((oa_wa u_hdip)((u_isn u_hdip)(oa_wa u_csn))))	27	8
2376	((p_isn p_re)((ca_wa (p_csn cp_ent))((oa_ent u_isn) u_hdip)))	32	8
2377	((p_isn p_re)((ca_wa (p_csn cp_ent))(oa_ent (oa_wa u_hdip))))	28	8
2378	((p_isn p_re)((ca_wa (p_csn cp_ent))(oa_ent (u_isn u_hdip))))	31	8
2379	((p_isn p_re)(((ca_wa p_csn)(oa_ent u_isn))(u_hdip u_hdip)))	30	8
2380	((p_isn p_re)(((ca_wa p_csn)(cp_ent oa_ent))(u_isn u_hdip)))	34	8
2381	((p_isn p_re)(((ca_wa p_csn)(oa_ent u_hdip))(u_isn u_hdip)))	29	8
2382	((p_re ca_wa)(((p_csn cp_ent)(oa_ent oa_wa))(u_isn u_hdip)))	29	8
2383	((p_re ca_wa)(((p_csn cp_ent)(oa_ent u_isn))(u_hdip u_hdip)))	33	8
2384	((p_re ca_wa)(((p_csn cp_ent) oa_ent)((u_isn oa_wa) u_hdip)))	28	8
2385	((p_re ca_wa)(((p_csn cp_ent)(oa_ent u_isn))(oa_wa u_hdip)))	30	8
2386	((p_re ca_wa)(((p_csn oa_ent) u_hdip)((u_isn oa_wa) u_hdip)))	30	8
2387	((p_re ca_wa)(((p_csn oa_ent)(u_isn u_hdip))(oa_wa u_hdip)))	31	8
2388	((p_re ca_wa)(((p_csn oa_ent)(u_isn u_hdip))(u_isn oa_wa)))	30	8
2389	((p_re ca_wa)(((p_csn oa_ent)(u_isn u_hdip))(u_isn u_hdip)))	36	8
2390	((u_csn (cp_ent ca_ent))((ca_wa p_re)(p_csn (oa_ent u_hdip))))	21	8
2391	((u_csn (cp_ent ca_ent))((ca_wa p_re)(p_csn (oa_ent u_isn))))	25	8
2392	((u_csn (cp_ent ca_ent))((ca_wa p_re)((p_csn oa_ent) u_hdip)))	23	8
2393	((u_csn (cp_ent ca_ent))(((ca_wa p_re) ca_wa)(cp_ent oa_ent)))	35	8
2394	((u_csn (cp_ent ca_ent))(((ca_wa p_re) ca_wa)(oa_ent u_isn)))	27	8
2395	((u_csn (cp_ent ca_ent))(((ca_wa p_re) cp_ent)(oa_ent u_isn)))	34	8
2396	((u_csn (cp_ent ca_ent))(((ca_wa p_re) cp_ent)(oa_ent oa_wa)))	27	8
2397	((u_csn (cp_ent ca_ent))(((ca_wa p_re) cp_ent)(oa_ent u_hdip)))	34	8
2398	((u_csn (cp_ent ca_ent))(((ca_wa p_re) cp_ent)(p_isn oa_ent)))	22	8
2399	(((u_csn cp_ent)(ca_ent ca_wa))(((p_re ca_wa) p_csn) oa_ent))	28	8
2400	((u_csn cp_ent)(((ca_ent ca_wa) cp_ent)(oa_ent (u_isn u_hdip))))	31	8
2401	((u_hdip oa_wa)((u_csn (cp_ent ca_ent))((ca_wa p_re) ca_wa)))	31	8
2402	((u_hdip oa_wa)(((u_csn cp_ent)(ca_ent p_isn))(p_re ca_wa)))	31	8
2403	((u_hdip oa_wa)(((u_csn cp_ent)(p_csn ca_ent))(ca_wa ca_wa)))	25	8
2404	((u_hdip oa_wa)(((u_csn cp_ent)(p_csn ca_ent))(ca_wa p_re)))	25	8
2405	((u_hdip oa_wa)(((u_csn cp_ent)(p_csn ca_ent))(p_re ca_wa)))	25	8
2406	((u_hdip oa_wa)(((u_csn cp_ent)(ca_ent ca_wa))(p_re ca_wa)))	32	8
2407	((u_hdip oa_wa)(((u_csn cp_ent)(oa_wa u_hdip))(u_isn u_hdip)))	23	8
2408	((u_hdip u_hdip)((oa_wa (u_csn cp_ent))(p_csn (ca_ent ca_wa))))	25	8
2409	((u_hdip u_hdip)((u_isn u_hdip)((u_isn u_hdip)(oa_wa u_csn))))	29	8
2410	((u_hdip u_hdip)(((u_isn oa_wa) u_csn)(p_csn (ca_ent ca_wa))))	22	8
2411	((u_hdip u_hdip)(((u_isn oa_wa) u_csn)((cp_ent ca_wa) cp_ent)))	21	8
2412	((u_isn oa_wa)((u_hdip (u_csn cp_ent))(p_csn (ca_ent ca_wa))))	24	8
2413	((u_isn oa_wa)((u_hdip (u_csn cp_ent))(p_csn (p_isn ca_ent))))	19	8
2414	((u_isn u_csn)(((u_isn cp_ent)(ca_ent ca_wa))(p_re ca_wa)))	26	8
2415	(((u_isn u_csn)((u_isn cp_ent)(ca_ent ca_wa)))(ca_wa p_csn))	21	8
2416	((u_isn u_csn)(((u_isn cp_ent) ca_ent)((ca_wa p_re) cp_ent)))	24	8
2417	((u_isn u_hdip)((u_isn u_hdip)((oa_wa u_csn)(cp_ent oa_ent))))	31	8
2418	((u_isn u_hdip)((u_isn u_hdip)((oa_wa u_csn)(oa_ent oa_wa))))	27	8
2419	((u_isn u_hdip)((u_isn u_hdip)((u_isn u_hdip)(oa_wa u_csn))))	33	8
2420	((u_isn u_hdip)(((u_isn oa_wa) u_csn)(p_csn (ca_ent ca_wa))))	23	8
2421	((u_isn u_hdip)(((u_isn oa_wa) u_csn)((cp_ent ca_wa) cp_ent)))	21	8
2422	((u_isn u_hdip)(((u_isn u_hdip)(oa_wa u_csn))(oa_ent u_isn)))	21	8
2423	((u_isn u_hdip)(((u_isn u_hdip)(oa_wa u_csn))(cp_ent oa_ent)))	32	8
2424	((oa_ent (oa_wa u_hdip))((u_isn oa_wa)((u_csn cp_ent) p_csn)))	32	8
2425	((oa_ent (oa_wa u_hdip))((u_isn u_hdip)(oa_wa (u_csn cp_ent))))	37	8
2426	((oa_ent (oa_wa u_hdip))((u_isn u_hdip)((u_isn oa_wa) u_csn)))	40	8
2427	((oa_ent (u_isn u_hdip))((u_isn u_hdip)(oa_wa (u_csn cp_ent))))	38	8
2428	((oa_ent (u_isn u_hdip))((u_isn u_hdip)((u_isn oa_wa) u_csn)))	42	8
2429	((oa_wa (u_csn (cp_ent ca_ent)))(((ca_wa p_re) cp_ent) oa_ent))	30	8
2430	((p_csn (ca_ent ca_wa))(((p_re ca_wa)((cp_ent oa_ent) oa_wa)))	28	8
2431	((p_csn (ca_ent ca_wa))(((p_re ca_wa)((cp_ent oa_ent) u_hdip)))	31	8
2432	((p_csn (ca_ent ca_wa))(((p_re ca_wa) oa_ent)(u_isn u_hdip)))	26	8
2433	((p_csn (ca_wa p_re))((ca_wa (p_csn cp_ent))(oa_ent u_isn)))	30	8
2434	((p_csn (p_isn ca_ent))(((ca_wa p_re) ca_wa)(cp_ent oa_ent)))	30	8
2435	((p_csn (p_isn ca_ent))(((ca_wa p_re) ca_wa)(oa_ent u_isn)))	25	8
2436	((p_csn (p_isn ca_ent))(((ca_wa p_re) cp_ent)(oa_ent u_isn)))	31	8
2437	((p_csn (p_isn ca_wa))((p_re ca_wa)((cp_ent oa_ent) u_hdip)))	27	8
2438	((u_csn (ca_ent ca_wa))((p_re ca_wa)(p_csn (p_isn oa_ent))))	21	8

2439	((u_csn (cp_ent ca_wa))((p_re ca_wa)((cp_ent oa_ent) oa_wa)))	24	8
2440	((u_csn (cp_ent ca_wa))((p_re ca_wa)((cp_ent oa_ent) u_hdip)))	27	8
2441	(((u_csn cp_ent)(p_csn ca_ent)))((ca_wa p_re) ca_wa) oa_ent))	22	8
2442	((u_csn (p_isn ca_ent))((ca_wa p_re)(p_csn (oa_ent u_hdip))))	18	8
2443	((u_csn (p_isn ca_ent))((ca_wa p_re)(p_csn (oa_ent u_isn))))	21	8
2444	((u_csn (p_isn ca_ent))((p_re ca_wa)((cp_ent oa_ent) oa_wa)))	21	8
2445	((u_csn (p_isn ca_ent))(((ca_wa p_re) ca_wa)(cp_ent oa_ent)))	29	8
2446	((u_csn (p_isn ca_ent)))((ca_wa p_re) ca_wa)(oa_ent u_isn)))	23	8
2447	((u_csn (p_isn ca_ent))(((ca_wa p_re) cp_ent)(oa_ent u_isn)))	28	8
2448	((u_csn (p_isn ca_ent))(((ca_wa p_re) cp_ent)(oa_ent oa_wa)))	22	8
2449	((u_csn (p_isn ca_ent)))((ca_wa p_re) cp_ent)(oa_ent u_hdip)))	28	8
2450	((u_hdip (u_csn cp_ent))((p_isn ca_ent)((p_isn ca_wa) p_csn)))	15	8
2451	((u_hdip (u_isn oa_wa))((u_csn cp_ent)(oa_ent (u_isn u_hdip))))	45	8
2452	((u_hdip (u_isn oa_wa))((u_csn cp_ent)(p_csn (ca_ent ca_wa))))	25	8
2453	((u_hdip (u_isn oa_wa))((u_csn cp_ent)(p_csn (p_isn ca_wa))))	16	8
2454	((u_hdip (u_isn oa_wa))((u_csn cp_ent)((oa_ent u_isn) u_hdip)))	47	8
2455	((u_hdip (u_isn oa_wa))((u_csn cp_ent)((p_isn oa_ent) oa_wa)))	32	8
2456	((u_hdip (u_isn oa_wa))((u_csn (oa_ent oa_wa))(u_isn u_hdip)))	25	8
2457	((u_hdip (u_isn oa_wa))((u_csn (oa_ent u_hdip))(u_isn u_hdip)))	23	8
2458	((u_hdip (u_isn oa_wa))(((u_csn cp_ent) p_csn)(ca_ent p_isn)))	28	8
2459	((u_hdip (u_isn oa_wa))(((u_csn cp_ent) p_csn)(ca_ent ca_wa)))	28	8
2460	((u_hdip (u_isn oa_wa))(((u_csn cp_ent) p_csn)(ca_wa ca_wa)))	24	8
2461	((u_hdip (u_isn oa_wa))(((u_csn cp_ent) p_csn)(p_isn ca_wa)))	17	8
2462	((u_hdip (u_isn u_csn))(((u_isn cp_ent) ca_ent)(ca_wa ca_wa)))	25	8
2463	(((ca_ent ca_wa) cp_ent)((p_csn oa_ent)((u_isn oa_wa) u_hdip)))	24	8
2464	(((ca_ent ca_wa) p_re)((p_csn cp_ent)(oa_ent (oa_wa u_hdip))))	27	8
2465	(((ca_wa p_re) ca_wa)(p_csn ((p_isn oa_ent)(u_isn u_hdip))))	29	8
2466	(((ca_wa p_re) ca_wa)((p_csn cp_ent)(p_csn (oa_ent u_isn))))	22	8
2467	(((ca_wa p_re) ca_wa)((p_csn cp_ent)((p_csn oa_ent) oa_wa)))	20	8
2468	(((ca_wa p_re) ca_wa)((p_csn (oa_ent u_hdip))(u_isn oa_wa)))	30	8
2469	(((ca_wa p_re) ca_wa)((p_csn (oa_ent u_isn))(u_hdip u_hdip)))	38	8
2470	(((ca_wa p_re) ca_wa)(((p_csn cp_ent) oa_ent)(u_hdip u_hdip)))	33	8
2471	(((ca_wa p_re) ca_wa)(((p_csn cp_ent) oa_ent)(u_isn u_hdip)))	33	8
2472	(((cp_ent ca_ent)(ca_wa p_re))((ca_wa (p_csn cp_ent)) oa_ent))	34	8
2473	(((cp_ent oa_ent) oa_wa)((u_hdip u_hdip)(oa_wa (u_csn cp_ent))))	44	8
2474	(((cp_ent oa_ent) oa_wa)((u_isn u_hdip)(oa_wa (u_csn cp_ent))))	39	8
2475	(((cp_ent oa_ent) oa_wa)((u_isn u_hdip)((u_isn oa_wa) u_csn)))	44	8
2476	(((cp_ent oa_ent) u_hdip)((u_isn u_hdip)(oa_wa (u_csn cp_ent))))	40	8
2477	(((cp_ent oa_ent) u_hdip)((u_isn u_hdip)((u_isn oa_wa) u_csn)))	44	8
2478	(((oa_ent oa_wa) u_hdip)((u_isn oa_wa)((u_csn cp_ent) p_csn)))	31	8
2479	(((oa_ent u_isn) u_hdip)((u_isn u_hdip)((oa_wa u_csn) oa_ent)))	18	8
2480	(((oa_ent u_isn) u_hdip)((u_isn u_hdip)((u_isn oa_wa) u_csn)))	44	8
2481	(((oa_wa (u_csn cp_ent)) ca_ent)(((ca_wa p_re) ca_wa) oa_ent))	25	8
2482	(((oa_wa (u_csn cp_ent)) ca_ent)(((ca_wa p_re) cp_ent) oa_ent))	31	8
2483	(((oa_wa u_csn)(p_isn ca_ent))(((ca_wa p_re) cp_ent) oa_ent))	24	8
2484	(((oa_wa u_hdip) oa_wa)((u_csn cp_ent)(p_csn (ca_ent ca_wa))))	23	8
2485	(((oa_wa u_hdip) oa_wa)((u_csn (cp_ent ca_wa))(p_re ca_wa)))	26	8
2486	(((oa_wa u_hdip) oa_wa)(((u_csn cp_ent) p_csn)(ca_ent p_isn)))	25	8
2487	(((oa_wa u_hdip) oa_wa)(((u_csn cp_ent) p_csn)(ca_ent ca_wa)))	25	8
2488	(((oa_wa u_hdip)(u_isn oa_wa))(((u_csn cp_ent) p_csn) ca_ent))	24	8
2489	(((p_csn oa_ent)(u_isn oa_wa))((u_hdip (u_isn oa_wa)) u_csn))	27	8
2490	(((p_isn ca_ent)(ca_wa p_re))((p_csn (p_isn oa_ent)) u_hdip))	27	8
2491	(((p_isn oa_ent) oa_wa)((u_hdip u_hdip)(oa_wa (u_csn cp_ent))))	31	8
2492	(((p_isn oa_ent) oa_wa)((u_isn u_hdip)(oa_wa (u_csn cp_ent))))	29	8
2493	(((p_isn oa_ent) oa_wa)((u_isn u_hdip)((u_isn oa_wa) u_csn)))	31	8
2494	(((u_csn cp_ent)(oa_ent oa_wa))((u_hdip (u_isn oa_wa)) u_csn))	33	8
2495	(((u_csn cp_ent)(p_isn ca_ent))(((ca_wa p_re) ca_wa) oa_ent))	25	8
2496	(((u_csn cp_ent)(p_isn ca_ent))(((ca_wa p_re) cp_ent) oa_ent))	29	8
2497	(((u_csn cp_ent)(p_isn ca_ent))(((p_re ca_wa) p_csn) oa_ent))	24	8
2498	(((u_hdip oa_wa) u_csn)((cp_ent ca_ent)((ca_wa p_re) cp_ent)))	30	8
2499	(((u_hdip u_csn)(cp_ent ca_ent))(((ca_wa p_re) ca_wa) oa_ent))	23	8
2500	(((u_hdip u_csn)(cp_ent ca_ent))(((ca_wa p_re) cp_ent) oa_ent))	26	8
2501	(((u_isn cp_ent) ca_ent)(((ca_wa p_re) ca_wa)(cp_ent oa_ent)))	24	8
2502	(((u_isn cp_ent) ca_ent)(((ca_wa p_re) cp_ent)(oa_ent u_isn)))	23	8
2503	(((u_isn cp_ent) ca_ent)(((ca_wa p_re) cp_ent)(oa_ent u_hdip)))	23	8
2504	(((u_isn cp_ent)(p_isn ca_ent))(((ca_wa p_re) cp_ent) oa_ent))	21	8
2505	(((u_isn oa_wa) u_hdip)((u_csn cp_ent)(p_csn (ca_ent ca_wa))))	25	8
2506	(((u_isn oa_wa) u_hdip)((u_isn u_hdip)((oa_wa u_csn) oa_ent)))	19	8
2507	(((u_isn oa_wa) u_hdip)((u_csn (oa_ent oa_wa))(u_isn u_hdip)))	18	8
2508	(((u_isn oa_wa) u_hdip)((u_csn (oa_ent u_hdip))(u_isn u_hdip)))	17	8
2509	(((u_isn oa_wa) u_hdip)((u_csn (oa_ent u_isn))(u_hdip u_hdip)))	17	8
2510	(((u_isn oa_wa) u_hdip)(((u_csn cp_ent) p_csn)(ca_ent p_isn)))	29	8
2511	(((u_isn oa_wa) u_hdip)(((u_csn cp_ent) p_csn)(ca_ent ca_wa)))	29	8
2512	(((u_isn oa_wa) u_hdip)(((u_csn cp_ent) p_csn)(ca_wa ca_wa)))	23	8
2513	(((u_isn oa_wa) u_hdip)(((u_csn cp_ent) p_csn)(p_isn ca_ent)))	18	8
2514	(((u_isn oa_wa) u_hdip)(((u_csn cp_ent) p_csn)(p_isn ca_wa)))	18	8
2515	(((u_isn oa_wa) u_hdip)(((u_isn oa_wa) u_csn)(ca_ent ca_wa)))	25	8
2516	(((u_isn oa_wa) u_hdip)(((u_isn oa_wa) u_csn)(cp_ent ca_ent)))	25	8
2517	(((u_isn oa_wa) u_hdip)(((u_isn oa_wa) u_csn)(cp_ent ca_wa)))	21	8
2518	(((u_isn u_csn)(cp_ent ca_ent))(((ca_wa p_re) cp_ent) oa_ent))	24	8
2519	(((ca_ent ca_wa)((p_isn ca_wa) p_csn))(oa_ent (u_isn u_hdip)))	32	8
2520	(((ca_ent ca_wa)((p_isn ca_wa) p_csn))((p_isn oa_ent) u_hdip))	23	8
2521	(((ca_ent ca_wa)((p_isn ca_wa)(p_csn cp_ent)))(oa_ent oa_wa))	32	8
2522	(((ca_ent ca_wa)((p_isn ca_wa)(p_csn cp_ent)))(oa_ent u_isn))	37	8
2523	(((ca_ent ca_wa)((p_re ca_wa) p_csn))(oa_ent (oa_wa u_hdip)))	31	8
2524	(((ca_ent ca_wa)((p_re ca_wa) p_csn))(oa_ent (u_isn u_hdip)))	38	8
2525	(((ca_ent ca_wa)((p_re ca_wa) p_csn))((oa_ent oa_wa) u_hdip))	34	8
2526	(((ca_ent ca_wa)((p_re ca_wa) p_csn))((p_isn oa_ent) u_hdip))	29	8
2527	(((ca_ent ca_wa)((p_re ca_wa)(cp_ent oa_ent)))(u_isn oa_wa))	41	8
2528	(((ca_ent p_isn)((p_re ca_wa)(cp_ent oa_ent)))(u_isn oa_wa))	41	8
2529	(((ca_ent ca_wa)((p_re ca_wa)(p_csn cp_ent)))(oa_ent u_isn))	39	8
2530	(((ca_ent ca_wa)((p_re ca_wa)(p_csn cp_ent)))(p_csn oa_ent))	22	8
2531	(((ca_wa p_re)(ca_wa (p_csn cp_ent)))((oa_ent u_isn) u_hdip))	34	8
2532	(((ca_ent p_isn)(ca_wa (p_csn cp_ent)))(oa_ent (oa_wa u_hdip)))	28	8
2533	(((ca_ent p_isn)(ca_wa (p_csn cp_ent)))(p_csn (oa_ent u_isn)))	22	8
2534	(((ca_ent p_isn)(ca_wa (p_csn cp_ent)))((p_csn oa_ent) oa_wa))	21	8
2535	(((ca_ent p_isn)((p_re ca_wa) p_csn))(oa_ent (oa_wa u_hdip)))	33	8
2536	(((ca_ent p_isn)((p_re ca_wa)(p_csn cp_ent)))(oa_ent oa_wa))	33	8
2537	(((ca_ent p_isn)((p_re ca_wa)(p_csn cp_ent)))(oa_ent u_isn))	38	8
2538	(((ca_ent p_isn)((p_re ca_wa)(p_csn cp_ent)))(p_csn oa_ent))	21	8
2539	(((ca_ent p_re)(ca_wa (p_csn cp_ent)))(p_csn (oa_ent u_isn)))	21	8
2540	(((ca_wa p_re)((ca_wa p_csn)(p_isn cp_ent)))(oa_ent u_isn))	23	8
2541	((((ca_ent ca_wa) p_re)(p_csn cp_ent)) oa_ent)(u_hdip u_hdip))	33	8
2542	(((ca_wa p_re)((p_csn oa_ent) u_hdip))((u_isn oa_wa) u_hdip))	25	8
2543	(((ca_wa p_re)((p_csn oa_ent)(oa_wa u_hdip)))(u_isn u_hdip))	24	8
2544	(((ca_wa p_re)((p_csn oa_ent)(u_isn u_hdip)))(u_isn u_hdip))	33	8
2545	(((cp_ent ca_ent)((ca_wa ca_wa)(cp_ent oa_ent)))(oa_wa u_hdip))	24	8
2546	(((cp_ent ca_ent)((ca_wa ca_wa)(cp_ent oa_ent)))(u_hdip u_hdip))	31	8
2547	(((cp_ent ca_ent)((ca_wa ca_wa)(cp_ent oa_ent)))(u_isn u_hdip))	27	8
2548	(((cp_ent ca_ent)((ca_wa ca_wa)(p_csn oa_ent)))(oa_wa u_hdip))	21	8
2549	(((cp_ent ca_ent)((ca_wa ca_wa)(p_csn oa_ent)))(u_hdip u_hdip))	31	8
2550	(((cp_ent ca_ent)((ca_wa ca_wa)(p_csn oa_ent)))(u_isn u_hdip))	31	8
2551	(((cp_ent ca_ent)((ca_wa ca_wa)(p_isn cp_ent)))(oa_ent u_isn))	25	8
2552	(((cp_ent ca_ent)((ca_wa p_re) cp_ent))(oa_ent (oa_wa u_hdip)))	39	8
2553	(((cp_ent ca_ent)((ca_wa p_re) cp_ent))(oa_ent (u_isn u_hdip)))	40	8
2554	(((cp_ent ca_ent)((ca_wa p_re) cp_ent))((oa_ent oa_wa) u_hdip))	40	8
2555	(((cp_ent ca_ent)((ca_wa p_re) cp_ent))((oa_ent u_isn) u_hdip))	42	8
2556	(((cp_ent ca_ent)((ca_wa p_re) cp_ent))((p_isn oa_ent) u_hdip))	28	8
2557	(((cp_ent ca_ent)((ca_wa p_re)(p_csn cp_ent)))(oa_ent oa_wa))	30	8
2558	(((cp_ent ca_ent)((ca_wa p_re)(p_csn cp_ent)))(oa_ent u_isn))	37	8
2559	(((cp_ent ca_ent)((ca_wa p_re)(p_csn oa_ent)))(oa_wa u_hdip))	30	8
2560	(((cp_ent ca_ent)((ca_wa p_re)(p_csn oa_ent)))(u_hdip u_hdip))	33	8
2561	(((cp_ent ca_ent)((ca_wa p_re)(p_csn oa_ent)))(u_isn u_hdip))	34	8
2562	(((cp_ent ca_ent)((ca_wa p_re)(p_isn cp_ent)))(oa_ent u_isn))	27	8
2563	(((cp_ent ca_ent)((p_re ca_wa)(p_csn oa_ent)))(oa_wa u_hdip))	33	8
2564	(((cp_ent ca_wa)((p_re ca_wa)(p_csn cp_ent)))(oa_ent oa_wa))	29	8
2565	(((cp_ent ca_wa)((p_re ca_wa)(p_csn cp_ent)))(oa_ent u_hdip))	30	8
2566	(((cp_ent ca_wa)((p_re ca_wa)(p_csn cp_ent)))(oa_ent u_isn))	35	8
2567	(((cp_ent ca_wa)((p_re ca_wa)(p_csn oa_ent)))(u_hdip u_hdip))	29	8
2568	((((cp_ent oa_ent) oa_wa)(u_hdip (oa_wa u_csn)))(u_isn cp_ent))	30	8
2569	((((cp_ent oa_ent) oa_wa)((u_hdip oa_wa) u_csn))(u_isn cp_ent))	33	8
2570	((((cp_ent oa_ent) oa_wa)((u_isn oa_wa) u_csn))(oa_ent u_hdip))	19	8
2571	((((cp_ent oa_ent) oa_wa)((u_isn oa_wa) u_csn))(u_isn cp_ent))	33	8
2572	(((oa_ent oa_wa)((u_isn oa_wa) u_csn))((oa_ent u_isn) u_hdip))	19	8
2573	(((oa_ent oa_wa)((u_isn u_hdip)(oa_wa u_csn)))(u_isn cp_ent))	30	8
2574	(((oa_wa oa_wa)(u_csn (cp_ent ca_ent)))(((ca_wa p_re) cp_ent))	23	8
2575	(((oa_wa oa_wa)((u_csn cp_ent)(ca_ent ca_wa)))(p_isn p_re))	25	8
2576	(((oa_wa oa_wa)((u_csn cp_ent)(ca_ent ca_wa)))(p_re ca_wa))	25	8
2577	(((oa_wa oa_wa)((u_csn cp_ent)(p_isn ca_wa)))(p_re ca_wa))	22	8
2578	(((oa_wa (u_csn cp_ent))(p_csn (ca_ent ca_wa)))(p_re ca_wa))	31	8
2579	(((oa_wa (u_csn cp_ent))(((ca_ent ca_wa) p_re))(ca_wa p_csn))	28	8
2580	(((oa_wa u_csn)((cp_ent ca_wa) cp_ent))(oa_ent (oa_wa u_hdip)))	24	8
2581	(((oa_wa u_csn)((cp_ent ca_wa) cp_ent))(oa_ent (u_isn u_hdip)))	26	8
2582	(((oa_wa u_hdip)(oa_wa (u_csn cp_ent)))(p_csn (ca_ent ca_wa)))	22	8
2583	(((oa_wa u_hdip)(oa_wa (u_csn cp_ent)))((oa_wa u_hdip) oa_wa))	21	8
2584	(((oa_wa u_hdip)((u_isn u_hdip)(oa_wa u_csn)))(cp_ent oa_ent))	27	8
2585	(((oa_wa u_hdip)((u_isn u_hdip)(oa_wa u_csn)))(oa_ent oa_wa))	25	8
2586	(((oa_wa u_hdip)((u_isn u_hdip)(oa_wa u_csn)))(oa_ent u_hdip))	18	8
2587	(((oa_wa u_hdip)((u_isn u_hdip)(oa_wa u_csn)))(oa_ent u_isn))	20	8
2588	(((p_csn ca_ent)((p_re ca_wa)(cp_ent oa_ent)))(u_hdip u_hdip))	26	8
2589	(((ca_wa (p_csn cp_ent))(oa_ent (u_isn u_hdip)))(u_isn u_hdip))	30	8
2590	(((ca_wa (p_csn cp_ent))((oa_ent u_isn) u_hdip))(u_isn oa_wa))	22	8
2591	(((ca_wa (p_csn cp_ent))((oa_ent u_isn) u_hdip))(u_isn u_hdip))	31	8
2592	(((p_csn oa_ent)((u_isn oa_wa) u_hdip))(oa_wa (u_csn cp_ent)))	27	8
2593	(((p_csn oa_ent)((u_isn oa_wa) u_hdip))((u_csn cp_ent) p_csn))	29	8
2594	(((p_isn ca_ent)((ca_wa ca_wa)(cp_ent oa_ent)))(u_isn u_hdip))	22	8
2595	(((p_isn ca_ent)((ca_wa p_re)(p_csn cp_ent)))(oa_ent u_isn))	30	8
2596	(((p_isn ca_ent)((ca_wa p_re)(p_csn oa_ent)))(u_isn u_hdip))	30	8
2597	(((p_isn ca_ent)((p_isn ca_wa)(p_csn cp_ent)))(oa_ent u_isn))	26	8
2598	(((p_isn ca_ent)((p_re ca_wa) p_csn))(oa_ent (oa_wa u_hdip)))	25	8
2599	(((p_isn ca_ent)((p_re ca_wa) p_csn))(oa_ent (u_isn u_hdip)))	31	8
2600	(((p_isn ca_ent)((p_re ca_wa) p_csn))((oa_ent oa_wa) u_hdip))	26	8
2601	(((p_isn ca_ent)((p_re ca_wa) p_csn))((p_isn oa_ent) u_hdip))	28	8
2602	(((p_isn ca_ent)((p_re ca_wa)(cp_ent oa_ent)))(u_hdip u_hdip))	36	8
2603	(((p_isn ca_ent)((p_re ca_wa)(cp_ent oa_ent)))(u_isn u_hdip))	36	8
2604	(((p_isn ca_wa)((p_csn cp_ent)(oa_ent u_hdip)))(u_isn u_hdip))	32	8
2605	(((p_isn ca_wa)((p_re ca_wa)(p_csn oa_ent)))(oa_wa u_hdip))	29	8
2606	(((p_isn p_re)(ca_wa (p_csn cp_ent)))(oa_ent (u_isn u_hdip)))	32	8
2607	(((p_isn p_re)(ca_wa (p_csn cp_ent)))(p_csn (oa_ent u_isn)))	22	8
2608	(((p_isn p_re)(ca_wa (p_csn cp_ent)))((oa_ent u_isn) u_hdip))	33	8
2609	(((p_isn p_re)(ca_wa (p_csn cp_ent)))((p_csn oa_ent) oa_wa))	20	8
2610	(((p_isn p_re)((ca_wa p_csn)(p_isn cp_ent)))(oa_ent u_isn))	22	8
2611	((((ca_wa p_re) ca_wa)(p_csn (oa_ent oa_wa)))(u_hdip u_hdip))	29	8
2612	((((ca_wa p_re) ca_wa)(p_csn (oa_ent oa_wa)))(u_isn u_hdip))	33	8
2613	((((ca_wa p_re) ca_wa)(p_csn (oa_ent u_hdip)))(u_isn u_hdip))	35	8
2614	((((ca_wa p_re) ca_wa)(p_csn (oa_ent u_isn)))(oa_wa u_hdip))	35	8
2615	((((ca_wa p_re) ca_wa)(p_csn (oa_ent u_isn)))(u_hdip u_hdip))	39	8
2616	((((ca_wa p_re) ca_wa)(p_csn (p_isn oa_ent)))(u_hdip u_hdip))	27	8
2617	((((ca_wa p_re) ca_wa)(p_csn (p_isn oa_ent)))(u_isn u_hdip))	30	8
2618	((((ca_wa p_re) ca_wa)((cp_ent oa_ent) u_hdip))(u_isn oa_wa))	39	8
2619	((((ca_wa p_re) ca_wa)((cp_ent oa_ent) u_hdip))(u_isn u_hdip))	41	8
2620	(((p_re ca_wa)((cp_ent oa_ent)(oa_wa u_hdip)))(u_isn u_hdip))	34	8
2621	(((p_re ca_wa)((cp_ent oa_ent)(u_isn u_hdip)))(u_isn oa_wa))	33	8
2622	((((ca_wa p_re) ca_wa)((p_csn oa_ent) u_hdip))(oa_wa u_hdip))	32	8
2623	((((ca_wa p_re) ca_wa)((p_csn oa_ent) u_hdip))(u_isn u_hdip))	37	8
2624	(((p_re ca_wa)((p_csn oa_ent) u_hdip))((u_isn oa_wa) u_hdip))	31	8
2625	(((p_re ca_wa)((p_csn oa_ent)(oa_wa u_hdip)))(u_isn u_hdip))	28	8
2626	(((u_csn (cp_ent ca_ent))((ca_wa p_re) ca_wa))(cp_ent oa_ent))	36	8
2627	(((u_csn cp_ent)(p_csn (p_isn ca_ent)))((ca_wa p_re) ca_wa))	26	8
2628	(((u_csn cp_ent)((ca_ent ca_wa) p_csn))(oa_ent (u_isn u_hdip)))	31	8
2629	(((u_csn cp_ent)((ca_ent ca_wa) p_csn))((oa_ent u_isn) u_hdip))	32	8
2630	(((u_csn cp_ent)((p_isn ca_ent)(ca_wa p_re)))(p_isn ca_wa))	26	8
2631	(((u_hdip oa_wa)(u_csn (cp_ent ca_ent)))((ca_wa p_re) cp_ent))	30	8
2632	(((u_hdip oa_wa)((u_csn cp_ent) p_csn))((ca_ent ca_wa) cp_ent))	26	8
2633	(((u_hdip oa_wa)((u_csn cp_ent)(ca_ent ca_wa)))(ca_wa p_csn))	24	8
2634	(((u_hdip u_csn)((cp_ent oa_ent)(u_isn u_hdip)))(u_isn u_hdip))	41	8
2635	(((u_hdip u_hdip)(oa_wa (u_csn cp_ent)))(oa_ent (oa_wa u_hdip)))	42	8
2636	(((u_hdip u_hdip)(oa_wa (u_csn cp_ent)))(p_csn (ca_ent ca_wa)))	24	8
2637	(((u_hdip u_hdip)((u_csn cp_ent)(p_csn ca_ent)))(ca_wa ca_wa))	22	8
2638	(((u_hdip u_hdip)((oa_wa u_csn)(cp_ent oa_ent)))(u_isn u_hdip))	28	8
2639	(((u_isn cp_ent)((ca_wa p_re) ca_wa))((cp_ent oa_ent) u_hdip))	24	8
2640	(((u_isn cp_ent)((p_isn ca_ent)(ca_wa p_re)))(ca_wa p_csn))	22	8
2641	(((u_isn oa_wa)((u_csn cp_ent) p_csn))((ca_ent ca_wa) cp_ent))	27	8
2642	(((u_isn u_hdip)((oa_wa u_csn)(oa_ent oa_wa)))(u_isn u_hdip))	26	8
2643	(((u_isn u_hdip)((u_isn oa_wa) u_csn))(p_csn (ca_ent ca_wa)))	24	8
2644	(((u_isn u_hdip)((u_isn u_hdip)(oa_wa u_csn)))(cp_ent oa_ent))	34	8
2645	(((u_isn u_hdip)((u_isn u_hdip)(oa_wa u_csn)))(oa_ent oa_wa))	31	8
2646	(((u_isn u_hdip)((u_isn u_hdip)(oa_wa u_csn)))(oa_ent u_isn))	33	8
2647	((((ca_ent p_isn)(p_re (ca_wa p_csn))) oa_ent)(u_hdip u_hdip))	31	8
2648	(((p_re (ca_wa p_csn))((oa_ent oa_wa) u_hdip))(u_isn u_hdip))	25	8
2649	(((oa_ent (oa_wa u_hdip))((u_isn oa_wa) u_csn))(cp_ent oa_ent))	38	8
2650	(((oa_ent (oa_wa u_hdip))((u_isn oa_wa) u_csn))(oa_ent u_isn))	21	8
2651	(((oa_ent (oa_wa u_hdip))((u_isn oa_wa) u_csn))(u_isn cp_ent))	35	8
2652	(((oa_ent (u_isn u_hdip))((u_isn oa_wa) u_csn))(cp_ent oa_ent))	41	8
2653	(((oa_ent (u_isn u_hdip))((u_isn oa_wa) u_csn))(u_isn cp_ent))	36	8
2654	(((oa_ent (u_isn u_hdip))((u_isn u_hdip)(oa_wa u_csn))) oa_ent)	20	8
2655	(((oa_wa (u_csn cp_ent)) p_csn)((p_isn ca_ent)(ca_wa p_re)))	28	8
2656	(((oa_wa (u_csn cp_ent))(p_isn ca_ent))((ca_wa p_re) ca_wa))	31	8
2657	(((oa_wa (u_csn cp_ent))(p_isn ca_ent))((p_re ca_wa) p_csn))	25	8
2658	(((oa_wa (u_csn cp_ent))(p_isn ca_wa))((p_re ca_wa) p_csn))	24	8
2659	(((oa_wa (u_csn cp_ent))(p_csn (p_isn ca_ent)))(ca_wa ca_wa))	24	8
2660	(((oa_wa (u_csn cp_ent))(p_csn (p_isn ca_wa)))(p_re ca_wa))	21	8
2661	(((p_csn (ca_ent ca_wa))(p_re (ca_wa p_csn)))(cp_ent oa_ent))	27	8
2662	(((p_csn (ca_ent ca_wa))((p_re ca_wa) p_csn))(cp_ent oa_ent))	29	8
2663	(((p_csn (ca_ent ca_wa))((p_re ca_wa) p_csn))(oa_ent oa_wa))	27	8
2664	(((p_csn (ca_ent ca_wa))((p_re ca_wa) p_csn))(oa_ent u_hdip))	30	8
2665	(((p_csn (ca_ent ca_wa))((p_re ca_wa) p_csn))(oa_ent u_isn))	37	8
2666	(((p_csn (ca_ent ca_wa))((p_re ca_wa) p_csn))(p_isn oa_ent))	26	8
2667	(((p_csn (ca_wa p_re))(ca_wa p_csn))((p_isn oa_ent) u_hdip))	19	8
2668	(((p_csn (ca_wa p_re))(ca_wa (p_csn cp_ent)))(oa_ent u_hdip))	25	8
2669	(((p_csn (oa_ent u_hdip))(u_isn oa_wa))((u_csn cp_ent) p_csn))	33	8
2670	(((p_csn (oa_ent u_isn))(u_hdip (u_isn oa_wa)))(u_isn u_hdip))	33	8
2671	(((p_csn (p_isn oa_ent))(oa_wa u_hdip))((u_csn cp_ent) p_csn))	28	8
2672	((((u_csn cp_ent)(ca_ent ca_wa))(p_isn ca_wa))(cp_ent oa_ent))	34	8
2673	(((u_csn (ca_ent ca_wa))(p_isn ca_wa))(p_csn (oa_ent u_hdip)))	26	8
2674	(((u_csn (ca_ent ca_wa))(p_isn ca_wa))(p_csn (oa_ent u_isn)))	28	8
2675	(((u_csn (ca_ent ca_wa))(p_isn ca_wa))((cp_ent oa_ent) u_hdip))	35	8
2676	(((u_csn (ca_ent ca_wa))(p_isn ca_wa))((oa_ent u_isn) u_hdip))	35	8
2677	(((u_csn (ca_ent ca_wa))(p_re p_re))((p_csn oa_ent) u_hdip))	25	8
2678	(((u_csn (ca_ent ca_wa))(p_re p_re))((p_isn oa_ent) u_hdip))	22	8
2679	(((u_csn (cp_ent ca_wa))(p_isn ca_wa))((cp_ent oa_ent) u_hdip))	28	8
2680	(((u_csn (cp_ent ca_wa))(p_isn ca_wa))(oa_ent (oa_wa u_hdip)))	29	8
2681	(((u_csn (cp_ent ca_wa))(p_isn ca_wa))((cp_ent oa_ent) oa_wa))	27	8
2682	(((u_csn (cp_ent ca_wa))(p_isn ca_wa))((oa_ent u_isn) u_hdip))	28	8
2683	(((u_csn (cp_ent ca_wa))(p_re ca_wa))((cp_ent oa_ent) u_hdip))	30	8
2684	(((u_csn (p_isn ca_ent))(p_isn ca_wa))((cp_ent oa_ent) u_hdip))	25	8
2685	(((u_csn (p_isn ca_ent))((p_re ca_wa) oa_ent))(u_hdip u_hdip))	20	8
2686	((((u_csn (u_isn cp_ent)) ca_ent)(p_isn p_re))(ca_wa p_csn))	24	8
2687	(((u_csn (u_isn cp_ent))(ca_ent ca_wa))((p_re ca_wa) p_csn))	26	8
2688	(((u_csn (u_isn cp_ent))(p_isn ca_ent))((ca_wa p_re) ca_wa))	26	8
2689	(((u_csn (u_isn cp_ent))(p_isn ca_ent))((ca_wa p_re) cp_ent))	25	8
2690	(((u_hdip (oa_wa u_csn))(cp_ent ca_ent))((ca_wa p_re) ca_wa))	29	8
2691	(((u_hdip (oa_wa u_csn))(cp_ent ca_ent))((ca_wa p_re) cp_ent))	28	8
2692	(((u_hdip (oa_wa u_csn))(p_csn (ca_ent ca_wa)))(p_re ca_wa))	23	8
2693	(((u_hdip (u_csn cp_ent))(ca_ent ca_wa))((p_isn ca_wa) p_csn))	20	8
2694	(((u_hdip (u_csn cp_ent))(p_csn ca_ent))((ca_wa p_re) ca_wa))	25	8
2695	(((u_hdip (u_csn cp_ent))(p_csn ca_ent))((ca_wa p_re) cp_ent))	25	8
2696	(((u_hdip (u_csn cp_ent))(p_isn ca_ent))((ca_wa p_re) cp_ent))	26	8
2697	(((u_hdip (u_csn cp_ent))(p_isn ca_ent))((p_isn ca_wa) p_csn))	17	8
2698	(((u_hdip (u_csn cp_ent))(oa_ent (u_isn u_hdip)))(u_isn u_hdip))	46	8
2699	(((u_hdip (u_csn cp_ent))(p_csn (p_isn ca_ent)))(ca_wa ca_wa))	20	8
2700	(((u_hdip (u_csn cp_ent))((oa_ent u_isn) u_hdip))(u_isn u_hdip))	52	8
2701	(((u_hdip (u_isn oa_wa)) u_csn)((cp_ent ca_ent)(ca_wa p_re)))	30	8
2702	(((u_hdip (u_isn oa_wa)) u_csn)((cp_ent oa_ent)(u_isn u_hdip)))	44	8
2703	(((u_hdip (u_isn oa_wa))(u_csn (cp_ent ca_ent)))(ca_wa ca_wa))	30	8
2704	(((u_hdip (u_isn oa_wa))(u_csn (cp_ent ca_ent)))(ca_wa p_csn))	23	8
2705	(((u_hdip (u_isn oa_wa))(u_csn (cp_ent ca_ent)))(ca_wa p_re))	30	8
2706	(((u_hdip (u_isn oa_wa))(u_csn (cp_ent ca_ent)))(p_isn ca_wa))	22	8
2707	(((u_hdip (u_isn oa_wa))(u_csn (cp_ent oa_ent)))(oa_wa u_hdip))	28	8
2708	(((u_hdip (u_isn oa_wa))(u_csn (cp_ent oa_ent)))(u_isn u_hdip))	25	8
2709	(((u_hdip (u_isn oa_wa))(u_csn (oa_ent oa_wa)))(u_hdip u_hdip))	23	8
2710	(((u_hdip (u_isn oa_wa))(u_csn (oa_ent u_hdip)))(u_isn u_hdip))	25	8
2711	(((u_hdip (u_isn oa_wa))((u_csn cp_ent) p_csn))(ca_wa ca_wa))	18	8
2712	(((u_hdip (u_isn u_csn))(cp_ent ca_ent))((ca_wa p_re) ca_wa))	31	8
2713	(((u_hdip (u_isn u_csn))(cp_ent ca_ent))((ca_wa p_re) cp_ent))	28	8
2714	((((ca_ent ca_wa) cp_ent) oa_ent)((oa_wa u_hdip)(u_isn oa_wa)))	30	8
2715	((((ca_ent ca_wa) cp_ent)(oa_ent u_hdip))((u_isn oa_wa) u_hdip))	35	8
2716	((((ca_ent ca_wa) cp_ent)(oa_ent u_isn))(u_hdip (oa_wa u_hdip)))	27	8
2717	((((ca_ent ca_wa) cp_ent)(oa_ent (oa_wa u_hdip)))(u_isn u_hdip))	35	8
2718	((((ca_ent ca_wa) cp_ent)(oa_ent (u_isn u_hdip)))(oa_wa u_hdip))	34	8
2719	((((ca_ent ca_wa) cp_ent)(oa_ent (u_isn u_hdip)))(u_isn oa_wa))	39	8
2720	((((ca_ent ca_wa) cp_ent)(oa_ent (u_isn u_hdip)))(u_isn u_hdip))	42	8
2721	((((ca_ent ca_wa) cp_ent)(p_csn (oa_ent oa_wa)))(u_isn u_hdip))	22	8
2722	((((ca_ent ca_wa) cp_ent)((oa_ent oa_wa) u_hdip))(u_isn u_hdip))	36	8
2723	((((ca_ent ca_wa) cp_ent)((oa_ent u_isn) u_hdip))(oa_wa u_hdip))	36	8
2724	((((ca_ent ca_wa) cp_ent)((oa_ent u_isn) u_hdip))(u_isn oa_wa))	41	8
2725	((((ca_ent ca_wa) cp_ent)((oa_ent u_isn) u_hdip))(u_isn u_hdip))	44	8
2726	((((ca_ent ca_wa) cp_ent)((p_csn oa_ent) oa_wa))(u_isn u_hdip))	28	8
2727	((((ca_ent ca_wa) cp_ent)((p_isn oa_ent) u_hdip))(u_isn u_hdip))	28	8
2728	((((ca_ent ca_wa) p_csn) oa_ent)((oa_wa u_hdip)(u_isn oa_wa)))	19	8
2729	((((ca_ent ca_wa) p_csn)(oa_ent (oa_wa u_hdip)))(u_isn u_hdip))	27	8
2730	((((ca_ent ca_wa) p_csn)((oa_ent u_isn) u_hdip))(u_isn u_hdip))	33	8
2731	((((ca_ent ca_wa) p_re)(p_csn cp_ent))(p_csn (oa_ent u_isn)))	22	8
2732	((((ca_ent ca_wa) p_re)(p_csn oa_ent))((oa_wa u_hdip) oa_wa))	26	8
2733	((((ca_ent ca_wa) p_re)(p_isn p_csn))((p_isn cp_ent) p_csn))	37	8
2734	((((ca_ent ca_wa) p_re)(p_csn (oa_ent u_isn)))(u_hdip u_hdip))	33	8
2735	((((p_isn ca_wa) p_csn)(oa_ent (oa_wa u_hdip)))(u_isn u_hdip))	24	8
2736	((((ca_ent p_isn)(p_csn cp_ent)) oa_ent)((u_isn oa_wa) u_hdip))	25	8
2737	((((ca_ent p_isn)(p_csn cp_ent))(oa_ent u_isn))(oa_wa u_hdip))	27	8
2738	((((ca_ent p_re) p_csn) oa_ent)((oa_wa u_hdip)(u_isn oa_wa)))	23	8
2739	((((ca_ent p_re)(cp_ent oa_ent))(oa_wa u_hdip))(u_isn oa_wa))	27	8
2740	((((ca_ent p_re)(cp_ent oa_ent))(oa_wa u_hdip))(u_isn u_hdip))	27	8
2741	((((ca_ent p_re)(cp_ent oa_ent))(u_hdip u_hdip))(u_isn oa_wa))	27	8
2742	((((ca_ent p_re)(cp_ent oa_ent))(u_hdip u_hdip))(u_isn u_hdip))	31	8
2743	((((p_re ca_wa)(p_csn cp_ent))(oa_ent oa_wa))(u_isn oa_wa))	27	8
2744	((((ca_ent p_re)(p_csn cp_ent))(oa_ent oa_wa))(u_isn u_hdip))	30	8
2745	((((p_re ca_wa)(p_csn cp_ent))(oa_ent oa_wa))(u_isn u_hdip))	30	8
2746	((((ca_wa ca_wa)(cp_ent oa_ent))(oa_wa u_hdip))(u_isn u_hdip))	24	8
2747	((((ca_wa ca_wa)(cp_ent oa_ent))(u_hdip u_hdip))(u_isn oa_wa))	30	8
2748	((((ca_wa ca_wa)(cp_ent oa_ent))(u_hdip u_hdip))(u_isn u_hdip))	33	8
2749	((((ca_wa ca_wa)(cp_ent oa_ent))(u_isn u_hdip))(u_isn oa_wa))	32	8
2750	((((ca_wa ca_wa)(cp_ent oa_ent))(u_isn u_hdip))(u_isn u_hdip))	33	8
2751	((((ca_wa ca_wa)(p_csn oa_ent))(oa_wa u_hdip))(u_isn u_hdip))	26	8
2752	((((ca_wa ca_wa)(p_csn oa_ent))(u_hdip u_hdip))(u_isn u_hdip))	32	8
2753	((((ca_wa ca_wa)(p_csn oa_ent))(u_isn u_hdip))(u_isn u_hdip))	35	8
2754	((((ca_wa p_csn)(cp_ent oa_ent))(u_isn u_hdip))(u_isn u_hdip))	31	8
2755	((((ca_wa p_re) ca_wa) oa_ent)((oa_wa u_hdip)(u_isn oa_wa)))	26	8
2756	((((ca_wa p_re) ca_wa)(cp_ent oa_ent))((u_hdip oa_wa) u_hdip))	29	8
2757	((((ca_wa p_re) ca_wa)(cp_ent oa_ent))((u_isn oa_wa) u_hdip))	39	8
2758	((((ca_wa p_re) ca_wa)((cp_ent oa_ent) oa_wa))(u_hdip u_hdip))	35	8
2759	((((ca_wa p_re) ca_wa)((cp_ent oa_ent) oa_wa))(u_isn oa_wa))	37	8
2760	((((ca_wa p_re) ca_wa)((cp_ent oa_ent) oa_wa))(u_isn u_hdip))	40	8
2761	((((ca_wa p_re) ca_wa)((p_csn oa_ent) oa_wa))(u_hdip u_hdip))	30	8
2762	((((ca_wa p_re) ca_wa)((p_csn oa_ent) oa_wa))(u_isn u_hdip))	36	8
2763	((((ca_wa p_re) cp_ent) oa_ent)((oa_wa u_hdip)(u_isn oa_wa)))	33	8
2764	((((ca_wa p_re) cp_ent)(oa_ent u_hdip))((u_isn oa_wa) u_hdip))	36	8
2765	((((ca_wa p_re) cp_ent)(oa_ent (oa_wa u_hdip)))(u_isn u_hdip))	33	8
2766	((((ca_wa p_re) cp_ent)(oa_ent (u_isn u_hdip)))(u_isn oa_wa))	37	8
2767	((((ca_wa p_re) cp_ent)(oa_ent (u_isn u_hdip)))(u_isn u_hdip))	41	8
2768	((((ca_wa p_re) cp_ent)((oa_ent oa_wa) u_hdip))(u_isn u_hdip))	34	8
2769	((((ca_wa p_re) cp_ent)((oa_ent u_isn) u_hdip))(u_isn oa_wa))	39	8
2770	((((ca_wa p_re) cp_ent)((oa_ent u_isn) u_hdip))(u_isn u_hdip))	43	8
2771	((((ca_wa p_re) cp_ent)((p_csn oa_ent) oa_wa))(u_isn u_hdip))	25	8
2772	((((ca_wa p_re) cp_ent)((p_isn oa_ent) u_hdip))(u_isn u_hdip))	25	8
2773	((((ca_wa p_re)(p_csn oa_ent))(oa_wa u_hdip))(u_isn u_hdip))	25	8
2774	((((ca_wa p_re)(p_csn oa_ent))(u_isn u_hdip))(oa_wa u_hdip))	28	8
2775	((((ca_wa p_re)(p_csn oa_ent))(u_isn u_hdip))(u_isn u_hdip))	33	8
2776	((((cp_ent ca_ent) cp_ent) oa_ent)((oa_wa u_hdip)(u_isn oa_wa)))	34	8
2777	((((cp_ent ca_ent) cp_ent)(oa_ent u_hdip))((u_isn oa_wa) u_hdip))	33	8
2778	((((cp_ent ca_ent) cp_ent)(oa_ent u_isn))((u_hdip oa_wa) u_hdip))	28	8
2779	((((cp_ent ca_ent) cp_ent)(oa_ent (oa_wa u_hdip)))(u_isn u_hdip))	33	8
2780	((((cp_ent ca_ent) cp_ent)(oa_ent (u_isn u_hdip)))(oa_wa u_hdip))	32	8
2781	((((cp_ent ca_ent) cp_ent)(oa_ent (u_isn u_hdip)))(u_isn oa_wa))	37	8
2782	((((cp_ent ca_ent) cp_ent)(oa_ent (u_isn u_hdip)))(u_isn u_hdip))	40	8
2783	((((cp_ent ca_ent) cp_ent)((oa_ent oa_wa) u_hdip))(u_isn u_hdip))	34	8
2784	((((cp_ent ca_ent) cp_ent)((oa_ent u_isn) u_hdip))(oa_wa u_hdip))	34	8
2785	((((cp_ent ca_ent) cp_ent)((oa_ent u_isn) u_hdip))(u_isn oa_wa))	39	8
2786	((((cp_ent ca_ent) cp_ent)((oa_ent u_isn) u_hdip))(u_isn u_hdip))	42	8
2787	((((cp_ent ca_ent) cp_ent)((p_isn oa_ent) u_hdip))(u_isn u_hdip))	28	8
2788	((((cp_ent ca_ent)(ca_wa p_re))(cp_ent oa_ent))(oa_wa u_hdip))	34	8
2789	((((cp_ent ca_ent)(ca_wa p_re))(cp_ent oa_ent))(u_hdip u_hdip))	36	8
2790	((((cp_ent ca_ent)(ca_wa p_re))(p_csn oa_ent))(u_hdip u_hdip))	32	8
2791	((((cp_ent ca_ent)(ca_wa p_re))(p_csn oa_ent))(u_isn u_hdip))	33	8
2792	((((cp_ent ca_ent)(ca_wa p_re))(ca_wa (p_csn cp_ent))) oa_ent)	37	8
2793	((((cp_ent ca_wa) cp_ent) oa_ent)((oa_wa u_hdip)(u_isn oa_wa)))	33	8
2794	((((cp_ent ca_wa) cp_ent)(oa_ent u_hdip))((u_isn oa_wa) u_hdip))	33	8
2795	((((cp_ent ca_wa) cp_ent)(oa_ent (oa_wa u_hdip)))(u_isn u_hdip))	32	8
2796	((((cp_ent ca_wa) cp_ent)(oa_ent (u_isn u_hdip)))(u_isn oa_wa))	36	8
2797	((((cp_ent ca_wa) cp_ent)(oa_ent (u_isn u_hdip)))(u_isn u_hdip))	39	8
2798	((((cp_ent ca_wa) cp_ent)((oa_ent oa_wa) u_hdip))(u_isn u_hdip))	33	8
2799	((((cp_ent ca_wa) cp_ent)((oa_ent u_isn) u_hdip))(oa_wa u_hdip))	34	8
2800	((((cp_ent ca_wa) cp_ent)((oa_ent u_isn) u_hdip))(u_isn oa_wa))	38	8
2801	((((cp_ent ca_wa) cp_ent)((oa_ent u_isn) u_hdip))(u_isn u_hdip))	41	8
2802	((((cp_ent ca_wa) cp_ent)((p_isn oa_ent) u_hdip))(u_isn u_hdip))	27	8
2803	((((cp_ent oa_ent) u_hdip)((u_isn oa_wa) u_csn))(u_isn cp_ent))	38	8
2804	((((cp_ent oa_ent)(oa_wa u_hdip))(oa_wa u_csn))(u_isn cp_ent))	40	8
2805	((((cp_ent oa_ent)(oa_wa u_hdip))((u_isn oa_wa) u_csn)) oa_ent)	21	8
2806	((((cp_ent oa_ent)(u_isn u_hdip))(oa_wa u_csn))(u_isn cp_ent))	39	8
2807	(((((oa_ent oa_wa) u_csn)(u_isn cp_ent)) ca_ent)(p_isn p_re))	21	8
2808	((((oa_ent oa_wa) u_csn)(u_isn cp_ent))((ca_ent ca_wa) p_re))	21	8
2809	((((oa_ent oa_wa) u_hdip)((u_isn oa_wa) u_csn))(cp_ent oa_ent))	20	8
2810	((((oa_ent oa_wa) u_hdip)((u_isn oa_wa) u_csn))(oa_ent oa_wa))	20	8
2811	((((oa_ent oa_wa) u_hdip)((u_isn oa_wa) u_csn))(oa_ent u_isn))	20	8
2812	((((oa_ent u_isn) u_hdip)((u_isn oa_wa) u_csn))(cp_ent oa_ent))	43	8
2813	((((oa_ent u_isn) u_hdip)((u_isn oa_wa) u_csn))(u_isn cp_ent))	38	8
2814	((((oa_wa u_csn)(u_isn cp_ent))(p_isn p_re))(ca_wa p_csn))	20	8
2815	((((oa_wa u_hdip) oa_wa)(u_csn (cp_ent ca_ent)))(ca_wa ca_wa))	29	8
2816	((((oa_wa u_hdip) oa_wa)(u_csn (cp_ent ca_ent)))(ca_wa p_re))	29	8
2817	((((oa_wa u_hdip) oa_wa)(u_csn (cp_ent ca_ent)))(p_isn ca_wa))	24	8
2818	((((oa_wa u_hdip) oa_wa)((u_csn cp_ent) p_csn))(ca_wa ca_wa))	18	8
2819	((((oa_wa u_hdip)(oa_wa u_csn))(cp_ent ca_ent))(ca_wa ca_wa))	25	8
2820	((((p_csn ca_ent)(ca_wa p_re))(ca_wa p_csn))(cp_ent oa_ent))	28	8
2821	((((p_csn ca_ent)(p_isn p_re))(ca_wa p_csn))(p_isn p_csn))	48	8
2822	((((p_csn oa_ent)(u_hdip oa_wa))(u_isn u_hdip))(u_isn u_hdip))	27	8
2823	((((p_csn oa_ent)(u_isn u_hdip))(u_isn oa_wa))(u_csn cp_ent))	36	8
2824	((((p_csn oa_ent)(u_isn u_hdip))(u_isn u_hdip))(u_isn oa_wa))	43	8
2825	((((p_csn oa_ent)(u_isn u_hdip))(u_isn u_hdip))(u_isn u_hdip))	41	8
2826	((((p_isn ca_ent)(ca_wa p_re))(cp_ent oa_ent))(u_hdip u_hdip))	33	8
2827	((((p_isn ca_ent)(ca_wa p_re))(p_csn oa_ent))(u_hdip u_hdip))	28	8
2828	((((p_isn ca_ent)(ca_wa p_re))(p_csn oa_ent))(u_isn u_hdip))	27	8
2829	((((p_isn ca_wa) p_csn)(oa_ent (u_isn u_hdip)))(u_isn u_hdip))	29	8
2830	((((p_isn ca_wa) p_csn)((p_isn oa_ent) u_hdip))(u_isn u_hdip))	21	8
2831	((((p_isn p_re)(p_csn cp_ent)) oa_ent)((u_isn oa_wa) u_hdip))	27	8
2832	((((p_isn p_re)(p_csn cp_ent))(oa_ent oa_wa))(u_isn oa_wa))	26	8
2833	((((p_isn p_re)(p_csn cp_ent))(oa_ent oa_wa))(u_isn u_hdip))	28	8
2834	((((p_isn p_re)(p_csn cp_ent))(oa_ent u_hdip))(u_isn u_hdip))	30	8
2835	((((p_isn p_re)(p_csn cp_ent))(oa_ent u_isn))(oa_wa u_hdip))	29	8
2836	((((p_re ca_wa) p_csn) oa_ent)((oa_wa u_hdip)(u_isn oa_wa)))	26	8
2837	((((p_re ca_wa) p_csn)(cp_ent oa_ent))((u_isn oa_wa) u_hdip))	28	8
2838	((((p_re ca_wa) p_csn)(oa_ent (oa_wa u_hdip)))(u_isn u_hdip))	30	8
2839	((((p_re ca_wa) p_csn)(oa_ent (u_isn u_hdip)))(u_isn u_hdip))	35	8
2840	((((p_re ca_wa) p_csn)((oa_ent oa_wa) u_hdip))(u_isn u_hdip))	29	8
2841	((((p_re ca_wa) p_csn)((p_isn oa_ent) u_hdip))(u_isn u_hdip))	26	8
2842	((((p_re ca_wa)(cp_ent oa_ent))(oa_wa u_hdip))(u_isn oa_wa))	32	8
2843	((((p_re ca_wa)(cp_ent oa_ent))(oa_wa u_hdip))(u_isn u_hdip))	35	8
2844	((((p_re ca_wa)(cp_ent oa_ent))(u_hdip u_hdip))(u_isn oa_wa))	35	8
2845	((((p_re ca_wa)(cp_ent oa_ent))(u_hdip u_hdip))(u_isn u_hdip))	39	8

2846	((((p_re ca_wa)(cp_ent oa_ent))(u_isn u_hdip))(u_isn oa_wa))	37	8
2847	((((p_re ca_wa)(cp_ent oa_ent))(u_isn u_hdip))(u_isn u_hdip))	39	8
2848	((((p_re ca_wa)(p_csn cp_ent))(oa_ent u_hdip))(u_isn u_hdip))	32	8
2849	((((p_re ca_wa)(p_csn oa_ent))(oa_wa u_hdip))(u_isn u_hdip))	27	8
2850	((((p_re ca_wa)(p_csn oa_ent))(u_hdip u_hdip))(u_isn u_hdip))	33	8
2851	((((p_re ca_wa)(p_csn oa_ent))(u_isn u_hdip))(oa_wa u_hdip))	32	8
2852	((((p_re ca_wa)(p_csn oa_ent))(u_isn u_hdip))(u_isn oa_wa))	34	8
2853	((((p_re ca_wa)(p_csn oa_ent))(u_isn u_hdip))(u_isn u_hdip))	37	8
2854	((((u_csn cp_ent) p_csn)(ca_ent ca_wa)))((p_re ca_wa) oa_ent))	22	8
2855	((((u_csn cp_ent) p_csn) ca_ent))((ca_wa p_re) ca_wa) oa_ent))	23	8
2856	((((u_csn cp_ent) p_csn)(p_isn ca_ent)))((ca_wa p_re) ca_wa))	29	8
2857	((((u_csn cp_ent)(p_isn ca_ent))(ca_wa p_re))(p_isn ca_wa))	27	8
2858	((((u_csn cp_ent)(p_isn ca_wa))(p_re ca_wa))(cp_ent oa_ent))	29	8
2859	((((u_hdip oa_wa) u_csn)(cp_ent ca_ent)))((ca_wa p_re) ca_wa))	32	8
2860	((((u_hdip oa_wa) u_csn)(cp_ent ca_ent)))((ca_wa p_re) cp_ent))	31	8
2861	((((u_hdip oa_wa) u_csn)(ca_ent ca_wa)))((p_re ca_wa) oa_ent))	21	8
2862	((((u_hdip oa_wa) u_csn)(ca_ent ca_wa)))((p_re ca_wa) p_csn))	27	8
2863	((((u_hdip oa_wa) u_csn)(cp_ent ca_wa)))((p_re ca_wa) p_csn))	23	8
2864	((((u_hdip oa_wa) u_csn)((cp_ent ca_wa) cp_ent))(oa_ent oa_wa))	20	8
2865	((((u_hdip oa_wa) u_csn)((cp_ent ca_wa) cp_ent))(oa_ent u_hdip))	21	8
2866	((((u_hdip oa_wa) u_csn)((cp_ent ca_wa) cp_ent))(oa_ent u_isn))	21	8
2867	((((u_hdip u_csn)(cp_ent oa_ent))(oa_wa u_hdip))(u_isn u_hdip))	30	8
2868	((((u_hdip u_csn)(u_isn cp_ent))(oa_ent u_hdip))(u_isn u_hdip))	31	8
2869	((((u_isn cp_ent)(ca_ent ca_wa))(p_re ca_wa))(p_csn cp_ent))	22	8
2870	((((u_isn cp_ent) ca_ent)((ca_wa p_re) ca_wa))(p_csn cp_ent))	23	8
2871	((((u_isn cp_ent)(p_isn ca_wa))(p_isn p_re))(ca_wa p_csn))	17	8
2872	((((u_isn oa_wa) u_csn)(cp_ent ca_ent)))((ca_wa p_re) ca_wa))	31	8
2873	((((u_isn oa_wa) u_csn)(ca_ent ca_wa)))((p_re ca_wa) oa_ent))	20	8
2874	((((u_isn oa_wa) u_csn)(cp_ent ca_ent)))((ca_wa p_re) cp_ent))	30	8
2875	((((u_isn oa_wa) u_csn)(p_csn (ca_ent ca_wa)))(p_re ca_wa))	26	8
2876	((((u_isn oa_wa) u_hdip)(oa_wa u_csn))(oa_ent (u_isn u_hdip)))	21	8
2877	((((u_isn oa_wa) u_hdip)(oa_wa u_csn)))((cp_ent ca_wa) cp_ent))	22	8
2878	((((u_isn oa_wa) u_hdip)(u_csn (cp_ent ca_ent)))(ca_wa ca_wa))	25	8
2879	((((u_isn oa_wa) u_hdip)(u_csn (cp_ent ca_ent)))(ca_wa p_csn))	21	8
2880	((((u_isn oa_wa) u_hdip)(u_csn (cp_ent oa_ent)))(oa_wa u_hdip))	20	8
2881	((((u_isn oa_wa) u_hdip)(u_csn (cp_ent oa_ent)))(u_isn u_hdip))	18	8
2882	((((u_isn oa_wa) u_hdip)(u_isn u_hdip)(oa_wa u_csn))) oa_ent)	17	8
2883	((((u_isn u_csn)(u_isn cp_ent)) ca_ent)(ca_wa p_re) cp_ent))	25	8
2884	((((u_isn u_csn)(u_isn cp_ent))(ca_ent ca_wa))(ca_wa p_csn))	23	8
2885	((((u_isn u_hdip)(oa_wa u_csn))(cp_ent ca_wa))(ca_wa p_csn))	18	8
2886	(p_csn ((ca_ent ca_wa)(p_re ca_wa)((cp_ent oa_ent) u_hdip))))	30	8
2887	(p_csn ((ca_ent ca_wa)))(p_re ca_wa) oa_ent)(u_isn u_hdip)))	25	8
2888	(p_csn ((ca_ent p_isn)(((p_re ca_wa) oa_ent)(u_isn u_hdip)))	24	8
2889	(p_csn ((p_isn ca_ent)((p_re ca_wa)((cp_ent oa_ent) u_hdip)))	26	8
2890	(p_csn ((p_isn ca_ent)(((ca_wa p_re) ca_wa)(cp_ent oa_ent)))	31	8
2891	(p_csn ((p_isn ca_ent)(((ca_wa p_re) ca_wa)(oa_ent u_isn)))	26	8
2892	(p_csn ((p_isn ca_ent)(((ca_wa p_re) cp_ent)(oa_ent u_isn)))	31	8
2893	(p_csn ((p_isn ca_ent)(((ca_wa p_re) cp_ent)(oa_ent u_hdip)))	26	8
2894	(p_csn ((p_isn ca_ent)(((p_re ca_wa) oa_ent)(u_isn u_hdip)))	20	8
2895	(p_csn (((ca_ent ca_wa)(p_re ca_wa) oa_ent))(u_isn u_hdip)))	26	8
2896	(p_csn ((((ca_ent ca_wa) cp_ent)(oa_ent u_hdip))(u_isn u_hdip)))	28	8
2897	(p_csn ((((ca_ent ca_wa) p_re)(cp_ent oa_ent))(u_hdip u_hdip)))	26	8
2898	(p_csn ((((ca_ent ca_wa) p_re)(cp_ent oa_ent))(u_isn u_hdip)))	20	8
2899	((ca_ent ca_wa)((p_re ca_wa)(p_csn (oa_ent (oa_wa u_hdip)))))	31	8
2900	((ca_ent ca_wa)((p_re ca_wa)(p_csn (oa_ent (u_isn u_hdip)))))	36	8
2901	((ca_ent ca_wa)((p_re ca_wa)(p_csn ((oa_ent oa_wa) u_hdip))))	34	8
2902	((ca_ent ca_wa)((p_re ca_wa)(p_csn ((p_isn oa_ent) u_hdip))))	26	8
2903	((ca_ent ca_wa)(((p_re (ca_wa p_csn)) oa_ent)(u_isn u_hdip)))	33	8
2904	((ca_ent ca_wa)((((p_re ca_wa) p_csn) oa_ent)(oa_wa u_hdip)))	37	8
2905	((ca_ent ca_wa)((((p_re ca_wa) p_csn) oa_ent)(u_hdip u_hdip)))	40	8
2906	((ca_ent ca_wa)((((p_re ca_wa) p_csn) oa_ent)(u_isn u_hdip)))	42	8
2907	((ca_ent p_isn)((p_re ca_wa)(p_csn (oa_ent (oa_wa u_hdip)))))	27	8
2908	((ca_ent p_isn)((p_re ca_wa)(p_csn (oa_ent (u_isn u_hdip)))))	35	8
2909	((ca_ent p_isn)((p_re ca_wa)(p_csn ((oa_ent oa_wa) u_hdip))))	32	8
2910	((ca_ent p_isn)((p_re ca_wa)(p_csn ((p_isn oa_ent) u_hdip))))	24	8
2911	((ca_ent p_isn)(((ca_wa (p_csn cp_ent)) oa_ent)(u_hdip u_hdip)))	29	8
2912	((ca_ent p_isn)(((ca_wa (p_csn cp_ent)) oa_ent)(u_isn u_hdip)))	29	8
2913	((ca_ent p_isn)(((p_re (ca_wa p_csn)) oa_ent)(u_isn u_hdip)))	32	8
2914	((ca_ent p_isn)((((p_re ca_wa) p_csn) oa_ent)(oa_wa u_hdip)))	36	8
2915	((ca_ent p_isn)((((p_re ca_wa) p_csn) oa_ent)(u_hdip u_hdip)))	39	8
2916	((ca_wa ca_wa)((p_re p_re)((ca_wa (p_csn cp_ent)) oa_ent)))	25	8
2917	((ca_wa ca_wa)((p_csn (oa_ent (u_isn u_hdip)))(u_isn u_hdip)))	32	8
2918	((ca_wa p_re)((p_csn (oa_ent (u_isn u_hdip)))(u_isn u_hdip)))	27	8
2919	((ca_wa p_re)(((p_csn (oa_ent u_isn)) u_hdip)(oa_wa u_hdip)))	26	8
2920	((cp_ent ca_ent)((p_isn p_re)((ca_wa (p_csn cp_ent)) oa_ent)))	34	8
2921	((cp_ent ca_ent)((p_re ca_wa)(p_csn (oa_ent (oa_wa u_hdip)))))	25	8
2922	((cp_ent ca_ent)(((p_re (ca_wa p_csn)) oa_ent)(u_isn u_hdip)))	30	8
2923	((cp_ent ca_ent)((((ca_wa p_re) ca_wa) oa_ent)(u_hdip u_hdip)))	35	8
2924	((cp_ent ca_ent)((((ca_wa p_re) ca_wa) oa_ent)(u_isn u_hdip)))	35	8
2925	((cp_ent ca_ent)((((ca_wa p_re) cp_ent) oa_ent)(oa_wa oa_wa)))	39	8
2926	((cp_ent ca_ent)((((ca_wa p_re) cp_ent) oa_ent)(oa_wa u_hdip)))	39	8
2927	((cp_ent ca_ent)((((ca_wa p_re) cp_ent) oa_ent)(u_hdip u_hdip)))	40	8
2928	((cp_ent ca_ent)((((ca_wa p_re) cp_ent) oa_ent)(u_isn oa_wa)))	36	8
2929	((cp_ent ca_ent)((((ca_wa p_re) cp_ent) oa_ent)(u_isn u_hdip)))	43	8
2930	((cp_ent ca_ent)((((p_re ca_wa) p_csn) oa_ent)(oa_wa u_hdip)))	31	8
2931	((cp_ent ca_ent)((((p_re ca_wa) p_csn) oa_ent)(u_hdip u_hdip)))	37	8
2932	((cp_ent ca_ent)((((p_re ca_wa) p_csn) oa_ent)(u_isn u_hdip)))	39	8
2933	((cp_ent ca_wa)((p_re ca_wa)(p_csn (oa_ent (u_isn u_hdip)))))	32	8
2934	((cp_ent ca_wa)((p_re ca_wa)(p_csn ((p_isn oa_ent) u_hdip))))	22	8
2935	((cp_ent ca_wa)(((p_re (ca_wa p_csn)) oa_ent)(u_isn u_hdip)))	29	8
2936	((cp_ent ca_wa)((((p_re ca_wa) p_csn) oa_ent)(oa_wa u_hdip)))	32	8
2937	((cp_ent ca_wa)((((p_re ca_wa) p_csn) oa_ent)(u_hdip u_hdip)))	35	8
2938	((cp_ent ca_wa)((((p_re ca_wa) p_csn) oa_ent)(u_isn u_hdip)))	37	8
2939	((oa_ent oa_wa)((u_hdip u_hdip)((oa_wa (u_csn cp_ent)) oa_ent)))	34	8
2940	((oa_ent oa_wa)(((u_isn u_hdip)(oa_wa (u_csn cp_ent))) oa_ent))	24	8
2941	((oa_ent u_hdip)(((u_isn u_hdip)(oa_wa (u_csn cp_ent))) oa_ent))	30	8
2942	((oa_wa oa_wa)((((u_csn cp_ent) p_csn) ca_ent)(ca_wa ca_wa)))	22	8
2943	((oa_wa oa_wa)((((u_csn cp_ent) p_csn) ca_ent)(ca_wa p_re)))	22	8
2944	((oa_wa u_hdip)((u_isn u_hdip)((oa_wa (u_csn cp_ent)) oa_ent)))	21	8
2945	((oa_wa u_hdip)((u_isn u_hdip)(((oa_wa u_csn) oa_ent) oa_wa)))	18	8
2946	((oa_wa u_hdip)(((u_isn u_hdip)(oa_wa (u_csn cp_ent))) oa_ent))	28	8
2947	((oa_wa u_hdip)((((u_csn cp_ent) p_csn) ca_ent)(ca_wa ca_wa)))	26	8
2948	((oa_wa u_hdip)((((u_csn cp_ent) p_csn) ca_ent)(ca_wa p_re)))	26	8
2949	((p_csn ca_ent)((((ca_wa p_re) ca_wa) oa_ent)(u_hdip u_hdip)))	27	8
2950	((p_csn ca_ent)((((ca_wa p_re) ca_wa) oa_ent)(u_isn u_hdip)))	26	8
2951	((p_csn ca_ent)((((ca_wa p_re) cp_ent) oa_ent)(u_hdip u_hdip)))	30	8
2952	((p_csn ca_ent)((((ca_wa p_re) cp_ent) oa_ent)(u_isn u_hdip)))	33	8
2953	((p_isn ca_ent)((ca_wa p_re)(p_csn (oa_ent (u_isn u_hdip)))))	24	8
2954	((p_isn ca_ent)((ca_wa p_re)((p_csn (oa_ent u_isn)) u_hdip)))	27	8
2955	((p_isn ca_ent)((ca_wa p_re)((p_csn (p_isn oa_ent)) u_hdip)))	24	8
2956	((p_isn ca_ent)((p_isn p_re)((ca_wa (p_csn cp_ent)) p_csn)))	29	8
2957	((p_isn ca_ent)((p_re ca_wa)(p_csn (oa_ent (oa_wa u_hdip)))))	20	8
2958	((p_isn ca_ent)((p_re ca_wa)(p_csn ((oa_ent oa_wa) u_hdip))))	21	8
2959	((p_isn ca_ent)(((p_re (ca_wa p_csn)) oa_ent)(u_isn u_hdip)))	26	8
2960	((p_isn ca_ent)((((ca_wa p_re) ca_wa) oa_ent)(oa_wa u_hdip)))	27	8
2961	((p_isn ca_ent)((((ca_wa p_re) ca_wa) oa_ent)(u_hdip u_hdip)))	32	8
2962	((p_isn ca_ent)((((ca_wa p_re) ca_wa) oa_ent)(u_isn u_hdip)))	32	8
2963	((p_isn ca_ent)((((ca_wa p_re) cp_ent) oa_ent)(oa_wa oa_wa)))	34	8
2964	((p_isn ca_ent)((((ca_wa p_re) cp_ent) oa_ent)(oa_wa u_hdip)))	34	8
2965	((p_isn ca_ent)((((ca_wa p_re) cp_ent) oa_ent)(u_isn oa_wa)))	33	8
2966	((p_isn ca_ent)((((ca_wa p_re) cp_ent) oa_ent)(u_isn u_hdip)))	38	8
2967	((p_isn ca_ent)((((p_re ca_wa) p_csn) oa_ent)(oa_wa u_hdip)))	25	8
2968	((p_isn ca_ent)((((p_re ca_wa) p_csn) oa_ent)(u_hdip u_hdip)))	32	8
2969	((p_isn ca_ent)((((p_re ca_wa) p_csn) oa_ent)(u_isn u_hdip)))	34	8
2970	((p_isn ca_wa)((p_re ca_wa)(p_csn (oa_ent (oa_wa u_hdip)))))	29	8
2971	((p_isn ca_wa)((p_re ca_wa)(p_csn (oa_ent (u_isn u_hdip)))))	34	8
2972	((p_isn ca_wa)((p_re ca_wa)(p_csn ((oa_ent oa_wa) u_hdip))))	32	8
2973	((p_isn ca_wa)((p_re ca_wa)(p_csn ((p_isn oa_ent) u_hdip))))	25	8
2974	((p_isn ca_wa)(((p_re (ca_wa p_csn)) oa_ent)(u_isn u_hdip)))	31	8
2975	((p_isn ca_wa)((((p_re ca_wa) p_csn) oa_ent)(oa_wa u_hdip)))	33	8
2976	((p_isn ca_wa)((((p_re ca_wa) p_csn) oa_ent)(u_hdip u_hdip)))	36	8
2977	((p_isn ca_wa)((((p_re ca_wa) p_csn) oa_ent)(u_isn u_hdip)))	38	8
2978	((p_isn p_re)(((ca_wa (p_csn cp_ent)) oa_ent)(u_hdip u_hdip)))	32	8
2979	((p_re ca_wa)((p_csn (oa_ent (u_isn u_hdip)))(u_isn oa_wa)))	29	8
2980	((p_re ca_wa)((p_csn (oa_ent (u_isn u_hdip)))(u_isn u_hdip)))	33	8
2981	((p_re ca_wa)((p_csn ((oa_ent u_isn) u_hdip))(u_isn oa_wa)))	28	8
2982	((p_re ca_wa)(((p_csn (oa_ent u_isn)) u_hdip)(oa_wa u_hdip)))	30	8
2983	((p_re ca_wa)(((p_csn (oa_ent u_isn)) u_hdip)(u_isn oa_wa)))	29	8
2984	((u_hdip oa_wa)((((u_csn cp_ent) p_csn) ca_ent)(ca_wa ca_wa)))	27	8
2985	((u_hdip oa_wa)((((u_csn cp_ent) p_csn) ca_ent)(ca_wa p_re)))	27	8
2986	((u_hdip u_hdip)(((u_isn oa_wa)((u_csn cp_ent) p_csn)) ca_ent))	26	8
2987	((u_hdip u_hdip)(((oa_wa (u_csn cp_ent)) p_csn)(ca_ent p_isn)))	28	8
2988	((u_hdip u_hdip)(((oa_wa (u_csn cp_ent)) p_csn)(ca_ent ca_wa)))	28	8
2989	((u_hdip u_hdip)(((oa_wa (u_csn cp_ent)) p_csn)(p_isn ca_ent)))	22	8
2990	((u_isn u_hdip)((u_isn u_hdip)((oa_wa (u_csn cp_ent)) oa_ent)))	25	8
2991	((u_isn u_hdip)((u_isn u_hdip)(((oa_wa u_csn) oa_ent) oa_wa)))	21	8
2992	((u_isn u_hdip)(((u_isn oa_wa)((u_csn cp_ent) p_csn)) ca_ent))	26	8
2993	((u_isn u_hdip)(((u_isn u_hdip)(oa_wa (u_csn cp_ent))) oa_ent))	33	8
2994	((oa_ent ((oa_wa u_hdip)((u_isn u_hdip)(oa_wa u_csn)))) oa_ent)	18	8
2995	((p_csn ((ca_wa p_re)(ca_wa (p_csn cp_ent))))(oa_ent u_isn))	24	8
2996	(((((ca_ent ca_wa)(((p_re ca_wa) p_csn) oa_ent))(u_isn oa_wa))	34	8
2997	(((((ca_ent p_isn)(((p_re ca_wa) p_csn) oa_ent))(u_isn oa_wa))	34	8
2998	((((ca_wa (p_csn cp_ent)) oa_ent)(u_isn u_hdip))(u_isn oa_wa))	22	8
2999	((((ca_wa (p_csn cp_ent)) oa_ent)(u_isn u_hdip))(u_isn u_hdip))	31	8
3000	(((ca_wa p_re)((p_csn (oa_ent u_isn)) u_hdip))(oa_wa u_hdip))	25	8
3001	(((ca_wa p_re)((p_csn (oa_ent u_isn)) u_hdip))(u_isn u_hdip))	30	8
3002	(((ca_wa p_re)((p_csn (p_isn oa_ent)) u_hdip))(u_isn u_hdip))	23	8
3003	((((p_re (ca_wa p_csn)) oa_ent)(u_hdip u_hdip))(u_isn u_hdip))	28	8
3004	(((((p_isn ca_wa) p_csn) oa_ent)(u_hdip u_hdip))(u_isn u_hdip))	28	8
3005	(((((p_isn ca_wa) p_csn) oa_ent)(u_isn u_hdip))(u_isn u_hdip))	30	8
3006	(((cp_ent ca_ent)((p_re (ca_wa p_csn)) oa_ent))(u_hdip u_hdip))	29	8
3007	(((cp_ent ca_ent)((p_re (ca_wa p_csn)) oa_ent))(u_isn u_hdip))	31	8
3008	(((cp_ent ca_ent)(((ca_wa p_re) ca_wa) oa_ent))(oa_wa u_hdip))	32	8
3009	(((cp_ent ca_ent)(((ca_wa p_re) ca_wa) oa_ent))(u_hdip u_hdip))	36	8
3010	(((cp_ent ca_ent)(((ca_wa p_re) ca_wa) oa_ent))(u_isn u_hdip))	36	8
3011	(((cp_ent ca_ent)(((ca_wa p_re) ca_wa)(p_isn cp_ent))) oa_ent)	27	8
3012	(((cp_ent ca_ent)(((ca_wa p_re) cp_ent) oa_ent))(oa_wa oa_wa))	40	8
3013	(((cp_ent ca_ent)(((ca_wa p_re) cp_ent) oa_ent))(u_isn oa_wa))	35	8
3014	(((cp_ent ca_ent)(((p_re ca_wa) p_csn) oa_ent))(oa_wa oa_wa))	31	8
3015	(((cp_ent ca_ent)(((p_re ca_wa) p_csn) oa_ent))(oa_wa u_hdip))	34	8
3016	(((cp_ent ca_wa)(((p_re ca_wa) p_csn) oa_ent))(oa_wa oa_wa))	30	8
3017	(((cp_ent ca_wa)(((p_re ca_wa) p_csn) oa_ent))(u_hdip u_hdip))	32	8
3018	(((oa_ent oa_wa)(u_isn u_hdip)((u_isn oa_wa) u_csn))) oa_ent)	21	8
3019	(((oa_wa u_hdip)((u_isn oa_wa)((u_csn cp_ent) p_csn))) ca_ent)	17	8
3020	(((p_csn ca_ent)(((ca_wa p_re) ca_wa) oa_ent))(u_hdip u_hdip))	24	8
3021	(((p_csn ca_ent)(((ca_wa p_re) ca_wa) oa_ent))(u_isn u_hdip))	21	8
3022	(((p_isn ca_ent)(((ca_wa p_re) ca_wa) oa_ent))(oa_wa u_hdip))	22	8
3023	(((p_isn ca_ent)(((ca_wa p_re) cp_ent) oa_ent))(oa_wa oa_wa))	35	8
3024	(((p_isn ca_ent)(((ca_wa p_re) cp_ent) oa_ent))(oa_wa u_hdip))	34	8
3025	(((p_isn ca_ent)(((ca_wa p_re) cp_ent) oa_ent))(u_hdip u_hdip))	36	8
3026	(((p_isn ca_ent)(((p_re ca_wa) p_csn) oa_ent))(u_hdip u_hdip))	29	8
3027	(((p_re ca_wa)(p_csn (oa_ent (oa_wa u_hdip))))(u_isn u_hdip))	29	8
3028	(((p_re ca_wa)(p_csn ((oa_ent oa_wa) u_hdip)))(u_isn u_hdip))	28	8
3029	(((p_re ca_wa)(p_csn ((p_isn oa_ent) u_hdip)))(u_isn u_hdip))	25	8
3030	(((u_hdip u_hdip)((u_isn oa_wa)((u_csn cp_ent) p_csn))) ca_ent)	28	8
3031	(((u_hdip u_hdip)(((oa_wa u_csn) oa_ent) oa_wa))(u_hdip u_hdip))	20	8
3032	(((u_hdip u_hdip)(((oa_wa u_csn) oa_ent) oa_wa))(u_isn u_hdip))	22	8
3033	(((u_isn u_hdip)((u_isn oa_wa)((u_csn cp_ent) p_csn))) ca_ent)	26	8
3034	(((u_isn u_hdip)((oa_wa (u_csn cp_ent)) oa_ent))(oa_wa u_hdip))	26	8
3035	(((u_isn u_hdip)((oa_wa (u_csn cp_ent)) oa_ent))(u_isn oa_wa))	23	8
3036	(((u_isn u_hdip)((oa_wa (u_csn cp_ent)) oa_ent))(u_isn u_hdip))	24	8
3037	(((u_isn u_hdip)(((oa_wa u_csn) oa_ent) oa_wa))(u_isn u_hdip))	18	8
3038	(((u_isn oa_wa)((u_csn cp_ent) p_csn)) ca_ent)(ca_wa ca_wa))	28	8
3039	(((u_isn oa_wa)((u_csn cp_ent) p_csn)) ca_ent)(ca_wa p_re))	28	8
3040	(((p_csn (oa_ent (u_isn u_hdip)))(u_isn oa_wa))(u_csn cp_ent))	35	8
3041	(((p_csn (oa_ent (u_isn u_hdip)))(u_isn u_hdip))(u_isn oa_wa))	42	8
3042	(((p_csn (oa_ent (u_isn u_hdip)))(u_isn u_hdip))(u_isn u_hdip))	40	8
3043	(((p_csn ((oa_ent u_isn) u_hdip))(u_isn oa_wa))(u_csn cp_ent))	36	8
3044	(((p_csn ((oa_ent u_isn) u_hdip))(u_isn oa_wa))(u_isn u_hdip))	33	8
3045	((((ca_ent ca_wa)((p_isn ca_wa) p_csn)) oa_ent)(oa_wa u_hdip))	26	8
3046	((((p_re ca_wa) oa_ent)(oa_wa u_hdip))(u_isn oa_wa)) u_csn)	24	8
3047	((((ca_ent ca_wa)((p_re ca_wa) p_csn)) oa_ent)(oa_wa u_hdip))	36	8
3048	((((ca_ent ca_wa)((p_re ca_wa) p_csn)) oa_ent)(u_hdip u_hdip))	39	8
3049	((((ca_ent ca_wa)((p_re ca_wa) p_csn)) oa_ent)(u_isn u_hdip))	41	8
3050	((((ca_ent p_isn)((p_re ca_wa) p_csn)) oa_ent)(u_isn u_hdip))	43	8
3051	((((cp_ent ca_ent)((ca_wa p_re) cp_ent)) oa_ent)(oa_wa oa_wa))	41	8
3052	((((cp_ent ca_ent)((ca_wa p_re) cp_ent)) oa_ent)(oa_wa u_hdip))	40	8
3053	((((cp_ent ca_ent)((ca_wa p_re) cp_ent)) oa_ent)(u_hdip u_hdip))	41	8
3054	((((cp_ent ca_ent)((ca_wa p_re) cp_ent)) oa_ent)(u_isn oa_wa))	36	8
3055	((((oa_wa u_csn)((cp_ent ca_wa) cp_ent)) oa_ent)(oa_wa oa_wa))	23	8
3056	((((oa_wa u_csn)((cp_ent ca_wa) cp_ent)) oa_ent)(oa_wa u_hdip))	26	8
3057	((((oa_wa u_csn)((cp_ent ca_wa) cp_ent)) oa_ent)(u_hdip oa_wa))	24	8
3058	((((p_csn oa_ent)((u_isn oa_wa) u_hdip))(u_isn oa_wa)) u_csn)	27	8
3059	((((p_isn ca_ent)((p_re ca_wa) p_csn)) oa_ent)(u_hdip u_hdip))	31	8
3060	((((p_isn ca_ent)((p_re ca_wa) p_csn)) oa_ent)(u_isn u_hdip))	33	8
3061	((((u_hdip u_hdip)(oa_wa (u_csn cp_ent))) ca_ent)(ca_wa ca_wa))	29	8
3062	((((u_hdip u_hdip)(oa_wa (u_csn cp_ent))) ca_ent)(ca_wa p_re))	29	8
3063	((((u_hdip u_hdip)(oa_wa (u_csn cp_ent))) ca_ent)(p_isn ca_wa))	22	8
3064	((((oa_wa (u_csn cp_ent)) p_csn)(ca_ent p_isn))(p_re ca_wa))	33	8
3065	((((oa_wa (u_csn cp_ent)) p_csn) ca_ent)((ca_wa p_re) ca_wa))	33	8
3066	((((oa_wa (u_csn cp_ent)) p_csn)(p_isn ca_ent))(ca_wa ca_wa))	27	8
3067	((((oa_wa (u_csn cp_ent)) p_csn)(p_isn ca_wa))(p_re ca_wa))	22	8
3068	((((p_csn (ca_wa p_re))(ca_wa p_csn)) oa_ent)(u_hdip u_hdip))	22	8
3069	((((p_csn (ca_wa p_re))(ca_wa p_csn)) oa_ent)(u_isn u_hdip))	23	8
3070	((((p_csn (oa_ent u_isn)) u_hdip)(u_isn oa_wa))(u_csn cp_ent))	37	8
3071	((((p_re (ca_wa p_csn)) oa_ent)(u_isn u_hdip))(u_isn u_hdip))	30	8
3072	((((u_hdip (oa_wa u_csn)) oa_ent)(oa_wa u_hdip))(u_isn u_hdip))	24	8
3073	((((u_hdip (oa_wa u_csn)) oa_ent)(u_isn u_hdip))(u_isn u_hdip))	21	8
3074	((((u_hdip (u_csn cp_ent)) oa_ent)(u_isn u_hdip))(u_isn u_hdip))	44	8
3075	((((u_hdip (u_isn oa_wa)) u_csn)(cp_ent ca_ent))(ca_wa ca_wa))	31	8
3076	((((u_hdip (u_isn oa_wa)) u_csn)(ca_ent ca_wa))(ca_wa p_csn))	24	8
3077	((((u_hdip (u_isn oa_wa)) u_csn)(cp_ent ca_ent))(ca_wa p_re))	31	8
3078	((((u_hdip (u_isn oa_wa)) u_csn)(cp_ent ca_ent))(p_isn ca_wa))	23	8
3079	((((u_hdip (u_isn oa_wa)) u_csn)(cp_ent ca_ent))(p_isn p_re))	30	8
3080	((((u_hdip (u_isn oa_wa)) u_csn)(ca_ent p_isn))(p_re ca_wa))	31	8
3081	((((u_hdip (u_isn oa_wa)) u_csn)(ca_ent ca_wa))(p_isn p_re))	30	8
3082	((((u_hdip (u_isn oa_wa)) u_csn)(cp_ent ca_wa))(p_isn p_re))	25	8
3083	((((u_hdip (u_isn oa_wa)) u_csn)(cp_ent ca_wa))(p_re ca_wa))	26	8
3084	((((u_hdip (u_isn oa_wa)) u_csn)((cp_ent ca_wa) cp_ent)) oa_ent)	20	8
3085	((((u_hdip (u_isn u_csn))(u_isn cp_ent)) ca_ent)(ca_wa ca_wa))	26	8
3086	((((((ca_ent ca_wa) cp_ent) oa_ent)(oa_wa oa_wa))(u_isn u_hdip))	28	8
3087	((((((ca_ent ca_wa) cp_ent) oa_ent)(oa_wa u_hdip))(u_isn u_hdip))	36	8
3088	((((((ca_ent ca_wa) cp_ent) oa_ent)(u_hdip oa_wa))(u_isn u_hdip))	28	8
3089	((((((ca_ent ca_wa) cp_ent) oa_ent)(u_hdip u_hdip))(u_isn oa_wa))	34	8
3090	((((((ca_ent ca_wa) cp_ent) oa_ent)(u_hdip u_hdip))(u_isn u_hdip))	37	8
3091	((((((ca_ent ca_wa) cp_ent) oa_ent)(u_isn oa_wa))(u_isn oa_wa))	35	8
3092	((((((ca_ent ca_wa) cp_ent) oa_ent)(u_isn oa_wa))(u_isn u_hdip))	36	8
3093	((((((ca_ent ca_wa) cp_ent) oa_ent)(u_isn u_hdip))(u_isn oa_wa))	41	8
3094	((((((ca_ent ca_wa) cp_ent) oa_ent)(u_isn u_hdip))(u_isn u_hdip))	44	8
3095	((((((ca_ent ca_wa) p_csn) oa_ent)(oa_wa u_hdip))(u_isn u_hdip))	29	8
3096	((((((ca_ent ca_wa) p_csn) oa_ent)(u_hdip u_hdip))(u_isn u_hdip))	31	8
3097	((((((ca_ent ca_wa) p_csn) oa_ent)(u_isn u_hdip))(oa_wa u_hdip))	31	8
3098	(((((ca_ent ca_wa) p_re)(p_isn p_csn))(p_isn cp_ent)) oa_ent)	22	8
3099	(((((ca_ent p_re) p_csn) oa_ent)(oa_wa u_hdip))(u_isn u_hdip))	28	8
3100	(((((ca_ent p_re) p_csn) oa_ent)(u_isn u_hdip))(u_isn oa_wa))	31	8
3101	(((((ca_ent p_re) p_csn) oa_ent)(u_isn u_hdip))(u_isn u_hdip))	35	8
3102	((((((ca_wa p_re) ca_wa) oa_ent)(oa_wa u_hdip))(u_isn u_hdip))	32	8
3103	((((((ca_wa p_re) ca_wa) oa_ent)(u_hdip u_hdip))(u_isn oa_wa))	30	8
3104	((((((ca_wa p_re) ca_wa) oa_ent)(u_hdip u_hdip))(u_isn u_hdip))	35	8
3105	((((((ca_wa p_re) ca_wa) oa_ent)(u_isn u_hdip))(u_isn oa_wa))	32	8
3106	((((((ca_wa p_re) ca_wa) oa_ent)(u_isn u_hdip))(u_isn u_hdip))	36	8
3107	((((((ca_wa p_re) cp_ent) oa_ent)(oa_wa oa_wa))(u_csn cp_ent))	29	8
3108	((((((ca_wa p_re) cp_ent) oa_ent)(oa_wa u_hdip))(u_isn u_hdip))	34	8
3109	((((((ca_wa p_re) cp_ent) oa_ent)(u_hdip u_hdip))(u_isn u_hdip))	38	8
3110	((((((ca_wa p_re) cp_ent) oa_ent)(u_isn oa_wa))(u_csn cp_ent))	27	8
3111	((((((ca_wa p_re) cp_ent) oa_ent)(u_isn oa_wa))(u_isn oa_wa))	34	8
3112	((((((ca_wa p_re) cp_ent) oa_ent)(u_isn oa_wa))(u_isn u_hdip))	36	8
3113	((((((ca_wa p_re) cp_ent) oa_ent)(u_isn u_hdip))(oa_wa u_hdip))	33	8
3114	((((((ca_wa p_re) cp_ent) oa_ent)(u_isn u_hdip))(u_isn oa_wa))	39	8
3115	((((((ca_wa p_re) cp_ent) oa_ent)(u_isn u_hdip))(u_isn u_hdip))	42	8
3116	((((((cp_ent ca_ent) cp_ent) oa_ent)(oa_wa u_hdip))(u_isn u_hdip))	34	8
3117	((((((cp_ent ca_ent) cp_ent) oa_ent)(u_hdip oa_wa))(u_isn oa_wa))	26	8
3118	((((((cp_ent ca_ent) cp_ent) oa_ent)(u_hdip oa_wa))(u_isn u_hdip))	26	8
3119	((((((cp_ent ca_ent) cp_ent) oa_ent)(u_hdip u_hdip))(u_isn u_hdip))	35	8
3120	((((((cp_ent ca_ent) cp_ent) oa_ent)(u_isn oa_wa))(u_isn oa_wa))	35	8
3121	((((((cp_ent ca_ent) cp_ent) oa_ent)(u_isn oa_wa))(u_isn u_hdip))	34	8
3122	((((((cp_ent ca_wa) cp_ent) oa_ent)(oa_wa oa_wa))(u_csn cp_ent))	29	8
3123	((((((cp_ent ca_wa) cp_ent) oa_ent)(oa_wa u_hdip))(u_isn u_hdip))	33	8
3124	((((((cp_ent ca_wa) cp_ent) oa_ent)(u_hdip u_hdip))(u_isn u_hdip))	35	8
3125	((((((cp_ent oa_ent) oa_wa) u_csn)(u_isn cp_ent))(ca_ent p_isn))	27	8
3126	((((((cp_ent oa_ent) oa_wa) u_csn)(u_isn cp_ent))(ca_ent ca_wa))	27	8
3127	((((((cp_ent oa_ent) oa_wa) u_csn)(u_isn cp_ent))(ca_wa ca_wa))	21	8
3128	((((((cp_ent oa_ent) oa_wa) u_csn)(u_isn cp_ent))(ca_wa p_re))	20	8
3129	(((((oa_ent oa_wa) u_csn)(u_isn cp_ent)) ca_ent)(ca_wa ca_wa))	22	8
3130	(((((oa_ent oa_wa) u_csn)(u_isn cp_ent)) ca_ent)(ca_wa p_csn))	19	8
3131	(((((p_re ca_wa) p_csn) oa_ent)(oa_wa u_hdip))(u_isn u_hdip))	28	8
3132	(((((p_re ca_wa) p_csn) oa_ent)(u_hdip u_hdip))(u_isn u_hdip))	34	8
3133	(((((p_re ca_wa) p_csn) oa_ent)(u_isn u_hdip))(oa_wa u_hdip))	33	8
3134	(((((p_re ca_wa) p_csn) oa_ent)(u_isn u_hdip))(u_isn oa_wa))	35	8
3135	(((((p_re ca_wa) p_csn) oa_ent)(u_isn u_hdip))(u_isn u_hdip))	38	8
3136	(((((u_isn oa_wa) u_hdip)(oa_wa u_csn)) oa_ent)(oa_wa u_hdip))	24	8
3137	(((u_csn cp_ent)(p_csn (ca_ent ca_wa)))((p_re ca_wa)(cp_ent oa_ent)))	24	9
3138	(((ca_ent p_isn)(p_re (ca_wa p_csn)))((p_isn oa_ent)(u_isn u_hdip)))	24	9
3139	((ca_ent p_isn)((p_re ca_wa)((p_csn cp_ent)(oa_ent (u_isn u_hdip)))))	30	9
3140	((ca_ent p_isn)((p_re ca_wa)((p_csn cp_ent)((oa_ent u_isn) u_hdip))))	31	9
3141	((cp_ent ca_ent)((p_isn p_re)((ca_wa (p_csn cp_ent))(oa_ent u_hdip))))	29	9
3142	((((p_isn ca_ent)(ca_wa p_re))(ca_wa p_csn))((p_isn oa_ent) u_hdip))	22	9
3143	(((u_csn (cp_ent ca_wa))(p_re ca_wa))((cp_ent oa_ent)(u_isn u_hdip)))	28	9
3144	(((u_hdip (u_csn cp_ent))(ca_ent ca_wa))((p_re ca_wa)(cp_ent oa_ent)))	27	9
3145	((ca_ent ca_wa)(((p_re ca_wa)(p_csn cp_ent))(p_csn (oa_ent u_isn))))	20	9
3146	((ca_ent ca_wa)((ca_wa (p_csn cp_ent))((p_csn oa_ent)(u_isn oa_wa))))	18	9
3147	(((ca_ent p_isn)((p_re (ca_wa p_csn))(oa_ent u_isn)))(u_hdip u_hdip))	30	9
3148	((ca_ent p_isn)(((p_re (ca_wa p_csn))(cp_ent oa_ent))(u_isn u_hdip)))	34	9
3149	((ca_ent p_isn)(((p_re (ca_wa p_csn))(oa_ent u_hdip))(u_isn u_hdip)))	29	9
3150	((ca_ent ca_wa)(((p_isn ca_wa) p_csn)((p_isn oa_ent)(u_isn u_hdip))))	21	9
3151	((ca_ent ca_wa)(((p_isn ca_wa)(p_csn cp_ent))(oa_ent (u_isn u_hdip))))	30	9
3152	((ca_ent ca_wa)(((p_isn ca_wa)(p_csn cp_ent))((oa_ent u_isn) u_hdip)))	31	9
3153	((ca_ent ca_wa)((p_isn p_re)((ca_wa (p_csn cp_ent))(oa_ent u_isn))))	37	9
3154	((ca_ent ca_wa)((p_isn p_re)((ca_wa (p_csn cp_ent))(oa_ent oa_wa))))	30	9
3155	((ca_ent ca_wa)((p_isn p_re)((ca_wa (p_csn cp_ent))(oa_ent u_hdip))))	32	9
3156	((ca_ent ca_wa)(((p_re ca_wa) oa_ent)((oa_wa u_hdip)(u_isn oa_wa))))	24	9
3157	((ca_ent ca_wa)(((p_re ca_wa) p_csn)((p_isn oa_ent)(u_isn u_hdip))))	29	9
3158	((ca_ent ca_wa)(((p_re ca_wa)(p_csn cp_ent))((p_csn oa_ent) oa_wa)))	19	9
3159	((ca_ent p_isn)(((p_re ca_wa) p_csn)((p_isn oa_ent)(u_isn u_hdip))))	27	9
3160	((ca_ent p_isn)(((p_re ca_wa)(p_csn cp_ent))((oa_ent u_isn) u_hdip)))	32	9
3161	(((ca_ent ca_wa) p_re)(((p_csn cp_ent)(oa_ent u_isn))(oa_wa u_hdip)))	28	9
3162	((ca_wa p_re)(((p_csn oa_ent)(u_isn u_hdip))((u_isn oa_wa) u_hdip)))	27	9
3163	((cp_ent ca_ent)(((ca_wa p_re) ca_wa)((p_csn cp_ent)(oa_ent u_isn))))	36	9
3164	(((cp_ent ca_ent)(ca_wa p_re))((ca_wa (p_csn cp_ent))(oa_ent oa_wa)))	28	9
3165	((cp_ent ca_ent)(((ca_wa p_re) ca_wa)((p_csn oa_ent)(oa_wa u_hdip))))	31	9
3166	((cp_ent ca_ent)((p_re ca_wa)((p_csn cp_ent)((oa_ent u_isn) u_hdip))))	29	9
3167	((cp_ent ca_ent)((p_re ca_wa)((p_csn cp_ent)(oa_ent (u_isn u_hdip)))))	28	9
3168	(((cp_ent ca_ent)(((p_isn ca_wa) p_csn)(oa_ent u_isn)))(u_hdip u_hdip))	28	9
3169	((cp_ent ca_ent)((((p_isn ca_wa) p_csn)(oa_ent u_hdip))(u_isn u_hdip)))	27	9
3170	((cp_ent ca_ent)(((ca_wa p_re) ca_wa)((cp_ent oa_ent)(oa_wa u_hdip))))	36	9
3171	((cp_ent ca_ent)(((ca_wa p_re) ca_wa)((cp_ent oa_ent)(u_isn u_hdip))))	36	9
3172	((cp_ent ca_ent)(((ca_wa p_re) ca_wa)((p_csn oa_ent)(u_isn oa_wa))))	26	9
3173	((cp_ent ca_ent)(((ca_wa p_re) ca_wa)((p_csn oa_ent)(u_isn u_hdip))))	35	9
3174	((cp_ent ca_ent)(((p_isn ca_wa) p_csn)((p_isn oa_ent)(u_isn u_hdip))))	21	9
3175	((cp_ent ca_ent)(((p_isn ca_wa)(p_csn cp_ent))(oa_ent (u_isn u_hdip))))	28	9
3176	((cp_ent ca_ent)(((p_isn ca_wa)(p_csn cp_ent))((oa_ent u_isn) u_hdip)))	29	9
3177	((cp_ent ca_ent)((p_isn p_re)((ca_wa (p_csn cp_ent))(oa_ent oa_wa))))	28	9
3178	((cp_ent ca_ent)(((p_re ca_wa) p_csn)((p_isn oa_ent)(u_isn u_hdip))))	27	9
3179	((cp_ent ca_ent)(((ca_wa p_re)(ca_wa (p_csn cp_ent)))(oa_ent oa_wa)))	29	9
3180	((cp_ent ca_ent)(((ca_wa p_re)(ca_wa (p_csn cp_ent)))(oa_ent u_hdip)))	30	9
3181	((cp_ent ca_ent)((((ca_wa p_re) ca_wa)(p_csn oa_ent))(oa_wa u_hdip)))	30	9
3182	((cp_ent ca_ent)((((ca_wa p_re) ca_wa)(p_csn oa_ent))(u_isn u_hdip)))	38	9
3183	((cp_ent ca_wa)(((p_isn ca_wa) p_csn)((p_isn oa_ent)(u_isn u_hdip))))	20	9
3184	((cp_ent ca_wa)(((p_re ca_wa) p_csn)((p_isn oa_ent)(u_isn u_hdip))))	28	9
3185	((oa_wa oa_wa)(((u_csn cp_ent)(p_csn (ca_ent ca_wa)))(p_re ca_wa)))	20	9
3186	((oa_wa oa_wa)(((u_csn cp_ent)(p_isn ca_ent)))((ca_wa p_re) ca_wa)))	20	9
3187	((oa_wa oa_wa)(((u_csn cp_ent) p_csn)((p_isn ca_ent)(ca_wa p_re))))	18	9
3188	(((oa_wa u_csn)((u_isn cp_ent)(ca_ent ca_wa)))((p_re ca_wa) p_csn))	24	9
3189	((oa_wa u_hdip)(((u_csn cp_ent)(p_csn (ca_ent ca_wa)))(p_re ca_wa)))	25	9
3190	(((oa_wa u_hdip)(u_isn oa_wa))((u_csn cp_ent) p_csn))(ca_ent ca_wa))	17	9
3191	((p_csn ca_ent)(((ca_wa p_re) ca_wa)((cp_ent oa_ent)(oa_wa u_hdip))))	24	9
3192	((p_csn ca_ent)(((ca_wa p_re) ca_wa)((cp_ent oa_ent)(u_isn u_hdip))))	29	9
3193	((p_csn (ca_ent ca_wa))(((p_re ca_wa)(cp_ent oa_ent))(u_hdip u_hdip)))	29	9
3194	(((p_isn ca_ent)(ca_wa p_re))((ca_wa (p_csn cp_ent))(oa_ent u_isn)))	28	9
3195	(((p_isn ca_ent)(ca_wa p_re))((ca_wa (p_csn cp_ent))(oa_ent oa_wa)))	22	9
3196	((p_isn ca_ent)((p_isn p_re)((ca_wa (p_csn cp_ent))(oa_ent u_hdip))))	25	9
3197	((p_isn ca_ent)(((ca_wa p_re) ca_wa)((cp_ent oa_ent)(oa_wa u_hdip))))	30	9
3198	((p_isn ca_ent)(((ca_wa p_re) ca_wa)((p_csn (oa_ent u_isn)) u_hdip)))	31	9
3199	((p_isn ca_ent)((p_re ca_wa)((p_csn cp_ent)((oa_ent u_isn) u_hdip))))	25	9
3200	((p_isn ca_ent)((p_re ca_wa)((p_csn cp_ent)(oa_ent (u_isn u_hdip)))))	24	9
3201	(((p_isn ca_ent)(((p_isn ca_wa) p_csn)(oa_ent u_isn)))(u_hdip u_hdip))	24	9
3202	((p_isn ca_ent)((((p_isn ca_wa) p_csn)(oa_ent u_hdip))(u_isn u_hdip)))	23	9
3203	((p_isn ca_ent)(((ca_wa p_re) ca_wa)((cp_ent oa_ent)(u_isn u_hdip))))	33	9
3204	((p_isn ca_ent)(((ca_wa p_re) ca_wa)((p_csn oa_ent)(oa_wa u_hdip))))	27	9
3205	((p_isn ca_ent)(((ca_wa p_re) ca_wa)((p_csn oa_ent)(u_isn oa_wa))))	22	9
3206	((p_isn ca_ent)(((ca_wa p_re) ca_wa)((p_csn oa_ent)(u_isn u_hdip))))	32	9
3207	((p_isn ca_ent)(((p_isn ca_wa) p_csn)((p_isn oa_ent)(u_isn u_hdip))))	20	9
3208	((p_isn ca_ent)(((p_isn ca_wa)(p_csn cp_ent))(oa_ent (u_isn u_hdip))))	24	9
3209	((p_isn ca_ent)(((p_isn ca_wa)(p_csn cp_ent))((oa_ent u_isn) u_hdip)))	25	9
3210	((p_isn ca_ent)((p_isn p_re)((ca_wa (p_csn cp_ent))(oa_ent u_isn))))	28	9
3211	((p_isn ca_ent)(((ca_wa p_re) ca_wa)((p_csn (p_isn oa_ent)) u_hdip)))	26	9
3212	((p_isn ca_ent)(((p_re ca_wa) p_csn)((p_isn oa_ent)(u_isn u_hdip))))	26	9
3213	((p_isn ca_ent)(((ca_wa p_re)(ca_wa (p_csn cp_ent)))(oa_ent oa_wa)))	23	9
3214	((p_isn ca_ent)(((ca_wa p_re)(ca_wa (p_csn cp_ent)))(oa_ent u_hdip)))	26	9
3215	((p_isn ca_ent)((((ca_wa p_re) ca_wa)(p_csn oa_ent))(u_hdip u_hdip)))	31	9
3216	((p_isn ca_ent)((((ca_wa p_re) ca_wa)(p_csn oa_ent))(u_isn u_hdip)))	33	9
3217	((p_isn ca_wa)(((p_isn p_re)(p_csn cp_ent))((oa_ent u_isn) u_hdip)))	31	9
3218	((p_isn ca_wa)(((p_isn p_re)(p_csn cp_ent))(oa_ent (oa_wa u_hdip))))	27	9
3219	((p_isn ca_wa)(((p_isn p_re)(p_csn cp_ent))(oa_ent (u_isn u_hdip))))	30	9
3220	((p_isn ca_wa)(((p_re ca_wa) oa_ent)((oa_wa u_hdip)(u_isn oa_wa))))	22	9
3221	((p_isn ca_wa)(((p_re ca_wa) p_csn)((p_isn oa_ent)(u_isn u_hdip))))	29	9
3222	((p_re ca_wa)(((p_csn oa_ent)(u_isn u_hdip))((u_isn oa_wa) u_hdip)))	30	9
3223	((u_csn (cp_ent ca_ent))((ca_wa p_re)((p_csn oa_ent)(u_isn u_hdip))))	23	9
3224	(((u_csn cp_ent)(ca_ent ca_wa))((p_re ca_wa)(p_csn (oa_ent u_hdip))))	24	9
3225	(((u_csn cp_ent)(ca_ent ca_wa))((p_re ca_wa)(p_csn (oa_ent u_isn))))	27	9
3226	(((u_csn cp_ent)(ca_ent ca_wa))((p_re ca_wa)((cp_ent oa_ent) oa_wa)))	26	9
3227	(((u_csn cp_ent)(ca_ent ca_wa))((p_re ca_wa)((cp_ent oa_ent) u_hdip)))	33	9
3228	(((u_csn cp_ent)(ca_ent ca_wa))((p_re ca_wa)((p_csn oa_ent) u_hdip)))	26	9
3229	((u_csn (cp_ent ca_ent))(((ca_wa p_re) cp_ent)(oa_ent (u_isn u_hdip))))	32	9
3230	((u_csn (cp_ent ca_ent))(((ca_wa p_re) cp_ent)((oa_ent oa_wa) u_hdip)))	27	9
3231	((u_csn (cp_ent ca_ent))(((ca_wa p_re) cp_ent)((oa_ent u_isn) u_hdip)))	33	9
3232	((u_csn (cp_ent ca_ent))(((ca_wa p_re) cp_ent)((p_isn oa_ent) u_hdip)))	21	9
3233	((u_csn (cp_ent ca_ent))(((ca_wa p_re)(p_csn oa_ent))(u_hdip u_hdip)))	24	9
3234	((u_csn (cp_ent ca_ent))(((ca_wa p_re)(p_csn oa_ent))(u_isn u_hdip)))	24	9
3235	(((u_csn cp_ent)(ca_ent ca_wa))(((p_re ca_wa) oa_ent)(u_hdip u_hdip)))	24	9
3236	(((u_csn cp_ent)(ca_ent ca_wa))(((p_re ca_wa) oa_ent)(u_isn u_hdip)))	24	9
3237	(((u_csn cp_ent)(ca_ent ca_wa))(((p_re ca_wa) p_csn)(oa_ent u_isn)))	28	9
3238	(((u_csn cp_ent)(ca_ent ca_wa))(((p_re ca_wa) p_csn)(oa_ent u_hdip)))	26	9
3239	(((u_csn cp_ent)(ca_ent ca_wa))(((p_re ca_wa) p_csn)(p_isn oa_ent)))	22	9
3240	((u_hdip oa_wa)(((u_csn cp_ent)(p_csn (ca_ent ca_wa)))(p_re ca_wa)))	25	9
3241	((u_isn u_hdip)(((u_isn u_hdip)(oa_wa u_csn))(oa_ent (oa_wa u_hdip))))	17	9
3242	((u_isn u_hdip)(((u_isn u_hdip)(oa_wa u_csn))(oa_ent (u_isn u_hdip))))	17	9
3243	((oa_ent (oa_wa u_hdip))(((u_isn u_hdip)(oa_wa u_csn))(cp_ent oa_ent)))	25	9
3244	((oa_ent (oa_wa u_hdip))(((u_isn u_hdip)(oa_wa u_csn))(u_isn cp_ent)))	29	9
3245	((oa_ent (u_isn u_hdip))(((u_isn u_hdip)(oa_wa u_csn))(cp_ent oa_ent)))	27	9
3246	((oa_ent (u_isn u_hdip))(((u_isn u_hdip)(oa_wa u_csn))(cp_ent oa_ent)))	28	9
3247	((oa_wa (u_csn (cp_ent ca_ent)))((ca_wa p_re)(p_csn (oa_ent u_isn))))	22	9
3248	((oa_wa (u_csn (cp_ent ca_ent)))((ca_wa p_re)((p_csn oa_ent) u_hdip)))	20	9
3249	((oa_wa (u_csn (cp_ent ca_ent)))(((ca_wa p_re) ca_wa)(p_csn oa_ent)))	24	9
3250	((oa_wa (u_csn (cp_ent ca_ent)))(((ca_wa p_re) ca_wa)(cp_ent oa_ent)))	31	9
3251	((oa_wa (u_csn (cp_ent ca_ent)))(((ca_wa p_re) cp_ent)(oa_ent u_isn)))	30	9
3252	((oa_wa (u_csn (cp_ent ca_ent)))(((ca_wa p_re) cp_ent)(oa_ent oa_wa)))	24	9

3253	((oa_wa (u_csn (cp_ent ca_ent)))(((ca_wa p_re) cp_ent)(oa_ent u_hdip)))	30	9
3254	((oa_wa (u_csn (cp_ent ca_ent)))((p_re ca_wa) p_csn)(oa_ent u_isn)))	25	9
3255	((p_csn (ca_ent ca_wa))((p_re ca_wa))((cp_ent oa_ent)(oa_wa u_hdip)))	28	9
3256	((p_csn (ca_ent ca_wa))((p_re ca_wa))((cp_ent oa_ent)(u_isn u_hdip))))	27	9
3257	((p_csn (ca_ent ca_wa))(((p_re ca_wa)(cp_ent oa_ent))(oa_wa u_hdip)))	25	9
3258	((p_csn (ca_ent ca_wa))(((p_re ca_wa)(cp_ent oa_ent))(u_isn u_hdip)))	28	9
3259	((p_csn (p_isn ca_ent))(((ca_wa p_re) ca_wa)((cp_ent oa_ent) u_hdip)))	21	9
3260	((p_csn (p_isn ca_ent))(((p_re ca_wa)(cp_ent oa_ent))(u_isn u_hdip)))	19	9
3261	((p_csn (p_isn ca_wa))(((p_re ca_wa)(cp_ent oa_ent))(u_hdip u_hdip)))	26	9
3262	((p_csn (p_isn ca_wa))(((p_re ca_wa)(cp_ent oa_ent))(u_isn u_hdip)))	24	9
3263	((u_csn (cp_ent ca_wa))((p_re ca_wa))((cp_ent oa_ent)(oa_wa u_hdip)))	24	9
3264	((u_csn (cp_ent ca_wa))(((p_re ca_wa)(cp_ent oa_ent))(oa_wa u_hdip)))	23	9
3265	((u_csn (cp_ent ca_wa))(((p_re ca_wa)(cp_ent oa_ent))(u_isn u_hdip)))	24	9
3266	(((u_csn cp_ent)(p_csn ca_ent))(((ca_wa p_re) ca_wa)(cp_ent oa_ent)))	27	9
3267	(((u_csn cp_ent)(p_csn ca_ent))(((ca_wa p_re) ca_wa)(oa_ent u_isn)))	22	9
3268	((((u_csn cp_ent) p_csn) ca_ent)(((ca_wa p_re) cp_ent)(oa_ent u_isn)))	27	9
3269	((((u_csn cp_ent) p_csn) ca_ent)(((ca_wa p_re) cp_ent)(oa_ent u_hdip)))	27	9
3270	(((u_csn cp_ent)(p_csn ca_ent))(((ca_wa p_re) cp_ent)(p_isn oa_ent)))	18	9
3271	((u_csn (p_isn ca_ent))((ca_wa p_re))((p_csn oa_ent)(u_isn u_hdip))))	19	9
3272	((u_csn (p_isn ca_ent))(((ca_wa p_re) ca_wa)((cp_ent oa_ent) u_hdip)))	27	9
3273	((u_csn (p_isn ca_ent))(((ca_wa p_re) ca_wa)(p_csn (oa_ent u_isn))))	22	9
3274	(((u_csn cp_ent)(p_isn ca_ent))(((ca_wa p_re) cp_ent)(p_isn oa_ent)))	20	9
3275	((u_csn (p_isn ca_ent))(((ca_wa p_re) cp_ent)(oa_ent (oa_wa u_hdip))))	22	9
3276	((u_csn (p_isn ca_ent))(((ca_wa p_re) cp_ent)(oa_ent (u_isn u_hdip))))	26	9
3277	((u_csn (p_isn ca_ent))(((ca_wa p_re) cp_ent)((oa_ent u_isn) u_hdip)))	27	9
3278	((u_csn (p_isn ca_ent))(((ca_wa p_re)(p_csn oa_ent))(u_isn u_hdip)))	20	9
3279	((u_csn (p_isn ca_ent))(((p_re ca_wa)(cp_ent oa_ent))(oa_wa u_hdip)))	20	9
3280	((u_csn (p_isn ca_ent))(((p_re ca_wa)(cp_ent oa_ent))(u_isn u_hdip)))	25	9
3281	((u_hdip (u_isn oa_wa))((u_csn (cp_ent ca_ent)))((ca_wa p_re) ca_wa)))	30	9
3282	((u_hdip (u_isn oa_wa))(((u_csn cp_ent)(ca_ent p_isn))(p_re ca_wa)))	30	9
3283	((u_hdip (u_isn oa_wa))(((u_csn cp_ent)(p_csn ca_ent))(ca_wa ca_wa)))	25	9
3284	((u_hdip (u_isn oa_wa))(((u_csn cp_ent)(p_csn ca_ent))(ca_wa p_re)))	25	9
3285	((u_hdip (u_isn oa_wa))(((u_csn cp_ent)(p_csn ca_ent))(p_re ca_wa)))	25	9
3286	((u_hdip (u_isn oa_wa))(((u_csn cp_ent)(ca_ent ca_wa))(p_re ca_wa)))	31	9
3287	((u_hdip (u_isn oa_wa))(((u_csn cp_ent)(oa_wa u_hdip))(u_isn u_hdip)))	22	9
3288	((u_hdip (u_isn u_csn))(((u_isn cp_ent)(ca_ent ca_wa))(p_re ca_wa)))	25	9
3289	((u_hdip (u_isn u_csn))(((u_isn cp_ent)(ca_ent ca_wa))(ca_wa p_csn)))	20	9
3290	((u_hdip (u_isn u_csn))(((u_isn cp_ent) ca_ent)((ca_wa p_re) cp_ent)))	23	9
3291	((((ca_ent ca_wa) cp_ent)(((p_csn (oa_ent u_isn)) u_hdip)(u_isn oa_wa)))	27	9
3292	((((ca_ent ca_wa) cp_ent)(((p_csn oa_ent)(u_isn u_hdip))(u_isn u_hdip)))	26	9
3293	((((ca_ent ca_wa) p_re)(((p_csn cp_ent)(oa_ent oa_wa))(u_isn u_hdip)))	27	9
3294	((((ca_wa p_re) ca_wa)(((p_csn cp_ent)(oa_ent oa_wa))(u_isn u_hdip)))	29	9
3295	((((ca_wa p_re) ca_wa)(((p_csn cp_ent)(oa_ent u_isn))(u_hdip u_hdip)))	33	9
3296	((((ca_wa p_re) ca_wa)(((p_csn cp_ent) oa_ent)((u_isn oa_wa) u_hdip)))	28	9
3297	((((ca_wa p_re) ca_wa)(((p_csn cp_ent)(oa_ent u_hdip))(u_isn u_hdip)))	31	9
3298	((((ca_wa p_re) ca_wa)(((p_csn cp_ent)(oa_ent u_isn))(oa_wa u_hdip)))	30	9
3299	((((ca_wa p_re) ca_wa)(((p_csn oa_ent) u_hdip)((u_isn oa_wa) u_hdip)))	30	9
3300	((((ca_wa p_re) ca_wa)(((p_csn oa_ent)(u_hdip oa_wa))(u_isn u_hdip)))	24	9
3301	((((ca_wa p_re) ca_wa)(((p_csn oa_ent)(u_isn u_hdip))(oa_wa u_hdip)))	31	9
3302	((((ca_wa p_re) ca_wa)(((p_csn oa_ent)(u_isn u_hdip))(u_isn oa_wa)))	30	9
3303	((((ca_wa p_re) ca_wa)(((p_csn oa_ent)(u_isn u_hdip))(u_isn u_hdip)))	36	9
3304	(((cp_ent ca_ent)(ca_wa p_re))((ca_wa (p_csn cp_ent))(oa_ent u_isn)))	34	9
3305	((((cp_ent oa_ent) oa_wa)((u_hdip u_hdip)((oa_wa u_csn)(u_isn cp_ent))))	30	9
3306	((((cp_ent oa_ent) oa_wa)((u_isn u_hdip)((u_isn u_hdip)(oa_wa u_csn))))	36	9
3307	((((cp_ent oa_ent) u_hdip)((u_isn u_hdip)((u_isn u_hdip)(oa_wa u_csn))))	33	9
3308	((((cp_ent oa_ent)(oa_wa u_hdip)))((u_isn u_hdip)(oa_wa (u_csn cp_ent))))	37	9
3309	((((cp_ent oa_ent)(oa_wa u_hdip)))((u_isn u_hdip)((u_isn oa_wa) u_csn)))	40	9
3310	((((cp_ent oa_ent)(u_isn u_hdip)))((u_isn u_hdip)(oa_wa (u_csn cp_ent))))	38	9
3311	((((cp_ent oa_ent)(u_isn u_hdip)))((u_isn u_hdip)((u_isn oa_wa) u_csn)))	41	9
3312	((((oa_ent u_isn) u_hdip)((u_isn u_hdip)(oa_wa u_csn))(cp_ent oa_ent)))	29	9
3313	((((oa_ent u_isn) u_hdip)((u_isn u_hdip)(oa_wa u_csn))(u_isn cp_ent)))	26	9
3314	((((oa_ent u_isn) u_hdip)((u_isn u_hdip)(u_isn u_hdip))(oa_wa u_csn)))	33	9
3315	((((oa_ent u_isn) u_hdip)(((u_isn u_hdip)(oa_wa u_csn))(cp_ent oa_ent)))	30	9
3316	((((oa_ent u_isn) u_hdip)(((u_isn u_hdip)(oa_wa u_csn))(u_isn cp_ent)))	30	9
3317	((((oa_wa (u_csn cp_ent)) ca_ent)((ca_wa p_re)(p_csn (oa_ent u_hdip))))	19	9
3318	((((oa_wa (u_csn cp_ent)) ca_ent)((ca_wa p_re)(p_csn (oa_ent u_isn))))	23	9
3319	((((oa_wa (u_csn cp_ent)) ca_ent)((ca_wa p_re)((p_csn oa_ent) u_hdip)))	21	9
3320	(((oa_wa (u_csn cp_ent))(ca_ent ca_wa))(((p_re ca_wa)(p_csn oa_ent)))	25	9
3321	((((oa_wa (u_csn cp_ent)) ca_ent)(((ca_wa p_re) ca_wa)(cp_ent oa_ent)))	32	9
3322	((((oa_wa (u_csn cp_ent)) ca_ent)(((ca_wa p_re) ca_wa)(oa_ent u_isn)))	24	9
3323	((((oa_wa (u_csn cp_ent)) ca_ent)(((ca_wa p_re) cp_ent)(oa_ent u_isn)))	31	9
3324	((((oa_wa (u_csn cp_ent)) ca_ent)(((ca_wa p_re) cp_ent)(oa_ent oa_wa)))	25	9
3325	((((oa_wa (u_csn cp_ent)) ca_ent)(((ca_wa p_re) cp_ent)(oa_ent u_hdip)))	31	9
3326	(((oa_wa (u_csn cp_ent))(ca_ent ca_wa))(((p_re ca_wa) p_csn) oa_ent))	26	9
3327	(((oa_wa u_csn)(cp_ent ca_wa))(((p_re ca_wa)((cp_ent oa_ent) oa_wa)))	24	9
3328	(((oa_wa u_csn)(cp_ent ca_wa))(((p_re ca_wa)((cp_ent oa_ent) u_hdip)))	25	9
3329	(((oa_wa (u_csn cp_ent))(p_csn ca_ent))(((ca_wa p_re) ca_wa) oa_ent))	20	9
3330	((((oa_wa u_csn)(p_isn ca_ent))(((ca_wa p_re) ca_wa)(cp_ent oa_ent)))	25	9
3331	((((oa_wa u_csn)(p_isn ca_ent))(((ca_wa p_re) cp_ent)(oa_ent u_isn)))	24	9
3332	((((oa_wa u_csn)(p_isn ca_ent))(((ca_wa p_re) cp_ent)(oa_ent oa_wa)))	19	9
3333	((((oa_wa u_csn)(p_isn ca_ent))(((ca_wa p_re) cp_ent)(oa_ent u_hdip)))	24	9
3334	((((oa_wa u_hdip) oa_wa)((u_csn (cp_ent ca_ent)))((ca_wa p_re) ca_wa)))	29	9
3335	((((oa_wa u_hdip) oa_wa)(((u_csn cp_ent)(ca_ent p_isn))(p_re ca_wa)))	29	9
3336	((((oa_wa u_hdip) oa_wa)(((u_csn cp_ent)(p_csn ca_ent))(ca_wa ca_wa)))	23	9
3337	((((oa_wa u_hdip) oa_wa)(((u_csn cp_ent)(p_csn ca_ent))(ca_wa p_re)))	23	9
3338	((((oa_wa u_hdip) oa_wa)(((u_csn cp_ent)(p_csn ca_ent))(p_re ca_wa)))	23	9
3339	((((oa_wa u_hdip) oa_wa)(((u_csn cp_ent)(ca_ent ca_wa))(p_re ca_wa)))	30	9
3340	((((oa_wa u_hdip) oa_wa)(((u_csn cp_ent)(p_isn ca_wa))(p_re ca_wa)))	24	9
3341	(((oa_wa u_hdip)(u_isn oa_wa))(((u_csn cp_ent)(p_csn (ca_ent ca_wa))))	22	9
3342	(((oa_wa u_hdip)(u_isn oa_wa))((u_csn (cp_ent ca_wa))(p_re ca_wa)))	22	9
3343	(((oa_wa u_hdip)(u_isn oa_wa))((((u_csn cp_ent) p_csn)(ca_ent p_isn)))	24	9
3344	(((oa_wa u_hdip)(u_isn oa_wa))((((u_csn cp_ent) p_csn)(ca_ent ca_wa)))	24	9
3345	(((oa_wa u_hdip)(u_isn oa_wa))((((u_csn cp_ent) p_csn)(ca_wa ca_wa)))	21	9
3346	((((p_csn oa_ent)(oa_wa u_hdip)))((u_isn oa_wa)((u_csn cp_ent) p_csn)))	25	9
3347	((((p_isn ca_ent)(ca_wa p_re))(p_csn ((p_isn oa_ent)(u_isn u_hdip))))	26	9
3348	((((p_isn oa_ent) oa_wa)((u_isn u_hdip)((u_isn u_hdip)(oa_wa u_csn))))	27	9
3349	((p_isn oa_ent)((u_isn u_hdip)((u_isn u_hdip)(oa_wa (u_csn cp_ent)))))	32	9
3350	((((p_isn oa_ent)(oa_wa u_hdip))((u_isn u_hdip)(oa_wa (u_csn cp_ent))))	29	9
3351	((((p_isn oa_ent)(oa_wa u_hdip)))((u_isn u_hdip)((u_isn oa_wa) u_csn)))	29	9
3352	((((p_isn oa_ent)(u_isn u_hdip))((u_isn oa_wa)((u_csn cp_ent) p_csn)))	28	9
3353	((((p_isn oa_ent)(u_isn u_hdip)))((u_isn u_hdip)((u_isn oa_wa) u_csn)))	32	9
3354	((((u_csn cp_ent) p_csn)(((ca_wa p_re) cp_ent)((oa_ent u_isn) u_hdip)))	26	9
3355	((((u_csn cp_ent)(ca_wa p_re))((p_csn (oa_ent u_isn))(u_hdip u_hdip)))	27	9
3356	((((u_csn cp_ent)(oa_ent oa_wa))((u_isn u_hdip)((u_isn oa_wa) u_csn)))	33	9
3357	((((u_csn cp_ent)(p_isn ca_ent))((ca_wa p_re)(p_csn (oa_ent u_hdip))))	19	9
3358	((((u_csn cp_ent)(p_isn ca_ent))((ca_wa p_re)(p_csn (oa_ent u_isn))))	22	9
3359	((((u_csn cp_ent)(p_isn ca_ent))(((ca_wa p_re) ca_wa)(cp_ent oa_ent)))	30	9
3360	((((u_csn cp_ent)(p_isn ca_ent))(((ca_wa p_re) ca_wa)(oa_ent u_isn)))	24	9
3361	((((u_csn cp_ent)(p_isn ca_ent))(((ca_wa p_re) cp_ent)(oa_ent u_isn)))	29	9
3362	((((u_csn cp_ent)(p_isn ca_ent))(((ca_wa p_re) cp_ent)(oa_ent oa_wa)))	23	9
3363	((((u_csn cp_ent)(p_isn ca_ent))(((ca_wa p_re) cp_ent)(oa_ent u_hdip)))	29	9
3364	((((u_csn cp_ent)(p_isn ca_ent))(((p_re ca_wa) p_csn)(oa_ent u_isn)))	24	9
3365	((((u_csn cp_ent)(p_isn ca_ent))(((p_re ca_wa) p_csn)(oa_ent u_hdip)))	22	9
3366	((((u_csn cp_ent)(p_isn ca_wa))((p_re ca_wa)((cp_ent oa_ent) oa_wa)))	23	9
3367	((((u_csn cp_ent)(p_isn ca_wa))((p_re ca_wa)((cp_ent oa_ent) u_hdip)))	25	9
3368	((((u_hdip u_csn)(cp_ent ca_ent))(((ca_wa p_re) cp_ent)(oa_ent u_hdip)))	26	9
3369	((((u_hdip u_csn)(cp_ent ca_wa))((p_re ca_wa)((cp_ent oa_ent) u_hdip)))	23	9
3370	((((u_isn cp_ent) ca_ent)(((ca_wa p_re) cp_ent)(oa_ent (oa_wa u_hdip))))	19	9
3371	((((u_isn cp_ent) ca_ent)(((ca_wa p_re) cp_ent)(oa_ent (u_isn u_hdip))))	22	9
3372	((((u_isn cp_ent) ca_ent)(((ca_wa p_re) cp_ent)((oa_ent u_isn) u_hdip)))	23	9
3373	((((u_isn cp_ent)(p_isn ca_ent))(((ca_wa p_re) ca_wa)(cp_ent oa_ent)))	22	9
3374	((((u_isn cp_ent)(p_isn ca_ent))(((ca_wa p_re) cp_ent)(oa_ent u_isn)))	21	9
3375	((((u_isn cp_ent)(p_isn ca_ent))(((ca_wa p_re) cp_ent)(oa_ent u_hdip)))	21	9
3376	((((u_isn oa_wa) u_hdip)((u_csn (cp_ent ca_ent)))((ca_wa p_re) ca_wa)))	25	9
3377	((((u_isn oa_wa) u_hdip)(((u_csn cp_ent)(ca_ent p_isn))(p_re ca_wa)))	25	9
3378	((((u_isn oa_wa) u_hdip)(((u_csn cp_ent)(p_csn ca_ent))(ca_wa ca_wa)))	26	9
3379	((((u_isn oa_wa) u_hdip)(((u_csn cp_ent)(p_csn ca_ent))(ca_wa p_re)))	26	9
3380	((((u_isn oa_wa) u_hdip)(((u_csn cp_ent)(p_csn ca_ent))(p_re ca_wa)))	26	9
3381	((((u_isn oa_wa) u_hdip)(((u_isn oa_wa) u_csn)((cp_ent ca_wa) cp_ent)))	19	9
3382	((((u_isn u_csn)(ca_ent ca_wa))((p_re ca_wa)((cp_ent oa_ent) u_hdip)))	23	9
3383	((((u_isn u_csn)(cp_ent ca_ent))(((ca_wa p_re) cp_ent)(oa_ent u_isn)))	24	9
3384	((((u_isn u_csn)(cp_ent ca_ent))(((ca_wa p_re) cp_ent)(oa_ent u_hdip)))	24	9
3385	((((u_isn u_csn)(cp_ent ca_wa))((p_re ca_wa)((cp_ent oa_ent) u_hdip)))	18	9
3386	(((((ca_ent ca_wa) p_re)((ca_wa p_csn)(cp_ent oa_ent)))(u_hdip u_hdip))	31	9
3387	((((ca_ent ca_wa)((p_isn ca_wa) p_csn))((p_isn oa_ent)(u_isn u_hdip)))	22	9
3388	((((ca_ent ca_wa)((p_isn ca_wa)(p_csn cp_ent)))(oa_ent (oa_wa u_hdip)))	30	9
3389	((((ca_ent ca_wa)((p_re ca_wa) oa_ent))((oa_wa u_hdip)(u_isn oa_wa)))	28	9
3390	((((ca_ent ca_wa)((p_re ca_wa) p_csn))((p_isn oa_ent)(u_isn u_hdip)))	31	9
3391	((((ca_ent ca_wa)((p_re ca_wa)(p_csn cp_ent)))(oa_ent (oa_wa u_hdip)))	29	9
3392	((((ca_ent ca_wa)((p_re ca_wa)(p_csn cp_ent)))(p_csn (oa_ent u_isn)))	22	9
3393	((((ca_ent ca_wa)((p_re ca_wa)(p_csn cp_ent)))((p_csn oa_ent) oa_wa))	20	9
3394	((((ca_ent ca_wa)((p_re ca_wa)(p_csn oa_ent)))((u_isn oa_wa) u_hdip))	32	9
3395	((((ca_ent p_isn)(ca_wa (p_csn cp_ent)))((p_csn oa_ent)(u_isn oa_wa)))	19	9
3396	((((ca_ent p_isn)((p_re ca_wa)(p_csn cp_ent)))(p_csn (oa_ent u_isn)))	21	9
3397	((((ca_ent p_isn)((p_re ca_wa)(p_csn cp_ent)))((p_csn oa_ent) oa_wa))	20	9
3398	(((((ca_ent ca_wa) p_re)(p_csn cp_ent))(oa_ent oa_wa))(u_isn u_hdip))	29	9
3399	(((((ca_ent p_isn)(p_re (ca_wa p_csn)))(oa_ent u_isn))(u_hdip u_hdip))	31	9
3400	(((((ca_ent ca_wa) p_re)(p_csn cp_ent)) oa_ent)((u_isn oa_wa) u_hdip))	28	9
3401	(((((ca_ent ca_wa) p_re)(p_csn cp_ent))(oa_ent u_isn))(oa_wa u_hdip))	30	9
3402	(((((ca_ent ca_wa) p_re)(p_csn cp_ent))(oa_ent u_isn))(u_hdip u_hdip))	33	9
3403	(((ca_wa p_re)((p_csn cp_ent)(oa_ent u_isn)))((oa_wa u_hdip) oa_wa))	28	9
3404	((((ca_ent ca_wa)(p_csn cp_ent))(oa_ent u_isn))((oa_wa u_hdip) oa_wa))	28	9
3405	((((cp_ent ca_ent)((ca_wa ca_wa)(cp_ent oa_ent)))((oa_wa u_hdip) oa_wa))	25	9
3406	((((cp_ent ca_ent)((ca_wa ca_wa)(p_csn oa_ent)))((oa_wa u_hdip) oa_wa))	23	9
3407	((((cp_ent ca_ent)((ca_wa p_re) cp_ent))((p_isn oa_ent)(u_isn u_hdip)))	29	9
3408	(((((cp_ent ca_ent)(ca_wa p_re))(ca_wa (p_csn cp_ent)))(oa_ent u_hdip))	31	9
3409	((((cp_ent ca_ent)((ca_wa p_re)(p_csn cp_ent)))((oa_ent u_isn) u_hdip))	31	9
3410	((((cp_ent ca_ent)((ca_wa p_re)(p_csn oa_ent)))((oa_wa u_hdip) oa_wa))	21	9
3411	((((cp_ent ca_ent)((p_isn ca_wa)(p_csn cp_ent)))(oa_ent (u_isn u_hdip)))	27	9
3412	((((cp_ent ca_ent)((p_re ca_wa) oa_ent))((oa_wa u_hdip)(u_isn oa_wa)))	27	9
3413	(((((cp_ent ca_ent)(ca_wa p_re))(ca_wa (p_csn cp_ent)))(oa_ent oa_wa))	30	9
3414	((((cp_ent ca_ent)((p_re ca_wa)(p_csn cp_ent)))(oa_ent (u_isn u_hdip)))	31	9
3415	((((cp_ent ca_ent)(((ca_wa p_re) ca_wa)(p_csn oa_ent)))(u_hdip u_hdip))	33	9
3416	((((cp_ent ca_wa)((p_re ca_wa) oa_ent))((oa_wa u_hdip)(u_isn oa_wa)))	22	9
3417	((((cp_ent ca_wa)((p_re ca_wa)(p_csn cp_ent)))(oa_ent (oa_wa u_hdip)))	26	9
3418	((((cp_ent ca_wa)((p_re ca_wa)(p_csn cp_ent)))(oa_ent (u_isn u_hdip)))	29	9
3419	((((cp_ent ca_wa)((p_re ca_wa)(p_csn cp_ent)))((oa_ent u_isn) u_hdip))	30	9
3420	((((cp_ent ca_wa)((p_re ca_wa)(p_csn oa_ent)))((oa_wa u_hdip) oa_wa))	22	9
3421	((((oa_ent oa_wa)((u_isn oa_wa) u_csn))((cp_ent oa_ent)(oa_wa u_hdip)))	17	9
3422	((((oa_ent oa_wa)((u_isn oa_wa) u_csn))((cp_ent oa_ent)(u_isn u_hdip)))	17	9
3423	(((((cp_ent oa_ent) oa_wa)((u_isn oa_wa) u_csn))((oa_ent u_isn) u_hdip))	19	9
3424	((((oa_ent (oa_wa u_hdip))((u_isn u_hdip)(oa_wa (u_csn cp_ent))))) oa_ent)	19	9
3425	(((((cp_ent oa_ent) oa_wa)((u_isn u_hdip)(oa_wa u_csn)))(u_isn cp_ent))	30	9
3426	((((oa_ent u_isn) u_hdip)(((u_isn u_hdip)(oa_wa (u_csn cp_ent))) oa_ent))	31	9
3427	(((((oa_ent u_isn) u_hdip)((u_isn u_hdip)(oa_wa u_csn)))(u_isn cp_ent))	31	9
3428	(((((cp_ent oa_ent) u_hdip)((u_isn u_hdip)(oa_wa u_csn)))(u_isn cp_ent))	31	9
3429	((((oa_wa oa_wa)((u_csn cp_ent)(p_csn ca_ent)))(((ca_wa p_re) cp_ent))	20	9
3430	((((oa_wa oa_wa)((u_csn cp_ent)(p_isn ca_ent)))(((ca_wa p_re) ca_wa))	19	9
3431	((((oa_wa (u_csn cp_ent))(p_csn (ca_ent ca_wa)))(((p_re ca_wa) oa_ent))	19	9
3432	((((oa_wa u_csn)((ca_wa p_re) ca_wa)))((cp_ent oa_ent)(u_isn u_hdip)))	26	9
3433	((((oa_wa u_hdip)((u_csn cp_ent)(p_csn ca_ent)))(((ca_wa p_re) cp_ent))	25	9
3434	((((oa_wa u_hdip)((u_isn u_hdip)(oa_wa u_csn)))(((cp_ent oa_ent) oa_wa))	26	9
3435	((((p_isn ca_ent)((ca_wa ca_wa)(cp_ent oa_ent)))((oa_wa u_hdip) oa_wa))	19	9
3436	((((p_isn ca_ent)((ca_wa p_re)(p_csn cp_ent)))(oa_ent (u_isn u_hdip)))	25	9
3437	((((p_isn ca_ent)((ca_wa p_re)(p_csn cp_ent)))((oa_ent u_isn) u_hdip))	26	9
3438	((((p_isn ca_ent)((p_isn ca_wa) p_csn))((p_isn oa_ent)(u_isn u_hdip)))	21	9
3439	((((p_isn ca_ent)((p_isn ca_wa)(p_csn cp_ent)))(oa_ent (u_isn u_hdip)))	23	9
3440	((((p_isn ca_ent)((p_isn ca_wa)(p_csn cp_ent)))((oa_ent u_isn) u_hdip))	24	9
3441	((((p_isn ca_ent)((p_re ca_wa) oa_ent))((oa_wa u_hdip)(u_isn oa_wa)))	19	9
3442	((((p_isn ca_ent)((p_re ca_wa) p_csn))((p_isn oa_ent)(u_isn u_hdip)))	28	9
3443	((((p_isn ca_wa)(p_csn cp_ent))(oa_ent u_isn)))((oa_wa u_hdip) oa_wa))	28	9
3444	((((p_isn ca_wa)((p_re ca_wa) oa_ent))((oa_wa u_hdip)(u_isn oa_wa)))	23	9
3445	(((((ca_wa p_re) ca_wa)((cp_ent oa_ent)(oa_wa u_hdip)))(u_isn u_hdip))	34	9
3446	(((((ca_wa p_re) ca_wa)((p_csn oa_ent) u_hdip))((u_isn oa_wa) u_hdip))	31	9
3447	(((((ca_wa p_re) ca_wa)((p_csn oa_ent)(oa_wa u_hdip)))(u_isn u_hdip))	28	9
3448	(((((u_csn cp_ent)(ca_ent ca_wa))(p_re ca_wa))((cp_ent oa_ent) u_hdip))	36	9
3449	((((u_csn (cp_ent ca_ent))((ca_wa p_re) ca_wa))((oa_ent u_isn) u_hdip))	35	9
3450	((((u_csn (cp_ent ca_ent))((ca_wa p_re) ca_wa))((p_csn oa_ent) u_hdip))	29	9
3451	((((u_csn cp_ent)(p_csn (p_isn ca_ent)))((p_re ca_wa)(cp_ent oa_ent)))	19	9
3452	((((u_csn cp_ent)(p_csn (p_isn ca_ent)))(((ca_wa p_re) ca_wa) oa_ent))	18	9
3453	(((((u_csn cp_ent)(ca_ent ca_wa))(p_isn p_re))((cp_ent oa_ent) u_hdip))	35	9
3454	((((u_csn cp_ent)((p_isn ca_ent)(ca_wa p_re)))((cp_ent oa_ent) u_hdip))	31	9
3455	((((u_hdip oa_wa)((u_csn cp_ent)(ca_ent ca_wa)))(((p_re ca_wa) oa_ent))	20	9
3456	((((u_hdip oa_wa)(u_csn (cp_ent ca_ent)))(((ca_wa p_re) ca_wa) oa_ent))	22	9
3457	((((u_hdip oa_wa)(u_csn (cp_ent ca_ent)))(((ca_wa p_re) cp_ent) oa_ent))	25	9
3458	((((u_hdip oa_wa)((u_csn cp_ent)(p_csn ca_ent)))(((ca_wa p_re) cp_ent))	25	9
3459	((((u_hdip u_hdip)((u_csn cp_ent)(p_csn (ca_ent ca_wa))))((p_re ca_wa))	22	9
3460	((((u_hdip u_hdip)((u_csn cp_ent)(p_csn ca_ent)))(((ca_wa p_re) cp_ent))	22	9
3461	((((u_isn cp_ent)((ca_wa p_re) ca_wa))((cp_ent oa_ent)(u_isn u_hdip)))	24	9
3462	(((((u_isn cp_ent) ca_ent)((ca_wa p_re) ca_wa))((p_csn cp_ent) oa_ent))	17	9
3463	((((u_isn u_hdip)((u_isn u_hdip)(oa_wa u_csn)))(oa_ent (oa_wa u_hdip)))	20	9
3464	((((u_isn u_hdip)((u_isn u_hdip)(oa_wa u_csn)))(oa_ent (u_isn u_hdip)))	20	9
3465	((((u_isn u_hdip)((u_isn u_hdip)(oa_wa u_csn)))((cp_ent oa_ent) oa_wa))	34	9
3466	((((oa_ent (oa_wa u_hdip)))((u_isn oa_wa) u_csn))(oa_ent (oa_wa u_hdip)))	17	9
3467	((((oa_ent (oa_wa u_hdip)))((u_isn u_hdip)(oa_wa u_csn)))(u_isn cp_ent))	30	9
3468	((((oa_ent (u_isn u_hdip)))((u_isn u_hdip)(oa_wa u_csn)))(u_isn cp_ent))	29	9
3469	((((oa_wa (u_csn cp_ent))(p_isn ca_ent))((p_re ca_wa)(cp_ent oa_ent)))	24	9
3470	((((oa_wa (u_csn cp_ent))(p_isn ca_ent)))(((ca_wa p_re) ca_wa) oa_ent))	21	9
3471	((((oa_wa (u_csn cp_ent))(p_isn ca_ent)))(((ca_wa p_re) cp_ent) oa_ent))	25	9
3472	((((oa_wa (u_csn cp_ent))(p_isn ca_ent)))(((p_re ca_wa) p_csn) oa_ent))	21	9
3473	((((oa_wa (u_csn cp_ent))(p_isn ca_wa))((p_re ca_wa)(cp_ent oa_ent)))	22	9
3474	((((oa_wa (u_csn cp_ent))((ca_wa p_re) ca_wa))((cp_ent oa_ent) u_hdip))	28	9
3475	((((p_csn (ca_ent ca_wa))((p_re ca_wa) p_csn))(oa_ent (oa_wa u_hdip)))	24	9
3476	((((p_csn (ca_ent ca_wa))((p_re ca_wa) p_csn))(oa_ent (u_isn u_hdip)))	28	9
3477	((((p_csn (ca_ent ca_wa))((p_re ca_wa) p_csn))((oa_ent oa_wa) u_hdip))	25	9
3478	((((p_csn (ca_ent ca_wa))((p_re ca_wa) p_csn))((p_isn oa_ent) u_hdip))	23	9
3479	((((p_csn (ca_ent ca_wa))((p_re ca_wa)(cp_ent oa_ent)))(u_hdip u_hdip))	28	9
3480	((((p_csn (ca_ent ca_wa))((p_re ca_wa)(cp_ent oa_ent)))(u_isn u_hdip))	24	9
3481	((((p_csn (ca_wa p_re))(ca_wa p_csn))((p_isn oa_ent)(u_isn u_hdip)))	21	9
3482	((((p_csn (ca_wa p_re))(ca_wa (p_csn cp_ent)))(oa_ent (u_isn u_hdip)))	24	9
3483	((((p_csn (ca_wa p_re))(ca_wa (p_csn cp_ent)))((oa_ent u_isn) u_hdip))	25	9
3484	((((p_csn (oa_ent u_hdip))(u_isn oa_wa))(((u_csn cp_ent) p_csn) ca_ent))	25	9
3485	((((p_csn (p_isn ca_wa)))((p_re ca_wa)(cp_ent oa_ent)))(u_hdip u_hdip))	24	9
3486	(((((u_csn cp_ent)(ca_ent ca_wa))(p_isn ca_wa))((cp_ent oa_ent) u_hdip))	34	9
3487	((((u_csn (ca_ent ca_wa))(p_isn ca_wa))((cp_ent oa_ent)(u_isn u_hdip)))	34	9
3488	(((((u_csn cp_ent)(ca_ent ca_wa))(p_isn ca_wa))((p_csn oa_ent) u_hdip))	28	9
3489	(((((u_csn cp_ent)(ca_ent ca_wa))(p_re p_re))(p_csn (oa_ent u_hdip)))	21	9
3490	(((((u_csn cp_ent)(ca_ent ca_wa))(p_re p_re))(p_csn (oa_ent u_isn)))	23	9
3491	(((((u_csn cp_ent)(ca_ent ca_wa))(p_re p_re))(p_csn (p_isn oa_ent)))	20	9
3492	((((u_csn (cp_ent ca_wa))(p_isn ca_wa))((cp_ent oa_ent)(oa_wa u_hdip)))	29	9
3493	((((u_csn (cp_ent ca_wa))(p_isn ca_wa))((cp_ent oa_ent)(u_isn u_hdip)))	27	9
3494	((((u_csn (cp_ent ca_wa))(p_re ca_wa))((cp_ent oa_ent)(oa_wa u_hdip)))	28	9
3495	((((u_csn (cp_ent ca_wa))(p_re ca_wa))((cp_ent oa_ent)(oa_wa oa_wa)))	23	9
3496	((((u_csn (cp_ent ca_wa))(p_re ca_wa))((cp_ent oa_ent)(u_isn oa_wa)))	24	9
3497	(((((u_csn cp_ent)(p_isn ca_ent))(ca_wa ca_wa))((cp_ent oa_ent) u_hdip))	31	9
3498	((((u_csn (p_isn ca_ent))(ca_wa p_re))((p_csn oa_ent)(u_isn u_hdip)))	24	9
3499	((((u_csn (p_isn ca_ent))(p_isn ca_wa))((cp_ent oa_ent)(oa_wa u_hdip)))	24	9
3500	((((u_csn (p_isn ca_ent))(p_re p_re))((p_csn oa_ent)(u_isn u_hdip)))	21	9
3501	((((u_csn (p_isn ca_ent))(p_re p_re))(p_csn ((p_isn oa_ent) u_hdip)))	19	9
3502	(((((u_csn cp_ent)(p_isn ca_ent))((ca_wa p_re) ca_wa))(cp_ent oa_ent))	31	9
3503	((((u_csn (p_isn ca_ent))((p_re ca_wa)(cp_ent oa_ent)))(u_hdip u_hdip))	22	9
3504	((((u_csn (u_isn cp_ent)) ca_ent)(((ca_wa p_re) ca_wa)(cp_ent oa_ent)))	23	9
3505	((((u_csn (u_isn cp_ent)) ca_ent)(((ca_wa p_re) cp_ent)(oa_ent u_isn)))	23	9
3506	((((u_csn (u_isn cp_ent)) ca_ent)(((ca_wa p_re) cp_ent)(oa_ent oa_wa)))	19	9
3507	((((u_csn (u_isn cp_ent)) ca_ent)(((ca_wa p_re) cp_ent)(oa_ent u_hdip)))	23	9
3508	(((((u_csn (u_isn cp_ent))(ca_ent ca_wa))(p_isn p_re))(ca_wa p_csn))	25	9
3509	((((u_csn (u_isn cp_ent))(ca_wa ca_wa))((cp_ent oa_ent)(oa_wa u_hdip)))	20	9
3510	((((u_csn (u_isn cp_ent))((ca_wa p_re) ca_wa))((cp_ent oa_ent) u_hdip))	23	9
3511	(((((u_csn (u_isn cp_ent))(p_isn ca_ent))((ca_wa p_re))(ca_wa p_csn))	21	9
3512	((((u_hdip (oa_wa u_csn))(ca_ent ca_wa))((p_re ca_wa)(cp_ent oa_ent)))	24	9
3513	((((u_hdip (oa_wa u_csn))(cp_ent ca_ent))(((ca_wa p_re) cp_ent) oa_ent))	23	9
3514	((((u_hdip (oa_wa u_csn))(cp_ent ca_wa))((p_re ca_wa)(cp_ent oa_ent)))	19	9
3515	((((u_hdip (u_csn cp_ent)) ca_ent)(((ca_wa p_re) cp_ent)(oa_ent oa_wa)))	20	9
3516	((((u_hdip (u_csn cp_ent))(p_csn ca_ent))(((ca_wa p_re) ca_wa) oa_ent))	18	9
3517	((((u_hdip (u_csn cp_ent))(p_isn ca_ent)))(((ca_wa p_re) ca_wa) oa_ent))	20	9
3518	((((u_hdip (u_csn cp_ent))(p_isn ca_ent)))(((ca_wa p_re) cp_ent) oa_ent))	22	9
3519	((((u_hdip (u_csn cp_ent))(p_isn ca_wa))((p_re ca_wa)(cp_ent oa_ent)))	24	9
3520	((((u_hdip (u_csn cp_ent))(p_csn (p_isn ca_ent)))((ca_wa p_re) cp_ent))	20	9
3521	((((u_hdip (u_isn oa_wa)) u_csn)((cp_ent ca_ent))((ca_wa p_re) cp_ent)))	29	9
3522	((((u_hdip (u_isn oa_wa))(u_csn (cp_ent ca_ent)))((ca_wa p_re) cp_ent))	29	9
3523	((((u_hdip (u_isn oa_wa))(((u_csn cp_ent) p_csn))((ca_ent ca_wa) cp_ent))	26	9
3524	((((u_hdip (u_isn oa_wa))((u_csn cp_ent)(ca_ent ca_wa)))(ca_wa p_csn))	24	9
3525	((((u_hdip (u_isn u_csn))(ca_ent ca_wa))((p_re ca_wa)(cp_ent oa_ent)))	23	9
3526	((((u_hdip (u_isn u_csn))(cp_ent ca_ent))(((ca_wa p_re) cp_ent) oa_ent))	23	9
3527	(((((ca_ent ca_wa) cp_ent)(oa_ent (u_isn u_hdip)))((u_isn oa_wa) u_hdip))	33	9
3528	(((((ca_ent ca_wa) cp_ent)((oa_ent u_isn) u_hdip)))((u_isn oa_wa) u_hdip))	35	9
3529	(((((ca_ent ca_wa) cp_ent)((p_csn oa_ent)(u_isn oa_wa)))(u_isn u_hdip))	24	9
3530	(((((ca_ent ca_wa) cp_ent)((p_isn oa_ent)(u_isn u_hdip)))(u_isn u_hdip))	28	9
3531	(((ca_ent p_isn)((((p_re ca_wa) p_csn)(cp_ent oa_ent))(u_hdip u_hdip)))	32	9
3532	(((ca_ent ca_wa)((((p_re ca_wa) p_csn)(oa_ent oa_wa))(u_hdip u_hdip)))	28	9
3533	(((ca_ent p_isn)((((p_re ca_wa) p_csn)(oa_ent u_hdip))(u_isn u_hdip)))	35	9
3534	(((ca_ent ca_wa)((((p_re ca_wa) p_csn)(p_isn oa_ent)))(u_hdip u_hdip))	26	9
3535	(((((ca_ent ca_wa) p_re)(cp_ent oa_ent))((oa_wa u_hdip)(u_isn oa_wa)))	30	9
3536	(((((ca_ent ca_wa) p_re)(p_csn oa_ent))((oa_wa u_hdip)(u_isn oa_wa)))	23	9
3537	((((ca_wa p_re)(p_csn cp_ent))(oa_ent (u_isn u_hdip)))(u_isn oa_wa))	22	9
3538	((((p_re ca_wa)(p_csn cp_ent))(oa_ent (u_isn u_hdip)))(u_isn oa_wa))	22	9
3539	((((ca_wa p_re)(p_csn cp_ent))(oa_ent (u_isn u_hdip)))(u_isn u_hdip))	31	9
3540	((((ca_wa p_re)(p_csn cp_ent))((oa_ent u_isn) u_hdip))(u_isn oa_wa))	23	9
3541	((((ca_wa p_re)(p_csn cp_ent))((oa_ent u_isn) u_hdip))(u_isn u_hdip))	32	9
3542	((((ca_ent ca_wa)((p_isn ca_wa) p_csn)) oa_ent)((oa_wa u_hdip) oa_wa))	26	9
3543	((((ca_wa p_re) ca_wa)(cp_ent oa_ent))((oa_wa u_hdip)(u_isn oa_wa)))	30	9
3544	(((((ca_wa p_re) ca_wa)(p_csn oa_ent))((oa_wa u_hdip)(u_isn oa_wa)))	22	9
3545	(((((ca_wa p_re) ca_wa)((cp_ent oa_ent)(u_isn u_hdip)))(u_isn oa_wa))	35	9
3546	(((((ca_wa p_re) ca_wa)((cp_ent oa_ent)(u_isn u_hdip)))(u_isn u_hdip))	37	9
3547	(((((ca_wa p_re) ca_wa)((p_csn oa_ent)(u_isn oa_wa)))(cp_ent ca_wa))	28	9
3548	(((((ca_wa p_re) ca_wa)((p_csn oa_ent)(u_isn u_hdip)))(u_isn oa_wa))	25	9
3549	(((((ca_wa p_re) cp_ent) oa_ent)(u_isn u_hdip))((u_isn oa_wa) u_hdip))	34	9
3550	(((((ca_wa p_re) cp_ent)((oa_ent u_isn) u_hdip))((u_isn oa_wa) u_hdip))	36	9
3551	(((((ca_wa p_re) cp_ent)((p_isn oa_ent)(u_isn u_hdip)))(u_isn u_hdip))	26	9
3552	((((ca_wa p_re)(p_csn oa_ent))((u_isn u_hdip)))((u_isn oa_wa) u_hdip))	27	9
3553	(((((cp_ent ca_ent) cp_ent)(oa_ent (u_isn u_hdip)))((u_isn oa_wa) u_hdip))	31	9
3554	(((((cp_ent ca_ent) cp_ent)((oa_ent u_isn) u_hdip)))((u_isn oa_wa) u_hdip))	33	9
3555	(((((cp_ent ca_ent) cp_ent)((p_isn oa_ent)(u_isn u_hdip)))(u_isn u_hdip))	28	9
3556	(((((cp_ent ca_ent)(ca_wa p_re))(p_csn oa_ent)))((oa_wa u_hdip) oa_wa))	21	9
3557	(((((cp_ent ca_ent)(ca_wa p_re))(ca_wa (p_csn cp_ent)))(oa_ent u_isn))	37	9
3558	(((((cp_ent ca_ent)(ca_wa p_re))(p_csn (oa_ent u_isn)))(u_hdip u_hdip))	32	9
3559	(((((cp_ent ca_ent)(ca_wa p_re))((p_csn oa_ent) u_hdip))(u_isn u_hdip))	28	9
3560	(((((cp_ent ca_wa) cp_ent)(oa_ent (u_isn u_hdip)))((u_isn oa_wa) u_hdip))	31	9
3561	(((((cp_ent ca_wa) cp_ent)((oa_ent u_isn) u_hdip)))((u_isn oa_wa) u_hdip))	33	9
3562	(((((cp_ent ca_wa) cp_ent)((p_isn oa_ent)(u_isn u_hdip)))(u_isn u_hdip))	27	9
3563	(((((cp_ent oa_ent)(oa_wa u_hdip))((u_isn oa_wa) u_csn))(oa_ent u_isn))	21	9
3564	(((((cp_ent oa_ent)(oa_wa u_hdip))((u_isn oa_wa) u_csn))(u_isn cp_ent))	35	9
3565	(((((cp_ent oa_ent)(u_isn u_hdip))((u_isn oa_wa) u_csn))(u_isn cp_ent))	36	9
3566	(((((oa_wa u_csn) p_csn) ca_ent)(((ca_wa p_re) ca_wa)(cp_ent oa_ent)))	23	9
3567	(((((oa_wa u_csn) p_csn) ca_ent)(((ca_wa p_re) cp_ent)(oa_ent u_isn)))	23	9
3568	(((((oa_wa u_csn) p_csn) ca_ent)(((ca_wa p_re) cp_ent)(oa_ent u_hdip)))	24	9
3569	(((((oa_wa u_hdip) oa_wa)(u_csn (cp_ent ca_ent)))((ca_wa p_re) cp_ent))	28	9
3570	(((((oa_wa u_hdip)(oa_wa u_csn))(cp_ent ca_ent)))((ca_wa p_re) ca_wa))	25	9
3571	(((((oa_wa u_hdip)(oa_wa u_csn))(cp_ent ca_ent)))((ca_wa p_re) cp_ent))	24	9
3572	(((((oa_wa u_hdip)(u_isn oa_wa))(u_csn (cp_ent ca_ent)))(ca_wa ca_wa))	25	9
3573	(((((oa_wa u_hdip)(u_isn oa_wa))(u_csn (cp_ent ca_ent)))(ca_wa p_csn))	21	9
3574	(((((oa_wa u_hdip)(u_isn oa_wa))(u_csn (cp_ent ca_wa)))(ca_wa p_csn))	19	9
3575	(((((oa_wa u_hdip)(u_isn oa_wa))((u_csn cp_ent) p_csn))(ca_wa ca_wa))	17	9
3576	((((p_csn (ca_ent ca_wa))(p_isn p_re))(ca_wa p_csn))(oa_ent u_hdip))	25	9
3577	((((p_csn (ca_ent ca_wa))(p_isn p_re))(ca_wa p_csn))(cp_ent oa_ent))	27	9
3578	((((p_csn oa_ent)(u_isn u_hdip))(u_isn oa_wa))((u_csn cp_ent) p_csn))	33	9
3579	((((p_isn ca_ent)(ca_wa p_re))(p_csn (oa_ent u_isn)))(u_hdip u_hdip))	28	9
3580	((((p_isn ca_ent)(ca_wa p_re))(p_csn (p_isn oa_ent)))(u_hdip u_hdip))	24	9
3581	((((p_isn ca_ent)(ca_wa p_re))(p_csn (p_isn oa_ent)))(u_isn u_hdip))	27	9
3582	((((p_isn ca_wa) p_csn)((p_isn oa_ent)(u_isn u_hdip)))(u_isn u_hdip))	20	9
3583	((((p_isn ca_wa)(p_csn cp_ent))(oa_ent (u_isn u_hdip)))(u_isn u_hdip))	32	9
3584	((((p_isn ca_wa)(p_csn cp_ent))((oa_ent u_isn) u_hdip))(u_isn u_hdip))	33	9
3585	((((p_isn p_re)(p_csn cp_ent))(oa_ent (u_isn u_hdip)))(u_isn oa_wa))	21	9
3586	((((p_isn p_re)(p_csn cp_ent))(oa_ent (u_isn u_hdip)))(u_isn u_hdip))	29	9
3587	((((p_isn p_re)(p_csn cp_ent))((oa_ent u_isn) u_hdip))(u_isn oa_wa))	22	9
3588	((((p_isn p_re)(p_csn cp_ent))((oa_ent u_isn) u_hdip))(u_isn u_hdip))	30	9
3589	((((p_re ca_wa) p_csn)((p_isn oa_ent)(u_isn u_hdip)))(u_isn u_hdip))	25	9
3590	((((p_re ca_wa)(p_csn oa_ent))(u_isn u_hdip))((u_isn oa_wa) u_hdip))	31	9
3591	(((((u_csn cp_ent) p_csn) ca_ent)(((ca_wa p_re) ca_wa)(cp_ent oa_ent)))	26	9
3592	(((((u_csn cp_ent) p_csn) ca_ent)(((ca_wa p_re) ca_wa)(oa_ent u_isn)))	23	9
3593	(((((u_csn cp_ent) p_csn)(p_isn ca_ent))((p_re ca_wa)(cp_ent oa_ent)))	21	9
3594	(((((u_csn cp_ent) p_csn)(p_isn ca_ent))(((ca_wa p_re) ca_wa) oa_ent))	20	9
3595	(((((u_csn cp_ent)(ca_ent ca_wa))(p_isn ca_wa))((oa_ent u_isn) u_hdip))	34	9
3596	(((((u_csn cp_ent)(ca_ent ca_wa))(p_re p_re))((p_csn oa_ent) u_hdip))	24	9
3597	(((((u_csn cp_ent)(ca_wa ca_wa))((cp_ent oa_ent) u_hdip))(u_isn oa_wa))	27	9
3598	(((((u_csn cp_ent)(ca_wa ca_wa))((cp_ent oa_ent) u_hdip))(u_isn u_hdip))	26	9
3599	(((((u_csn cp_ent)(ca_wa p_re))(p_isn ca_wa))((cp_ent oa_ent) u_hdip))	24	9
3600	(((((u_csn cp_ent)(ca_wa p_re))(p_isn ca_wa))((p_csn oa_ent) u_hdip))	21	9
3601	(((((u_csn cp_ent)(ca_wa p_re))((cp_ent oa_ent) u_hdip))(u_isn u_hdip))	30	9
3602	(((((u_csn cp_ent)(ca_wa p_re))((p_csn oa_ent) u_hdip))(u_isn u_hdip))	26	9
3603	(((((u_csn cp_ent)(p_isn ca_ent))(p_re p_re))((p_csn oa_ent) u_hdip))	22	9
3604	(((((u_csn cp_ent)(p_isn ca_ent))((p_re ca_wa) oa_ent))(u_hdip u_hdip))	21	9
3605	(((((u_csn cp_ent)(p_isn ca_wa))(p_isn ca_wa))(oa_ent (oa_wa u_hdip)))	24	9
3606	(((((u_csn cp_ent)(p_isn ca_wa))(p_isn ca_wa))((cp_ent oa_ent) oa_wa))	23	9
3607	(((((u_csn cp_ent)(p_isn ca_wa))(p_isn ca_wa))((cp_ent oa_ent) u_hdip))	25	9
3608	(((((u_csn cp_ent)(p_isn ca_wa))(p_isn ca_wa))((oa_ent u_isn) u_hdip))	25	9
3609	(((((u_csn cp_ent)(p_isn ca_wa))(p_isn p_re))((cp_ent oa_ent) u_hdip))	28	9
3610	(((((u_csn cp_ent)(p_isn ca_wa))(p_re ca_wa))((cp_ent oa_ent) oa_wa))	27	9
3611	(((((u_csn cp_ent)(p_isn ca_wa))(p_re ca_wa))((cp_ent oa_ent) u_hdip))	29	9
3612	(((((u_hdip oa_wa) u_csn)(ca_ent ca_wa))((p_re ca_wa)(cp_ent oa_ent)))	27	9
3613	(((((u_hdip oa_wa) u_csn)(ca_ent ca_wa))((p_re ca_wa)(p_csn oa_ent)))	21	9
3614	(((((u_hdip oa_wa) u_csn)(cp_ent ca_ent))(((ca_wa p_re) ca_wa) oa_ent))	23	9
3615	(((((u_hdip oa_wa) u_csn)(cp_ent ca_ent))(((ca_wa p_re) cp_ent) oa_ent))	26	9
3616	(((((u_hdip oa_wa) u_csn)(ca_ent ca_wa))(((p_re ca_wa) p_csn) oa_ent))	22	9
3617	(((((u_hdip oa_wa) u_csn)(ca_ent ca_wa))(p_isn p_re))(ca_wa p_csn))	23	9
3618	(((((u_hdip oa_wa) u_csn)(cp_ent ca_wa))((p_re ca_wa)(cp_ent oa_ent)))	22	9
3619	((((((u_hdip oa_wa) u_csn)(cp_ent ca_wa)) cp_ent)(oa_ent (oa_wa u_hdip)))	19	9
3620	(((((u_hdip u_csn)(ca_ent ca_wa))(p_isn ca_wa))((cp_ent oa_ent) u_hdip))	23	9
3621	(((((u_hdip u_csn)(ca_ent ca_wa))(p_isn ca_wa))(oa_ent (oa_wa u_hdip)))	22	9
3622	(((((u_hdip u_csn)(ca_ent ca_wa))(p_isn ca_wa))((oa_ent u_isn) u_hdip))	23	9
3623	(((((u_hdip u_csn)(cp_ent ca_ent))((ca_wa p_re) ca_wa))(cp_ent oa_ent))	29	9
3624	(((((u_hdip u_csn)(cp_ent ca_ent))(p_isn ca_wa))((cp_ent oa_ent) u_hdip))	25	9
3625	(((((u_hdip u_csn)(cp_ent ca_wa))(p_re ca_wa))((cp_ent oa_ent) u_hdip))	25	9
3626	(((((u_hdip u_csn)(u_isn cp_ent))((oa_ent u_isn) u_hdip))(u_isn u_hdip))	31	9
3627	(((((u_isn cp_ent)(p_isn ca_ent))((ca_wa p_re) ca_wa))(p_csn cp_ent))	19	9
3628	(((((u_isn oa_wa) u_csn)(cp_ent ca_ent)))(((ca_wa p_re) ca_wa) oa_ent))	22	9
3629	(((((u_isn oa_wa) u_csn)(cp_ent ca_ent)))(((ca_wa p_re) cp_ent) oa_ent))	25	9
3630	(((((u_isn oa_wa) u_csn)(cp_ent ca_wa))((p_re ca_wa)(cp_ent oa_ent)))	21	9
3631	(((((u_isn oa_wa) u_hdip)(u_csn (cp_ent ca_ent)))((ca_wa p_re) cp_ent))	24	9
3632	(((((u_isn oa_wa) u_hdip)((u_csn cp_ent)(p_csn ca_ent)))(ca_wa ca_wa))	25	9
3633	(((((u_isn oa_wa) u_hdip)((u_csn cp_ent)(p_csn ca_ent)))(ca_wa p_re))	25	9
3634	(((((u_isn oa_wa) u_hdip)((u_csn cp_ent)(p_csn ca_ent)))(p_re ca_wa))	25	9
3635	(((((u_isn oa_wa) u_hdip)((u_isn u_hdip)(oa_wa u_csn)))(cp_ent oa_ent))	19	9
3636	(((((u_isn oa_wa) u_hdip)((u_isn u_hdip)(oa_wa u_csn)))(oa_ent u_isn))	17	9
3637	((((u_isn u_hdip)(oa_wa u_csn))(oa_ent (oa_wa u_hdip)))(u_isn u_hdip))	19	9
3638	((((u_isn u_hdip)(oa_wa u_csn))(oa_ent (u_isn u_hdip)))(u_isn u_hdip))	19	9
3639	(p_csn ((ca_ent ca_wa)((p_re ca_wa)(cp_ent oa_ent))(u_isn u_hdip))))	25	9
3640	(p_csn ((ca_ent ca_wa)(((p_re ca_wa)(cp_ent oa_ent))(u_hdip u_hdip))))	28	9
3641	(p_csn ((ca_ent ca_wa)(((p_re ca_wa)(cp_ent oa_ent))(u_isn u_hdip))))	28	9
3642	(p_csn ((ca_ent p_isn)((p_re ca_wa)(cp_ent oa_ent))(u_isn u_hdip))))	24	9
3643	(p_csn ((p_isn ca_ent)(((ca_wa p_re) ca_wa)((cp_ent oa_ent) u_hdip))))	25	9
3644	(p_csn ((p_isn ca_ent)(((ca_wa p_re) cp_ent)(oa_ent (u_isn u_hdip)))))	23	9
3645	(p_csn ((p_isn ca_ent)(((ca_wa p_re) cp_ent)((oa_ent u_isn) u_hdip))))	25	9
3646	(p_csn ((p_isn ca_ent)(((ca_wa p_re) cp_ent)((p_isn oa_ent) u_hdip))))	19	9
3647	(p_csn ((p_isn ca_ent)(((p_re ca_wa)(cp_ent oa_ent))(u_isn u_hdip))))	22	9
3648	(p_csn (((p_isn oa_ent) oa_wa)((u_isn u_hdip)((u_isn oa_wa) u_csn))))	19	9
3649	(p_csn (((p_isn ca_ent)((p_re ca_wa)(cp_ent oa_ent)))(u_isn u_hdip)))	23	9
3650	((p_csn (((ca_ent ca_wa) cp_ent)((oa_ent u_isn) u_hdip)))(u_isn u_hdip))	28	9
3651	(p_csn ((((ca_ent ca_wa) cp_ent)(oa_ent (u_isn u_hdip)))(u_isn u_hdip)))	27	9
3652	((ca_ent ca_wa)(((p_isn ca_wa)((p_csn cp_ent) oa_ent))(oa_wa u_hdip)))	28	9
3653	((ca_ent ca_wa)((p_re ca_wa)(p_csn ((p_isn oa_ent)(u_isn u_hdip)))))	25	9
3654	((ca_ent ca_wa)((p_re ca_wa)((p_csn cp_ent)(oa_ent (u_isn u_hdip)))))	32	9
3655	((ca_ent ca_wa)((p_re ca_wa)((p_csn cp_ent)((oa_ent u_isn) u_hdip))))	33	9
3656	((ca_ent ca_wa)((p_re ca_wa)((p_csn (oa_ent u_hdip))(u_isn oa_wa))))	27	9
3657	((ca_ent ca_wa)((p_re ca_wa)((p_csn (oa_ent u_isn))(u_hdip u_hdip))))	38	9
3658	((ca_ent ca_wa)(((p_re (ca_wa p_csn))(cp_ent oa_ent))(u_isn u_hdip)))	35	9
3659	((ca_ent ca_wa)(((p_isn p_re)(ca_wa (p_csn cp_ent)))(p_csn oa_ent)))	20	9

3660	((ca_ent ca_wa)(((p_re ca_wa)(p_csn (oa_ent oa_wa)))(u_hdip u_hdip)))	27	9
3661	((ca_ent ca_wa)(((p_re ca_wa)(p_csn (oa_ent oa_wa)))(u_isn u_hdip)))	33	9
3662	((ca_ent ca_wa)(((p_re ca_wa)(p_csn (oa_ent u_hdip)))(u_isn u_hdip)))	35	9
3663	((ca_ent ca_wa)(((p_re ca_wa)(p_csn (oa_ent u_isn)))(oa_wa u_hdip)))	35	9
3664	((ca_ent ca_wa)(((p_re ca_wa)(p_csn (oa_ent u_isn)))(u_hdip u_hdip)))	39	9
3665	((ca_ent ca_wa)(((p_re ca_wa)(p_csn (p_isn oa_ent)))(u_isn u_hdip)))	29	9
3666	((ca_ent ca_wa)(((p_re ca_wa)((cp_ent oa_ent) oa_wa))(u_hdip u_hdip)))	36	9
3667	((ca_ent ca_wa)(((p_re ca_wa)((cp_ent oa_ent) oa_wa))(u_isn oa_wa)))	37	9
3668	((ca_ent ca_wa)(((p_re ca_wa)((cp_ent oa_ent) oa_wa))(u_isn u_hdip)))	38	9
3669	((ca_ent ca_wa)(((p_re ca_wa)((cp_ent oa_ent) u_hdip))(u_isn oa_wa)))	39	9
3670	((ca_ent ca_wa)(((p_re ca_wa)((cp_ent oa_ent) u_hdip))(u_isn u_hdip)))	41	9
3671	((ca_ent ca_wa)(((p_re ca_wa)((p_csn oa_ent) oa_wa))(u_hdip u_hdip)))	31	9
3672	((ca_ent ca_wa)(((p_re ca_wa)((p_csn oa_ent) oa_wa))(u_isn u_hdip)))	32	9
3673	((ca_ent ca_wa)(((p_re ca_wa)((p_csn oa_ent) u_hdip))(oa_wa u_hdip)))	32	9
3674	((ca_ent ca_wa)(((p_re ca_wa)((p_csn oa_ent) u_hdip))(u_isn u_hdip)))	37	9
3675	((ca_ent ca_wa)((((p_isn ca_wa) p_csn)(oa_ent oa_wa))(u_hdip u_hdip)))	23	9
3676	((ca_ent ca_wa)((((p_isn ca_wa) p_csn)(oa_ent oa_wa))(u_isn u_hdip)))	23	9
3677	((ca_ent ca_wa)((((p_isn ca_wa) p_csn) oa_ent)((oa_wa u_hdip) oa_wa)))	25	9
3678	((ca_ent ca_wa)((((p_isn ca_wa) p_csn)(p_isn oa_ent))(u_hdip u_hdip)))	21	9
3679	((ca_ent ca_wa)((((p_isn ca_wa) p_csn)(p_isn oa_ent))(u_isn u_hdip)))	22	9
3680	((ca_ent ca_wa)((((p_re ca_wa) oa_ent)(oa_wa u_hdip))(u_isn oa_wa)))	27	9
3681	((ca_ent ca_wa)((((p_re ca_wa) oa_ent)(oa_wa u_hdip))(u_isn u_hdip)))	29	9
3682	((ca_ent ca_wa)((((p_re ca_wa) oa_ent)(u_hdip u_hdip))(u_isn u_hdip)))	33	9
3683	((ca_ent ca_wa)((((p_re ca_wa) oa_ent)(u_isn u_hdip))(u_isn oa_wa)))	31	9
3684	((ca_ent ca_wa)((((p_re ca_wa) oa_ent)(u_isn u_hdip))(u_isn u_hdip)))	34	9
3685	((ca_ent ca_wa)((((p_re ca_wa) p_csn)(oa_ent u_isn))(u_hdip u_hdip)))	40	9
3686	((ca_ent ca_wa)((((p_re ca_wa) p_csn) oa_ent)((u_isn oa_wa) u_hdip)))	33	9
3687	((ca_ent ca_wa)((((p_re ca_wa) p_csn)(cp_ent oa_ent))(oa_wa u_hdip)))	31	9
3688	((ca_ent ca_wa)((((p_re ca_wa) p_csn)(cp_ent oa_ent))(u_hdip u_hdip)))	34	9
3689	((ca_ent ca_wa)((((p_re ca_wa) p_csn)(cp_ent oa_ent))(u_isn u_hdip)))	37	9
3690	((ca_ent ca_wa)((((p_re ca_wa) p_csn)(oa_ent oa_wa))(u_isn u_hdip)))	34	9
3691	((ca_ent ca_wa)((((p_re ca_wa) p_csn)(oa_ent u_hdip))(u_isn u_hdip)))	36	9
3692	((ca_ent ca_wa)((((p_re ca_wa) p_csn)(oa_ent u_isn))(oa_wa u_hdip)))	36	9
3693	((ca_ent p_isn)((p_re ca_wa)(p_csn ((p_isn oa_ent)(u_isn u_hdip)))))	23	9
3694	((ca_ent p_isn)((p_re ca_wa)((p_csn (oa_ent u_hdip))(u_isn oa_wa))))	23	9
3695	((ca_ent p_isn)((p_re ca_wa)((p_csn (oa_ent u_isn))(u_hdip u_hdip))))	37	9
3696	((ca_ent p_isn)(((ca_wa (p_csn cp_ent))(oa_ent u_isn))(u_hdip u_hdip)))	29	9
3697	((ca_ent p_isn)(((ca_wa (p_csn cp_ent)) oa_ent)((u_isn oa_wa) u_hdip)))	24	9
3698	((ca_ent p_isn)(((ca_wa (p_csn cp_ent))(oa_ent u_isn))(oa_wa u_hdip)))	26	9
3699	((ca_ent p_isn)(((ca_wa (p_csn cp_ent))(oa_ent oa_wa))(u_isn u_hdip)))	25	9
3700	((ca_ent p_isn)(((p_re ca_wa)(p_csn (oa_ent oa_wa)))(u_hdip u_hdip)))	23	9
3701	((ca_ent p_isn)(((p_re ca_wa)(p_csn (oa_ent oa_wa)))(u_isn u_hdip)))	29	9
3702	((ca_ent p_isn)(((p_re ca_wa)(p_csn (oa_ent u_hdip)))(u_isn u_hdip)))	34	9
3703	((ca_ent p_isn)(((p_re ca_wa)(p_csn (oa_ent u_isn)))(oa_wa u_hdip)))	33	9
3704	((ca_ent p_isn)(((p_re ca_wa)(p_csn (oa_ent u_isn)))(u_hdip u_hdip)))	38	9
3705	((ca_ent p_isn)(((p_re ca_wa)(p_csn (p_isn oa_ent)))(u_hdip u_hdip)))	24	9
3706	((ca_ent p_isn)(((p_re ca_wa)(p_csn (p_isn oa_ent)))(u_isn u_hdip)))	27	9
3707	((ca_ent p_isn)(((p_re ca_wa)((cp_ent oa_ent) oa_wa))(u_hdip u_hdip)))	35	9
3708	((ca_ent p_isn)(((p_re ca_wa)((cp_ent oa_ent) oa_wa))(u_isn oa_wa)))	36	9
3709	((ca_ent p_isn)(((p_re ca_wa)((cp_ent oa_ent) oa_wa))(u_isn u_hdip)))	37	9
3710	((ca_ent p_isn)(((p_re ca_wa)((cp_ent oa_ent) u_hdip))(u_isn oa_wa)))	38	9
3711	((ca_ent p_isn)(((p_re ca_wa)((cp_ent oa_ent) u_hdip))(u_isn u_hdip)))	40	9
3712	((ca_ent p_isn)(((p_re ca_wa)((p_csn oa_ent) oa_wa))(u_hdip u_hdip)))	27	9
3713	((ca_ent p_isn)(((p_re ca_wa)((p_csn oa_ent) oa_wa))(u_isn u_hdip)))	28	9
3714	((ca_ent p_isn)(((p_re ca_wa)((p_csn oa_ent) u_hdip))(oa_wa u_hdip)))	28	9
3715	((ca_ent p_isn)(((p_re ca_wa)((p_csn oa_ent) u_hdip))(u_isn u_hdip)))	36	9
3716	((ca_ent p_isn)((((ca_wa p_csn) oa_ent)(u_hdip u_hdip))(u_isn u_hdip)))	25	9
3717	((ca_ent p_isn)((((ca_wa p_csn) oa_ent)(u_isn u_hdip))(u_isn u_hdip)))	27	9
3718	((ca_ent p_isn)((((p_re ca_wa) oa_ent)(u_hdip u_hdip))(u_isn u_hdip)))	32	9
3719	((ca_ent p_isn)((((p_re ca_wa) oa_ent)(u_isn u_hdip))(u_isn u_hdip)))	33	9
3720	((ca_ent p_isn)((((p_re ca_wa) p_csn)(oa_ent u_isn))(u_hdip u_hdip)))	39	9
3721	((ca_ent p_isn)((((p_re ca_wa) p_csn) oa_ent)((u_isn oa_wa) u_hdip)))	29	9
3722	((ca_ent p_isn)((((p_re ca_wa) p_csn)(cp_ent oa_ent))(oa_wa u_hdip)))	29	9
3723	((ca_ent p_isn)((((p_re ca_wa) p_csn)(cp_ent oa_ent))(u_isn u_hdip)))	36	9
3724	((ca_ent p_isn)((((p_re ca_wa) p_csn)(oa_ent oa_wa))(u_hdip u_hdip)))	26	9
3725	((ca_ent p_isn)((((p_re ca_wa) p_csn)(oa_ent oa_wa))(u_isn u_hdip)))	32	9
3726	((ca_ent p_isn)((((p_re ca_wa) p_csn)(oa_ent u_isn))(oa_wa u_hdip)))	35	9
3727	((ca_ent p_isn)((((p_re ca_wa) p_csn)(p_isn oa_ent))(u_isn u_hdip)))	30	9
3728	((ca_wa ca_wa)((p_re p_re)((ca_wa (p_csn cp_ent))(oa_ent u_isn))))	25	9
3729	((ca_wa ca_wa)(((p_csn cp_ent)(oa_ent (u_isn u_hdip)))(u_isn u_hdip)))	30	9
3730	((ca_wa ca_wa)(((p_csn cp_ent)((oa_ent u_isn) u_hdip))(u_isn oa_wa)))	22	9
3731	((ca_wa ca_wa)(((p_csn cp_ent)((oa_ent u_isn) u_hdip))(u_isn u_hdip)))	31	9
3732	((ca_wa ca_wa)((((p_csn cp_ent) oa_ent)(u_isn u_hdip))(u_isn oa_wa)))	22	9
3733	((ca_wa ca_wa)((((p_csn cp_ent) oa_ent)(u_isn u_hdip))(u_isn u_hdip)))	31	9
3734	((ca_wa p_re)(((p_csn cp_ent)(oa_ent (u_isn u_hdip)))(u_isn u_hdip)))	30	9
3735	((ca_wa p_re)(((p_csn cp_ent)((oa_ent u_isn) u_hdip))(u_isn u_hdip)))	31	9
3736	((ca_wa p_re)((((ca_wa p_csn) oa_ent)(u_hdip u_hdip))(u_isn u_hdip)))	28	9
3737	((ca_wa p_re)((((ca_wa p_csn) oa_ent)(u_isn u_hdip))(u_isn u_hdip)))	30	9
3738	((cp_ent ca_ent)((ca_wa ca_wa)((p_csn (oa_ent u_isn))(u_hdip u_hdip))))	29	9
3739	((cp_ent ca_ent)((ca_wa p_re)((p_csn (oa_ent u_isn))(u_hdip u_hdip))))	32	9
3740	((cp_ent ca_ent)(((p_isn ca_wa)((p_csn cp_ent) oa_ent))(oa_wa u_hdip)))	26	9
3741	((cp_ent ca_ent)((p_isn p_re)((ca_wa (p_csn cp_ent))(oa_ent u_isn))))	34	9
3742	((cp_ent ca_ent)(((ca_wa p_re) ca_wa)(p_csn (oa_ent (u_isn u_hdip)))))	30	9
3743	((cp_ent ca_ent)(((ca_wa p_re) ca_wa)(p_csn ((oa_ent oa_wa) u_hdip))))	28	9
3744	((cp_ent ca_ent)(((ca_wa p_re) ca_wa)(p_csn ((p_isn oa_ent) u_hdip))))	24	9
3745	((cp_ent ca_ent)((p_re ca_wa)(p_csn ((p_isn oa_ent)(u_isn u_hdip)))))	23	9
3746	((cp_ent ca_ent)((p_re ca_wa)((p_csn (oa_ent u_hdip))(u_isn oa_wa))))	23	9
3747	((cp_ent ca_ent)((p_re ca_wa)((p_csn (oa_ent u_isn))(u_hdip u_hdip))))	35	9
3748	((cp_ent ca_ent)(((ca_wa p_re) ca_wa)(p_csn (oa_ent (oa_wa u_hdip)))))	27	9
3749	((cp_ent ca_ent)(((ca_wa p_re) ca_wa)((p_csn (oa_ent u_isn)) u_hdip)))	34	9
3750	((cp_ent ca_ent)(((ca_wa p_re) ca_wa)((p_csn (p_isn oa_ent)) u_hdip)))	27	9
3751	((cp_ent ca_ent)(((ca_wa ca_wa)(p_csn (oa_ent u_isn)))(u_hdip u_hdip)))	28	9
3752	((cp_ent ca_ent)(((ca_wa p_re)(p_csn (oa_ent u_isn)))(u_hdip u_hdip)))	33	9
3753	((cp_ent ca_ent)(((p_re ca_wa)(p_csn (oa_ent u_isn)))(u_hdip u_hdip)))	36	9
3754	((cp_ent ca_ent)((((ca_wa p_re) ca_wa)(p_csn oa_ent))(u_hdip u_hdip)))	34	9
3755	((cp_ent ca_ent)(((p_re ca_wa)((cp_ent oa_ent) oa_wa))(u_hdip u_hdip)))	30	9
3756	((cp_ent ca_ent)(((p_re ca_wa)((cp_ent oa_ent) oa_wa))(u_isn u_hdip)))	35	9
3757	((cp_ent ca_ent)(((p_re ca_wa)((cp_ent oa_ent) u_hdip))(u_isn oa_wa)))	36	9
3758	((cp_ent ca_ent)(((p_re ca_wa)((cp_ent oa_ent) u_hdip))(u_isn u_hdip)))	38	9
3759	((cp_ent ca_ent)(((p_re ca_wa)((p_csn oa_ent) oa_wa))(u_hdip u_hdip)))	25	9
3760	((cp_ent ca_ent)(((p_re ca_wa)((p_csn oa_ent) oa_wa))(u_isn u_hdip)))	26	9
3761	((cp_ent ca_ent)(((p_re ca_wa)((p_csn oa_ent) u_hdip))(u_isn u_hdip)))	32	9
3762	((cp_ent ca_ent)((((p_isn ca_wa) p_csn)(oa_ent oa_wa))(u_hdip u_hdip)))	21	9
3763	((cp_ent ca_ent)((((p_isn ca_wa) p_csn)(oa_ent oa_wa))(u_isn u_hdip)))	21	9
3764	((cp_ent ca_ent)((((ca_wa p_re) ca_wa)(cp_ent oa_ent))(oa_wa u_hdip)))	41	9
3765	((cp_ent ca_ent)((((ca_wa p_re) ca_wa)(cp_ent oa_ent))(u_hdip u_hdip)))	42	9
3766	((cp_ent ca_ent)((((ca_wa p_re) ca_wa)(cp_ent oa_ent))(u_isn oa_wa)))	37	9
3767	((cp_ent ca_ent)((((ca_wa p_re) ca_wa)(cp_ent oa_ent))(u_isn u_hdip)))	42	9
3768	((cp_ent ca_ent)((((ca_wa p_re) ca_wa)(oa_ent u_isn))(u_hdip u_hdip)))	34	9
3769	((cp_ent ca_ent)((((ca_wa p_re) ca_wa)(p_csn oa_ent))(u_isn u_hdip)))	36	9
3770	((cp_ent ca_ent)((((ca_wa p_re) cp_ent)(oa_ent u_hdip))(u_isn u_hdip)))	40	9
3771	((cp_ent ca_ent)((((ca_wa p_re) cp_ent)(oa_ent u_isn))(u_hdip u_hdip)))	40	9
3772	((cp_ent ca_ent)((((ca_wa p_re) cp_ent) oa_ent)((u_isn oa_wa) u_hdip)))	34	9
3773	((cp_ent ca_ent)((((ca_wa p_re) cp_ent)(oa_ent oa_wa))(u_isn oa_wa)))	37	9
3774	((cp_ent ca_ent)((((ca_wa p_re) cp_ent)(oa_ent oa_wa))(u_isn u_hdip)))	39	9
3775	((cp_ent ca_ent)((((ca_wa p_re) cp_ent)(oa_ent u_hdip))(u_isn oa_wa)))	34	9
3776	((cp_ent ca_ent)((((p_isn ca_wa) p_csn) oa_ent)((oa_wa u_hdip) oa_wa)))	23	9
3777	((cp_ent ca_ent)((((p_isn ca_wa) p_csn)(p_isn oa_ent))(u_hdip u_hdip)))	21	9
3778	((cp_ent ca_ent)((((p_isn ca_wa) p_csn)(p_isn oa_ent))(u_isn u_hdip)))	22	9
3779	((cp_ent ca_ent)((((p_re ca_wa) p_csn)(oa_ent u_isn))(u_hdip u_hdip)))	37	9
3780	((cp_ent ca_ent)((((p_re ca_wa) p_csn) oa_ent)((u_isn oa_wa) u_hdip)))	27	9
3781	((cp_ent ca_ent)((((p_re ca_wa) p_csn)(cp_ent oa_ent))(oa_wa u_hdip)))	27	9
3782	((cp_ent ca_ent)((((p_re ca_wa) p_csn)(cp_ent oa_ent))(u_hdip u_hdip)))	30	9
3783	((cp_ent ca_ent)((((p_re ca_wa) p_csn)(cp_ent oa_ent))(u_isn u_hdip)))	35	9
3784	((cp_ent ca_ent)((((p_re ca_wa) p_csn)(oa_ent oa_wa))(u_hdip u_hdip)))	24	9
3785	((cp_ent ca_ent)((((p_re ca_wa) p_csn)(oa_ent oa_wa))(u_isn u_hdip)))	28	9
3786	((cp_ent ca_ent)((((p_re ca_wa) p_csn)(oa_ent u_hdip))(u_isn u_hdip)))	31	9
3787	((cp_ent ca_ent)((((p_re ca_wa) p_csn)(oa_ent u_isn))(oa_wa u_hdip)))	30	9
3788	((cp_ent ca_ent)((((p_re ca_wa) p_csn)(p_isn oa_ent))(u_isn u_hdip)))	28	9
3789	((cp_ent ca_wa)((p_re ca_wa)(p_csn ((p_isn oa_ent)(u_isn u_hdip)))))	22	9
3790	((cp_ent ca_wa)((p_re ca_wa)((p_csn (oa_ent u_isn))(u_hdip u_hdip))))	34	9
3791	((cp_ent ca_wa)(((p_re ca_wa)(p_csn (oa_ent u_hdip)))(u_isn u_hdip)))	30	9
3792	((cp_ent ca_wa)(((p_re ca_wa)(p_csn (oa_ent u_isn)))(oa_wa u_hdip)))	30	9
3793	((cp_ent ca_wa)(((p_re ca_wa)(p_csn (p_isn oa_ent)))(u_isn u_hdip)))	25	9
3794	((cp_ent ca_wa)(((p_re ca_wa)((cp_ent oa_ent) oa_wa))(u_hdip u_hdip)))	31	9
3795	((cp_ent ca_wa)(((p_re ca_wa)((cp_ent oa_ent) oa_wa))(u_isn oa_wa)))	33	9
3796	((cp_ent ca_wa)(((p_re ca_wa)((cp_ent oa_ent) oa_wa))(u_isn u_hdip)))	32	9
3797	((cp_ent ca_wa)(((p_re ca_wa)((cp_ent oa_ent) u_hdip))(u_isn oa_wa)))	33	9
3798	((cp_ent ca_wa)(((p_re ca_wa)((cp_ent oa_ent) u_hdip))(u_isn u_hdip)))	36	9
3799	((cp_ent ca_wa)(((p_re ca_wa)((p_csn oa_ent) u_hdip))(u_isn u_hdip)))	32	9
3800	((cp_ent ca_wa)((((p_re ca_wa) oa_ent)(u_hdip u_hdip))(u_isn u_hdip)))	29	9
3801	((cp_ent ca_wa)((((p_re ca_wa) oa_ent)(u_isn u_hdip))(u_isn u_hdip)))	30	9
3802	((cp_ent ca_wa)((((p_re ca_wa) p_csn)(oa_ent u_isn))(u_hdip u_hdip)))	35	9
3803	((cp_ent ca_wa)((((p_re ca_wa) p_csn)(oa_ent u_hdip))(u_isn u_hdip)))	31	9
3804	((cp_ent ca_wa)((((p_re ca_wa) p_csn)(oa_ent u_isn))(oa_wa u_hdip)))	31	9
3805	((cp_ent oa_ent)((oa_wa u_hdip)((u_isn u_hdip)(oa_wa (u_csn cp_ent)))))	33	9
3806	((cp_ent oa_ent)((u_isn u_hdip)((u_isn u_hdip)(oa_wa (u_csn cp_ent)))))	34	9
3807	((cp_ent oa_ent)((u_isn u_hdip)((u_isn u_hdip)((u_isn oa_wa) u_csn))))	37	9
3808	((oa_ent oa_wa)((u_isn u_hdip)((u_isn u_hdip)(oa_wa (u_csn cp_ent)))))	34	9
3809	((oa_ent oa_wa)((u_isn u_hdip)((u_isn u_hdip)((u_isn oa_wa) u_csn))))	36	9
3810	((oa_ent u_hdip)((u_isn u_hdip)((u_isn u_hdip)((u_isn oa_wa) u_csn))))	35	9
3811	((oa_wa oa_wa)(((u_csn cp_ent)(p_csn (p_isn ca_ent)))(ca_wa ca_wa)))	16	9
3812	((oa_wa oa_wa)((((u_csn cp_ent) p_csn)(ca_ent p_isn))(p_re ca_wa)))	22	9
3813	((oa_wa oa_wa)((((u_csn cp_ent) p_csn)(p_isn ca_ent))(ca_wa ca_wa)))	18	9
3814	((oa_wa u_hdip)((u_isn oa_wa)(((u_csn cp_ent) p_csn)(p_isn ca_wa))))	14	9
3815	((oa_wa u_hdip)((u_isn u_hdip)((oa_wa u_csn)((cp_ent oa_ent) oa_wa))))	21	9
3816	((oa_wa u_hdip)((((u_csn cp_ent) p_csn)(ca_ent p_isn))(p_re ca_wa)))	26	9
3817	((oa_wa u_hdip)((((u_csn cp_ent) p_csn) ca_ent)((ca_wa p_re) ca_wa)))	26	9
3818	((oa_wa u_hdip)((((u_isn oa_wa) u_csn)(cp_ent ca_wa))(p_re ca_wa)))	17	9
3819	((p_csn ca_ent)((((ca_wa p_re) ca_wa)(cp_ent oa_ent))(oa_wa u_hdip)))	29	9
3820	((p_csn ca_ent)((((ca_wa p_re) ca_wa)(cp_ent oa_ent))(u_hdip u_hdip)))	31	9
3821	((p_csn ca_ent)((((ca_wa p_re) ca_wa)(cp_ent oa_ent))(u_isn oa_wa)))	27	9
3822	((p_csn ca_ent)((((ca_wa p_re) ca_wa)(cp_ent oa_ent))(u_isn u_hdip)))	29	9
3823	((p_csn ca_ent)((((ca_wa p_re) ca_wa)(oa_ent u_isn))(u_hdip u_hdip)))	27	9
3824	((p_csn ca_ent)((((ca_wa p_re) cp_ent)(oa_ent u_hdip))(u_isn u_hdip)))	30	9
3825	((p_isn ca_ent)((ca_wa ca_wa)((p_csn (oa_ent u_isn))(u_hdip u_hdip))))	27	9
3826	((p_isn ca_ent)((ca_wa p_re)(p_csn ((p_isn oa_ent)(u_isn u_hdip)))))	23	9
3827	((p_isn ca_ent)((ca_wa p_re)((p_csn (oa_ent u_isn))(u_hdip u_hdip))))	29	9
3828	((p_isn ca_ent)((p_isn p_re)((ca_wa (p_csn cp_ent))(oa_ent oa_wa))))	22	9
3829	((p_isn ca_ent)(((ca_wa p_re) ca_wa)(p_csn (oa_ent (u_isn u_hdip)))))	28	9
3830	((p_isn ca_ent)(((ca_wa p_re) ca_wa)(p_csn ((p_isn oa_ent) u_hdip))))	25	9
3831	((p_isn ca_ent)((p_re ca_wa)(p_csn ((p_isn oa_ent)(u_isn u_hdip)))))	24	9
3832	((p_isn ca_ent)((p_re ca_wa)((p_csn (oa_ent u_hdip))(u_isn oa_wa))))	19	9
3833	((p_isn ca_ent)(((ca_wa p_re) ca_wa)(p_csn (oa_ent (oa_wa u_hdip)))))	21	9
3834	((p_isn ca_ent)(((ca_wa p_re) ca_wa)(p_csn ((oa_ent oa_wa) u_hdip))))	22	9
3835	((p_isn ca_ent)(((ca_wa ca_wa)(p_csn (oa_ent u_isn)))(u_hdip u_hdip)))	26	9
3836	((p_isn ca_ent)(((ca_wa p_re)(p_csn (oa_ent u_hdip)))(u_isn u_hdip)))	25	9
3837	((p_isn ca_ent)(((ca_wa p_re)(p_csn (oa_ent u_isn)))(oa_wa u_hdip)))	26	9
3838	((p_isn ca_ent)(((ca_wa p_re)(p_csn (oa_ent u_isn)))(u_hdip u_hdip)))	30	9
3839	((p_isn ca_ent)(((ca_wa p_re)((p_csn oa_ent) u_hdip))(u_isn u_hdip)))	27	9
3840	((p_isn ca_ent)(((p_re ca_wa)((cp_ent oa_ent) oa_wa))(u_hdip u_hdip)))	28	9
3841	((p_isn ca_ent)(((p_re ca_wa)((cp_ent oa_ent) oa_wa))(u_isn u_hdip)))	30	9
3842	((p_isn ca_ent)(((p_re ca_wa)((p_csn oa_ent) oa_wa))(u_hdip u_hdip)))	21	9
3843	((p_isn ca_ent)(((p_re ca_wa)((p_csn oa_ent) oa_wa))(u_isn u_hdip)))	22	9
3844	((p_isn ca_ent)(((p_re ca_wa)((p_csn oa_ent) u_hdip))(oa_wa u_hdip)))	23	9
3845	((p_isn ca_ent)((((ca_wa p_re) ca_wa)(cp_ent oa_ent))(oa_wa u_hdip)))	34	9
3846	((p_isn ca_ent)((((ca_wa p_re) ca_wa)(cp_ent oa_ent))(u_hdip u_hdip)))	37	9
3847	((p_isn ca_ent)((((ca_wa p_re) ca_wa)(cp_ent oa_ent))(u_isn oa_wa)))	33	9
3848	((p_isn ca_ent)((((ca_wa p_re) ca_wa)(cp_ent oa_ent))(u_isn u_hdip)))	37	9
3849	((p_isn ca_ent)((((ca_wa p_re) ca_wa)(oa_ent u_isn))(u_hdip u_hdip)))	31	9
3850	((p_isn ca_ent)((((ca_wa p_re) ca_wa)(p_csn oa_ent))(oa_wa u_hdip)))	26	9
3851	((p_isn ca_ent)((((ca_wa p_re) cp_ent)(oa_ent u_hdip))(u_isn u_hdip)))	35	9
3852	((p_isn ca_ent)((((ca_wa p_re) cp_ent) oa_ent)((u_isn oa_wa) u_hdip)))	31	9
3853	((p_isn ca_ent)((((ca_wa p_re) cp_ent)(oa_ent oa_wa))(u_hdip u_hdip)))	27	9
3854	((p_isn ca_ent)((((ca_wa p_re) cp_ent)(oa_ent oa_wa))(u_isn oa_wa)))	32	9
3855	((p_isn ca_ent)((((ca_wa p_re) cp_ent)(oa_ent oa_wa))(u_isn u_hdip)))	34	9
3856	((p_isn ca_ent)((((ca_wa p_re) cp_ent)(oa_ent u_hdip))(u_isn oa_wa)))	31	9
3857	((p_isn ca_ent)((((ca_wa p_re) cp_ent)(p_isn oa_ent))(u_hdip u_hdip)))	20	9
3858	((p_isn ca_ent)((((p_isn ca_wa) p_csn)(p_isn oa_ent))(u_hdip u_hdip)))	20	9
3859	((p_isn ca_ent)((((p_re ca_wa) oa_ent)(u_hdip u_hdip))(u_isn u_hdip)))	26	9
3860	((p_isn ca_ent)((((p_re ca_wa) oa_ent)(u_isn u_hdip))(u_isn u_hdip)))	27	9
3861	((p_isn ca_ent)((((p_re ca_wa) p_csn)(oa_ent u_isn))(u_hdip u_hdip)))	32	9
3862	((p_isn ca_ent)((((p_re ca_wa) p_csn) oa_ent)((u_isn oa_wa) u_hdip)))	23	9
3863	((p_isn ca_ent)((((p_re ca_wa) p_csn)(cp_ent oa_ent))(u_hdip u_hdip)))	26	9
3864	((p_isn ca_ent)((((p_re ca_wa) p_csn)(cp_ent oa_ent))(u_isn u_hdip)))	27	9
3865	((p_isn ca_ent)((((p_re ca_wa) p_csn)(oa_ent oa_wa))(u_hdip u_hdip)))	20	9
3866	((p_isn ca_ent)((((p_re ca_wa) p_csn)(oa_ent oa_wa))(u_isn u_hdip)))	22	9
3867	((p_isn ca_ent)((((p_re ca_wa) p_csn)(oa_ent u_hdip))(u_isn u_hdip)))	29	9
3868	((p_isn ca_ent)((((p_re ca_wa) p_csn)(oa_ent u_isn))(oa_wa u_hdip)))	24	9
3869	((p_isn ca_ent)((((p_re ca_wa) p_csn)(p_isn oa_ent))(u_isn u_hdip)))	27	9
3870	((p_isn ca_wa)((p_isn p_re)((((ca_wa p_csn) oa_ent)(u_hdip u_hdip)))))	29	9
3871	((p_isn ca_wa)((p_isn p_re)((((ca_wa p_csn) oa_ent)(u_isn u_hdip)))))	30	9
3872	((p_isn ca_wa)((p_re ca_wa)(p_csn ((p_isn oa_ent)(u_isn u_hdip)))))	24	9
3873	((p_isn ca_wa)((p_re ca_wa)((p_csn (oa_ent u_hdip))(u_isn oa_wa))))	27	9
3874	((p_isn ca_wa)((p_re ca_wa)((p_csn (oa_ent u_isn))(u_hdip u_hdip))))	34	9
3875	((p_isn ca_wa)(((p_csn cp_ent)(oa_ent (u_isn u_hdip)))(u_isn u_hdip)))	30	9
3876	((p_isn ca_wa)(((p_csn cp_ent)((oa_ent u_isn) u_hdip))(u_isn u_hdip)))	31	9
3877	((p_isn ca_wa)(((p_re ca_wa)(p_csn (oa_ent oa_wa)))(u_hdip u_hdip)))	27	9
3878	((p_isn ca_wa)(((p_re ca_wa)(p_csn (oa_ent oa_wa)))(u_isn u_hdip)))	31	9
3879	((p_isn ca_wa)(((p_re ca_wa)(p_csn (oa_ent u_hdip)))(u_isn u_hdip)))	33	9
3880	((p_isn ca_wa)(((p_re ca_wa)(p_csn (oa_ent u_isn)))(u_hdip u_hdip)))	35	9
3881	((p_isn ca_wa)(((p_re ca_wa)(p_csn (p_isn oa_ent)))(u_hdip u_hdip)))	26	9
3882	((p_isn ca_wa)(((p_re ca_wa)(p_csn (p_isn oa_ent)))(u_isn u_hdip)))	29	9
3883	((p_isn ca_wa)(((p_re ca_wa)((cp_ent oa_ent) oa_wa))(u_hdip u_hdip)))	34	9
3884	((p_isn ca_wa)(((p_re ca_wa)((cp_ent oa_ent) oa_wa))(u_isn oa_wa)))	35	9
3885	((p_isn ca_wa)(((p_re ca_wa)((cp_ent oa_ent) oa_wa))(u_isn u_hdip)))	36	9
3886	((p_isn ca_wa)(((p_re ca_wa)((cp_ent oa_ent) u_hdip))(u_isn oa_wa)))	37	9
3887	((p_isn ca_wa)(((p_re ca_wa)((cp_ent oa_ent) u_hdip))(u_isn u_hdip)))	39	9
3888	((p_isn ca_wa)(((p_re ca_wa)((p_csn oa_ent) oa_wa))(u_hdip u_hdip)))	29	9
3889	((p_isn ca_wa)(((p_re ca_wa)((p_csn oa_ent) oa_wa))(u_isn u_hdip)))	30	9
3890	((p_isn ca_wa)((((p_csn cp_ent) oa_ent)(u_isn u_hdip))(u_isn u_hdip)))	31	9
3891	((p_isn ca_wa)((((p_re ca_wa) oa_ent)(oa_wa u_hdip))(u_isn oa_wa)))	25	9
3892	((p_isn ca_wa)((((p_re ca_wa) oa_ent)(oa_wa u_hdip))(u_isn u_hdip)))	28	9
3893	((p_isn ca_wa)((((p_re ca_wa) oa_ent)(u_hdip u_hdip))(u_isn u_hdip)))	31	9
3894	((p_isn ca_wa)((((p_re ca_wa) oa_ent)(u_isn u_hdip))(u_isn oa_wa)))	28	9
3895	((p_isn ca_wa)((((p_re ca_wa) oa_ent)(u_isn u_hdip))(u_isn u_hdip)))	32	9
3896	((p_isn ca_wa)((((p_re ca_wa) p_csn)(oa_ent u_isn))(u_hdip u_hdip)))	36	9
3897	((p_isn ca_wa)((((p_re ca_wa) p_csn)(oa_ent oa_wa))(u_hdip u_hdip)))	28	9
3898	((p_isn ca_wa)((((p_re ca_wa) p_csn)(oa_ent oa_wa))(u_isn u_hdip)))	32	9
3899	((p_isn ca_wa)((((p_re ca_wa) p_csn)(oa_ent u_hdip))(u_isn u_hdip)))	34	9
3900	((p_isn ca_wa)((((p_re ca_wa) p_csn)(p_isn oa_ent))(u_isn u_hdip)))	30	9
3901	((p_isn oa_ent)((oa_wa u_hdip)((u_isn u_hdip)(oa_wa (u_csn cp_ent)))))	26	9
3902	((p_isn p_re)(((ca_wa (p_csn cp_ent))(oa_ent u_isn))(oa_wa u_hdip)))	29	9
3903	((p_isn p_re)(((ca_wa (p_csn cp_ent))(oa_ent oa_wa))(u_isn u_hdip)))	28	9
3904	((p_isn p_re)(((ca_wa (p_csn cp_ent))(oa_ent u_hdip))(u_isn u_hdip)))	30	9
3905	((p_isn p_re)((((ca_wa p_csn) oa_ent)(u_isn u_hdip))(u_isn u_hdip)))	29	9
3906	((p_re ca_wa)((p_csn ((p_isn oa_ent)(u_isn u_hdip)))(u_isn u_hdip)))	24	9
3907	((p_re ca_wa)(((p_csn cp_ent)(oa_ent (u_isn u_hdip)))(u_isn u_hdip)))	30	9
3908	((p_re ca_wa)(((p_csn cp_ent)((oa_ent u_isn) u_hdip))(u_isn oa_wa)))	22	9
3909	((u_csn (cp_ent ca_ent))((ca_wa p_re)((p_csn (oa_ent u_isn)) u_hdip)))	22	9
3910	((u_csn (cp_ent ca_ent))((ca_wa p_re)((p_csn (p_isn oa_ent)) u_hdip)))	19	9
3911	((u_csn (cp_ent ca_ent))((((ca_wa p_re) ca_wa) oa_ent)(oa_wa u_hdip)))	22	9
3912	((u_csn (cp_ent ca_ent))((((ca_wa p_re) ca_wa) oa_ent)(u_hdip u_hdip)))	26	9
3913	((u_csn (cp_ent ca_ent))((((ca_wa p_re) ca_wa) oa_ent)(u_isn u_hdip)))	26	9
3914	((u_csn (cp_ent ca_ent))((((ca_wa p_re) cp_ent) oa_ent)(oa_wa oa_wa)))	26	9
3915	((u_csn (cp_ent ca_ent))((((ca_wa p_re) cp_ent) oa_ent)(u_isn oa_wa)))	28	9
3916	((u_csn (cp_ent ca_ent))((((ca_wa p_re) cp_ent) oa_ent)(u_isn u_hdip)))	33	9
3917	((u_csn (cp_ent ca_ent))((((p_re ca_wa) p_csn) oa_ent)(u_hdip u_hdip)))	28	9
3918	((u_csn (cp_ent ca_ent))((((p_re ca_wa) p_csn) oa_ent)(u_isn u_hdip)))	29	9
3919	((u_csn cp_ent)((((ca_ent ca_wa) cp_ent)(oa_ent u_hdip))(u_isn oa_wa)))	29	9
3920	((u_hdip oa_wa)(((u_csn (oa_ent oa_wa))(u_isn u_hdip))(u_isn u_hdip)))	23	9
3921	((u_hdip oa_wa)((((u_csn cp_ent) p_csn)(ca_ent p_isn))(p_re ca_wa)))	27	9
3922	((u_hdip oa_wa)((((u_csn cp_ent) p_csn) ca_ent)((ca_wa p_re) ca_wa)))	27	9
3923	((u_hdip u_hdip)((u_isn u_hdip)((u_isn u_hdip)((u_isn oa_wa) u_csn))))	26	9
3924	((u_hdip u_hdip)(((u_isn oa_wa)((u_csn cp_ent) p_csn))(ca_ent p_isn)))	26	9
3925	((u_hdip u_hdip)(((u_isn oa_wa)((u_csn cp_ent) p_csn))(ca_ent ca_wa)))	26	9
3926	((u_hdip u_hdip)(((u_isn oa_wa)((u_csn cp_ent) p_csn))(ca_wa ca_wa)))	20	9
3927	((u_hdip u_hdip)((((u_isn oa_wa) u_csn)(ca_ent ca_wa))(ca_wa p_csn)))	19	9
3928	((u_hdip u_hdip)((((u_isn oa_wa) u_csn)(cp_ent ca_ent))(ca_wa ca_wa)))	27	9
3929	((u_hdip u_hdip)((((u_isn oa_wa) u_csn)(cp_ent ca_ent))(ca_wa p_re)))	27	9
3930	((u_hdip u_hdip)((((u_isn oa_wa) u_csn)(cp_ent ca_ent))(p_re ca_wa)))	27	9
3931	((u_hdip u_hdip)((((u_isn oa_wa) u_csn)(cp_ent ca_ent))(p_isn ca_wa)))	21	9
3932	((u_hdip u_hdip)((((u_isn oa_wa) u_csn)(cp_ent ca_wa))(p_re ca_wa)))	21	9
3933	((u_isn u_hdip)((u_isn u_hdip)((oa_wa u_csn)((cp_ent oa_ent) oa_wa))))	25	9
3934	((u_isn u_hdip)((u_isn u_hdip)((u_isn u_hdip)(oa_wa (u_csn cp_ent)))))	31	9
3935	((u_isn u_hdip)((u_isn u_hdip)((u_isn u_hdip)((u_isn oa_wa) u_csn))))	34	9
3936	((u_isn u_hdip)(((u_isn oa_wa)((u_csn cp_ent) p_csn))(ca_ent p_isn)))	26	9
3937	((u_isn u_hdip)(((u_isn oa_wa)((u_csn cp_ent) p_csn))(ca_ent ca_wa)))	26	9
3938	((u_isn u_hdip)(((u_isn oa_wa)((u_csn cp_ent) p_csn))(ca_wa ca_wa)))	22	9
3939	((u_isn u_hdip)(((u_isn u_hdip)(oa_wa (u_csn cp_ent)))(oa_ent u_isn)))	33	9
3940	((u_isn u_hdip)(((u_isn u_hdip)(oa_wa (u_csn cp_ent)))(oa_wa u_hdip)))	16	9
3941	((u_isn u_hdip)(((u_isn u_hdip)((oa_wa u_csn) oa_ent))(oa_wa u_hdip)))	18	9
3942	((u_isn u_hdip)((((u_isn oa_wa) u_csn)(ca_ent ca_wa))(ca_wa p_csn)))	21	9
3943	((u_isn u_hdip)((((u_isn oa_wa) u_csn)(cp_ent ca_ent))(ca_wa ca_wa)))	26	9
3944	((u_isn u_hdip)((((u_isn oa_wa) u_csn)(cp_ent ca_ent))(ca_wa p_re)))	26	9
3945	((u_isn u_hdip)((((u_isn oa_wa) u_csn)(cp_ent ca_ent))(p_re ca_wa)))	26	9
3946	((u_isn u_hdip)((((u_isn oa_wa) u_csn)(cp_ent ca_wa))(p_re ca_wa)))	21	9
3947	((u_isn u_hdip)(((u_isn u_hdip)((oa_wa u_csn) oa_ent))(oa_wa u_hdip)))	19	9
3948	((oa_ent (oa_wa u_hdip))(((u_isn u_hdip)(oa_wa (u_csn cp_ent))) oa_ent))	26	9
3949	((oa_ent (u_isn u_hdip))(((u_isn u_hdip)(oa_wa (u_csn cp_ent))) oa_ent))	29	9
3950	((p_csn (p_isn ca_ent))((((ca_wa p_re) ca_wa) oa_ent)(u_isn u_hdip)))	18	9
3951	((u_csn (p_isn ca_ent))((((ca_wa p_re) ca_wa) oa_ent)(u_hdip u_hdip)))	22	9
3952	((u_csn (p_isn ca_ent))((((ca_wa p_re) ca_wa) oa_ent)(u_isn u_hdip)))	22	9
3953	((u_csn (p_isn ca_ent))((((ca_wa p_re) cp_ent) oa_ent)(oa_wa oa_wa)))	21	9
3954	((u_csn (p_isn ca_ent))((((ca_wa p_re) cp_ent) oa_ent)(u_isn oa_wa)))	22	9
3955	((u_csn (p_isn ca_ent))((((ca_wa p_re) cp_ent) oa_ent)(u_isn u_hdip)))	27	9
3956	((u_csn (p_isn ca_ent))((((p_re ca_wa) p_csn) oa_ent)(u_hdip u_hdip)))	22	9
3957	((u_csn (p_isn ca_ent))((((p_re ca_wa) p_csn) oa_ent)(u_isn u_hdip)))	23	9
3958	((u_hdip (u_isn oa_wa))((((u_csn cp_ent) p_csn) ca_ent)(ca_wa ca_wa)))	27	9
3959	((u_hdip (u_isn oa_wa))((((u_csn cp_ent) p_csn) ca_ent)(ca_wa p_re)))	27	9
3960	(((ca_ent ca_wa) cp_ent)((p_csn (oa_ent (u_isn u_hdip)))(u_isn oa_wa)))	25	9
3961	(((ca_wa p_re) ca_wa)((p_csn (oa_ent (u_isn u_hdip)))(u_isn oa_wa)))	29	9
3962	(((ca_wa p_re) ca_wa)((p_csn (oa_ent (u_isn u_hdip)))(u_isn u_hdip)))	33	9
3963	(((ca_wa p_re) ca_wa)(((p_csn (oa_ent u_isn)) u_hdip)(oa_wa u_hdip)))	30	9
3964	(((ca_wa p_re) cp_ent)(((p_csn (oa_ent u_isn)) u_hdip)(oa_wa u_hdip)))	21	9
3965	(((oa_ent u_isn) u_hdip)((u_isn u_hdip)((oa_wa (u_csn cp_ent)) oa_ent)))	21	9
3966	(((oa_wa u_hdip) oa_wa)((((u_csn cp_ent) p_csn) ca_ent)(ca_wa ca_wa)))	24	9
3967	(((oa_wa u_hdip) oa_wa)((((u_csn cp_ent) p_csn) ca_ent)(ca_wa p_re)))	24	9
3968	(((u_hdip oa_wa) u_hdip)((((u_csn cp_ent) p_csn) ca_ent)(ca_wa ca_wa)))	20	9
3969	(((u_hdip oa_wa) u_hdip)((((u_csn cp_ent) p_csn) ca_ent)(ca_wa p_re)))	20	9
3970	(((u_isn cp_ent) ca_ent)((((ca_wa p_re) cp_ent) oa_ent)(u_hdip u_hdip)))	22	9
3971	(((u_isn cp_ent) ca_ent)((((ca_wa p_re) cp_ent) oa_ent)(u_isn oa_wa)))	19	9
3972	(((u_isn cp_ent) ca_ent)((((ca_wa p_re) cp_ent) oa_ent)(u_isn u_hdip)))	23	9
3973	(((u_isn oa_wa) u_hdip)((u_isn u_hdip)((oa_wa (u_csn cp_ent)) oa_ent)))	24	9
3974	(((u_isn oa_wa) u_hdip)((u_isn u_hdip)(((oa_wa u_csn) oa_ent) oa_wa)))	19	9
3975	(((u_isn oa_wa) u_hdip)(((u_isn oa_wa)((u_csn cp_ent) p_csn)) ca_ent))	17	9
3976	(((u_isn oa_wa) u_hdip)((((u_csn cp_ent) p_csn) ca_ent)(ca_wa ca_wa)))	28	9
3977	(((u_isn oa_wa) u_hdip)((((u_csn cp_ent) p_csn) ca_ent)(ca_wa p_re)))	28	9
3978	((oa_ent ((oa_wa u_hdip)((u_isn u_hdip)(oa_wa u_csn))))(cp_ent oa_ent))	25	9
3979	((oa_ent ((oa_wa u_hdip)((u_isn u_hdip)(oa_wa u_csn))))(oa_ent oa_wa))	17	9
3980	((p_csn ((ca_ent ca_wa)((p_re ca_wa)(cp_ent oa_ent))))(u_hdip u_hdip))	26	9
3981	((p_csn ((ca_ent ca_wa)((p_re ca_wa)(cp_ent oa_ent))))(u_isn u_hdip))	24	9
3982	((p_csn ((p_isn ca_wa)((p_re ca_wa)(cp_ent oa_ent))))(u_hdip u_hdip))	22	9
3983	((p_csn ((p_isn ca_wa)((p_re ca_wa)(cp_ent oa_ent))))(u_isn u_hdip))	21	9
3984	(((ca_ent ca_wa)((p_re ca_wa)(p_csn (oa_ent oa_wa))))(u_hdip u_hdip))	29	9
3985	(((ca_ent p_isn)((p_re ca_wa)((cp_ent oa_ent) oa_wa)))(u_isn oa_wa))	37	9
3986	(((p_isn p_re)(((ca_wa p_csn) oa_ent)(u_hdip u_hdip)))(u_isn u_hdip))	27	9
3987	(((ca_ent ca_wa)(((p_re ca_wa) oa_ent)(oa_wa u_hdip)))(u_isn u_hdip))	30	9
3988	(((ca_ent ca_wa)(((p_re ca_wa) p_csn)(cp_ent oa_ent)))(u_isn oa_wa))	32	9
3989	(((ca_ent ca_wa)(((p_re ca_wa) p_csn)(oa_ent oa_wa)))(u_hdip u_hdip))	30	9
3990	((((ca_wa p_re)(ca_wa (p_csn cp_ent)))(oa_ent oa_wa))(u_isn oa_wa))	27	9
3991	((((ca_wa p_re)(ca_wa (p_csn cp_ent)))(oa_ent oa_wa))(u_isn u_hdip))	30	9
3992	((((ca_wa p_re)(ca_wa (p_csn cp_ent)))(oa_ent u_hdip))(u_isn u_hdip))	32	9
3993	((((ca_wa p_re) ca_wa)(p_csn oa_ent))(oa_wa u_hdip))(u_isn u_hdip))	27	9
3994	((((ca_wa p_re) ca_wa)(p_csn oa_ent))(u_hdip u_hdip))(u_isn u_hdip))	33	9
3995	((((ca_wa p_re) ca_wa)(p_csn oa_ent))(u_isn u_hdip))(oa_wa u_hdip))	32	9
3996	((((ca_wa p_re) ca_wa)(p_csn oa_ent))(u_isn u_hdip))(u_isn oa_wa))	34	9
3997	((((ca_wa p_re) ca_wa)(p_csn oa_ent))(u_isn u_hdip))(u_isn u_hdip))	37	9
3998	(((ca_ent p_isn)((p_re ca_wa)(p_csn (oa_ent oa_wa))))(u_hdip u_hdip))	28	9
3999	(((ca_ent p_isn)((p_re ca_wa)(p_csn (oa_ent oa_wa))))(u_isn u_hdip))	32	9
4000	(((ca_ent p_isn)((p_re ca_wa)((p_csn oa_ent) oa_wa)))(u_hdip u_hdip))	29	9
4001	(((ca_ent p_isn)((p_re ca_wa)((p_csn oa_ent) oa_wa)))(u_isn u_hdip))	30	9
4002	(((ca_ent p_isn)((p_re ca_wa)((p_csn oa_ent) u_hdip)))(oa_wa u_hdip))	31	9
4003	(((ca_ent p_isn)((ca_wa (p_csn cp_ent))(oa_ent u_hdip)))(u_isn u_hdip))	28	9
4004	(((ca_ent p_isn)(((p_re ca_wa) oa_ent)(u_isn u_hdip)))(u_isn oa_wa))	30	9
4005	(((ca_ent p_isn)(((p_re ca_wa) p_csn)(cp_ent oa_ent)))(oa_wa u_hdip))	30	9
4006	(((ca_ent p_isn)(((p_re ca_wa) p_csn)(cp_ent oa_ent)))(u_hdip u_hdip))	33	9
4007	(((ca_ent p_isn)(((p_re ca_wa) p_csn)(cp_ent oa_ent)))(u_isn oa_wa))	31	9
4008	(((ca_ent p_isn)(((p_re ca_wa) p_csn)(oa_ent oa_wa)))(u_hdip u_hdip))	29	9
4009	(((ca_ent p_isn)(((p_re ca_wa) p_csn)(oa_ent oa_wa)))(u_isn u_hdip))	32	9
4010	(((ca_ent p_isn)(((p_re ca_wa) p_csn)(p_isn oa_ent)))(u_isn u_hdip))	28	9
4011	(((ca_wa p_re)((p_csn cp_ent)((oa_ent u_isn) u_hdip)))(u_isn oa_wa))	22	9
4012	(((ca_wa ca_wa)((p_csn (oa_ent u_isn))(u_hdip u_hdip)))(u_isn u_hdip))	30	9
4013	(((ca_wa p_re)(p_csn ((p_isn oa_ent)(u_isn u_hdip))))(u_isn u_hdip))	22	9
4014	(((ca_wa p_re)((p_csn (oa_ent u_isn)) u_hdip))((u_isn oa_wa) u_hdip))	24	9
4015	((((p_re (ca_wa p_csn))(cp_ent oa_ent))(u_isn u_hdip))(u_isn u_hdip))	31	9
4016	(((cp_ent ca_ent)((ca_wa ca_wa)(p_csn (oa_ent u_isn))))(u_hdip u_hdip))	29	9
4017	(((cp_ent ca_ent)((ca_wa p_re)(p_csn (oa_ent u_isn))))(oa_wa u_hdip))	30	9
4018	(((cp_ent ca_ent)((ca_wa p_re)(p_csn (oa_ent u_isn))))(u_hdip u_hdip))	34	9
4019	(((cp_ent ca_ent)((ca_wa p_re)((ca_wa p_csn)(p_isn cp_ent)))) oa_ent)	21	9
4020	(((cp_ent ca_ent)((p_re ca_wa)(p_csn (oa_ent u_hdip))))(u_isn u_hdip))	32	9
4021	(((cp_ent ca_ent)((p_re ca_wa)((cp_ent oa_ent) oa_wa)))(u_hdip u_hdip))	33	9
4022	(((cp_ent ca_ent)((p_re ca_wa)((p_csn cp_ent) oa_ent)))(u_hdip u_hdip))	31	9
4023	(((cp_ent ca_ent)((p_re ca_wa)((p_csn oa_ent) oa_wa)))(u_isn oa_wa))	26	9
4024	(((cp_ent ca_ent)((p_re ca_wa)((p_csn oa_ent) u_hdip)))(oa_wa u_hdip))	29	9
4025	(((cp_ent ca_ent)((p_re ca_wa)((p_csn oa_ent) u_hdip)))(u_isn u_hdip))	34	9
4026	(((cp_ent ca_ent)((p_re (ca_wa p_csn))(oa_ent u_isn)))(u_hdip u_hdip))	29	9
4027	(((cp_ent ca_ent)(((ca_wa p_re) ca_wa)(cp_ent oa_ent)))(oa_wa oa_wa))	40	9
4028	(((cp_ent ca_ent)(((ca_wa p_re) ca_wa)(cp_ent oa_ent)))(oa_wa u_hdip))	40	9
4029	(((cp_ent ca_ent)(((ca_wa p_re) ca_wa)(oa_ent u_isn)))(u_hdip u_hdip))	35	9
4030	(((cp_ent ca_ent)(((ca_wa p_re) ca_wa)(p_isn cp_ent)))(oa_ent u_isn))	27	9
4031	(((cp_ent ca_ent)(((ca_wa p_re) cp_ent) oa_ent))((u_isn oa_wa) u_hdip))	36	9
4032	(((cp_ent ca_ent)(((ca_wa p_re) cp_ent)(oa_ent oa_wa)))(u_hdip u_hdip))	32	9
4033	(((cp_ent ca_ent)(((ca_wa p_re) cp_ent)(oa_ent u_hdip)))(u_isn oa_wa))	36	9
4034	(((cp_ent ca_ent)(((ca_wa p_re) cp_ent)(oa_ent u_hdip)))(u_isn u_hdip))	39	9
4035	(((cp_ent ca_ent)(((p_re ca_wa) p_csn) oa_ent))((oa_wa u_hdip) oa_wa))	28	9
4036	(((cp_ent ca_ent)(((p_re ca_wa) p_csn)(cp_ent oa_ent)))(oa_wa u_hdip))	29	9
4037	(((cp_ent ca_ent)(((p_re ca_wa) p_csn)(cp_ent oa_ent)))(u_hdip u_hdip))	32	9
4038	(((cp_ent ca_ent)(((p_re ca_wa) p_csn)(oa_ent oa_wa)))(u_isn u_hdip))	23	9
4039	(((cp_ent ca_ent)(((p_re ca_wa) p_csn)(oa_ent u_hdip)))(u_isn u_hdip))	33	9
4040	(((cp_ent ca_wa)((p_re ca_wa)(p_csn (oa_ent oa_wa))))(u_hdip u_hdip))	24	9
4041	(((cp_ent ca_wa)((p_re ca_wa)(p_csn (oa_ent oa_wa))))(u_isn u_hdip))	28	9
4042	(((cp_ent ca_wa)((p_re ca_wa)(p_csn (oa_ent u_isn))))(u_hdip u_hdip))	31	9
4043	(((cp_ent ca_wa)((p_re ca_wa)((cp_ent oa_ent) oa_wa)))(u_isn u_hdip))	31	9
4044	(((cp_ent ca_wa)(((p_re ca_wa) oa_ent)(u_isn u_hdip)))(u_isn oa_wa))	26	9
4045	(((cp_ent ca_wa)(((p_re ca_wa) p_csn) oa_ent))((oa_wa u_hdip) oa_wa))	23	9
4046	(((cp_ent ca_wa)(((p_re ca_wa) p_csn)(oa_ent u_isn)))(u_hdip u_hdip))	32	9
4047	(((cp_ent ca_wa)(((p_re ca_wa) p_csn)(oa_ent oa_wa)))(u_hdip u_hdip))	25	9
4048	(((cp_ent ca_wa)(((p_re ca_wa) p_csn)(oa_ent oa_wa)))(u_isn u_hdip))	27	9
4049	(((cp_ent ca_wa)(((p_re ca_wa) p_csn)(p_isn oa_ent)))(u_hdip u_hdip))	24	9
4050	(((oa_ent (oa_wa u_hdip))((u_isn u_hdip)((u_isn oa_wa) u_csn))) oa_ent)	22	9
4051	(((oa_ent oa_wa)((u_isn u_hdip)((u_isn oa_wa) u_csn)))(u_isn cp_ent))	32	9
4052	(((oa_ent u_hdip)((u_isn u_hdip)((u_isn oa_wa) u_csn)))(cp_ent oa_ent))	37	9
4053	(((oa_wa oa_wa)(((u_csn cp_ent) p_csn) ca_ent))((ca_wa p_re) cp_ent))	22	9
4054	(((oa_wa u_hdip)((u_isn oa_wa)((u_csn cp_ent) p_csn)))(ca_ent p_isn))	17	9
4055	(((oa_wa u_hdip)((u_isn oa_wa)((u_csn cp_ent) p_csn)))(ca_wa ca_wa))	18	9
4056	(((oa_wa u_hdip)((u_isn oa_wa)((u_csn cp_ent) p_csn)))(p_isn ca_wa))	17	9
4057	(((oa_wa u_hdip)(((u_csn cp_ent) p_csn) ca_ent))((ca_wa p_re) cp_ent))	26	9
4058	(((oa_wa u_hdip)(((u_isn oa_wa) u_csn)(cp_ent ca_ent)))(ca_wa ca_wa))	18	9
4059	(((oa_wa u_hdip)((u_isn u_hdip)((oa_wa u_csn) oa_ent)))(oa_wa u_hdip))	17	9
4060	(((p_csn ca_ent)(((ca_wa p_re) ca_wa)(cp_ent oa_ent)))(u_isn u_hdip))	23	9
4061	(((p_csn ca_ent)(((ca_wa p_re) ca_wa)(oa_ent u_isn)))(u_hdip u_hdip))	23	9
4062	(((p_csn ca_ent)(((ca_wa p_re) cp_ent)(oa_ent u_hdip)))(oa_wa u_hdip))	25	9
4063	(((p_csn ca_ent)(((ca_wa p_re) cp_ent)(oa_ent u_hdip)))(u_isn oa_wa))	25	9
4064	(((p_isn ca_ent)((ca_wa p_re)((p_csn oa_ent) oa_wa)))(u_isn oa_wa))	22	9
4065	(((p_isn ca_ent)((ca_wa p_re)((p_csn oa_ent) oa_wa)))(u_isn u_hdip))	24	9
4066	(((p_isn ca_ent)((ca_wa p_re)((p_csn oa_ent) u_hdip)))(oa_wa u_hdip))	23	9

4067	(((p_isn ca_ent)((p_re ca_wa)(p_csn (oa_ent oa_wa))))(u_hdip u_hdip))	20	9
4068	(((p_isn ca_ent)((p_re ca_wa)((cp_ent oa_ent)u_hdip)))(u_isn oa_wa))	32	9
4069	(((p_isn ca_ent)((p_re ca_wa)((cp_ent oa_ent)u_hdip)))(u_isn u_hdip))	35	9
4070	(((p_isn ca_ent)((p_re ca_wa)((p_csn oa_ent)oa_wa)))(u_isn u_hdip))	24	9
4071	(((p_isn ca_ent)(((ca_wa p_re)ca_wa)(cp_ent oa_ent)))(oa_wa u_hdip))	34	9
4072	(((p_isn ca_ent)(((ca_wa p_re)ca_wa)(cp_ent oa_ent)))(u_hdip u_hdip))	38	9
4073	(((p_isn ca_ent)(((ca_wa p_re)ca_wa)(cp_ent oa_ent)))(u_isn u_hdip))	38	9
4074	(((p_isn ca_ent)(((ca_wa p_re)cp_ent)oa_ent))((u_isn oa_wa)u_hdip))	31	9
4075	(((p_isn ca_ent)(((ca_wa p_re)cp_ent)(oa_ent u_isn)))(u_hdip u_hdip))	36	9
4076	(((p_isn ca_ent)(((ca_wa p_re)cp_ent)(oa_ent u_hdip)))(oa_wa oa_wa))	25	9
4077	(((p_isn ca_ent)(((p_re ca_wa)p_csn)(oa_ent u_isn)))(u_hdip u_hdip))	29	9
4078	(((p_isn ca_ent)(((p_re ca_wa)p_csn)(oa_ent oa_wa)))(u_isn u_hdip))	19	9
4079	(((p_isn ca_wa)((p_csn cp_ent)(oa_ent (u_isn u_hdip))))(u_isn u_hdip))	31	9
4080	(((p_isn ca_wa)((p_csn cp_ent)((oa_ent u_isn)u_hdip)))(u_isn u_hdip))	32	9
4081	(((p_isn ca_wa)(((p_isn p_re)(p_csn cp_ent))oa_ent))(u_hdip u_hdip))	31	9
4082	(((p_isn ca_wa)(((p_isn p_re)(p_csn cp_ent))oa_ent))(u_isn u_hdip))	31	9
4083	(((p_isn ca_wa)(((p_re ca_wa)oa_ent)(u_isn u_hdip)))(u_isn oa_wa))	29	9
4084	(((p_isn ca_wa)(((p_re ca_wa)p_csn)(oa_ent oa_wa)))(u_isn u_hdip))	31	9
4085	(((p_isn p_re)((ca_wa (p_csn cp_ent))oa_ent))((u_isn oa_wa)u_hdip))	27	9
4086	(((p_isn p_re)((ca_wa (p_csn cp_ent))(oa_ent oa_wa)))(u_isn oa_wa))	26	9
4087	((((ca_wa p_re)ca_wa)(p_csn ((oa_ent oa_wa)u_hdip)))(u_isn u_hdip))	28	9
4088	((((ca_wa p_re)ca_wa)(p_csn ((p_isn oa_ent)u_hdip)))(u_isn u_hdip))	25	9
4089	(((u_csn (cp_ent ca_ent))(((ca_wa p_re)cp_ent)oa_ent))(u_isn oa_wa))	27	9
4090	(((u_csn cp_ent)(((ca_ent ca_wa)cp_ent)(oa_ent u_hdip)))(u_isn u_hdip))	28	9
4091	(((u_hdip oa_wa)((u_csn cp_ent)((p_isn oa_ent)oa_wa)))(u_hdip u_hdip))	24	9
4092	(((u_hdip oa_wa)((u_csn cp_ent)((p_isn oa_ent)oa_wa)))(u_isn u_hdip))	27	9
4093	(((u_hdip oa_wa)(((u_csn cp_ent)p_csn)ca_ent))((ca_wa p_re)cp_ent))	27	9
4094	(((u_hdip u_hdip)((u_isn oa_wa)((u_csn cp_ent)p_csn)))(ca_ent p_isn))	28	9
4095	(((u_hdip u_hdip)((u_isn oa_wa)((u_csn cp_ent)p_csn)))(ca_ent ca_wa))	28	9
4096	(((u_hdip u_hdip)((u_isn oa_wa)((u_csn cp_ent)p_csn)))(oa_ent u_hdip))	34	9
4097	(((u_hdip u_hdip)(((u_isn oa_wa)u_csn)(cp_ent ca_ent)))(p_isn ca_wa))	22	9
4098	(((u_isn oa_wa)((u_csn cp_ent)((p_isn oa_ent)oa_wa)))(u_hdip u_hdip))	27	9
4099	(((u_isn oa_wa)((u_csn cp_ent)((p_isn oa_ent)oa_wa)))(u_isn u_hdip))	30	9
4100	(((u_isn oa_wa)((u_hdip (u_csn cp_ent))(p_csn ca_ent)))(ca_wa ca_wa))	24	9
4101	(((u_isn oa_wa)((u_hdip (u_csn cp_ent))(p_csn ca_ent)))(ca_wa p_re))	24	9
4102	(((u_isn oa_wa)((u_hdip (u_csn cp_ent))(p_csn ca_ent)))(p_re ca_wa))	24	9
4103	(((u_isn u_hdip)((oa_wa u_csn)((cp_ent oa_ent)oa_wa)))(u_isn u_hdip))	24	9
4104	(((u_isn u_hdip)((u_isn oa_wa)((u_csn cp_ent)p_csn)))(ca_ent p_isn))	26	9
4105	(((u_isn u_hdip)((u_isn oa_wa)((u_csn cp_ent)p_csn)))(ca_ent ca_wa))	27	9
4106	(((u_isn u_hdip)((u_isn oa_wa)((u_csn cp_ent)p_csn)))(ca_wa ca_wa))	17	9
4107	(((u_isn u_hdip)((u_isn u_hdip)(oa_wa (u_csn cp_ent))))(oa_ent u_isn))	34	9
4108	(((u_isn u_hdip)((u_isn u_hdip)(oa_wa (u_csn cp_ent))))(oa_wa u_hdip))	19	9
4109	(((u_isn u_hdip)((u_isn u_hdip)((oa_wa u_csn)oa_ent)))(oa_wa u_hdip))	19	9
4110	(((oa_ent (u_isn u_hdip))((u_isn u_hdip)((u_isn oa_wa)u_csn)))oa_ent)	31	9
4111	(((p_csn (p_isn ca_ent))(((ca_wa p_re)ca_wa)oa_ent))(u_isn u_hdip))	16	9
4112	((((ca_ent ca_wa)cp_ent)((p_csn (oa_ent u_isn))oa_wa))(u_isn u_hdip))	25	9
4113	((((ca_wa p_re)ca_wa)(p_csn (oa_ent (oa_wa u_hdip))))(u_isn u_hdip))	27	9
4114	((((ca_wa p_re)ca_wa)((p_csn (p_isn oa_ent))u_hdip))(u_isn u_hdip))	26	9
4115	((((ca_wa p_re)cp_ent)((p_csn (oa_ent u_isn))oa_wa))(u_isn u_hdip))	22	9
4116	((((u_hdip oa_wa)u_csn)((cp_ent ca_ent)((ca_wa p_re)cp_ent)))oa_ent)	25	9
4117	((((u_isn oa_wa)((u_csn cp_ent)p_csn))(ca_ent p_isn))(p_re ca_wa))	28	9
4118	((((u_isn oa_wa)((u_csn cp_ent)p_csn))ca_ent)((ca_wa p_re)ca_wa))	28	9
4119	((((u_isn oa_wa)((u_csn cp_ent)p_csn))ca_ent)((ca_wa p_re)cp_ent))	28	9
4120	(((p_csn (oa_ent (u_isn u_hdip)))(u_isn oa_wa))((u_csn cp_ent)p_csn))	34	9
4121	(((p_csn (oa_ent (u_isn u_hdip)))(u_isn u_hdip))((u_csn cp_ent)p_csn))	31	9
4122	(((p_csn ((p_isn ca_ent)(ca_wa p_re)))(ca_wa p_csn))(cp_ent oa_ent))	21	9
4123	(((p_csn ((p_isn oa_ent)u_hdip))(u_isn oa_wa))((u_csn cp_ent)p_csn))	22	9
4124	(((p_csn ((p_isn oa_ent)(u_isn u_hdip)))(u_isn u_hdip))(u_isn u_hdip))	32	9
4125	((((ca_ent ca_wa)((p_isn ca_wa)p_csn))(oa_ent oa_wa))(u_hdip u_hdip))	24	9
4126	((((ca_ent ca_wa)((p_isn ca_wa)p_csn))(p_isn oa_ent))(u_hdip u_hdip))	22	9
4127	((((ca_ent ca_wa)((p_isn ca_wa)p_csn))(p_isn oa_ent))(u_isn u_hdip))	23	9
4128	((((ca_ent ca_wa)((p_re ca_wa)oa_ent))(u_isn u_hdip))(u_isn oa_wa))	33	9
4129	((((ca_ent ca_wa)((p_re ca_wa)oa_ent))(u_isn u_hdip))(u_isn u_hdip))	36	9
4130	(((((p_re ca_wa)oa_ent)(oa_wa u_hdip))(u_isn oa_wa))(u_csn cp_ent))	22	9
4131	((((ca_ent ca_wa)((p_re ca_wa)p_csn))oa_ent)((u_isn oa_wa)u_hdip))	32	9
4132	((((ca_ent ca_wa)((p_re ca_wa)p_csn))(cp_ent oa_ent))(oa_wa u_hdip))	30	9
4133	((((ca_ent ca_wa)((p_re ca_wa)p_csn))(cp_ent oa_ent))(u_hdip u_hdip))	33	9
4134	((((ca_ent ca_wa)((p_re ca_wa)p_csn))(cp_ent oa_ent))(u_isn u_hdip))	36	9
4135	((((ca_ent ca_wa)((p_re ca_wa)p_csn))(oa_ent oa_wa))(u_hdip u_hdip))	29	9
4136	((((ca_ent ca_wa)((p_re ca_wa)p_csn))(oa_ent oa_wa))(u_isn u_hdip))	32	9
4137	((((ca_ent ca_wa)((p_re ca_wa)p_csn))(oa_ent u_hdip))(u_isn oa_wa))	32	9
4138	((((ca_ent ca_wa)((p_re ca_wa)p_csn))(oa_ent u_isn))(oa_wa u_hdip))	35	9
4139	((((ca_ent ca_wa)((p_re ca_wa)p_csn))(oa_ent u_isn))(u_hdip u_hdip))	39	9
4140	((((ca_ent ca_wa)((p_re ca_wa)p_csn))(p_isn oa_ent))(u_hdip u_hdip))	27	9
4141	(((((p_re ca_wa)p_csn)(p_isn oa_ent))(u_isn u_hdip))(u_isn u_hdip))	26	9
4142	((((ca_ent p_isn)(ca_wa (p_csn cp_ent)))(oa_ent oa_wa))(u_isn u_hdip))	29	9
4143	((((ca_ent p_isn)((p_re ca_wa)p_csn))(oa_ent oa_wa))(u_isn u_hdip))	33	9
4144	((((cp_ent ca_ent)((ca_wa p_re)cp_ent))oa_ent)((u_isn oa_wa)u_hdip))	37	9
4145	((((cp_ent ca_ent)((ca_wa p_re)cp_ent))(oa_ent oa_wa))(u_hdip u_hdip))	33	9
4146	((((cp_ent ca_ent)((ca_wa p_re)cp_ent))(oa_ent oa_wa))(u_isn oa_wa))	38	9
4147	((((cp_ent ca_ent)((ca_wa p_re)cp_ent))(oa_ent oa_wa))(u_isn u_hdip))	40	9
4148	((((cp_ent ca_ent)((ca_wa p_re)cp_ent))(oa_ent u_hdip))(u_isn oa_wa))	37	9
4149	((((cp_ent ca_ent)((ca_wa p_re)cp_ent))(oa_ent u_hdip))(u_isn u_hdip))	41	9
4150	((((cp_ent ca_ent)((ca_wa p_re)cp_ent))(oa_ent u_isn))(u_hdip u_hdip))	41	9
4151	((((cp_ent ca_ent)((ca_wa p_re)cp_ent))(p_isn oa_ent))(u_hdip u_hdip))	22	9
4152	((((cp_ent ca_ent)((p_re ca_wa)oa_ent))(oa_wa u_hdip))(u_isn oa_wa))	23	9
4153	((((cp_ent ca_ent)((p_re ca_wa)oa_ent))(oa_wa u_hdip))(u_isn u_hdip))	21	9
4154	((((cp_ent ca_ent)((p_re ca_wa)oa_ent))(u_hdip u_hdip))(u_isn u_hdip))	30	9
4155	((((cp_ent ca_ent)((p_re ca_wa)oa_ent))(u_isn u_hdip))(u_isn oa_wa))	28	9
4156	((((cp_ent ca_ent)((p_re ca_wa)oa_ent))(u_isn u_hdip))(u_isn u_hdip))	31	9
4157	((((cp_ent ca_wa)((p_re ca_wa)oa_ent))(oa_wa u_hdip))(u_isn oa_wa))	21	9
4158	((((cp_ent ca_wa)((p_re ca_wa)oa_ent))(oa_wa u_hdip))(u_isn u_hdip))	20	9
4159	((((cp_ent ca_wa)((p_re ca_wa)(p_csn cp_ent)))oa_ent)(u_isn u_hdip))	28	9
4160	((((oa_wa oa_wa)((u_csn cp_ent)p_csn))(p_isn ca_ent))(ca_wa ca_wa))	14	9
4161	((((oa_wa u_csn)((cp_ent ca_wa)cp_ent))oa_ent)((oa_wa u_hdip)oa_wa))	22	9
4162	((((oa_wa u_csn)((cp_ent ca_wa)cp_ent))oa_ent)((u_hdip oa_wa)u_hdip))	21	9
4163	((((oa_wa u_csn)((cp_ent ca_wa)cp_ent))oa_ent)((u_isn oa_wa)u_hdip))	23	9
4164	((((oa_wa u_csn)((cp_ent ca_wa)cp_ent))(oa_ent oa_wa))(u_hdip u_hdip))	21	9
4165	((((oa_wa u_csn)((cp_ent ca_wa)cp_ent))(oa_ent oa_wa))(u_isn oa_wa))	23	9
4166	((((oa_wa u_csn)((cp_ent ca_wa)cp_ent))(oa_ent oa_wa))(u_isn u_hdip))	24	9
4167	((((oa_wa u_csn)((cp_ent ca_wa)cp_ent))(oa_ent u_isn))(oa_wa u_hdip))	22	9
4168	((((oa_wa u_csn)((cp_ent ca_wa)cp_ent))(oa_ent u_isn))(u_hdip oa_wa))	24	9
4169	((((oa_wa u_hdip)(oa_wa (u_csn cp_ent)))(oa_wa u_hdip))(u_isn u_hdip))	21	9
4170	((((oa_wa u_hdip)(oa_wa (u_csn cp_ent)))(p_csn ca_ent))(ca_wa ca_wa))	22	9
4171	((((oa_wa u_hdip)((u_isn u_hdip)(oa_wa u_csn)))oa_ent)(oa_wa u_hdip))	15	9
4172	((((p_csn ca_ent)((p_re ca_wa)p_csn))(cp_ent oa_ent))(u_isn u_hdip))	26	9
4173	((((p_csn oa_ent)((u_isn oa_wa)u_hdip))(u_isn oa_wa))(u_csn cp_ent))	24	9
4174	((((p_isn ca_ent)((p_re ca_wa)oa_ent))(u_isn u_hdip))(u_isn u_hdip))	24	9
4175	((((p_isn ca_ent)((p_re ca_wa)p_csn))(cp_ent oa_ent))(u_hdip u_hdip))	27	9
4176	((((p_isn ca_ent)((p_re ca_wa)p_csn))(cp_ent oa_ent))(u_isn u_hdip))	28	9
4177	((((p_isn ca_ent)((p_re ca_wa)p_csn))(oa_ent oa_wa))(u_hdip u_hdip))	24	9
4178	((((p_isn ca_ent)((p_re ca_wa)p_csn))(oa_ent oa_wa))(u_isn u_hdip))	25	9
4179	((((p_isn ca_ent)((p_re ca_wa)p_csn))(oa_ent u_hdip))(u_isn u_hdip))	30	9
4180	((((p_isn ca_ent)((p_re ca_wa)p_csn))(oa_ent u_isn))(u_hdip u_hdip))	33	9
4181	((((p_isn ca_ent)((p_re ca_wa)p_csn))(p_isn oa_ent))(u_hdip u_hdip))	26	9
4182	((((p_re ca_wa)(p_csn (oa_ent u_isn)))(u_hdip u_hdip))(u_isn u_hdip))	32	9
4183	((((p_re ca_wa)((cp_ent oa_ent)oa_wa))(u_hdip u_hdip))(u_isn u_hdip))	29	9
4184	((((p_re ca_wa)((cp_ent oa_ent)oa_wa))(u_isn u_hdip))(u_isn u_hdip))	34	9
4185	((((p_re ca_wa)((cp_ent oa_ent)u_hdip))(u_isn u_hdip))(u_isn oa_wa))	34	9
4186	((((p_re ca_wa)((cp_ent oa_ent)u_hdip))(u_isn u_hdip))(u_isn u_hdip))	38	9
4187	((((p_re ca_wa)((p_csn oa_ent)u_hdip))(u_isn u_hdip))(u_isn u_hdip))	32	9
4188	((((u_csn cp_ent)((ca_ent ca_wa)p_csn))(oa_ent u_hdip))(u_isn u_hdip))	29	9
4189	((((u_csn cp_ent)((ca_wa p_re)ca_wa))(cp_ent oa_ent))(oa_wa u_hdip))	28	9
4190	((((u_csn cp_ent)((ca_wa p_re)ca_wa))(cp_ent oa_ent))(u_hdip u_hdip))	32	9
4191	((((u_csn cp_ent)((ca_wa p_re)ca_wa))(cp_ent oa_ent))(u_isn oa_wa))	28	9
4192	((((u_hdip oa_wa)(u_csn (cp_ent oa_ent)))(oa_wa u_hdip))(u_isn u_hdip))	27	9
4193	((((u_hdip u_hdip)(oa_wa (u_csn cp_ent)))(ca_ent p_isn))(p_re ca_wa))	29	9
4194	((((u_hdip u_hdip)(oa_wa (u_csn cp_ent)))ca_ent)((ca_wa p_re)ca_wa))	29	9
4195	((((u_hdip u_hdip)(oa_wa (u_csn cp_ent)))ca_ent)((ca_wa p_re)cp_ent))	29	9
4196	((((u_hdip u_hdip)(oa_wa (u_csn cp_ent)))(p_csn ca_ent))(ca_wa ca_wa))	24	9
4197	((((u_hdip u_hdip)(oa_wa (u_csn cp_ent)))(p_csn ca_ent))(ca_wa p_re))	24	9
4198	((((u_hdip u_hdip)(oa_wa (u_csn cp_ent)))(p_csn ca_ent))(p_re ca_wa))	24	9
4199	((((u_hdip u_hdip)((oa_wa u_csn)oa_ent))(oa_wa u_hdip))(u_isn u_hdip))	22	9
4200	((((u_hdip u_hdip)((oa_wa u_csn)oa_ent))(u_isn u_hdip))(u_isn u_hdip))	19	9
4201	((((u_isn oa_wa)((u_csn cp_ent)p_csn))(p_isn ca_ent))(ca_wa ca_wa))	20	9
4202	((((u_isn u_hdip)(oa_wa (u_csn cp_ent)))(oa_wa u_hdip))(u_isn u_hdip))	18	9
4203	((((u_isn u_hdip)((u_isn oa_wa)u_csn))(ca_ent ca_wa))(ca_wa p_csn))	22	9
4204	((((u_isn u_hdip)((u_isn oa_wa)u_csn))(cp_ent ca_ent))(ca_wa ca_wa))	27	9
4205	((((oa_ent (oa_wa u_hdip))((u_isn oa_wa)u_csn))oa_ent)(oa_wa u_hdip))	19	9
4206	((((oa_wa (u_csn cp_ent))p_csn)ca_ent)(((ca_wa p_re)ca_wa)oa_ent))	21	9
4207	((((oa_wa (u_csn cp_ent))p_csn)(ca_ent ca_wa))((p_re ca_wa)oa_ent))	20	9
4208	((((p_csn (ca_ent ca_wa))(p_isn p_re))(ca_wa p_csn))(p_isn p_csn))	47	9
4209	((((p_csn (ca_ent ca_wa))((p_re ca_wa)p_csn))oa_ent)(u_isn u_hdip))	27	9
4210	((((p_csn (ca_wa p_re))(ca_wa p_csn))(oa_ent u_hdip))(u_isn u_hdip))	22	9
4211	((((p_csn (oa_ent u_hdip))(u_isn oa_wa))((u_csn cp_ent)p_csn))ca_ent)	26	9
4212	((((p_csn (oa_ent u_isn))(u_hdip u_hdip))(u_isn oa_wa))(u_csn cp_ent))	32	9
4213	((((p_csn (p_isn ca_wa))(p_isn p_re))(cp_ent oa_ent))(u_hdip u_hdip))	23	9
4214	((((u_csn (ca_ent ca_wa))(p_isn ca_wa))(oa_ent u_isn))(oa_wa oa_wa))	25	9
4215	((((u_csn (ca_ent ca_wa))(p_isn ca_wa))(oa_ent u_isn))(oa_wa u_hdip))	28	9
4216	((((u_csn (ca_ent ca_wa))(p_isn ca_wa))(oa_ent u_isn))(u_hdip oa_wa))	28	9
4217	((((u_csn (ca_ent ca_wa))(p_isn ca_wa))(oa_ent u_isn))(u_hdip u_hdip))	33	9
4218	((((u_csn (ca_ent ca_wa))(p_isn ca_wa))(p_csn oa_ent))(u_hdip u_hdip))	28	9
4219	((((u_csn (u_isn cp_ent))(p_isn ca_wa))(p_isn p_re))(ca_wa p_csn))	16	9
4220	((((u_hdip (u_csn cp_ent))ca_ent)(p_isn ca_wa))((cp_ent oa_ent)u_hdip))	23	9
4221	((((u_hdip (u_isn oa_wa))u_csn)(cp_ent ca_ent))((ca_wa p_re)ca_wa))	31	9
4222	((((u_hdip (u_isn oa_wa))u_csn)(cp_ent ca_ent))((ca_wa p_re)cp_ent))	30	9
4223	((((u_hdip (u_isn oa_wa))u_csn)(ca_ent ca_wa))((p_re ca_wa)oa_ent))	20	9
4224	((((u_hdip (u_isn oa_wa))u_csn)(ca_ent ca_wa))((p_re ca_wa)p_csn))	26	9
4225	((((u_hdip (u_isn oa_wa))u_csn)(cp_ent ca_wa))((p_re ca_wa)p_csn))	22	9
4226	((((u_hdip (u_isn oa_wa))u_csn)(oa_ent (oa_wa u_hdip)))(u_isn u_hdip))	25	9
4227	((((u_hdip (u_isn oa_wa))u_csn)((cp_ent ca_wa)cp_ent))(oa_ent u_hdip))	20	9
4228	((((u_hdip (u_isn oa_wa))u_csn)((cp_ent ca_wa)cp_ent))(oa_ent u_isn))	20	9
4229	((((u_hdip (u_isn u_csn))(u_isn cp_ent))ca_ent)((ca_wa p_re)cp_ent))	24	9
4230	((((u_hdip (u_isn u_csn))(u_isn cp_ent))(ca_ent ca_wa))(ca_wa p_csn))	22	9
4231	(((((ca_ent ca_wa)cp_ent)oa_ent)(u_hdip (u_isn oa_wa)))(u_isn oa_wa))	32	9
4232	(((((ca_ent ca_wa)cp_ent)oa_ent)(u_hdip (u_isn oa_wa)))(u_isn u_hdip))	33	9
4233	(((((ca_ent ca_wa)cp_ent)(oa_ent oa_wa))(u_isn u_hdip))(u_isn u_hdip))	34	9
4234	(((((ca_ent ca_wa)cp_ent)(oa_ent u_hdip))(u_isn u_hdip))(u_isn oa_wa))	35	9
4235	(((((ca_ent ca_wa)cp_ent)(oa_ent u_isn))(u_hdip u_hdip))(u_isn oa_wa))	34	9
4236	(((((ca_ent ca_wa)cp_ent)(oa_ent u_isn))(u_hdip u_hdip))(u_isn u_hdip))	34	9
4237	(((((ca_ent ca_wa)p_csn)oa_ent)(u_isn u_hdip))((u_isn oa_wa)u_hdip))	30	9
4238	(((((ca_ent ca_wa)p_csn)(cp_ent oa_ent))(u_isn oa_wa))(u_isn oa_wa))	25	9
4239	(((((ca_ent ca_wa)p_csn)(cp_ent oa_ent))(u_isn oa_wa))(u_isn u_hdip))	25	9
4240	(((((ca_ent ca_wa)p_csn)(cp_ent oa_ent))(u_isn u_hdip))(u_isn oa_wa))	27	9
4241	(((((ca_ent ca_wa)p_csn)(oa_ent u_isn))(u_hdip u_hdip))(u_isn u_hdip))	30	9
4242	(((((ca_ent ca_wa)p_re)(cp_ent oa_ent))(oa_wa u_hdip))(u_isn u_hdip))	31	9
4243	(((((ca_ent ca_wa)p_re)(cp_ent oa_ent))(u_hdip u_hdip))(u_isn oa_wa))	29	9
4244	(((((ca_ent ca_wa)p_re)(cp_ent oa_ent))(u_hdip u_hdip))(u_isn u_hdip))	34	9
4245	(((((ca_ent ca_wa)p_re)(cp_ent oa_ent))(u_isn u_hdip))(u_isn u_hdip))	28	9
4246	(((((ca_ent ca_wa)p_re)(p_csn oa_ent))(oa_wa u_hdip))(u_isn u_hdip))	23	9
4247	(((((ca_ent ca_wa)p_re)(p_csn oa_ent))(u_hdip u_hdip))(u_isn u_hdip))	29	9
4248	(((((ca_ent ca_wa)p_re)(p_csn oa_ent))(u_isn u_hdip))(u_isn u_hdip))	32	9
4249	(((((ca_ent ca_wa)p_re)(p_isn p_csn))(p_isn cp_ent))(oa_ent u_isn))	22	9
4250	(((((ca_ent p_re)p_csn)(oa_ent u_isn))(u_hdip u_hdip))(u_isn u_hdip))	31	9
4251	(((((ca_ent p_re)(cp_ent oa_ent))oa_wa)(u_hdip u_hdip))(u_isn oa_wa))	20	9
4252	(((((ca_ent p_re)(cp_ent oa_ent))oa_wa)(u_hdip u_hdip))(u_isn u_hdip))	21	9
4253	(((((ca_wa p_re)ca_wa)(cp_ent oa_ent))(oa_wa u_hdip))(u_isn u_hdip))	35	9
4254	(((((ca_wa p_re)ca_wa)(cp_ent oa_ent))(u_hdip u_hdip))(u_isn oa_wa))	34	9
4255	(((((ca_wa p_re)ca_wa)(cp_ent oa_ent))(u_hdip u_hdip))(u_isn u_hdip))	39	9
4256	(((((ca_wa p_re)ca_wa)(cp_ent oa_ent))(u_isn oa_wa))(u_isn oa_wa))	34	9
4257	(((((ca_wa p_re)ca_wa)(cp_ent oa_ent))(u_isn oa_wa))(u_isn u_hdip))	35	9
4258	(((((ca_wa p_re)ca_wa)(cp_ent oa_ent))(u_isn u_hdip))(oa_wa u_hdip))	36	9
4259	(((((ca_wa p_re)ca_wa)(cp_ent oa_ent))(u_isn u_hdip))(u_isn oa_wa))	39	9
4260	(((((ca_wa p_re)ca_wa)(cp_ent oa_ent))(u_isn u_hdip))(u_isn u_hdip))	41	9
4261	(((((ca_wa p_re)ca_wa)(oa_ent u_isn))(u_hdip u_hdip))(u_isn oa_wa))	31	9
4262	(((((ca_wa p_re)ca_wa)(oa_ent u_isn))(u_hdip u_hdip))(u_isn u_hdip))	33	9
4263	(((((ca_wa p_re)cp_ent)(oa_ent u_hdip))(u_isn u_hdip))(u_isn oa_wa))	33	9
4264	(((((ca_wa p_re)cp_ent)(oa_ent oa_wa))(u_hdip u_hdip))(u_isn u_hdip))	30	9
4265	(((((ca_wa p_re)cp_ent)(oa_ent oa_wa))(u_isn u_hdip))(u_isn u_hdip))	32	9
4266	(((((ca_wa p_re)cp_ent)(oa_ent u_hdip))(u_isn oa_wa))(u_isn oa_wa))	31	9
4267	(((((ca_wa p_re)cp_ent)(oa_ent u_hdip))(u_isn oa_wa))(u_isn u_hdip))	34	9
4268	(((((ca_wa p_re)cp_ent)(oa_ent u_hdip))(u_isn u_hdip))(u_isn u_hdip))	38	9
4269	(((((ca_wa p_re)cp_ent)(oa_ent u_isn))(u_hdip u_hdip))(u_isn u_hdip))	34	9
4270	(((((cp_ent ca_ent)cp_ent)oa_ent)(u_hdip (u_isn oa_wa)))(u_isn oa_wa))	29	9
4271	(((((cp_ent ca_ent)cp_ent)oa_ent)(u_hdip (u_isn oa_wa)))(u_isn u_hdip))	28	9
4272	(((((cp_ent ca_ent)cp_ent)(oa_ent oa_wa))(u_isn u_hdip))(u_isn u_hdip))	32	9
4273	(((((cp_ent ca_ent)cp_ent)(oa_ent u_hdip))(u_isn oa_wa))(u_isn oa_wa))	32	9
4274	(((((cp_ent ca_ent)cp_ent)(oa_ent u_hdip))(u_isn oa_wa))(u_isn u_hdip))	31	9
4275	(((((cp_ent ca_ent)cp_ent)(oa_ent u_hdip))(u_isn u_hdip))(u_isn oa_wa))	32	9
4276	(((((cp_ent ca_ent)cp_ent)(oa_ent u_hdip))(u_isn u_hdip))(u_isn u_hdip))	35	9
4277	(((((cp_ent ca_ent)cp_ent)(oa_ent u_isn))(u_hdip oa_wa))(u_isn oa_wa))	27	9
4278	(((((cp_ent ca_ent)cp_ent)(oa_ent u_isn))(u_hdip oa_wa))(u_isn u_hdip))	26	9
4279	(((((cp_ent ca_ent)cp_ent)(oa_ent u_isn))(u_hdip u_hdip))(u_isn u_hdip))	32	9
4280	(((((cp_ent ca_wa)cp_ent)(oa_ent oa_wa))(u_hdip u_hdip))(u_isn u_hdip))	23	9
4281	(((((cp_ent ca_wa)cp_ent)(oa_ent oa_wa))(u_isn u_hdip))(u_isn u_hdip))	31	9
4282	(((((cp_ent ca_wa)cp_ent)(oa_ent u_hdip))(u_isn oa_wa))(u_isn oa_wa))	31	9
4283	(((((cp_ent ca_wa)cp_ent)(oa_ent u_hdip))(u_isn oa_wa))(u_isn u_hdip))	31	9
4284	(((((cp_ent ca_wa)cp_ent)(oa_ent u_hdip))(u_isn u_hdip))(u_isn u_hdip))	34	9
4285	(((((cp_ent ca_wa)cp_ent)(oa_ent u_isn))(u_hdip u_hdip))(u_isn u_hdip))	29	9
4286	((((((cp_ent oa_ent)oa_wa)u_csn)(u_isn cp_ent))ca_ent)(p_isn p_re))	24	9
4287	(((((cp_ent oa_ent)oa_wa)u_csn)(u_isn cp_ent))((ca_ent ca_wa)p_re))	24	9
4288	(((((cp_ent oa_ent)oa_wa)u_csn)(u_isn cp_ent))((ca_wa p_re)ca_wa))	20	9
4289	(((((oa_ent oa_wa)u_csn)(u_isn cp_ent))(ca_ent p_isn))(p_re ca_wa))	21	9
4290	(((((oa_ent oa_wa)u_csn)(u_isn cp_ent))ca_ent)((ca_wa p_re)ca_wa))	21	9
4291	(((((oa_ent oa_wa)u_csn)(u_isn cp_ent))(ca_ent ca_wa))(ca_wa p_csn))	20	9
4292	(((((oa_ent oa_wa)u_csn)(u_isn cp_ent))(ca_ent ca_wa))(p_isn p_re))	22	9
4293	(((((oa_ent oa_wa)u_csn)(u_isn cp_ent))(ca_ent ca_wa))(p_re ca_wa))	22	9
4294	(((((oa_ent oa_wa)u_csn)(u_isn cp_ent))(ca_ent ca_wa))(p_re p_csn))	20	9
4295	(((((p_isn ca_wa)p_csn)(oa_ent oa_wa))(u_isn u_hdip))(u_isn u_hdip))	24	9
4296	(((((p_isn ca_wa)p_csn)(p_isn oa_ent))(u_isn u_hdip))(u_isn u_hdip))	21	9
4297	(((((p_isn oa_ent)oa_wa)u_csn)((cp_ent oa_ent)u_hdip))(u_isn oa_wa))	17	9
4298	(((((p_isn oa_ent)oa_wa)u_csn)((cp_ent oa_ent)u_hdip))(u_isn u_hdip))	17	9
4299	(((((p_isn oa_ent)oa_wa)u_csn)(oa_ent (oa_wa u_hdip)))(u_isn u_hdip))	16	9
4300	(((((p_isn oa_ent)oa_wa)u_csn)(oa_ent (u_isn u_hdip)))(u_isn oa_wa))	17	9
4301	(((((p_isn oa_ent)oa_wa)u_csn)(oa_ent (u_isn u_hdip)))(u_isn u_hdip))	17	9
4302	(((((p_isn oa_ent)oa_wa)u_csn)((cp_ent oa_ent)oa_wa))(u_isn u_hdip))	17	9
4303	(((((p_re ca_wa)oa_ent)(oa_wa u_hdip))(u_isn u_hdip))(oa_wa u_csn))	26	9
4304	(((((p_re ca_wa)oa_ent)(oa_wa u_hdip))(u_isn u_hdip))(u_isn u_hdip))	24	9
4305	(((((p_re ca_wa)oa_ent)(u_hdip u_hdip))(u_isn u_hdip))(oa_wa u_csn))	28	9
4306	(((((p_re ca_wa)p_csn)oa_ent)(u_isn u_hdip))((u_isn oa_wa)u_hdip))	32	9
4307	(((((p_re ca_wa)p_csn)(cp_ent oa_ent))(u_isn u_hdip))(u_isn u_hdip))	32	9
4308	(((((p_re ca_wa)p_csn)(oa_ent u_isn))(u_hdip u_hdip))(u_isn u_hdip))	33	9
4309	(((((u_hdip oa_wa)u_csn)(cp_ent ca_wa))(p_isn p_re))(ca_wa p_csn))	19	9
4310	(((((u_hdip oa_wa)u_csn)((cp_ent ca_wa)cp_ent))oa_ent)(oa_wa u_hdip))	21	9
4311	(((((u_isn cp_ent)ca_ent)((ca_wa p_re)ca_wa))(p_csn cp_ent))oa_ent)	18	9
4312	(((((u_isn oa_wa)u_hdip)(oa_wa u_csn))oa_ent)((oa_wa u_hdip)oa_wa))	21	9
4313	(((((u_isn oa_wa)u_hdip)(oa_wa u_csn))(cp_ent ca_ent))(ca_wa ca_wa))	25	9
4314	(((((u_isn u_hdip)(oa_wa u_csn))oa_ent)(oa_wa u_hdip))(u_isn u_hdip))	22	9
4315	(p_csn ((p_isn ca_ent)((((ca_wa p_re)ca_wa)oa_ent)(u_hdip u_hdip))))	21	9
4316	(p_csn ((p_isn ca_ent)((((ca_wa p_re)ca_wa)oa_ent)(u_isn u_hdip))))	21	9
4317	(p_csn ((p_isn ca_ent)((((ca_wa p_re)cp_ent)oa_ent)(u_isn u_hdip))))	27	9
4318	(p_csn (((p_isn ca_ent)(((ca_wa p_re)cp_ent)oa_ent))(u_hdip u_hdip)))	25	9
4319	(p_csn (((((ca_ent ca_wa)cp_ent)oa_ent)(u_isn u_hdip))(u_isn u_hdip)))	28	9
4320	(((u_hdip u_hdip)(((oa_wa (u_csn cp_ent))p_csn)ca_ent))(ca_wa ca_wa))	27	9
4321	((((((cp_ent oa_ent)oa_wa)u_csn)(u_isn cp_ent))ca_ent)(ca_wa ca_wa))	25	9
4322	(((p_isn ca_ent)(ca_wa p_re))((ca_wa p_csn)((p_isn oa_ent)(u_isn u_hdip))))	21	10
4323	((((u_isn cp_ent)(ca_ent ca_wa))(p_re ca_wa))((p_csn cp_ent)(oa_ent u_hdip)))	17	10
4324	((ca_ent ca_wa)((p_isn p_re)((ca_wa (p_csn cp_ent))(oa_ent (oa_wa u_hdip)))))	27	10
4325	((ca_ent ca_wa)((p_isn p_re)((ca_wa (p_csn cp_ent))((oa_ent u_isn)u_hdip))))	32	10
4326	((ca_ent ca_wa)(((p_re ca_wa)(cp_ent oa_ent))((oa_wa u_hdip)(u_isn oa_wa))))	33	10
4327	((ca_ent ca_wa)(((p_re ca_wa)(p_csn oa_ent))((oa_wa u_hdip)(u_isn oa_wa))))	20	10
4328	((ca_ent p_isn)(((p_re ca_wa)(cp_ent oa_ent))((oa_wa u_hdip)(u_isn oa_wa))))	28	10
4329	(((ca_ent ca_wa)p_re)((ca_wa (p_csn cp_ent))((p_csn oa_ent)(u_isn oa_wa))))	17	10
4330	((cp_ent ca_ent)(((ca_wa ca_wa)(cp_ent oa_ent))((oa_wa u_hdip)(u_isn oa_wa))))	20	10
4331	((cp_ent ca_ent)(((ca_wa ca_wa)(p_csn oa_ent))((oa_wa u_hdip)(u_isn oa_wa))))	20	10
4332	(((cp_ent ca_ent)((p_isn p_re)((ca_wa p_csn)(cp_ent oa_ent))))(u_isn u_hdip))	32	10
4333	(((cp_ent ca_ent)(ca_wa p_re))(((ca_wa (p_csn cp_ent))oa_ent)(u_isn u_hdip)))	29	10
4334	((cp_ent ca_ent)((p_isn p_re)((ca_wa (p_csn cp_ent))((oa_ent u_isn)u_hdip))))	30	10
4335	((cp_ent ca_ent)((p_isn p_re)((ca_wa (p_csn cp_ent))(oa_ent (oa_wa u_hdip)))))	25	10
4336	((cp_ent ca_ent)(((p_re ca_wa)(cp_ent oa_ent))((oa_wa u_hdip)(u_isn oa_wa))))	29	10
4337	((cp_ent ca_wa)(((p_re ca_wa)(cp_ent oa_ent))((oa_wa u_hdip)(u_isn oa_wa))))	29	10
4338	(((oa_wa u_csn)((u_isn cp_ent)(ca_ent ca_wa)))((p_isn p_re)(ca_wa p_csn)))	23	10
4339	(((p_isn ca_ent)((p_isn p_re)((ca_wa p_csn)(cp_ent oa_ent))))(u_isn u_hdip))	26	10
4340	((p_isn ca_ent)(((p_re ca_wa)(cp_ent oa_ent))((oa_wa u_hdip)(u_isn oa_wa))))	26	10
4341	((p_isn ca_ent)((p_isn p_re)((ca_wa (p_csn cp_ent))((oa_ent u_isn)u_hdip))))	26	10
4342	(((p_isn ca_ent)(ca_wa p_re))(((ca_wa (p_csn cp_ent))oa_ent)(u_isn u_hdip)))	25	10
4343	((p_isn ca_wa)(((p_re ca_wa)(cp_ent oa_ent))((oa_wa u_hdip)(u_isn oa_wa))))	30	10
4344	(((u_csn cp_ent)(ca_ent ca_wa))((p_re ca_wa)((cp_ent oa_ent)(oa_wa u_hdip))))	26	10
4345	((u_csn (cp_ent ca_ent))(((ca_wa p_re)ca_wa)((p_csn (oa_ent u_isn))u_hdip)))	25	10
4346	((u_csn (cp_ent ca_ent))(((ca_wa p_re)ca_wa)((p_csn oa_ent)(u_isn u_hdip))))	26	10
4347	((u_csn (cp_ent ca_ent))(((ca_wa p_re)ca_wa)((cp_ent oa_ent)(oa_wa u_hdip))))	28	10
4348	((u_csn (cp_ent ca_ent))(((ca_wa p_re)cp_ent)((p_isn oa_ent)(u_isn u_hdip))))	22	10
4349	(((u_csn cp_ent)(ca_ent ca_wa))(((p_re ca_wa)p_csn)(oa_ent (u_isn u_hdip))))	25	10
4350	(((u_csn cp_ent)(ca_ent ca_wa))(((p_re ca_wa)p_csn)((p_isn oa_ent)u_hdip)))	20	10
4351	(((u_csn cp_ent)(ca_ent ca_wa))(((p_re ca_wa)(cp_ent oa_ent))(oa_wa u_hdip)))	25	10
4352	(((u_csn cp_ent)(ca_ent ca_wa))(((p_re ca_wa)(cp_ent oa_ent))(u_hdip u_hdip)))	30	10
4353	(((u_csn cp_ent)(ca_ent ca_wa))(((p_re ca_wa)(cp_ent oa_ent))(u_isn u_hdip)))	31	10
4354	((u_csn (cp_ent ca_ent))(((ca_wa p_re)ca_wa)((p_csn oa_ent)(u_hdip u_hdip))))	26	10
4355	(((u_csn cp_ent)(ca_ent ca_wa))(((p_re ca_wa)(p_csn oa_ent))(u_isn u_hdip)))	27	10
4356	((u_hdip oa_wa)((u_csn (cp_ent ca_ent))(((ca_wa p_re)ca_wa)(cp_ent oa_ent))))	26	10
4357	((u_hdip oa_wa)(((u_csn cp_ent)(p_csn (ca_ent ca_wa)))((p_re ca_wa)oa_ent)))	17	10
4358	((u_isn oa_wa)(((u_csn cp_ent)(p_csn (ca_ent ca_wa)))((p_re ca_wa)oa_ent)))	18	10
4359	((oa_wa (u_csn (cp_ent ca_ent)))(((ca_wa p_re)(p_csn oa_ent))(u_isn u_hdip)))	21	10
4360	((oa_wa (u_csn (cp_ent ca_ent)))(((ca_wa p_re)ca_wa)(p_csn (oa_ent u_isn))))	24	10
4361	((oa_wa (u_csn (cp_ent ca_ent)))(((ca_wa p_re)ca_wa)((p_csn oa_ent)u_hdip)))	23	10
4362	((oa_wa (u_csn (cp_ent ca_ent)))(((ca_wa p_re)cp_ent)(oa_ent (oa_wa u_hdip))))	24	10
4363	((oa_wa (u_csn (cp_ent ca_ent)))(((ca_wa p_re)cp_ent)(oa_ent (u_isn u_hdip))))	29	10
4364	((oa_wa (u_csn (cp_ent ca_ent)))(((ca_wa p_re)cp_ent)((oa_ent u_isn)u_hdip)))	30	10
4365	((oa_wa (u_csn (cp_ent ca_ent)))(((ca_wa p_re)(p_csn oa_ent))(u_hdip u_hdip)))	21	10
4366	((oa_wa (u_csn (cp_ent ca_ent)))(((ca_wa p_re)(p_csn oa_ent))(u_isn u_hdip)))	21	10
4367	((p_csn (p_isn ca_ent))((((ca_wa p_re)ca_wa)(cp_ent oa_ent))(u_isn u_hdip)))	18	10
4368	(((u_csn cp_ent)(p_csn (ca_ent ca_wa)))(((p_re ca_wa)(cp_ent oa_ent))u_hdip))	25	10
4369	((((u_csn cp_ent)p_csn)ca_ent)(((ca_wa p_re)cp_ent)((oa_ent u_isn)u_hdip)))	27	10
4370	((((u_csn cp_ent)p_csn)ca_ent)(((ca_wa p_re)cp_ent)((p_isn oa_ent)u_hdip)))	17	10
4371	(((u_csn cp_ent)(p_isn ca_ent))(((p_re ca_wa)(cp_ent oa_ent))(oa_wa u_hdip)))	21	10
4372	((u_csn (p_isn ca_ent))((((ca_wa p_re)ca_wa)(p_csn oa_ent))(u_isn u_hdip)))	21	10
4373	(((u_csn cp_ent)(p_isn ca_ent))(((ca_wa p_re)cp_ent)((p_isn oa_ent)u_hdip)))	20	10
4374	((u_csn (p_isn ca_ent))(((ca_wa p_re)((p_csn oa_ent)u_hdip))(u_isn u_hdip)))	20	10
4375	((u_csn (p_isn ca_ent))((((ca_wa p_re)ca_wa)(p_csn oa_ent))(u_isn u_hdip)))	22	10
4376	((u_hdip (u_isn oa_wa))(((u_csn cp_ent)(p_csn (ca_ent ca_wa)))(p_re ca_wa)))	25	10
4377	(((ca_wa p_re)ca_wa)(((p_csn oa_ent)(u_isn u_hdip))((u_isn oa_wa)u_hdip)))	30	10
4378	(((ca_wa p_re)cp_ent)(((p_csn oa_ent)(u_isn u_hdip))((u_isn oa_wa)u_hdip)))	22	10
4379	((cp_ent ca_ent)((p_isn p_re)(((ca_wa (p_csn cp_ent))oa_ent)(u_hdip u_hdip))))	29	10
4380	(((cp_ent oa_ent)(oa_wa u_hdip))(((u_isn u_hdip)(oa_wa u_csn))(u_isn cp_ent)))	25	10
4381	(((cp_ent oa_ent)(oa_wa u_hdip))((u_isn u_hdip)((oa_wa u_csn)(u_isn cp_ent))))	29	10
4382	(((cp_ent oa_ent)(u_isn u_hdip))((u_isn u_hdip)((u_isn u_hdip)(oa_wa u_csn))))	31	10
4383	(((cp_ent oa_ent)(u_isn u_hdip))(((u_isn u_hdip)(oa_wa u_csn))(u_isn cp_ent)))	28	10
4384	(((oa_wa (u_csn cp_ent))ca_ent)((ca_wa p_re)((p_csn oa_ent)(u_isn u_hdip))))	22	10
4385	(((oa_wa (u_csn cp_ent))(ca_ent ca_wa))((p_re ca_wa)(p_csn (oa_ent u_hdip))))	22	10
4386	(((oa_wa (u_csn cp_ent))(ca_ent ca_wa))((p_re ca_wa)(p_csn (oa_ent u_isn))))	25	10
4387	(((oa_wa (u_csn cp_ent))(ca_ent ca_wa))((p_re ca_wa)((cp_ent oa_ent)oa_wa)))	24	10
4388	(((oa_wa (u_csn cp_ent))(ca_ent ca_wa))((p_re ca_wa)((cp_ent oa_ent)u_hdip)))	30	10
4389	(((oa_wa (u_csn cp_ent))(ca_ent ca_wa))((p_re ca_wa)((p_csn oa_ent)u_hdip)))	24	10
4390	(((oa_wa (u_csn cp_ent))ca_ent)(((ca_wa p_re)cp_ent)(oa_ent (u_isn u_hdip))))	30	10
4391	(((oa_wa (u_csn cp_ent))ca_ent)(((ca_wa p_re)cp_ent)((oa_ent u_isn)u_hdip)))	31	10
4392	(((oa_wa (u_csn cp_ent))ca_ent)(((ca_wa p_re)(p_csn oa_ent))(u_hdip u_hdip)))	22	10
4393	(((oa_wa (u_csn cp_ent))ca_ent)(((ca_wa p_re)(p_csn oa_ent))(u_isn u_hdip)))	22	10
4394	(((oa_wa (u_csn cp_ent))(ca_ent ca_wa))(((p_re ca_wa)oa_ent)(u_hdip u_hdip)))	22	10
4395	(((oa_wa (u_csn cp_ent))(ca_ent ca_wa))(((p_re ca_wa)oa_ent)(u_isn u_hdip)))	22	10
4396	(((oa_wa (u_csn cp_ent))(ca_ent ca_wa))(((p_re ca_wa)p_csn)(oa_ent u_isn)))	26	10
4397	(((oa_wa (u_csn cp_ent))(ca_ent ca_wa))(((p_re ca_wa)p_csn)(oa_ent u_hdip)))	24	10
4398	(((oa_wa (u_csn cp_ent))(ca_ent ca_wa))(((p_re ca_wa)p_csn)(p_isn oa_ent)))	20	10
4399	(((oa_wa u_csn)(cp_ent ca_wa))((p_re ca_wa)((cp_ent oa_ent)(oa_wa u_hdip))))	24	10
4400	(((oa_wa u_csn)(cp_ent ca_wa))(((p_re ca_wa)(cp_ent oa_ent))(oa_wa u_hdip)))	23	10
4401	(((oa_wa u_csn)(cp_ent ca_wa))(((p_re ca_wa)(cp_ent oa_ent))(u_hdip u_hdip)))	24	10
4402	(((oa_wa u_csn)(cp_ent ca_wa))(((p_re ca_wa)(cp_ent oa_ent))(u_isn u_hdip)))	24	10
4403	(((oa_wa (u_csn cp_ent))(p_csn ca_ent))(((ca_wa p_re)ca_wa)(cp_ent oa_ent)))	25	10
4404	(((oa_wa (u_csn cp_ent))(p_csn ca_ent))(((ca_wa p_re)ca_wa)(oa_ent u_isn)))	20	10
4405	(((oa_wa (u_csn cp_ent))(p_csn ca_ent))(((ca_wa p_re)cp_ent)(oa_ent u_isn)))	25	10
4406	(((oa_wa (u_csn cp_ent))(p_csn ca_ent))(((ca_wa p_re)cp_ent)(oa_ent u_hdip)))	25	10
4407	(((oa_wa u_csn)(p_isn ca_ent))((ca_wa p_re)((p_csn oa_ent)(u_isn u_hdip))))	17	10
4408	(((oa_wa u_csn)(p_isn ca_ent))(((ca_wa p_re)cp_ent)(oa_ent (oa_wa u_hdip))))	19	10
4409	(((oa_wa u_csn)(p_isn ca_ent))(((ca_wa p_re)cp_ent)(oa_ent (u_isn u_hdip))))	23	10
4410	(((oa_wa u_csn)(p_isn ca_ent))(((ca_wa p_re)cp_ent)((oa_ent u_isn)u_hdip)))	24	10
4411	((((oa_wa u_hdip)oa_wa)(u_csn (cp_ent ca_wa)))((p_re ca_wa)(cp_ent oa_ent)))	21	10
4412	(((oa_wa u_hdip)oa_wa)(((u_csn cp_ent)(p_csn (ca_ent ca_wa)))(p_re ca_wa)))	23	10
4413	(((oa_wa u_hdip)(u_isn oa_wa))((u_csn (cp_ent ca_ent))((ca_wa p_re)ca_wa)))	25	10
4414	(((oa_wa u_hdip)(u_isn oa_wa))(((u_csn cp_ent)(ca_ent p_isn))(p_re ca_wa)))	25	10
4415	(((oa_wa u_hdip)(u_isn oa_wa))(((u_csn cp_ent)(p_csn ca_ent))(ca_wa ca_wa)))	22	10
4416	(((oa_wa u_hdip)(u_isn oa_wa))(((u_csn cp_ent)(p_csn ca_ent))(ca_wa p_re)))	22	10
4417	(((oa_wa u_hdip)(u_isn oa_wa))(((u_csn cp_ent)(p_csn ca_ent))(p_re ca_wa)))	22	10
4418	(((oa_wa u_hdip)(u_isn oa_wa))(((u_csn cp_ent)(ca_ent ca_wa))(p_re ca_wa)))	26	10
4419	(((p_isn ca_ent)(ca_wa p_re))((ca_wa (p_csn cp_ent))(oa_ent (oa_wa u_hdip))))	19	10
4420	(((u_csn cp_ent)(ca_wa p_re))(((p_csn oa_ent)(u_isn u_hdip))(u_isn u_hdip)))	26	10
4421	(((u_csn cp_ent)(oa_ent oa_wa))((u_isn u_hdip)((u_isn u_hdip)(oa_wa u_csn))))	26	10
4422	(((u_csn cp_ent)(p_isn ca_ent))((ca_wa p_re)((p_csn oa_ent)(u_isn u_hdip))))	20	10
4423	(((u_csn cp_ent)(p_isn ca_ent))((((ca_wa p_re)ca_wa)(cp_ent oa_ent))u_hdip))	28	10
4424	(((u_csn cp_ent)(p_isn ca_ent))(((ca_wa p_re)cp_ent)(oa_ent (oa_wa u_hdip))))	23	10
4425	(((u_csn cp_ent)(p_isn ca_ent))(((ca_wa p_re)cp_ent)(oa_ent (u_isn u_hdip))))	27	10
4426	(((u_csn cp_ent)(p_isn ca_ent))(((ca_wa p_re)cp_ent)((oa_ent u_isn)u_hdip)))	28	10
4427	(((u_csn cp_ent)(p_isn ca_ent))((ca_wa p_re)((p_csn oa_ent)(u_isn u_hdip))))	21	10
4428	(((u_csn cp_ent)(p_isn ca_ent))(((p_re ca_wa)(cp_ent oa_ent))(u_isn u_hdip)))	26	10
4429	(((u_csn cp_ent)(p_isn ca_wa))((p_re ca_wa)((cp_ent oa_ent)(oa_wa u_hdip))))	23	10
4430	(((u_csn cp_ent)(p_isn ca_wa))(((p_re ca_wa)(cp_ent oa_ent))(oa_wa u_hdip)))	22	10
4431	(((u_csn cp_ent)(p_isn ca_wa))(((p_re ca_wa)(cp_ent oa_ent))(u_hdip u_hdip)))	23	10
4432	(((u_csn cp_ent)(p_isn ca_wa))(((p_re ca_wa)(cp_ent oa_ent))(u_isn u_hdip)))	23	10
4433	(((u_hdip (u_csn cp_ent))(ca_ent ca_wa))((p_re ca_wa)((cp_ent oa_ent)oa_wa)))	21	10
4434	((((u_hdip (u_csn cp_ent))ca_ent)((ca_wa p_re)ca_wa))((cp_ent oa_ent)u_hdip))	27	10
4435	(((u_hdip (u_csn cp_ent))(ca_ent ca_wa))(((p_re ca_wa)oa_ent)(u_hdip u_hdip)))	20	10
4436	(((u_hdip u_csn)(cp_ent ca_ent))(((ca_wa p_re)cp_ent)((oa_ent u_isn)u_hdip)))	25	10
4437	(((u_hdip u_csn)(cp_ent ca_wa))(((p_re ca_wa)(cp_ent oa_ent))(u_hdip u_hdip)))	21	10
4438	(((u_isn cp_ent)(p_isn ca_ent))(((ca_wa p_re)cp_ent)(oa_ent (u_isn u_hdip))))	20	10
4439	(((u_isn cp_ent)(p_isn ca_ent))(((ca_wa p_re)cp_ent)((oa_ent u_isn)u_hdip)))	21	10
4440	(((u_isn oa_wa)u_hdip)(((u_csn cp_ent)(p_csn (ca_ent ca_wa)))(p_re ca_wa)))	26	10
4441	(((u_isn oa_wa)u_hdip)(((u_csn cp_ent)p_csn)((p_isn ca_ent)(ca_wa p_re))))	20	10
4442	(((u_isn oa_wa)u_hdip)(((u_isn oa_wa)((u_csn cp_ent)p_csn))(ca_ent ca_wa)))	17	10
4443	(((u_isn u_csn)(cp_ent ca_ent))(((ca_wa p_re)cp_ent)(oa_ent (oa_wa u_hdip))))	19	10
4444	(((u_isn u_csn)(cp_ent ca_ent))(((ca_wa p_re)cp_ent)((oa_ent u_isn)u_hdip)))	24	10
4445	(((u_isn u_csn)(ca_ent ca_wa))(((p_re ca_wa)(cp_ent oa_ent))(u_hdip u_hdip)))	21	10
4446	(((u_isn u_csn)(cp_ent ca_wa))((p_re ca_wa)((cp_ent oa_ent)(oa_wa u_hdip))))	17	10
4447	((((ca_ent ca_wa)p_re)((ca_wa (p_csn cp_ent))(oa_ent oa_wa)))(u_isn oa_wa))	25	10
4448	(((ca_ent ca_wa)((p_re ca_wa)(p_csn oa_ent)))((oa_wa u_hdip)(u_isn oa_wa)))	22	10
4449	((ca_ent ca_wa)(((p_isn p_re)(ca_wa (p_csn cp_ent)))(oa_ent (u_isn u_hdip))))	32	10
4450	((ca_ent ca_wa)(((p_isn p_re)(ca_wa (p_csn cp_ent)))((oa_ent u_isn)u_hdip)))	33	10
4451	(((cp_ent ca_ent)((ca_wa ca_wa)(cp_ent oa_ent)))((oa_wa u_hdip)(u_isn oa_wa)))	24	10
4452	(((cp_ent ca_ent)((ca_wa ca_wa)(p_csn oa_ent)))((oa_wa u_hdip)(u_isn oa_wa)))	21	10
4453	((((cp_ent ca_ent)(ca_wa p_re))(ca_wa (p_csn cp_ent)))(oa_ent (u_isn u_hdip)))	30	10
4454	(((cp_ent ca_ent)((ca_wa p_re)(p_csn oa_ent)))((oa_wa u_hdip)(u_isn oa_wa)))	22	10
4455	(((cp_ent ca_ent)((p_re ca_wa)(cp_ent oa_ent)))((oa_wa u_hdip)(u_isn oa_wa)))	32	10
4456	((((cp_ent ca_ent)(ca_wa p_re))(ca_wa (p_csn cp_ent)))((oa_ent oa_wa)u_hdip))	27	10
4457	((((cp_ent ca_ent)(ca_wa p_re))(ca_wa (p_csn cp_ent)))((oa_ent u_isn)u_hdip))	32	10
4458	(((cp_ent ca_ent)(((ca_wa p_re)ca_wa)(p_csn oa_ent)))((oa_wa u_hdip)oa_wa))	23	10
4459	(((cp_ent ca_ent)((p_re ca_wa)(p_csn oa_ent)))((oa_wa u_hdip)(u_isn oa_wa)))	23	10
4460	(((cp_ent ca_wa)((p_re ca_wa)(p_csn oa_ent)))((oa_wa u_hdip)(u_isn oa_wa)))	20	10
4461	((((cp_ent oa_ent)oa_wa)((u_isn oa_wa)u_csn))((cp_ent oa_ent)(oa_wa u_hdip)))	17	10
4462	((((cp_ent oa_ent)oa_wa)((u_isn oa_wa)u_csn))((cp_ent oa_ent)(u_isn u_hdip)))	17	10
4463	(((oa_ent (oa_wa u_hdip))((u_isn u_hdip)(oa_wa (u_csn cp_ent))))(oa_ent oa_wa))	18	10
4464	((((oa_wa (u_csn cp_ent))p_csn)(ca_ent ca_wa))((p_re ca_wa)(cp_ent oa_ent)))	22	10
4465	(((oa_wa u_csn)(p_csn (p_isn ca_ent)))(((ca_wa p_re)ca_wa)(cp_ent oa_ent)))	18	10
4466	(((oa_wa u_csn)(p_csn (p_isn ca_ent)))(((ca_wa p_re)cp_ent)(oa_ent u_isn)))	18	10
4467	(((oa_wa u_csn)(p_csn (p_isn ca_ent)))(((ca_wa p_re)cp_ent)(oa_ent u_hdip)))	18	10
4468	(((oa_wa (u_csn cp_ent))((ca_ent ca_wa)p_re))((cp_ent oa_ent)(u_isn u_hdip)))	30	10
4469	(((oa_wa u_hdip)((u_csn cp_ent)(p_csn ca_ent)))(((ca_wa p_re)ca_wa)oa_ent))	18	10
4470	(((p_csn oa_ent)((u_isn oa_wa)u_hdip))((u_isn oa_wa)((u_csn cp_ent)p_csn)))	22	10
4471	(((p_isn ca_ent)((ca_wa ca_wa)(cp_ent oa_ent)))((oa_wa u_hdip)(u_isn oa_wa)))	18	10
4472	(((p_isn ca_wa)((p_re ca_wa)(cp_ent oa_ent)))((oa_wa u_hdip)(u_isn oa_wa)))	31	10
4473	(((p_isn ca_wa)((p_re ca_wa)(p_csn oa_ent)))((oa_wa u_hdip)(u_isn oa_wa)))	18	10

4474	(((u_csn (cp_ent ca_ent))((ca_wa p_re) ca_wa))((cp_ent oa_ent)(u_isn u_hdip)))	34	10
4475	(((u_csn (cp_ent ca_ent))((ca_wa p_re) ca_wa))((p_csn oa_ent)(u_isn u_hdip)))	29	10
4476	(((u_csn (cp_ent ca_ent))((ca_wa p_re) ca_wa))((p_csn (oa_ent u_isn)) u_hdip))	30	10
4477	((((u_csn cp_ent)(ca_ent ca_wa)))((p_re ca_wa)(cp_ent oa_ent)))((oa_wa u_hdip))	24	10
4478	(((u_csn cp_ent)(ca_ent ca_wa)))((p_re ca_wa)(cp_ent oa_ent)))((u_isn u_hdip))	32	10
4479	(((u_csn cp_ent)((ca_ent p_isn)(ca_wa p_csn)))((cp_ent oa_ent)(u_isn u_hdip)))	25	10
4480	(((u_csn cp_ent)(p_csn (p_isn ca_ent))))((ca_wa p_re) ca_wa)(cp_ent oa_ent)))	21	10
4481	(((u_csn cp_ent)(p_csn (p_isn ca_ent))))((ca_wa p_re) ca_wa)(oa_ent u_isn)))	18	10
4482	(((u_csn cp_ent)(p_csn (p_isn ca_ent))))((ca_wa p_re) cp_ent)(oa_ent u_isn)))	21	10
4483	(((u_csn cp_ent)(p_csn (p_isn ca_ent))))((ca_wa p_re) cp_ent)(oa_ent u_hdip)))	21	10
4484	(((u_csn cp_ent)(p_csn (p_isn ca_ent))))((ca_wa p_re) cp_ent)(p_isn oa_ent)))	16	10
4485	(((u_csn cp_ent)((p_isn ca_ent)(ca_wa p_re)))((p_csn oa_ent)(u_isn u_hdip)))	25	10
4486	(((u_hdip oa_wa)(u_csn (cp_ent ca_ent))))((ca_wa p_re) ca_wa)(oa_ent u_isn)))	21	10
4487	(((u_hdip oa_wa)(u_csn (cp_ent ca_ent))))((ca_wa p_re) cp_ent)(oa_ent u_isn)))	25	10
4488	(((u_hdip oa_wa)(u_csn (cp_ent ca_ent))))((ca_wa p_re) cp_ent)(oa_ent oa_wa)))	20	10
4489	(((u_hdip oa_wa)(u_csn (cp_ent ca_ent))))((ca_wa p_re) cp_ent)(oa_ent u_hdip)))	25	10
4490	(((u_hdip (u_isn oa_wa))((u_csn cp_ent)(p_csn ca_ent))))((ca_wa p_re) cp_ent))	25	10
4491	(((u_hdip oa_wa))((u_csn cp_ent)(p_csn ca_ent))))((ca_wa p_re) ca_wa) oa_ent))	18	10
4492	(((u_hdip u_hdip))((u_csn cp_ent)(p_csn ca_ent))))((ca_wa p_re) ca_wa) oa_ent))	16	10
4493	((((u_isn cp_ent) ca_ent)((ca_wa p_re) ca_wa))((p_csn cp_ent)(oa_ent u_isn)))	17	10
4494	((((cp_ent oa_ent)(oa_wa u_hdip)))((u_isn u_hdip)(oa_wa u_csn)))((u_isn cp_ent))	30	10
4495	((((cp_ent oa_ent)(u_isn u_hdip)))((u_isn u_hdip)(oa_wa u_csn)))((u_isn cp_ent))	29	10
4496	(((oa_wa (u_csn cp_ent))(p_isn ca_ent))((ca_wa p_re)(p_csn (oa_ent u_isn))))	19	10
4497	(((oa_wa (u_csn cp_ent))(p_isn ca_ent)))((p_re ca_wa)((cp_ent oa_ent) oa_wa)))	19	10
4498	(((oa_wa (u_csn cp_ent))(p_isn ca_ent)))((ca_wa p_re) ca_wa)(cp_ent oa_ent)))	26	10
4499	(((oa_wa (u_csn cp_ent))(p_isn ca_ent)))((ca_wa p_re) ca_wa)(oa_ent u_isn)))	20	10
4500	(((oa_wa (u_csn cp_ent))(p_isn ca_ent)))((ca_wa p_re) ca_wa)(p_csn oa_ent)))	20	10
4501	(((oa_wa (u_csn cp_ent))(p_isn ca_ent)))((ca_wa p_re) cp_ent)(oa_ent u_isn)))	25	10
4502	(((oa_wa (u_csn cp_ent))(p_isn ca_ent)))((ca_wa p_re) cp_ent)(oa_ent oa_wa)))	20	10
4503	(((oa_wa (u_csn cp_ent))(p_isn ca_ent)))((ca_wa p_re) cp_ent)(oa_ent u_hdip)))	25	10
4504	(((oa_wa (u_csn cp_ent))(p_isn ca_ent)))((p_re ca_wa) p_csn)(oa_ent u_isn)))	21	10
4505	(((oa_wa (u_csn cp_ent))(p_isn ca_wa)))((p_re ca_wa)((cp_ent oa_ent) oa_wa)))	21	10
4506	(((oa_wa (u_csn cp_ent))(p_isn ca_wa)))((p_re ca_wa)((cp_ent oa_ent) u_hdip)))	22	10
4507	(((oa_wa (u_csn cp_ent))(p_isn ca_wa)))((p_re ca_wa) oa_ent)(u_hdip u_hdip)))	17	10
4508	(((oa_wa (u_csn cp_ent))((ca_wa p_re) ca_wa))((cp_ent oa_ent)(u_isn u_hdip)))	28	10
4509	(((oa_wa (u_csn cp_ent))((ca_wa p_re) ca_wa))((p_csn oa_ent)(u_isn u_hdip)))	24	10
4510	(((p_csn (ca_ent ca_wa)))((p_re ca_wa) p_csn)))((p_isn oa_ent)(u_isn u_hdip)))	24	10
4511	(((p_csn (oa_ent u_hdip))(u_isn oa_wa)))((u_csn cp_ent) p_csn)(ca_ent p_isn)))	25	10
4512	(((p_csn (oa_ent u_hdip))(u_isn oa_wa)))((u_csn cp_ent) p_csn)(ca_ent ca_wa)))	25	10
4513	((((u_csn cp_ent)(ca_ent ca_wa))(p_isn ca_wa)))((cp_ent oa_ent)(u_isn u_hdip)))	33	10
4514	((((u_csn cp_ent)(ca_ent ca_wa))(p_isn ca_wa)))((cp_ent oa_ent)(oa_wa u_hdip)))	30	10
4515	((((u_csn cp_ent)(ca_ent ca_wa))(p_isn ca_wa)))((p_csn (oa_ent u_isn)) u_hdip))	28	10
4516	(((u_csn (ca_ent ca_wa))(p_isn ca_wa)))((p_csn (oa_ent u_isn))(u_hdip u_hdip)))	29	10
4517	((((u_csn cp_ent)(ca_ent ca_wa))(p_re p_re)))((p_csn (oa_ent u_isn)) u_hdip))	25	10
4518	((((u_csn cp_ent)(ca_ent ca_wa))(p_re p_re)))((p_csn (p_isn oa_ent)) u_hdip))	20	10
4519	((((u_csn cp_ent)(ca_ent p_isn))(p_re p_re)))((p_csn oa_ent)(u_isn u_hdip)))	23	10
4520	((((u_csn cp_ent)(ca_wa p_re))(ca_wa p_csn)))((cp_ent oa_ent)(u_isn u_hdip)))	24	10
4521	(((u_csn (p_isn ca_ent))(p_re p_re))(p_csn ((p_isn oa_ent)(u_isn u_hdip))))	19	10
4522	(((u_csn (u_isn cp_ent)) ca_ent)(((ca_wa p_re) cp_ent))((oa_ent u_isn) u_hdip)))	23	10
4523	(((u_csn (u_isn cp_ent))((ca_wa p_re) ca_wa))((cp_ent oa_ent)(u_isn u_hdip)))	23	10
4524	(((u_csn (u_isn cp_ent))(p_isn ca_ent)))((ca_wa p_re) ca_wa)(cp_ent oa_ent)))	21	10
4525	(((u_csn (u_isn cp_ent))(p_isn ca_ent)))((ca_wa p_re) cp_ent)(oa_ent u_isn)))	21	10
4526	(((u_csn (u_isn cp_ent))(p_isn ca_ent)))((ca_wa p_re) cp_ent)(oa_ent u_hdip)))	21	10
4527	(((u_hdip (oa_wa u_csn))(ca_ent ca_wa)))((p_re ca_wa)((cp_ent oa_ent) oa_wa)))	19	10
4528	(((u_hdip (oa_wa u_csn))(cp_ent ca_ent)))((ca_wa p_re) cp_ent)(oa_ent u_isn)))	23	10
4529	(((u_hdip (oa_wa u_csn))(cp_ent ca_ent)))((ca_wa p_re) cp_ent)(oa_ent u_hdip)))	23	10
4530	(((u_hdip (oa_wa u_csn))(cp_ent ca_wa)))((p_re ca_wa)((cp_ent oa_ent) oa_wa)))	18	10
4531	(((u_hdip (oa_wa u_csn))(cp_ent ca_wa)))((p_re ca_wa)((cp_ent oa_ent) u_hdip)))	19	10
4532	(((u_hdip (oa_wa u_csn))(p_csn (ca_ent ca_wa))))((p_re ca_wa)(cp_ent oa_ent)))	18	10
4533	(((u_hdip (u_csn cp_ent))(p_csn (ca_ent ca_wa))))((p_re ca_wa)(cp_ent oa_ent)))	20	10
4534	(((u_hdip (u_csn cp_ent))(p_csn ca_ent)))((ca_wa p_re) ca_wa)(oa_ent u_isn)))	18	10
4535	(((u_hdip (u_csn cp_ent))(p_csn ca_ent)))((ca_wa p_re) cp_ent)(oa_ent u_isn)))	20	10
4536	(((u_hdip (u_csn cp_ent))(p_isn ca_ent)))((ca_wa p_re) ca_wa)(cp_ent oa_ent)))	23	10
4537	(((u_hdip (u_csn cp_ent))(p_isn ca_ent)))((ca_wa p_re) cp_ent)(oa_ent u_hdip)))	22	10
4538	(((u_hdip (u_csn cp_ent))(p_isn ca_ent)))((p_re ca_wa) oa_ent)(u_hdip u_hdip)))	17	10
4539	(((u_hdip (u_csn cp_ent))(p_isn ca_wa)))((p_re ca_wa)((cp_ent oa_ent) oa_wa)))	20	10
4540	(((u_hdip (u_csn cp_ent))(p_isn ca_wa)))((p_re ca_wa)((cp_ent oa_ent) u_hdip)))	24	10
4541	(((u_hdip (u_csn cp_ent))(p_csn (p_isn ca_ent))))((ca_wa p_re) ca_wa) oa_ent))	15	10
4542	(((u_hdip (u_csn cp_ent))(p_csn (p_isn ca_wa))))(p_csn (p_isn oa_ent)) u_hdip))	18	10
4543	(((u_hdip (u_isn oa_wa)))((u_csn cp_ent)(ca_ent ca_wa))))((p_re ca_wa) oa_ent))	19	10
4544	(((u_hdip (u_isn oa_wa))(u_csn (cp_ent ca_ent))))((ca_wa p_re) ca_wa) oa_ent))	21	10
4545	(((u_hdip (u_isn oa_wa))(u_csn (cp_ent ca_ent))))((ca_wa p_re) cp_ent) oa_ent))	24	10
4546	(((u_hdip (u_isn u_csn))(ca_ent ca_wa)))((p_re ca_wa)((cp_ent oa_ent) u_hdip)))	23	10
4547	(((u_hdip (u_isn u_csn))(cp_ent ca_ent)))((ca_wa p_re) ca_wa)(cp_ent oa_ent)))	24	10
4548	(((u_hdip (u_isn u_csn))(cp_ent ca_ent)))((ca_wa p_re) cp_ent)(oa_ent u_isn)))	23	10
4549	(((u_hdip (u_isn u_csn))(cp_ent ca_ent)))((ca_wa p_re) cp_ent)(oa_ent u_hdip)))	23	10
4550	(((u_hdip (u_isn u_csn))(cp_ent ca_wa)))((p_re ca_wa)((cp_ent oa_ent) oa_wa)))	17	10
4551	(((u_hdip (u_isn u_csn))(cp_ent ca_wa)))((p_re ca_wa)((cp_ent oa_ent) u_hdip)))	18	10
4552	((((ca_ent ca_wa) cp_ent)((p_csn oa_ent)(u_isn u_hdip))))((u_isn oa_wa) u_hdip))	22	10
4553	(((ca_ent p_isn)))((p_re ca_wa) p_csn)(oa_ent (u_isn u_hdip))))((u_isn u_hdip))	34	10
4554	(((ca_ent p_isn)))((p_re ca_wa) p_csn))(oa_ent oa_wa) u_hdip)))(u_isn u_hdip))	28	10
4555	((ca_ent ca_wa)))((p_re ca_wa) p_csn))(p_isn oa_ent) u_hdip))(u_isn u_hdip)))	25	10
4556	(((ca_ent ca_wa)))((p_re ca_wa)(p_csn cp_ent))(oa_ent oa_wa)))(u_isn oa_wa))	27	10
4557	(((ca_wa p_re) cp_ent)(oa_ent (u_isn u_hdip)))((oa_wa u_hdip)(u_isn oa_wa)))	27	10
4558	(((ca_wa p_re) cp_ent)((oa_ent u_isn) u_hdip)))((oa_wa u_hdip)(u_isn oa_wa)))	29	10
4559	((((cp_ent ca_ent)(ca_wa p_re))(cp_ent oa_ent)))((oa_wa u_hdip)(u_isn oa_wa)))	29	10
4560	((((cp_ent ca_ent)(ca_wa p_re))(p_csn oa_ent)))((oa_wa u_hdip)(u_isn oa_wa)))	19	10
4561	((((cp_ent ca_ent)(ca_wa p_re)))((p_csn cp_ent)(oa_ent oa_wa)))((u_isn oa_wa))	25	10
4562	((((cp_ent ca_ent)(ca_wa p_re)))((p_csn cp_ent)(oa_ent oa_wa)))((u_isn u_hdip))	25	10
4563	((((cp_ent oa_ent)(oa_wa u_hdip)))((u_isn oa_wa) u_csn))(oa_ent (oa_wa u_hdip)))	17	10
4564	((((cp_ent oa_ent)(oa_wa u_hdip)))((u_isn oa_wa) u_csn))(oa_ent (u_isn u_hdip)))	17	10
4565	((((oa_ent oa_wa)((u_isn u_hdip))(u_isn oa_wa) u_csn))) oa_ent)(oa_wa u_hdip))	16	10
4566	((((oa_wa u_csn) p_csn) ca_ent)(((ca_wa p_re) cp_ent))((oa_ent u_isn) u_hdip)))	24	10
4567	((((oa_wa u_csn) p_csn)(p_isn ca_ent)))((ca_wa p_re) ca_wa)(cp_ent oa_ent)))	20	10
4568	((((oa_wa u_csn) p_csn)(p_isn ca_ent)))((ca_wa p_re) cp_ent)(oa_ent u_isn)))	20	10
4569	((((oa_wa u_csn) p_csn)(p_isn ca_ent)))((ca_wa p_re) cp_ent)(oa_ent u_hdip)))	20	10
4570	((((oa_wa u_csn)(cp_ent ca_wa)))((p_re ca_wa)))((cp_ent oa_ent)(oa_wa u_hdip)))	25	10
4571	((((oa_wa u_csn)(cp_ent ca_wa)))((p_re ca_wa)(cp_ent oa_ent)))((oa_wa oa_wa))	23	10
4572	((((oa_wa u_csn)(cp_ent ca_wa)))((p_re ca_wa)(cp_ent oa_ent)))((oa_wa u_hdip))	24	10
4573	((((oa_wa u_csn)(cp_ent ca_wa)))((p_re ca_wa)(cp_ent oa_ent)))((u_isn oa_wa))	24	10
4574	((((oa_wa u_csn)(p_isn ca_ent)))((ca_wa p_re) ca_wa)))((cp_ent oa_ent) u_hdip))	25	10
4575	((((oa_wa u_hdip) oa_wa)(u_csn (cp_ent ca_ent))))((ca_wa p_re) cp_ent) oa_ent))	26	10
4576	((((oa_wa u_hdip) oa_wa)(u_csn (cp_ent ca_wa))))((p_re ca_wa)(cp_ent oa_ent))	22	10
4577	((((oa_wa u_hdip) oa_wa))((u_csn cp_ent)(p_csn ca_ent))))((ca_wa p_re) cp_ent))	23	10
4578	((((oa_wa u_hdip)(oa_wa u_csn))(cp_ent ca_ent))))((ca_wa p_re) cp_ent) oa_ent))	20	10
4579	((((oa_wa u_hdip)(u_isn oa_wa))(u_csn (cp_ent ca_ent))))((ca_wa p_re) cp_ent))	24	10
4580	((((oa_wa u_hdip)(u_isn oa_wa)))((u_csn cp_ent) p_csn))((ca_ent ca_wa) cp_ent))	22	10
4581	((((oa_wa u_hdip)(u_isn oa_wa)))((u_csn cp_ent)(ca_ent ca_wa)))((ca_wa p_csn))	22	10
4582	((((p_csn oa_ent)(u_isn u_hdip))(u_isn oa_wa))))((u_csn cp_ent) p_csn) ca_ent))	25	10
4583	((((p_isn ca_ent)(ca_wa p_re))(cp_ent oa_ent)))((oa_wa u_hdip)(u_isn oa_wa)))	23	10
4584	((((p_re ca_wa)(cp_ent oa_ent)))((oa_wa u_hdip)(u_isn oa_wa)))((u_csn cp_ent))	25	10
4585	((((u_csn cp_ent) p_csn)(ca_ent ca_wa)))((p_re ca_wa)((cp_ent oa_ent) u_hdip)))	24	10
4586	((((u_csn cp_ent) p_csn)(p_isn ca_ent)))((ca_wa p_re) ca_wa)(cp_ent oa_ent)))	23	10
4587	((((u_csn cp_ent) p_csn)(p_isn ca_ent)))((ca_wa p_re) ca_wa)(oa_ent u_isn)))	20	10
4588	((((u_csn cp_ent) p_csn)(p_isn ca_ent)))((ca_wa p_re) cp_ent)(oa_ent u_isn)))	23	10
4589	((((u_csn cp_ent) p_csn)(p_isn ca_ent)))((ca_wa p_re) cp_ent)(oa_ent u_hdip)))	23	10
4590	((((u_csn cp_ent)(ca_wa p_re))(p_isn ca_wa)))((cp_ent oa_ent)(u_isn u_hdip)))	24	10
4591	((((u_csn cp_ent)(ca_wa p_re))(p_isn ca_wa)))((p_csn oa_ent)(u_isn u_hdip)))	21	10
4592	((((u_csn cp_ent)(ca_wa p_re))((cp_ent oa_ent)(u_isn u_hdip)))((u_isn oa_wa))	24	10
4593	((((u_csn cp_ent)(ca_wa p_re))((cp_ent oa_ent)(u_isn u_hdip)))((u_isn u_hdip))	30	10
4594	((((u_csn cp_ent)(p_isn ca_ent)))((ca_wa p_re) ca_wa)))((cp_ent oa_ent) u_hdip))	32	10
4595	((((u_csn cp_ent)(p_isn ca_ent))(p_isn ca_wa)))((cp_ent oa_ent)(oa_wa u_hdip)))	25	10
4596	((((u_csn cp_ent)(p_isn ca_ent))(p_re p_re)))((p_csn oa_ent)(u_isn u_hdip)))	22	10
4597	((((u_csn cp_ent)(p_isn ca_ent)))((p_re ca_wa) p_csn))(oa_ent (u_isn u_hdip)))	26	10
4598	((((u_csn cp_ent)(p_isn ca_wa))(p_isn ca_wa)))((cp_ent oa_ent)(oa_wa u_hdip)))	25	10
4599	((((u_csn cp_ent)(p_isn ca_wa))(p_isn ca_wa)))((cp_ent oa_ent)(u_isn u_hdip)))	24	10
4600	((((u_csn cp_ent)(p_isn ca_wa))(p_isn p_re)))((cp_ent oa_ent)(u_isn u_hdip)))	27	10
4601	((((u_csn cp_ent)(p_isn ca_wa))(p_re ca_wa)))((cp_ent oa_ent)(oa_wa u_hdip)))	27	10
4602	((((u_csn cp_ent)(p_isn p_re))(ca_wa p_csn)))((cp_ent oa_ent)(u_isn u_hdip)))	22	10
4603	((((u_hdip oa_wa) u_csn)(ca_ent ca_wa)))((p_re ca_wa)(p_csn (oa_ent u_isn)))	21	10
4604	((((u_hdip oa_wa) u_csn)(cp_ent ca_ent)))((ca_wa p_re)(p_csn (oa_ent u_hdip))))	17	10
4605	((((u_hdip oa_wa) u_csn)(cp_ent ca_ent)))((ca_wa p_re)(p_csn (oa_ent u_isn))))	19	10
4606	((((u_hdip oa_wa) u_csn)(ca_ent ca_wa)))((p_re ca_wa)((p_csn oa_ent) u_hdip)))	20	10
4607	((((u_hdip oa_wa) u_csn)(ca_ent ca_wa)))((p_re ca_wa)(p_csn (p_isn oa_ent)))	18	10
4608	((((u_hdip oa_wa) u_csn)(ca_ent ca_wa)))((p_re ca_wa)((cp_ent oa_ent) oa_wa)))	22	10
4609	((((u_hdip oa_wa) u_csn)(cp_ent ca_ent)))((ca_wa p_re) ca_wa)(oa_ent u_isn)))	22	10
4610	((((u_hdip oa_wa) u_csn)(cp_ent ca_ent)))((ca_wa p_re) cp_ent)(oa_ent u_isn)))	26	10
4611	((((u_hdip oa_wa) u_csn)(cp_ent ca_ent)))((ca_wa p_re) cp_ent)(oa_ent oa_wa)))	21	10
4612	((((u_hdip oa_wa) u_csn)(cp_ent ca_ent)))((ca_wa p_re) cp_ent)(oa_ent u_hdip)))	26	10
4613	((((u_hdip oa_wa) u_csn)(ca_ent ca_wa)))((p_re ca_wa) oa_ent)(u_hdip u_hdip)))	20	10
4614	((((u_hdip oa_wa) u_csn)(ca_ent ca_wa)))((p_re ca_wa) oa_ent)(u_isn u_hdip)))	20	10
4615	((((u_hdip oa_wa) u_csn)(ca_ent ca_wa)))((p_re ca_wa) p_csn)(oa_ent u_isn)))	22	10
4616	((((u_hdip oa_wa) u_csn)(ca_ent ca_wa)))((p_re ca_wa) p_csn)(oa_ent u_hdip)))	22	10
4617	((((u_hdip oa_wa) u_csn)(ca_ent ca_wa)))((p_re ca_wa) p_csn)(p_isn oa_ent)))	19	10
4618	((((u_hdip oa_wa) u_csn)(cp_ent ca_wa)))((p_re ca_wa)((cp_ent oa_ent) oa_wa)))	21	10
4619	((((u_hdip oa_wa) u_csn)(cp_ent ca_wa)))((p_re ca_wa)((cp_ent oa_ent) u_hdip)))	24	10
4620	((((u_hdip oa_wa) u_csn)(cp_ent ca_wa)))((p_re ca_wa) oa_ent)(u_hdip u_hdip)))	16	10
4621	((((u_hdip u_csn)(ca_ent ca_wa))(p_isn ca_wa)))((cp_ent oa_ent)(oa_wa u_hdip)))	23	10
4622	((((u_hdip u_csn)(cp_ent ca_ent))((ca_wa p_re) ca_wa))((cp_ent oa_ent) u_hdip))	29	10
4623	((((u_hdip u_csn)(cp_ent ca_ent))(p_isn ca_wa)))((cp_ent oa_ent)(oa_wa u_hdip)))	24	10
4624	((((u_isn cp_ent) ca_ent)((ca_wa p_re) ca_wa)))((cp_ent oa_ent)(u_isn u_hdip)))	25	10
4625	((((u_isn cp_ent)(p_isn ca_wa))(p_re ca_wa)))((cp_ent oa_ent)(oa_wa u_hdip)))	19	10
4626	((((u_isn cp_ent)(p_isn ca_wa))(p_re ca_wa)))((cp_ent oa_ent)(u_isn u_hdip)))	19	10
4627	((((u_isn oa_wa) u_csn)(cp_ent ca_ent)))((ca_wa p_re) ca_wa)(cp_ent oa_ent)))	26	10
4628	((((u_isn oa_wa) u_csn)(cp_ent ca_ent)))((ca_wa p_re) ca_wa)(oa_ent u_isn)))	22	10
4629	((((u_isn oa_wa) u_csn)(cp_ent ca_ent)))((ca_wa p_re) cp_ent)(oa_ent u_isn)))	25	10
4630	((((u_isn oa_wa) u_csn)(cp_ent ca_ent)))((ca_wa p_re) cp_ent)(oa_ent oa_wa)))	20	10
4631	((((u_isn oa_wa) u_csn)(cp_ent ca_ent)))((ca_wa p_re) cp_ent)(oa_ent u_hdip)))	25	10
4632	((((u_isn oa_wa) u_csn)(cp_ent ca_wa)))((p_re ca_wa)((cp_ent oa_ent) oa_wa)))	20	10
4633	((((u_isn oa_wa) u_csn)(cp_ent ca_wa)))((p_re ca_wa)((cp_ent oa_ent) u_hdip)))	21	10
4634	((((u_isn oa_wa) u_hdip)(u_csn (cp_ent ca_ent))))((ca_wa ca_wa)(cp_ent oa_ent)))	23	10
4635	((((u_isn oa_wa) u_hdip)(u_csn (cp_ent ca_ent))))((ca_wa p_re) ca_wa) oa_ent))	18	10
4636	((((u_isn oa_wa) u_hdip)(u_csn (cp_ent ca_ent))))((ca_wa p_re) cp_ent) oa_ent))	21	10
4637	((((u_isn oa_wa) u_hdip))((u_csn cp_ent)(p_csn (ca_ent ca_wa))))((p_re ca_wa))	25	10
4638	((((u_isn oa_wa) u_hdip))((u_csn cp_ent)(p_csn ca_ent))))((ca_wa p_re) cp_ent))	25	10
4639	((((u_isn u_csn)(cp_ent ca_ent))(p_isn ca_wa)))((cp_ent oa_ent)(oa_wa u_hdip)))	21	10
4640	((((u_isn u_csn)(cp_ent ca_ent))((ca_wa p_re) ca_wa)))((cp_ent oa_ent) u_hdip))	27	10
4641	((((u_isn u_csn)(cp_ent ca_wa))(p_isn ca_wa)))((cp_ent oa_ent)(oa_wa u_hdip)))	19	10
4642	(p_csn ((p_isn ca_ent)(((ca_wa p_re) ca_wa)((cp_ent oa_ent)(u_isn u_hdip)))))	21	10
4643	((p_csn ((p_isn ca_ent)((p_re ca_wa)((cp_ent oa_ent) u_hdip))))((u_isn u_hdip))	24	10
4644	((ca_ent ca_wa)((p_re ca_wa)(((p_csn cp_ent)(oa_ent u_hdip)))(u_isn u_hdip))))	31	10
4645	((ca_ent ca_wa)))((p_re ca_wa)(p_csn cp_ent))(oa_ent oa_wa)))(u_isn u_hdip)))	28	10
4646	((ca_ent ca_wa)((p_re ca_wa)(((p_csn oa_ent) u_hdip))((u_isn oa_wa) u_hdip))))	28	10
4647	((ca_ent ca_wa)((p_re ca_wa)(((p_csn oa_ent)(u_isn u_hdip))(oa_wa u_hdip))))	31	10
4648	((ca_ent ca_wa)((p_re ca_wa)(((p_csn oa_ent)(u_isn u_hdip))(u_isn oa_wa))))	28	10
4649	((ca_ent ca_wa)((p_re ca_wa)(((p_csn oa_ent)(u_isn u_hdip))(u_isn u_hdip))))	36	10
4650	((ca_ent ca_wa)))((p_isn p_re)(ca_wa (p_csn cp_ent)))((p_csn (oa_ent u_isn))))	20	10
4651	((ca_ent ca_wa)))((p_isn p_re)(ca_wa (p_csn cp_ent)))((p_csn oa_ent) oa_wa)))	19	10
4652	((ca_ent ca_wa)))((p_re ca_wa)((cp_ent oa_ent)(oa_wa u_hdip)))((u_isn u_hdip)))	34	10
4653	((ca_ent ca_wa)))((p_re ca_wa))(cp_ent oa_ent)(u_isn u_hdip)))((u_isn oa_wa)))	33	10
4654	((ca_ent ca_wa)))((p_re ca_wa)(p_csn oa_ent))(u_isn u_hdip))(oa_wa u_hdip)))	32	10
4655	((ca_ent ca_wa)))((p_re ca_wa)(p_csn oa_ent))(u_isn u_hdip))(u_isn u_hdip)))	37	10
4656	((ca_ent ca_wa)))((p_re ca_wa))(p_csn oa_ent) u_hdip))((u_isn oa_wa) u_hdip)))	31	10
4657	((ca_ent ca_wa)))((p_re ca_wa))(p_csn oa_ent)(oa_wa u_hdip)))(u_isn u_hdip)))	27	10
4658	((ca_ent ca_wa)))((p_re (ca_wa p_csn)))(oa_ent oa_wa) u_hdip))(u_isn u_hdip)))	24	10
4659	((ca_ent ca_wa)))((p_re ca_wa)(p_csn cp_ent))(oa_ent u_hdip))(u_isn u_hdip)))	32	10
4660	((ca_ent ca_wa)((((p_isn ca_wa) p_csn)(oa_ent (oa_wa u_hdip)))(u_isn u_hdip)))	23	10
4661	((ca_ent ca_wa)))((p_re ca_wa)(p_csn cp_ent))(oa_ent oa_wa))(u_isn oa_wa)))	26	10
4662	((ca_ent p_isn)))((p_re (ca_wa p_csn)) oa_ent)(u_hdip u_hdip))(u_isn u_hdip)))	27	10
4663	(((ca_ent p_isn)))((p_re (ca_wa p_csn)) oa_ent)(u_isn u_hdip)))(u_isn u_hdip))	29	10
4664	((ca_ent ca_wa)((((p_isn ca_wa) p_csn)(oa_ent (u_isn u_hdip)))(u_isn u_hdip)))	28	10
4665	((ca_ent ca_wa)((((p_isn ca_wa) p_csn))(p_isn oa_ent) u_hdip))(u_isn u_hdip)))	20	10
4666	((ca_ent ca_wa)))((p_re ca_wa) p_csn) oa_ent)((oa_wa u_hdip)(u_isn oa_wa))))	24	10
4667	((ca_ent ca_wa)))((p_re ca_wa) p_csn)(cp_ent oa_ent))((u_isn oa_wa) u_hdip)))	26	10
4668	((ca_ent ca_wa)))((p_re ca_wa) p_csn)(oa_ent (oa_wa u_hdip)))(u_isn u_hdip)))	28	10
4669	((ca_ent ca_wa)))((p_re ca_wa) p_csn)(oa_ent (u_isn u_hdip)))(u_isn u_hdip)))	35	10
4670	((ca_ent ca_wa)))((p_re ca_wa)(cp_ent oa_ent))(oa_wa u_hdip))(u_isn oa_wa)))	32	10
4671	((ca_ent ca_wa)))((p_re ca_wa)(cp_ent oa_ent))(oa_wa u_hdip))(u_isn u_hdip)))	35	10
4672	((ca_ent ca_wa)))((p_re ca_wa)(cp_ent oa_ent))(u_hdip u_hdip))(u_isn oa_wa)))	35	10
4673	((ca_ent ca_wa)))((p_re ca_wa)(cp_ent oa_ent))(u_hdip u_hdip))(u_isn u_hdip)))	39	10
4674	((ca_ent ca_wa)))((p_re ca_wa)(cp_ent oa_ent))(u_isn u_hdip))(u_isn oa_wa)))	37	10
4675	((ca_ent ca_wa)))((p_re ca_wa)(cp_ent oa_ent))(u_isn u_hdip))(u_isn u_hdip)))	39	10
4676	((ca_ent ca_wa)))((p_re ca_wa)(p_csn oa_ent))(oa_wa u_hdip))(u_isn u_hdip)))	26	10
4677	((ca_ent ca_wa)))((p_re ca_wa)(p_csn oa_ent))(u_hdip u_hdip))(u_isn u_hdip)))	33	10
4678	((ca_ent ca_wa)))((p_re ca_wa)(p_csn oa_ent))(u_isn u_hdip))(u_isn oa_wa)))	34	10
4679	((ca_ent p_isn)((p_re ca_wa)(((p_csn oa_ent) u_hdip))((u_isn oa_wa) u_hdip))))	26	10
4680	((ca_ent p_isn)((p_re ca_wa)(((p_csn oa_ent)(u_isn u_hdip))(oa_wa u_hdip))))	27	10
4681	((ca_ent p_isn)((p_re ca_wa)(((p_csn oa_ent)(u_isn u_hdip))(u_isn oa_wa))))	26	10
4682	((ca_ent p_isn)((p_re ca_wa)(((p_csn oa_ent)(u_isn u_hdip))(u_isn u_hdip))))	35	10
4683	((ca_ent p_isn)(((ca_wa (p_csn cp_ent))(oa_ent (u_isn u_hdip)))(u_isn u_hdip)))	27	10
4684	((ca_ent p_isn)(((ca_wa (p_csn cp_ent))((oa_ent u_isn) u_hdip))(u_isn oa_wa)))	20	10
4685	((ca_ent p_isn)(((p_re ca_wa)((cp_ent oa_ent)(oa_wa u_hdip)))(u_isn u_hdip)))	33	10
4686	((ca_ent p_isn)))((p_re ca_wa)(p_csn oa_ent))(u_isn u_hdip))(oa_wa u_hdip)))	28	10
4687	((ca_ent p_isn)))((p_re ca_wa)(p_csn oa_ent))(u_isn u_hdip))(u_isn u_hdip)))	36	10
4688	((ca_ent p_isn)))((p_re ca_wa)((p_csn oa_ent) u_hdip))((u_isn oa_wa) u_hdip)))	27	10
4689	((ca_ent p_isn)))((p_re ca_wa)((p_csn oa_ent)(oa_wa u_hdip)))(u_isn u_hdip)))	25	10
4690	((ca_ent p_isn)))((p_re ca_wa)(p_csn cp_ent))(oa_ent u_hdip))(u_isn u_hdip)))	31	10
4691	((ca_ent p_isn)))((p_re ca_wa)(p_csn cp_ent))(oa_ent oa_wa))(u_isn oa_wa)))	26	10
4692	((ca_ent p_isn)))((p_re ca_wa) p_csn) oa_ent)((oa_wa u_hdip)(u_isn oa_wa))))	22	10
4693	((ca_ent p_isn)))((p_re ca_wa) p_csn)(oa_ent (oa_wa u_hdip)))(u_isn u_hdip)))	26	10
4694	((ca_ent p_isn)))((p_re ca_wa) p_csn))(oa_ent oa_wa) u_hdip))(u_isn u_hdip)))	26	10
4695	((ca_ent p_isn)))((p_re ca_wa) p_csn))(p_isn oa_ent) u_hdip))(u_isn u_hdip)))	23	10
4696	((ca_ent p_isn)))((p_re ca_wa)(cp_ent oa_ent))(oa_wa u_hdip))(u_isn u_hdip)))	34	10
4697	((ca_ent p_isn)))((p_re ca_wa)(cp_ent oa_ent))(u_hdip u_hdip))(u_isn u_hdip)))	38	10
4698	((ca_ent p_isn)))((p_re ca_wa)(cp_ent oa_ent))(u_isn u_hdip))(u_isn oa_wa)))	36	10
4699	((ca_ent p_isn)))((p_re ca_wa)(cp_ent oa_ent))(u_isn u_hdip))(u_isn u_hdip)))	38	10
4700	((ca_ent p_isn)))((p_re ca_wa)(p_csn oa_ent))(oa_wa u_hdip))(u_isn u_hdip)))	22	10
4701	((ca_ent p_isn)))((p_re ca_wa)(p_csn oa_ent))(u_hdip u_hdip))(u_isn u_hdip)))	29	10
4702	((ca_ent p_isn)))((p_re ca_wa)(p_csn oa_ent))(u_isn u_hdip))(u_isn oa_wa)))	32	10
4703	((ca_wa ca_wa)))((p_isn p_re)(p_csn cp_ent))(oa_ent oa_wa))(u_isn oa_wa)))	20	10
4704	((ca_wa p_re)(((ca_wa (p_csn cp_ent))((oa_ent u_isn) u_hdip))(u_isn u_hdip)))	31	10
4705	((ca_wa p_re)(((ca_wa (p_csn cp_ent))(oa_ent (u_isn u_hdip)))(u_isn u_hdip)))	30	10
4706	((ca_wa p_re)((((ca_wa p_csn)(cp_ent oa_ent))(u_isn u_hdip))(u_isn u_hdip)))	31	10
4707	((cp_ent ca_ent)((ca_wa ca_wa)(((p_csn oa_ent)(u_isn u_hdip))(u_isn u_hdip))))	28	10
4708	((cp_ent ca_ent)((ca_wa p_re)(((p_csn oa_ent)(u_isn u_hdip))(u_isn u_hdip))))	31	10
4709	((cp_ent ca_ent)(((ca_wa p_re) ca_wa)(p_csn ((p_isn oa_ent)(u_isn u_hdip)))))	26	10
4710	((cp_ent ca_ent)(((ca_wa p_re) ca_wa)((p_csn (oa_ent u_hdip))(u_isn oa_wa))))	24	10
4711	((cp_ent ca_ent)(((ca_wa p_re) ca_wa)((p_csn (oa_ent u_isn))(u_hdip u_hdip))))	32	10
4712	((cp_ent ca_ent)(((ca_wa p_re) ca_wa)(p_csn (p_isn oa_ent)))(u_isn u_hdip)))	27	10
4713	((cp_ent ca_ent)(((ca_wa p_re) ca_wa)(p_csn (oa_ent oa_wa)))(u_hdip u_hdip)))	23	10
4714	((cp_ent ca_ent)(((ca_wa p_re) ca_wa)(p_csn (oa_ent oa_wa)))(u_isn u_hdip)))	27	10
4715	(((cp_ent ca_ent)(((ca_wa p_re) ca_wa)(p_csn (oa_ent u_hdip))))(u_isn u_hdip))	30	10
4716	((cp_ent ca_ent)(((ca_wa p_re) ca_wa)(p_csn (oa_ent u_isn)))(oa_wa u_hdip)))	29	10
4717	((cp_ent ca_ent)(((ca_wa p_re) ca_wa)(p_csn (oa_ent u_isn)))(u_hdip u_hdip)))	34	10
4718	((cp_ent ca_ent)(((ca_wa p_re) ca_wa)(p_csn (p_isn oa_ent)))(u_hdip u_hdip)))	24	10
4719	(((cp_ent ca_ent)((ca_wa p_re) ca_wa))((cp_ent oa_ent) oa_wa)))(u_isn oa_wa))	36	10
4720	((cp_ent ca_ent)(((ca_wa p_re) ca_wa)((cp_ent oa_ent) u_hdip))(u_isn oa_wa)))	34	10
4721	((cp_ent ca_ent)(((ca_wa p_re) ca_wa)((cp_ent oa_ent) u_hdip))(u_isn u_hdip)))	37	10
4722	((cp_ent ca_ent)(((ca_wa p_re) ca_wa)((p_csn oa_ent) u_hdip))(oa_wa u_hdip)))	26	10
4723	((cp_ent ca_ent)(((p_re ca_wa)(p_csn oa_ent))(u_isn u_hdip))(u_isn u_hdip)))	32	10
4724	((cp_ent ca_ent)(((ca_wa p_re) ca_wa)((p_csn oa_ent) u_hdip))(u_isn u_hdip)))	33	10
4725	((cp_ent ca_ent)(((p_re (ca_wa p_csn))((oa_ent oa_wa) u_hdip))(u_isn u_hdip)))	22	10
4726	((cp_ent ca_ent)((((p_isn ca_wa) p_csn)(oa_ent (oa_wa u_hdip)))(u_isn u_hdip)))	21	10
4727	((cp_ent ca_ent)((((ca_wa ca_wa)(cp_ent oa_ent))(u_hdip u_hdip))(u_isn oa_wa)))	24	10
4728	((cp_ent ca_ent)((((ca_wa ca_wa)(cp_ent oa_ent))(u_hdip u_hdip))(u_isn u_hdip)))	27	10
4729	((cp_ent ca_ent)((((ca_wa ca_wa)(cp_ent oa_ent))(u_isn u_hdip))(u_isn oa_wa)))	25	10
4730	((cp_ent ca_ent)((((ca_wa ca_wa)(cp_ent oa_ent))(u_isn u_hdip))(u_isn u_hdip)))	28	10
4731	((cp_ent ca_ent)((((ca_wa ca_wa)(p_csn oa_ent))(oa_wa u_hdip))(u_isn u_hdip)))	21	10
4732	((cp_ent ca_ent)((((ca_wa ca_wa)(p_csn oa_ent))(u_hdip u_hdip))(u_isn u_hdip)))	26	10
4733	((cp_ent ca_ent)((((ca_wa ca_wa)(p_csn oa_ent))(u_isn u_hdip))(u_isn u_hdip)))	29	10
4734	((cp_ent ca_ent)((((p_isn ca_wa) p_csn) oa_ent)(u_hdip u_hdip))(u_isn u_hdip)))	25	10
4735	(((cp_ent ca_ent)(((p_re (ca_wa p_csn)) oa_ent)(u_isn u_hdip)))(u_isn u_hdip))	27	10
4736	((cp_ent ca_ent)(((ca_wa p_re) ca_wa)(cp_ent oa_ent)))((u_hdip oa_wa) u_hdip)))	27	10
4737	((cp_ent ca_ent)(((ca_wa p_re) ca_wa)(cp_ent oa_ent)))((u_isn oa_wa) u_hdip)))	34	10
4738	((cp_ent ca_ent)(((ca_wa p_re) ca_wa)((cp_ent oa_ent) oa_wa))(u_hdip u_hdip)))	29	10
4739	((cp_ent ca_ent)(((ca_wa p_re) ca_wa)((cp_ent oa_ent) oa_wa))(u_isn oa_wa)))	35	10
4740	((cp_ent ca_ent)(((ca_wa p_re) ca_wa)((cp_ent oa_ent) oa_wa))(u_isn u_hdip)))	35	10
4741	((cp_ent ca_ent)(((ca_wa p_re) ca_wa)((p_csn oa_ent) oa_wa))(u_hdip u_hdip)))	24	10
4742	((cp_ent ca_ent)(((ca_wa p_re) ca_wa)((p_csn oa_ent) oa_wa))(u_isn u_hdip)))	30	10
4743	((cp_ent ca_ent)(((ca_wa p_re) cp_ent)((oa_ent u_isn) u_hdip))(u_isn u_hdip)))	40	10
4744	((cp_ent ca_ent)(((ca_wa p_re) cp_ent)((oa_ent u_isn) u_hdip))(u_isn oa_wa)))	34	10
4745	((cp_ent ca_ent)(((ca_wa p_re) cp_ent)(oa_ent (u_isn u_hdip)))(u_isn u_hdip)))	36	10
4746	((cp_ent ca_ent)(((ca_wa p_re)(p_csn oa_ent))(u_isn u_hdip))(u_isn u_hdip)))	31	10
4747	((cp_ent ca_ent)((((p_isn ca_wa) p_csn)(oa_ent (u_isn u_hdip)))(u_isn u_hdip)))	26	10
4748	((cp_ent ca_ent)((((p_isn ca_wa) p_csn)((p_isn oa_ent) u_hdip))(u_isn u_hdip)))	20	10
4749	((cp_ent ca_ent)((((p_re ca_wa) p_csn) oa_ent)((oa_wa u_hdip)(u_isn oa_wa)))	22	10
4750	((cp_ent ca_ent)((((p_re ca_wa) p_csn)(cp_ent oa_ent))((u_isn oa_wa) u_hdip)))	24	10
4751	((cp_ent ca_ent)((((p_re ca_wa) p_csn)(oa_ent (oa_wa u_hdip)))(u_isn u_hdip)))	24	10
4752	((cp_ent ca_ent)((((p_re ca_wa) p_csn)(oa_ent (u_isn u_hdip)))(u_isn u_hdip)))	30	10
4753	((cp_ent ca_ent)((((p_re ca_wa) p_csn)((oa_ent oa_wa) u_hdip))(u_isn u_hdip)))	24	10
4754	((cp_ent ca_ent)((((p_re ca_wa)(cp_ent oa_ent))(oa_wa u_hdip))(u_isn u_hdip)))	30	10
4755	((cp_ent ca_ent)((((p_re ca_wa)(cp_ent oa_ent))(u_isn u_hdip))(u_isn oa_wa)))	31	10
4756	((cp_ent ca_ent)((((p_re ca_wa)(cp_ent oa_ent))(u_isn u_hdip))(u_isn u_hdip)))	36	10
4757	((cp_ent ca_wa)((p_re ca_wa)(((p_csn oa_ent)(u_isn u_hdip))(u_isn u_hdip))))	32	10
4758	((cp_ent ca_wa)((((p_re ca_wa) p_csn)(oa_ent (u_isn u_hdip)))(u_isn u_hdip)))	31	10
4759	((cp_ent ca_wa)((((p_re ca_wa)(cp_ent oa_ent))(oa_wa u_hdip))(u_isn u_hdip)))	30	10
4760	((cp_ent ca_wa)((((p_re ca_wa)(cp_ent oa_ent))(u_hdip u_hdip))(u_isn oa_wa)))	30	10
4761	((cp_ent ca_wa)((((p_re ca_wa)(cp_ent oa_ent))(u_hdip u_hdip))(u_isn u_hdip)))	34	10
4762	((cp_ent ca_wa)((((p_re ca_wa)(cp_ent oa_ent))(u_isn u_hdip))(u_isn oa_wa)))	31	10
4763	((cp_ent ca_wa)((((p_re ca_wa)(cp_ent oa_ent))(u_isn u_hdip))(u_isn u_hdip)))	34	10
4764	((oa_ent oa_wa)((u_isn u_hdip)))((u_isn u_hdip)(oa_wa u_csn))(cp_ent oa_ent)))	21	10
4765	((oa_ent oa_wa)(((u_isn u_hdip))(u_isn u_hdip)(oa_wa u_csn)))(cp_ent oa_ent)))	22	10
4766	((oa_wa oa_wa)))((u_csn cp_ent)(p_csn (p_isn ca_ent))))((ca_wa p_re) ca_wa)))	16	10
4767	((oa_wa oa_wa)))((u_csn cp_ent) p_csn)(p_isn ca_ent)))((ca_wa p_re) ca_wa)))	18	10
4768	((oa_wa u_hdip)))((u_csn cp_ent) p_csn) ca_ent)))((ca_wa p_re) ca_wa) oa_ent)))	19	10
4769	(((p_csn (ca_ent ca_wa)))((p_re ca_wa)((cp_ent oa_ent) u_hdip)))((u_isn u_hdip))	29	10
4770	((p_csn ca_ent)((((ca_wa p_re) ca_wa)(cp_ent oa_ent))((u_hdip oa_wa) u_hdip)))	20	10
4771	((p_csn ca_ent)((((ca_wa p_re) ca_wa)((cp_ent oa_ent) oa_wa))(u_isn u_hdip)))	27	10
4772	((p_csn ca_ent)((((ca_wa p_re) cp_ent)((oa_ent u_isn) u_hdip))(u_isn u_hdip)))	30	10
4773	((p_csn ca_ent)((((p_re ca_wa)(cp_ent oa_ent))(u_isn u_hdip))(u_isn u_hdip)))	25	10
4774	((p_isn ca_ent)((ca_wa ca_wa)(((p_csn oa_ent)(u_isn u_hdip))(u_isn u_hdip))))	26	10
4775	((p_isn ca_ent)((ca_wa p_re)(((p_csn oa_ent)(u_isn u_hdip))(u_isn u_hdip))))	28	10
4776	((p_isn ca_ent)(((ca_wa p_re) ca_wa)((p_csn (oa_ent u_isn))(u_hdip u_hdip))))	30	10
4777	((p_isn ca_ent)((p_re ca_wa)(((p_csn oa_ent)(u_isn u_hdip))(oa_wa u_hdip))))	22	10
4778	((p_isn ca_ent)(((ca_wa p_re) ca_wa)(p_csn ((p_isn oa_ent)(u_isn u_hdip)))))	25	10
4779	((p_isn ca_ent)(((ca_wa p_re) ca_wa)((p_csn (oa_ent u_hdip))(u_isn oa_wa))))	20	10
4780	((p_isn ca_ent)(((ca_wa p_re) ca_wa)(p_csn (p_isn oa_ent)))(u_isn u_hdip)))	26	10
4781	((p_isn ca_ent)(((ca_wa p_re) ca_wa)(p_csn (oa_ent oa_wa)))(u_hdip u_hdip)))	19	10
4782	((p_isn ca_ent)(((ca_wa p_re) ca_wa)(p_csn (oa_ent oa_wa)))(u_isn u_hdip)))	21	10
4783	((p_isn ca_ent)(((ca_wa p_re) ca_wa)(p_csn (oa_ent u_hdip)))(u_isn u_hdip)))	28	10
4784	((p_isn ca_ent)(((ca_wa p_re) ca_wa)(p_csn (oa_ent u_isn)))(oa_wa u_hdip)))	23	10
4785	((p_isn ca_ent)(((ca_wa p_re) ca_wa)(p_csn (oa_ent u_isn)))(u_hdip u_hdip)))	31	10
4786	((p_isn ca_ent)(((ca_wa p_re) ca_wa)(p_csn (p_isn oa_ent)))(u_hdip u_hdip)))	25	10
4787	((p_isn ca_ent)(((ca_wa p_re) ca_wa)((cp_ent oa_ent) oa_wa))(u_isn oa_wa)))	31	10
4788	(((p_isn ca_ent)(((ca_wa p_re) ca_wa)((cp_ent oa_ent) u_hdip)))(u_isn oa_wa))	31	10
4789	(((p_isn ca_ent)(((ca_wa p_re) ca_wa)((cp_ent oa_ent) u_hdip)))(u_isn u_hdip))	34	10
4790	((p_isn ca_ent)(((p_re ca_wa)(p_csn oa_ent))(u_isn u_hdip))(oa_wa u_hdip)))	23	10
4791	((p_isn ca_ent)(((ca_wa p_re) ca_wa)((p_csn oa_ent) u_hdip))(oa_wa u_hdip)))	25	10
4792	((p_isn ca_ent)(((ca_wa p_re) ca_wa)((p_csn oa_ent) u_hdip))(u_isn u_hdip)))	30	10
4793	((p_isn ca_ent)(((p_re ca_wa)((p_csn oa_ent) u_hdip))((u_isn oa_wa) u_hdip)))	22	10
4794	((p_isn ca_ent)(((p_re (ca_wa p_csn))((oa_ent oa_wa) u_hdip))(u_isn u_hdip)))	18	10
4795	((p_isn ca_ent)((((ca_wa ca_wa)(cp_ent oa_ent))(u_isn u_hdip))(u_isn u_hdip)))	26	10
4796	((p_isn ca_ent)((((ca_wa ca_wa)(p_csn oa_ent))(u_isn u_hdip))(u_isn u_hdip)))	27	10
4797	((p_isn ca_ent)((((p_isn ca_wa) p_csn) oa_ent)(u_hdip u_hdip))(u_isn u_hdip)))	21	10
4798	(((p_isn ca_ent)(((p_re (ca_wa p_csn)) oa_ent)(u_isn u_hdip)))(u_isn u_hdip))	23	10
4799	((p_isn ca_ent)((((ca_wa p_re) ca_wa)(cp_ent oa_ent))((u_hdip oa_wa) u_hdip)))	24	10
4800	((p_isn ca_ent)((((ca_wa p_re) ca_wa)(cp_ent oa_ent))((u_isn oa_wa) u_hdip)))	31	10
4801	((p_isn ca_ent)((((ca_wa p_re) ca_wa)((cp_ent oa_ent) oa_wa))(u_hdip u_hdip)))	27	10
4802	((p_isn ca_ent)((((ca_wa p_re) ca_wa)((cp_ent oa_ent) oa_wa))(u_isn u_hdip)))	29	10
4803	((p_isn ca_ent)((((ca_wa p_re) ca_wa)((p_csn oa_ent) oa_wa))(u_hdip u_hdip)))	20	10
4804	((p_isn ca_ent)((((ca_wa p_re) ca_wa)((p_csn oa_ent) oa_wa))(u_isn u_hdip)))	26	10
4805	(((p_isn ca_ent)(((ca_wa p_re) cp_ent)((oa_ent u_isn) u_hdip)))(u_isn u_hdip))	35	10
4806	(((p_isn ca_ent)(((ca_wa p_re) cp_ent)(oa_ent u_hdip)))((u_isn oa_wa) u_hdip))	29	10
4807	((p_isn ca_ent)((((ca_wa p_re) cp_ent)((oa_ent u_isn) u_hdip))(u_isn oa_wa)))	31	10
4808	((p_isn ca_ent)((((ca_wa p_re) cp_ent)(oa_ent (oa_wa u_hdip)))(u_isn u_hdip)))	26	10
4809	((p_isn ca_ent)((((ca_wa p_re) cp_ent)(oa_ent (u_isn u_hdip)))(u_isn u_hdip)))	33	10
4810	((p_isn ca_ent)((((ca_wa p_re) cp_ent)((oa_ent oa_wa) u_hdip))(u_isn u_hdip)))	27	10
4811	((p_isn ca_ent)((((ca_wa p_re) cp_ent)((p_isn oa_ent) u_hdip))(u_isn u_hdip)))	23	10
4812	((p_isn ca_ent)((((ca_wa p_re)(p_csn oa_ent))(u_isn u_hdip))(u_isn u_hdip)))	28	10
4813	((p_isn ca_ent)((((p_isn ca_wa) p_csn)(oa_ent (u_isn u_hdip)))(u_isn u_hdip)))	22	10
4814	((p_isn ca_ent)((((p_isn ca_wa) p_csn)((p_isn oa_ent) u_hdip))(u_isn u_hdip)))	19	10
4815	((p_isn ca_ent)((((p_re ca_wa) p_csn) oa_ent)((oa_wa u_hdip)(u_isn oa_wa))))	18	10
4816	((p_isn ca_ent)((((p_re ca_wa) p_csn)(oa_ent (oa_wa u_hdip)))(u_isn u_hdip)))	20	10
4817	((p_isn ca_ent)((((p_re ca_wa) p_csn)(oa_ent (u_isn u_hdip)))(u_isn u_hdip)))	28	10
4818	((p_isn ca_ent)((((p_re ca_wa) p_csn)((oa_ent oa_wa) u_hdip))(u_isn u_hdip)))	20	10
4819	((p_isn ca_ent)((((p_re ca_wa)(cp_ent oa_ent))(oa_wa u_hdip))(u_isn u_hdip)))	28	10
4820	((p_isn ca_ent)((((p_re ca_wa)(cp_ent oa_ent))(u_isn u_hdip))(u_isn oa_wa)))	28	10
4821	((p_isn ca_ent)((((p_re ca_wa)(cp_ent oa_ent))(u_isn u_hdip))(u_isn u_hdip)))	32	10
4822	((p_isn ca_ent)((((p_re ca_wa)(p_csn oa_ent))(u_isn u_hdip))(u_isn oa_wa)))	24	10
4823	((p_isn ca_wa)((p_isn p_re)(((ca_wa p_csn)(oa_ent u_isn))(u_hdip u_hdip))))	29	10
4824	((p_isn ca_wa)((p_isn p_re)(((ca_wa p_csn)(oa_ent u_hdip))(u_isn u_hdip))))	28	10
4825	((p_isn ca_wa)((p_re ca_wa)(((p_csn oa_ent)(u_isn u_hdip))(u_isn oa_wa))))	28	10
4826	((p_isn ca_wa)((p_re ca_wa)(((p_csn oa_ent)(u_isn u_hdip))(u_isn u_hdip))))	32	10
4827	((p_isn ca_wa)(((p_re ca_wa)((cp_ent oa_ent)(oa_wa u_hdip)))(u_isn u_hdip)))	32	10
4828	((p_isn ca_wa)(((p_re ca_wa)((cp_ent oa_ent)(u_isn u_hdip)))(u_isn oa_wa)))	30	10
4829	((p_isn ca_wa)((((p_re ca_wa)(p_csn oa_ent))(u_isn u_hdip))(u_isn u_hdip)))	33	10
4830	((p_isn ca_wa)(((p_re ca_wa)((p_csn oa_ent)(oa_wa u_hdip)))(u_isn u_hdip)))	26	10
4831	((p_isn ca_wa)(((p_re (ca_wa p_csn))((oa_ent oa_wa) u_hdip))(u_isn u_hdip)))	23	10
4832	((p_isn ca_wa)((((p_isn p_re)(p_csn cp_ent)) oa_ent)((u_isn oa_wa) u_hdip)))	26	10
4833	((p_isn ca_wa)((((p_isn p_re)(p_csn cp_ent))(oa_ent oa_wa))(u_isn oa_wa)))	25	10
4834	((p_isn ca_wa)((((p_isn p_re)(p_csn cp_ent))(oa_ent oa_wa))(u_isn u_hdip)))	27	10
4835	((p_isn ca_wa)((((p_isn p_re)(p_csn cp_ent))(oa_ent u_hdip))(u_isn u_hdip)))	29	10
4836	((p_isn ca_wa)((((p_isn p_re)(p_csn cp_ent))(oa_ent u_isn))(oa_wa u_hdip)))	28	10
4837	((p_isn ca_wa)((((p_re ca_wa) p_csn) oa_ent)((oa_wa u_hdip)(u_isn oa_wa))))	24	10
4838	((p_isn ca_wa)((((p_re ca_wa) p_csn)(oa_ent (oa_wa u_hdip)))(u_isn u_hdip)))	28	10
4839	((p_isn ca_wa)((((p_re ca_wa) p_csn)(oa_ent (u_isn u_hdip)))(u_isn u_hdip)))	33	10
4840	((p_isn ca_wa)((((p_re ca_wa) p_csn)((oa_ent oa_wa) u_hdip))(u_isn u_hdip)))	27	10
4841	((p_isn ca_wa)((((p_re ca_wa) p_csn)((p_isn oa_ent) u_hdip))(u_isn u_hdip)))	25	10
4842	((p_isn ca_wa)((((p_re ca_wa)(cp_ent oa_ent))(oa_wa u_hdip))(u_isn oa_wa)))	30	10
4843	((p_isn ca_wa)((((p_re ca_wa)(cp_ent oa_ent))(oa_wa u_hdip))(u_isn u_hdip)))	33	10
4844	((p_isn ca_wa)((((p_re ca_wa)(cp_ent oa_ent))(u_hdip u_hdip))(u_isn oa_wa)))	33	10
4845	((p_isn ca_wa)((((p_re ca_wa)(cp_ent oa_ent))(u_hdip u_hdip))(u_isn u_hdip)))	37	10
4846	((p_isn ca_wa)((((p_re ca_wa)(cp_ent oa_ent))(u_isn u_hdip))(u_isn oa_wa)))	35	10
4847	((p_isn ca_wa)((((p_re ca_wa)(cp_ent oa_ent))(u_isn u_hdip))(u_isn u_hdip)))	37	10
4848	((p_isn ca_wa)((((p_re ca_wa)(p_csn oa_ent))(oa_wa u_hdip))(u_isn u_hdip)))	25	10
4849	((p_isn ca_wa)((((p_re ca_wa)(p_csn oa_ent))(u_hdip u_hdip))(u_isn u_hdip)))	31	10
4850	((p_isn ca_wa)((((p_re ca_wa)(p_csn oa_ent))(u_isn u_hdip))(u_isn oa_wa)))	30	10
4851	((p_isn p_re)(((ca_wa (p_csn cp_ent))((oa_ent u_isn) u_hdip))(u_isn u_hdip)))	30	10
4852	((p_isn p_re)(((ca_wa (p_csn cp_ent))(oa_ent (u_isn u_hdip)))(u_isn u_hdip)))	29	10
4853	((p_isn p_re)((((ca_wa p_csn)(cp_ent oa_ent))(u_isn u_hdip))(u_isn u_hdip)))	30	10
4854	((u_csn (cp_ent ca_ent))((ca_wa p_re)(p_csn ((p_isn oa_ent)(u_isn u_hdip)))))	18	10
4855	((u_csn (cp_ent ca_ent))(((ca_wa p_re) ca_wa)((p_csn (p_isn oa_ent)) u_hdip)))	21	10
4856	((u_csn (cp_ent ca_ent))(((ca_wa p_re)(p_csn (oa_ent u_hdip)))(u_isn u_hdip)))	21	10
4857	((u_csn (cp_ent ca_ent))(((ca_wa p_re)((p_csn oa_ent) u_hdip))(u_isn u_hdip)))	23	10
4858	((u_csn (cp_ent ca_ent))((((ca_wa p_re) ca_wa)(cp_ent oa_ent))(oa_wa u_hdip)))	29	10
4859	((u_csn (cp_ent ca_ent))((((ca_wa p_re) ca_wa)(cp_ent oa_ent))(u_hdip u_hdip)))	32	10
4860	((u_csn (cp_ent ca_ent))((((ca_wa p_re) ca_wa)(cp_ent oa_ent))(u_isn oa_wa)))	28	10
4861	((u_csn (cp_ent ca_ent))((((ca_wa p_re) ca_wa)(cp_ent oa_ent))(u_isn u_hdip)))	33	10
4862	((u_csn (cp_ent ca_ent))((((ca_wa p_re) ca_wa)(oa_ent u_isn))(u_hdip u_hdip)))	25	10
4863	((u_csn (cp_ent ca_ent))((((ca_wa p_re) ca_wa)(p_csn oa_ent))(u_isn u_hdip)))	28	10
4864	((u_csn (cp_ent ca_ent))((((ca_wa p_re) cp_ent)(oa_ent u_hdip))(u_isn u_hdip)))	32	10
4865	((u_csn (cp_ent ca_ent))((((ca_wa p_re) cp_ent)(oa_ent u_isn))(u_hdip u_hdip)))	32	10
4866	((u_csn (cp_ent ca_ent))((((ca_wa p_re) cp_ent) oa_ent)((u_hdip oa_wa) u_hdip)))	24	10
4867	((u_csn (cp_ent ca_ent))((((ca_wa p_re) cp_ent) oa_ent)((u_isn oa_wa) u_hdip)))	28	10
4868	((u_csn (cp_ent ca_ent))((((ca_wa p_re) cp_ent)(oa_ent oa_wa))(u_isn oa_wa)))	27	10
4869	((u_csn (cp_ent ca_ent))((((ca_wa p_re) cp_ent)(oa_ent oa_wa))(u_isn u_hdip)))	27	10
4870	((u_csn (cp_ent ca_ent))((((ca_wa p_re) cp_ent)(oa_ent u_hdip))(u_isn oa_wa)))	28	10
4871	((u_csn (cp_ent ca_ent))((((ca_wa p_re) cp_ent)(p_isn oa_ent))(u_hdip u_hdip)))	17	10
4872	((u_csn (cp_ent ca_ent))((((p_re ca_wa) p_csn)(oa_ent u_isn))(u_hdip u_hdip)))	28	10
4873	((u_csn cp_ent)((((ca_ent ca_wa) cp_ent)((oa_ent u_isn) u_hdip))(u_isn oa_wa)))	29	10
4874	(((u_hdip oa_wa)))((u_csn cp_ent) p_csn)(ca_ent ca_wa)))((p_re ca_wa) oa_ent))	18	10
4875	((u_hdip oa_wa)))((u_csn cp_ent) p_csn) ca_ent)))((ca_wa p_re) ca_wa) oa_ent)))	19	10
4876	((u_hdip u_hdip)(((u_isn oa_wa)((u_csn cp_ent) p_csn))((ca_ent ca_wa) cp_ent)))	23	10
4877	(((u_hdip u_hdip)))((u_isn oa_wa) u_csn)(cp_ent ca_ent)))((ca_wa p_re) ca_wa))	27	10
4878	((u_hdip u_hdip)))((u_isn oa_wa) u_csn)(ca_ent ca_wa)))((p_re ca_wa) oa_ent)))	18	10
4879	((u_hdip u_hdip)))((u_isn oa_wa) u_csn)(cp_ent ca_ent)))((ca_wa p_re) cp_ent)))	27	10
4880	((u_hdip u_hdip)))((u_isn oa_wa) u_csn)(p_csn (ca_ent ca_wa))))((p_re ca_wa)))	22	10

4881	((u_isn oa_wa)(((u_csn cp_ent)(p_csn ca_ent))(((ca_wa p_re)ca_wa)oa_ent)))	19	10
4882	(((u_isn oa_wa)((u_csn cp_ent)p_csn))((ca_ent ca_wa))((p_re ca_wa)oa_ent))	19	10
4883	((u_isn u_hdip)(((u_isn oa_wa)((u_csn cp_ent)p_csn))((ca_ent ca_wa)cp_ent)))	24	10
4884	(((u_isn u_hdip)((u_isn oa_wa)u_csn)(cp_ent ca_ent)))((ca_wa p_re)ca_wa))	26	10
4885	((u_isn u_hdip)(((u_isn oa_wa)u_csn)(cp_ent ca_ent)))((ca_wa p_re)cp_ent)))	25	10
4886	((u_isn u_hdip)(((u_isn oa_wa)u_csn)(p_csn(ca_ent ca_wa)))((p_re ca_wa)))	23	10
4887	((oa_ent(u_isn u_hdip))((u_isn u_hdip)((u_isn u_hdip))((u_isn oa_wa)u_csn))))	33	10
4888	((oa_wa(u_csn(cp_ent ca_ent)))(((ca_wa p_re))((p_csn(oa_ent u_isn))u_hdip)))	20	10
4889	((oa_wa(u_csn(cp_ent ca_ent)))((((ca_wa p_re)cp_ent)oa_ent)(oa_wa oa_wa)))	23	10
4890	((oa_wa(u_csn(cp_ent ca_ent)))((((ca_wa p_re)cp_ent)oa_ent)(u_isn oa_wa)))	24	10
4891	((oa_wa(u_csn(cp_ent ca_ent)))((((ca_wa p_re)cp_ent)oa_ent)(u_isn u_hdip)))	30	10
4892	((oa_wa(u_csn(cp_ent ca_ent)))((((p_re ca_wa)p_csn)oa_ent)(u_hdip u_hdip)))	24	10
4893	((oa_wa(u_csn(cp_ent ca_ent)))((((p_re ca_wa)p_csn)oa_ent)(u_isn u_hdip)))	26	10
4894	((p_csn(ca_ent ca_wa))(((p_re ca_wa)((cp_ent oa_ent)oa_wa))(u_isn u_hdip)))	26	10
4895	((p_csn(ca_ent ca_wa))(((p_re ca_wa)((cp_ent oa_ent)u_hdip))(u_isn oa_wa)))	26	10
4896	((p_csn(ca_ent ca_wa))((((p_re ca_wa)oa_ent)(u_isn u_hdip))(u_isn oa_wa)))	21	10
4897	((p_csn(p_isn ca_ent))((((ca_wa p_re)ca_wa)(cp_ent oa_ent))(u_hdip u_hdip)))	22	10
4898	((p_csn(p_isn ca_ent))((((ca_wa p_re)ca_wa)(cp_ent oa_ent))(u_isn u_hdip)))	21	10
4899	((p_csn(p_isn ca_wa))(((p_re ca_wa)((cp_ent oa_ent)u_hdip))(u_isn u_hdip)))	25	10
4900	((u_csn(ca_ent ca_wa))((((p_re ca_wa)oa_ent)(u_isn u_hdip))(u_isn oa_wa)))	20	10
4901	(((u_csn cp_ent)(ca_ent ca_wa))((((p_re ca_wa)p_csn)oa_ent)(u_hdip u_hdip)))	27	10
4902	((u_csn(cp_ent ca_wa))(((p_re ca_wa)((cp_ent oa_ent)oa_wa))(u_isn oa_wa)))	23	10
4903	((u_csn(cp_ent ca_wa))(((p_re ca_wa)((cp_ent oa_ent)oa_wa))(u_isn u_hdip)))	23	10
4904	((u_csn(cp_ent ca_wa))(((p_re ca_wa)((cp_ent oa_ent)u_hdip))(u_isn oa_wa)))	25	10
4905	((u_csn(cp_ent ca_wa))(((p_re ca_wa)((cp_ent oa_ent)u_hdip))(u_isn u_hdip)))	23	10
4906	((u_csn(cp_ent ca_wa))((((p_re ca_wa)oa_ent)(u_hdip u_hdip))(u_isn u_hdip)))	19	10
4907	((u_csn(cp_ent ca_wa))((((p_re ca_wa)oa_ent)(u_isn u_hdip))(u_isn oa_wa)))	18	10
4908	((((u_csn cp_ent)p_csn)ca_ent)((((ca_wa p_re)ca_wa)oa_ent)(u_hdip u_hdip)))	21	10
4909	((((u_csn cp_ent)p_csn)ca_ent)((((ca_wa p_re)cp_ent)oa_ent)(u_isn u_hdip)))	27	10
4910	(((u_csn cp_ent)(p_isn ca_ent))(((ca_wa p_re)((p_csn(p_isn oa_ent))u_hdip)))	17	10
4911	((u_csn(p_isn ca_ent))(((ca_wa p_re)(p_csn(oa_ent u_hdip)))(u_isn u_hdip)))	18	10
4912	((u_csn(p_isn ca_ent))(((p_re ca_wa)((cp_ent oa_ent)oa_wa))(u_isn u_hdip)))	20	10
4913	((u_csn(p_isn ca_ent))((((ca_wa p_re)ca_wa)(cp_ent oa_ent))(oa_wa u_hdip)))	23	10
4914	((u_csn(p_isn ca_ent))((((ca_wa p_re)ca_wa)(cp_ent oa_ent))(u_hdip u_hdip)))	26	10
4915	((u_csn(p_isn ca_ent))((((ca_wa p_re)ca_wa)(cp_ent oa_ent))(u_isn oa_wa)))	22	10
4916	((u_csn(p_isn ca_ent))((((ca_wa p_re)ca_wa)(cp_ent oa_ent))(u_isn u_hdip)))	27	10
4917	((u_csn(p_isn ca_ent))((((ca_wa p_re)ca_wa)(oa_ent u_isn))(u_hdip u_hdip)))	21	10
4918	((u_csn(p_isn ca_ent))((((ca_wa p_re)cp_ent)oa_ent)((u_isn oa_wa)u_hdip)))	22	10
4919	((u_csn(p_isn ca_ent))((((ca_wa p_re)cp_ent)(oa_ent oa_wa))(u_isn oa_wa)))	21	10
4920	((u_csn(p_isn ca_ent))((((ca_wa p_re)cp_ent)(oa_ent oa_wa))(u_isn u_hdip)))	22	10
4921	((u_csn(p_isn ca_ent))((((ca_wa p_re)cp_ent)(oa_ent u_hdip))(u_isn oa_wa)))	22	10
4922	((u_hdip(u_isn oa_wa))((u_csn(oa_ent oa_wa))(u_isn u_hdip))(u_isn u_hdip)))	21	10
4923	(((u_hdip(u_isn oa_wa))(((u_csn cp_ent)p_csn)(ca_ent p_isn)))((p_re ca_wa))	27	10
4924	((u_hdip(u_isn oa_wa))((((u_csn cp_ent)p_csn)ca_ent)((ca_wa p_re)ca_wa)))	27	10
4925	(((ca_wa p_re)ca_wa)((p_csn((p_isn oa_ent)(u_isn u_hdip)))(u_isn u_hdip)))	24	10
4926	(((ca_wa p_re)ca_wa)(((p_csn cp_ent)((oa_ent u_isn)u_hdip))(u_isn oa_wa)))	22	10
4927	(((ca_wa p_re)ca_wa)((((p_csn cp_ent)oa_ent)(u_isn u_hdip))(u_isn oa_wa)))	22	10
4928	(((ca_wa p_re)ca_wa)((((p_csn cp_ent)oa_ent)(u_isn u_hdip))(u_isn u_hdip)))	31	10
4929	(((cp_ent oa_ent)oa_wa)((u_isn u_hdip)((u_isn u_hdip)(oa_wa(u_csn cp_ent)))))	34	10
4930	(((cp_ent oa_ent)oa_wa)((u_isn u_hdip)((u_isn u_hdip)((u_isn oa_wa)u_csn))))	36	10
4931	(((cp_ent oa_ent)u_hdip)((u_isn u_hdip)((u_isn u_hdip)((u_isn oa_wa)u_csn))))	34	10
4932	(((oa_wa(u_csn cp_ent))ca_ent)((ca_wa p_re)((p_csn(oa_ent u_isn))u_hdip)))	21	10
4933	(((oa_wa(u_csn cp_ent))ca_ent)((((ca_wa p_re)ca_wa)oa_ent)(u_hdip u_hdip)))	25	10
4934	(((oa_wa(u_csn cp_ent))ca_ent)((((ca_wa p_re)ca_wa)oa_ent)(u_isn u_hdip)))	25	10
4935	(((oa_wa(u_csn cp_ent))ca_ent)((((ca_wa p_re)cp_ent)oa_ent)(oa_wa oa_wa)))	24	10
4936	(((oa_wa(u_csn cp_ent))ca_ent)((((ca_wa p_re)cp_ent)oa_ent)(u_isn oa_wa)))	26	10
4937	(((oa_wa(u_csn cp_ent))ca_ent)((((ca_wa p_re)cp_ent)oa_ent)(u_isn u_hdip)))	31	10
4938	(((oa_wa u_csn)(p_isn ca_ent))((((ca_wa p_re)cp_ent)oa_ent)(u_isn oa_wa)))	19	10
4939	(((oa_wa u_csn)(p_isn ca_ent))((((ca_wa p_re)cp_ent)oa_ent)(u_isn u_hdip)))	24	10
4940	(((oa_wa u_csn)(p_isn ca_ent))((((p_re ca_wa)p_csn)oa_ent)(u_hdip u_hdip)))	19	10
4941	(((oa_wa u_csn)(p_isn ca_ent))((((p_re ca_wa)p_csn)oa_ent)(u_isn u_hdip)))	20	10
4942	(((oa_wa u_hdip)oa_wa)((u_csn(cp_ent ca_ent))(((ca_wa p_re)cp_ent)oa_ent)))	24	10
4943	(((oa_wa u_hdip)oa_wa)((((u_csn cp_ent)p_csn)(ca_ent p_isn))((p_re ca_wa)))	24	10
4944	(((oa_wa u_hdip)oa_wa)((((u_csn cp_ent)p_csn)ca_ent)((ca_wa p_re)ca_wa)))	24	10
4945	(((oa_wa u_hdip)(u_isn oa_wa))((((u_csn cp_ent)p_csn)ca_ent)(ca_wa ca_wa)))	23	10
4946	(((oa_wa u_hdip)(u_isn oa_wa))((((u_csn cp_ent)p_csn)ca_ent)(ca_wa p_re)))	23	10
4947	(((p_isn oa_ent)oa_wa)((u_isn u_hdip)((u_isn u_hdip)(oa_wa(u_csn cp_ent)))))	26	10
4948	(((p_isn oa_ent)u_hdip)((u_isn u_hdip)((u_isn u_hdip)((u_isn oa_wa)u_csn))))	28	10
4949	(((u_csn cp_ent)(ca_ent ca_wa))((((p_re ca_wa)p_csn)oa_ent)(u_isn u_hdip)))	28	10
4950	(((u_csn cp_ent)(p_isn ca_ent))((((ca_wa p_re)ca_wa)oa_ent)(u_hdip u_hdip)))	23	10
4951	(((u_csn cp_ent)(p_isn ca_ent))((((ca_wa p_re)ca_wa)oa_ent)(u_isn u_hdip)))	23	10
4952	(((u_csn cp_ent)(p_isn ca_ent))((((ca_wa p_re)cp_ent)oa_ent)(oa_wa oa_wa)))	22	10
4953	(((u_csn cp_ent)(p_isn ca_ent))((((ca_wa p_re)cp_ent)oa_ent)(u_isn oa_wa)))	23	10
4954	(((u_csn cp_ent)(p_isn ca_ent))((((ca_wa p_re)cp_ent)oa_ent)(u_isn u_hdip)))	28	10
4955	(((u_csn cp_ent)(p_isn ca_ent))((((p_re ca_wa)p_csn)oa_ent)(u_hdip u_hdip)))	23	10
4956	(((u_csn cp_ent)(p_isn ca_ent))((((p_re ca_wa)p_csn)oa_ent)(u_isn u_hdip)))	24	10
4957	(((u_hdip oa_wa)u_hdip)((((u_csn cp_ent)p_csn)(ca_ent p_isn))((p_re ca_wa)))	20	10
4958	(((u_hdip oa_wa)u_hdip)((((u_csn cp_ent)p_csn)ca_ent)((ca_wa p_re)ca_wa)))	20	10
4959	(((u_hdip u_csn)(cp_ent ca_ent))((((ca_wa p_re)ca_wa)oa_ent)(u_hdip u_hdip)))	22	10
4960	(((u_hdip u_csn)(cp_ent ca_ent))((((ca_wa p_re)cp_ent)oa_ent)(u_isn oa_wa)))	20	10
4961	(((u_hdip u_csn)(cp_ent ca_ent))((((ca_wa p_re)cp_ent)oa_ent)(u_isn u_hdip)))	25	10
4962	(((u_isn cp_ent)ca_ent)((((ca_wa p_re)ca_wa)(cp_ent oa_ent))(oa_wa u_hdip)))	20	10
4963	(((u_isn cp_ent)ca_ent)((((ca_wa p_re)ca_wa)(cp_ent oa_ent))(u_hdip u_hdip)))	23	10
4964	(((u_isn cp_ent)ca_ent)((((ca_wa p_re)ca_wa)(cp_ent oa_ent))(u_isn oa_wa)))	20	10
4965	(((u_isn cp_ent)ca_ent)((((ca_wa p_re)ca_wa)(cp_ent oa_ent))(u_isn u_hdip)))	23	10
4966	(((u_isn cp_ent)ca_ent)((((ca_wa p_re)cp_ent)(oa_ent u_hdip))(u_isn u_hdip)))	22	10
4967	(((u_isn cp_ent)ca_ent)((((ca_wa p_re)cp_ent)oa_ent)((u_isn oa_wa)u_hdip)))	19	10
4968	(((u_isn cp_ent)(ca_ent ca_wa))((((p_re ca_wa)p_csn)oa_ent)(u_hdip u_hdip)))	19	10
4969	(((u_isn cp_ent)(p_isn ca_ent))((((ca_wa p_re)cp_ent)oa_ent)(u_isn u_hdip)))	21	10
4970	(((u_isn oa_wa)u_hdip)((u_isn oa_wa)((u_csn cp_ent)p_csn)(p_isn ca_wa))))	14	10
4971	(((u_isn oa_wa)u_hdip)(((u_csn cp_ent)(p_csn(p_isn ca_ent)))(ca_wa ca_wa)))	19	10
4972	(((u_isn oa_wa)u_hdip)(((u_isn oa_wa)((u_csn cp_ent)p_csn))(ca_ent p_isn)))	17	10
4973	(((u_isn oa_wa)u_hdip)(((u_isn oa_wa)((u_csn cp_ent)p_csn))(ca_wa ca_wa)))	16	10
4974	(((u_isn oa_wa)u_hdip)((u_csn(oa_ent oa_wa))(u_isn u_hdip))(u_isn u_hdip)))	16	10
4975	((((u_isn oa_wa)u_hdip)(((u_csn cp_ent)p_csn)(ca_ent p_isn)))((p_re ca_wa))	28	10
4976	(((u_isn oa_wa)u_hdip)((((u_csn cp_ent)p_csn)ca_ent)((ca_wa p_re)ca_wa)))	28	10
4977	(((u_isn oa_wa)u_hdip)((((u_isn oa_wa)u_csn)(cp_ent ca_ent))(ca_wa ca_wa)))	20	10
4978	(((u_isn oa_wa)u_hdip)((((u_isn oa_wa)u_csn)(cp_ent ca_ent))(ca_wa p_re)))	20	10
4979	(((u_isn oa_wa)u_hdip)((((u_isn oa_wa)u_csn)(cp_ent ca_wa))((p_re ca_wa)))	19	10
4980	(((u_isn u_csn)(cp_ent ca_ent))((((ca_wa p_re)cp_ent)oa_ent)(u_isn oa_wa)))	19	10
4981	(((u_isn u_csn)(cp_ent ca_ent))((((ca_wa p_re)cp_ent)oa_ent)(u_isn u_hdip)))	24	10
4982	(((u_csn(u_isn cp_ent))ca_ent)((((ca_wa p_re)cp_ent)oa_ent)(u_isn u_hdip)))	23	10
4983	((((oa_wa u_csn)p_csn)ca_ent)((((ca_wa p_re)cp_ent)oa_ent)(u_hdip u_hdip)))	23	10
4984	((((oa_wa u_csn)p_csn)ca_ent)((((ca_wa p_re)cp_ent)oa_ent)(u_isn u_hdip)))	24	10
4985	(((cp_ent oa_ent)((oa_wa u_hdip)((u_isn u_hdip)(oa_wa u_csn))))(oa_ent oa_wa))	17	10
4986	(((ca_ent ca_wa)((p_re ca_wa)((p_csn cp_ent)(oa_ent u_isn))))(oa_wa u_hdip))	30	10
4987	(((ca_ent ca_wa)((p_re ca_wa)((p_csn cp_ent)(oa_ent oa_wa))))(u_isn u_hdip))	29	10
4988	(((ca_ent ca_wa)((p_re ca_wa)((p_csn oa_ent)(oa_wa u_hdip))))(u_isn u_hdip))	28	10
4989	(((p_isn p_re)(((ca_wa p_csn)(oa_ent u_isn))(u_hdip u_hdip)))(u_isn u_hdip))	26	10
4990	(((p_isn p_re)(((ca_wa p_csn)(cp_ent oa_ent))(u_isn u_hdip)))(u_isn oa_wa))	26	10
4991	(((p_isn p_re)(((ca_wa p_csn)(oa_ent u_hdip))(u_isn u_hdip)))(u_isn u_hdip))	27	10
4992	(((p_isn p_re)((ca_wa(p_csn cp_ent))((oa_ent u_isn)u_hdip)))(u_isn oa_wa))	22	10
4993	(((ca_ent ca_wa)(((p_re ca_wa)p_csn)oa_ent))((oa_wa u_hdip)(u_isn oa_wa)))	26	10
4994	(((ca_ent ca_wa)(((p_re ca_wa)p_csn)(cp_ent oa_ent)))((u_isn oa_wa)u_hdip))	28	10
4995	(((ca_ent ca_wa)(((p_re ca_wa)p_csn)(oa_ent(oa_wa u_hdip))))(u_isn u_hdip))	30	10
4996	(((ca_ent ca_wa)(((p_re ca_wa)p_csn)((oa_ent oa_wa)u_hdip)))(u_isn u_hdip))	29	10
4997	((((ca_ent p_isn)((p_re ca_wa)(p_csn cp_ent)))(oa_ent oa_wa))(u_isn oa_wa))	27	10
4998	(((ca_ent ca_wa)(((p_re ca_wa)(p_csn cp_ent))(oa_ent oa_wa)))(u_isn u_hdip))	30	10
4999	(((ca_ent ca_wa)(((p_re ca_wa)(p_csn oa_ent))(oa_wa u_hdip)))(u_isn u_hdip))	27	10
5000	(((((ca_wa p_re)ca_wa)(p_csn oa_ent))(u_isn u_hdip))((u_isn oa_wa)u_hdip))	31	10
5001	(((ca_ent p_isn)((p_re ca_wa)((cp_ent oa_ent)(u_isn u_hdip))))(u_isn oa_wa))	32	10
5002	(((ca_ent p_isn)((p_re ca_wa)((p_csn oa_ent)u_hdip)))((u_isn oa_wa)u_hdip))	30	10
5003	(((ca_ent p_isn)((p_re ca_wa)((p_csn oa_ent)(u_isn u_hdip))))(oa_wa u_hdip))	30	10
5004	(((ca_ent p_isn)((p_re ca_wa)((p_csn oa_ent)(u_isn u_hdip))))(u_isn oa_wa))	31	10
5005	(((ca_ent p_isn)((ca_wa(p_csn cp_ent))((oa_ent u_isn)u_hdip)))(u_isn u_hdip))	28	10
5006	(((ca_ent p_isn)(((p_re ca_wa)p_csn)(cp_ent oa_ent)))((u_isn oa_wa)u_hdip))	27	10
5007	(((ca_ent p_isn)(((p_re ca_wa)(p_csn cp_ent))(oa_ent u_hdip)))(u_isn oa_wa))	25	10
5008	(((ca_ent p_isn)(((p_re ca_wa)(p_csn oa_ent))(u_hdip u_hdip)))(u_isn u_hdip))	32	10
5009	(((ca_ent p_isn)(((p_re ca_wa)(p_csn oa_ent))(u_isn u_hdip)))(oa_wa u_hdip))	31	10
5010	(((ca_ent p_isn)(((p_re ca_wa)(p_csn oa_ent))(u_isn u_hdip)))(u_isn oa_wa))	33	10
5011	(((ca_wa ca_wa)(((p_isn p_re)(p_csn cp_ent))(oa_ent oa_wa)))(u_isn u_hdip))	22	10
5012	((((ca_ent ca_wa)((p_isn ca_wa)p_csn))(oa_ent(u_isn u_hdip)))(u_isn u_hdip))	30	10
5013	(((ca_wa p_re)(((p_csn oa_ent)(u_isn u_hdip))(u_isn oa_wa)))(u_isn u_hdip))	23	10
5014	(((cp_ent ca_ent)((ca_wa p_re)((ca_wa p_csn)(p_isn cp_ent))))(oa_ent u_isn))	21	10
5015	(((cp_ent ca_ent)((p_isn ca_wa)((p_csn cp_ent)(oa_ent oa_wa))))(u_isn oa_wa))	24	10
5016	(((cp_ent ca_ent)(((ca_wa p_re)ca_wa)(p_csn(oa_ent u_isn))))(u_hdip u_hdip))	33	10
5017	(((cp_ent ca_ent)((p_re ca_wa)((cp_ent oa_ent)(oa_wa u_hdip))))(u_isn u_hdip))	31	10
5018	(((cp_ent ca_ent)((p_re ca_wa)((p_csn cp_ent)oa_ent)))((u_isn oa_wa)u_hdip))	26	10
5019	(((cp_ent ca_ent)((p_re ca_wa)((p_csn cp_ent)(oa_ent u_isn))))(oa_wa u_hdip))	28	10
5020	(((cp_ent ca_ent)((p_re ca_wa)((p_csn cp_ent)(oa_ent u_isn))))(u_hdip u_hdip))	31	10
5021	(((cp_ent ca_ent)((p_re ca_wa)((p_csn oa_ent)u_hdip)))((u_isn oa_wa)u_hdip))	28	10
5022	(((cp_ent ca_ent)((p_re ca_wa)((p_csn oa_ent)(u_isn u_hdip))))(u_isn u_hdip))	33	10
5023	(((cp_ent ca_ent)(((ca_wa ca_wa)(cp_ent oa_ent))(u_isn u_hdip)))(u_isn oa_wa))	26	10
5024	(((cp_ent ca_ent)(((ca_wa ca_wa)(p_csn oa_ent))(u_isn u_hdip)))(u_isn oa_wa))	24	10
5025	(((cp_ent ca_ent)(((ca_wa p_re)ca_wa)oa_ent))((oa_wa u_hdip)(u_isn oa_wa)))	28	10
5026	(((cp_ent ca_ent)(((ca_wa p_re)ca_wa)(cp_ent oa_ent)))((u_isn oa_wa)u_hdip))	36	10
5027	(((cp_ent ca_ent)(((ca_wa p_re)ca_wa)((cp_ent oa_ent)oa_wa)))(u_hdip u_hdip))	32	10
5028	(((cp_ent ca_ent)(((ca_wa p_re)ca_wa)((cp_ent oa_ent)oa_wa)))(u_isn u_hdip))	37	10
5029	(((cp_ent ca_ent)(((ca_wa p_re)ca_wa)((p_csn oa_ent)oa_wa)))(u_hdip u_hdip))	25	10
5030	(((cp_ent ca_ent)(((ca_wa p_re)ca_wa)((p_csn oa_ent)oa_wa)))(u_isn oa_wa))	28	10
5031	(((cp_ent ca_ent)(((ca_wa p_re)ca_wa)((p_csn oa_ent)oa_wa)))(u_isn u_hdip))	31	10
5032	(((cp_ent ca_ent)(((ca_wa p_re)ca_wa)((p_csn oa_ent)u_hdip)))(oa_wa u_hdip))	27	10
5033	(((cp_ent ca_ent)(((ca_wa p_re)cp_ent)oa_ent))((oa_wa u_hdip)(u_isn oa_wa)))	32	10
5034	(((cp_ent ca_ent)(((ca_wa p_re)cp_ent)(oa_ent u_hdip)))((u_isn oa_wa)u_hdip))	33	10
5035	(((cp_ent ca_ent)(((ca_wa p_re)cp_ent)(oa_ent(oa_wa u_hdip))))(u_isn u_hdip))	29	10
5036	(((cp_ent ca_ent)(((ca_wa p_re)cp_ent)(oa_ent(u_isn u_hdip))))(u_isn oa_wa))	34	10
5037	(((cp_ent ca_ent)(((ca_wa p_re)cp_ent)(oa_ent(u_isn u_hdip))))(u_isn u_hdip))	38	10
5038	(((cp_ent ca_ent)(((ca_wa p_re)cp_ent)((oa_ent oa_wa)u_hdip)))(u_isn u_hdip))	31	10
5039	(((cp_ent ca_ent)(((ca_wa p_re)cp_ent)((oa_ent u_isn)u_hdip)))(u_isn oa_wa))	36	10
5040	(((cp_ent ca_ent)(((ca_wa p_re)(p_csn oa_ent))(u_isn u_hdip)))(u_isn oa_wa))	27	10
5041	(((cp_ent ca_ent)(((p_isn p_re)(p_csn cp_ent))oa_ent))((u_isn oa_wa)u_hdip))	25	10
5042	(((cp_ent ca_ent)(((p_isn p_re)(p_csn cp_ent))(oa_ent u_isn)))(u_hdip u_hdip))	30	10
5043	(((cp_ent ca_ent)(((p_re ca_wa)p_csn)oa_ent))((oa_wa u_hdip)(u_isn oa_wa)))	25	10
5044	(((cp_ent ca_ent)(((p_re ca_wa)p_csn)(oa_ent(u_isn u_hdip))))(u_isn u_hdip))	32	10
5045	(((cp_ent ca_ent)(((p_re ca_wa)p_csn)((oa_ent oa_wa)u_hdip)))(u_isn u_hdip))	23	10
5046	(((cp_ent ca_ent)(((p_re ca_wa)(cp_ent oa_ent))(oa_wa u_hdip)))(u_isn u_hdip))	32	10
5047	(((cp_ent ca_ent)(((p_re ca_wa)(cp_ent oa_ent))(u_hdip u_hdip)))(u_isn oa_wa))	31	10
5048	(((cp_ent ca_ent)(((p_re ca_wa)(cp_ent oa_ent))(u_isn u_hdip)))(u_isn oa_wa))	34	10
5049	(((cp_ent ca_wa)((p_re ca_wa)((cp_ent oa_ent)(oa_wa u_hdip))))(u_isn u_hdip))	29	10
5050	(((cp_ent ca_wa)((p_re ca_wa)((p_csn oa_ent)u_hdip)))((u_isn oa_wa)u_hdip))	26	10
5051	(((cp_ent ca_wa)(((p_re ca_wa)p_csn)oa_ent))((oa_wa u_hdip)(u_isn oa_wa)))	21	10
5052	(((cp_ent ca_wa)(((p_re ca_wa)p_csn)((oa_ent oa_wa)u_hdip)))(u_isn u_hdip))	24	10
5053	(((cp_ent ca_wa)(((p_re ca_wa)(cp_ent oa_ent))(oa_wa u_hdip)))(u_isn u_hdip))	31	10
5054	((((cp_ent oa_ent)oa_wa)((u_isn u_hdip)((u_isn oa_wa)u_csn)))(u_isn cp_ent))	32	10
5055	(((oa_ent oa_wa)((u_isn u_hdip)((u_isn oa_wa)u_csn)))((oa_ent u_isn)u_hdip))	18	10
5056	(((oa_ent oa_wa)((u_isn u_hdip)((u_isn u_hdip)(oa_wa(u_csn cp_ent)))))oa_ent)	18	10
5057	((((oa_ent u_isn)u_hdip)((u_isn u_hdip)((u_isn oa_wa)u_csn)))(u_isn cp_ent))	31	10
5058	(((oa_wa oa_wa)((u_csn cp_ent)(p_csn(p_isn ca_ent))))((ca_wa p_re)cp_ent))	16	10
5059	(((oa_wa oa_wa)(((u_csn cp_ent)p_csn)(p_isn ca_ent)))((ca_wa p_re)cp_ent))	18	10
5060	(((p_csn ca_ent)(((ca_wa p_re)cp_ent)(oa_ent u_hdip)))((u_isn oa_wa)u_hdip))	24	10
5061	(((p_isn ca_ent)((ca_wa p_re)((p_csn oa_ent)u_hdip)))((u_isn oa_wa)u_hdip))	22	10
5062	(((p_isn ca_ent)((p_isn ca_wa)((p_csn cp_ent)(oa_ent oa_wa))))(u_isn oa_wa))	20	10
5063	(((p_isn ca_ent)(((ca_wa p_re)ca_wa)((cp_ent oa_ent)oa_wa)))(u_isn u_hdip))	29	10
5064	(((p_isn ca_ent)(((ca_wa p_re)ca_wa)((p_csn oa_ent)oa_wa)))(u_hdip u_hdip))	23	10
5065	(((p_isn ca_ent)((p_re ca_wa)((p_csn oa_ent)(oa_wa u_hdip))))(u_isn u_hdip))	21	10
5066	(((p_isn ca_ent)(((ca_wa ca_wa)(cp_ent oa_ent))(u_hdip u_hdip)))(u_isn u_hdip))	25	10
5067	(((p_isn ca_ent)(((ca_wa ca_wa)(p_csn oa_ent))(u_hdip u_hdip)))(u_isn u_hdip))	24	10
5068	(((p_isn ca_ent)(((ca_wa p_re)ca_wa)oa_ent))((oa_wa u_hdip)(u_isn oa_wa)))	20	10
5069	(((p_isn ca_ent)(((ca_wa p_re)ca_wa)(cp_ent oa_ent)))((u_isn oa_wa)u_hdip))	32	10
5070	(((p_isn ca_ent)(((ca_wa p_re)ca_wa)(p_csn(oa_ent oa_wa))))(u_hdip u_hdip))	22	10
5071	(((p_isn ca_ent)(((ca_wa p_re)ca_wa)(p_csn(oa_ent oa_wa))))(u_isn u_hdip))	23	10
5072	(((p_isn ca_ent)(((ca_wa p_re)ca_wa)((p_csn oa_ent)oa_wa)))(u_isn oa_wa))	26	10
5073	(((p_isn ca_ent)(((ca_wa p_re)cp_ent)oa_ent))((oa_wa u_hdip)(u_isn oa_wa)))	24	10
5074	(((p_isn ca_ent)(((ca_wa p_re)cp_ent)(oa_ent(u_isn u_hdip))))(u_isn oa_wa))	29	10
5075	(((p_isn ca_ent)(((ca_wa p_re)cp_ent)((oa_ent oa_wa)u_hdip)))(u_isn u_hdip))	26	10
5076	(((p_isn ca_ent)(((ca_wa p_re)cp_ent)((oa_ent u_isn)u_hdip)))(oa_wa oa_wa))	25	10
5077	(((p_isn ca_ent)(((p_re ca_wa)p_csn)(oa_ent(oa_wa u_hdip))))(u_isn u_hdip))	19	10
5078	(((p_isn ca_ent)(((p_re ca_wa)(cp_ent oa_ent))(oa_wa u_hdip)))(u_isn oa_wa))	27	10
5079	(((p_isn ca_ent)(((p_re ca_wa)(cp_ent oa_ent))(u_isn u_hdip)))(u_isn oa_wa))	29	10
5080	(((p_isn ca_ent)(((p_re ca_wa)(p_csn oa_ent))(oa_wa u_hdip)))(u_isn u_hdip))	20	10
5081	(((p_isn ca_wa)((p_re ca_wa)((cp_ent oa_ent)(u_isn u_hdip))))(u_isn oa_wa))	31	10
5082	(((p_isn ca_wa)((p_re ca_wa)((p_csn oa_ent)(oa_wa u_hdip))))(u_isn u_hdip))	27	10
5083	(((p_isn ca_wa)(((p_isn p_re)(p_csn cp_ent))(oa_ent u_isn)))(u_hdip u_hdip))	31	10
5084	(((p_isn ca_wa)(((p_re ca_wa)p_csn)oa_ent))((oa_wa u_hdip)(u_isn oa_wa)))	23	10
5085	(((p_isn ca_wa)(((p_re ca_wa)(p_csn oa_ent))(oa_wa u_hdip)))(u_isn u_hdip))	26	10
5086	(((p_isn p_re)((ca_wa(p_csn cp_ent))(oa_ent(u_isn u_hdip))))(u_isn oa_wa))	21	10
5087	(((u_csn(cp_ent ca_ent))(((ca_wa p_re)ca_wa)oa_ent))((oa_wa u_hdip)oa_wa))	20	10
5088	(((u_csn(cp_ent ca_ent))(((ca_wa p_re)ca_wa)(cp_ent oa_ent)))(oa_wa oa_wa))	26	10
5089	(((u_csn(cp_ent ca_ent))(((ca_wa p_re)ca_wa)(cp_ent oa_ent)))(u_isn u_hdip))	34	10
5090	(((u_csn(cp_ent ca_ent))(((ca_wa p_re)cp_ent)(oa_ent oa_wa)))(u_isn oa_wa))	26	10
5091	(((u_csn(cp_ent ca_ent))(((ca_wa p_re)cp_ent)(oa_ent u_hdip)))(u_isn u_hdip))	31	10
5092	(((u_csn cp_ent)(((ca_ent ca_wa)cp_ent)(oa_ent u_hdip)))((u_isn oa_wa)u_hdip))	23	10
5093	(((u_csn cp_ent)(((ca_ent ca_wa)cp_ent)(oa_ent(u_isn u_hdip))))(u_isn u_hdip))	28	10
5094	(((u_hdip u_hdip)((oa_wa(u_csn cp_ent))(p_csn(ca_ent ca_wa))))(p_re ca_wa))	25	10
5095	(((u_hdip(u_isn oa_wa))(((u_csn cp_ent)p_csn)ca_ent))((ca_wa p_re)cp_ent))	27	10
5096	(((u_hdip u_hdip)((u_isn oa_wa)((u_csn cp_ent)p_csn)))((ca_ent ca_wa)cp_ent))	26	10
5097	(((u_hdip u_hdip)((u_isn oa_wa)((u_csn cp_ent)p_csn)))((ca_wa p_re)cp_ent))	28	10
5098	(((u_hdip u_hdip)((u_isn oa_wa)((u_csn cp_ent)p_csn)))((p_isn oa_ent)u_hdip))	24	10
5099	(((u_hdip u_hdip)(((u_isn oa_wa)u_csn)(ca_ent ca_wa)))((p_re ca_wa)oa_ent))	19	10
5100	(((u_isn oa_wa)((u_hdip(u_csn cp_ent))(p_csn ca_ent)))((ca_wa p_re)ca_wa))	24	10
5101	(((u_isn oa_wa)((u_hdip(u_csn cp_ent))(p_csn ca_ent)))((ca_wa p_re)cp_ent))	24	10
5102	(((u_isn u_hdip)((u_isn oa_wa)((u_csn cp_ent)p_csn)))((ca_ent ca_wa)cp_ent))	25	10
5103	(((u_isn u_hdip)((u_isn u_hdip)((u_isn oa_wa)u_csn)))((u_isn oa_wa)u_hdip))	24	10
5104	(((oa_ent(oa_wa u_hdip))((u_isn u_hdip)((u_isn oa_wa)u_csn)))(cp_ent oa_ent))	28	10
5105	(((oa_ent(oa_wa u_hdip))((u_isn u_hdip)((u_isn oa_wa)u_csn)))(u_isn cp_ent))	30	10
5106	(((oa_ent(u_isn u_hdip))((u_isn u_hdip)((u_isn oa_wa)u_csn)))(cp_ent oa_ent))	34	10
5107	(((oa_ent(u_isn u_hdip))((u_isn u_hdip)((u_isn oa_wa)u_csn)))(u_isn cp_ent))	29	10
5108	(((oa_wa(u_csn(cp_ent ca_ent)))((((ca_wa p_re)cp_ent)oa_ent)(oa_wa u_hdip)))	25	10
5109	(((oa_wa(u_csn(cp_ent ca_ent)))((((p_re ca_wa)p_csn)oa_ent)(u_isn u_hdip)))	25	10
5110	(((u_csn(ca_ent ca_wa))((p_re ca_wa)(p_csn(p_isn oa_ent))))(u_isn u_hdip))	20	10
5111	(((u_csn(cp_ent ca_wa))((p_re ca_wa)((cp_ent oa_ent)u_hdip)))(u_isn u_hdip))	24	10
5112	(((u_csn(p_isn ca_ent))(((ca_wa p_re)ca_wa)(oa_ent u_isn)))(u_hdip u_hdip))	18	10
5113	(((u_hdip(u_isn oa_wa))((u_csn cp_ent)((p_isn oa_ent)oa_wa)))(u_hdip u_hdip))	24	10
5114	(((u_hdip(u_isn oa_wa))((u_csn cp_ent)((p_isn oa_ent)oa_wa)))(u_isn u_hdip))	27	10
5115	((((ca_wa p_re)ca_wa)(p_csn((p_isn oa_ent)(u_isn u_hdip))))(u_isn u_hdip))	25	10
5116	((((cp_ent oa_ent)u_hdip)((u_isn u_hdip)((u_isn oa_wa)u_csn)))(u_isn cp_ent))	31	10
5117	((((oa_ent u_isn)u_hdip)((u_isn u_hdip)((u_isn oa_wa)u_csn)))(cp_ent oa_ent))	36	10
5118	((((oa_wa u_hdip)oa_wa)(((u_csn cp_ent)p_csn)ca_ent))((ca_wa p_re)cp_ent))	24	10
5119	((((p_isn ca_ent)((ca_wa p_re)(p_csn(p_isn oa_ent))))u_hdip)(u_isn u_hdip))	23	10
5120	((((u_hdip oa_wa)u_csn)((cp_ent ca_ent)((ca_wa p_re)cp_ent)))(oa_ent u_hdip))	25	10
5121	((((u_hdip oa_wa)u_csn)((cp_ent ca_ent)((ca_wa p_re)cp_ent)))(oa_ent u_isn))	25	10
5122	((((u_isn oa_wa)u_hdip)(((u_csn cp_ent)p_csn)ca_ent))((ca_wa p_re)cp_ent))	28	10
5123	((((u_isn oa_wa)((u_csn cp_ent)p_csn))ca_ent)(((ca_wa p_re)ca_wa)oa_ent))	20	10
5124	((((u_isn oa_wa)u_hdip)(((u_csn cp_ent)p_csn)(p_isn ca_ent)))(ca_wa ca_wa))	18	10
5125	((((u_isn oa_wa)u_hdip)(((u_isn oa_wa)u_csn)(cp_ent ca_ent)))(ca_wa ca_wa))	24	10
5126	(((p_csn(oa_ent(u_isn u_hdip)))(u_isn oa_wa))((u_csn cp_ent)(p_csn ca_ent)))	22	10
5127	(((p_csn(oa_ent(u_isn u_hdip)))(u_isn oa_wa))(((u_csn cp_ent)p_csn)ca_ent))	26	10
5128	(((p_csn(oa_ent(u_isn u_hdip)))((u_isn oa_wa)u_hdip))((u_csn cp_ent)p_csn))	24	10
5129	(((p_csn((p_isn oa_ent)(u_isn u_hdip)))(u_isn oa_wa))((u_csn cp_ent)p_csn))	20	10
5130	((((ca_ent ca_wa)((p_isn ca_wa)p_csn))((p_isn oa_ent)u_hdip))(u_isn u_hdip))	21	10
5131	((((ca_ent ca_wa)((p_isn ca_wa)(p_csn cp_ent)))oa_ent)((oa_wa u_hdip)oa_wa))	28	10
5132	((((ca_ent ca_wa)((p_isn ca_wa)(p_csn cp_ent)))(oa_ent u_hdip))(u_isn u_hdip))	33	10
5133	((((ca_ent ca_wa)((p_re ca_wa)oa_ent))((oa_wa u_hdip)(u_isn oa_wa)))u_csn)	24	10
5134	((((ca_ent ca_wa)((p_re ca_wa)p_csn))oa_ent)((oa_wa u_hdip)(u_isn oa_wa)))	22	10
5135	((((ca_ent ca_wa)((p_re ca_wa)p_csn))(cp_ent oa_ent))((u_isn oa_wa)u_hdip))	27	10
5136	((((ca_ent ca_wa)((p_re ca_wa)p_csn))(oa_ent(oa_wa u_hdip)))(u_isn u_hdip))	29	10
5137	((((ca_ent ca_wa)((p_re ca_wa)p_csn))(oa_ent(u_isn u_hdip)))(u_isn oa_wa))	31	10
5138	((((ca_ent ca_wa)((p_re ca_wa)p_csn))((p_isn oa_ent)u_hdip))(u_isn u_hdip))	26	10
5139	((((ca_ent ca_wa)((p_re ca_wa)(cp_ent oa_ent)))(u_isn oa_wa))(u_csn cp_ent))	28	10
5140	((((ca_wa p_re)(ca_wa(p_csn cp_ent)))((oa_ent u_isn)u_hdip))(u_isn oa_wa))	23	10
5141	((((ca_wa p_re)(ca_wa(p_csn cp_ent)))((oa_ent u_isn)u_hdip))(u_isn u_hdip))	32	10
5142	((((p_isn p_re)(ca_wa(p_csn cp_ent)))(oa_ent(u_isn u_hdip)))(u_isn u_hdip))	30	10
5143	((((p_isn p_re)(ca_wa(p_csn cp_ent)))((oa_ent u_isn)u_hdip))(u_isn oa_wa))	25	10
5144	((((p_isn p_re)(ca_wa(p_csn cp_ent)))((oa_ent u_isn)u_hdip))(u_isn u_hdip))	31	10
5145	((((ca_ent p_isn)((p_re ca_wa)p_csn))oa_ent)((oa_wa u_hdip)(u_isn oa_wa)))	25	10
5146	((((ca_ent p_isn)((p_re ca_wa)p_csn))(oa_ent(oa_wa u_hdip)))(u_isn u_hdip))	28	10
5147	((((cp_ent ca_ent)((ca_wa ca_wa)(cp_ent oa_ent)))(oa_wa u_hdip))(u_isn oa_wa))	23	10
5148	((((cp_ent ca_ent)((ca_wa ca_wa)(cp_ent oa_ent)))(u_isn u_hdip))(u_isn u_hdip))	24	10
5149	((((cp_ent ca_ent)((ca_wa ca_wa)(p_csn oa_ent)))(u_hdip u_hdip))(u_isn u_hdip))	27	10
5150	((((cp_ent ca_ent)((ca_wa ca_wa)(p_csn oa_ent)))(u_isn u_hdip))(u_isn u_hdip))	30	10
5151	((((cp_ent ca_ent)((ca_wa p_re)cp_ent))oa_ent)((oa_wa u_hdip)(u_isn oa_wa)))	33	10
5152	((((cp_ent ca_ent)((ca_wa p_re)cp_ent))(oa_ent u_hdip))((u_isn oa_wa)u_hdip))	34	10
5153	((((cp_ent ca_ent)((ca_wa p_re)cp_ent))(oa_ent(oa_wa u_hdip)))(u_isn u_hdip))	31	10
5154	((((cp_ent ca_ent)((ca_wa p_re)cp_ent))(oa_ent(u_isn u_hdip)))(u_isn oa_wa))	35	10
5155	((((cp_ent ca_ent)((ca_wa p_re)cp_ent))(oa_ent(u_isn u_hdip)))(u_isn u_hdip))	39	10
5156	((((cp_ent ca_ent)((ca_wa p_re)cp_ent))((oa_ent oa_wa)u_hdip))(u_isn u_hdip))	32	10
5157	((((cp_ent ca_ent)((ca_wa p_re)cp_ent))((oa_ent u_isn)u_hdip))(oa_wa oa_wa))	32	10
5158	((((cp_ent ca_ent)((ca_wa p_re)cp_ent))((oa_ent u_isn)u_hdip))(u_isn oa_wa))	37	10
5159	((((cp_ent ca_ent)((ca_wa p_re)cp_ent))((oa_ent u_isn)u_hdip))(u_isn u_hdip))	41	10
5160	((((cp_ent ca_ent)((ca_wa p_re)cp_ent))((p_isn oa_ent)u_hdip))(u_isn u_hdip))	26	10
5161	((((cp_ent ca_ent)((ca_wa p_re)(p_csn cp_ent)))(oa_ent oa_wa))(u_isn oa_wa))	27	10
5162	((((cp_ent ca_ent)((ca_wa p_re)(p_csn cp_ent)))(oa_ent oa_wa))(u_isn u_hdip))	27	10
5163	((((cp_ent ca_ent)((ca_wa p_re)(p_csn oa_ent)))(oa_wa u_hdip))(u_isn u_hdip))	24	10
5164	((((cp_ent ca_ent)((ca_wa p_re)(p_csn oa_ent)))(u_isn u_hdip))(oa_wa u_hdip))	27	10
5165	((((cp_ent ca_ent)((ca_wa p_re)(p_csn oa_ent)))(u_isn u_hdip))(u_isn oa_wa))	28	10
5166	((((cp_ent ca_ent)((ca_wa p_re)(p_csn oa_ent)))(u_isn u_hdip))(u_isn u_hdip))	32	10
5167	((((cp_ent ca_wa)((p_re ca_wa)(p_csn cp_ent)))(oa_ent oa_wa))(u_isn oa_wa))	26	10
5168	((((cp_ent ca_wa)((p_re ca_wa)(p_csn cp_ent)))(oa_ent oa_wa))(u_isn u_hdip))	26	10
5169	((((cp_ent ca_wa)((p_re ca_wa)(p_csn cp_ent)))(oa_ent u_hdip))(u_isn u_hdip))	28	10
5170	((((cp_ent ca_wa)((p_re ca_wa)(p_csn cp_ent)))(oa_ent u_isn))(u_hdip u_hdip))	30	10
5171	((((oa_wa u_csn)((cp_ent ca_wa)cp_ent))(oa_ent u_isn))(u_hdip(u_isn oa_wa)))	23	10
5172	((((oa_wa u_csn)((cp_ent ca_wa)cp_ent))oa_ent)((oa_wa u_hdip)(u_isn oa_wa)))	20	10
5173	((((oa_wa u_csn)((cp_ent ca_wa)cp_ent))(oa_ent u_isn))((oa_wa u_hdip)oa_wa))	21	10
5174	((((oa_wa u_csn)((cp_ent ca_wa)cp_ent))(oa_ent u_isn))((u_hdip oa_wa)u_hdip))	21	10
5175	((((oa_wa u_csn)((cp_ent ca_wa)cp_ent))(oa_ent(u_isn u_hdip)))(u_isn oa_wa))	24	10
5176	((((oa_wa u_csn)((cp_ent ca_wa)cp_ent))(oa_ent(u_isn u_hdip)))(u_isn u_hdip))	23	10
5177	((((oa_wa u_hdip)(oa_wa(u_csn cp_ent)))(p_csn ca_ent))((ca_wa p_re)ca_wa))	22	10
5178	((((oa_wa u_hdip)(oa_wa(u_csn cp_ent)))(p_csn ca_ent))((ca_wa p_re)cp_ent))	22	10
5179	((((p_csn ca_ent)((p_re ca_wa)(cp_ent oa_ent)))(u_isn u_hdip))(u_isn u_hdip))	22	10
5180	((((p_csn oa_ent)((u_isn oa_wa)u_hdip))(u_isn oa_wa))((u_csn cp_ent)p_csn))	24	10
5181	((((p_isn ca_ent)((ca_wa ca_wa)(cp_ent oa_ent)))(u_isn u_hdip))(u_isn u_hdip))	20	10
5182	((((p_isn ca_ent)((ca_wa p_re)(p_csn oa_ent)))(u_isn u_hdip))(oa_wa u_hdip))	24	10
5183	((((p_isn ca_ent)((ca_wa p_re)(p_csn oa_ent)))(u_isn u_hdip))(u_isn oa_wa))	24	10
5184	((((p_isn ca_ent)((p_re ca_wa)oa_ent))((oa_wa u_hdip)(u_isn oa_wa)))u_csn)	18	10
5185	((((p_isn ca_ent)((p_re ca_wa)p_csn))(oa_ent(oa_wa u_hdip)))(u_isn u_hdip))	23	10
5186	((((p_isn ca_ent)((p_re ca_wa)p_csn))(oa_ent(u_isn u_hdip)))(u_isn u_hdip))	28	10
5187	((((p_isn ca_ent)((p_re ca_wa)p_csn))((oa_ent oa_wa)u_hdip))(u_isn u_hdip))	23	10
5188	((((p_isn ca_ent)((p_re ca_wa)p_csn))((p_isn oa_ent)u_hdip))(u_isn u_hdip))	25	10
5189	((((p_isn ca_ent)((p_re ca_wa)(cp_ent oa_ent)))(u_isn u_hdip))(u_isn oa_wa))	30	10
5190	((((p_isn ca_ent)((p_re ca_wa)(cp_ent oa_ent)))(u_isn u_hdip))(u_isn u_hdip))	33	10
5191	((((p_isn ca_wa)((p_re ca_wa)(p_csn oa_ent)))(oa_wa u_hdip))(u_isn u_hdip))	23	10
5192	(((((ca_wa p_re)ca_wa)(p_csn(oa_ent u_isn)))(u_hdip u_hdip))(u_isn u_hdip))	32	10
5193	(((((ca_wa p_re)ca_wa)(p_csn(p_isn oa_ent)))(u_isn u_hdip))(u_isn u_hdip))	26	10
5194	((((p_re ca_wa)((cp_ent oa_ent)oa_wa))(u_isn oa_wa))(u_hdip(u_isn u_csn)))	20	10
5195	(((((ca_wa p_re)ca_wa)((cp_ent oa_ent)u_hdip))(u_isn u_hdip))(u_isn oa_wa))	34	10
5196	(((((ca_wa p_re)ca_wa)((cp_ent oa_ent)u_hdip))(u_isn u_hdip))(u_isn u_hdip))	38	10
5197	((((p_re ca_wa)((cp_ent oa_ent)(oa_wa u_hdip)))(u_isn u_hdip))(u_isn u_hdip))	29	10
5198	(((((ca_wa p_re)ca_wa)((p_csn oa_ent)u_hdip))(u_isn u_hdip))(u_isn u_hdip))	32	10
5199	((((u_csn(cp_ent ca_ent))((ca_wa p_re)ca_wa))(cp_ent oa_ent))(oa_wa oa_wa))	27	10
5200	((((u_csn(cp_ent ca_ent))((ca_wa p_re)ca_wa))(cp_ent oa_ent))(oa_wa u_hdip))	29	10
5201	((((u_csn(cp_ent ca_ent))((ca_wa p_re)ca_wa))(cp_ent oa_ent))(u_hdip u_hdip))	33	10
5202	((((u_csn(cp_ent ca_ent))((ca_wa p_re)ca_wa))(cp_ent oa_ent))(u_isn oa_wa))	29	10
5203	((((u_csn(cp_ent ca_ent))((ca_wa p_re)ca_wa))(cp_ent oa_ent))(u_isn u_hdip))	35	10
5204	((((u_csn cp_ent)((ca_ent ca_wa)p_csn))(oa_ent(u_isn u_hdip)))(u_isn u_hdip))	28	10
5205	((((u_csn cp_ent)((ca_ent ca_wa)p_csn))((oa_ent u_isn)u_hdip))(u_isn u_hdip))	29	10
5206	((((u_csn cp_ent)((ca_ent ca_wa)p_re))(p_isn ca_wa))((cp_ent oa_ent)u_hdip))	26	10
5207	((((u_csn cp_ent)((ca_ent ca_wa)p_re))(p_isn ca_wa))((p_csn oa_ent)u_hdip))	22	10
5208	((((u_csn cp_ent)((ca_wa p_re)ca_wa))(cp_ent oa_ent))((u_isn oa_wa)u_hdip))	27	10
5209	((((u_hdip u_hdip)(oa_wa(u_csn cp_ent)))(ca_ent ca_wa))((p_re ca_wa)oa_ent))	19	10
5210	((((u_hdip u_hdip)(oa_wa(u_csn cp_ent)))ca_ent)(((ca_wa p_re)ca_wa)oa_ent))	21	10
5211	((((u_hdip u_hdip)(oa_wa(u_csn cp_ent)))(p_csn ca_ent))((ca_wa p_re)ca_wa))	24	10
5212	((((u_hdip u_hdip)(oa_wa(u_csn cp_ent)))(p_csn ca_ent))((ca_wa p_re)cp_ent))	24	10
5213	((((u_isn u_hdip)((u_isn oa_wa)u_csn))(p_csn(ca_ent ca_wa)))(p_re ca_wa))	24	10
5214	((((u_isn oa_wa)((u_csn cp_ent)p_csn))(p_isn ca_ent))((ca_wa p_re)cp_ent))	20	10
5215	((((u_isn u_hdip)((u_isn oa_wa)u_csn))(cp_ent ca_ent))((ca_wa p_re)ca_wa))	27	10
5216	((((u_isn u_hdip)((u_isn oa_wa)u_csn))(cp_ent ca_ent))((ca_wa p_re)cp_ent))	26	10
5217	((((oa_ent(oa_wa u_hdip))((u_isn oa_wa)u_csn))oa_ent)((oa_wa u_hdip)oa_wa))	17	10
5218	((((oa_wa(u_csn cp_ent))p_csn)ca_ent)(((ca_wa p_re)ca_wa)(cp_ent oa_ent)))	24	10
5219	((((oa_wa(u_csn cp_ent))p_csn)ca_ent)(((ca_wa p_re)ca_wa)(oa_ent u_isn)))	21	10
5220	((((oa_wa(u_csn cp_ent))p_csn)ca_ent)(((ca_wa p_re)cp_ent)(oa_ent u_isn)))	24	10
5221	((((oa_wa(u_csn cp_ent))(p_isn ca_wa))(p_re ca_wa))((cp_ent oa_ent)u_hdip))	24	10
5222	((((p_csn(ca_ent ca_wa))(p_re(ca_wa p_csn)))(cp_ent oa_ent))(u_isn u_hdip))	25	10
5223	((((p_csn(ca_ent ca_wa))((p_re ca_wa)p_csn))(cp_ent oa_ent))(u_hdip u_hdip))	26	10
5224	((((p_csn(ca_ent ca_wa))((p_re ca_wa)p_csn))(cp_ent oa_ent))(u_isn u_hdip))	27	10
5225	((((p_csn(ca_ent ca_wa))((p_re ca_wa)p_csn))(oa_ent oa_wa))(u_hdip u_hdip))	24	10
5226	((((p_csn(ca_ent ca_wa))((p_re ca_wa)p_csn))(oa_ent oa_wa))(u_isn u_hdip))	25	10
5227	((((p_csn(ca_ent ca_wa))((p_re ca_wa)p_csn))(oa_ent u_hdip))(u_isn u_hdip))	29	10
5228	((((p_csn(ca_ent ca_wa))((p_re ca_wa)p_csn))(oa_ent u_isn))(u_hdip u_hdip))	28	10
5229	((((p_csn(ca_ent ca_wa))((p_re ca_wa)p_csn))(p_isn oa_ent))(u_isn u_hdip))	25	10
5230	(((((p_csn(ca_wa p_re))(ca_wa p_csn))oa_ent)(u_isn u_hdip))(u_isn u_hdip))	22	10
5231	((((p_csn(ca_wa p_re))(ca_wa p_csn))((p_isn oa_ent)u_hdip))(u_isn u_hdip))	18	10
5232	((((p_csn(oa_ent u_hdip))(u_isn oa_wa))(u_csn cp_ent))(p_csn(ca_ent ca_wa)))	25	10
5233	((((p_csn(oa_ent u_hdip))(u_isn oa_wa))((u_csn cp_ent)p_csn))(ca_ent p_isn))	26	10
5234	((((p_csn(oa_ent u_hdip))(u_isn oa_wa))((u_csn cp_ent)p_csn))(ca_ent ca_wa))	26	10
5235	((((p_csn(oa_ent u_hdip))(u_isn oa_wa))((u_csn cp_ent)p_csn))(ca_wa ca_wa))	19	10
5236	((((p_csn(oa_ent u_hdip))(u_isn oa_wa))((u_csn cp_ent)p_csn))(p_isn ca_wa))	16	10
5237	((((p_csn(oa_ent u_isn))(u_hdip u_hdip))(u_isn oa_wa))((u_csn cp_ent)p_csn))	29	10
5238	(((((u_csn cp_ent)(ca_ent ca_wa))(p_isn ca_wa))(cp_ent oa_ent))(oa_wa oa_wa))	26	10
5239	(((((u_csn cp_ent)(ca_ent ca_wa))(p_isn ca_wa))(cp_ent oa_ent))(u_hdip oa_wa))	28	10
5240	(((((u_csn cp_ent)(ca_ent ca_wa))(p_isn ca_wa))(cp_ent oa_ent))(u_isn oa_wa))	28	10
5241	(((((u_csn cp_ent)(ca_ent ca_wa))(p_isn ca_wa))(cp_ent oa_ent))(u_isn u_hdip))	34	10
5242	((((u_csn(ca_ent ca_wa))(p_isn ca_wa))(oa_ent u_isn))(u_hdip(u_isn oa_wa)))	28	10
5243	((((u_csn(ca_ent ca_wa))(p_isn ca_wa))(p_csn(oa_ent u_hdip)))(u_isn u_hdip))	25	10
5244	((((u_csn(ca_ent ca_wa))(p_isn ca_wa))((cp_ent oa_ent)u_hdip))(u_isn oa_wa))	27	10
5245	((((u_csn(ca_ent ca_wa))(p_isn ca_wa))((cp_ent oa_ent)u_hdip))(u_isn u_hdip))	33	10
5246	((((u_csn(ca_ent ca_wa))(p_isn ca_wa))((oa_ent u_isn)u_hdip))(u_isn oa_wa))	27	10
5247	((((u_csn(ca_ent ca_wa))(p_isn ca_wa))((oa_ent u_isn)u_hdip))(u_isn u_hdip))	33	10
5248	(((((u_csn cp_ent)(ca_ent ca_wa))(p_re p_re))(p_csn oa_ent))(u_isn u_hdip))	23	10
5249	((((u_csn(ca_ent ca_wa))(p_re p_re))((p_csn oa_ent)u_hdip))(u_isn u_hdip))	23	10
5250	((((u_csn(cp_ent ca_wa))(p_isn ca_wa))((cp_ent oa_ent)u_hdip))(oa_wa u_hdip))	23	10
5251	((((u_csn(cp_ent ca_wa))(p_isn ca_wa))((cp_ent oa_ent)u_hdip))(u_isn oa_wa))	27	10
5252	((((u_csn(cp_ent ca_wa))(p_re ca_wa))((cp_ent oa_ent)u_hdip))(u_isn u_hdip))	27	10
5253	((((u_csn(cp_ent ca_wa))(p_isn ca_wa))((cp_ent oa_ent)oa_wa))(u_isn oa_wa))	26	10
5254	((((u_csn(cp_ent ca_wa))(p_isn ca_wa))((cp_ent oa_ent)oa_wa))(u_isn u_hdip))	27	10
5255	((((u_csn(cp_ent ca_wa))(p_isn ca_wa))((oa_ent u_isn)u_hdip))(u_isn oa_wa))	27	10
5256	((((u_csn(cp_ent ca_wa))(p_isn ca_wa))((oa_ent u_isn)u_hdip))(u_isn u_hdip))	27	10
5257	((((u_csn(cp_ent ca_wa))(p_re ca_wa))((cp_ent oa_ent)u_hdip))(u_isn oa_wa))	28	10
5258	((((u_csn(p_isn ca_ent))(ca_wa p_re))(p_isn ca_wa))((cp_ent oa_ent)u_hdip))	23	10
5259	((((u_csn(p_isn ca_ent))(ca_wa p_re))(p_isn ca_wa))((oa_ent u_isn)u_hdip))	23	10
5260	((((u_csn(p_isn ca_ent))(p_isn ca_wa))((cp_ent oa_ent)u_hdip))(u_isn u_hdip))	22	10
5261	((((u_csn(u_isn cp_ent))ca_ent)((ca_wa p_re)ca_wa))((cp_ent oa_ent)u_hdip))	25	10
5262	((((u_csn(u_isn cp_ent))(p_isn ca_wa))(p_re ca_wa))((cp_ent oa_ent)oa_wa))	18	10
5263	((((u_csn(u_isn cp_ent))(p_isn ca_wa))(p_re ca_wa))((cp_ent oa_ent)u_hdip))	18	10
5264	((((u_hdip(u_csn cp_ent))ca_ent)(p_isn ca_wa))((cp_ent oa_ent)(oa_wa u_hdip)))	22	10
5265	((((u_hdip(u_csn cp_ent))(p_isn ca_wa))(p_isn ca_wa))((cp_ent oa_ent)u_hdip))	19	10
5266	((((u_hdip(u_csn cp_ent))(p_isn ca_wa))(p_isn ca_wa))(oa_ent(oa_wa u_hdip)))	19	10
5267	((((u_hdip(u_csn cp_ent))(p_isn ca_wa))(p_isn ca_wa))((oa_ent u_isn)u_hdip))	19	10
5268	(((((u_hdip(u_isn oa_wa))u_csn)(ca_ent ca_wa))(p_isn p_re))(ca_wa p_csn))	23	10
5269	((((u_hdip(u_isn oa_wa))u_csn)(ca_ent ca_wa))((p_re ca_wa)(cp_ent oa_ent)))	26	10
5270	((((u_hdip(u_isn oa_wa))u_csn)(cp_ent ca_ent))(((ca_wa p_re)ca_wa)oa_ent))	22	10
5271	((((u_hdip(u_isn oa_wa))u_csn)(cp_ent ca_ent))(((ca_wa p_re)cp_ent)oa_ent))	25	10
5272	((((u_hdip(u_isn oa_wa))u_csn)(ca_ent ca_wa))(((p_re ca_wa)p_csn)oa_ent))	21	10
5273	((((u_hdip(u_isn oa_wa))u_csn)(cp_ent ca_wa))((p_re ca_wa)(cp_ent oa_ent)))	21	10
5274	((((u_hdip(u_isn oa_wa))u_csn)((cp_ent ca_ent)((ca_wa p_re)cp_ent)))oa_ent)	24	10
5275	((((u_hdip(u_isn oa_wa))(u_csn(cp_ent oa_ent)))(oa_wa u_hdip))(u_isn u_hdip))	26	10
5276	(((((ca_ent ca_wa)cp_ent)((oa_ent oa_wa)u_hdip))(u_isn u_hdip))(u_isn u_hdip))	29	10
5277	(((((ca_ent ca_wa)cp_ent)(oa_ent(u_isn u_hdip)))(u_isn oa_wa))(u_isn u_hdip))	31	10
5278	(((((ca_ent ca_wa)cp_ent)(oa_ent(u_isn u_hdip)))(u_isn u_hdip))(u_isn oa_wa))	32	10
5279	(((((ca_ent ca_wa)cp_ent)((oa_ent u_isn)u_hdip))(u_isn oa_wa))(u_isn oa_wa))	32	10
5280	(((((ca_ent ca_wa)cp_ent)((oa_ent u_isn)u_hdip))(u_isn oa_wa))(u_isn u_hdip))	33	10
5281	(((((ca_ent ca_wa)cp_ent)((oa_ent u_isn)u_hdip))(u_isn u_hdip))(u_isn oa_wa))	35	10
5282	(((((ca_ent ca_wa)cp_ent)((oa_ent u_isn)u_hdip))(u_isn u_hdip))(u_isn u_hdip))	36	10
5283	(((((ca_ent ca_wa)cp_ent)((p_isn oa_ent)u_hdip))(u_isn u_hdip))(u_isn u_hdip))	24	10
5284	((ca_ent p_isn)(((((p_re ca_wa)p_csn)oa_ent)(u_isn u_hdip))(u_isn oa_wa)))	33	10
5285	((ca_ent p_isn)(((((p_re ca_wa)p_csn)oa_ent)(u_isn u_hdip))(u_isn u_hdip)))	37	10
5286	(((((cp_ent ca_ent)(ca_wa p_re))(cp_ent oa_ent))(u_isn u_hdip))(u_isn u_hdip))	28	10
5287	(((((ca_ent ca_wa)p_re)(p_csn(oa_ent u_isn)))(u_hdip u_hdip))(u_isn u_hdip))	28	10

5288	((ca_ent p_isn))))(((p_re ca_wa) p_csn) oa_ent)(u_hdip u_hdip))(u_isn u_hdip)))	32	10
5289	(((((ca_ent p_re)(cp_ent oa_ent)))(oa_wa u_hdip))(u_isn u_hdip))(u_isn oa_wa))	23	10
5290	((((ca_ent p_re)(cp_ent oa_ent)))(oa_wa u_hdip))(u_isn u_hdip))(u_isn u_hdip))	20	10
5291	(((((ca_ent p_re)(cp_ent oa_ent))(u_hdip u_hdip))(u_isn oa_wa))(u_csn cp_ent))	22	10
5292	((((ca_ent p_re)(cp_ent oa_ent))(u_hdip u_hdip))(u_isn u_hdip))(u_isn oa_wa))	26	10
5293	(((((ca_ent p_re)(cp_ent oa_ent))(u_hdip u_hdip))(u_isn u_hdip))(u_isn u_hdip))	21	10
5294	(((((ca_wa ca_wa)(cp_ent oa_ent))(u_hdip u_hdip))(u_isn u_hdip))(oa_wa u_csn))	28	10
5295	(((((ca_wa ca_wa)(cp_ent oa_ent))(u_hdip u_hdip))(u_isn u_hdip))(u_isn oa_wa))	27	10
5296	(((((ca_wa ca_wa)(cp_ent oa_ent))(u_hdip u_hdip))(u_isn u_hdip))(u_isn u_hdip))	25	10
5297	((((ca_wa ca_wa)(cp_ent oa_ent))(u_isn u_hdip))(u_isn u_hdip))(u_isn oa_wa))	27	10
5298	((((ca_wa ca_wa)(p_csn oa_ent))(u_hdip u_hdip))(u_isn u_hdip))(u_isn u_hdip))	24	10
5299	(((((ca_wa p_re) ca_wa)(cp_ent oa_ent))(u_isn u_hdip)))((u_isn oa_wa) u_hdip))	35	10
5300	(((((ca_wa p_re) ca_wa)((cp_ent oa_ent) oa_wa))(u_hdip u_hdip))(u_isn u_hdip))	30	10
5301	(((((ca_wa p_re) ca_wa)((cp_ent oa_ent) oa_wa))(u_isn u_hdip))(u_isn u_hdip))	32	10
5302	((((ca_wa p_re) cp_ent) oa_ent)((oa_wa u_hdip)(u_isn oa_wa)))(u_csn cp_ent))	25	10
5303	(((((ca_wa p_re) cp_ent)(oa_ent oa_wa))(u_isn oa_wa))(u_hdip (u_isn u_csn)))	19	10
5304	(((((ca_wa p_re) cp_ent)((oa_ent u_isn) u_hdip))(u_isn u_hdip))(u_isn oa_wa))	33	10
5305	(((((ca_wa p_re) cp_ent)(oa_ent (oa_wa u_hdip)))(u_isn u_hdip))(u_isn oa_wa))	27	10
5306	(((((ca_wa p_re) cp_ent)(oa_ent (oa_wa u_hdip)))(u_isn u_hdip))(u_isn u_hdip))	28	10
5307	(((((ca_wa p_re) cp_ent)(oa_ent (u_isn u_hdip)))(u_isn oa_wa))(u_isn u_hdip))	32	10
5308	(((((ca_wa p_re) cp_ent)((oa_ent u_isn) u_hdip))(u_isn oa_wa))(u_isn oa_wa))	31	10
5309	(((((ca_wa p_re) cp_ent)((oa_ent u_isn) u_hdip))(u_isn oa_wa))(u_isn u_hdip))	34	10
5310	(((((ca_wa p_re) cp_ent)((oa_ent u_isn) u_hdip))(u_isn u_hdip))(u_isn u_hdip))	34	10
5311	(((((cp_ent ca_ent) cp_ent) oa_ent)((oa_wa u_hdip)(u_isn oa_wa)))(u_csn cp_ent))	25	10
5312	(((((cp_ent ca_ent) cp_ent)((oa_ent oa_wa) u_hdip))(u_isn u_hdip))(u_isn u_hdip))	24	10
5313	(((((cp_ent ca_ent) cp_ent)(oa_ent (oa_wa u_hdip)))(u_isn u_hdip))(u_isn oa_wa))	29	10
5314	(((((cp_ent ca_ent) cp_ent)(oa_ent (oa_wa u_hdip)))(u_isn u_hdip))(u_isn u_hdip))	26	10
5315	(((((cp_ent ca_ent) cp_ent)(oa_ent (u_isn u_hdip)))(u_isn oa_wa))(u_isn oa_wa))	30	10
5316	(((((cp_ent ca_ent) cp_ent)(oa_ent (u_isn u_hdip)))(u_isn oa_wa))(u_isn u_hdip))	29	10
5317	(((((cp_ent ca_ent) cp_ent)(oa_ent (u_isn u_hdip)))(u_isn u_hdip))(u_isn u_hdip))	32	10
5318	(((((cp_ent ca_ent) cp_ent)((oa_ent u_isn) u_hdip))(u_isn oa_wa))(u_isn oa_wa))	32	10
5319	(((((cp_ent ca_ent) cp_ent)((oa_ent u_isn) u_hdip))(u_isn oa_wa))(u_isn u_hdip))	31	10
5320	(((((cp_ent ca_ent) cp_ent)((oa_ent u_isn) u_hdip))(u_isn u_hdip))(u_isn oa_wa))	32	10
5321	(((((cp_ent ca_ent) cp_ent)((oa_ent u_isn) u_hdip))(u_isn u_hdip))(u_isn u_hdip))	34	10
5322	(((((cp_ent ca_ent)(ca_wa p_re))(cp_ent oa_ent))(oa_wa u_hdip))(u_isn u_hdip))	29	10
5323	(((((cp_ent ca_ent)(ca_wa p_re))(cp_ent oa_ent))(u_hdip u_hdip))(u_isn u_hdip))	33	10
5324	(((((cp_ent ca_ent)(ca_wa p_re))(p_csn oa_ent))(u_isn u_hdip))(u_isn u_hdip))	31	10
5325	(((((cp_ent ca_wa) cp_ent) oa_ent)((oa_wa u_hdip)(u_isn oa_wa)))(u_csn cp_ent))	25	10
5326	(((((cp_ent ca_wa) cp_ent)(oa_ent oa_wa))(u_isn oa_wa))(u_hdip (u_isn u_csn)))	17	10
5327	(((((cp_ent ca_wa) cp_ent)((oa_ent u_isn) u_hdip))(u_isn oa_wa))(u_csn cp_ent))	27	10
5328	(((((cp_ent ca_wa) cp_ent)(oa_ent (oa_wa u_hdip)))(u_isn u_hdip))(u_isn oa_wa))	28	10
5329	(((((cp_ent ca_wa) cp_ent)(oa_ent (oa_wa u_hdip)))(u_isn u_hdip))(u_isn u_hdip))	22	10
5330	(((((cp_ent ca_wa) cp_ent)(oa_ent (u_isn u_hdip)))(u_isn oa_wa))(u_isn oa_wa))	29	10
5331	(((((cp_ent ca_wa) cp_ent)(oa_ent (u_isn u_hdip)))(u_isn oa_wa))(u_isn u_hdip))	29	10
5332	(((((cp_ent ca_wa) cp_ent)((oa_ent u_isn) u_hdip))(u_isn oa_wa))(u_isn oa_wa))	31	10
5333	(((((cp_ent ca_wa) cp_ent)((oa_ent u_isn) u_hdip))(u_isn oa_wa))(u_isn u_hdip))	31	10
5334	(((((cp_ent ca_wa) cp_ent)((oa_ent u_isn) u_hdip))(u_isn u_hdip))(u_isn u_hdip))	28	10
5335	(((((cp_ent oa_ent)(oa_wa u_hdip))((u_isn oa_wa) u_csn)) oa_ent)(oa_wa u_hdip))	19	10
5336	(((((oa_ent oa_wa) u_csn)(u_isn cp_ent))(ca_ent ca_wa)))((p_re ca_wa) p_csn))	20	10
5337	(((((oa_ent oa_wa) u_csn)(u_isn cp_ent)))((ca_ent ca_wa) p_re))(ca_wa p_csn))	18	10
5338	(((((oa_ent oa_wa) u_hdip)((u_isn oa_wa) u_csn))(cp_ent oa_ent))(oa_wa u_hdip))	18	10
5339	(((((oa_ent oa_wa) u_hdip)((u_isn oa_wa) u_csn))(oa_ent oa_wa))(u_isn u_hdip))	15	10
5340	(((((oa_wa u_hdip) oa_wa)(u_csn (cp_ent ca_ent)))((ca_wa p_re) cp_ent)) oa_ent)	23	10
5341	(((((p_csn ca_ent)(ca_wa p_re))(ca_wa p_csn))(cp_ent oa_ent))(u_isn u_hdip))	26	10
5342	(((((p_csn oa_ent)(u_isn u_hdip))(u_isn u_hdip))(u_isn u_hdip))(u_isn u_hdip))	29	10
5343	(((((p_isn ca_ent)(ca_wa p_re))(cp_ent oa_ent))(u_hdip u_hdip))(u_isn u_hdip))	30	10
5344	(((((p_isn ca_ent)(ca_wa p_re))(cp_ent oa_ent))(u_isn u_hdip))(u_isn u_hdip))	25	10
5345	(((((p_isn ca_ent)(ca_wa p_re))(p_csn oa_ent))(u_isn u_hdip))(u_isn u_hdip))	25	10
5346	(((((p_re ca_wa)(cp_ent oa_ent))(oa_wa u_hdip))(u_isn oa_wa))(u_csn cp_ent))	24	10
5347	(((((p_re ca_wa)(cp_ent oa_ent))(oa_wa u_hdip))(u_isn u_hdip))(u_isn u_hdip))	30	10
5348	(((((p_re ca_wa)(cp_ent oa_ent))(u_hdip u_hdip))(u_isn u_hdip))(oa_wa u_csn))	32	10
5349	(((((p_re ca_wa)(cp_ent oa_ent))(u_hdip u_hdip))(u_isn u_hdip))(u_isn oa_wa))	32	10
5350	(((((p_re ca_wa)(cp_ent oa_ent))(u_hdip u_hdip))(u_isn u_hdip))(u_isn u_hdip))	32	10
5351	(((((p_re ca_wa)(cp_ent oa_ent))(u_isn u_hdip))(u_isn u_hdip))(u_isn oa_wa))	32	10
5352	(((((p_re ca_wa)(p_csn oa_ent))(u_hdip u_hdip))(u_isn u_hdip))(u_isn u_hdip))	25	10
5353	(((((p_re ca_wa)(p_csn oa_ent))(u_isn u_hdip))(u_isn oa_wa))(u_isn u_hdip))	29	10
5354	(((((u_csn cp_ent)(p_isn ca_ent))(ca_wa p_re))(p_isn ca_wa))(cp_ent oa_ent))	24	10
5355	(((((u_csn cp_ent)(p_isn ca_wa))(p_re ca_wa))(cp_ent oa_ent))(oa_wa oa_wa))	27	10
5356	(((((u_csn cp_ent)(p_isn ca_wa))(p_re ca_wa))(cp_ent oa_ent))(oa_wa u_hdip))	28	10
5357	(((((u_csn cp_ent)(p_isn ca_wa))(p_re ca_wa))(cp_ent oa_ent))(u_hdip oa_wa))	27	10
5358	(((((u_csn cp_ent)(p_isn ca_wa))(p_re ca_wa))(cp_ent oa_ent))(u_hdip u_hdip))	27	10
5359	(((((u_csn cp_ent)(p_isn ca_wa))(p_re ca_wa))(cp_ent oa_ent))(u_isn oa_wa))	28	10
5360	(((((u_hdip oa_wa) u_csn)(cp_ent ca_ent))(ca_wa ca_wa))((cp_ent oa_ent) u_hdip))	26	10
5361	(((((u_hdip oa_wa) u_csn)(cp_ent ca_ent))(p_isn ca_wa))((cp_ent oa_ent) u_hdip))	22	10
5362	(((((u_hdip oa_wa) u_csn)((cp_ent ca_wa) cp_ent))(oa_ent oa_wa))(u_hdip u_hdip))	19	10
5363	(((((u_hdip oa_wa) u_csn)((cp_ent ca_wa) cp_ent))(oa_ent oa_wa))(u_isn u_hdip))	19	10
5364	(((((u_isn cp_ent) ca_ent)((ca_wa p_re) ca_wa))(p_csn cp_ent))(oa_ent u_isn))	18	10
5365	(((((u_isn cp_ent)(p_isn ca_ent)))((ca_wa p_re) ca_wa))(p_csn cp_ent)) oa_ent)	15	10
5366	(((((u_isn oa_wa) u_hdip)(oa_wa u_csn))(cp_ent ca_ent))((ca_wa p_re) ca_wa))	25	10
5367	(((((u_isn oa_wa) u_hdip)(oa_wa u_csn))(cp_ent ca_ent))((ca_wa p_re) cp_ent))	24	10
5368	(((((u_isn oa_wa) u_hdip)(oa_wa u_csn))(oa_ent (u_isn u_hdip)))(u_isn u_hdip))	19	10
5369	(p_csn ((ca_ent ca_wa)(((p_re ca_wa)((cp_ent oa_ent) u_hdip))(u_isn u_hdip))))	27	10
5370	(p_csn ((ca_ent p_isn)(((p_re ca_wa)((cp_ent oa_ent) u_hdip))(u_isn u_hdip))))	26	10
5371	(p_csn ((p_isn ca_ent)((((ca_wa p_re) ca_wa)(cp_ent oa_ent))(u_hdip u_hdip))))	25	10
5372	(p_csn ((p_isn ca_ent)((((ca_wa p_re) ca_wa)(cp_ent oa_ent))(u_isn u_hdip))))	24	10
5373	(p_csn ((p_isn ca_ent)((((ca_wa p_re) ca_wa)(oa_ent u_isn))(u_hdip u_hdip))))	21	10
5374	((p_csn ((p_isn ca_ent)(((ca_wa p_re) cp_ent)(oa_ent u_hdip))))(u_isn u_hdip))	24	10
5375	(p_csn (((p_isn ca_ent)(((ca_wa p_re) ca_wa)(cp_ent oa_ent)))(u_hdip u_hdip)))	26	10
5376	(p_csn (((p_isn ca_ent)(((ca_wa p_re) ca_wa)(cp_ent oa_ent)))(u_isn u_hdip)))	25	10
5377	(p_csn (((p_isn ca_ent)(((ca_wa p_re) cp_ent)(oa_ent u_isn)))(u_hdip u_hdip)))	25	10
5378	(p_csn ((((ca_ent ca_wa) p_re)(cp_ent oa_ent))(u_isn u_hdip))(u_isn u_hdip)))	20	10
5379	((ca_ent ca_wa)((p_isn p_re)(((ca_wa (p_csn cp_ent)) oa_ent)(u_hdip u_hdip))))	32	10
5380	((ca_ent ca_wa)((p_re ca_wa)((p_csn (oa_ent (u_isn u_hdip)))(u_isn oa_wa))))	27	10
5381	((ca_ent ca_wa)((p_re ca_wa)((p_csn (oa_ent (u_isn u_hdip)))(u_isn u_hdip))))	33	10
5382	((ca_ent ca_wa)((p_re ca_wa)((p_csn ((oa_ent u_isn) u_hdip))(u_isn oa_wa))))	26	10
5383	((ca_ent ca_wa)((p_re ca_wa)(((p_csn (oa_ent u_isn)) u_hdip)(oa_wa u_hdip))))	29	10
5384	((ca_ent ca_wa)((p_re ca_wa)(((p_csn (oa_ent u_isn)) u_hdip)(u_isn oa_wa))))	27	10
5385	((ca_ent ca_wa)(((p_re ca_wa)(p_csn (oa_ent (oa_wa u_hdip))))(u_isn u_hdip)))	27	10
5386	((ca_ent ca_wa)(((p_re ca_wa)(p_csn ((oa_ent oa_wa) u_hdip)))(u_isn u_hdip)))	27	10
5387	((ca_ent ca_wa)(((p_re ca_wa)(p_csn ((p_isn oa_ent) u_hdip)))(u_isn u_hdip)))	24	10
5388	((ca_ent ca_wa)((((p_re ca_wa) oa_ent)(oa_wa u_hdip))((u_isn oa_wa) u_csn)))	23	10
5389	((ca_ent ca_wa)(((((p_re ca_wa) p_csn) oa_ent)(oa_wa u_hdip))(u_isn u_hdip)))	27	10
5390	((ca_ent ca_wa)(((((p_re ca_wa) p_csn) oa_ent)(u_hdip u_hdip))(u_isn u_hdip)))	34	10
5391	((ca_ent ca_wa)(((((p_re ca_wa) p_csn) oa_ent)(u_isn u_hdip))(oa_wa u_hdip)))	33	10
5392	((ca_ent ca_wa)(((((p_re ca_wa) p_csn) oa_ent)(u_isn u_hdip))(u_isn oa_wa)))	35	10
5393	((ca_ent ca_wa)(((((p_re ca_wa) p_csn) oa_ent)(u_isn u_hdip))(u_isn u_hdip)))	38	10
5394	((ca_ent p_isn)((p_re ca_wa)((p_csn (oa_ent (u_isn u_hdip)))(u_isn oa_wa))))	23	10
5395	((ca_ent p_isn)((p_re ca_wa)((p_csn (oa_ent (u_isn u_hdip)))(u_isn u_hdip))))	32	10
5396	((ca_ent p_isn)((p_re ca_wa)((p_csn ((oa_ent u_isn) u_hdip))(u_isn oa_wa))))	22	10
5397	((ca_ent p_isn)((p_re ca_wa)(((p_csn (oa_ent u_isn)) u_hdip)(u_isn oa_wa))))	23	10
5398	((ca_ent p_isn)((((ca_wa (p_csn cp_ent)) oa_ent)(u_isn u_hdip))(u_isn oa_wa)))	20	10
5399	((ca_ent p_isn)(((p_re ca_wa)(p_csn (oa_ent (oa_wa u_hdip))))(u_isn u_hdip)))	23	10
5400	((ca_ent p_isn)(((p_re ca_wa)(p_csn ((oa_ent oa_wa) u_hdip)))(u_isn u_hdip)))	25	10
5401	((ca_ent p_isn)(((p_re ca_wa)(p_csn ((p_isn oa_ent) u_hdip)))(u_isn u_hdip)))	22	10
5402	((ca_ent p_isn)(((((p_re ca_wa) p_csn) oa_ent)(oa_wa u_hdip))(u_isn u_hdip)))	25	10
5403	((ca_ent p_isn)(((((p_re ca_wa) p_csn) oa_ent)(u_isn u_hdip))(oa_wa u_hdip)))	29	10
5404	((cp_ent ca_ent)((ca_wa ca_wa)((p_csn (oa_ent (u_isn u_hdip)))(u_isn u_hdip))))	27	10
5405	((cp_ent ca_ent)((p_re ca_wa)((p_csn ((oa_ent u_isn) u_hdip))(u_isn oa_wa))))	22	10
5406	((cp_ent ca_ent)((p_re ca_wa)(((p_csn (oa_ent u_isn)) u_hdip)(u_isn oa_wa))))	23	10
5407	((cp_ent ca_ent)(((((ca_wa p_re) cp_ent) oa_ent)(u_isn u_hdip))(u_isn oa_wa)))	34	10
5408	((cp_ent ca_ent)(((((ca_wa p_re) cp_ent) oa_ent)(u_isn u_hdip))(u_isn u_hdip)))	39	10
5409	((cp_ent ca_ent)(((((p_re ca_wa) p_csn) oa_ent)(oa_wa u_hdip))(u_isn u_hdip)))	23	10
5410	((cp_ent ca_ent)(((((p_re ca_wa) p_csn) oa_ent)(u_hdip u_hdip))(u_isn u_hdip)))	28	10
5411	((cp_ent ca_ent)(((((p_re ca_wa) p_csn) oa_ent)(u_isn u_hdip))(oa_wa u_hdip)))	27	10
5412	((cp_ent ca_ent)(((((p_re ca_wa) p_csn) oa_ent)(u_isn u_hdip))(u_isn oa_wa)))	29	10
5413	((cp_ent ca_ent)(((((p_re ca_wa) p_csn) oa_ent)(u_isn u_hdip))(u_isn u_hdip)))	35	10
5414	((cp_ent ca_wa)((p_re ca_wa)((p_csn (oa_ent (u_isn u_hdip)))(u_isn u_hdip))))	29	10
5415	((cp_ent ca_wa)(((((p_re ca_wa) p_csn) oa_ent)(u_isn u_hdip))(u_isn oa_wa)))	30	10
5416	((cp_ent ca_wa)(((((p_re ca_wa) p_csn) oa_ent)(u_isn u_hdip))(u_isn u_hdip)))	33	10
5417	((oa_wa u_hdip)((((u_isn oa_wa)((u_csn cp_ent) p_csn)) ca_ent)(ca_wa ca_wa)))	17	10
5418	((oa_wa u_hdip)((((u_isn oa_wa)((u_csn cp_ent) p_csn)) ca_ent)(ca_wa p_re)))	17	10
5419	((p_csn ca_ent)(((((ca_wa p_re) ca_wa) oa_ent)(u_isn u_hdip))(u_isn u_hdip)))	25	10
5420	((p_csn ca_ent)(((((ca_wa p_re) cp_ent) oa_ent)(u_isn u_hdip))(u_isn u_hdip)))	30	10
5421	((p_isn ca_ent)((ca_wa ca_wa)((p_csn (oa_ent (u_isn u_hdip)))(u_isn u_hdip))))	25	10
5422	((p_isn ca_ent)((ca_wa p_re)((p_csn (oa_ent (u_isn u_hdip)))(u_isn u_hdip))))	23	10
5423	((p_isn ca_ent)((p_re ca_wa)((p_csn ((oa_ent u_isn) u_hdip))(u_isn oa_wa))))	18	10
5424	((p_isn ca_ent)((p_re ca_wa)(((p_csn (oa_ent u_isn)) u_hdip)(u_isn oa_wa))))	19	10
5425	((p_isn ca_ent)((ca_wa p_re)(((p_csn (oa_ent u_isn)) u_hdip)(u_isn u_hdip))))	26	10
5426	((p_isn ca_ent)((ca_wa p_re)(((p_csn (p_isn oa_ent)) u_hdip)(u_isn u_hdip))))	21	10
5427	((p_isn ca_ent)(((((ca_wa p_re) ca_wa) oa_ent)(oa_wa u_hdip))(u_isn u_hdip)))	25	10
5428	((p_isn ca_ent)(((((ca_wa p_re) ca_wa) oa_ent)(u_hdip u_hdip))(u_isn u_hdip)))	28	10
5429	((p_isn ca_ent)(((((ca_wa p_re) ca_wa) oa_ent)(u_isn u_hdip))(u_isn u_hdip)))	29	10
5430	((p_isn ca_ent)(((((ca_wa p_re) cp_ent) oa_ent)(oa_wa oa_wa))(u_csn cp_ent)))	24	10
5431	((p_isn ca_ent)(((((ca_wa p_re) cp_ent) oa_ent)(oa_wa u_hdip))(u_isn u_hdip)))	27	10
5432	((p_isn ca_ent)(((((ca_wa p_re) cp_ent) oa_ent)(u_isn u_hdip))(u_isn oa_wa)))	31	10
5433	((p_isn ca_ent)(((((ca_wa p_re) cp_ent) oa_ent)(u_isn u_hdip))(u_isn u_hdip)))	35	10
5434	((p_isn ca_ent)(((((p_re ca_wa) p_csn) oa_ent)(oa_wa u_hdip))(u_isn u_hdip)))	19	10
5435	((p_isn ca_ent)(((((p_re ca_wa) p_csn) oa_ent)(u_hdip u_hdip))(u_isn u_hdip)))	24	10
5436	((p_isn ca_ent)(((((p_re ca_wa) p_csn) oa_ent)(u_isn u_hdip))(oa_wa u_hdip)))	26	10
5437	((p_isn ca_ent)(((((p_re ca_wa) p_csn) oa_ent)(u_isn u_hdip))(u_isn oa_wa)))	27	10
5438	((p_isn ca_ent)(((((p_re ca_wa) p_csn) oa_ent)(u_isn u_hdip))(u_isn u_hdip)))	31	10
5439	((p_isn ca_wa)((p_re ca_wa)((p_csn (oa_ent (u_isn u_hdip)))(u_isn oa_wa))))	27	10
5440	((p_isn ca_wa)((p_re ca_wa)((p_csn (oa_ent (u_isn u_hdip)))(u_isn u_hdip))))	31	10
5441	((p_isn ca_wa)((p_re ca_wa)((p_csn ((oa_ent u_isn) u_hdip))(u_isn oa_wa))))	26	10
5442	((p_isn ca_wa)((p_re ca_wa)(((p_csn (oa_ent u_isn)) u_hdip)(u_isn oa_wa))))	27	10
5443	((p_isn ca_wa)(((p_re ca_wa)(p_csn (oa_ent (oa_wa u_hdip))))(u_isn u_hdip)))	27	10
5444	((p_isn ca_wa)(((p_re ca_wa)(p_csn ((oa_ent oa_wa) u_hdip)))(u_isn u_hdip)))	26	10
5445	((p_isn ca_wa)(((p_re ca_wa)(p_csn ((p_isn oa_ent) u_hdip)))(u_isn u_hdip)))	24	10
5446	((p_isn ca_wa)((((p_re ca_wa) oa_ent)(oa_wa u_hdip))((u_isn oa_wa) u_csn)))	21	10
5447	((p_isn ca_wa)(((((p_re ca_wa) p_csn) oa_ent)(oa_wa u_hdip))(u_isn u_hdip)))	26	10
5448	((p_isn ca_wa)(((((p_re ca_wa) p_csn) oa_ent)(u_hdip u_hdip))(u_isn u_hdip)))	32	10
5449	((p_isn ca_wa)(((((p_re ca_wa) p_csn) oa_ent)(u_isn u_hdip))(u_isn oa_wa)))	31	10
5450	((p_isn ca_wa)(((((p_re ca_wa) p_csn) oa_ent)(u_isn u_hdip))(u_isn u_hdip)))	34	10
5451	((u_hdip u_hdip)((((u_isn oa_wa)((u_csn cp_ent) p_csn)) ca_ent)(ca_wa ca_wa)))	25	10
5452	((u_hdip u_hdip)((((u_isn oa_wa)((u_csn cp_ent) p_csn)) ca_ent)(ca_wa p_re)))	25	10
5453	((u_isn u_hdip)(((u_isn u_hdip)((oa_wa (u_csn cp_ent)) oa_ent))(u_isn oa_wa)))	22	10
5454	((u_isn u_hdip)(((u_isn u_hdip)((oa_wa (u_csn cp_ent)) oa_ent))(u_isn u_hdip)))	19	10
5455	((u_isn u_hdip)(((u_isn u_hdip)(((oa_wa u_csn) oa_ent) oa_wa))(u_isn u_hdip)))	16	10
5456	((u_isn u_hdip)((((u_isn oa_wa)((u_csn cp_ent) p_csn)) ca_ent)(ca_wa ca_wa)))	25	10
5457	((u_isn u_hdip)((((u_isn oa_wa)((u_csn cp_ent) p_csn)) ca_ent)(ca_wa p_re)))	25	10
5458	(((ca_ent ca_wa)((p_re ca_wa)(p_csn (oa_ent (oa_wa u_hdip)))))(u_isn u_hdip))	29	10
5459	(((ca_ent ca_wa)((p_re ca_wa)(p_csn ((oa_ent oa_wa) u_hdip))))(u_isn u_hdip))	28	10
5460	(((ca_ent ca_wa)((((p_re ca_wa) p_csn) oa_ent)(oa_wa u_hdip)))(u_isn u_hdip))	28	10
5461	(((ca_ent p_isn)((p_re ca_wa)(p_csn (oa_ent (oa_wa u_hdip)))))(u_isn u_hdip))	25	10
5462	(((ca_ent p_isn)(((ca_wa (p_csn cp_ent)) oa_ent)(u_isn u_hdip)))(u_isn u_hdip))	28	10
5463	(((ca_ent p_isn)((((p_re ca_wa) p_csn) oa_ent)(u_hdip u_hdip)))(u_isn u_hdip))	33	10
5464	((((p_re (ca_wa p_csn)) oa_ent)(u_hdip u_hdip))(u_isn u_hdip))(u_isn u_hdip))	21	10
5465	(((((p_isn ca_wa) p_csn) oa_ent)(u_hdip u_hdip))(u_isn u_hdip))(u_isn u_hdip))	21	10
5466	(((cp_ent ca_ent)((((ca_wa p_re) ca_wa) oa_ent)(u_hdip u_hdip)))(u_isn u_hdip))	31	10
5467	(((cp_ent ca_ent)((((ca_wa p_re) ca_wa) oa_ent)(u_isn u_hdip)))(u_isn oa_wa))	28	10
5468	(((cp_ent ca_ent)((((ca_wa p_re) ca_wa) oa_ent)(u_isn u_hdip)))(u_isn u_hdip))	32	10
5469	(((cp_ent ca_ent)((((ca_wa p_re) cp_ent) oa_ent)(oa_wa oa_wa)))(u_csn cp_ent))	28	10
5470	(((cp_ent ca_ent)((((ca_wa p_re) cp_ent) oa_ent)(oa_wa u_hdip)))(u_isn u_hdip))	31	10
5471	(((cp_ent ca_ent)((((ca_wa p_re) cp_ent) oa_ent)(u_hdip u_hdip)))(u_isn u_hdip))	35	10
5472	(((cp_ent ca_ent)((((ca_wa p_re) cp_ent) oa_ent)(u_isn oa_wa)))(u_csn cp_ent))	24	10
5473	(((cp_ent ca_ent)((((ca_wa p_re) cp_ent) oa_ent)(u_isn oa_wa)))(u_isn oa_wa))	31	10
5474	(((cp_ent ca_ent)((((ca_wa p_re) cp_ent) oa_ent)(u_isn oa_wa)))(u_isn u_hdip))	31	10
5475	(((cp_ent ca_ent)((((ca_wa p_re) cp_ent) oa_ent)(u_isn u_hdip)))(u_isn oa_wa))	36	10
5476	(((cp_ent ca_ent)((((p_re ca_wa) p_csn) oa_ent)(oa_wa u_hdip)))(u_isn u_hdip))	25	10
5477	(((cp_ent ca_ent)((((p_re ca_wa) p_csn) oa_ent)(u_hdip u_hdip)))(u_isn u_hdip))	31	10
5478	(((cp_ent ca_ent)((((p_re ca_wa) p_csn) oa_ent)(u_isn u_hdip)))(oa_wa u_hdip))	29	10
5479	(((oa_ent u_hdip)((u_isn u_hdip)((u_isn u_hdip)((u_isn oa_wa) u_csn))))) oa_ent)	26	10
5480	(((p_isn ca_ent)((ca_wa p_re)((p_csn (oa_ent u_isn)) u_hdip)))(oa_wa u_hdip))	22	10
5481	(((p_isn ca_ent)((((ca_wa p_re) cp_ent) oa_ent)(oa_wa oa_wa)))(u_csn cp_ent))	25	10
5482	(((p_isn ca_ent)((((ca_wa p_re) cp_ent) oa_ent)(oa_wa u_hdip)))(u_isn u_hdip))	26	10
5483	(((p_isn ca_ent)((((ca_wa p_re) cp_ent) oa_ent)(u_isn oa_wa)))(u_csn cp_ent))	23	10
5484	(((p_isn ca_ent)((((ca_wa p_re) cp_ent) oa_ent)(u_isn oa_wa)))(u_isn oa_wa))	28	10
5485	(((p_isn ca_ent)((((ca_wa p_re) cp_ent) oa_ent)(u_isn oa_wa)))(u_isn u_hdip))	29	10
5486	(((p_isn ca_ent)((((ca_wa p_re) cp_ent) oa_ent)(u_isn u_hdip)))(u_isn oa_wa))	28	10
5487	(((p_isn ca_ent)((((p_re ca_wa) p_csn) oa_ent)(oa_wa u_hdip)))(u_isn u_hdip))	21	10
5488	(((p_isn ca_wa)((((p_re ca_wa) p_csn) oa_ent)(oa_wa u_hdip)))(u_isn u_hdip))	27	10
5489	((((u_hdip u_hdip)((u_isn oa_wa)((u_csn cp_ent) p_csn))) ca_ent)(ca_wa ca_wa))	27	10
5490	(((u_hdip u_hdip)(((oa_wa (u_csn cp_ent)) p_csn) ca_ent))((ca_wa p_re) ca_wa))	27	10
5491	(((u_hdip u_hdip)(((oa_wa (u_csn cp_ent)) p_csn) ca_ent))((ca_wa p_re) cp_ent))	27	10
5492	(((u_hdip u_hdip)(((oa_wa (u_csn cp_ent)) p_csn)(ca_ent p_isn)))(p_re ca_wa))	27	10
5493	(((u_hdip u_hdip)(((oa_wa (u_csn cp_ent)) p_csn)(p_isn ca_ent)))(ca_wa ca_wa))	21	10
5494	(((u_isn u_hdip)((u_isn u_hdip)((oa_wa (u_csn cp_ent)) oa_ent)))(u_isn oa_wa))	21	10
5495	(((u_isn u_hdip)((u_isn u_hdip)((oa_wa (u_csn cp_ent)) oa_ent)))(u_isn u_hdip))	21	10
5496	(((u_isn u_hdip)((u_isn u_hdip)(((oa_wa u_csn) oa_ent) oa_wa)))(u_isn u_hdip))	17	10
5497	((((ca_ent ca_wa)(((p_re ca_wa) p_csn) oa_ent))(u_isn oa_wa))(u_isn oa_wa))	28	10
5498	((((ca_ent ca_wa)(((p_re ca_wa) p_csn) oa_ent))(u_isn oa_wa))(u_isn u_hdip))	29	10
5499	((((cp_ent ca_ent)((p_re (ca_wa p_csn)) oa_ent))(u_hdip u_hdip))(u_isn u_hdip))	26	10
5500	((((cp_ent ca_ent)((p_re (ca_wa p_csn)) oa_ent))(u_isn u_hdip))(u_isn u_hdip))	28	10
5501	((((cp_ent ca_ent)(((ca_wa p_re) ca_wa) oa_ent))(oa_wa u_hdip))(u_isn u_hdip))	29	10
5502	((((cp_ent ca_ent)(((ca_wa p_re) ca_wa) oa_ent))(u_hdip u_hdip))(u_isn u_hdip))	32	10
5503	((((cp_ent ca_ent)(((ca_wa p_re) ca_wa) oa_ent))(u_isn u_hdip))(u_isn oa_wa))	29	10
5504	((((cp_ent ca_ent)(((ca_wa p_re) ca_wa) oa_ent))(u_isn u_hdip))(u_isn u_hdip))	33	10
5505	((((cp_ent ca_ent)(((ca_wa p_re) cp_ent) oa_ent))(oa_wa oa_wa))(u_csn cp_ent))	29	10
5506	((((cp_ent ca_ent)(((ca_wa p_re) cp_ent) oa_ent))(u_isn oa_wa))(u_isn oa_wa))	31	10
5507	((((cp_ent ca_ent)(((ca_wa p_re) cp_ent) oa_ent))(u_isn oa_wa))(u_isn u_hdip))	31	10
5508	((((cp_ent ca_wa)(((p_re ca_wa) p_csn) oa_ent))(u_hdip u_hdip))(u_isn u_hdip))	28	10
5509	((((p_isn ca_ent)(((ca_wa p_re) ca_wa) oa_ent))(oa_wa u_hdip))(oa_wa u_csn))	21	10
5510	((((p_isn ca_ent)(((ca_wa p_re) ca_wa) oa_ent))(oa_wa u_hdip))(u_csn cp_ent))	21	10
5511	((((p_isn ca_ent)(((ca_wa p_re) ca_wa) oa_ent))(oa_wa u_hdip))(u_isn u_csn))	20	10
5512	((((p_isn ca_ent)(((ca_wa p_re) cp_ent) oa_ent))(oa_wa oa_wa))(u_csn cp_ent))	26	10
5513	((((p_isn ca_ent)(((ca_wa p_re) cp_ent) oa_ent))(oa_wa u_hdip))(u_isn u_hdip))	26	10
5514	((((p_isn ca_ent)(((ca_wa p_re) cp_ent) oa_ent))(u_hdip u_hdip))(u_isn u_hdip))	31	10
5515	((((p_isn ca_ent)(((p_re ca_wa) p_csn) oa_ent))(u_hdip u_hdip))(u_isn u_hdip))	25	10
5516	((((u_hdip u_hdip)(((oa_wa u_csn) oa_ent) oa_wa))(u_isn u_hdip))(u_isn u_hdip))	20	10
5517	((((u_isn u_hdip)((u_isn oa_wa)((u_csn cp_ent) p_csn))) ca_ent)(ca_wa ca_wa))	25	10
5518	((((u_isn u_hdip)((oa_wa (u_csn cp_ent)) oa_ent))(u_isn u_hdip))(u_isn u_hdip))	21	10
5519	((((p_csn (oa_ent (u_isn u_hdip)))(u_isn u_hdip))(u_isn u_hdip))(u_isn u_hdip))	28	10
5520	((((p_csn (oa_ent (u_isn u_hdip)))(u_isn u_hdip))((u_csn cp_ent) p_csn)) ca_ent)	24	10
5521	((((p_csn ((oa_ent u_isn) u_hdip))(u_isn oa_wa))((u_csn cp_ent)(p_csn ca_ent)))	25	10
5522	(((((ca_ent ca_wa)((p_isn ca_wa) p_csn)) oa_ent)(oa_wa u_hdip))(u_isn u_hdip))	25	10
5523	(((((ca_ent ca_wa)((p_re ca_wa) p_csn)) oa_ent)(oa_wa u_hdip))(u_isn u_hdip))	29	10
5524	(((((ca_ent ca_wa)((p_re ca_wa) p_csn)) oa_ent)(u_hdip u_hdip))(u_isn u_hdip))	33	10
5525	(((((ca_ent ca_wa)((p_re ca_wa) p_csn)) oa_ent)(u_isn u_hdip))(oa_wa u_hdip))	32	10
5526	(((((ca_ent ca_wa)((p_re ca_wa) p_csn)) oa_ent)(u_isn u_hdip))(u_isn oa_wa))	34	10
5527	(((((cp_ent ca_ent)((ca_wa p_re) cp_ent)) oa_ent)(oa_wa oa_wa))(u_csn cp_ent))	30	10
5528	(((((cp_ent ca_ent)((ca_wa p_re) cp_ent)) oa_ent)(oa_wa u_hdip))(u_isn u_hdip))	32	10
5529	(((((cp_ent ca_ent)((ca_wa p_re) cp_ent)) oa_ent)(u_hdip u_hdip))(u_isn u_hdip))	36	10
5530	(((((cp_ent ca_ent)((ca_wa p_re) cp_ent)) oa_ent)(u_isn oa_wa))(u_isn oa_wa))	32	10
5531	(((((cp_ent ca_ent)((ca_wa p_re) cp_ent)) oa_ent)(u_isn oa_wa))(u_isn u_hdip))	32	10
5532	(((((p_isn ca_ent)((p_re ca_wa) p_csn)) oa_ent)(u_isn u_hdip))(u_isn u_hdip))	30	10
5533	(((((u_hdip (u_isn oa_wa)) u_csn)(cp_ent ca_wa))(p_isn p_re))(ca_wa p_csn))	19	10
5534	((((((ca_ent ca_wa) cp_ent) oa_ent)(oa_wa u_hdip))(u_isn u_hdip))(u_isn u_hdip))	30	10
5535	((((((ca_ent ca_wa) cp_ent) oa_ent)(u_hdip u_hdip))(u_isn u_hdip))(u_isn oa_wa))	30	10
5536	((((((ca_ent ca_wa) cp_ent) oa_ent)(u_hdip u_hdip))(u_isn u_hdip))(u_isn u_hdip))	30	10
5537	((((((ca_ent ca_wa) cp_ent) oa_ent)(u_isn oa_wa))(u_isn oa_wa))(u_isn u_hdip))	27	10
5538	((((((ca_ent ca_wa) cp_ent) oa_ent)(u_isn u_hdip))(u_isn oa_wa))(u_isn u_hdip))	29	10
5539	((((((ca_ent ca_wa) cp_ent) oa_ent)(u_isn u_hdip))(u_isn u_hdip))(u_isn u_hdip))	36	10
5540	((((((ca_ent ca_wa) p_csn) oa_ent)(u_hdip u_hdip))(u_isn u_hdip))(u_isn u_hdip))	23	10
5541	((((((ca_wa p_re) ca_wa) oa_ent)(oa_wa u_hdip))(u_isn u_hdip))(oa_wa u_csn))	28	10
5542	((((((ca_wa p_re) ca_wa) oa_ent)(oa_wa u_hdip))(u_isn u_hdip))(u_isn oa_wa))	26	10
5543	((((((ca_wa p_re) ca_wa) oa_ent)(oa_wa u_hdip))(u_isn u_hdip))(u_isn u_hdip))	27	10
5544	((((((ca_wa p_re) ca_wa) oa_ent)(u_hdip u_hdip))(u_isn u_hdip))(oa_wa u_csn))	30	10
5545	((((((ca_wa p_re) ca_wa) oa_ent)(u_hdip u_hdip))(u_isn u_hdip))(u_isn oa_wa))	28	10
5546	((((((ca_wa p_re) ca_wa) oa_ent)(u_hdip u_hdip))(u_isn u_hdip))(u_isn u_hdip))	28	10
5547	((((((ca_wa p_re) ca_wa) oa_ent)(u_isn u_hdip))(u_isn oa_wa))(u_csn cp_ent))	24	10
5548	((((((ca_wa p_re) cp_ent) oa_ent)(oa_wa u_hdip))(u_isn u_hdip))(u_isn u_hdip))	29	10
5549	((((((ca_wa p_re) cp_ent) oa_ent)(u_hdip u_hdip))(u_isn u_hdip))(oa_wa u_csn))	31	10
5550	((((((ca_wa p_re) cp_ent) oa_ent)(u_hdip u_hdip))(u_isn u_hdip))(u_isn oa_wa))	31	10
5551	((((((ca_wa p_re) cp_ent) oa_ent)(u_hdip u_hdip))(u_isn u_hdip))(u_isn u_hdip))	31	10
5552	((((((ca_wa p_re) cp_ent) oa_ent)(u_isn oa_wa))(u_isn oa_wa))(u_isn u_hdip))	26	10
5553	((((((ca_wa p_re) cp_ent) oa_ent)(u_isn u_hdip))(u_isn u_hdip))(u_isn oa_wa))	32	10
5554	((((((ca_wa p_re) cp_ent) oa_ent)(u_isn u_hdip))(u_isn u_hdip))(u_isn u_hdip))	33	10
5555	((((((cp_ent ca_ent) cp_ent) oa_ent)(oa_wa u_hdip))(u_isn u_hdip))(u_isn u_hdip))	28	10
5556	((((((cp_ent ca_ent) cp_ent) oa_ent)(u_hdip u_hdip))(u_isn u_hdip))(u_isn u_hdip))	28	10
5557	((((((cp_ent ca_ent) cp_ent) oa_ent)(u_isn oa_wa))(u_isn oa_wa))(u_isn u_hdip))	27	10
5558	((((((cp_ent ca_wa) cp_ent) oa_ent)(oa_wa u_hdip))(u_isn u_hdip))(u_isn oa_wa))	29	10
5559	((((((cp_ent ca_wa) cp_ent) oa_ent)(oa_wa u_hdip))(u_isn u_hdip))(u_isn u_hdip))	23	10
5560	((((((cp_ent ca_wa) cp_ent) oa_ent)(u_hdip u_hdip))(u_isn u_hdip))(u_isn oa_wa))	30	10
5561	((((((cp_ent ca_wa) cp_ent) oa_ent)(u_hdip u_hdip))(u_isn u_hdip))(u_isn u_hdip))	28	10
5562	((((((cp_ent oa_ent) oa_wa) u_csn)(u_isn cp_ent))(ca_ent p_isn))(p_re ca_wa))	24	10
5563	((((((cp_ent oa_ent) oa_wa) u_csn)(u_isn cp_ent)) ca_ent)((ca_wa p_re) ca_wa))	26	10
5564	((((((cp_ent oa_ent) oa_wa) u_csn)(u_isn cp_ent)) ca_ent)((ca_wa p_re) cp_ent))	23	10
5565	((((((cp_ent oa_ent) oa_wa) u_csn)(u_isn cp_ent))(ca_ent ca_wa))(p_isn p_re))	24	10
5566	((((((p_re ca_wa) p_csn) oa_ent)(u_hdip u_hdip))(u_isn u_hdip))(u_isn u_hdip))	26	10
5567	((((((p_re ca_wa) p_csn) oa_ent)(u_isn u_hdip))(u_isn oa_wa))(u_isn oa_wa))	27	10
5568	((((((p_re ca_wa) p_csn) oa_ent)(u_isn u_hdip))(u_isn oa_wa))(u_isn u_hdip))	30	10
5569	((ca_ent ca_wa)((p_isn p_re)(((ca_wa (p_csn cp_ent))(oa_ent u_hdip))(u_isn u_hdip))))	30	11
5570	(((u_csn cp_ent)(ca_ent ca_wa))(((p_re ca_wa) p_csn)((p_isn oa_ent)(u_isn u_hdip))))	21	11
5571	((u_hdip oa_wa)(((u_csn cp_ent)(p_csn (ca_ent ca_wa)))((p_re ca_wa)(cp_ent oa_ent))))	20	11
5572	((oa_wa (u_csn (cp_ent ca_ent)))((((ca_wa p_re) ca_wa)((p_csn (oa_ent u_isn)) u_hdip)))	23	11
5573	((oa_wa (u_csn (cp_ent ca_ent)))((((ca_wa p_re) ca_wa)((p_csn oa_ent)(u_isn u_hdip))))	25	11
5574	((oa_wa (u_csn (cp_ent ca_ent)))((((ca_wa p_re) ca_wa)(p_csn oa_ent))(u_hdip u_hdip)))	23	11
5575	((oa_wa (u_csn (cp_ent ca_ent)))((((ca_wa p_re) ca_wa)(p_csn oa_ent))(u_isn u_hdip)))	24	11
5576	(((p_csn (p_isn ca_ent))((((ca_wa p_re) ca_wa)((cp_ent oa_ent) u_hdip))(u_isn u_hdip))	20	11
5577	((u_csn (cp_ent ca_wa))(((p_re ca_wa)(cp_ent oa_ent))((oa_wa u_hdip)(u_isn oa_wa))))	20	11
5578	((((u_csn cp_ent) p_csn) ca_ent)(((ca_wa p_re) cp_ent)((p_isn oa_ent)(u_isn u_hdip))))	19	11
5579	(((u_csn cp_ent)(p_csn (ca_ent ca_wa)))(((p_re ca_wa)(cp_ent oa_ent))(u_hdip u_hdip)))	24	11
5580	(((u_csn cp_ent)(p_isn ca_ent))(((ca_wa p_re) cp_ent)((p_isn oa_ent)(u_isn u_hdip))))	19	11
5581	(((u_csn cp_ent)(p_isn ca_ent))((((ca_wa p_re) ca_wa)(p_csn (p_isn oa_ent))) u_hdip))	18	11
5582	(((u_csn (p_isn ca_ent))(((ca_wa p_re) ca_wa)((cp_ent oa_ent) u_hdip)))(u_isn u_hdip))	24	11
5583	(((u_csn (p_isn ca_ent))(((ca_wa p_re) ca_wa)((p_csn oa_ent) u_hdip)))(u_isn u_hdip))	21	11
5584	((u_hdip (u_isn oa_wa))((u_csn (cp_ent ca_ent))(((ca_wa p_re) ca_wa)(cp_ent oa_ent))))	25	11
5585	((u_hdip (u_isn oa_wa))(((u_csn cp_ent)(p_csn (ca_ent ca_wa)))((p_re ca_wa) oa_ent)))	17	11
5586	((ca_ent ca_wa)(((p_isn p_re)((ca_wa (p_csn cp_ent)) oa_ent))((u_isn oa_wa) u_hdip)))	26	11
5587	((cp_ent ca_ent)((p_isn p_re)(((ca_wa (p_csn cp_ent))(oa_ent u_hdip))(u_isn u_hdip))))	28	11
5588	(((oa_wa (u_csn cp_ent))(ca_ent ca_wa))(((p_re ca_wa)(cp_ent oa_ent))(oa_wa u_hdip)))	24	11
5589	(((oa_wa (u_csn cp_ent))(ca_ent ca_wa))(((p_re ca_wa)(p_csn oa_ent))(u_isn u_hdip)))	24	11
5590	(((oa_wa (u_csn cp_ent)) ca_ent)((((ca_wa p_re) ca_wa)(p_csn oa_ent))(u_isn u_hdip)))	26	11
5591	(((oa_wa (u_csn cp_ent)) ca_ent)((((ca_wa p_re) ca_wa)(cp_ent oa_ent)))(oa_wa u_hdip))	26	11
5592	(((oa_wa (u_csn cp_ent))(ca_ent ca_wa))(((p_re ca_wa) p_csn)(oa_ent (u_isn u_hdip))))	24	11
5593	(((oa_wa (u_csn cp_ent))(ca_ent ca_wa))((((p_re ca_wa)(cp_ent oa_ent))(oa_wa u_hdip)))	23	11
5594	(((oa_wa (u_csn cp_ent))(ca_ent ca_wa))((((p_re ca_wa)(cp_ent oa_ent))(u_hdip u_hdip)))	28	11
5595	(((oa_wa (u_csn cp_ent))(ca_ent ca_wa))((((p_re ca_wa)(cp_ent oa_ent))(u_isn u_hdip)))	29	11
5596	(((oa_wa (u_csn cp_ent))(ca_ent ca_wa))((((p_re ca_wa)(p_csn oa_ent))(u_hdip u_hdip)))	24	11
5597	(((oa_wa (u_csn cp_ent))(ca_ent p_isn))(((p_re ca_wa)(p_csn oa_ent))(u_isn u_hdip)))	25	11
5598	(((oa_wa (u_csn cp_ent))(ca_ent ca_wa))(((p_re ca_wa) p_csn)((p_isn oa_ent) u_hdip)))	18	11
5599	((((oa_wa (u_csn cp_ent)) p_csn)(ca_ent ca_wa))(((p_re ca_wa)((cp_ent oa_ent) u_hdip)))	22	11
5600	(((oa_wa (u_csn cp_ent))(p_csn ca_ent))((((ca_wa p_re) ca_wa)((cp_ent oa_ent) u_hdip)))	23	11
5601	(((oa_wa (u_csn cp_ent))(p_csn ca_ent))((((ca_wa p_re) cp_ent)((oa_ent u_isn) u_hdip)))	25	11
5602	(((oa_wa u_csn)(p_isn ca_ent))((((ca_wa p_re) ca_wa)(p_csn oa_ent))(u_isn u_hdip)))	19	11
5603	((((oa_wa u_csn)(p_isn ca_ent))(((ca_wa p_re)((p_csn oa_ent) u_hdip)))(u_isn u_hdip))	17	11
5604	(((oa_wa u_hdip) oa_wa)((u_csn (cp_ent ca_ent))(((ca_wa p_re) ca_wa)(cp_ent oa_ent))))	25	11
5605	(((oa_wa u_hdip) oa_wa)(((u_csn cp_ent)(p_isn ca_wa))((p_re ca_wa)(cp_ent oa_ent))))	20	11
5606	(((oa_wa u_hdip)(u_isn oa_wa))(((u_csn cp_ent)(p_csn (ca_ent ca_wa)))(p_re ca_wa)))	22	11
5607	(((u_csn cp_ent)(p_isn ca_ent))((((ca_wa p_re) ca_wa)(cp_ent oa_ent))(oa_wa u_hdip)))	22	11
5608	(((u_csn cp_ent)(p_isn ca_ent))((((ca_wa p_re) ca_wa)(p_csn oa_ent))(u_isn u_hdip)))	22	11
5609	((((u_csn cp_ent)(p_isn ca_ent))((ca_wa p_re)((p_csn oa_ent) u_hdip)))(u_isn u_hdip))	21	11
5610	(((u_csn cp_ent)(p_isn ca_ent))((((ca_wa p_re) ca_wa)(p_csn oa_ent))(u_isn u_hdip)))	23	11
5611	(((u_hdip (u_csn cp_ent)) ca_ent)((((ca_wa p_re) ca_wa)(cp_ent oa_ent))(oa_wa u_hdip)))	21	11
5612	(((u_hdip (u_csn cp_ent))(ca_ent ca_wa))(((p_re ca_wa)(cp_ent oa_ent))(oa_wa u_hdip)))	20	11
5613	(((u_hdip (u_csn cp_ent)) ca_ent)((((ca_wa p_re) ca_wa)(cp_ent oa_ent))(u_hdip u_hdip)))	25	11
5614	(((u_isn oa_wa) u_hdip)((u_csn (cp_ent ca_ent))(((ca_wa p_re) ca_wa)(cp_ent oa_ent))))	22	11
5615	((((cp_ent oa_ent)(oa_wa u_hdip))((u_isn u_hdip)(oa_wa (u_csn cp_ent))))(oa_ent oa_wa))	18	11
5616	(((oa_wa u_csn)(p_csn (p_isn ca_ent)))(((ca_wa p_re) cp_ent)((oa_ent u_isn) u_hdip)))	18	11
5617	(((oa_wa u_hdip)(((u_csn cp_ent) p_csn)(ca_ent ca_wa)))((p_re ca_wa)(cp_ent oa_ent)))	20	11
5618	(((oa_wa u_hdip)((u_csn cp_ent)(p_csn ca_ent)))(((ca_wa p_re) ca_wa)(oa_ent u_isn)))	18	11
5619	(((oa_wa u_hdip)(((u_csn cp_ent) p_csn) ca_ent))(((ca_wa p_re) cp_ent)(oa_ent u_isn)))	20	11
5620	(((p_isn ca_ent)(((ca_wa p_re) ca_wa)(cp_ent oa_ent)))((oa_wa u_hdip)(u_isn oa_wa)))	25	11
5621	(((u_csn (cp_ent ca_ent))((ca_wa p_re) ca_wa))((p_csn (oa_ent u_isn))(u_hdip u_hdip)))	28	11
5622	((((u_csn cp_ent)(ca_ent ca_wa))((p_re ca_wa) oa_ent))((oa_wa u_hdip)(u_isn oa_wa)))	17	11
5623	(((u_csn cp_ent)(p_csn (p_isn ca_ent)))((((ca_wa p_re) ca_wa)((cp_ent oa_ent) u_hdip)))	19	11
5624	(((u_csn cp_ent)(p_csn (p_isn ca_ent)))((((ca_wa p_re) cp_ent)((oa_ent u_isn) u_hdip)))	21	11
5625	(((u_csn cp_ent)(p_csn (p_isn ca_ent)))((((ca_wa p_re) cp_ent)((p_isn oa_ent) u_hdip)))	16	11
5626	((((u_csn cp_ent)(p_isn ca_ent))((ca_wa p_re) ca_wa))((cp_ent oa_ent)(u_isn u_hdip)))	30	11
5627	(((u_hdip oa_wa)((u_csn cp_ent)(ca_ent ca_wa)))(((p_re ca_wa)((cp_ent oa_ent) oa_wa)))	21	11
5628	(((u_hdip oa_wa)(((u_csn cp_ent)(ca_ent ca_wa))(p_re ca_wa)))((cp_ent oa_ent) u_hdip))	26	11
5629	(((u_hdip oa_wa)(u_csn (cp_ent ca_ent)))((((ca_wa p_re) cp_ent)((oa_ent u_isn) u_hdip)))	25	11
5630	(((u_hdip oa_wa)((u_csn cp_ent)(ca_ent ca_wa)))((((p_re ca_wa) oa_ent)(u_hdip u_hdip)))	19	11
5631	(((u_hdip (u_isn oa_wa))((u_csn cp_ent)(p_csn ca_ent)))((((ca_wa p_re) ca_wa) oa_ent))	18	11
5632	(((u_hdip oa_wa)((u_csn cp_ent)(p_csn ca_ent)))((((ca_wa p_re) ca_wa)(oa_ent u_isn)))	18	11
5633	(((u_hdip oa_wa)(((u_csn cp_ent) p_csn) ca_ent))(((ca_wa p_re) cp_ent)(oa_ent u_isn)))	20	11
5634	(((u_hdip oa_wa)((u_csn cp_ent) p_csn))((((ca_wa p_re) ca_wa)((cp_ent oa_ent) u_hdip)))	21	11
5635	(((u_hdip u_hdip)((u_csn cp_ent)(p_csn (ca_ent ca_wa))))((p_re ca_wa)(cp_ent oa_ent)))	17	11
5636	(((u_hdip u_hdip)((u_csn cp_ent)(p_csn ca_ent)))((((ca_wa p_re) ca_wa)(oa_ent u_isn)))	16	11
5637	(((u_hdip u_hdip)((u_csn cp_ent)(p_csn ca_ent)))((((ca_wa p_re) cp_ent)(oa_ent u_isn)))	17	11
5638	(((u_isn cp_ent)(ca_ent ca_wa))((p_isn p_re)((ca_wa (p_csn cp_ent))(oa_ent u_hdip))))	18	11
5639	(((oa_wa (u_csn cp_ent))(p_isn ca_ent))(((ca_wa p_re)((p_csn oa_ent)(u_isn u_hdip)))))	18	11
5640	(((oa_wa (u_csn cp_ent))(p_isn ca_ent))((((ca_wa p_re) ca_wa)((cp_ent oa_ent) u_hdip)))	24	11
5641	(((oa_wa (u_csn cp_ent))(p_isn ca_ent))((((ca_wa p_re) ca_wa)(p_csn (oa_ent u_isn))))	20	11
5642	(((oa_wa (u_csn cp_ent))(p_isn ca_ent))((((ca_wa p_re) cp_ent)(oa_ent (oa_wa u_hdip)))))	20	11
5643	(((oa_wa (u_csn cp_ent))(p_isn ca_ent))((((ca_wa p_re) cp_ent)(oa_ent (u_isn u_hdip)))))	24	11
5644	(((oa_wa (u_csn cp_ent))(p_isn ca_ent))((((ca_wa p_re) cp_ent)((oa_ent u_isn) u_hdip)))	25	11
5645	(((oa_wa (u_csn cp_ent))(p_isn ca_ent))(((p_re ca_wa) p_csn)(oa_ent (u_isn u_hdip))))	19	11
5646	(((oa_wa (u_csn cp_ent))(p_isn ca_ent))((((p_re ca_wa)(cp_ent oa_ent))(u_isn u_hdip)))	23	11
5647	(((oa_wa (u_csn cp_ent))(p_isn ca_wa))(((p_re ca_wa)(cp_ent oa_ent))(oa_wa u_hdip)))	21	11
5648	(((oa_wa (u_csn cp_ent))(p_isn ca_wa))((((p_re ca_wa)(cp_ent oa_ent))(oa_wa u_hdip)))	20	11
5649	(((oa_wa (u_csn cp_ent))(p_isn ca_wa))((((p_re ca_wa)(cp_ent oa_ent))(u_isn u_hdip)))	21	11
5650	(((oa_wa (u_csn cp_ent))(p_csn (p_isn ca_ent)))((((ca_wa p_re) ca_wa)(cp_ent oa_ent)))	19	11
5651	(((oa_wa (u_csn cp_ent))(p_csn (p_isn ca_ent)))((((ca_wa p_re) ca_wa)(oa_ent u_isn)))	16	11
5652	(((oa_wa (u_csn cp_ent))(p_csn (p_isn ca_ent)))((((ca_wa p_re) cp_ent)(oa_ent u_isn)))	19	11
5653	(((oa_wa (u_csn cp_ent))(p_csn (p_isn ca_ent)))((((ca_wa p_re) cp_ent)(oa_ent u_hdip)))	19	11
5654	(((oa_wa (u_csn cp_ent))((ca_wa p_re) ca_wa))((p_csn (oa_ent u_isn))(u_hdip u_hdip)))	23	11
5655	(((u_csn cp_ent)(ca_ent ca_wa))((p_re p_re)(p_csn ((p_isn oa_ent)(u_isn u_hdip)))))	20	11
5656	((((u_csn cp_ent)(ca_ent ca_wa))(p_re p_re))((p_csn (oa_ent u_isn))(u_hdip u_hdip)))	24	11
5657	(((u_csn (ca_ent ca_wa))(p_re p_re))(((p_csn oa_ent)(u_isn u_hdip))(u_isn u_hdip)))	23	11
5658	((((u_csn cp_ent)(ca_ent ca_wa))(p_isn ca_wa))(((cp_ent oa_ent) u_hdip)(u_isn u_hdip)))	32	11
5659	((((u_csn cp_ent)(p_isn ca_ent))((ca_wa p_re) ca_wa))((cp_ent oa_ent)(u_isn u_hdip)))	29	11
5660	(((u_csn (p_isn ca_ent))((ca_wa p_re)((p_csn oa_ent)(u_isn u_hdip))))(u_isn u_hdip))	23	11
5661	(((u_csn (p_isn ca_ent))((p_re p_re)((p_csn oa_ent)(u_isn u_hdip))))(u_isn u_hdip))	20	11
5662	(((u_csn (u_isn cp_ent)) ca_ent)((((ca_wa p_re) ca_wa)(cp_ent oa_ent))(oa_wa u_hdip)))	19	11
5663	(((u_csn (u_isn cp_ent)) ca_ent)((((ca_wa p_re) ca_wa)(cp_ent oa_ent))(u_isn u_hdip)))	22	11
5664	(((u_csn (u_isn cp_ent))(p_isn ca_ent))((((ca_wa p_re) cp_ent)((oa_ent u_isn) u_hdip)))	21	11
5665	(((u_hdip (oa_wa u_csn))(cp_ent ca_ent))((((ca_wa p_re) ca_wa)((cp_ent oa_ent) u_hdip)))	24	11
5666	(((u_hdip (oa_wa u_csn))(ca_ent ca_wa))((((p_re ca_wa)(cp_ent oa_ent))(oa_wa u_hdip)))	18	11
5667	(((u_hdip (oa_wa u_csn))(cp_ent ca_ent))((((ca_wa p_re) cp_ent)((oa_ent u_isn) u_hdip)))	23	11
5668	(((u_hdip (oa_wa u_csn))(cp_ent ca_wa))(((p_re ca_wa)(cp_ent oa_ent))(oa_wa u_hdip)))	18	11
5669	(((u_hdip (oa_wa u_csn))(cp_ent ca_wa))((((p_re ca_wa)(cp_ent oa_ent))(oa_wa u_hdip)))	17	11
5670	(((u_hdip (oa_wa u_csn))(cp_ent ca_wa))((((p_re ca_wa)(cp_ent oa_ent))(u_hdip u_hdip)))	18	11
5671	(((u_hdip (oa_wa u_csn))(p_csn (ca_ent ca_wa)))(((p_re ca_wa)((cp_ent oa_ent) u_hdip)))	18	11
5672	(((u_hdip (u_csn cp_ent))(p_csn (ca_ent ca_wa)))(((p_re ca_wa)((cp_ent oa_ent) u_hdip)))	20	11
5673	(((u_hdip (u_csn cp_ent))(p_csn ca_ent))(((ca_wa p_re) cp_ent)((oa_ent u_isn) u_hdip)))	20	11
5674	(((u_hdip (u_csn cp_ent))(p_isn ca_ent))((((ca_wa p_re) ca_wa)((cp_ent oa_ent) u_hdip)))	23	11
5675	(((u_hdip (u_csn cp_ent))(p_isn ca_ent))((((ca_wa p_re) cp_ent)((oa_ent u_isn) u_hdip)))	21	11
5676	(((u_hdip (u_csn cp_ent))(p_isn ca_wa))(((p_re ca_wa)(cp_ent oa_ent))(oa_wa u_hdip)))	20	11
5677	(((u_hdip (u_csn cp_ent))(p_isn ca_wa))((((p_re ca_wa)(cp_ent oa_ent))(oa_wa u_hdip)))	19	11
5678	(((u_hdip (u_csn cp_ent))(p_isn ca_wa))((((p_re ca_wa)(cp_ent oa_ent))(u_hdip u_hdip)))	20	11
5679	(((u_hdip (u_csn cp_ent))(p_csn (p_isn ca_ent)))((((ca_wa p_re) ca_wa)(cp_ent oa_ent)))	16	11
5680	(((u_hdip (u_csn cp_ent))(p_csn (p_isn ca_ent)))((((ca_wa p_re) ca_wa)(oa_ent u_isn)))	15	11
5681	(((u_hdip (u_csn cp_ent))(p_csn (p_isn ca_ent)))((((ca_wa p_re) cp_ent)(oa_ent u_isn)))	16	11
5682	(((u_hdip (u_isn oa_wa))(u_csn (cp_ent ca_ent)))((((ca_wa p_re) ca_wa)(oa_ent u_isn)))	20	11
5683	(((u_hdip (u_isn oa_wa))(u_csn (cp_ent ca_ent)))((((ca_wa p_re) cp_ent)(oa_ent u_isn)))	24	11
5684	(((u_hdip (u_isn oa_wa))(u_csn (cp_ent ca_ent)))((((ca_wa p_re) cp_ent)(oa_ent oa_wa)))	19	11
5685	(((u_hdip (u_isn oa_wa))(u_csn (cp_ent ca_ent)))((((ca_wa p_re) cp_ent)(oa_ent u_hdip)))	24	11
5686	(((u_hdip (u_isn u_csn))(cp_ent ca_ent))((((ca_wa p_re) cp_ent)((oa_ent u_isn) u_hdip)))	23	11
5687	(((u_hdip (u_isn u_csn))(ca_ent ca_wa))((((p_re ca_wa)(cp_ent oa_ent))(u_hdip u_hdip)))	21	11
5688	(((u_hdip (u_isn u_csn))(cp_ent ca_wa))(((p_re ca_wa)(cp_ent oa_ent))(oa_wa u_hdip)))	17	11
5689	(((u_hdip (u_isn u_csn))(cp_ent ca_wa))((((p_re ca_wa)(cp_ent oa_ent))(u_hdip u_hdip)))	17	11
5690	(((ca_ent ca_wa)(((p_re ca_wa) p_csn)((p_isn oa_ent)(u_isn u_hdip))))(u_isn u_hdip))	22	11
5691	(((ca_ent ca_wa)((p_isn ca_wa)((p_csn cp_ent)((oa_ent u_isn) u_hdip))))(u_isn u_hdip))	30	11
5692	(((ca_ent ca_wa)((((p_re ca_wa) p_csn)((p_isn oa_ent)(u_isn u_hdip))))(u_isn u_hdip))	24	11
5693	((((oa_wa u_csn) p_csn)(p_isn ca_ent))((((ca_wa p_re) cp_ent)((oa_ent u_isn) u_hdip)))	20	11
5694	(((oa_wa u_csn)(cp_ent ca_wa))(((p_re ca_wa)(cp_ent oa_ent))((oa_wa u_hdip) oa_wa)))	23	11

5695	([[oa_wa u_csn](p_isn ca_ent])))(((ca_wa p_re) ca_wa)(cp_ent oa_ent))(u_isn u_hdip]])	24	11
5696	((([oa_wa u_hdip) oa_wa)(u_csn (cp_ent ca_ent])))((ca_wa p_re) ca_wa)(cp_ent oa_ent)))	27	11
5697	((([oa_wa u_hdip) oa_wa)(u_csn (cp_ent ca_ent])))((ca_wa p_re) cp_ent)(oa_ent u_isn)))	26	11
5698	((([oa_wa u_hdip) oa_wa)(u_csn (cp_ent ca_ent])))((ca_wa p_re) cp_ent)(oa_ent oa_wa)))	20	11
5699	((([oa_wa u_hdip) oa_wa)(u_csn (ca_ent ca_wa])))((p_re ca_wa)(cp_ent oa_ent) oa_wa)))	24	11
5700	((([oa_wa u_hdip) oa_wa)(u_csn (cp_ent ca_wa])))((p_re ca_wa)(cp_ent oa_ent) oa_wa)))	21	11
5701	((([oa_wa u_hdip) oa_wa))(u_csn cp_ent)(p_csn ca_ent])))((ca_wa p_re) cp_ent) oa_ent])	21	11
5702	((([oa_wa u_hdip)(oa_wa (u_csn cp_ent]))(ca_ent ca_wa))((p_re ca_wa)(cp_ent oa_ent)))	21	11
5703	((([oa_wa u_hdip)(oa_wa u_csn])(cp_ent ca_ent])))((ca_wa p_re) cp_ent)(oa_ent u_isn)))	20	11
5704	((([oa_wa u_hdip)(u_isn oa_wa])(u_csn (cp_ent ca_ent])))((ca_wa p_re) cp_ent) oa_ent])	19	11
5705	((([oa_wa u_hdip)(u_isn oa_wa)))(u_csn cp_ent)(p_csn ca_ent]))(ca_wa p_re) cp_ent])	22	11
5706	((([p_csn oa_ent)(u_isn u_hdip])(u_isn oa_wa)))((u_csn cp_ent) p_csn)(ca_ent p_isn]])	25	11
5707	((([p_csn oa_ent)(u_isn u_hdip])(u_isn oa_wa)))((u_csn cp_ent) p_csn)(ca_ent ca_wa]])	25	11
5708	((([u_csn cp_ent) p_csn)(ca_ent ca_wa])))((p_re ca_wa)(cp_ent oa_ent))(u_hdip u_hdip]))	23	11
5709	((([u_csn cp_ent) p_csn)(p_isn ca_ent])))((ca_wa p_re) ca_wa)(cp_ent oa_ent) u_hdip]))	21	11
5710	((([u_csn cp_ent) p_csn)(p_isn ca_ent])))((ca_wa p_re) cp_ent)(oa_ent u_isn) u_hdip]))	23	11
5711	((([u_csn cp_ent)(ca_wa p_re])(p_isn ca_wa]))(p_csn (oa_ent u_isn))(u_hdip u_hdip]))	21	11
5712	((([u_hdip oa_wa) u_csn)(cp_ent ca_ent]))(ca_wa p_re))(p_csn oa_ent)(u_isn u_hdip]]))	18	11
5713	((([u_hdip oa_wa) u_csn)(cp_ent ca_ent])))(ca_wa p_re) ca_wa]))(cp_ent oa_ent) u_hdip]))	27	11
5714	((([u_hdip oa_wa) u_csn)(ca_ent ca_wa])))((p_re ca_wa)(cp_ent oa_ent))(oa_wa u_hdip]))	21	11
5715	((([u_hdip oa_wa) u_csn))(cp_ent ca_ent))(ca_wa p_re) cp_ent])))(oa_ent u_isn) u_hdip]))	25	11
5716	((([u_hdip oa_wa) u_csn)(cp_ent ca_ent])))(ca_wa p_re) cp_ent))(oa_ent u_isn) u_hdip]))	26	11
5717	((([u_hdip oa_wa) u_csn)(cp_ent ca_ent])))((ca_wa p_re)(p_csn oa_ent))(u_hdip u_hdip]))	18	11
5718	((([u_hdip oa_wa) u_csn)(cp_ent ca_ent])))(ca_wa p_re)(p_csn oa_ent))(u_isn u_hdip]))	18	11
5719	((([u_hdip oa_wa) u_csn)(ca_ent ca_wa])))((p_re ca_wa) p_csn) oa_ent)(u_isn u_hdip]))	22	11
5720	((([u_hdip oa_wa) u_csn)(ca_ent ca_wa])))((p_re ca_wa) p_csn))(p_isn oa_ent) u_hdip]))	17	11
5721	((([u_hdip oa_wa) u_csn)(ca_ent ca_wa))(p_isn p_re]))(cp_ent oa_ent)(u_isn u_hdip]))	26	11
5722	((([u_hdip oa_wa) u_csn)(cp_ent ca_wa])))((p_re ca_wa))(cp_ent oa_ent)(oa_wa u_hdip]]))	21	11
5723	((([u_hdip oa_wa) u_csn)(cp_ent ca_wa])))((p_re ca_wa)(cp_ent oa_ent))(oa_wa u_hdip]))	20	11
5724	((([u_hdip oa_wa) u_csn)(cp_ent ca_wa])))((p_re ca_wa)(cp_ent oa_ent))(u_hdip u_hdip]))	21	11
5725	((([u_hdip oa_wa) u_csn)(cp_ent ca_wa])))((p_re ca_wa)(cp_ent oa_ent))(u_isn u_hdip]))	21	11
5726	((([u_isn cp_ent)(p_isn ca_ent])))(ca_wa p_re) ca_wa]))(cp_ent oa_ent)(u_isn u_hdip]))	23	11
5727	((([u_isn oa_wa) u_csn)(cp_ent ca_ent])))(ca_wa p_re) ca_wa))(cp_ent oa_ent) oa_wa]))	21	11
5728	((([u_isn oa_wa) u_csn)(cp_ent ca_ent])))(ca_wa p_re) ca_wa))(cp_ent oa_ent) u_hdip]))	26	11
5729	((([u_isn oa_wa) u_csn)(ca_ent ca_wa])))((p_re ca_wa)(cp_ent oa_ent))(oa_wa u_hdip]))	20	11
5730	((([u_isn oa_wa) u_csn)(cp_ent ca_ent])))((ca_wa p_re) cp_ent))(oa_ent u_isn) u_hdip]))	25	11
5731	((([u_isn oa_wa) u_csn)(cp_ent ca_wa])))((p_re ca_wa))(cp_ent oa_ent)(oa_wa u_hdip]]))	20	11
5732	((([u_isn oa_wa) u_csn)(cp_ent ca_wa])))((p_re ca_wa)(cp_ent oa_ent))(oa_wa u_hdip]))	19	11
5733	((([u_isn oa_wa) u_csn)(cp_ent ca_wa])))((p_re ca_wa)(cp_ent oa_ent))(u_hdip u_hdip]))	20	11
5734	((([u_isn oa_wa))(u_csn cp_ent) p_csn]) ca_ent])))(ca_wa p_re) ca_wa)(cp_ent oa_ent]))	21	11
5735	((([u_isn oa_wa) u_hdip)(u_csn (cp_ent ca_ent])))(ca_wa p_re) ca_wa)(oa_ent u_isn]))	17	11
5736	((([u_isn oa_wa) u_hdip)(u_csn (cp_ent ca_ent])))(ca_wa p_re) cp_ent)(oa_ent u_isn]))	21	11
5737	((([u_isn oa_wa) u_hdip)(u_csn (cp_ent ca_ent])))((ca_wa p_re) cp_ent)(oa_ent u_hdip]))	21	11
5738	((([u_isn oa_wa) u_hdip))(u_csn cp_ent)(p_csn (ca_ent ca_wa])))((p_re ca_wa) oa_ent]))	17	11
5739	((([u_isn oa_wa) u_hdip))(u_csn cp_ent)(p_csn ca_ent])))(ca_wa p_re) ca_wa) oa_ent]))	18	11
5740	((p_csn ((p_isn ca_ent))((ca_wa p_re) ca_wa))(cp_ent oa_ent) u_hdip])))(u_isn u_hdip]))	23	11
5741	((ca_ent ca_wa))(p_isn p_re))((ca_wa (p_csn cp_ent))(oa_ent oa_wa))(u_isn u_hdip]))	27	11
5742	((ca_ent ca_wa))(p_isn p_re))((ca_wa (p_csn cp_ent))(oa_ent u_isn))(oa_wa u_hdip]))	28	11
5743	((ca_ent ca_wa))(p_re ca_wa)))(p_csn oa_ent)(u_isn u_hdip))(u_isn oa_wa) u_hdip]))	28	11
5744	(((ca_ent ca_wa))(p_isn ca_wa))(p_csn cp_ent)(oa_ent (u_isn u_hdip])))(u_isn u_hdip])	29	11
5745	(((ca_ent ca_wa))(p_re ca_wa))(p_csn cp_ent))(oa_ent u_isn) u_hdip])))(u_isn oa_wa])	22	11
5746	((ca_ent ca_wa))(p_isn ca_wa))(p_csn cp_ent)(oa_ent u_isn])))(oa_wa u_hdip) oa_wa]))	26	11
5747	((ca_ent ca_wa)))((p_re ca_wa)(p_csn oa_ent))(u_isn u_hdip))(u_isn oa_wa) u_hdip]))	31	11
5748	(((ca_ent ca_wa) p_re))))(ca_wa (p_csn cp_ent]) oa_ent)(u_isn u_hdip))(u_isn u_hdip]))	30	11
5749	((ca_ent ca_wa)))((p_re ca_wa)(p_csn cp_ent))(oa_ent (u_isn u_hdip))(u_isn u_hdip]))	31	11
5750	((ca_ent ca_wa)))((p_re ca_wa)(p_csn cp_ent]))(oa_ent u_isn) u_hdip))(u_isn oa_wa]))	23	11
5751	((ca_ent ca_wa)))((p_re ca_wa)(p_csn cp_ent]))(oa_ent u_isn) u_hdip))(u_isn u_hdip]))	32	11
5752	((ca_ent p_isn)))((p_re (ca_wa p_csn))(cp_ent oa_ent))(u_isn u_hdip))(u_isn u_hdip]))	30	11
5753	((ca_ent ca_wa)))((p_isn ca_wa) p_csn))(p_isn oa_ent)(u_isn u_hdip]))(u_isn u_hdip]))	19	11
5754	((ca_ent ca_wa)))(p_isn p_re))((ca_wa (p_csn cp_ent))(oa_ent oa_wa))(u_isn oa_wa]))	25	11
5755	((ca_ent p_isn))(p_re ca_wa)))(p_csn oa_ent)(u_isn u_hdip]))(u_isn oa_wa) u_hdip])))	26	11
5756	((ca_ent p_isn)))((p_re ca_wa)(p_csn oa_ent))(u_isn u_hdip]))(u_isn oa_wa) u_hdip]))	27	11
5757	((ca_ent p_isn)))((p_re ca_wa) p_csn))(p_isn oa_ent)(u_isn u_hdip]))(u_isn u_hdip]))	22	11
5758	(((ca_ent p_isn)))(p_re ca_wa))(cp_ent oa_ent) u_hdip))(u_isn u_hdip]))(u_isn oa_wa])	33	11
5759	((cp_ent ca_ent))(p_isn p_re))((ca_wa (p_csn cp_ent))(oa_ent oa_wa))(u_isn u_hdip]))	25	11
5760	((cp_ent ca_ent)))(p_isn p_re))((ca_wa (p_csn cp_ent]) oa_ent]))(u_isn oa_wa) u_hdip]))	24	11
5761	((cp_ent ca_ent))(p_isn p_re))((ca_wa (p_csn cp_ent))(oa_ent u_isn))(oa_wa u_hdip]))	26	11
5762	((cp_ent ca_ent)))((ca_wa p_re)(ca_wa (p_csn cp_ent]))(oa_ent oa_wa))(u_isn u_hdip]))	26	11
5763	((cp_ent ca_ent)))((ca_wa p_re) ca_wa)))(p_csn oa_ent) u_hdip))(u_isn oa_wa) u_hdip]))	24	11
5764	((cp_ent ca_ent)))((ca_wa p_re) ca_wa)))(p_csn (oa_ent u_isn)) u_hdip)(oa_wa u_hdip]))	25	11
5765	((cp_ent ca_ent)))((ca_wa p_re) ca_wa)))(p_csn oa_ent)(u_isn u_hdip))(u_isn oa_wa]))	24	11
5766	((cp_ent ca_ent)))((ca_wa p_re) ca_wa)))(p_csn oa_ent)(u_isn u_hdip))(u_isn u_hdip]))	31	11
5767	((cp_ent ca_ent)))((ca_wa p_re) ca_wa)))(p_csn oa_ent)(u_hdip oa_wa))(u_isn u_hdip]))	22	11
5768	(((cp_ent ca_ent))(p_isn ca_wa))(p_csn cp_ent))(oa_ent u_isn) u_hdip])))(u_isn u_hdip])	28	11
5769	(((cp_ent ca_ent))(p_isn ca_wa))(p_csn cp_ent)(oa_ent (u_isn u_hdip])))(u_isn u_hdip])	27	11
5770	((cp_ent ca_ent))(p_isn ca_wa))(p_csn cp_ent)(oa_ent u_isn])))(oa_wa u_hdip) oa_wa]))	24	11
5771	((cp_ent ca_ent)))((ca_wa p_re) ca_wa))(cp_ent oa_ent)(oa_wa u_hdip])))(u_isn u_hdip]))	29	11
5772	((cp_ent ca_ent))))(ca_wa p_re) ca_wa)(p_csn oa_ent))(u_isn u_hdip))(oa_wa u_hdip]))	26	11
5773	((cp_ent ca_ent)))((ca_wa p_re) ca_wa)(p_csn oa_ent) u_hdip]))(u_isn oa_wa) u_hdip]))	25	11
5774	((cp_ent ca_ent)))((ca_wa p_re) ca_wa)(p_csn oa_ent)(oa_wa u_hdip]))(u_isn u_hdip]))	23	11
5775	((cp_ent ca_ent)))((ca_wa p_re)(ca_wa (p_csn cp_ent]))(oa_ent u_hdip))(u_isn u_hdip]))	29	11
5776	((cp_ent ca_ent)))((ca_wa p_re)(ca_wa (p_csn cp_ent]))(oa_ent oa_wa))(u_isn oa_wa]))	26	11
5777	((cp_ent ca_ent)))((p_re (ca_wa p_csn))(cp_ent oa_ent))(u_isn u_hdip))(u_isn u_hdip]))	28	11
5778	((cp_ent ca_ent)))((ca_wa p_re) ca_wa)(cp_ent oa_ent]))(oa_wa u_hdip)(u_isn oa_wa])))	25	11
5779	((cp_ent ca_ent)))((ca_wa p_re) ca_wa))(cp_ent oa_ent)(u_isn u_hdip]))(u_isn oa_wa]))	28	11
5780	((cp_ent ca_ent)))((ca_wa p_re) ca_wa))(cp_ent oa_ent)(u_isn u_hdip]))(u_isn u_hdip]))	33	11
5781	((cp_ent ca_ent)))((ca_wa p_re) ca_wa))(p_csn oa_ent)(u_isn u_hdip]))(u_isn oa_wa]))	23	11
5782	((cp_ent ca_ent)))((p_isn ca_wa) p_csn))(p_isn oa_ent)(u_isn u_hdip]))(u_isn u_hdip]))	19	11
5783	((cp_ent ca_ent)))(p_isn p_re))(ca_wa (p_csn cp_ent))(oa_ent oa_wa]))(u_isn oa_wa]))	25	11
5784	(((cp_ent ca_ent)))(ca_wa p_re) ca_wa))(p_csn (p_isn oa_ent)) u_hdip]))(u_isn u_hdip]))	23	11
5785	(((cp_ent ca_ent)))((ca_wa p_re) ca_wa)(cp_ent oa_ent))(u_hdip u_hdip]))(u_isn u_hdip]))	36	11
5786	((cp_ent ca_ent))))(ca_wa p_re) ca_wa)(p_csn oa_ent])(oa_wa u_hdip))(u_isn u_hdip]))	22	11
5787	((cp_ent ca_ent))))(ca_wa p_re) ca_wa)(p_csn oa_ent))(u_hdip u_hdip))(u_isn u_hdip]))	27	11
5788	((cp_ent ca_ent))))(ca_wa p_re) ca_wa)(p_csn oa_ent))(u_isn u_hdip))(u_isn oa_wa]))	28	11
5789	((oa_wa u_hdip))))(u_csn cp_ent) p_csn) ca_ent)))(ca_wa p_re) ca_wa)(cp_ent oa_ent]))	22	11
5790	((oa_wa u_hdip))))(u_csn cp_ent) p_csn) ca_ent)))(ca_wa p_re) ca_wa)(oa_ent u_isn]))	19	11
5791	((oa_wa u_hdip))))(u_isn oa_wa)(u_csn cp_ent) p_csn)) ca_ent))(ca_wa p_re) ca_wa]))	17	11
5792	((p_csn ca_ent)))((ca_wa p_re) ca_wa))(cp_ent oa_ent)(u_isn u_hdip))(u_isn u_hdip]))	26	11
5793	((p_isn ca_ent))))(ca_wa p_re)(ca_wa (p_csn cp_ent)))(oa_ent u_hdip))(u_isn u_hdip]))	25	11
5794	((p_isn ca_ent))(p_isn p_re))((ca_wa (p_csn cp_ent))(oa_ent u_hdip))(u_isn u_hdip])))	24	11
5795	((p_isn ca_ent)))(ca_wa p_re) ca_wa)))(p_csn cp_ent))(oa_ent oa_wa))(u_isn u_hdip]))	19	11
5796	((p_isn ca_ent)))(p_isn p_re))(ca_wa (p_csn cp_ent]) oa_ent]))(u_isn oa_wa) u_hdip]))	20	11
5797	((p_isn ca_ent)))((ca_wa p_re) ca_wa)))(p_csn oa_ent)(u_isn u_hdip))(u_isn oa_wa]))	20	11
5798	(((p_isn ca_ent)))(ca_wa p_re) ca_wa))(p_csn oa_ent)(u_isn u_hdip))))(u_isn u_hdip])	29	11
5799	((p_isn ca_ent)))(ca_wa p_re) ca_wa)))(p_csn oa_ent) u_hdip))(u_isn oa_wa) u_hdip]))	21	11
5800	((p_isn ca_ent)))(ca_wa p_re) ca_wa)))(p_csn oa_ent)(u_hdip oa_wa))(u_isn u_hdip]))	19	11
5801	((p_isn ca_ent)))(ca_wa p_re) ca_wa)))(p_csn oa_ent)(u_isn u_hdip))(oa_wa u_hdip]))	24	11
5802	((p_isn ca_ent)))(p_isn ca_wa))(p_csn cp_ent))(oa_ent u_isn) u_hdip]))(u_isn u_hdip]))	24	11
5803	((p_isn ca_ent)))(p_isn ca_wa))(p_csn cp_ent)(oa_ent (u_isn u_hdip]))(u_isn u_hdip]))	23	11
5804	((p_isn ca_ent))))(ca_wa p_re) ca_wa))(cp_ent oa_ent)(oa_wa u_hdip]))(u_isn u_hdip]))	27	11
5805	((p_isn ca_ent))))(ca_wa p_re) ca_wa)(p_csn oa_ent))(u_isn u_hdip))(u_isn u_hdip]))	30	11
5806	((p_isn ca_ent))))(p_re ca_wa)(p_csn oa_ent))(u_isn u_hdip]))(u_isn oa_wa) u_hdip]))	22	11
5807	((p_isn ca_ent))))(ca_wa p_re) ca_wa))(p_csn oa_ent) u_hdip]))(u_isn oa_wa) u_hdip]))	24	11
5808	((p_isn ca_ent))))(ca_wa p_re) ca_wa))(p_csn oa_ent)(oa_wa u_hdip]))(u_isn u_hdip]))	19	11
5809	((p_isn ca_ent))))(ca_wa p_re)(ca_wa (p_csn cp_ent)))(oa_ent oa_wa))(u_isn oa_wa]))	22	11
5810	((p_isn ca_ent))))(p_re (ca_wa p_csn))(cp_ent oa_ent))(u_isn u_hdip))(u_isn u_hdip]))	24	11
5811	((p_isn ca_ent))))(ca_wa p_re) ca_wa)(cp_ent oa_ent))(oa_wa u_hdip)(u_isn oa_wa]))	21	11
5812	((p_isn ca_ent))))(ca_wa p_re) ca_wa))(cp_ent oa_ent)(u_isn u_hdip]))(u_isn oa_wa]))	26	11
5813	((p_isn ca_ent))))(ca_wa p_re) ca_wa))(cp_ent oa_ent)(u_isn u_hdip]))(u_isn u_hdip]))	30	11
5814	((p_isn ca_ent))))(ca_wa p_re) ca_wa))(p_csn oa_ent)(u_isn u_hdip))(u_isn oa_wa]))	20	11
5815	(((p_isn ca_ent)))(ca_wa p_re) cp_ent))(oa_ent u_isn) u_hdip])))(u_isn oa_wa) u_hdip])	29	11
5816	((p_isn ca_ent))))(ca_wa p_re) cp_ent))(p_isn oa_ent)(u_isn u_hdip]))(u_isn u_hdip]))	23	11
5817	((p_isn ca_ent))))(p_isn ca_wa) p_csn))(p_isn oa_ent)(u_isn u_hdip]))(u_isn u_hdip]))	18	11
5818	((p_isn ca_ent))))(ca_wa p_re) ca_wa))(p_csn (p_isn oa_ent)) u_hdip))(u_isn u_hdip]))	24	11
5819	(((p_isn ca_ent))))(ca_wa p_re) ca_wa)(cp_ent oa_ent))(u_hdip u_hdip]))(u_isn u_hdip])	31	11
5820	((p_isn ca_ent))))(ca_wa p_re) ca_wa)(p_csn oa_ent))(oa_wa u_hdip))(u_isn u_hdip]))	18	11
5821	((p_isn ca_ent)))((p_re ca_wa))(p_csn oa_ent) u_hdip))(u_isn u_hdip))(u_isn u_hdip]))	23	11
5822	((p_isn ca_wa))))(p_isn p_re)(p_csn cp_ent))(oa_ent u_isn) u_hdip))(u_isn u_hdip]))	29	11
5823	((p_isn ca_wa))))(p_isn p_re)(p_csn cp_ent))(oa_ent (u_isn u_hdip]))(u_isn oa_wa]))	20	11
5824	((p_isn ca_wa))))(p_isn p_re)(p_csn cp_ent))(oa_ent (u_isn u_hdip]))(u_isn u_hdip]))	28	11
5825	((p_isn ca_wa))))(p_isn p_re)(p_csn cp_ent))(oa_ent u_isn) u_hdip))(u_isn oa_wa]))	21	11
5826	((p_isn ca_wa))))(p_re ca_wa) p_csn))(p_isn oa_ent)(u_isn u_hdip]))(u_isn u_hdip]))	24	11
5827	(((u_csn cp_ent)(ca_ent ca_wa]))(p_re ca_wa))(p_csn (oa_ent u_isn))(u_hdip u_hdip]]))	25	11
5828	((u_csn (cp_ent ca_ent)))((ca_wa p_re) ca_wa)(p_csn (p_isn oa_ent]))(u_isn u_hdip]))	21	11
5829	((u_csn (cp_ent ca_ent])))(ca_wa p_re)(p_csn oa_ent)(u_isn u_hdip]))(u_isn u_hdip]))	23	11
5830	(((u_csn cp_ent)(ca_ent ca_wa])))((p_re ca_wa)(p_csn (oa_ent u_hdip]))(u_isn u_hdip]))	23	11
5831	(((u_csn cp_ent)(ca_ent ca_wa])))((p_re ca_wa)(p_csn (oa_ent u_isn]))(u_hdip u_hdip]))	26	11
5832	(((u_csn cp_ent)(ca_ent ca_wa])))(p_re ca_wa))(cp_ent oa_ent) oa_wa))(u_isn oa_wa]))	25	11
5833	(((u_csn cp_ent)(ca_ent ca_wa])))(p_re ca_wa))(cp_ent oa_ent) oa_wa))(u_isn u_hdip]))	25	11
5834	(((u_csn cp_ent)(ca_ent ca_wa])))(p_re ca_wa))(cp_ent oa_ent) u_hdip))(u_isn oa_wa]))	26	11
5835	((u_csn (cp_ent ca_ent])))((ca_wa p_re) ca_wa))(cp_ent oa_ent) u_hdip))(u_isn u_hdip]))	29	11
5836	(((u_csn cp_ent)(ca_ent ca_wa])))((p_re ca_wa)(p_csn oa_ent) u_hdip))(u_isn u_hdip]))	25	11
5837	((u_csn (cp_ent ca_ent])))((ca_wa p_re) ca_wa)(cp_ent oa_ent]))(u_isn oa_wa) u_hdip]))	26	11
5838	((u_csn (cp_ent ca_ent)))((ca_wa p_re) cp_ent))(oa_ent u_isn) u_hdip))(u_isn u_hdip]))	32	11
5839	((u_csn (cp_ent ca_ent])))((ca_wa p_re) cp_ent) oa_ent))(oa_wa u_hdip)(u_isn oa_wa]))	23	11
5840	((u_csn (cp_ent ca_ent)))((ca_wa p_re) cp_ent))(oa_ent u_isn) u_hdip))(u_isn oa_wa]))	28	11
5841	((u_csn (cp_ent ca_ent])))((ca_wa p_re) cp_ent)(oa_ent u_hdip))(u_isn oa_wa) u_hdip]))	24	11
5842	((u_csn (cp_ent ca_ent)))((ca_wa p_re) cp_ent)(oa_ent (u_isn u_hdip]))(u_isn oa_wa]))	27	11
5843	((u_csn (cp_ent ca_ent)))((ca_wa p_re) cp_ent)(oa_ent (u_isn u_hdip]))(u_isn u_hdip]))	31	11
5844	((u_csn (cp_ent ca_ent)))((ca_wa p_re) cp_ent))(p_isn oa_ent) u_hdip))(u_isn u_hdip]))	19	11
5845	((u_csn (cp_ent ca_ent])))(ca_wa p_re)(p_csn oa_ent))(u_isn u_hdip])(u_isn u_hdip]))	24	11
5846	(((u_csn cp_ent)(ca_ent ca_wa])))(p_re ca_wa) oa_ent)(u_hdip u_hdip))(u_isn u_hdip]))	22	11
5847	(((u_csn cp_ent)(ca_ent ca_wa])))(p_re ca_wa) oa_ent)(u_isn u_hdip))(u_isn u_hdip]))	23	11
5848	(((u_csn cp_ent)(ca_ent ca_wa])))(p_re ca_wa) p_csn)(oa_ent u_hdip))(u_isn u_hdip]))	24	11
5849	(((u_csn cp_ent)(ca_ent ca_wa])))(p_re ca_wa) p_csn)(p_isn oa_ent))(u_isn u_hdip]))	22	11
5850	((u_csn (cp_ent ca_ent)))((p_re ca_wa)(cp_ent oa_ent))(u_isn u_hdip))(u_isn u_hdip]))	28	11
5851	((u_hdip oa_wa))))(u_csn cp_ent) p_csn) ca_ent)))(ca_wa p_re) ca_wa)(oa_ent u_isn]))	19	11
5852	((u_hdip u_hdip))))(u_isn oa_wa) u_csn)(cp_ent ca_ent])))(ca_wa p_re) ca_wa) oa_ent]))	20	11
5853	((u_hdip u_hdip))))(u_isn oa_wa) u_csn)(cp_ent ca_wa]))((p_re ca_wa)(cp_ent oa_ent]))	19	11
5854	((u_isn oa_wa)))(u_csn cp_ent)(p_csn ca_ent])))(ca_wa p_re) ca_wa)(oa_ent u_isn]))	19	11
5855	((u_isn u_hdip))))(u_isn oa_wa) u_csn)(cp_ent ca_ent])))(ca_wa p_re) cp_ent) oa_ent]))	20	11
5856	((oa_wa (u_csn (cp_ent ca_ent])))((ca_wa p_re))(p_csn oa_ent) u_hdip))(u_isn u_hdip]))	20	11
5857	((oa_wa (u_csn (cp_ent ca_ent])))((ca_wa p_re) ca_wa)(cp_ent oa_ent))(oa_wa u_hdip]))	26	11
5858	((oa_wa (u_csn (cp_ent ca_ent])))((ca_wa p_re) ca_wa)(cp_ent oa_ent))(u_hdip u_hdip]))	29	11
5859	((oa_wa (u_csn (cp_ent ca_ent])))((ca_wa p_re) ca_wa)(cp_ent oa_ent))(u_isn oa_wa]))	26	11
5860	((oa_wa (u_csn (cp_ent ca_ent])))((ca_wa p_re) ca_wa)(cp_ent oa_ent))(u_isn u_hdip]))	30	11
5861	((oa_wa (u_csn (cp_ent ca_ent])))((ca_wa p_re) cp_ent)(oa_ent u_hdip))(u_isn u_hdip]))	29	11
5862	((oa_wa (u_csn (cp_ent ca_ent])))((ca_wa p_re) cp_ent)(oa_ent u_isn))(u_hdip u_hdip]))	29	11
5863	((oa_wa (u_csn (cp_ent ca_ent])))((ca_wa p_re) cp_ent) oa_ent))(u_hdip oa_wa) u_hdip]))	22	11
5864	((oa_wa (u_csn (cp_ent ca_ent])))((ca_wa p_re) cp_ent) oa_ent))(u_isn oa_wa) u_hdip]))	26	11
5865	((oa_wa (u_csn (cp_ent ca_ent])))((ca_wa p_re) cp_ent)(oa_ent oa_wa))(u_isn oa_wa]))	23	11
5866	((oa_wa (u_csn (cp_ent ca_ent])))((ca_wa p_re) cp_ent)(oa_ent oa_wa))(u_isn u_hdip]))	24	11
5867	((oa_wa (u_csn (cp_ent ca_ent])))((ca_wa p_re) cp_ent)(oa_ent u_hdip))(u_isn oa_wa]))	25	11
5868	((oa_wa (u_csn (cp_ent ca_ent])))((p_re ca_wa) p_csn)(oa_ent u_isn))(u_hdip u_hdip]))	24	11
5869	((p_csn (ca_ent ca_wa])))((p_re ca_wa)(cp_ent oa_ent))(u_isn u_hdip))(u_isn oa_wa]))	24	11
5870	((p_csn (ca_ent ca_wa])))((p_re ca_wa)(cp_ent oa_ent))(u_isn u_hdip))(u_isn u_hdip]))	27	11
5871	((p_csn (p_isn ca_ent])))((p_re ca_wa)(cp_ent oa_ent))(u_isn u_hdip))(u_isn u_hdip]))	19	11
5872	((p_csn (p_isn ca_wa])))((p_re ca_wa)(cp_ent oa_ent))(u_isn u_hdip))(u_isn u_hdip]))	23	11
5873	(((u_csn cp_ent)(ca_ent ca_wa])))((p_re ca_wa) p_csn)(oa_ent u_isn))(u_hdip u_hdip]))	27	11
5874	((u_csn (cp_ent ca_wa])))((p_re ca_wa)(cp_ent oa_ent))(oa_wa u_hdip))(u_isn oa_wa]))	19	11
5875	((u_csn (cp_ent ca_wa])))((p_re ca_wa)(cp_ent oa_ent))(u_hdip u_hdip))(u_isn oa_wa]))	21	11
5876	((u_csn (cp_ent ca_wa])))((p_re ca_wa)(cp_ent oa_ent))(u_hdip u_hdip))(u_isn u_hdip]))	25	11
5877	((u_csn (cp_ent ca_wa])))((p_re ca_wa)(cp_ent oa_ent))(u_isn u_hdip))(u_isn oa_wa]))	23	11
5878	((u_csn (cp_ent ca_wa])))((p_re ca_wa)(cp_ent oa_ent))(u_isn u_hdip))(u_isn u_hdip]))	22	11
5879	((([u_csn cp_ent) p_csn) ca_ent])))(ca_wa p_re) ca_wa)(cp_ent oa_ent))(u_hdip u_hdip]))	26	11
5880	(((u_csn cp_ent)(p_csn ca_ent])))(ca_wa p_re) ca_wa)(oa_ent u_isn))(u_hdip u_hdip]))	22	11
5881	((([u_csn cp_ent) p_csn) ca_ent])))(ca_wa p_re) cp_ent)(oa_ent u_hdip))(u_isn u_hdip]))	26	11
5882	((([u_csn cp_ent) p_csn) ca_ent])))(ca_wa p_re) cp_ent)(oa_ent u_isn))(u_hdip u_hdip]))	26	11
5883	((u_csn (p_isn ca_ent])))(ca_wa p_re))(p_csn oa_ent)(u_isn u_hdip]))(u_isn u_hdip]))	19	11
5884	((u_csn (p_isn ca_ent])))(ca_wa p_re) ca_wa)(p_csn (oa_ent u_hdip]))(u_isn u_hdip]))	19	11
5885	((u_csn (p_isn ca_ent])))((ca_wa p_re) ca_wa))(cp_ent oa_ent) oa_wa))(u_isn oa_wa]))	20	11
5886	((u_csn (p_isn ca_ent])))((ca_wa p_re) ca_wa))(cp_ent oa_ent) u_hdip))(u_isn oa_wa]))	21	11
5887	((u_csn (p_isn ca_ent])))((ca_wa p_re) ca_wa)(cp_ent oa_ent]))(u_isn oa_wa) u_hdip]))	21	11
5888	(((u_csn (p_isn ca_ent])))(ca_wa p_re) cp_ent))(oa_ent u_isn) u_hdip]))(u_isn u_hdip])	26	11
5889	((u_csn (p_isn ca_ent])))(ca_wa p_re) cp_ent)(oa_ent u_hdip]))(u_isn oa_wa) u_hdip]))	20	11
5890	((u_csn (p_isn ca_ent])))((ca_wa p_re) cp_ent))(oa_ent u_isn) u_hdip))(u_isn oa_wa]))	22	11
5891	(((u_csn cp_ent)(p_isn ca_ent])))(ca_wa p_re) cp_ent)(p_isn oa_ent))(u_hdip u_hdip]))	16	11
5892	((u_csn (p_isn ca_ent])))((ca_wa p_re) cp_ent)(oa_ent (u_isn u_hdip]))(u_isn oa_wa]))	21	11
5893	((u_csn (p_isn ca_ent])))(ca_wa p_re)(p_csn oa_ent))(u_isn u_hdip))(u_isn u_hdip]))	20	11
5894	(((u_csn (p_isn ca_ent])))(p_re ca_wa) p_csn) oa_ent)(u_isn u_hdip]))(u_isn u_hdip]))	22	11
5895	(((u_csn cp_ent)(p_isn ca_ent])))(p_re ca_wa) p_csn)(p_isn oa_ent))(u_isn u_hdip]))	19	11
5896	((u_csn (p_isn ca_ent])))((p_re ca_wa)(cp_ent oa_ent))(u_isn u_hdip))(u_isn u_hdip]))	23	11
5897	(((u_hdip (u_isn oa_wa)))((u_csn cp_ent) p_csn)(ca_ent ca_wa])))(p_re ca_wa) oa_ent]))	18	11
5898	((u_hdip (u_isn oa_wa))))(u_csn cp_ent) p_csn) ca_ent)))(ca_wa p_re) ca_wa) oa_ent]))	19	11
5899	(((ca_ent ca_wa) cp_ent)))(p_csn (oa_ent u_isn))(u_hdip (u_isn oa_wa)))(u_isn u_hdip]))	19	11
5900	(((cp_ent oa_ent)(u_isn u_hdip))(u_isn u_hdip))(u_isn u_hdip))(u_isn oa_wa) u_csn]))	32	11
5901	(((oa_wa (u_csn cp_ent)) ca_ent))(ca_wa p_re)(p_csn ((p_isn oa_ent)(u_isn u_hdip])))	17	11
5902	(((oa_wa (u_csn cp_ent)) ca_ent)))(ca_wa p_re) ca_wa))(p_csn (oa_ent u_isn)) u_hdip]))	25	11
5903	(((oa_wa (u_csn cp_ent]) ca_ent)))(ca_wa p_re)(p_csn (oa_ent u_hdip]))(u_isn u_hdip]))	19	11
5904	(((oa_wa (u_csn cp_ent]) ca_ent)))(ca_wa p_re))(p_csn oa_ent) u_hdip))(u_isn u_hdip]))	21	11
5905	(((oa_wa (u_csn cp_ent]) ca_ent))))(ca_wa p_re) ca_wa)(p_csn oa_ent))(u_hdip u_hdip]))	25	11
5906	(((oa_wa (u_csn cp_ent]) ca_ent))))(ca_wa p_re) ca_wa)(cp_ent oa_ent))(oa_wa u_hdip]))	27	11
5907	(((oa_wa (u_csn cp_ent]) ca_ent))))(ca_wa p_re) ca_wa)(cp_ent oa_ent))(u_hdip u_hdip]))	30	11
5908	(((oa_wa (u_csn cp_ent]) ca_ent))))(ca_wa p_re) ca_wa)(cp_ent oa_ent))(u_isn u_hdip]))	31	11
5909	(((oa_wa (u_csn cp_ent]) ca_ent))))(ca_wa p_re) ca_wa)(oa_ent u_isn))(u_hdip u_hdip]))	23	11
5910	(((oa_wa (u_csn cp_ent]) ca_ent))))(ca_wa p_re) cp_ent)(oa_ent u_hdip))(u_isn u_hdip]))	30	11
5911	(((oa_wa (u_csn cp_ent]) ca_ent))))(ca_wa p_re) cp_ent)(oa_ent u_isn))(u_hdip u_hdip]))	30	11
5912	(((oa_wa (u_csn cp_ent]) ca_ent))))(ca_wa p_re) cp_ent)(oa_ent oa_wa))(u_isn oa_wa]))	25	11
5913	(((oa_wa (u_csn cp_ent]) ca_ent))))(ca_wa p_re) cp_ent)(oa_ent oa_wa))(u_isn u_hdip]))	26	11
5914	(((oa_wa (u_csn cp_ent]) ca_ent))))(ca_wa p_re) cp_ent)(oa_ent u_hdip))(u_isn oa_wa]))	26	11
5915	(((oa_wa (u_csn cp_ent])(ca_ent ca_wa])))(p_re ca_wa) p_csn) oa_ent)(u_hdip u_hdip]))	26	11
5916	(((oa_wa (u_csn cp_ent])(ca_ent ca_wa])))(p_re ca_wa) p_csn) oa_ent)(u_isn u_hdip]))	27	11
5917	(((oa_wa u_csn)(cp_ent ca_wa])))(p_re ca_wa))(cp_ent oa_ent) oa_wa))(u_hdip u_hdip]))	23	11
5918	(((oa_wa u_csn)(cp_ent ca_wa])))(p_re ca_wa))(cp_ent oa_ent) oa_wa))(u_isn oa_wa]))	23	11
5919	(((oa_wa u_csn)(cp_ent ca_wa])))(p_re ca_wa))(cp_ent oa_ent) oa_wa))(u_isn u_hdip]))	23	11
5920	(((oa_wa u_csn)(cp_ent ca_wa])))(p_re ca_wa))(cp_ent oa_ent) u_hdip))(u_isn oa_wa]))	24	11
5921	(((oa_wa u_csn)(cp_ent ca_wa])))((p_re ca_wa) oa_ent)(u_hdip u_hdip))(u_isn u_hdip]))	18	11
5922	(((oa_wa u_csn)(cp_ent ca_wa])))((p_re ca_wa) oa_ent)(u_isn u_hdip))(u_isn oa_wa]))	17	11
5923	(((oa_wa (u_csn cp_ent])(p_csn ca_ent])))((ca_wa p_re) ca_wa) oa_ent)(u_hdip u_hdip]))	19	11
5924	(((oa_wa (u_csn cp_ent])(p_csn ca_ent])))(ca_wa p_re) cp_ent) oa_ent)(u_isn u_hdip]))	25	11
5925	(((oa_wa u_csn)(p_isn ca_ent])))(ca_wa p_re) ca_wa)(cp_ent oa_ent))(oa_wa u_hdip]))	20	11
5926	(((oa_wa u_csn)(p_isn ca_ent])))(ca_wa p_re) ca_wa)(cp_ent oa_ent))(u_hdip u_hdip]))	23	11
5927	(((oa_wa u_csn)(p_isn ca_ent])))(ca_wa p_re) cp_ent)(oa_ent oa_wa))(u_isn u_hdip]))	19	11
5928	(((oa_wa u_csn)(p_isn ca_ent])))(ca_wa p_re) cp_ent)(oa_ent u_hdip))(u_isn oa_wa]))	19	11
5929	(((oa_wa u_hdip) oa_wa)((u_csn (cp_ent ca_ent])))(ca_wa p_re) cp_ent)(oa_ent u_isn]))	24	11
5930	(((oa_wa u_hdip) oa_wa)((u_csn (cp_ent ca_ent])))(ca_wa p_re) ca_wa)(cp_ent oa_ent]))	24	11
5931	(((oa_wa u_hdip)(u_isn oa_wa))))(u_csn cp_ent) p_csn)(ca_ent p_isn))(p_re ca_wa]))	23	11
5932	(((oa_wa u_hdip)(u_isn oa_wa))))(u_csn cp_ent) p_csn) ca_ent))(ca_wa p_re) ca_wa]))	23	11
5933	(((p_isn oa_ent)(u_isn u_hdip))(u_isn u_hdip))(u_isn u_hdip))(u_isn oa_wa) u_csn]]))	22	11
5934	(((u_csn cp_ent)(ca_ent ca_wa])))(p_re ca_wa))(cp_ent oa_ent) u_hdip))(u_isn u_hdip]))	28	11
5935	(((u_csn cp_ent)(oa_ent oa_wa))(u_isn u_hdip))(u_isn u_hdip))(u_isn oa_wa) u_csn]))	24	11
5936	(((u_csn cp_ent)(p_isn ca_ent])))(ca_wa p_re)(p_csn (oa_ent u_hdip]))(u_isn u_hdip]))	19	11
5937	(((u_csn cp_ent)(p_isn ca_ent])))(ca_wa p_re) ca_wa)(cp_ent oa_ent))(oa_wa u_hdip]))	24	11
5938	(((u_csn cp_ent)(p_isn ca_ent])))(ca_wa p_re) ca_wa)(cp_ent oa_ent))(u_hdip u_hdip]))	27	11
5939	(((u_csn cp_ent)(p_isn ca_ent])))(ca_wa p_re) ca_wa)(cp_ent oa_ent))(u_isn oa_wa]))	23	11
5940	(((u_csn cp_ent)(p_isn ca_ent])))(ca_wa p_re) ca_wa)(cp_ent oa_ent))(u_isn u_hdip]))	28	11
5941	(((u_csn cp_ent)(p_isn ca_ent])))(ca_wa p_re) ca_wa)(oa_ent u_isn))(u_hdip u_hdip]))	22	11
5942	(((u_csn cp_ent)(p_isn ca_ent])))(ca_wa p_re) cp_ent) oa_ent))(u_isn oa_wa) u_hdip]))	23	11
5943	(((u_csn cp_ent)(p_isn ca_ent])))(ca_wa p_re) cp_ent)(oa_ent oa_wa))(u_isn oa_wa]))	22	11
5944	(((u_csn cp_ent)(p_isn ca_ent])))(ca_wa p_re) cp_ent)(oa_ent oa_wa))(u_isn u_hdip]))	23	11
5945	(((u_csn cp_ent)(p_isn ca_ent])))(ca_wa p_re) cp_ent)(oa_ent u_hdip))(u_isn oa_wa]))	23	11
5946	(((u_csn cp_ent)(p_isn ca_ent])))((p_re ca_wa) p_csn)(oa_ent u_hdip))(u_isn u_hdip]))	21	11
5947	(((u_csn cp_ent)(p_isn ca_wa])))(p_re ca_wa))(cp_ent oa_ent) oa_wa))(u_isn oa_wa]))	22	11
5948	(((u_csn cp_ent)(p_isn ca_wa])))(p_re ca_wa))(cp_ent oa_ent) oa_wa))(u_isn u_hdip]))	22	11
5949	(((u_csn cp_ent)(p_isn ca_wa])))(p_re ca_wa))(cp_ent oa_ent) u_hdip))(u_isn oa_wa]))	23	11
5950	(((u_csn cp_ent)(p_isn ca_wa])))(p_re ca_wa))(cp_ent oa_ent) u_hdip))(u_isn u_hdip]))	22	11
5951	(((u_csn cp_ent)(p_isn ca_wa])))((p_re ca_wa) oa_ent)(u_hdip u_hdip))(u_isn u_hdip]))	18	11
5952	(((u_hdip u_csn)(cp_ent ca_ent])))((ca_wa p_re) ca_wa)(oa_ent u_isn))(u_hdip u_hdip]))	21	11
5953	(((u_hdip u_csn)(cp_ent ca_ent])))((ca_wa p_re) cp_ent)(oa_ent u_hdip))(u_isn u_hdip]))	25	11
5954	(((u_hdip u_csn)(cp_ent ca_ent])))((ca_wa p_re) cp_ent)(oa_ent u_isn))(u_hdip u_hdip]))	25	11
5955	(((u_hdip u_csn)(cp_ent ca_ent])))((ca_wa p_re) cp_ent)(oa_ent u_hdip))(u_isn oa_wa]))	20	11
5956	(((u_hdip u_csn)(cp_ent ca_wa])))(p_re ca_wa))(cp_ent oa_ent) u_hdip))(u_isn u_hdip]))	20	11
5957	(((u_isn cp_ent) ca_ent))))(ca_wa p_re) ca_wa)(cp_ent oa_ent]))(u_isn oa_wa) u_hdip]))	20	11
5958	(((u_isn cp_ent) ca_ent)))(ca_wa p_re) cp_ent))(oa_ent u_isn) u_hdip]))(u_isn u_hdip]))	22	11
5959	(((u_isn cp_ent) ca_ent))))(ca_wa p_re) cp_ent)(oa_ent (u_isn u_hdip]))(u_isn u_hdip]))	21	11
5960	(((u_isn cp_ent)(p_isn ca_ent])))((ca_wa p_re) ca_wa)(cp_ent oa_ent))(u_hdip u_hdip]))	21	11
5961	(((u_isn cp_ent)(p_isn ca_ent])))(ca_wa p_re) ca_wa)(cp_ent oa_ent))(u_isn oa_wa]))	18	11
5962	(((u_isn cp_ent)(p_isn ca_ent])))(ca_wa p_re) ca_wa)(cp_ent oa_ent))(u_isn u_hdip]))	21	11
5963	(((u_isn cp_ent)(p_isn ca_ent])))((ca_wa p_re) cp_ent)(oa_ent u_hdip))(u_isn u_hdip]))	20	11
5964	(((u_isn oa_wa) u_hdip))((u_csn cp_ent)(p_csn ca_ent])))(ca_wa p_re) ca_wa) oa_ent]))	17	11
5965	(((u_isn oa_wa) u_hdip))((u_csn cp_ent)(p_csn (p_isn ca_ent])))(ca_wa p_re) ca_wa]))	19	11
5966	(((u_isn oa_wa) u_hdip))))(u_csn cp_ent) p_csn) ca_ent)))(ca_wa p_re) ca_wa) oa_ent]))	18	11
5967	(((u_isn oa_wa) u_hdip))))(u_isn oa_wa) u_csn)(cp_ent ca_ent])))(ca_wa p_re) ca_wa]))	20	11
5968	(((u_isn u_csn)(cp_ent ca_ent])))((ca_wa p_re) ca_wa)(cp_ent oa_ent))(oa_wa u_hdip]))	20	11
5969	(((u_isn u_csn)(cp_ent ca_ent])))((ca_wa p_re) ca_wa)(cp_ent oa_ent))(u_hdip u_hdip]))	23	11
5970	(((u_isn u_csn)(cp_ent ca_ent])))((ca_wa p_re) ca_wa)(cp_ent oa_ent))(u_isn oa_wa]))	19	11
5971	(((u_isn u_csn)(cp_ent ca_ent])))((ca_wa p_re) cp_ent)(oa_ent u_hdip))(u_isn u_hdip]))	23	11
5972	(((u_isn u_csn)(cp_ent ca_ent])))((ca_wa p_re) cp_ent)(oa_ent u_isn))(u_hdip u_hdip]))	23	11
5973	(((u_isn u_csn)(cp_ent ca_ent])))((ca_wa p_re) cp_ent) oa_ent))(u_isn oa_wa) u_hdip]))	19	11
5974	(((u_isn u_csn)(cp_ent ca_ent])))((ca_wa p_re) cp_ent)(oa_ent u_hdip))(u_isn oa_wa]))	19	11
5975	(((u_isn u_csn)(cp_ent ca_wa])))(p_re ca_wa))(cp_ent oa_ent) oa_wa))(u_hdip u_hdip]))	16	11
5976	(((u_isn u_csn)(cp_ent ca_wa])))(p_re ca_wa))(cp_ent oa_ent) u_hdip))(u_isn oa_wa]))	17	11
5977	(((oa_wa u_csn)(p_csn (p_isn ca_ent])))((ca_wa p_re) cp_ent) oa_ent)(u_isn u_hdip]))	18	11
5978	(((u_csn cp_ent)(p_csn (p_isn ca_ent])))((ca_wa p_re) ca_wa) oa_ent)(u_hdip u_hdip]))	17	11
5979	(((u_csn cp_ent)(p_csn (p_isn ca_ent])))((ca_wa p_re) cp_ent) oa_ent)(u_isn u_hdip]))	21	11
5980	(((u_hdip oa_wa)(u_csn (cp_ent ca_ent])))((ca_wa p_re) ca_wa) oa_ent)(u_hdip u_hdip]))	21	11
5981	(((u_hdip oa_wa)(u_csn (cp_ent ca_ent])))((ca_wa p_re) cp_ent) oa_ent)(u_isn oa_wa]))	20	11
5982	(((u_hdip oa_wa)(u_csn (cp_ent ca_ent])))((ca_wa p_re) cp_ent) oa_ent)(u_isn u_hdip]))	25	11
5983	(((oa_wa (u_csn cp_ent))(p_isn ca_ent])))(ca_wa p_re) ca_wa) oa_ent)(u_hdip u_hdip]))	20	11
5984	(((oa_wa (u_csn cp_ent))(p_isn ca_ent])))(ca_wa p_re) ca_wa) oa_ent)(u_isn u_hdip]))	20	11
5985	(((oa_wa (u_csn cp_ent))(p_isn ca_ent])))(ca_wa p_re) cp_ent) oa_ent)(oa_wa oa_wa]))	19	11
5986	(((oa_wa (u_csn cp_ent))(p_isn ca_ent])))(ca_wa p_re) cp_ent) oa_ent)(u_isn oa_wa]))	20	11
5987	(((oa_wa (u_csn cp_ent))(p_isn ca_ent])))(ca_wa p_re) cp_ent) oa_ent)(u_isn u_hdip]))	25	11
5988	(((oa_wa (u_csn cp_ent])(p_isn ca_ent])))(p_re ca_wa) p_csn) oa_ent)(u_hdip u_hdip]))	20	11
5989	(((oa_wa (u_csn cp_ent))(p_isn ca_ent])))(p_re ca_wa) p_csn) oa_ent)(u_isn u_hdip]))	21	11
5990	(((p_csn (oa_ent u_hdip])(u_isn oa_wa])))(u_csn cp_ent) p_csn) ca_ent)(ca_wa ca_wa]))	24	11
5991	(((p_csn (oa_ent u_hdip))(u_isn oa_wa])))(u_csn cp_ent) p_csn) ca_ent)(ca_wa p_re]))	24	11
5992	(((u_csn (u_isn cp_ent]) ca_ent))))(ca_wa p_re) ca_wa)(cp_ent oa_ent))(u_hdip u_hdip]))	22	11
5993	(((u_csn (u_isn cp_ent]) ca_ent))))(ca_wa p_re) ca_wa)(cp_ent oa_ent))(u_isn oa_wa]))	19	11
5994	(((u_csn (u_isn cp_ent]) ca_ent))))(ca_wa p_re) cp_ent)(oa_ent u_hdip))(u_isn u_hdip]))	22	11
5995	(((u_csn (u_isn cp_ent]) ca_ent))))(ca_wa p_re) cp_ent) oa_ent))(u_hdip oa_wa) u_hdip]))	17	11
5996	(((u_csn (u_isn cp_ent]) ca_ent))))(ca_wa p_re) cp_ent)(oa_ent oa_wa))(u_isn oa_wa]))	18	11
5997	(((u_csn (u_isn cp_ent]) ca_ent))))(ca_wa p_re) cp_ent)(oa_ent oa_wa))(u_isn u_hdip]))	19	11
5998	(((u_csn (u_isn cp_ent]) ca_ent))))(ca_wa p_re) cp_ent)(oa_ent u_hdip))(u_isn oa_wa]))	18	11
5999	(((u_csn (u_isn cp_ent])(p_isn ca_ent])))(ca_wa p_re) cp_ent) oa_ent)(u_isn u_hdip]))	21	11
6000	(((u_hdip (oa_wa u_csn])(cp_ent ca_ent])))(ca_wa p_re) cp_ent) oa_ent)(u_isn oa_wa]))	18	11
6001	(((u_hdip (oa_wa u_csn])(cp_ent ca_ent])))(ca_wa p_re) cp_ent) oa_ent)(u_isn u_hdip]))	23	11
6002	(((u_hdip (u_csn cp_ent])(p_csn ca_ent])))(ca_wa p_re) ca_wa) oa_ent)(u_hdip u_hdip]))	18	11
6003	(((u_hdip (u_csn cp_ent])(p_csn ca_ent])))(ca_wa p_re) cp_ent) oa_ent)(u_isn u_hdip]))	20	11
6004	(((u_hdip (u_csn cp_ent])(p_isn ca_ent])))(ca_wa p_re) ca_wa) oa_ent)(u_hdip u_hdip]))	19	11
6005	(((u_hdip (u_csn cp_ent])(p_isn ca_ent])))(ca_wa p_re) cp_ent) oa_ent)(u_isn u_hdip]))	21	11
6006	(((u_hdip (u_isn u_csn])(cp_ent ca_ent])))(ca_wa p_re) cp_ent) oa_ent)(oa_wa u_hdip]))	19	11
6007	(((u_hdip (u_isn u_csn])(cp_ent ca_ent])))(ca_wa p_re) cp_ent) oa_ent)(u_isn u_hdip]))	23	11
6008	(((oa_wa u_csn) p_csn) ca_ent))))(ca_wa p_re) ca_wa)(cp_ent oa_ent))(u_hdip u_hdip]))	22	11
6009	(((oa_wa u_csn) p_csn) ca_ent))))(ca_wa p_re) cp_ent)(oa_ent u_hdip))(u_isn u_hdip]))	23	11
6010	(((oa_wa u_csn) p_csn) ca_ent))))(ca_wa p_re) cp_ent)(oa_ent u_isn))(u_hdip u_hdip]))	23	11
6011	((((oa_wa u_csn) p_csn)(p_isn ca_ent])))(ca_wa p_re) cp_ent) oa_ent)(u_isn u_hdip]))	20	11
6012	((((u_csn cp_ent) p_csn) ca_ent))))(ca_wa p_re) ca_wa)(oa_ent u_isn))(u_hdip u_hdip]))	21	11
6013	((((u_csn cp_ent) p_csn)(p_isn ca_ent])))(ca_wa p_re) ca_wa) oa_ent)(u_hdip u_hdip]))	19	11
6014	((((u_csn cp_ent) p_csn)(p_isn ca_ent])))(ca_wa p_re) cp_ent) oa_ent)(u_isn u_hdip]))	23	11
6015	((((u_hdip oa_wa) u_csn)(cp_ent ca_ent]))(ca_wa p_re))(p_csn (oa_ent u_isn)) u_hdip]))	17	11
6016	((((u_hdip oa_wa) u_csn)(cp_ent ca_ent]))(ca_wa p_re))(p_csn (p_isn oa_ent)) u_hdip]))	16	11
6017	((((u_hdip oa_wa) u_csn)(cp_ent ca_ent])))(ca_wa p_re) ca_wa) oa_ent)(u_hdip u_hdip]))	22	11
6018	((((u_hdip oa_wa) u_csn)(cp_ent ca_ent])))((ca_wa p_re) ca_wa) oa_ent)(u_isn u_hdip]))	22	11
6019	((((u_hdip oa_wa) u_csn)(cp_ent ca_ent])))(ca_wa p_re) cp_ent) oa_ent)(u_isn oa_wa]))	21	11
6020	((((u_hdip oa_wa) u_csn)(cp_ent ca_ent])))(ca_wa p_re) cp_ent) oa_ent)(u_isn u_hdip]))	26	11
6021	((((u_hdip oa_wa) u_csn)(ca_ent ca_wa])))((p_re ca_wa) p_csn) oa_ent)(u_hdip u_hdip]))	21	11
6022	((((u_isn oa_wa) u_csn)(cp_ent ca_ent])))(ca_wa p_re) ca_wa) oa_ent)(u_hdip u_hdip]))	21	11
6023	((((u_isn oa_wa) u_csn)(cp_ent ca_ent])))(ca_wa p_re) cp_ent) oa_ent)(u_isn oa_wa]))	20	11
6024	((((u_isn oa_wa) u_csn)(cp_ent ca_ent])))(ca_wa p_re) cp_ent) oa_ent)(u_isn u_hdip]))	25	11
6025	(((ca_ent ca_wa))(p_re ca_wa))(p_csn cp_ent)(oa_ent u_isn])))(oa_wa u_hdip) oa_wa]))	28	11
6026	(((ca_ent ca_wa))(p_re ca_wa))(p_csn oa_ent)(u_isn u_hdip])))(u_isn oa_wa) u_hdip]))	30	11
6027	(((p_isn ca_wa))(p_re ca_wa) p_csn)(p_isn oa_ent)(u_isn u_hdip])))(u_isn u_hdip]))	22	11
6028	(((ca_ent p_isn))(p_re ca_wa))(p_csn oa_ent)(u_isn u_hdip])))(u_isn oa_wa) u_hdip]))	29	11
6029	(((ca_ent p_isn)))(p_re ca_wa) p_csn))(p_isn oa_ent)(u_isn u_hdip])))(u_isn u_hdip]))	20	11
6030	(((ca_ent p_isn)))(p_re ca_wa)(p_csn oa_ent))(u_isn u_hdip])))(u_isn oa_wa) u_hdip]))	30	11
6031	(((cp_ent ca_ent)))(ca_wa p_re) ca_wa))(p_csn oa_ent)(oa_wa u_hdip])))(u_isn u_hdip]))	25	11
6032	(((cp_ent ca_ent))(p_re ca_wa))(p_csn oa_ent)(u_isn u_hdip])))(u_isn oa_wa) u_hdip]))	27	11
6033	(((cp_ent ca_ent)))(ca_wa p_re) ca_wa)(cp_ent oa_ent])))(oa_wa u_hdip)(u_isn oa_wa]))	33	11
6034	(((cp_ent ca_ent)))(ca_wa p_re) ca_wa)(p_csn oa_ent])))(oa_wa u_hdip)(u_isn oa_wa]))	21	11
6035	(((cp_ent ca_ent)))(ca_wa p_re) ca_wa))(cp_ent oa_ent)(oa_wa u_hdip])))(u_isn oa_wa]))	30	11
6036	(((cp_ent ca_ent)))(ca_wa p_re) ca_wa))(cp_ent oa_ent)(oa_wa u_hdip])))(u_isn u_hdip]))	30	11
6037	(((cp_ent ca_ent)))(ca_wa p_re) ca_wa))(cp_ent oa_ent)(u_isn u_hdip])))(u_isn oa_wa]))	30	11
6038	(((cp_ent ca_ent)))(ca_wa p_re) ca_wa))(p_csn oa_ent) u_hdip])))(u_isn oa_wa) u_hdip]))	26	11
6039	(((cp_ent ca_ent)))(ca_wa p_re) ca_wa))(p_csn oa_ent)(u_isn u_hdip])))(u_isn oa_wa]))	28	11
6040	(((cp_ent ca_ent)))(ca_wa p_re) ca_wa))(p_csn oa_ent)(u_isn u_hdip])))(u_isn u_hdip]))	32	11
6041	(((cp_ent ca_ent)))(ca_wa p_re) cp_ent) oa_ent)(u_isn u_hdip])))(u_isn oa_wa) u_hdip]))	31	11
6042	(((cp_ent ca_ent)))(ca_wa p_re) cp_ent))(oa_ent u_isn) u_hdip])))(u_isn oa_wa) u_hdip]))	33	11
6043	(((cp_ent ca_ent)))(ca_wa p_re)(p_csn oa_ent))(u_isn u_hdip])))(u_isn oa_wa) u_hdip]))	25	11
6044	(((cp_ent ca_wa))(p_re ca_wa))(p_csn oa_ent)(u_isn u_hdip])))(u_isn oa_wa) u_hdip]))	26	11
6045	(((cp_ent ca_wa)))(p_re ca_wa) p_csn))(p_isn oa_ent)(u_isn u_hdip])))(u_isn u_hdip]))	21	11
6046	(((cp_ent ca_wa)))(p_re ca_wa)(p_csn oa_ent))(u_isn u_hdip])))(u_isn oa_wa) u_hdip]))	25	11
6047	(((cp_ent oa_ent) oa_wa))(u_isn u_hdip))(u_isn oa_wa) u_csn])))(oa_ent u_isn) u_hdip]))	18	11
6048	(((oa_ent oa_wa))(u_isn u_hdip))(u_isn u_hdip)(oa_wa (u_csn cp_ent])))(oa_ent oa_wa]))	17	11
6049	(((p_csn ca_ent)))(ca_wa p_re) ca_wa))(cp_ent oa_ent)(u_isn u_hdip])))(u_isn u_hdip]))	27	11
6050	(((p_isn ca_ent)(ca_wa p_re]))(ca_wa (p_csn cp_ent))(oa_ent oa_wa])))(u_isn oa_wa]))	21	11
6051	(((p_isn ca_ent)))(ca_wa p_re) ca_wa))(cp_ent oa_ent)(oa_wa u_hdip])))(u_isn oa_wa]))	26	11
6052	(((p_isn ca_ent)))(ca_wa p_re) ca_wa))(cp_ent oa_ent)(oa_wa u_hdip])))(u_isn u_hdip]))	28	11
6053	(((p_isn ca_ent)))(ca_wa p_re) ca_wa))(p_csn oa_ent)(oa_wa u_hdip])))(u_isn u_hdip]))	23	11
6054	(((p_isn ca_ent))))(ca_wa p_re) cp_ent) oa_ent)(u_isn u_hdip])))(u_isn oa_wa) u_hdip]))	27	11
6055	(((p_isn ca_ent)))(ca_wa p_re)(p_csn oa_ent))(u_isn u_hdip])))(u_isn oa_wa) u_hdip]))	23	11
6056	(((u_csn (cp_ent ca_ent)))(ca_wa p_re))(p_csn oa_ent)(u_isn u_hdip])))(u_isn u_hdip]))	22	11
6057	(((u_csn (cp_ent ca_ent])))(ca_wa p_re) ca_wa) oa_ent])))(oa_wa u_hdip)(u_isn oa_wa]))	18	11
6058	(((u_csn (cp_ent ca_ent])))(ca_wa p_re) ca_wa)(cp_ent oa_ent])))(u_isn oa_wa) u_hdip]))	27	11
6059	(((u_csn (cp_ent ca_ent])))(ca_wa p_re) cp_ent)(oa_ent u_hdip])))(u_isn oa_wa) u_hdip]))	24	11
6060	(((u_csn (cp_ent ca_ent)))(ca_wa p_re) cp_ent))(oa_ent u_isn) u_hdip])))(u_isn u_hdip]))	31	11
6061	((((u_csn cp_ent)(ca_ent ca_wa])))(p_re ca_wa) p_csn)(p_isn oa_ent])))(u_isn u_hdip]))	21	11
6062	(((u_csn cp_ent)))(ca_ent ca_wa) cp_ent)(oa_ent (u_isn u_hdip])))(u_isn oa_wa) u_hdip]))	23	11
6063	(((u_hdip u_hdip))(oa_wa (u_csn cp_ent))(p_csn (ca_ent ca_wa])))(p_re ca_wa) oa_ent]))	17	11
6064	(((u_hdip u_hdip))(u_isn oa_wa))(u_csn cp_ent) p_csn])))(p_isn ca_ent)(ca_wa p_re]))	21	11
6065	(((u_hdip u_hdip)))(u_isn oa_wa) u_csn)(cp_ent ca_ent])))(ca_wa p_re) ca_wa) oa_ent]))	21	11
6066	(((u_hdip u_hdip)))(u_isn oa_wa) u_csn)(cp_ent ca_wa])))(p_re ca_wa)(cp_ent oa_ent]))	18	11
6067	(((u_hdip u_hdip)))(u_isn oa_wa) u_csn)(p_csn (ca_ent ca_wa])))(p_re ca_wa) oa_ent]))	16	11
6068	(((u_isn oa_wa))(u_hdip (u_csn cp_ent])(p_csn ca_ent])))(ca_wa p_re) ca_wa) oa_ent]))	17	11
6069	(((u_isn oa_wa))(u_hdip (u_csn cp_ent])(p_csn (p_isn ca_ent])))(ca_wa p_re) cp_ent]))	19	11
6070	(((u_isn u_hdip)))(u_isn oa_wa) u_csn)(cp_ent ca_ent])))(ca_wa p_re) ca_wa) oa_ent]))	17	11
6071	(((u_isn u_hdip)))(u_isn oa_wa) u_csn)(p_csn (ca_ent ca_wa])))(p_re ca_wa) oa_ent]))	15	11
6072	(((cp_ent oa_ent)(oa_wa u_hdip))(u_isn u_hdip))(u_isn oa_wa) u_csn])))(u_isn cp_ent]))	30	11
6073	(((oa_wa (u_csn (cp_ent ca_ent])))(ca_wa p_re) cp_ent) oa_ent])))(oa_wa u_hdip) oa_wa]))	24	11
6074	(((oa_wa (u_csn (cp_ent ca_ent])))(ca_wa p_re) cp_ent)(oa_ent u_isn])))(oa_wa u_hdip]))	25	11
6075	(((u_csn (cp_ent ca_wa])))(p_re ca_wa)(cp_ent oa_ent))(u_hdip u_hdip])))(u_isn u_hdip]))	24	11
6076	(((u_csn (p_isn ca_ent])))(ca_wa p_re) cp_ent)(oa_ent oa_wa])))(u_isn oa_wa) u_hdip]))	18	11
6077	(((cp_ent oa_ent)(u_isn u_hdip))(u_isn u_hdip)(u_isn oa_wa) u_csn])))(u_isn cp_ent]))	29	11
6078	(((oa_wa (u_csn cp_ent]) ca_ent)))(ca_wa p_re) cp_ent)(oa_ent oa_wa])))(u_isn oa_wa]))	24	11
6079	(((oa_wa (u_csn cp_ent]) ca_ent)))(ca_wa p_re) cp_ent)(oa_ent oa_wa])))(u_isn u_hdip]))	25	11
6080	(((oa_wa (u_csn cp_ent])(ca_ent ca_wa])))(p_re ca_wa) p_csn) oa_ent])(u_hdip u_hdip]))	25	11
6081	(((oa_wa (u_csn cp_ent])(ca_ent ca_wa])))(p_re ca_wa) p_csn) oa_ent])(u_isn u_hdip]))	26	11
6082	(((oa_wa u_hdip)(u_isn oa_wa])))(u_csn cp_ent) p_csn) ca_ent]))(ca_wa p_re) cp_ent]))	23	11
6083	(((p_isn ca_ent)(ca_wa p_re])(p_csn ((p_isn oa_ent)(u_isn u_hdip])))(u_isn u_hdip]))	20	11
6084	(((u_csn cp_ent) p_csn))((ca_wa p_re) cp_ent))(oa_ent u_isn) u_hdip])))(u_isn u_hdip]))	24	11
6085	(((u_hdip oa_wa) u_csn))(cp_ent ca_ent))(ca_wa p_re) cp_ent])))(oa_ent (oa_wa u_hdip]))	20	11
6086	(((u_isn oa_wa) u_hdip)))(u_csn cp_ent) p_csn)(ca_ent ca_wa])))(p_re ca_wa) oa_ent]))	19	11
6087	(((u_isn oa_wa) u_hdip)))(u_csn cp_ent) p_csn) ca_ent])))(ca_wa p_re) ca_wa) oa_ent]))	20	11
6088	(((u_isn oa_wa))(u_csn cp_ent) p_csn)) ca_ent)))(ca_wa p_re) ca_wa)(oa_ent u_isn]))	20	11
6089	(((u_isn oa_wa))(u_csn cp_ent) p_csn]) ca_ent)))(ca_wa p_re) cp_ent)(oa_ent u_isn]))	21	11
6090	(((u_isn oa_wa) u_hdip))((u_csn cp_ent) p_csn)(p_isn ca_ent])))(ca_wa p_re) cp_ent]))	18	11
6091	(((u_isn oa_wa) u_hdip)))(u_isn oa_wa) u_csn)(cp_ent ca_ent])))(ca_wa p_re) cp_ent]))	23	11
6092	(((u_isn u_csn)(cp_ent ca_ent])))(ca_wa p_re) ca_wa)(cp_ent oa_ent])))(u_isn u_hdip]))	25	11
6093	(((p_csn (oa_ent (u_isn u_hdip])))(u_isn oa_wa])))(u_csn cp_ent) p_csn)(ca_ent p_isn]))	26	11
6094	(((p_csn (oa_ent (u_isn u_hdip])))(u_isn oa_wa])))(u_csn cp_ent) p_csn)(ca_ent ca_wa]))	26	11
6095	[illegible]	[illegible]	[illegible]
6096	[illegible]	[illegible]	[illegible]
6097	[illegible]	[illegible]	[illegible]
6098	[illegible]	[illegible]	[illegible]
6099	[illegible]	[illegible]	[illegible]
6100	[illegible]	[illegible]	[illegible]
6101	[illegible]	[illegible]	[illegible]

6102	((((cp_ent ca_ent)((ca_wa p_re)cp_ent)))((oa_ent u_isn)u_hdip)))((u_isn oa_wa)u_hdip))	34	11
6103	(((cp_ent ca_ent)((ca_wa p_re)cp_ent)))((p_isn oa_ent)(u_isn u_hdip)))((u_isn u_hdip))	26	11
6104	((((cp_ent ca_ent)(ca_wa p_re))(ca_wa (p_csn cp_ent)))(oa_ent oa_wa))(u_isn u_hdip))	27	11
6105	((((cp_ent ca_ent)(ca_wa p_re))(ca_wa (p_csn cp_ent)))(oa_ent u_hdip))(u_isn u_hdip))	29	11
6106	((((cp_ent ca_ent)((ca_wa p_re)(p_csn cp_ent)))((oa_ent u_isn)u_hdip))(u_isn u_hdip))	29	11
6107	((((cp_ent ca_ent)((ca_wa p_re)(p_csn oa_ent)))((u_isn u_hdip)))((u_isn oa_wa)u_hdip))	26	11
6108	((((cp_ent ca_ent)((p_re ca_wa)oa_ent)))((oa_wa u_hdip)(u_isn oa_wa)))((u_csn cp_ent))	21	11
6109	((((cp_ent ca_ent)((p_re ca_wa)(p_csn cp_ent)))(oa_ent (u_isn u_hdip)))((u_isn u_hdip))	29	11
6110	((((cp_ent ca_wa)((p_re ca_wa)(p_csn cp_ent)))(oa_ent (u_isn u_hdip)))((u_isn u_hdip))	27	11
6111	((((cp_ent ca_wa)((p_re ca_wa)(p_csn cp_ent)))((oa_ent u_isn)u_hdip))(u_isn u_hdip))	28	11
6112	((((oa_wa u_csn)((ca_wa p_re)ca_wa)))((cp_ent oa_ent)(u_isn u_hdip)))((u_isn u_hdip))	24	11
6113	((((p_isn ca_ent)((ca_wa p_re)(p_csn cp_ent)))(oa_ent (u_isn u_hdip)))((u_isn u_hdip))	24	11
6114	((((p_isn ca_ent)((ca_wa p_re)(p_csn cp_ent)))((oa_ent u_isn)u_hdip))(u_isn u_hdip))	25	11
6115	((((p_isn ca_ent)((p_isn ca_wa)p_csn)))((p_isn oa_ent)(u_isn u_hdip)))((u_isn u_hdip))	19	11
6116	((((p_isn ca_ent)((p_re ca_wa)p_csn)))((p_isn oa_ent)(u_isn u_hdip)))((u_isn u_hdip))	25	11
6117	((((p_isn ca_ent)))((ca_wa p_re)ca_wa)(cp_ent oa_ent)))((u_hdip u_hdip))(u_isn u_hdip))	32	11
6118	((((ca_wa p_re)ca_wa)((cp_ent oa_ent)oa_wa))(u_isn oa_wa))(u_hdip (u_isn u_csn)))	20	11
6119	((((ca_wa p_re)ca_wa)((cp_ent oa_ent)(oa_wa u_hdip)))(u_isn u_hdip))(u_isn u_hdip))	29	11
6120	((((u_csn (cp_ent ca_ent))((ca_wa p_re)ca_wa))(cp_ent oa_ent)))((u_isn oa_wa)u_hdip))	29	11
6121	((((u_csn cp_ent)(ca_ent ca_wa))((p_re ca_wa))((cp_ent oa_ent)u_hdip))(u_isn oa_wa))	27	11
6122	((((u_csn (cp_ent ca_ent))((ca_wa p_re)ca_wa)))((oa_ent u_isn)u_hdip))(u_isn oa_wa))	27	11
6123	((((u_csn (cp_ent ca_ent))((ca_wa p_re)ca_wa)))((oa_ent u_isn)u_hdip))(u_isn u_hdip))	32	11
6124	((((u_csn (cp_ent ca_ent))((ca_wa p_re)ca_wa)))((p_csn oa_ent)u_hdip))(u_isn u_hdip))	27	11
6125	((((u_csn cp_ent)((ca_ent ca_wa)p_re))((p_isn ca_wa)))((cp_ent oa_ent)(u_isn u_hdip))	26	11
6126	((((u_csn cp_ent)((ca_ent ca_wa)p_re))((p_isn ca_wa)))((p_csn oa_ent)(u_isn u_hdip))	22	11
6127	((((u_csn cp_ent)(ca_ent ca_wa))((p_isn p_re)))((cp_ent oa_ent)u_hdip))(u_isn u_hdip))	31	11
6128	((((u_csn cp_ent)((ca_wa p_re)ca_wa))((cp_ent oa_ent)))((oa_wa u_hdip))(u_isn oa_wa))	24	11
6129	((((u_csn cp_ent)((p_isn ca_ent)(ca_wa p_re))))((cp_ent oa_ent)u_hdip))(u_isn u_hdip))	28	11
6130	((((u_csn cp_ent)((p_isn ca_ent)(ca_wa p_re))))((p_isn ca_wa)))((cp_ent oa_ent)u_hdip))	23	11
6131	((((u_hdip u_hdip)(oa_wa (u_csn cp_ent)))((ca_ent ca_wa)))((p_re ca_wa)(cp_ent oa_ent)))	24	11
6132	((((u_hdip u_hdip)(oa_wa (u_csn cp_ent)))ca_ent)))((ca_wa p_re)ca_wa)(oa_ent u_isn)))	20	11
6133	((((u_hdip u_hdip)(oa_wa (u_csn cp_ent)))ca_ent)))((ca_wa p_re)cp_ent)(oa_ent u_isn)))	24	11
6134	((((u_hdip u_hdip)(oa_wa (u_csn cp_ent)))((p_csn ca_ent)))((ca_wa p_re)ca_wa)oa_ent))	17	11
6135	((((u_hdip u_hdip)(oa_wa (u_csn cp_ent)))((p_csn (ca_ent ca_wa)))((p_re ca_wa)oa_ent))	16	11
6136	((((u_isn u_hdip)((u_isn oa_wa)u_csn))((p_csn (ca_ent ca_wa)))((p_re ca_wa)oa_ent))	16	11
6137	((((u_isn oa_wa)((u_csn cp_ent)p_csn))((p_isn ca_ent)))((ca_wa p_re)ca_wa)oa_ent))	15	11
6138	((((u_isn oa_wa)((u_csn cp_ent)p_csn))((p_isn ca_wa)))((p_re ca_wa)(cp_ent oa_ent)))	16	11
6139	((((u_isn u_hdip)((u_isn oa_wa)u_csn))((cp_ent ca_ent)))((ca_wa p_re)ca_wa)oa_ent))	18	11
6140	((((u_isn u_hdip)((u_isn oa_wa)u_csn))((cp_ent ca_ent)))((ca_wa p_re)cp_ent)oa_ent))	21	11
6141	((((oa_ent (oa_wa u_hdip)))((u_isn oa_wa)u_csn))((oa_ent (oa_wa u_hdip)))((u_isn u_hdip))	15	11
6142	((((oa_wa (u_csn cp_ent))p_csn)((p_isn ca_ent)))((ca_wa p_re)ca_wa)(cp_ent oa_ent)))	21	11
6143	((((oa_wa (u_csn cp_ent))p_csn)((p_isn ca_ent)))((ca_wa p_re)cp_ent)(oa_ent u_isn)))	21	11
6144	((((oa_wa (u_csn cp_ent))p_csn)((p_isn ca_ent)))((ca_wa p_re)cp_ent)(oa_ent u_hdip)))	21	11
6145	((((oa_wa (u_csn cp_ent))((p_isn ca_ent)))((ca_wa p_re)ca_wa))((cp_ent oa_ent)u_hdip))	26	11
6146	((((oa_wa (u_csn cp_ent))((p_isn ca_wa))((p_re ca_wa)))((cp_ent oa_ent)(oa_wa u_hdip)))	23	11
6147	((((p_csn (ca_ent ca_wa)))((p_re ca_wa)p_csn))((p_isn oa_ent)u_hdip))(u_isn u_hdip))	22	11
6148	((((p_csn (ca_ent ca_wa)))((p_re ca_wa)p_csn))((oa_ent (oa_wa u_hdip)))((u_isn u_hdip))	23	11
6149	((((p_csn (ca_ent ca_wa)))((p_re ca_wa)p_csn))((oa_ent (u_isn u_hdip)))((u_isn u_hdip))	27	11
6150	((((p_csn (ca_ent ca_wa)))((p_re ca_wa)p_csn))((oa_ent oa_wa)u_hdip))(u_isn u_hdip))	24	11
6151	((((p_csn (ca_ent ca_wa)))((p_re ca_wa)(cp_ent oa_ent)))((u_isn u_hdip))(u_isn u_hdip))	23	11
6152	((((p_csn oa_ent)(u_isn u_hdip))((u_isn oa_wa)))((u_csn cp_ent)p_csn))((ca_wa ca_wa))	19	11
6153	((((p_csn oa_ent)(u_isn u_hdip))((u_isn oa_wa)))((u_csn cp_ent)p_csn))((p_isn ca_wa))	16	11
6154	((((p_csn (p_isn ca_ent)))((p_re ca_wa)(cp_ent oa_ent)))((u_isn u_hdip))(u_isn u_hdip))	17	11
6155	((((u_csn cp_ent)(ca_ent ca_wa))((p_isn ca_wa))((cp_ent oa_ent))(u_hdip (u_isn oa_wa)))	28	11
6156	((((u_csn cp_ent)(ca_ent ca_wa))((p_isn ca_wa))((cp_ent oa_ent)u_hdip))(u_isn oa_wa))	26	11
6157	((((u_csn (ca_ent ca_wa))((p_isn ca_wa))((oa_ent u_isn)))((oa_wa u_hdip))(u_isn oa_wa))	23	11
6158	((((u_csn (ca_ent ca_wa))((p_isn ca_wa))((cp_ent oa_ent)(u_isn u_hdip)))((u_isn oa_wa))	26	11
6159	((((u_csn (ca_ent ca_wa))((p_isn ca_wa))((cp_ent oa_ent)(u_isn u_hdip)))((u_isn u_hdip))	32	11
6160	((((u_csn cp_ent)(ca_ent ca_wa))((p_re p_re)))((p_csn oa_ent)u_hdip))(u_isn u_hdip))	22	11
6161	((((u_csn cp_ent)(ca_ent ca_wa))((p_re p_re)))((p_csn (oa_ent u_hdip)))((u_isn u_hdip))	20	11
6162	((((u_csn (cp_ent ca_wa))((p_re ca_wa)))((cp_ent oa_ent)u_hdip)))((u_isn oa_wa)u_hdip))	23	11
6163	((((u_csn (cp_ent ca_wa))((p_isn ca_wa))((cp_ent oa_ent)(u_isn u_hdip)))((u_isn oa_wa))	26	11
6164	((((u_csn (cp_ent ca_wa))((p_isn ca_wa))((cp_ent oa_ent)(u_isn u_hdip)))((u_isn u_hdip))	26	11
6165	((((u_csn (cp_ent ca_wa))((p_isn ca_wa))((oa_ent u_isn)u_hdip)))((u_isn oa_wa)u_hdip))	23	11
6166	((((u_csn cp_ent)(p_isn ca_ent)))((ca_wa p_re)ca_wa))((cp_ent oa_ent)(u_hdip u_hdip))	28	11
6167	((((u_csn (p_isn ca_ent))((ca_wa p_re))(p_isn ca_wa)))((cp_ent oa_ent)(u_isn u_hdip))	22	11
6168	((((u_csn (p_isn ca_ent))((p_re p_re))(p_csn ((p_isn oa_ent)u_hdip)))((u_isn u_hdip))	18	11
6169	((((u_csn (u_isn cp_ent))((ca_ent ca_wa))(p_re ca_wa)))((cp_ent oa_ent)(u_isn u_hdip))	24	11
6170	((((u_csn (u_isn cp_ent))((p_isn ca_ent)))((ca_wa p_re)ca_wa)))((cp_ent oa_ent)u_hdip))	22	11
6171	((((u_csn (u_isn cp_ent))((p_isn ca_wa))((p_re ca_wa)))((cp_ent oa_ent)(oa_wa u_hdip))	18	11
6172	((((u_csn (u_isn cp_ent))((p_isn ca_wa))((p_re ca_wa)))((cp_ent oa_ent)(u_isn u_hdip))	18	11
6173	((((u_hdip (u_csn cp_ent))((p_isn ca_ent)))((ca_wa p_re)cp_ent))((oa_ent (oa_wa u_hdip)))	16	11
6174	((((u_hdip (u_csn cp_ent))((p_isn ca_wa))((p_isn ca_wa)))((cp_ent oa_ent)(oa_wa u_hdip)))	19	11
6175	((((u_hdip (u_csn cp_ent))((p_isn ca_wa))((p_isn ca_wa)))((p_isn oa_ent)(oa_wa u_hdip)))	18	11
6176	((((u_hdip (u_isn oa_wa))u_csn)((ca_ent ca_wa)))((p_re ca_wa)((p_csn oa_ent)u_hdip))	19	11
6177	((((u_hdip (u_isn oa_wa))u_csn)((ca_ent ca_wa)))((p_re ca_wa)(p_csn (p_isn oa_ent))))	17	11
6178	((((u_hdip (u_isn oa_wa))u_csn)((ca_ent ca_wa)))((p_re ca_wa)((cp_ent oa_ent)oa_wa)))	21	11
6179	((((u_hdip (u_isn oa_wa))u_csn)((cp_ent ca_ent)))((ca_wa p_re)ca_wa)(oa_ent u_isn)))	22	11
6180	((((u_hdip (u_isn oa_wa))u_csn)((cp_ent ca_ent)))((ca_wa p_re)ca_wa)(p_csn oa_ent)))	20	11
6181	((((u_hdip (u_isn oa_wa))u_csn)((cp_ent ca_ent)))((ca_wa p_re)cp_ent)(oa_ent u_isn)))	25	11
6182	((((u_hdip (u_isn oa_wa))u_csn)((cp_ent ca_ent)))((ca_wa p_re)cp_ent)(oa_ent oa_wa)))	20	11
6183	((((u_hdip (u_isn oa_wa))u_csn)((cp_ent ca_ent)))((ca_wa p_re)cp_ent)(oa_ent u_hdip)))	25	11
6184	((((u_hdip (u_isn oa_wa))u_csn)((ca_ent ca_wa)))((p_re ca_wa)oa_ent)(u_isn u_hdip)))	19	11
6185	((((u_hdip (u_isn oa_wa))u_csn)((ca_ent ca_wa)))((p_re ca_wa)p_csn)(oa_ent u_isn)))	21	11
6186	((((u_hdip (u_isn oa_wa))u_csn)((ca_ent ca_wa)))((p_re ca_wa)p_csn)(oa_ent u_hdip)))	21	11
6187	((((u_hdip (u_isn oa_wa))u_csn)((ca_ent ca_wa)))((p_re ca_wa)p_csn)(p_isn oa_ent)))	18	11
6188	((((u_hdip (u_isn oa_wa))u_csn)((cp_ent ca_wa)))((p_re ca_wa)((cp_ent oa_ent)oa_wa)))	20	11
6189	((((u_hdip (u_isn oa_wa))u_csn)((cp_ent ca_wa)))((p_re ca_wa)((cp_ent oa_ent)u_hdip)))	21	11
6190	((((u_hdip (u_isn oa_wa))u_csn)((cp_ent ca_ent)((ca_wa p_re)cp_ent)))((oa_ent u_hdip))	24	11
6191	((((u_hdip (u_isn oa_wa))u_csn)((cp_ent ca_ent)((ca_wa p_re)cp_ent)))((oa_ent u_isn))	24	11
6192	((((u_hdip (u_isn u_csn))(cp_ent ca_ent))((p_isn ca_wa)))((cp_ent oa_ent)(oa_wa u_hdip)))	21	11
6193	((((u_hdip (u_isn u_csn))(cp_ent ca_ent)))((ca_wa p_re)ca_wa)))((cp_ent oa_ent)u_hdip))	26	11
6194	((((u_hdip (u_isn u_csn))(cp_ent ca_ent)))((ca_wa p_re)cp_ent))((oa_ent (oa_wa u_hdip)))	18	11
6195	((((u_hdip (u_isn u_csn))(cp_ent ca_wa))((p_isn ca_wa)))((cp_ent oa_ent)(oa_wa u_hdip)))	19	11
6196	(((ca_ent p_isn)))((p_re ca_wa)((cp_ent oa_ent)u_hdip))(u_isn u_hdip)))((u_isn u_hdip))	37	11
6197	((((ca_ent ca_wa)cp_ent))((p_isn oa_ent)(u_isn u_hdip)))((u_isn u_hdip))(u_isn u_hdip))	24	11
6198	(((ca_ent p_isn)))((p_re ca_wa)p_csn)(cp_ent oa_ent))(u_isn u_hdip)))((u_isn u_hdip))	31	11
6199	(((ca_ent p_isn)))((p_re ca_wa)p_csn)(p_isn oa_ent))(u_isn u_hdip)))((u_isn u_hdip))	25	11
6200	((((ca_wa p_re)ca_wa)((cp_ent oa_ent)(u_isn u_hdip)))((u_isn u_hdip))(u_isn oa_wa))	29	11
6201	((((ca_wa p_re)ca_wa)((p_csn oa_ent)(u_isn u_hdip)))((u_isn oa_wa))(u_isn oa_wa))	21	11
6202	((((ca_wa p_re)ca_wa)((p_csn oa_ent)(u_isn u_hdip)))((u_isn oa_wa))(u_isn u_hdip))	22	11
6203	(((cp_ent ca_ent)))((ca_wa p_re)ca_wa)(p_csn oa_ent))(u_hdip u_hdip)))((u_isn u_hdip))	28	11
6204	((((cp_ent ca_ent)(ca_wa p_re))(p_csn (oa_ent u_isn)))((u_hdip u_hdip))(u_isn u_hdip))	27	11
6205	(((((oa_ent oa_wa)u_csn)(u_isn cp_ent)))(ca_ent ca_wa))(p_isn p_re))(ca_wa p_csn))	19	11
6206	((((oa_wa u_hdip)oa_wa)(u_csn (cp_ent ca_ent)))((ca_wa p_re)cp_ent))(oa_ent u_isn))	23	11
6207	((((p_csn oa_ent)(u_isn u_hdip))((u_isn oa_wa))(u_csn cp_ent))(p_csn (ca_ent ca_wa)))	24	11
6208	((((p_csn oa_ent)(u_isn u_hdip))((u_isn u_hdip))(u_isn oa_wa)))((u_csn cp_ent)p_csn))	28	11
6209	((((p_isn ca_ent)(ca_wa p_re))(p_csn (p_isn oa_ent)))((u_isn u_hdip))(u_isn u_hdip))	23	11
6210	((((u_csn cp_ent)(ca_ent ca_wa))((p_isn ca_wa)))((oa_ent u_isn)u_hdip))(u_isn oa_wa))	26	11
6211	((((u_csn cp_ent)(ca_wa ca_wa))((cp_ent oa_ent)u_hdip))(u_isn u_hdip))(u_isn u_hdip))	22	11
6212	((((u_csn cp_ent)(ca_wa p_re))(p_isn ca_wa)))((cp_ent oa_ent)u_hdip))(u_isn u_hdip))	23	11
6213	((((u_csn cp_ent)(ca_wa p_re))(p_isn ca_wa)))((p_csn oa_ent)u_hdip))(u_isn u_hdip))	20	11
6214	((((u_csn cp_ent)(p_isn ca_ent))((ca_wa p_re))(p_isn ca_wa)))((cp_ent oa_ent)u_hdip))	24	11
6215	((((u_csn cp_ent)(p_isn ca_ent))((ca_wa p_re))(p_isn ca_wa)))((oa_ent u_isn)u_hdip))	24	11
6216	((((u_csn cp_ent)(p_isn ca_ent))((p_re p_re)))((p_csn oa_ent)u_hdip))(u_isn u_hdip))	21	11
6217	((((u_csn cp_ent)(p_isn ca_wa))((p_isn ca_wa)))((cp_ent oa_ent)oa_wa))(u_isn oa_wa))	22	11
6218	((((u_csn cp_ent)(p_isn ca_wa))((p_isn ca_wa)))((cp_ent oa_ent)oa_wa))(u_isn u_hdip))	23	11
6219	((((u_csn cp_ent)(p_isn ca_wa))((p_isn ca_wa)))((cp_ent oa_ent)u_hdip))(u_isn oa_wa))	24	11
6220	((((u_csn cp_ent)(p_isn ca_wa))((p_isn ca_wa)))((cp_ent oa_ent)u_hdip))(u_isn u_hdip))	24	11
6221	((((u_csn cp_ent)(p_isn ca_wa))((p_isn ca_wa)))((oa_ent u_isn)u_hdip))(u_isn oa_wa))	24	11
6222	((((u_csn cp_ent)(p_isn ca_wa))((p_isn ca_wa)))((oa_ent u_isn)u_hdip))(u_isn u_hdip))	24	11
6223	((((u_csn cp_ent)(p_isn ca_wa))((p_isn p_re)))((cp_ent oa_ent)u_hdip))(u_isn oa_wa))	26	11
6224	((((u_csn cp_ent)(p_isn ca_wa))((p_isn p_re)))((cp_ent oa_ent)u_hdip))(u_isn u_hdip))	25	11
6225	((((u_csn cp_ent)(p_isn ca_wa))((p_re ca_wa)))((cp_ent oa_ent))(u_hdip (u_isn oa_wa)))	26	11
6226	((((u_csn cp_ent)(p_isn ca_wa))((p_re ca_wa)))((cp_ent oa_ent))((u_isn oa_wa)u_hdip))	26	11
6227	((((u_csn cp_ent)(p_isn ca_wa))((p_re ca_wa)))((cp_ent oa_ent)oa_wa))(u_isn oa_wa))	26	11
6228	((((u_csn cp_ent)(p_isn ca_wa))((p_re ca_wa)))((cp_ent oa_ent)oa_wa))(u_isn u_hdip))	26	11
6229	((((u_csn cp_ent)(p_isn ca_wa))((p_re ca_wa)))((cp_ent oa_ent)u_hdip))(u_isn oa_wa))	27	11
6230	((((u_csn cp_ent)(p_isn ca_wa))((p_re ca_wa)))((cp_ent oa_ent)u_hdip))(u_isn u_hdip))	26	11
6231	((((u_hdip oa_wa)u_csn)(cp_ent ca_wa)))((p_re ca_wa)(cp_ent oa_ent)))((oa_wa u_hdip))	22	11
6232	((((u_hdip oa_wa)u_csn)((cp_ent ca_wa)cp_ent))(oa_ent (oa_wa u_hdip)))((u_isn u_hdip))	17	11
6233	((((u_hdip u_csn)(ca_ent ca_wa))((p_isn ca_wa))((cp_ent oa_ent)u_hdip))(u_isn oa_wa))	22	11
6234	((((u_hdip u_csn)(ca_ent ca_wa))((p_isn ca_wa))((cp_ent oa_ent)u_hdip))(u_isn u_hdip))	22	11
6235	((((u_hdip u_csn)(ca_ent ca_wa))((p_isn ca_wa))((oa_ent u_isn)u_hdip))(u_isn oa_wa))	22	11
6236	((((u_hdip u_csn)(ca_ent ca_wa))((p_isn ca_wa))((oa_ent u_isn)u_hdip))(u_isn u_hdip))	22	11
6237	((((u_hdip u_csn)(cp_ent ca_ent)))((ca_wa p_re)ca_wa))(cp_ent oa_ent)))(u_hdip u_hdip))	27	11
6238	((((u_isn cp_ent)(p_isn ca_ent)))((ca_wa p_re)ca_wa))(p_csn cp_ent))(oa_ent u_isn))	15	11
6239	((((u_isn oa_wa)u_csn)(cp_ent ca_wa)))((p_re ca_wa)(cp_ent oa_ent)))((oa_wa u_hdip))	21	11
6240	((((u_isn oa_wa)u_hdip)(oa_wa u_csn))((cp_ent ca_ent)))((ca_wa p_re)cp_ent)oa_ent))	20	11
6241	((((u_isn u_csn)(cp_ent ca_ent)))((ca_wa p_re)cp_ent))(oa_ent oa_wa))(u_isn u_hdip))	19	11
6242	(p_csn ((ca_ent ca_wa)))((p_re ca_wa)(cp_ent oa_ent)))(u_isn u_hdip))(u_isn u_hdip)))	26	11
6243	(p_csn ((ca_ent p_isn)))((p_re ca_wa)(cp_ent oa_ent)))(u_isn u_hdip))(u_isn u_hdip)))	25	11
6244	((p_csn ((p_isn ca_ent)))((ca_wa p_re)cp_ent))(oa_ent u_isn)u_hdip)))(u_isn u_hdip))	24	11
6245	(p_csn ((p_isn ca_ent)))((ca_wa p_re)cp_ent)(oa_ent (u_isn u_hdip)))(u_isn u_hdip)))	22	11
6246	(p_csn ((p_isn ca_ent)))((p_re ca_wa)(cp_ent oa_ent)))(u_isn u_hdip))(u_isn u_hdip)))	21	11
6247	(p_csn ((((p_isn ca_ent))((p_re ca_wa)(cp_ent oa_ent)))(u_isn u_hdip))(u_isn u_hdip))	22	11
6248	((ca_ent ca_wa)((p_isn p_re)))((ca_wa (p_csn cp_ent))(oa_ent u_isn)))(u_hdip u_hdip)))	32	11
6249	((ca_ent ca_wa)((p_re ca_wa))(p_csn ((p_isn oa_ent)(u_isn u_hdip)))(u_isn u_hdip))))	23	11
6250	((ca_ent ca_wa)((p_re ca_wa)))((p_csn cp_ent))(oa_ent u_isn)u_hdip))(u_isn u_hdip)))	31	11
6251	((ca_ent ca_wa)))((p_re (ca_wa p_csn))(cp_ent oa_ent))(u_isn u_hdip))(u_isn u_hdip))	31	11
6252	((ca_ent ca_wa)))((p_re ca_wa)oa_ent)(oa_wa u_hdip))(u_isn oa_wa))(u_csn cp_ent)))	21	11
6253	((ca_ent ca_wa)))((p_re ca_wa)(p_csn (oa_ent u_isn)))(u_hdip u_hdip))(u_isn u_hdip))	32	11
6254	((ca_ent ca_wa)))((p_re ca_wa)((cp_ent oa_ent)oa_wa))(u_hdip u_hdip))(u_isn u_hdip)))	29	11
6255	((ca_ent ca_wa)))((p_re ca_wa)((cp_ent oa_ent)oa_wa))(u_isn u_hdip))(u_isn u_hdip)))	34	11
6256	((ca_ent ca_wa)))((p_re ca_wa)((cp_ent oa_ent)u_hdip))(u_isn u_hdip))(u_isn oa_wa)))	34	11
6257	((ca_ent ca_wa)))((p_re ca_wa)(cp_ent oa_ent)u_hdip))(u_isn u_hdip))(u_isn u_hdip)))	38	11
6258	((ca_ent ca_wa)))((p_re ca_wa)((p_csn oa_ent)u_hdip))(u_isn u_hdip))(u_isn u_hdip))	32	11
6259	((ca_ent ca_wa)))((p_isn ca_wa)p_csn)(oa_ent oa_wa))(u_isn u_hdip))(u_isn u_hdip)))	23	11
6260	((ca_ent ca_wa)))((p_isn ca_wa)p_csn)(p_isn oa_ent))(u_isn u_hdip))(u_isn u_hdip))	20	11
6261	((ca_ent ca_wa)))((p_re ca_wa)oa_ent)(oa_wa u_hdip))(u_isn u_hdip))(oa_wa u_csn)))	25	11
6262	((ca_ent ca_wa)))((p_re ca_wa)oa_ent)(oa_wa u_hdip))(u_isn u_hdip))(u_isn u_hdip)))	24	11
6263	((ca_ent ca_wa)))((p_re ca_wa)oa_ent)(u_hdip u_hdip))(u_isn u_hdip))(oa_wa u_csn)))	27	11
6264	((ca_ent ca_wa)))((p_re ca_wa)p_csn)oa_ent)(u_isn u_hdip)))((u_isn oa_wa)u_hdip)))	32	11
6265	((ca_ent ca_wa)))((p_re ca_wa)p_csn)(cp_ent oa_ent))(u_isn u_hdip))(u_isn u_hdip)))	32	11
6266	((ca_ent ca_wa)))((p_re ca_wa)p_csn)(oa_ent u_isn))(u_hdip u_hdip))(u_isn u_hdip)))	33	11
6267	((ca_ent p_isn))((p_re ca_wa)((p_csn ((p_isn oa_ent)(u_isn u_hdip)))((u_isn u_hdip))))	21	11
6268	((ca_ent p_isn)))((p_re ca_wa)p_csn)(p_isn oa_ent))(u_isn u_hdip))(u_isn u_hdip)))	23	11
6269	((ca_ent p_isn)))((p_re ca_wa)(p_csn (oa_ent u_isn)))(u_hdip u_hdip))(u_isn u_hdip)))	28	11
6270	((ca_ent p_isn)))((p_re ca_wa)((cp_ent oa_ent)oa_wa))(u_hdip u_hdip))(u_isn u_hdip)))	26	11
6271	((ca_ent p_isn)))((p_re ca_wa)((cp_ent oa_ent)oa_wa))(u_isn u_hdip))(u_isn u_hdip)))	33	11
6272	((ca_ent p_isn)))((p_re ca_wa)((p_csn oa_ent)u_hdip))(u_isn u_hdip))(u_isn u_hdip)))	31	11
6273	((ca_ent p_isn)))((p_re ca_wa)p_csn)oa_ent)(u_isn u_hdip)))((u_isn oa_wa)u_hdip)))	28	11
6274	((ca_ent p_isn)))((p_re ca_wa)p_csn)(oa_ent u_isn))(u_hdip u_hdip))(u_isn u_hdip)))	29	11
6275	((ca_wa p_re)))((ca_wa p_csn)oa_ent)(u_hdip u_hdip))(u_isn u_hdip))(u_isn u_hdip)))	21	11
6276	((cp_ent ca_ent)))((ca_wa p_re)ca_wa)(p_csn (oa_ent (u_isn u_hdip)))(u_isn oa_wa)))	23	11
6277	((cp_ent ca_ent)))((ca_wa p_re)ca_wa)(p_csn (oa_ent (u_isn u_hdip)))(u_isn u_hdip)))	28	11
6278	((cp_ent ca_ent)))((ca_wa p_re)ca_wa)(p_csn oa_ent))(u_isn u_hdip))(u_isn u_hdip)))	33	11
6279	((cp_ent ca_ent)))((ca_wa ca_wa)(p_csn (oa_ent u_isn))(u_hdip u_hdip)))(u_isn u_hdip)))	24	11
6280	((cp_ent ca_ent)))((ca_wa p_re)ca_wa)(p_csn (oa_ent (oa_wa u_hdip)))((u_isn u_hdip)))	23	11
6281	((cp_ent ca_ent)))((ca_wa p_re)ca_wa)(p_csn ((oa_ent oa_wa)u_hdip)))(u_isn u_hdip)))	23	11
6282	((cp_ent ca_ent)))((ca_wa p_re)ca_wa)(p_csn ((p_isn oa_ent)u_hdip)))(u_isn u_hdip)))	22	11
6283	((cp_ent ca_ent)))((ca_wa p_re)ca_wa)(p_csn (oa_ent (oa_wa u_hdip)))(u_isn u_hdip)))	22	11
6284	((cp_ent ca_ent)))((p_re ca_wa)((cp_ent oa_ent)oa_wa))(u_isn u_hdip))(u_isn u_hdip)))	29	11
6285	((cp_ent ca_ent)))((p_re ca_wa)((cp_ent oa_ent)u_hdip))(u_isn u_hdip))(u_isn u_hdip)))	35	11
6286	((cp_ent ca_ent)))((ca_wa p_re)ca_wa)(cp_ent oa_ent))(oa_wa u_hdip))(u_isn u_hdip)))	30	11
6287	((cp_ent ca_ent)))((ca_wa p_re)ca_wa)(cp_ent oa_ent))(u_hdip u_hdip))(u_isn oa_wa)))	28	11
6288	((cp_ent ca_ent)))((ca_wa p_re)ca_wa)(cp_ent oa_ent))(u_hdip u_hdip))(u_isn u_hdip)))	34	11
6289	((cp_ent ca_ent)))((ca_wa p_re)ca_wa)(cp_ent oa_ent))(u_isn oa_wa))(u_isn oa_wa)))	32	11
6290	((cp_ent ca_ent)))((ca_wa p_re)ca_wa)(cp_ent oa_ent))(u_isn oa_wa))(u_isn u_hdip)))	30	11
6291	((cp_ent ca_ent)))((ca_wa p_re)ca_wa)(cp_ent oa_ent))(u_isn u_hdip))(oa_wa u_hdip)))	30	11
6292	((cp_ent ca_ent)))((ca_wa p_re)ca_wa)(cp_ent oa_ent))(u_isn u_hdip))(u_isn oa_wa)))	34	11
6293	((cp_ent ca_ent)))((ca_wa p_re)ca_wa)(cp_ent oa_ent))(u_isn u_hdip))(u_isn u_hdip)))	37	11
6294	((cp_ent ca_ent)))((p_isn ca_wa)p_csn)(oa_ent oa_wa))(u_isn u_hdip))(u_isn u_hdip)))	21	11
6295	((cp_ent ca_ent)))((p_isn ca_wa)p_csn)(p_isn oa_ent))(u_isn u_hdip))(u_isn u_hdip)))	20	11
6296	((cp_ent ca_ent)))((p_re ca_wa)p_csn)oa_ent)(u_isn u_hdip)))((u_isn oa_wa)u_hdip)))	26	11
6297	((cp_ent ca_ent)))((p_re ca_wa)p_csn)(cp_ent oa_ent))(u_isn u_hdip))(u_isn u_hdip)))	29	11
6298	((cp_ent ca_ent)))((p_re ca_wa)p_csn)(oa_ent u_isn))(u_hdip u_hdip))(u_isn u_hdip)))	27	11
6299	((cp_ent ca_wa)))((p_re ca_wa)((cp_ent oa_ent)u_hdip))(u_isn u_hdip))(u_isn u_hdip)))	33	11
6300	((oa_wa u_hdip)))((u_isn oa_wa)((u_csn cp_ent)p_csn))ca_ent))(ca_wa p_re)cp_ent)))	17	11
6301	((p_csn ca_ent)))((ca_wa p_re)ca_wa)(cp_ent oa_ent))(u_isn u_hdip))(u_isn oa_wa)))	23	11
6302	((p_csn ca_ent)))((ca_wa p_re)ca_wa)(cp_ent oa_ent))(u_isn u_hdip))(u_isn u_hdip)))	29	11
6303	((p_isn ca_ent)))((ca_wa p_re)ca_wa)((p_csn (oa_ent (u_isn u_hdip)))(u_isn oa_wa)))	19	11
6304	((p_isn ca_ent)))((ca_wa p_re)ca_wa)((p_csn (oa_ent (u_isn u_hdip)))(u_isn u_hdip)))	26	11
6305	((p_isn ca_ent)))((ca_wa p_re)(ca_wa (p_csn cp_ent)))(oa_ent oa_wa))(u_isn u_hdip)))	20	11
6306	((p_isn ca_ent)))((ca_wa p_re)ca_wa)(p_csn oa_ent))(u_isn u_hdip))(oa_wa u_hdip)))	25	11
6307	((p_isn ca_ent)))((ca_wa p_re)ca_wa)(p_csn oa_ent))(u_isn u_hdip))(u_isn oa_wa)))	26	11
6308	((p_isn ca_ent)))((ca_wa p_re)(p_csn ((p_isn oa_ent)(u_isn u_hdip)))(u_isn u_hdip)))	20	11
6309	((p_isn ca_ent)))((p_isn p_re))(ca_wa (p_csn cp_ent)))(oa_ent oa_wa))(u_isn oa_wa)))	21	11
6310	((p_isn ca_ent)))((ca_wa p_re)ca_wa)(p_csn (oa_ent (oa_wa u_hdip)))((u_isn u_hdip)))	19	11
6311	((p_isn ca_ent)))((ca_wa p_re)ca_wa)(p_csn ((oa_ent oa_wa)u_hdip)))(u_isn u_hdip)))	19	11
6312	((p_isn ca_ent)))((ca_wa p_re)ca_wa)(p_csn ((p_isn oa_ent)u_hdip)))(u_isn u_hdip)))	23	11
6313	((p_isn ca_ent)))((ca_wa p_re)ca_wa)(p_csn (oa_ent (oa_wa u_hdip)))(u_isn u_hdip)))	18	11
6314	((p_isn ca_ent)))((p_re ca_wa)(p_csn (oa_ent u_isn)))(u_hdip u_hdip))(u_isn u_hdip)))	23	11
6315	((p_isn ca_ent)))((p_re ca_wa)((cp_ent oa_ent)oa_wa))(u_hdip u_hdip))(u_isn u_hdip)))	19	11
6316	((p_isn ca_ent)))((ca_wa p_re)ca_wa)(cp_ent oa_ent))(oa_wa u_hdip))(u_isn u_hdip)))	28	11
6317	((p_isn ca_ent)))((ca_wa p_re)ca_wa)(cp_ent oa_ent))(u_hdip u_hdip))(u_isn oa_wa)))	26	11
6318	((p_isn ca_ent)))((ca_wa p_re)ca_wa)(cp_ent oa_ent))(u_isn oa_wa))(u_isn oa_wa)))	29	11
6319	((p_isn ca_ent)))((ca_wa p_re)ca_wa)(cp_ent oa_ent))(u_isn oa_wa))(u_isn u_hdip)))	29	11
6320	((p_isn ca_ent)))((ca_wa p_re)ca_wa)(cp_ent oa_ent))(u_isn u_hdip))(oa_wa u_hdip)))	29	11
6321	((p_isn ca_ent)))((ca_wa p_re)ca_wa)(cp_ent oa_ent))(u_isn u_hdip))(u_isn oa_wa)))	31	11
6322	((p_isn ca_ent)))((ca_wa p_re)ca_wa)(cp_ent oa_ent))(u_isn u_hdip))(u_isn u_hdip)))	34	11
6323	((p_isn ca_ent)))((ca_wa p_re)ca_wa)(oa_ent u_isn))(u_hdip u_hdip))(u_isn u_hdip)))	27	11
6324	((p_isn ca_ent)))((ca_wa p_re)cp_ent)(oa_ent u_hdip))(u_isn u_hdip))(u_isn u_hdip)))	30	11
6325	((p_isn ca_ent)))((ca_wa p_re)cp_ent)(oa_ent oa_wa))(u_isn u_hdip))(u_isn u_hdip)))	25	11
6326	((p_isn ca_ent)))((ca_wa p_re)cp_ent)(oa_ent u_hdip))(u_isn oa_wa))(u_isn oa_wa)))	27	11
6327	((p_isn ca_ent)))((ca_wa p_re)cp_ent)(oa_ent u_hdip))(u_isn oa_wa))(u_isn u_hdip)))	28	11
6328	((p_isn ca_ent)))((p_re ca_wa)p_csn)oa_ent)(u_isn u_hdip)))((u_isn oa_wa)u_hdip)))	23	11
6329	((p_isn ca_ent)))((p_re ca_wa)p_csn)(cp_ent oa_ent))(u_isn u_hdip))(u_isn u_hdip)))	25	11
6330	((p_isn ca_wa)((p_isn p_re)))((ca_wa p_csn)oa_ent)(u_isn u_hdip))(u_isn u_hdip))))	28	11
6331	((p_isn ca_wa)((p_re ca_wa))(p_csn ((p_isn oa_ent)(u_isn u_hdip)))(u_isn u_hdip))))	23	11
6332	((p_isn ca_wa)))((p_re ca_wa)p_csn)(p_isn oa_ent))(u_isn u_hdip))(u_isn u_hdip)))	25	11
6333	((p_isn ca_wa)))((p_re ca_wa)(p_csn (oa_ent u_isn)))(u_hdip u_hdip))(u_isn u_hdip)))	31	11
6334	((p_isn ca_wa)))((p_re ca_wa)((cp_ent oa_ent)oa_wa))(u_hdip u_hdip))(u_isn u_hdip)))	28	11
6335	((p_isn ca_wa)))((p_re ca_wa)((cp_ent oa_ent)oa_wa))(u_isn u_hdip))(u_isn u_hdip)))	32	11
6336	((p_isn ca_wa)))((p_re ca_wa)((cp_ent oa_ent)u_hdip))(u_isn u_hdip))(u_isn oa_wa)))	32	11
6337	((p_isn ca_wa)))((p_re ca_wa)((cp_ent oa_ent)u_hdip))(u_isn u_hdip))(u_isn u_hdip)))	36	11
6338	((p_isn ca_wa)))((p_re ca_wa)((p_csn oa_ent)u_hdip))(u_isn u_hdip))(u_isn u_hdip)))	30	11
6339	((p_isn ca_wa)))((p_re ca_wa)oa_ent)(oa_wa u_hdip))(u_isn u_hdip))(oa_wa u_csn)))	24	11
6340	((p_isn ca_wa)))((p_re ca_wa)oa_ent)(oa_wa u_hdip))(u_isn u_hdip))(u_isn u_hdip)))	23	11
6341	((p_isn ca_wa)))((p_re ca_wa)oa_ent)(u_hdip u_hdip))(u_isn u_hdip))(oa_wa u_csn)))	26	11
6342	((p_isn ca_wa)))((p_re ca_wa)p_csn)(oa_ent u_isn))(u_hdip u_hdip))(u_isn u_hdip)))	32	11
6343	((p_isn oa_ent)(u_hdip u_hdip))((u_isn u_hdip))(u_isn u_hdip))(u_isn oa_wa)u_csn))))	20	11
6344	((p_isn oa_ent)(u_isn u_hdip))((u_isn u_hdip))(u_isn u_hdip))(u_isn oa_wa)u_csn))))	22	11
6345	((u_csn (cp_ent ca_ent)))((ca_wa p_re)((p_csn (p_isn oa_ent))u_hdip))(u_isn u_hdip)))	19	11
6346	((u_csn (cp_ent ca_ent)))((ca_wa p_re)ca_wa)oa_ent)(u_hdip u_hdip))(u_isn u_hdip)))	24	11
6347	((u_csn (cp_ent ca_ent)))((ca_wa p_re)ca_wa)oa_ent)(u_isn u_hdip))(u_isn oa_wa)))	21	11
6348	((u_csn (cp_ent ca_ent)))((ca_wa p_re)ca_wa)oa_ent)(u_isn u_hdip))(u_isn u_hdip)))	25	11
6349	((u_csn (cp_ent ca_ent)))((ca_wa p_re)cp_ent)oa_ent)(u_isn oa_wa))(u_isn oa_wa)))	24	11
6350	((u_csn (cp_ent ca_ent)))((ca_wa p_re)cp_ent)oa_ent)(u_isn u_hdip))(oa_wa u_hdip)))	22	11
6351	((u_csn (cp_ent ca_ent)))((ca_wa p_re)cp_ent)oa_ent)(u_isn u_hdip))(u_isn oa_wa)))	28	11
6352	((u_hdip u_hdip)))((u_isn oa_wa)((u_csn cp_ent)p_csn))(ca_ent p_isn))(p_re ca_wa)))	25	11
6353	((u_hdip u_hdip)))((u_isn oa_wa)((u_csn cp_ent)p_csn))ca_ent))(ca_wa p_re)ca_wa)))	25	11
6354	((u_hdip u_hdip)))((u_isn oa_wa)((u_csn cp_ent)p_csn))ca_ent))(ca_wa p_re)cp_ent)))	25	11
6355	((u_isn u_hdip)((u_isn u_hdip))(u_isn u_hdip))(u_isn u_hdip))(u_isn oa_wa)u_csn))))	25	11
6356	((u_isn u_hdip)))((u_isn oa_wa)((u_csn cp_ent)p_csn))(ca_ent p_isn))(p_re ca_wa)))	25	11
6357	((u_isn u_hdip)))((u_isn oa_wa)((u_csn cp_ent)p_csn))ca_ent))(ca_wa p_re)ca_wa)))	25	11
6358	((u_isn u_hdip)))((u_isn oa_wa)((u_csn cp_ent)p_csn))ca_ent))(ca_wa p_re)cp_ent)))	25	11
6359	((u_csn (p_isn ca_ent)))((ca_wa p_re)ca_wa)oa_ent)(u_hdip u_hdip))(u_isn u_hdip)))	20	11
6360	((u_csn (p_isn ca_ent)))((ca_wa p_re)ca_wa)oa_ent)(u_isn u_hdip))(u_isn u_hdip)))	21	11
6361	((u_csn (p_isn ca_ent)))((ca_wa p_re)cp_ent)oa_ent)(u_isn oa_wa))(u_isn oa_wa)))	19	11
6362	((u_csn (p_isn ca_ent)))((ca_wa p_re)cp_ent)oa_ent)(u_isn u_hdip))(u_isn oa_wa)))	22	11
6363	((u_csn (p_isn ca_ent)))((ca_wa p_re)cp_ent)oa_ent)(u_isn u_hdip))(u_isn u_hdip)))	26	11
6364	(((ca_ent ca_wa)cp_ent)))((p_csn (oa_ent (u_isn u_hdip)))(u_isn u_hdip))(u_isn oa_wa)))	22	11
6365	(((u_isn cp_ent)ca_ent)))((ca_wa p_re)cp_ent)oa_ent)(u_isn oa_wa))(u_isn u_hdip)))	18	11
6366	(((u_isn cp_ent)ca_ent)))((ca_wa p_re)cp_ent)oa_ent)(u_isn u_hdip))(u_isn u_hdip)))	22	11
6367	(((u_isn oa_wa)u_hdip)))((u_isn oa_wa)((u_csn cp_ent)p_csn))ca_ent)(ca_wa ca_wa)))	17	11
6368	(((u_isn oa_wa)u_hdip)))((u_isn oa_wa)((u_csn cp_ent)p_csn))ca_ent)(ca_wa p_re)))	17	11
6369	(((ca_ent ca_wa)((p_re ca_wa))(p_csn cp_ent))(oa_ent (u_isn u_hdip)))((u_isn u_hdip))	30	11
6370	((((p_isn p_re))((ca_wa p_csn)oa_ent)(u_hdip u_hdip)))((u_isn u_hdip))(u_isn u_hdip))	20	11
6371	(((ca_ent ca_wa)))((p_re ca_wa)oa_ent)(oa_wa u_hdip))(u_isn u_hdip)))((u_isn u_hdip))	25	11
6372	(((ca_ent ca_wa)))((p_re ca_wa)oa_ent)(u_hdip u_hdip))(u_isn u_hdip)))((oa_wa u_csn))	28	11
6373	(((((ca_wa p_re)ca_wa)(cp_ent oa_ent)))(u_hdip u_hdip))(u_isn u_hdip))(oa_wa u_csn))	32	11
6374	(((((ca_wa p_re)ca_wa)(cp_ent oa_ent)))(u_hdip u_hdip))(u_isn u_hdip))(u_isn oa_wa))	32	11
6375	(((((ca_wa p_re)ca_wa)(cp_ent oa_ent)))(u_hdip u_hdip))(u_isn u_hdip))(u_isn u_hdip))	32	11
6376	(((((ca_wa p_re)ca_wa)(p_csn oa_ent)))(u_hdip u_hdip))(u_isn u_hdip))(u_isn u_hdip))	25	11
6377	(((((ca_wa p_re)ca_wa)(p_csn oa_ent)))(u_isn u_hdip))(u_isn oa_wa))(u_isn u_hdip))	29	11
6378	(((ca_ent p_isn))((p_re ca_wa))(p_csn (oa_ent u_isn))(u_hdip u_hdip)))((u_isn u_hdip))	30	11
6379	(((ca_ent p_isn)))((p_re ca_wa)(p_csn (oa_ent u_isn)))(u_hdip u_hdip)))((u_isn u_hdip))	31	11
6380	(((ca_ent p_isn)))((p_re ca_wa)((cp_ent oa_ent)oa_wa))(u_hdip u_hdip)))((u_isn u_hdip))	30	11
6381	(((ca_ent p_isn)))((p_re ca_wa)p_csn)(oa_ent u_isn))(u_hdip u_hdip)))((u_isn u_hdip))	32	11
6382	(((ca_ent p_isn)))((p_re ca_wa)p_csn)(cp_ent oa_ent))(u_isn u_hdip)))((u_isn oa_wa))	28	11
6383	(((cp_ent ca_ent)))((ca_wa p_re)ca_wa)((p_csn (oa_ent u_isn))u_hdip)))((u_isn oa_wa))	27	11
6384	(((cp_ent ca_ent)))((p_re ca_wa)((cp_ent oa_ent)oa_wa))(u_isn u_hdip)))((u_isn oa_wa))	32	11
6385	(((cp_ent ca_ent)))((p_re ca_wa)((cp_ent oa_ent)u_hdip))(u_isn oa_wa)))((u_csn cp_ent))	27	11
6386	(((cp_ent ca_ent)))((p_re ca_wa)((cp_ent oa_ent)u_hdip))(u_isn u_hdip)))((u_isn oa_wa))	31	11
6387	(((cp_ent ca_ent)))((ca_wa p_re)ca_wa)(cp_ent oa_ent))(oa_wa u_hdip)))((oa_wa cp_ent))	24	11
6388	(((cp_ent ca_ent)))((ca_wa p_re)ca_wa)(cp_ent oa_ent))(oa_wa u_hdip)))((u_isn u_hdip))	32	11
6389	(((cp_ent ca_ent)))((ca_wa p_re)ca_wa)(cp_ent oa_ent))(u_isn oa_wa)))((u_isn oa_wa))	33	11
6390	(((cp_ent ca_ent)))((ca_wa p_re)ca_wa)(cp_ent oa_ent))(u_isn oa_wa)))((u_isn u_hdip))	32	11
6391	(((cp_ent ca_ent)))((ca_wa p_re)ca_wa)(cp_ent oa_ent))(u_isn u_hdip)))((oa_wa u_hdip))	33	11
6392	(((cp_ent ca_ent)))((ca_wa p_re)ca_wa)(cp_ent oa_ent))(u_isn u_hdip)))((u_isn oa_wa))	36	11
6393	(((cp_ent ca_ent)))((ca_wa p_re)ca_wa)(cp_ent oa_ent))(u_isn u_hdip)))((u_isn u_hdip))	38	11
6394	(((cp_ent ca_ent)))((ca_wa p_re)ca_wa)(oa_ent u_isn))(u_hdip u_hdip)))((u_isn u_hdip))	29	11
6395	(((cp_ent ca_ent)))((ca_wa p_re)ca_wa)(p_csn oa_ent))(u_isn u_hdip)))((u_isn oa_wa))	29	11
6396	(((cp_ent ca_ent)))((ca_wa p_re)cp_ent)(oa_ent u_hdip))(u_isn u_hdip)))((u_isn u_hdip))	35	11
6397	(((cp_ent ca_ent)))((ca_wa p_re)cp_ent)(oa_ent u_isn))(u_hdip u_hdip)))((u_isn u_hdip))	31	11
6398	(((cp_ent ca_ent)))((ca_wa p_re)cp_ent)(oa_ent oa_wa))(u_isn u_hdip)))((u_isn u_hdip))	29	11
6399	(((cp_ent ca_ent)))((ca_wa p_re)cp_ent)(oa_ent u_hdip))(u_isn oa_wa)))((u_isn oa_wa))	29	11
6400	(((cp_ent ca_ent)))((ca_wa p_re)cp_ent)(oa_ent u_hdip))(u_isn oa_wa)))((u_isn u_hdip))	29	11
6401	(((cp_ent ca_ent)))((p_re ca_wa)p_csn)oa_ent)(u_isn u_hdip)))((u_isn oa_wa)u_hdip))	29	11
6402	(((cp_ent ca_ent)))((p_re ca_wa)p_csn)(cp_ent oa_ent))(u_isn u_hdip)))((u_isn u_hdip))	30	11
6403	(((cp_ent ca_ent)))((p_re ca_wa)p_csn)(p_isn oa_ent))(u_isn u_hdip)))((u_isn u_hdip))	21	11
6404	((((ca_ent p_isn)))((p_re ca_wa)p_csn)(p_isn oa_ent))(u_isn u_hdip))(u_isn u_hdip))	21	11
6405	(((cp_ent ca_wa)))((p_re ca_wa)((cp_ent oa_ent)oa_wa))(u_hdip u_hdip))(u_isn oa_wa))	26	11
6406	(((cp_ent ca_wa)))((p_re ca_wa)((cp_ent oa_ent)oa_wa))(u_hdip u_hdip)))((u_isn u_hdip))	22	11
6407	(((cp_ent ca_wa)))((p_re ca_wa)((cp_ent oa_ent)oa_wa))(u_isn u_hdip))(u_isn u_hdip))	27	11
6408	(((cp_ent ca_wa)))((p_re ca_wa)((cp_ent oa_ent)u_hdip))(u_isn oa_wa)))((u_csn cp_ent))	25	11
6409	(((cp_ent ca_wa)))((p_re ca_wa)((cp_ent oa_ent)u_hdip))(u_isn u_hdip)))((u_isn u_hdip))	30	11
6410	(((cp_ent ca_wa)))((p_re ca_wa)p_csn)oa_ent)(u_isn u_hdip)))((u_isn oa_wa)u_hdip))	27	11
6411	(((oa_ent oa_wa)((u_isn u_hdip))((u_isn u_hdip))(u_isn oa_wa)u_csn)))((cp_ent oa_ent))	20	11
6412	(((oa_ent oa_wa)((u_isn u_hdip))((u_isn u_hdip))(u_isn oa_wa)u_csn)))((oa_ent oa_wa))	19	11
6413	(((oa_ent u_hdip)((u_isn u_hdip))((u_isn u_hdip))(u_isn oa_wa)u_csn)))((cp_ent oa_ent))	27	11
6414	(((p_csn ca_ent)))((ca_wa p_re)ca_wa)(cp_ent oa_ent))(u_isn oa_wa)))((u_isn u_hdip))	23	11
6415	(((p_csn ca_ent)))((ca_wa p_re)ca_wa)(cp_ent oa_ent))(u_isn u_hdip)))((u_isn oa_wa))	23	11
6416	(((p_isn ca_ent)((ca_wa p_re))(p_csn (oa_ent u_isn))u_hdip)))((u_isn oa_wa)u_hdip))	21	11
6417	(((p_isn ca_ent)))((p_re ca_wa)((cp_ent oa_ent)oa_wa))(u_isn u_hdip)))((u_isn oa_wa))	26	11
6418	(((p_isn ca_ent)))((ca_wa p_re)ca_wa)(p_csn oa_ent))(oa_wa u_hdip)))((u_isn u_hdip))	22	11
6419	(((p_isn ca_ent)))((ca_wa p_re)cp_ent)oa_ent)(u_isn oa_wa)))((u_hdip (u_isn u_csn)))	20	11
6420	(((p_isn ca_ent)))((ca_wa p_re)cp_ent)(oa_ent oa_wa))(u_hdip u_hdip)))((u_isn oa_wa))	23	11
6421	(((p_isn ca_ent)))((p_re ca_wa)oa_ent)(u_hdip u_hdip))(u_isn u_hdip)))((oa_wa u_csn))	23	11
6422	(((p_isn ca_ent)))((p_re ca_wa)p_csn)oa_ent)(u_isn u_hdip)))((u_isn oa_wa)u_hdip))	25	11
6423	(((p_isn ca_wa)((p_re ca_wa))((p_csn (oa_ent u_isn))(u_hdip u_hdip)))((u_isn u_hdip))	30	11
6424	(((ca_ent ca_wa)((p_re ca_wa)p_csn)(p_isn oa_ent)))((u_hdip u_hdip))(u_isn u_hdip))	23	11
6425	(((p_isn ca_wa)))((p_re ca_wa)(p_csn (p_isn oa_ent)))((u_hdip u_hdip)))((u_isn u_hdip))	23	11
6426	(((p_isn ca_wa)))((p_re ca_wa)((cp_ent oa_ent)oa_wa))(u_hdip u_hdip)))((u_isn u_hdip))	29	11
6427	(((p_isn ca_wa)))((p_re ca_wa)((cp_ent oa_ent)oa_wa))(u_isn u_hdip)))((u_isn u_hdip))	30	11
6428	(((p_isn ca_wa)))((p_re ca_wa)oa_ent)(oa_wa u_hdip))(u_isn u_hdip)))((u_isn u_hdip))	24	11
6429	(((p_isn ca_wa)))((p_re ca_wa)oa_ent)(u_isn u_hdip))(u_isn u_hdip)))((u_isn u_hdip))	29	11
6430	(((p_isn ca_wa)))((p_re ca_wa)p_csn)(oa_ent u_hdip))(u_isn u_hdip)))((u_isn u_hdip))	31	11
6431	(((u_csn (cp_ent ca_ent)))((ca_wa p_re)cp_ent)oa_ent)(u_isn oa_wa)))((u_isn oa_wa))	25	11
6432	(((u_csn (cp_ent ca_ent)))((p_re ca_wa)p_csn)oa_ent)(u_hdip u_hdip)))((u_isn u_hdip))	24	11
6433	(((u_csn (cp_ent ca_ent)))((p_re ca_wa)p_csn)oa_ent)(u_isn u_hdip)))((u_isn u_hdip))	27	11
6434	((((u_hdip u_hdip)((u_isn oa_wa)((u_csn cp_ent)p_csn)))ca_ent))(ca_wa p_re)ca_wa))	27	11
6435	((((u_hdip u_hdip)((u_isn oa_wa)((u_csn cp_ent)p_csn)))ca_ent))(ca_wa p_re)cp_ent))	27	11
6436	(((u_hdip u_hdip)))((oa_wa (u_csn cp_ent))p_csn)(ca_ent ca_wa)))((p_re ca_wa)oa_ent))	18	11
6437	(((u_hdip u_hdip)))((oa_wa (u_csn cp_ent))p_csn)ca_ent)))((ca_wa p_re)ca_wa)oa_ent))	19	11
6438	((((u_hdip u_hdip)((u_isn oa_wa)((u_csn cp_ent)p_csn)))(ca_ent p_isn))(p_re ca_wa))	27	11
6439	(((u_hdip u_hdip)))((oa_wa (u_csn cp_ent))p_csn)(p_isn ca_ent)))((ca_wa p_re)cp_ent))	21	11
6440	(((oa_ent (u_isn u_hdip)))((u_isn u_hdip))(u_isn u_hdip))(u_isn oa_wa)u_csn)))oa_ent)	23	11
6441	(((p_csn ((ca_ent ca_wa))((p_re ca_wa)(cp_ent oa_ent)))((u_isn u_hdip))(u_isn u_hdip))	23	11
6442	(((p_csn ((p_isn ca_wa))((p_re ca_wa)(cp_ent oa_ent)))((u_isn u_hdip))(u_isn u_hdip))	20	11
6443	((((ca_ent ca_wa)((p_re ca_wa)oa_ent)(oa_wa u_hdip)))((u_isn u_hdip))(oa_wa u_csn))	26	11
6444	((((ca_ent ca_wa)((p_re ca_wa)p_csn)(cp_ent oa_ent)))((u_isn oa_wa))(u_isn oa_wa))	27	11
6445	((((ca_ent ca_wa)((p_re ca_wa)p_csn)(cp_ent oa_ent)))((u_isn oa_wa))(u_isn u_hdip))	27	11
6446	((((ca_ent p_isn))((p_re ca_wa)((p_csn oa_ent)oa_wa)))((u_isn u_hdip))(u_isn u_hdip))	28	11
6447	((((ca_ent p_isn))((p_re ca_wa)p_csn)(cp_ent oa_ent)))((u_isn oa_wa))(u_isn oa_wa))	26	11
6448	((((ca_ent p_isn))((p_re ca_wa)p_csn)(cp_ent oa_ent)))((u_isn oa_wa))(u_isn u_hdip))	26	11
6449	(((ca_wa ca_wa)((p_csn (oa_ent u_isn))(u_hdip u_hdip)))((u_isn u_hdip))(u_isn u_hdip))	22	11
6450	((((cp_ent ca_ent)((ca_wa p_re)ca_wa)(cp_ent oa_ent)))((oa_wa oa_wa))(u_csn cp_ent))	28	11
6451	((((cp_ent ca_ent)((ca_wa p_re)ca_wa)(cp_ent oa_ent)))((oa_wa u_hdip))(u_isn u_hdip))	31	11
6452	((((cp_ent ca_ent)((ca_wa p_re)ca_wa)(oa_ent u_isn)))((u_hdip u_hdip))(u_isn u_hdip))	30	11
6453	((((cp_ent ca_ent)((ca_wa p_re)cp_ent)(oa_ent oa_wa)))((u_hdip u_hdip))(u_isn oa_wa))	28	11
6454	((((cp_ent ca_ent)((ca_wa p_re)cp_ent)(oa_ent oa_wa)))((u_hdip u_hdip))(u_isn u_hdip))	23	11
6455	((((cp_ent ca_ent)((ca_wa p_re)cp_ent)(oa_ent u_hdip)))((u_isn oa_wa))(u_isn oa_wa))	30	11
6456	((((cp_ent ca_ent)((ca_wa p_re)cp_ent)(oa_ent u_hdip)))((u_isn oa_wa))(u_isn u_hdip))	31	11
6457	((((cp_ent ca_ent)((ca_wa p_re)cp_ent)(oa_ent u_hdip)))((u_isn u_hdip))(u_isn u_hdip))	34	11
6458	((((cp_ent ca_wa)((p_re ca_wa)((cp_ent oa_ent)oa_wa)))((u_isn u_hdip))(u_isn oa_wa))	28	11
6459	((((cp_ent ca_wa)((p_re ca_wa)((cp_ent oa_ent)oa_wa)))((u_isn u_hdip))(u_isn u_hdip))	29	11
6460	((((oa_wa u_hdip)((u_csn cp_ent)p_csn))ca_ent)))((ca_wa p_re)cp_ent))(oa_ent u_hdip))	21	11
6461	((((p_isn ca_ent)((p_re ca_wa)((cp_ent oa_ent)u_hdip)))((u_isn oa_wa))(u_csn cp_ent))	25	11
6462	((((p_isn ca_ent)((p_re ca_wa)((cp_ent oa_ent)u_hdip)))((u_isn u_hdip))(u_isn u_hdip))	29	11
6463	((((p_isn ca_ent)((ca_wa p_re)ca_wa)(cp_ent oa_ent)))((oa_wa u_hdip))(oa_wa cp_ent))	22	11
6464	((((p_isn ca_ent)((ca_wa p_re)ca_wa)(cp_ent oa_ent)))((oa_wa u_hdip))(u_isn u_hdip))	28	11
6465	((((p_isn ca_ent)((ca_wa p_re)ca_wa)(cp_ent oa_ent)))((u_isn u_hdip))(oa_wa u_hdip))	30	11
6466	((((p_isn ca_ent)((ca_wa p_re)ca_wa)(cp_ent oa_ent)))((u_isn u_hdip))(u_isn oa_wa))	32	11
6467	((((p_isn ca_ent)((ca_wa p_re)ca_wa)(cp_ent oa_ent)))((u_isn u_hdip))(u_isn u_hdip))	35	11
6468	((((p_isn ca_ent)((ca_wa p_re)cp_ent)(oa_ent u_isn)))((u_hdip u_hdip))(u_isn u_hdip))	31	11
6469	((((p_isn ca_ent)((p_re ca_wa)p_csn)(oa_ent u_isn)))((u_hdip u_hdip))(u_isn u_hdip))	25	11
6470	((((p_isn ca_wa)((p_isn p_re))(p_csn cp_ent))oa_ent))(u_isn u_hdip))(u_isn oa_wa))	21	11
6471	((((p_isn ca_wa)((p_isn p_re))(p_csn cp_ent))oa_ent))(u_isn u_hdip))(u_isn u_hdip))	29	11
6472	((((u_csn cp_ent)((ca_ent ca_wa)cp_ent))(oa_ent u_hdip)))((u_isn u_hdip))(u_isn u_hdip))	24	11
6473	((((u_hdip oa_wa)((u_csn cp_ent)((p_isn oa_ent)oa_wa)))((u_isn u_hdip))(u_isn u_hdip))	22	11
6474	((((u_isn oa_wa)((u_csn cp_ent)((p_isn oa_ent)oa_wa)))((u_isn u_hdip))(u_isn u_hdip))	24	11
6475	((((u_isn u_hdip)((u_isn oa_wa)((u_csn cp_ent)p_csn)))ca_ent))(ca_wa p_re)ca_wa))	25	11
6476	((((u_isn u_hdip)((u_isn oa_wa)((u_csn cp_ent)p_csn)))ca_ent))(ca_wa p_re)cp_ent))	25	11
6477	((((u_isn u_hdip)((u_isn oa_wa)((u_csn cp_ent)p_csn)))(ca_ent ca_wa))(p_re ca_wa))	26	11
6478	((((p_csn (oa_ent u_hdip))(u_isn oa_wa)))((u_csn cp_ent)p_csn))ca_ent)(ca_wa ca_wa))	25	11
6479	((((p_csn (oa_ent (u_isn u_hdip)))((u_isn u_hdip)))((u_csn cp_ent)p_csn))(ca_ent p_isn))	24	11
6480	((((p_csn (oa_ent (u_isn u_hdip)))((u_isn u_hdip)))((u_csn cp_ent)p_csn))(ca_ent ca_wa))	24	11
6481	((((p_csn (oa_ent (u_isn u_hdip)))((u_isn u_hdip)))((u_csn cp_ent)p_csn))(ca_wa ca_wa))	17	11
6482	((((p_csn (oa_ent (u_isn u_hdip)))((u_isn u_hdip)))((u_csn cp_ent)p_csn))(p_isn ca_wa))	15	11
6483	((((p_csn ((oa_ent u_isn)u_hdip))((u_isn oa_wa))(u_csn cp_ent))(p_csn (ca_ent ca_wa)))	25	11
6484	((((p_csn ((p_isn ca_ent)(ca_wa p_re)))((ca_wa p_csn))(cp_ent oa_ent))(u_isn u_hdip))	19	11
6485	((((ca_ent ca_wa)((p_isn ca_wa)p_csn))((oa_ent oa_wa)))((u_isn u_hdip))(u_isn u_hdip))	24	11
6486	((((ca_ent ca_wa)((p_isn ca_wa)p_csn))((p_isn oa_ent)))((u_isn u_hdip))(u_isn u_hdip))	21	11
6487	((((ca_ent ca_wa)((p_re ca_wa)oa_ent))(u_isn u_hdip))(u_isn oa_wa))(u_csn cp_ent))	25	11
6488	((((ca_ent ca_wa)((p_re ca_wa)oa_ent))(u_isn u_hdip))(u_isn u_hdip))(u_isn u_hdip))	29	11
6489	((((ca_ent ca_wa)((p_re ca_wa)p_csn))oa_ent)(u_isn u_hdip)))((u_isn oa_wa)u_hdip))	31	11
6490	((((ca_ent ca_wa)((p_re ca_wa)p_csn))(cp_ent oa_ent))(u_isn u_hdip))(u_isn oa_wa))	28	11
6491	((((ca_ent ca_wa)((p_re ca_wa)p_csn))(oa_ent u_isn))(oa_wa u_hdip))(oa_wa cp_ent))	21	11
6492	((((ca_ent ca_wa)((p_re ca_wa)p_csn))(oa_ent u_isn))(u_hdip u_hdip))(u_isn u_hdip))	32	11
6493	((((cp_ent ca_ent)((ca_wa p_re)cp_ent))(oa_ent oa_wa)))((u_hdip u_hdip))(u_isn oa_wa))	29	11
6494	((((cp_ent ca_ent)((ca_wa p_re)cp_ent))(oa_ent oa_wa)))((u_hdip u_hdip))(u_isn u_hdip))	26	11
6495	((((cp_ent ca_ent)((ca_wa p_re)cp_ent))(oa_ent oa_wa)))((u_isn u_hdip))(u_isn u_hdip))	30	11
6496	((((cp_ent ca_ent)((ca_wa p_re)cp_ent))(oa_ent u_hdip)))((u_isn oa_wa))(u_isn oa_wa))	31	11
6497	((((cp_ent ca_ent)((ca_wa p_re)cp_ent))(oa_ent u_hdip)))((u_isn oa_wa))(u_isn u_hdip))	32	11
6498	((((cp_ent ca_ent)((ca_wa p_re)cp_ent))(oa_ent u_hdip)))((u_isn u_hdip))(u_isn u_hdip))	36	11
6499	((((cp_ent ca_ent)((ca_wa p_re)cp_ent))(oa_ent u_isn)))((u_hdip u_hdip))(u_isn u_hdip))	32	11
6500	((((cp_ent ca_ent)((p_re ca_wa)oa_ent)))((u_hdip u_hdip))(u_isn u_hdip))(oa_wa u_csn))	25	11
6501	((((cp_ent ca_ent)((p_re ca_wa)oa_ent)))((u_isn u_hdip))(u_isn oa_wa))(u_csn cp_ent))	22	11
6502	((((cp_ent ca_wa)((p_re ca_wa)(p_csn cp_ent)))oa_ent)(u_isn u_hdip))(u_isn u_hdip))	26	11
6503	((((oa_wa u_csn)((cp_ent ca_wa)cp_ent))(oa_ent u_isn)))((u_hdip oa_wa))(u_isn oa_wa))	14	11
6504	((((p_csn oa_ent)((u_isn oa_wa)u_hdip))(u_isn oa_wa)))((u_csn cp_ent)p_csn))ca_ent)	21	11
6505	((((p_isn ca_ent)((p_re ca_wa)p_csn))(cp_ent oa_ent))(u_isn u_hdip))(u_isn u_hdip))	26	11
6506	((((p_re ca_wa)(p_csn (oa_ent u_isn)))((u_hdip u_hdip))(u_isn u_hdip))(u_isn u_hdip))	24	11
6507	((((p_re ca_wa)((cp_ent oa_ent)oa_wa)))((u_hdip u_hdip))(u_isn u_hdip))(u_isn oa_wa))	27	11
6508	((((p_re ca_wa)((cp_ent oa_ent)oa_wa)))((u_isn u_hdip))(u_isn u_hdip))(u_isn oa_wa))	28	11

6509	((((p_re ca_wa)((cp_ent oa_ent)oa_wa))(u_isn u_hdip))(u_isn u_hdip))(u_isn u_hdip))	27	11
6510	((((p_re ca_wa)((cp_ent oa_ent)u_hdip))(u_isn u_hdip))(u_isn u_hdip))(oa_wa u_csn))	31	11
6511	((((p_re ca_wa)((cp_ent oa_ent)u_hdip))(u_isn u_hdip))(u_isn u_hdip))(u_isn oa_wa))	31	11
6512	((((p_re ca_wa)((cp_ent oa_ent)u_hdip))(u_isn u_hdip))(u_isn u_hdip))(u_isn u_hdip))	30	11
6513	((((p_re ca_wa)((p_csn oa_ent)u_hdip))(u_isn u_hdip))(u_isn u_hdip))(u_isn u_hdip))	23	11
6514	(((((u_csn cp_ent)((ca_wa p_re)ca_wa))(cp_ent oa_ent))(u_hdip u_hdip))(u_isn u_hdip))	26	11
6515	((((u_csn cp_ent)((ca_wa p_re)ca_wa))(cp_ent oa_ent))(u_isn oa_wa))(u_isn oa_wa))	26	11
6516	((((u_csn cp_ent)((ca_wa p_re)ca_wa))(cp_ent oa_ent))(u_isn oa_wa))(u_isn u_hdip))	25	11
6517	((((oa_ent (oa_wa u_hdip)))(u_isn oa_wa)u_csn))oa_ent)(oa_wa u_hdip))(u_isn u_hdip))	17	11
6518	((((p_csn (ca_ent ca_wa)))(p_re ca_wa)p_csn))oa_ent)(u_isn u_hdip))(u_isn u_hdip))	25	11
6519	((((p_csn (oa_ent u_isn))(u_hdip u_hdip))(u_isn oa_wa))((u_csn cp_ent)p_csn))ca_ent)	24	11
6520	((((u_csn (ca_ent ca_wa))(p_isn ca_wa))(oa_ent u_isn))(u_hdip u_hdip))(u_isn u_hdip))	22	11
6521	(((((ca_ent ca_wa)cp_ent)(oa_ent oa_wa))(u_isn u_hdip))(u_isn u_hdip))(u_isn oa_wa))	28	11
6522	(((((ca_ent ca_wa)cp_ent)(oa_ent u_isn))(u_hdip u_hdip))(u_isn u_hdip))(u_isn oa_wa))	27	11
6523	(((((ca_ent ca_wa)cp_ent)(oa_ent u_isn))(u_hdip u_hdip))(u_isn u_hdip))(u_isn u_hdip))	24	11
6524	(((((ca_ent ca_wa)p_csn)(cp_ent oa_ent))(u_isn u_hdip))(u_isn oa_wa))(u_isn oa_wa))	23	11
6525	(((((ca_ent ca_wa)p_csn)(oa_ent u_isn))(u_hdip u_hdip))(u_isn u_hdip))(u_isn u_hdip))	22	11
6526	(((((ca_ent ca_wa)p_re)(cp_ent oa_ent))(oa_wa u_hdip))(u_isn u_hdip))(u_isn oa_wa))	27	11
6527	(((((ca_ent ca_wa)p_re)(cp_ent oa_ent))(oa_wa u_hdip))(u_isn u_hdip))(u_isn u_hdip))	27	11
6528	(((((ca_ent ca_wa)p_re)(cp_ent oa_ent))(u_hdip u_hdip))(u_isn oa_wa))(u_csn cp_ent))	23	11
6529	(((((ca_ent ca_wa)p_re)(cp_ent oa_ent))(u_hdip u_hdip))(u_isn u_hdip))(u_isn oa_wa))	29	11
6530	(((((ca_ent ca_wa)p_re)(cp_ent oa_ent))(u_hdip u_hdip))(u_isn u_hdip))(u_isn u_hdip))	24	11
6531	(((((ca_ent ca_wa)p_re)(p_csn oa_ent))(u_hdip u_hdip))(u_isn u_hdip))(u_isn u_hdip))	21	11
6532	(((((ca_ent p_re)(cp_ent oa_ent))oa_wa)(u_hdip u_hdip))(u_isn u_hdip))(u_isn oa_wa))	19	11
6533	(((((ca_wa p_re)ca_wa)oa_ent)(u_hdip u_hdip))(u_isn u_hdip))(oa_wa (u_csn cp_ent)))	28	11
6534	(((((ca_wa p_re)ca_wa)(cp_ent oa_ent))(oa_wa u_hdip))(u_isn u_hdip))(u_isn u_hdip))	30	11
6535	(((((ca_wa p_re)ca_wa)(cp_ent oa_ent))(u_hdip u_hdip))(u_isn oa_wa))(u_csn cp_ent))	26	11
6536	(((((ca_wa p_re)ca_wa)(cp_ent oa_ent))(u_isn u_hdip))(u_isn oa_wa))(u_isn oa_wa))	31	11
6537	(((((ca_wa p_re)ca_wa)(cp_ent oa_ent))(u_isn u_hdip))(u_isn oa_wa))(u_isn u_hdip))	33	11
6538	(((((ca_wa p_re)ca_wa)(oa_ent u_isn))(u_hdip u_hdip))(u_isn oa_wa))(u_csn cp_ent))	25	11
6539	(((((ca_wa p_re)ca_wa)(oa_ent u_isn))(u_hdip u_hdip))(u_isn u_hdip))(oa_wa u_csn))	28	11
6540	(((((ca_wa p_re)ca_wa)(oa_ent u_isn))(u_hdip u_hdip))(u_isn u_hdip))(u_isn oa_wa))	27	11
6541	(((((ca_wa p_re)ca_wa)(oa_ent u_isn))(u_hdip u_hdip))(u_isn u_hdip))(u_isn u_hdip))	23	11
6542	(((((ca_wa p_re)cp_ent)oa_ent))(oa_wa u_hdip)(u_isn oa_wa)))(u_csn cp_ent))oa_ent)	19	11
6543	(((((ca_wa p_re)cp_ent)(oa_ent oa_wa))(u_hdip u_hdip))(u_isn u_hdip))(u_isn oa_wa))	27	11
6544	(((((ca_wa p_re)cp_ent)(oa_ent oa_wa))(u_isn u_hdip))(u_isn u_hdip))(u_isn oa_wa))	26	11
6545	(((((ca_wa p_re)cp_ent)(oa_ent u_hdip))(u_isn u_hdip))(u_isn u_hdip))(u_isn oa_wa))	30	11
6546	(((((ca_wa p_re)cp_ent)(oa_ent u_hdip))(u_isn u_hdip))(u_isn u_hdip))(u_isn u_hdip))	30	11
6547	(((((ca_wa p_re)cp_ent)(oa_ent u_isn))(u_hdip u_hdip))(u_isn u_hdip))(u_isn oa_wa))	27	11
6548	(((((ca_wa p_re)cp_ent)(oa_ent u_isn))(u_hdip u_hdip))(u_isn u_hdip))(u_isn u_hdip))	24	11
6549	(((((cp_ent ca_ent)cp_ent)oa_ent))(oa_wa u_hdip)(u_isn oa_wa)))(u_csn cp_ent))oa_ent)	18	11
6550	(((((cp_ent ca_ent)cp_ent)(oa_ent oa_wa))(u_isn u_hdip))(u_isn u_hdip))(u_isn oa_wa))	28	11
6551	(((((cp_ent ca_ent)cp_ent)(oa_ent u_hdip))(u_isn u_hdip))(u_isn oa_wa))(u_csn cp_ent))	25	11
6552	(((((cp_ent ca_ent)cp_ent)(oa_ent u_hdip))(u_isn u_hdip))(u_isn u_hdip))(u_isn u_hdip))	24	11
6553	(((((cp_ent ca_ent)cp_ent)(oa_ent u_isn))(u_hdip u_hdip))(u_isn u_hdip))(u_isn oa_wa))	26	11
6554	(((((cp_ent ca_ent)cp_ent)(oa_ent u_isn))(u_hdip u_hdip))(u_isn u_hdip))(u_isn u_hdip))	22	11
6555	(((((cp_ent ca_wa)cp_ent)(oa_ent oa_wa))(u_hdip u_hdip))(u_isn u_hdip))(u_isn oa_wa))	21	11
6556	(((((cp_ent ca_wa)cp_ent)(oa_ent oa_wa))(u_isn u_hdip))(u_isn u_hdip))(u_isn oa_wa))	27	11
6557	(((((cp_ent ca_wa)cp_ent)(oa_ent u_hdip))(u_isn u_hdip))(u_isn u_hdip))(u_isn oa_wa))	29	11
6558	(((((cp_ent ca_wa)cp_ent)(oa_ent u_hdip))(u_isn u_hdip))(u_isn u_hdip))(u_isn u_hdip))	23	11
6559	(((((cp_ent ca_wa)cp_ent)(oa_ent u_isn))(u_hdip u_hdip))(u_isn u_hdip))(u_isn oa_wa))	26	11
6560	(((((cp_ent ca_wa)cp_ent)(oa_ent u_isn))(u_hdip u_hdip))(u_isn u_hdip))(u_isn u_hdip))	21	11
6561	(((((p_isn oa_ent)oa_wa)u_csn))(cp_ent oa_ent)oa_wa))(u_isn u_hdip))(u_isn u_hdip))	16	11
6562	(((((p_re ca_wa)p_csn)(oa_ent u_isn))(u_hdip u_hdip))(u_isn u_hdip))(u_isn u_hdip))	25	11
6563	(((((u_hdip oa_wa)u_csn)((cp_ent ca_wa)cp_ent))oa_ent)(oa_wa u_hdip))(u_isn u_hdip))	18	11
6564	(((((u_isn oa_wa)u_hdip)(oa_wa u_csn))oa_ent)((oa_wa u_hdip)oa_wa))(cp_ent ca_ent))	21	11
6565	(((((u_isn oa_wa)u_hdip)(oa_wa u_csn))oa_ent)((oa_wa u_hdip)oa_wa))(ca_ent p_isn))	21	11
6566	(((((u_isn oa_wa)u_hdip)(oa_wa u_csn))oa_ent)((oa_wa u_hdip)oa_wa))(ca_ent ca_wa))	21	11
6567	(((((u_isn oa_wa)u_hdip)(oa_wa u_csn))oa_ent)((oa_wa u_hdip)oa_wa))(cp_ent ca_wa))	21	11
6568	(p_csn ((p_isn ca_ent)))(((ca_wa p_re)ca_wa)oa_ent)(u_isn u_hdip))(u_isn u_hdip))))	19	11
6569	(p_csn ((p_isn ca_ent)))(((ca_wa p_re)cp_ent)oa_ent)(u_isn u_hdip))(u_isn u_hdip))))	24	11
6570	((ca_ent ca_wa))))(p_isn p_re)(ca_wa (p_csn cp_ent)))((oa_ent u_isn)u_hdip))(u_isn u_hdip)))	31	12
6571	(((u_csn cp_ent)(ca_ent ca_wa)))((p_re ca_wa)(cp_ent oa_ent))((oa_wa u_hdip)(u_isn oa_wa))))	22	12
6572	((u_hdip (u_isn oa_wa)))((u_csn cp_ent)(p_csn (ca_ent ca_wa)))((p_re ca_wa)(cp_ent oa_ent))))	20	12
6573	((cp_ent ca_ent)((((p_isn p_re)(ca_wa (p_csn cp_ent)))((oa_ent u_isn)u_hdip))(u_isn u_hdip)))	29	12
6574	(((oa_wa (u_csn cp_ent))(ca_ent ca_wa)))(((p_re ca_wa)p_csn)(p_isn oa_ent))(u_isn u_hdip)))	20	12
6575	(((oa_wa u_csn)(cp_ent ca_wa)))(((p_re ca_wa)(cp_ent oa_ent))((oa_wa u_hdip)(u_isn oa_wa))))	20	12
6576	(((oa_wa (u_csn cp_ent))p_csn)(ca_ent ca_wa)))((p_re ca_wa)(cp_ent oa_ent))(u_hdip u_hdip)))	21	12
6577	((((u_csn cp_ent)(p_isn ca_ent)))((ca_wa p_re)ca_wa)((cp_ent oa_ent)u_hdip)))(u_isn u_hdip))	25	12
6578	((((u_csn cp_ent)(p_isn ca_ent)))((ca_wa p_re)ca_wa)((p_csn oa_ent)u_hdip)))(u_isn u_hdip))	22	12
6579	(((u_csn cp_ent)(p_isn ca_wa)))(((p_re ca_wa)(cp_ent oa_ent))((oa_wa u_hdip)(u_isn oa_wa))))	19	12
6580	(((u_hdip oa_wa)u_hdip)))(u_csn cp_ent)(p_csn (ca_ent ca_wa)))((p_re ca_wa)(cp_ent oa_ent))))	17	12
6581	(((u_isn cp_ent)(ca_ent ca_wa)))((p_isn p_re)(ca_wa (p_csn cp_ent)))((oa_ent u_isn)u_hdip)))	17	12
6582	(((u_isn cp_ent)(ca_ent ca_wa))(p_isn p_re)))((ca_wa (p_csn cp_ent))oa_ent)(u_isn u_hdip)))	18	12
6583	(((u_isn cp_ent)(ca_ent ca_wa)))(p_isn p_re)((ca_wa (p_csn cp_ent))oa_ent)(u_hdip u_hdip))))	17	12
6584	((((u_isn oa_wa)u_hdip))(u_csn (cp_ent ca_ent)))((ca_wa p_re)ca_wa)))((cp_ent oa_ent)u_hdip))	22	12
6585	((((u_isn oa_wa)u_hdip)))(u_csn cp_ent)(p_csn (ca_ent ca_wa)))((p_re ca_wa)(cp_ent oa_ent))))	19	12
6586	(((oa_wa u_hdip)))(u_csn cp_ent)p_csn)(ca_ent ca_wa)))((p_re ca_wa)((cp_ent oa_ent)u_hdip)))	20	12
6587	(((oa_wa u_hdip)))(u_csn cp_ent)p_csn)ca_ent))((ca_wa p_re)cp_ent)((oa_ent u_isn)u_hdip)))	20	12
6588	(((u_csn (cp_ent ca_ent)))(ca_wa p_re)ca_wa)))((p_csn oa_ent)(u_isn u_hdip))(u_isn u_hdip)))	27	12
6589	((((u_csn cp_ent)(ca_ent ca_wa)))((p_re ca_wa)(cp_ent oa_ent)))((oa_wa u_hdip)(u_isn oa_wa)))	23	12
6590	((((u_csn cp_ent)(p_csn (p_isn ca_ent))))((ca_wa p_re)cp_ent)((p_isn oa_ent)(u_isn u_hdip)))	16	12
6591	((((u_csn cp_ent)((p_isn ca_ent)(ca_wa p_re)))((p_csn oa_ent)(u_isn u_hdip)))(u_isn u_hdip))	24	12
6592	(((u_hdip oa_wa)(u_csn (cp_ent ca_ent)))(((ca_wa p_re)ca_wa)(cp_ent oa_ent))(oa_wa u_hdip)))	21	12
6593	(((u_hdip oa_wa)((u_csn cp_ent)(ca_ent ca_wa)))((p_re ca_wa)(cp_ent oa_ent))(oa_wa u_hdip)))	20	12
6594	(((u_hdip oa_wa)(u_csn (cp_ent ca_ent)))(((ca_wa p_re)ca_wa)(cp_ent oa_ent))(u_hdip u_hdip)))	24	12
6595	(((u_hdip oa_wa)))(u_csn cp_ent)p_csn)(ca_ent ca_wa)))((p_re ca_wa)((cp_ent oa_ent)u_hdip)))	20	12
6596	(((u_hdip (u_isn oa_wa)))(u_csn cp_ent)(p_csn ca_ent))))((ca_wa p_re)ca_wa)(oa_ent u_isn)))	18	12
6597	(((u_hdip (u_isn oa_wa)))(u_csn cp_ent)p_csn)ca_ent))((ca_wa p_re)cp_ent)(oa_ent u_isn)))	20	12
6598	(((u_hdip oa_wa)))(u_csn cp_ent)p_csn)ca_ent))((ca_wa p_re)cp_ent)((oa_ent u_isn)u_hdip)))	20	12
6599	(((u_hdip u_hdip))(u_csn cp_ent)(p_csn (ca_ent ca_wa)))((p_re ca_wa)((cp_ent oa_ent)u_hdip)))	17	12
6600	(((u_hdip u_hdip))(u_csn cp_ent)(p_csn ca_ent)))((ca_wa p_re)cp_ent)((oa_ent u_isn)u_hdip)))	17	12
6601	(((oa_wa (u_csn cp_ent))(p_isn ca_ent)))((ca_wa p_re)ca_wa)((cp_ent oa_ent)(oa_wa u_hdip))))	19	12
6602	(((oa_wa (u_csn cp_ent))(p_isn ca_ent)))((ca_wa p_re)ca_wa)((p_csn (oa_ent u_isn))u_hdip)))	19	12
6603	(((oa_wa (u_csn cp_ent))(p_isn ca_ent)))((ca_wa p_re)ca_wa)((p_csn oa_ent)(u_isn u_hdip))))	20	12
6604	((((oa_wa (u_csn cp_ent))(p_isn ca_ent)))((ca_wa p_re)((p_csn oa_ent)u_hdip)))(u_isn u_hdip))	18	12
6605	(((oa_wa (u_csn cp_ent))(p_isn ca_ent))))((p_re ca_wa)p_csn))(p_isn oa_ent)(u_isn u_hdip)))	17	12
6606	(((oa_wa (u_csn cp_ent))(p_isn ca_wa)))((p_re ca_wa)((cp_ent oa_ent)u_hdip))(u_isn u_hdip)))	21	12
6607	(((oa_wa (u_csn cp_ent))(p_csn (p_isn ca_ent))))((ca_wa p_re)ca_wa)((cp_ent oa_ent)u_hdip)))	17	12
6608	(((oa_wa (u_csn cp_ent))(p_csn (p_isn ca_ent))))((ca_wa p_re)cp_ent)((oa_ent u_isn)u_hdip)))	19	12
6609	((((u_csn cp_ent)(ca_ent ca_wa))(p_isn ca_wa)))((cp_ent oa_ent)(u_isn u_hdip)))(u_isn u_hdip))	31	12
6610	(((u_csn (u_isn cp_ent))(p_isn ca_ent)))((ca_wa p_re)ca_wa)(cp_ent oa_ent))(u_isn u_hdip)))	20	12
6611	(((u_hdip (oa_wa u_csn))(cp_ent ca_ent))))((ca_wa p_re)ca_wa)(cp_ent oa_ent))(oa_wa u_hdip)))	19	12
6612	(((u_hdip (oa_wa u_csn))(cp_ent ca_ent))))((ca_wa p_re)ca_wa)(cp_ent oa_ent))(u_hdip u_hdip)))	22	12
6613	(((u_hdip (oa_wa u_csn))(p_csn (ca_ent ca_wa)))((p_re ca_wa)(cp_ent oa_ent))(u_hdip u_hdip)))	17	12
6614	(((u_hdip (u_csn cp_ent))(p_csn ca_ent))))((ca_wa p_re)ca_wa)(cp_ent oa_ent))(u_hdip u_hdip)))	20	12
6615	((((u_hdip (u_csn cp_ent))(p_isn ca_ent)))((ca_wa p_re)cp_ent)(oa_ent u_hdip)))(u_isn u_hdip))	21	12
6616	(((u_hdip (u_csn cp_ent))(p_csn (p_isn ca_ent))))((ca_wa p_re)ca_wa)((cp_ent oa_ent)u_hdip)))	16	12
6617	(((u_hdip (u_isn oa_wa)))(u_csn cp_ent)(ca_ent ca_wa)))((p_re ca_wa)((cp_ent oa_ent)oa_wa)))	20	12
6618	(((u_hdip (u_isn oa_wa)))(u_csn (cp_ent ca_ent)))((ca_wa p_re)ca_wa)))((cp_ent oa_ent)u_hdip)))	25	12
6619	(((u_hdip (u_isn oa_wa))(u_csn (cp_ent ca_ent))))((ca_wa p_re)cp_ent)((oa_ent u_isn)u_hdip)))	24	12
6620	(((oa_ent (oa_wa u_hdip)))(u_isn u_hdip))(u_isn oa_wa)u_csn))))(cp_ent oa_ent)(u_isn u_hdip)))	16	12
6621	((((oa_wa u_csn)(cp_ent ca_wa)))((p_re ca_wa)(cp_ent oa_ent)))((oa_wa u_hdip)(u_isn oa_wa)))	21	12
6622	((((oa_wa u_hdip)oa_wa)(u_csn (cp_ent ca_ent)))((ca_wa p_re)ca_wa)((cp_ent oa_ent)oa_wa)))	22	12
6623	((((oa_wa u_hdip)oa_wa))(u_csn cp_ent)(p_csn (ca_ent ca_wa)))((p_re ca_wa)(cp_ent oa_ent)))	19	12
6624	((((oa_wa u_hdip)(oa_wa (u_csn cp_ent)))(p_csn (ca_ent ca_wa)))((p_re ca_wa)(cp_ent oa_ent)))	18	12
6625	((((oa_wa u_hdip)oa_wa))(u_csn cp_ent)(p_csn ca_ent))))((ca_wa p_re)cp_ent)(oa_ent u_isn)))	21	12
6626	((((oa_wa u_hdip)oa_wa))(u_csn cp_ent)(ca_ent ca_wa)))((p_re ca_wa)((cp_ent oa_ent)oa_wa)))	21	12
6627	((((oa_wa u_hdip)oa_wa))(u_csn cp_ent)(p_isn ca_wa)))((p_re ca_wa)((cp_ent oa_ent)oa_wa)))	19	12
6628	((((oa_wa u_hdip)(u_isn oa_wa)))(u_csn cp_ent)(ca_ent ca_wa)))((p_re ca_wa)(cp_ent oa_ent)))	20	12
6629	((((oa_wa u_hdip)(u_isn oa_wa))(u_csn (cp_ent ca_ent)))((ca_wa p_re)cp_ent)(oa_ent u_isn)))	19	12
6630	((((u_csn cp_ent)(ca_ent ca_wa))(p_re p_re)))((p_csn oa_ent)(u_isn u_hdip))(u_isn u_hdip)))	22	12
6631	((((u_csn cp_ent)(ca_wa p_re))(p_isn ca_wa)))((p_csn oa_ent)(u_isn u_hdip))(u_isn u_hdip)))	20	12
6632	(((((u_csn cp_ent)(p_isn ca_ent))(p_re p_re)))((p_csn oa_ent)(u_isn u_hdip)))(u_isn u_hdip))	21	12
6633	(((u_hdip oa_wa)u_csn)(cp_ent ca_ent))))((ca_wa p_re)ca_wa)(cp_ent oa_ent))(oa_wa u_hdip)))	22	12
6634	((((u_hdip oa_wa)u_csn)(cp_ent ca_ent)))((ca_wa p_re)ca_wa)((p_csn (oa_ent u_isn))u_hdip)))	20	12
6635	((((u_hdip oa_wa)u_csn)(cp_ent ca_ent)))((ca_wa p_re)ca_wa)((p_csn oa_ent)(u_isn u_hdip))))	21	12
6636	((((u_hdip oa_wa)u_csn)(ca_ent ca_wa)))((p_re ca_wa)p_csn)(p_isn oa_ent))(u_isn u_hdip)))	19	12
6637	((((u_hdip oa_wa)u_csn)(cp_ent ca_ent)))((ca_wa p_re)ca_wa)(cp_ent oa_ent))(u_hdip u_hdip)))	25	12
6638	((((u_hdip oa_wa)u_csn)(cp_ent ca_ent)))((ca_wa p_re)ca_wa)(p_csn oa_ent))(u_hdip u_hdip)))	20	12
6639	((((u_isn oa_wa)u_csn)(cp_ent ca_ent)))((ca_wa p_re)ca_wa)(cp_ent oa_ent))(oa_wa u_hdip)))	21	12
6640	((((u_isn oa_wa)u_csn)(cp_ent ca_ent)))((ca_wa p_re)ca_wa)(cp_ent oa_ent))(u_hdip u_hdip)))	24	12
6641	((((u_isn oa_wa))(u_csn cp_ent)p_csn))ca_ent))((ca_wa p_re)ca_wa)((cp_ent oa_ent)u_hdip)))	21	12
6642	((((u_isn oa_wa)u_hdip)(u_csn (cp_ent ca_ent)))((ca_wa ca_wa)(cp_ent oa_ent))(u_hdip u_hdip)))	21	12
6643	((((u_isn oa_wa)u_hdip)(u_csn (cp_ent ca_ent)))((ca_wa p_re)cp_ent)((oa_ent u_isn)u_hdip)))	21	12
6644	((((u_isn oa_wa)u_hdip))(u_csn cp_ent)(p_csn (ca_ent ca_wa)))((p_re ca_wa)(cp_ent oa_ent)))	20	12
6645	((((u_isn oa_wa)u_hdip))(u_csn cp_ent)(p_csn ca_ent))))((ca_wa p_re)ca_wa)(oa_ent u_isn)))	18	12
6646	((((u_isn oa_wa)u_hdip))(u_csn cp_ent)(p_csn ca_ent))))((ca_wa p_re)cp_ent)(oa_ent u_isn)))	20	12
6647	((ca_ent ca_wa))))(p_isn p_re)(ca_wa (p_csn cp_ent)))(oa_ent (u_isn u_hdip))))(u_isn oa_wa)))	22	12
6648	((ca_ent ca_wa))))(p_isn p_re)((ca_wa (p_csn cp_ent))(oa_ent (u_isn u_hdip))))(u_isn oa_wa)))	21	12
6649	((ca_ent ca_wa))))(p_re ca_wa)(cp_ent oa_ent)))(oa_wa u_hdip)(u_isn oa_wa)))(u_csn cp_ent)))	23	12
6650	((ca_ent p_isn))))(p_re ca_wa)(cp_ent oa_ent)))(oa_wa u_hdip)(u_isn oa_wa)))(u_csn cp_ent)))	20	12
6651	((cp_ent ca_ent))((ca_wa p_re)ca_wa)))((p_csn oa_ent)(u_isn u_hdip)))((u_isn oa_wa)u_hdip))))	24	12
6652	((cp_ent ca_ent)))(p_isn p_re)((ca_wa (p_csn cp_ent)))((oa_ent u_isn)u_hdip)))(u_isn oa_wa)))	20	12
6653	((cp_ent ca_ent))))((ca_wa p_re)ca_wa)(p_csn oa_ent))(u_isn u_hdip)))(u_isn oa_wa)u_hdip)))	25	12
6654	(((cp_ent ca_ent)(ca_wa p_re))))((ca_wa (p_csn cp_ent))oa_ent)(u_isn u_hdip))(u_isn oa_wa)))	20	12
6655	(((cp_ent ca_ent)(ca_wa p_re))))((ca_wa (p_csn cp_ent))oa_ent)(u_isn u_hdip))(u_isn u_hdip)))	28	12
6656	((cp_ent ca_ent))))(ca_wa p_re)(ca_wa (p_csn cp_ent))))(oa_ent u_isn)u_hdip))(u_isn oa_wa)))	21	12
6657	((cp_ent ca_ent))))(ca_wa p_re)ca_wa)(p_csn ((p_isn oa_ent)(u_isn u_hdip))))(u_isn u_hdip)))	22	12
6658	((cp_ent ca_ent))))((ca_wa p_re)ca_wa)((cp_ent oa_ent)u_hdip))(u_isn u_hdip))(u_isn oa_wa)))	29	12
6659	((cp_ent ca_ent))))(p_re ca_wa)(cp_ent oa_ent)))(oa_wa u_hdip)(u_isn oa_wa)))(u_csn cp_ent)))	20	12
6660	((oa_wa u_hdip)))(u_csn cp_ent)(p_csn (p_isn ca_ent)))((ca_wa p_re)ca_wa)(cp_ent oa_ent))))	16	12
6661	((oa_wa u_hdip)))(u_csn cp_ent)(p_csn (p_isn ca_ent)))((ca_wa p_re)cp_ent)(oa_ent u_isn))))	16	12
6662	((oa_wa u_hdip)))(u_csn cp_ent)(p_csn (p_isn ca_ent)))((ca_wa p_re)cp_ent)(oa_ent u_hdip))))	16	12
6663	((p_isn ca_ent))))(p_isn p_re)(ca_wa (p_csn cp_ent)))((oa_ent u_isn)u_hdip))(u_isn u_hdip)))	25	12
6664	((p_isn ca_ent))((ca_wa p_re)ca_wa)))((p_csn oa_ent)(u_isn u_hdip)))((u_isn oa_wa)u_hdip))))	21	12
6665	(((p_isn ca_ent)(ca_wa p_re))))((ca_wa (p_csn cp_ent))oa_ent)(u_isn u_hdip))(u_isn u_hdip)))	24	12
6666	((p_isn ca_ent)))((ca_wa p_re)ca_wa)(p_csn ((p_isn oa_ent)(u_isn u_hdip))))(u_isn u_hdip)))	23	12
6667	((p_isn ca_ent))))((ca_wa p_re)ca_wa)((cp_ent oa_ent)u_hdip))(u_isn u_hdip))(u_isn oa_wa)))	27	12
6668	((p_isn ca_ent))))(p_re ca_wa)(cp_ent oa_ent)))(oa_wa u_hdip)(u_isn oa_wa)))(u_csn cp_ent)))	17	12
6669	(((u_csn (cp_ent ca_ent))))((ca_wa p_re)ca_wa)(p_csn oa_ent)(u_isn u_hdip))))(u_isn u_hdip)))	24	12
6670	(((u_csn (cp_ent ca_ent))))((ca_wa p_re)ca_wa)(p_csn oa_ent))(u_isn u_hdip)))(u_isn u_hdip)))	26	12
6671	(((u_csn (cp_ent ca_ent))))((ca_wa p_re)ca_wa)(p_csn (p_isn oa_ent))u_hdip))(u_isn u_hdip)))	20	12
6672	((u_csn (cp_ent ca_ent))))((ca_wa p_re)ca_wa)(p_csn oa_ent))(u_isn u_hdip))(u_isn u_hdip)))	25	12
6673	((u_csn (cp_ent ca_ent))))((ca_wa p_re)ca_wa)(cp_ent oa_ent))((oa_wa u_hdip)(u_isn oa_wa))))	21	12
6674	((u_csn (cp_ent ca_ent))))((ca_wa p_re)cp_ent)((oa_ent u_isn)u_hdip))((u_isn oa_wa)u_hdip)))	24	12
6675	((u_csn (cp_ent ca_ent))))((ca_wa p_re)cp_ent)((p_isn oa_ent)(u_isn u_hdip)))(u_isn u_hdip)))	20	12
6676	(((u_csn cp_ent)(ca_ent ca_wa))))((p_re ca_wa)p_csn)(oa_ent (u_isn u_hdip)))(u_isn u_hdip)))	23	12
6677	(((u_csn cp_ent)(ca_ent ca_wa))))((p_re ca_wa)(cp_ent oa_ent))(oa_wa u_hdip))(u_isn oa_wa)))	21	12
6678	(((u_csn cp_ent)(ca_ent ca_wa))))((p_re ca_wa)(cp_ent oa_ent))(u_hdip u_hdip))(u_isn oa_wa)))	22	12
6679	(((u_csn (cp_ent ca_ent))))((ca_wa p_re)ca_wa)(cp_ent oa_ent))(u_hdip u_hdip)))(u_isn u_hdip)))	28	12
6680	(((u_csn cp_ent)(ca_ent ca_wa))))((p_re ca_wa)(cp_ent oa_ent))(u_isn u_hdip))(u_isn oa_wa)))	25	12
6681	((u_csn (cp_ent ca_ent))))((ca_wa p_re)ca_wa)(p_csn oa_ent))(u_hdip u_hdip))(u_isn u_hdip)))	22	12
6682	((u_hdip u_hdip)))(oa_wa (u_csn cp_ent))(ca_ent ca_wa)))((p_re ca_wa)oa_ent)(u_hdip u_hdip))))	19	12
6683	((u_hdip u_hdip)))(oa_wa (u_csn cp_ent))p_csn)(ca_ent ca_wa)))((p_re ca_wa)(cp_ent oa_ent))))	20	12
6684	(((u_hdip u_hdip))(u_isn oa_wa)u_csn)(cp_ent ca_ent))))((ca_wa p_re)ca_wa)(cp_ent oa_ent)))	24	12
6685	((u_hdip u_hdip)))(u_isn oa_wa)u_csn)(cp_ent ca_ent)))((ca_wa p_re)ca_wa)(oa_ent u_isn))))	20	12
6686	((u_hdip u_hdip)))(u_isn oa_wa)u_csn)(cp_ent ca_ent)))((ca_wa p_re)cp_ent)(oa_ent u_isn))))	24	12
6687	((u_hdip u_hdip)))(u_isn oa_wa)u_csn)(cp_ent ca_wa)))(p_re ca_wa)((cp_ent oa_ent)u_hdip))))	19	12
6688	((u_isn u_hdip)))(u_isn oa_wa)u_csn)(cp_ent ca_ent))))(ca_wa p_re)ca_wa)(cp_ent oa_ent)))	21	12
6689	((u_isn u_hdip)))(u_isn oa_wa)u_csn)(cp_ent ca_ent))))(ca_wa p_re)cp_ent)(oa_ent u_isn))))	20	12
6690	((u_isn u_hdip))))(u_isn oa_wa)u_csn)(cp_ent ca_ent))))(ca_wa p_re)cp_ent)(oa_ent u_hdip))))	20	12
6691	((oa_wa (u_csn (cp_ent ca_ent))))((ca_wa p_re)ca_wa)(p_csn (oa_ent u_isn))(u_hdip u_hdip)))	23	12
6692	((oa_wa (u_csn (cp_ent ca_ent))))((ca_wa p_re)(p_csn oa_ent)(u_isn u_hdip)))(u_isn u_hdip)))	21	12
6693	((oa_wa (u_csn (cp_ent ca_ent))))((ca_wa p_re)ca_wa)(p_csn oa_ent)u_hdip))(u_isn u_hdip)))	22	12
6694	((oa_wa (u_csn (cp_ent ca_ent))))((ca_wa p_re)ca_wa)(cp_ent oa_ent))((u_isn oa_wa)u_hdip)))	25	12
6695	((oa_wa (u_csn (cp_ent ca_ent))))((ca_wa p_re)cp_ent)((oa_ent u_isn)u_hdip))(u_isn u_hdip)))	29	12
6696	((oa_wa (u_csn (cp_ent ca_ent))))((ca_wa p_re)cp_ent)oa_ent))(oa_wa u_hdip)(u_isn oa_wa))))	21	12
6697	((oa_wa (u_csn (cp_ent ca_ent))))((ca_wa p_re)cp_ent)((oa_ent u_isn)u_hdip))(u_isn oa_wa)))	25	12
6698	((oa_wa (u_csn (cp_ent ca_ent))))((ca_wa p_re)cp_ent)(oa_ent u_hdip))((u_isn oa_wa)u_hdip)))	22	12
6699	((oa_wa (u_csn (cp_ent ca_ent))))((ca_wa p_re)cp_ent)(oa_ent (u_isn u_hdip)))(u_isn oa_wa)))	24	12
6700	((oa_wa (u_csn (cp_ent ca_ent))))((ca_wa p_re)cp_ent)(oa_ent (u_isn u_hdip)))(u_isn u_hdip)))	28	12
6701	((oa_wa (u_csn (cp_ent ca_ent))))((ca_wa p_re)(p_csn oa_ent))(u_isn u_hdip))(u_isn u_hdip)))	21	12
6702	((p_csn (ca_ent ca_wa))))((p_re ca_wa)((cp_ent oa_ent)u_hdip))(u_isn u_hdip))(u_isn oa_wa)))	24	12
6703	((p_csn (p_isn ca_ent))))((ca_wa p_re)ca_wa)((cp_ent oa_ent)(u_isn u_hdip)))(u_isn u_hdip)))	18	12
6704	((((u_csn cp_ent)p_csn)(ca_ent ca_wa)))((p_re ca_wa)((cp_ent oa_ent)u_hdip))(u_isn u_hdip)))	22	12
6705	(((u_csn cp_ent)(p_csn ca_ent))))((ca_wa p_re)ca_wa)((cp_ent oa_ent)u_hdip))(u_isn u_hdip)))	23	12
6706	((((u_csn cp_ent)p_csn)ca_ent))))((ca_wa p_re)cp_ent)((oa_ent u_isn)u_hdip))(u_isn u_hdip)))	26	12
6707	((((u_csn cp_ent)p_csn)ca_ent))))((ca_wa p_re)cp_ent)oa_ent)(u_hdip u_hdip))(u_isn u_hdip)))	21	12
6708	(((u_csn (p_isn ca_ent))))((ca_wa p_re)ca_wa)(p_csn (oa_ent u_isn))u_hdip)))(u_isn u_hdip))	20	12
6709	(((u_csn cp_ent)(p_isn ca_ent))))((ca_wa p_re)ca_wa)(p_csn (p_isn oa_ent)))(u_isn u_hdip))	18	12
6710	((u_csn (p_isn ca_ent))))((ca_wa p_re)ca_wa)(p_csn oa_ent))(u_isn u_hdip))(u_isn u_hdip)))	21	12
6711	((u_csn (p_isn ca_ent))))((ca_wa p_re)cp_ent)((oa_ent u_isn)u_hdip))((u_isn oa_wa)u_hdip)))	20	12
6712	(((u_csn (p_isn ca_ent))))((ca_wa p_re)ca_wa)(cp_ent oa_ent))(u_isn u_hdip)))(u_isn u_hdip))	25	12
6713	(((u_csn cp_ent)(p_isn ca_ent))))((ca_wa p_re)cp_ent)((p_isn oa_ent)u_hdip))(u_isn u_hdip)))	18	12
6714	(((u_csn (p_isn ca_ent))))((ca_wa p_re)ca_wa)(cp_ent oa_ent))(u_hdip u_hdip)))(u_isn u_hdip))	22	12
6715	(((u_csn cp_ent)(p_isn ca_ent))))(p_re ca_wa)(cp_ent oa_ent))(u_isn u_hdip))(u_isn oa_wa)))	20	12
6716	((u_hdip (u_isn oa_wa))))((u_csn cp_ent)p_csn)ca_ent))((ca_wa p_re)ca_wa)(oa_ent u_isn)))	19	12
6717	(((oa_wa (u_csn cp_ent))(ca_ent ca_wa))))(p_re ca_wa)(p_csn (oa_ent u_isn))(u_hdip u_hdip)))	24	12
6718	(((oa_wa (u_csn cp_ent))ca_ent))((ca_wa p_re)ca_wa)((p_csn (oa_ent u_isn))(u_hdip u_hdip)))	25	12
6719	(((oa_wa (u_csn cp_ent))ca_ent))((ca_wa p_re))(p_csn oa_ent)(u_isn u_hdip)))(u_isn u_hdip)))	22	12
6720	(((oa_wa (u_csn cp_ent))(ca_ent ca_wa))))(p_re ca_wa)(p_csn (oa_ent u_hdip)))(u_isn u_hdip)))	21	12
6721	(((oa_wa (u_csn cp_ent))(ca_ent ca_wa))))(p_re ca_wa)(p_csn (p_isn oa_ent)))(u_isn u_hdip)))	19	12
6722	(((oa_wa (u_csn cp_ent))(ca_ent ca_wa))))(p_re ca_wa)((cp_ent oa_ent)oa_wa))(u_hdip u_hdip)))	22	12
6723	(((oa_wa (u_csn cp_ent))(ca_ent ca_wa))))(p_re ca_wa)((cp_ent oa_ent)oa_wa))(u_isn oa_wa)))	23	12
6724	(((oa_wa (u_csn cp_ent))(ca_ent ca_wa))))(p_re ca_wa)((cp_ent oa_ent)oa_wa))(u_isn u_hdip)))	23	12
6725	(((oa_wa (u_csn cp_ent))(ca_ent ca_wa))))(p_re ca_wa)((cp_ent oa_ent)u_hdip))(u_isn oa_wa)))	24	12
6726	(((oa_wa (u_csn cp_ent))ca_ent))((ca_wa p_re)ca_wa)((cp_ent oa_ent)u_hdip))(u_isn oa_wa)))	25	12
6727	(((oa_wa (u_csn cp_ent))(ca_ent ca_wa))))(p_re ca_wa)((cp_ent oa_ent)u_hdip))(u_isn u_hdip)))	27	12
6728	(((oa_wa (u_csn cp_ent))(ca_ent ca_wa))))(p_re ca_wa)((p_csn oa_ent)u_hdip))(u_isn u_hdip)))	23	12
6729	(((oa_wa (u_csn cp_ent))ca_ent))((ca_wa p_re)cp_ent)((oa_ent u_isn)u_hdip))(u_isn u_hdip)))	30	12
6730	(((oa_wa (u_csn cp_ent))ca_ent))((ca_wa p_re)cp_ent)oa_ent)((oa_wa u_hdip)(u_isn oa_wa))))	22	12
6731	(((oa_wa (u_csn cp_ent))ca_ent))((ca_wa p_re)cp_ent)((oa_ent u_isn)u_hdip))(u_isn oa_wa)))	26	12
6732	(((oa_wa (u_csn cp_ent))ca_ent))((ca_wa p_re)cp_ent)(oa_ent (oa_wa u_hdip)))(u_isn u_hdip)))	20	12
6733	(((oa_wa (u_csn cp_ent))ca_ent))((ca_wa p_re)cp_ent)(oa_ent (u_isn u_hdip)))(u_isn oa_wa)))	25	12
6734	(((oa_wa (u_csn cp_ent))ca_ent))((ca_wa p_re)cp_ent)(oa_ent (u_isn u_hdip)))(u_isn u_hdip)))	29	12
6735	(((oa_wa (u_csn cp_ent))ca_ent))((ca_wa p_re)(p_csn oa_ent)(u_isn u_hdip)))(u_isn u_hdip)))	22	12
6736	(((oa_wa (u_csn cp_ent))(ca_ent ca_wa))))(p_re ca_wa)oa_ent)(u_hdip u_hdip))(u_isn u_hdip)))	20	12
6737	(((oa_wa (u_csn cp_ent))(ca_ent ca_wa))))(p_re ca_wa)oa_ent)(u_isn u_hdip))(u_isn u_hdip)))	21	12
6738	(((oa_wa (u_csn cp_ent))(ca_ent ca_wa))))(p_re ca_wa)p_csn)(oa_ent u_isn))(u_hdip u_hdip)))	26	12
6739	(((oa_wa (u_csn cp_ent))(ca_ent ca_wa))))(p_re ca_wa)p_csn)(oa_ent u_hdip))(u_isn u_hdip)))	22	12
6740	(((oa_wa (u_csn cp_ent))(ca_ent ca_wa))((p_re ca_wa)(p_csn ((p_isn oa_ent)(u_isn u_hdip))))	17	12
6741	(((oa_wa u_csn)(cp_ent ca_wa))))(p_re ca_wa)(cp_ent oa_ent))(oa_wa u_hdip))(u_isn oa_wa)))	19	12
6742	(((oa_wa u_csn)(cp_ent ca_wa))))(p_re ca_wa)(cp_ent oa_ent))(oa_wa u_hdip))(u_isn u_hdip)))	22	12
6743	(((oa_wa u_csn)(cp_ent ca_wa))))(p_re ca_wa)(cp_ent oa_ent))(u_hdip u_hdip))(u_isn u_hdip)))	24	12
6744	(((oa_wa u_csn)(cp_ent ca_wa))))(p_re ca_wa)(cp_ent oa_ent))(u_isn u_hdip))(u_isn oa_wa)))	23	12
6745	(((oa_wa u_csn)(cp_ent ca_wa))))(p_re ca_wa)(cp_ent oa_ent))(u_isn u_hdip))(u_isn u_hdip)))	21	12
6746	(((oa_wa (u_csn cp_ent))(p_csn ca_ent))))((ca_wa p_re)ca_wa)(cp_ent oa_ent))(u_hdip u_hdip)))	24	12
6747	(((oa_wa (u_csn cp_ent))(p_csn ca_ent))))((ca_wa p_re)ca_wa)(oa_ent u_isn))(u_hdip u_hdip)))	19	12
6748	(((oa_wa (u_csn cp_ent))(p_csn ca_ent))))((ca_wa p_re)cp_ent)(oa_ent u_hdip))(u_isn u_hdip)))	24	12
6749	(((oa_wa (u_csn cp_ent))(p_csn ca_ent))))((ca_wa p_re)cp_ent)(oa_ent u_isn))(u_hdip u_hdip)))	24	12
6750	(((oa_wa u_csn)(p_isn ca_ent))))((ca_wa p_re)((p_csn oa_ent)(u_isn u_hdip)))(u_isn u_hdip)))	17	12
6751	(((oa_wa u_csn)(p_isn ca_ent))))((ca_wa p_re)ca_wa)((p_csn oa_ent)u_hdip))(u_isn u_hdip)))	18	12
6752	(((oa_wa (u_csn cp_ent))(p_isn ca_ent))))((ca_wa p_re)ca_wa)(cp_ent oa_ent))(u_isn oa_wa)))	20	12
6753	(((oa_wa u_csn)(p_isn ca_ent))))((ca_wa p_re)cp_ent)((oa_ent u_isn)u_hdip))(u_isn u_hdip)))	23	12
6754	(((oa_wa (u_csn cp_ent))(p_isn ca_ent))))((ca_wa p_re)cp_ent)oa_ent)((u_isn oa_wa)u_hdip)))	20	12
6755	((((oa_wa (u_csn cp_ent))(p_isn ca_ent))((ca_wa p_re)cp_ent)(oa_ent oa_wa))(u_isn oa_wa)))	18	12
6756	(((oa_wa u_csn)(p_isn ca_ent))))((ca_wa p_re)cp_ent)((oa_ent u_isn)u_hdip))(u_isn oa_wa)))	19	12
6757	(((oa_wa u_csn)(p_isn ca_ent))))((ca_wa p_re)cp_ent)(oa_ent (u_isn u_hdip)))(u_isn oa_wa)))	18	12
6758	(((oa_wa u_csn)(p_isn ca_ent))))((p_re ca_wa)p_csn)oa_ent)(u_isn u_hdip)))(u_isn u_hdip)))	19	12
6759	((p_isn ca_ent)(ca_wa p_re))))(ca_wa (p_csn cp_ent))((oa_ent u_isn)u_hdip))(u_isn oa_wa)))	17	12
6760	(((u_csn cp_ent)(ca_ent ca_wa))))(p_re ca_wa)(cp_ent oa_ent))(u_hdip u_hdip))(u_isn u_hdip)))	27	12
6761	(((u_csn cp_ent)(ca_ent ca_wa))))(p_re ca_wa)(cp_ent oa_ent))(u_isn u_hdip))(u_isn u_hdip)))	27	12
6762	(((u_csn cp_ent)(p_isn ca_ent))))((ca_wa p_re)((p_csn oa_ent)(u_isn u_hdip)))(u_isn u_hdip)))	20	12
6763	(((u_csn cp_ent)(p_isn ca_ent))))((ca_wa p_re)ca_wa)((cp_ent oa_ent)oa_wa))(u_isn oa_wa)))	21	12
6764	(((u_csn cp_ent)(p_isn ca_ent))))((ca_wa p_re)ca_wa)((cp_ent oa_ent)u_hdip))(u_isn oa_wa)))	22	12
6765	(((u_csn cp_ent)(p_isn ca_ent))))((ca_wa p_re)ca_wa)(cp_ent oa_ent)))(u_isn oa_wa)u_hdip)))	22	12
6766	(((u_csn cp_ent)(p_isn ca_ent))))((ca_wa p_re)cp_ent)((oa_ent u_isn)u_hdip))(u_isn u_hdip)))	27	12
6767	(((u_csn cp_ent)(p_isn ca_ent))))((ca_wa p_re)cp_ent)(oa_ent u_hdip))((u_isn oa_wa)u_hdip)))	21	12
6768	(((u_csn cp_ent)(p_isn ca_ent))))((ca_wa p_re)cp_ent)((oa_ent u_isn)u_hdip))(u_isn oa_wa)))	23	12
6769	(((u_csn cp_ent)(p_isn ca_ent))))((ca_wa p_re)cp_ent)(oa_ent (u_isn u_hdip)))(u_isn oa_wa)))	22	12
6770	(((u_csn cp_ent)(p_isn ca_ent))))((ca_wa p_re)(p_csn oa_ent))(u_isn u_hdip)))(u_isn u_hdip)))	21	12
6771	(((u_csn cp_ent)(p_isn ca_ent))))((p_re ca_wa)p_csn)oa_ent)(u_isn u_hdip)))(u_isn u_hdip)))	23	12
6772	(((u_csn cp_ent)(p_isn ca_ent))))(p_re ca_wa)(cp_ent oa_ent))(u_isn u_hdip))(u_isn u_hdip)))	24	12
6773	(((u_csn cp_ent)(p_isn ca_wa))))(p_re ca_wa)(cp_ent oa_ent))(oa_wa u_hdip))(u_isn oa_wa)))	18	12
6774	(((u_csn cp_ent)(p_isn ca_wa))))(p_re ca_wa)(cp_ent oa_ent))(u_hdip u_hdip))(u_isn oa_wa)))	20	12
6775	(((u_csn cp_ent)(p_isn ca_wa))))(p_re ca_wa)(cp_ent oa_ent))(u_hdip u_hdip))(u_isn u_hdip)))	23	12
6776	(((u_csn cp_ent)(p_isn ca_wa))))(p_re ca_wa)(cp_ent oa_ent))(u_isn u_hdip))(u_isn oa_wa)))	22	12
6777	(((u_hdip oa_wa)u_hdip)))(u_csn cp_ent)p_csn)))((ca_wa p_re)cp_ent)((oa_ent u_isn)u_hdip))))	17	12
6778	(((u_hdip (u_csn cp_ent))(ca_ent ca_wa))))(p_re ca_wa)((cp_ent oa_ent)oa_wa))(u_isn u_hdip)))	20	12
6779	(((u_hdip (u_csn cp_ent))(ca_ent ca_wa))))(p_re ca_wa)((cp_ent oa_ent)u_hdip))(u_isn oa_wa)))	21	12
6780	(((u_hdip (u_csn cp_ent))(ca_ent ca_wa))))(p_re ca_wa)((cp_ent oa_ent)u_hdip))(u_isn u_hdip)))	24	12
6781	(((u_hdip (u_csn cp_ent))(ca_ent ca_wa))))(p_re ca_wa)oa_ent)(u_hdip u_hdip))(u_isn u_hdip)))	18	12
6782	(((u_hdip u_csn)(cp_ent ca_ent))))((ca_wa p_re)cp_ent)((oa_ent u_isn)u_hdip))(u_isn u_hdip)))	25	12
6783	(((u_hdip u_csn)(cp_ent ca_ent))))((ca_wa p_re)cp_ent)((oa_ent u_isn)u_hdip))(u_isn oa_wa)))	20	12
6784	(((u_hdip u_csn)(cp_ent ca_wa))))(p_re ca_wa)(cp_ent oa_ent))(u_hdip u_hdip))(u_isn u_hdip)))	21	12
6785	(((u_isn cp_ent)(ca_ent ca_wa))))(p_re ca_wa)p_csn)oa_ent)(u_isn u_hdip)))(u_isn u_hdip)))	19	12
6786	(((u_isn cp_ent)(p_isn ca_ent))))((ca_wa p_re)ca_wa)(cp_ent oa_ent))((u_isn oa_wa)u_hdip)))	18	12
6787	(((u_isn cp_ent)(p_isn ca_ent))))((ca_wa p_re)cp_ent)((oa_ent u_isn)u_hdip)))(u_isn u_hdip)))	20	12
6788	(((u_isn cp_ent)(p_isn ca_ent))))((ca_wa p_re)cp_ent)(oa_ent (u_isn u_hdip)))(u_isn u_hdip)))	19	12
6789	(((u_isn oa_wa)u_hdip))))(u_csn cp_ent)p_csn)ca_ent))((ca_wa p_re)cp_ent)(oa_ent u_isn))))	19	12
6790	(((u_isn oa_wa)u_hdip))))(u_csn cp_ent)p_csn)ca_ent))((ca_wa p_re)ca_wa)(cp_ent oa_ent))))	21	12
6791	(((u_isn oa_wa)u_hdip))))(u_csn cp_ent)(p_csn (p_isn ca_ent))))((ca_wa p_re)ca_wa)oa_ent)))	14	12
6792	(((u_isn oa_wa)u_hdip))))(u_csn cp_ent)p_csn)ca_ent))((ca_wa p_re)ca_wa)(oa_ent u_isn))))	18	12
6793	(((u_isn oa_wa)u_hdip))))(u_isn oa_wa)(u_csn cp_ent)p_csn))ca_ent)((ca_wa p_re)ca_wa)))	17	12
6794	(((u_isn u_csn)(cp_ent ca_ent))))((ca_wa p_re)cp_ent)((oa_ent u_isn)u_hdip))(u_isn u_hdip)))	23	12
6795	(((u_isn u_csn)(cp_ent ca_ent))))((ca_wa p_re)cp_ent)((oa_ent u_isn)u_hdip))(u_isn oa_wa)))	19	12
6796	(((u_isn u_csn)(ca_ent ca_wa))))(p_re ca_wa)(cp_ent oa_ent))(u_hdip u_hdip))(u_isn u_hdip)))	19	12
6797	(((u_isn u_csn)(cp_ent ca_wa))))(p_re ca_wa)(cp_ent oa_ent))(u_hdip u_hdip))(u_isn u_hdip)))	17	12
6798	(((oa_wa u_csn)(p_csn (p_isn ca_ent)))(((ca_wa p_re)ca_wa)(cp_ent oa_ent))(u_hdip u_hdip)))	17	12
6799	(((oa_wa u_hdip)))(u_csn cp_ent)p_csn)ca_ent))((ca_wa p_re)ca_wa)oa_ent)(u_hdip u_hdip)))	18	12
6800	(((oa_wa u_hdip)))(u_csn cp_ent)p_csn)ca_ent))((ca_wa p_re)cp_ent)oa_ent)(u_isn u_hdip)))	20	12
6801	(((u_csn cp_ent)(p_csn (p_isn ca_ent))))((ca_wa p_re)ca_wa)(cp_ent oa_ent))(u_hdip u_hdip)))	20	12
6802	(((u_csn cp_ent)(p_csn (p_isn ca_ent))))((ca_wa p_re)ca_wa)(oa_ent u_isn))(u_hdip u_hdip)))	17	12
6803	(((u_hdip oa_wa)(u_csn (cp_ent ca_ent))))((ca_wa p_re)ca_wa)(oa_ent u_isn))(u_hdip u_hdip)))	20	12
6804	(((u_hdip oa_wa)(u_csn (cp_ent ca_ent))))((ca_wa p_re)cp_ent)(oa_ent u_hdip))(u_isn u_hdip)))	24	12
6805	(((u_hdip oa_wa)(u_csn (cp_ent ca_ent))))((ca_wa p_re)cp_ent)(oa_ent u_isn))(u_hdip u_hdip)))	24	12
6806	(((u_hdip oa_wa)))(u_csn cp_ent)p_csn)ca_ent))((ca_wa p_re)ca_wa)oa_ent)(u_hdip u_hdip)))	17	12
6807	(((u_hdip oa_wa)))(u_csn cp_ent)p_csn)ca_ent))((ca_wa p_re)cp_ent)oa_ent)(u_isn u_hdip)))	20	12
6808	(((u_hdip u_hdip))(u_csn cp_ent)(p_csn ca_ent))))((ca_wa p_re)ca_wa)oa_ent)(u_hdip u_hdip)))	16	12
6809	(((u_hdip u_hdip))(u_csn cp_ent)(p_csn ca_ent))))((ca_wa p_re)cp_ent)oa_ent)(u_isn u_hdip)))	17	12
6810	(((u_hdip u_hdip))(oa_wa (u_csn cp_ent))ca_ent))((ca_wa p_re)ca_wa)oa_ent)(u_hdip u_hdip)))	21	12
6811	(((u_hdip u_hdip))(oa_wa (u_csn cp_ent))ca_ent))((ca_wa p_re)cp_ent)oa_ent)(u_isn u_hdip)))	25	12
6812	(((u_hdip u_hdip))(oa_wa (u_csn cp_ent))ca_ent))((ca_wa p_re)cp_ent)((oa_ent oa_wa)u_hdip)))	20	12
6813	(((oa_wa (u_csn cp_ent))(p_isn ca_ent))((ca_wa p_re)(p_csn ((p_isn oa_ent)(u_isn u_hdip))))	15	12
6814	(((oa_wa (u_csn cp_ent))(p_isn ca_ent))((ca_wa p_re)(p_csn (oa_ent u_hdip)))(u_isn u_hdip)))	16	12
6815	(((oa_wa (u_csn cp_ent))(p_isn ca_ent))((p_re ca_wa)((cp_ent oa_ent)oa_wa))(u_isn u_hdip)))	18	12
6816	(((oa_wa (u_csn cp_ent))(p_isn ca_ent))((ca_wa p_re)ca_wa)(cp_ent oa_ent))(oa_wa u_hdip)))	21	12
6817	(((oa_wa (u_csn cp_ent))(p_isn ca_ent))((ca_wa p_re)ca_wa)(cp_ent oa_ent))(u_hdip u_hdip)))	24	12
6818	(((oa_wa (u_csn cp_ent))(p_isn ca_ent))((ca_wa p_re)ca_wa)(cp_ent oa_ent))(u_isn u_hdip)))	25	12
6819	(((oa_wa (u_csn cp_ent))(p_isn ca_ent))((ca_wa p_re)ca_wa)(oa_ent u_isn))(u_hdip u_hdip)))	19	12
6820	(((oa_wa (u_csn cp_ent))(p_isn ca_ent))((ca_wa p_re)cp_ent)(oa_ent oa_wa))(u_isn oa_wa)))	19	12
6821	(((oa_wa (u_csn cp_ent))(p_isn ca_ent))((ca_wa p_re)cp_ent)(oa_ent oa_wa))(u_isn u_hdip)))	20	12
6822	(((oa_wa (u_csn cp_ent))(p_isn ca_ent))((ca_wa p_re)cp_ent)(oa_ent u_hdip))(u_isn oa_wa)))	20	12
6823	(((oa_wa (u_csn cp_ent))(p_isn ca_wa))((p_re ca_wa)((cp_ent oa_ent)oa_wa))(u_hdip u_hdip)))	20	12
6824	(((oa_wa (u_csn cp_ent))(p_isn ca_wa))((p_re ca_wa)((cp_ent oa_ent)oa_wa))(u_isn oa_wa)))	20	12
6825	(((oa_wa (u_csn cp_ent))(p_isn ca_wa))((p_re ca_wa)((cp_ent oa_ent)oa_wa))(u_isn u_hdip)))	20	12
6826	(((oa_wa (u_csn cp_ent))(p_isn ca_wa))((p_re ca_wa)((cp_ent oa_ent)u_hdip))(u_isn oa_wa)))	21	12
6827	(((oa_wa (u_csn cp_ent))(p_isn ca_wa))((p_re ca_wa)oa_ent)(u_hdip u_hdip))(u_isn u_hdip)))	17	12
6828	(((oa_wa (u_csn cp_ent))(p_isn ca_wa))((p_re ca_wa)oa_ent)(u_isn u_hdip))(u_isn oa_wa)))	16	12
6829	(((oa_wa (u_csn cp_ent))(p_csn (p_isn ca_ent))))((ca_wa p_re)cp_ent)oa_ent)(u_isn u_hdip)))	19	12
6830	(((p_csn oa_ent)u_hdip)(u_isn oa_wa))))((u_csn cp_ent)p_csn)(ca_ent p_isn))(p_re ca_wa)))	24	12
6831	(((u_csn (u_isn cp_ent))ca_ent))((ca_wa p_re)ca_wa)(cp_ent oa_ent)))(u_isn oa_wa)u_hdip)))	19	12
6832	(((u_csn (u_isn cp_ent))ca_ent))((ca_wa p_re)cp_ent)((oa_ent u_isn)u_hdip)))(u_isn u_hdip))	22	12
6833	(((u_csn (u_isn cp_ent))ca_ent))((ca_wa p_re)cp_ent)((oa_ent u_isn)u_hdip))(u_isn oa_wa)))	18	12
6834	(((u_csn (u_isn cp_ent))(p_isn ca_ent))((ca_wa p_re)ca_wa)(cp_ent oa_ent))(u_hdip u_hdip)))	20	12
6835	(((u_csn (u_isn cp_ent))(p_isn ca_ent))((ca_wa p_re)ca_wa)(cp_ent oa_ent))(u_isn oa_wa)))	17	12
6836	(((u_hdip (oa_wa u_csn))(cp_ent ca_wa))((p_re ca_wa)((cp_ent oa_ent)oa_wa)))(u_hdip u_hdip))	17	12
6837	(((u_hdip (oa_wa u_csn))(ca_ent ca_wa))((p_re ca_wa)((cp_ent oa_ent)oa_wa))(u_isn u_hdip)))	18	12
6838	(((u_hdip (oa_wa u_csn))(cp_ent ca_ent))((ca_wa p_re)cp_ent)(oa_ent u_hdip))(u_isn u_hdip)))	22	12
6839	(((u_hdip (oa_wa u_csn))(cp_ent ca_ent))((ca_wa p_re)cp_ent)(oa_ent u_isn))(u_hdip u_hdip)))	22	12
6840	(((u_hdip (oa_wa u_csn))(cp_ent ca_ent))((ca_wa p_re)cp_ent)(oa_ent u_hdip))(u_isn oa_wa)))	18	12
6841	(((u_hdip (oa_wa u_csn))(cp_ent ca_wa))((p_re ca_wa)((cp_ent oa_ent)oa_wa))(u_isn u_hdip)))	17	12
6842	(((u_hdip (oa_wa u_csn))(cp_ent ca_wa))((p_re ca_wa)((cp_ent oa_ent)u_hdip))(u_isn oa_wa)))	18	12
6843	(((u_hdip (u_csn cp_ent))(p_csn ca_ent))((ca_wa p_re)ca_wa)(oa_ent u_isn))(u_hdip u_hdip)))	18	12
6844	(((u_hdip (u_csn cp_ent))(p_csn ca_ent))((ca_wa p_re)cp_ent)(oa_ent u_hdip))(u_isn u_hdip)))	20	12
6845	(((u_hdip (u_csn cp_ent))(p_csn ca_ent))((ca_wa p_re)cp_ent)(oa_ent u_isn))(u_hdip u_hdip)))	20	12
6846	(((u_hdip (u_csn cp_ent))(p_isn ca_ent))((ca_wa p_re)ca_wa)(oa_ent u_isn))(u_hdip u_hdip)))	18	12
6847	(((u_hdip (u_csn cp_ent))(p_isn ca_wa))((p_re ca_wa)((cp_ent oa_ent)oa_wa))(u_isn u_hdip)))	19	12
6848	(((u_hdip (u_csn cp_ent))(p_isn ca_wa))((p_re ca_wa)((cp_ent oa_ent)u_hdip))(u_isn u_hdip)))	22	12
6849	(((u_hdip (u_csn cp_ent))(p_isn ca_wa))((p_re ca_wa)oa_ent)(u_hdip u_hdip))(u_isn u_hdip)))	15	12
6850	(((u_hdip (u_csn cp_ent))(p_csn (p_isn ca_ent))))((ca_wa p_re)ca_wa)oa_ent)(u_hdip u_hdip)))	15	12
6851	(((u_hdip (u_csn cp_ent))(p_csn (p_isn ca_ent))))((ca_wa p_re)cp_ent)oa_ent)(u_isn u_hdip)))	16	12
6852	(((u_hdip (u_isn oa_wa))(u_csn (cp_ent ca_ent))))((ca_wa p_re)ca_wa)oa_ent)(u_hdip u_hdip)))	20	12
6853	(((u_hdip (u_isn oa_wa))(u_csn (cp_ent ca_ent))))((ca_wa p_re)cp_ent)oa_ent)(u_isn oa_wa)))	19	12
6854	(((u_hdip (u_isn oa_wa))(u_csn (cp_ent ca_ent))))((ca_wa p_re)cp_ent)oa_ent)(u_isn u_hdip)))	24	12
6855	(((u_hdip (u_isn u_csn))(cp_ent ca_ent))((ca_wa p_re)ca_wa)(cp_ent oa_ent))(u_hdip u_hdip)))	22	12
6856	(((u_hdip (u_isn u_csn))(cp_ent ca_ent))((ca_wa p_re)cp_ent)(oa_ent u_hdip))(u_isn u_hdip)))	22	12
6857	(((u_hdip (u_isn u_csn))(cp_ent ca_ent))((ca_wa p_re)cp_ent)(oa_ent u_isn))(u_hdip u_hdip)))	22	12
6858	(((u_hdip (u_isn u_csn))(cp_ent ca_wa))((p_re ca_wa)((cp_ent oa_ent)oa_wa))(u_hdip u_hdip)))	16	12
6859	(((u_hdip (u_isn u_csn))(cp_ent ca_wa))((p_re ca_wa)((cp_ent oa_ent)oa_wa))(u_isn u_hdip)))	16	12
6860	(((u_hdip (u_isn u_csn))(cp_ent ca_wa))((p_re ca_wa)((cp_ent oa_ent)u_hdip))(u_isn oa_wa)))	17	12
6861	(((oa_wa u_csn)p_csn)ca_ent))((ca_wa p_re)cp_ent)((oa_ent u_isn)u_hdip))(u_isn u_hdip)))	23	12
6862	(((oa_wa u_csn)p_csn)(p_isn ca_ent))((ca_wa p_re)ca_wa)(cp_ent oa_ent))(u_hdip u_hdip)))	19	12
6863	(((oa_wa u_hdip)oa_wa)(u_csn (cp_ent ca_ent))))((ca_wa p_re)cp_ent)oa_ent)(u_isn oa_wa)))	21	12
6864	((((p_csn oa_ent)(u_isn u_hdip))(u_isn oa_wa)))((u_csn cp_ent)p_csn)ca_ent)(ca_wa ca_wa)))	24	12
6865	((((p_csn oa_ent)(u_isn u_hdip))(u_isn oa_wa)))((u_csn cp_ent)p_csn)ca_ent)(ca_wa p_re)))	24	12
6866	((((u_csn cp_ent)p_csn)(p_isn ca_ent))((ca_wa p_re)ca_wa)(cp_ent oa_ent))(u_hdip u_hdip)))	22	12
6867	((((u_csn cp_ent)p_csn)(p_isn ca_ent))((ca_wa p_re)ca_wa)(oa_ent u_isn))(u_hdip u_hdip)))	19	12
6868	(((u_hdip oa_wa)u_csn)(cp_ent ca_ent))((ca_wa p_re)(p_csn ((p_isn oa_ent)(u_isn u_hdip))))	16	12
6869	(((u_hdip oa_wa)u_csn)(ca_ent ca_wa))((p_re ca_wa)(p_csn ((p_isn oa_ent)(u_isn u_hdip))))	16	12
6870	((((u_hdip oa_wa)u_csn)(ca_ent ca_wa))((p_re ca_wa)(p_csn (oa_ent u_isn)))(u_hdip u_hdip)))	20	12
6871	((((u_hdip oa_wa)u_csn)(cp_ent ca_ent))((ca_wa p_re)ca_wa)((p_csn (p_isn oa_ent))u_hdip)))	18	12
6872	((((u_hdip oa_wa)u_csn)(cp_ent ca_ent))((ca_wa p_re)(p_csn (oa_ent u_hdip)))(u_isn u_hdip)))	17	12
6873	((((u_hdip oa_wa)u_csn)(ca_ent ca_wa))((p_re ca_wa)((p_csn oa_ent)u_hdip))(u_isn u_hdip)))	19	12
6874	((((u_hdip oa_wa)u_csn)(cp_ent ca_wa))((p_re ca_wa)((cp_ent oa_ent)oa_wa)))(u_hdip u_hdip))	20	12
6875	((((u_hdip oa_wa)u_csn)(ca_ent ca_wa))((p_re ca_wa)((cp_ent oa_ent)oa_wa))(u_isn u_hdip)))	21	12
6876	((((u_hdip oa_wa)u_csn)(ca_ent ca_wa))((p_re ca_wa)((cp_ent oa_ent)u_hdip))(u_isn u_hdip)))	24	12
6877	((((u_hdip oa_wa)u_csn)(cp_ent ca_ent))((ca_wa p_re)ca_wa)(oa_ent u_isn))(u_hdip u_hdip)))	21	12
6878	((((u_hdip oa_wa)u_csn)(cp_ent ca_ent))((ca_wa p_re)cp_ent)(oa_ent u_hdip))(u_isn u_hdip)))	25	12
6879	((((u_hdip oa_wa)u_csn)(cp_ent ca_ent))((ca_wa p_re)cp_ent)(oa_ent u_isn))(u_hdip u_hdip)))	25	12
6880	((((u_hdip oa_wa)u_csn)(ca_ent ca_wa))((p_re ca_wa)oa_ent)(u_hdip u_hdip))(u_isn u_hdip)))	18	12
6881	((((u_hdip oa_wa)u_csn)(ca_ent ca_wa))((p_re ca_wa)oa_ent)(u_isn u_hdip))(u_isn u_hdip)))	19	12
6882	((((u_hdip oa_wa)u_csn)(ca_ent ca_wa))((p_re ca_wa)p_csn)(oa_ent u_isn))(u_hdip u_hdip)))	21	12
6883	((((u_hdip oa_wa)u_csn)(ca_ent ca_wa))((p_re ca_wa)p_csn)(oa_ent u_hdip))(u_isn u_hdip)))	20	12
6884	((((u_hdip oa_wa)u_csn)(cp_ent ca_wa))((p_re ca_wa)((cp_ent oa_ent)oa_wa))(u_isn u_hdip)))	20	12
6885	((((u_hdip oa_wa)u_csn)(cp_ent ca_wa))((p_re ca_wa)((cp_ent oa_ent)u_hdip))(u_isn u_hdip)))	20	12
6886	((((u_hdip oa_wa)u_csn)(cp_ent ca_wa))((p_re ca_wa)oa_ent)(u_hdip u_hdip))(u_isn u_hdip)))	16	12
6887	((((u_isn oa_wa)u_csn)(cp_ent ca_ent))((ca_wa p_re)ca_wa)(oa_ent u_isn))(u_hdip u_hdip)))	21	12
6888	((((u_isn oa_wa)u_csn)(cp_ent ca_ent))((ca_wa p_re)cp_ent)(oa_ent u_hdip))(u_isn u_hdip)))	24	12
6889	((((u_isn oa_wa)u_csn)(cp_ent ca_ent))((ca_wa p_re)cp_ent)(oa_ent u_isn))(u_hdip u_hdip)))	24	12
6890	((((u_isn oa_wa)u_csn)(cp_ent ca_wa))((p_re ca_wa)((cp_ent oa_ent)oa_wa))(u_isn u_hdip)))	19	12
6891	((((u_isn oa_wa)u_csn)(cp_ent ca_wa))((p_re ca_wa)((cp_ent oa_ent)u_hdip))(u_isn u_hdip)))	19	12
6892	((((u_isn oa_wa)u_hdip)(u_csn (cp_ent ca_ent))))((ca_wa p_re)ca_wa)oa_ent)(u_hdip u_hdip)))	18	12
6893	((((u_isn oa_wa)u_hdip)(u_csn (cp_ent ca_ent))))((ca_wa p_re)cp_ent)oa_ent)(u_isn u_hdip)))	21	12
6894	((((u_isn u_hdip)(oa_wa (u_csn cp_ent)))ca_ent))((ca_wa p_re)cp_ent)oa_ent)(u_isn u_hdip)))	20	12
6895	((ca_ent ca_wa))))(p_re ca_wa)(cp_ent oa_ent)))(oa_wa u_hdip)(u_isn oa_wa)))(u_csn cp_ent))	25	12
6896	(((cp_ent ca_ent))((ca_wa p_re)ca_wa)(p_csn ((p_isn oa_ent)(u_isn u_hdip))))(u_isn u_hdip))	20	12
6897	((ca_ent ca_wa))))(p_isn p_re)(ca_wa (p_csn cp_ent)))((oa_ent u_isn)u_hdip))(u_isn oa_wa)))	25	12
6898	((cp_ent ca_ent))((ca_wa p_re)ca_wa)((p_csn oa_ent)(u_isn u_hdip)))((u_isn oa_wa)u_hdip))	25	12
6899	(((cp_ent ca_ent))((ca_wa p_re)cp_ent)((oa_ent (u_isn u_hdip))))((oa_wa u_hdip)(u_isn oa_wa)))	26	12
6900	(((cp_ent ca_ent))((ca_wa p_re)cp_ent)((oa_ent u_isn)u_hdip))))(oa_wa u_hdip)(u_isn oa_wa)))	28	12
6901	((((cp_ent oa_ent)(oa_wa u_hdip))(u_isn u_hdip))(u_isn oa_wa)u_csn))))(oa_ent oa_wa)u_hdip))	16	12
6902	((((cp_ent oa_ent)oa_wa)(u_isn u_hdip))(u_isn u_hdip)(oa_wa (u_csn cp_ent)))))(oa_ent oa_wa))	17	12
6903	(((p_isn ca_ent))((ca_wa p_re)ca_wa)((p_csn oa_ent)(u_isn u_hdip))))(u_isn oa_wa)u_hdip))	23	12
6904	(((p_isn ca_ent))((ca_wa p_re)cp_ent)(oa_ent (u_isn u_hdip))))((oa_wa u_hdip)(u_isn oa_wa)))	23	12
6905	(((p_isn ca_ent))((ca_wa p_re)cp_ent)((oa_ent u_isn)u_hdip))))(oa_wa u_hdip)(u_isn oa_wa)))	25	12
6906	(((u_csn (cp_ent ca_ent))))(ca_wa p_re)ca_wa)(cp_ent oa_ent)))((oa_wa u_hdip)(u_isn oa_wa)))	24	12
6907	(((u_csn (cp_ent ca_ent))))(ca_wa p_re)ca_wa)(cp_ent oa_ent))(u_isn u_hdip)))(u_isn u_hdip))	30	12
6908	(((u_csn (cp_ent ca_ent))((ca_wa p_re)cp_ent)(oa_ent (u_isn u_hdip))))((u_isn oa_wa)u_hdip))	23	12
6909	(((u_csn (cp_ent ca_ent))((ca_wa p_re)cp_ent)((oa_ent u_isn)u_hdip))))(u_isn oa_wa)u_hdip))	25	12
6910	(((u_hdip u_hdip))(u_isn oa_wa)u_csn)(cp_ent ca_ent))))((ca_wa p_re)ca_wa)(oa_ent u_isn)))	21	12
6911	(((u_hdip u_hdip))(u_isn oa_wa)u_csn)(cp_ent ca_wa))))(p_re ca_wa)((cp_ent oa_ent)oa_wa)))	17	12
6912	(((u_hdip u_hdip))(u_isn oa_wa)u_csn)(cp_ent ca_wa))))(p_re ca_wa)((cp_ent oa_ent)u_hdip)))	18	12
6913	(((u_isn oa_wa))(u_hdip (u_csn cp_ent))(p_csn (ca_ent ca_wa))))(p_re ca_wa)(cp_ent oa_ent)))	19	12
6914	(((u_isn oa_wa))(u_hdip (u_csn cp_ent))(p_csn ca_ent))))((ca_wa p_re)ca_wa)(oa_ent u_isn)))	17	12
6915	(((u_isn oa_wa))(u_hdip (u_csn cp_ent))(p_csn ca_ent))))((ca_wa p_re)cp_ent)(oa_ent u_isn)))	19	12

6916	(((oa_wa (u_csn (cp_ent ca_ent)))((ca_wa p_re)((p_csn oa_ent)(u_isn u_hdip))))(u_isn u_hdip))	20	12
6917	(((oa_wa (u_csn (cp_ent ca_ent)))((ca_wa p_re) ca_wa)(cp_ent oa_ent)))(u_hdip (u_isn oa_wa)))	26	12
6918	(((oa_wa (u_csn (cp_ent ca_ent)))((ca_wa p_re) cp_ent) oa_ent)))(oa_wa u_hdip)(u_isn oa_wa)))	22	12
6919	(((oa_wa (u_csn (cp_ent ca_ent)))((ca_wa p_re) cp_ent)(oa_ent u_isn)))((oa_wa u_hdip) oa_wa))	24	12
6920	(((oa_wa (u_csn (cp_ent ca_ent)))((ca_wa p_re) cp_ent)((oa_ent u_isn) u_hdip)))(u_isn u_hdip))	28	12
6921	((((u_csn cp_ent) p_csn) ca_ent)((ca_wa p_re) cp_ent)((oa_ent u_isn) u_hdip)))(u_isn u_hdip))	25	12
6922	(((oa_wa (u_csn cp_ent)) ca_ent)((ca_wa p_re)((p_csn oa_ent)(u_isn u_hdip))))(u_isn u_hdip))	21	12
6923	((((oa_wa (u_csn cp_ent)) ca_ent)((ca_wa p_re) ca_wa)(cp_ent oa_ent)))((oa_wa u_hdip) oa_wa))	25	12
6924	((((oa_wa (u_csn cp_ent)) ca_ent)((ca_wa p_re) cp_ent) oa_ent)))(oa_wa u_hdip)(u_isn oa_wa)))	23	12
6925	((((oa_wa (u_csn cp_ent)) ca_ent)((ca_wa p_re) cp_ent)((oa_ent u_isn) u_hdip)))(u_isn u_hdip))	29	12
6926	((((oa_wa (u_csn cp_ent))((ca_ent ca_wa)))((p_re ca_wa) p_csn)(oa_ent u_isn)))(u_hdip u_hdip))	25	12
6927	((((oa_wa u_csn)(cp_ent ca_wa)))((p_re ca_wa)((cp_ent oa_ent)(oa_wa u_hdip))))(u_isn u_hdip))	23	12
6928	((((oa_wa u_csn)(cp_ent ca_wa)))((p_re ca_wa)(cp_ent oa_ent)((u_isn u_hdip)))(u_isn u_hdip))	22	12
6929	((((oa_wa u_hdip) oa_wa)((u_csn cp_ent) p_csn) ca_ent)))((ca_wa p_re) cp_ent)(oa_ent u_isn)))	18	12
6930	((((u_csn cp_ent)(p_isn ca_ent)))((ca_wa p_re) cp_ent)(oa_ent oa_wa)))((u_isn oa_wa) u_hdip))	19	12
6931	((((u_hdip u_csn)(cp_ent ca_ent)))((ca_wa p_re) cp_ent)((oa_ent u_isn) u_hdip)))(u_isn u_hdip))	24	12
6932	(((u_isn oa_wa) u_hdip)))((u_csn cp_ent) p_csn) ca_ent)))((ca_wa p_re) ca_wa)(oa_ent u_isn)))	20	12
6933	(((u_isn oa_wa) u_hdip)))((u_csn cp_ent) p_csn) ca_ent)))((ca_wa p_re) cp_ent)(oa_ent u_isn)))	21	12
6934	(((u_isn oa_wa)((u_csn cp_ent) p_csn)) ca_ent)((ca_wa p_re) cp_ent)((oa_ent u_isn) u_hdip)))	21	12
6935	((((u_isn oa_wa)((u_csn cp_ent) p_csn)) ca_ent)(((ca_wa p_re) ca_wa) oa_ent)(u_hdip u_hdip)))	18	12
6936	(((u_isn oa_wa)((u_csn cp_ent) p_csn)) ca_ent)(((ca_wa p_re) cp_ent) oa_ent)(u_isn u_hdip)))	21	12
6937	((((u_isn oa_wa) u_hdip)))((u_isn oa_wa) u_csn)(cp_ent ca_ent)))((ca_wa p_re) cp_ent) oa_ent))	19	12
6938	(((p_csn (oa_ent (u_isn u_hdip)))(u_isn oa_wa)))((u_csn cp_ent)(p_csn ca_ent)))(ca_wa ca_wa)))	22	12
6939	(((p_csn (oa_ent (u_isn u_hdip)))(u_isn oa_wa)))((u_csn cp_ent)(p_csn ca_ent)))(ca_wa p_re)))	22	12
6940	((((cp_ent ca_ent)((ca_wa p_re) cp_ent))(oa_ent (u_isn u_hdip))))(oa_wa u_hdip)(u_isn oa_wa)))	27	12
6941	((((cp_ent ca_ent)((ca_wa p_re) cp_ent))((oa_ent u_isn) u_hdip)))(oa_wa u_hdip)(u_isn oa_wa)))	29	12
6942	((((cp_ent ca_ent)(ca_wa p_re))(ca_wa (p_csn cp_ent)))(oa_ent (u_isn u_hdip)))(u_isn u_hdip))	28	12
6943	((((cp_ent ca_ent)((p_re ca_wa)(cp_ent oa_ent))))(oa_wa u_hdip)(u_isn oa_wa)))(u_csn cp_ent))	24	12
6944	((((cp_ent ca_ent)(ca_wa p_re))(ca_wa (p_csn cp_ent))))(oa_ent u_isn) u_hdip))(u_isn u_hdip))	30	12
6945	((((oa_wa oa_wa)((u_csn cp_ent) p_csn))(p_isn ca_ent)))((ca_wa p_re) cp_ent)(oa_ent u_hdip)))	14	12
6946	((((oa_wa (u_csn cp_ent))((ca_ent ca_wa) p_re)))(cp_ent oa_ent)(u_isn u_hdip)))(u_isn u_hdip))	27	12
6947	((((u_csn (cp_ent ca_ent)))((ca_wa p_re) ca_wa))(cp_ent oa_ent)))(oa_wa u_hdip)(u_isn oa_wa)))	25	12
6948	((((u_csn (cp_ent ca_ent)))((ca_wa p_re) ca_wa)))(cp_ent oa_ent)(u_isn u_hdip)))(u_isn oa_wa))	26	12
6949	((((u_csn cp_ent)(ca_ent ca_wa)))((p_re ca_wa)(cp_ent oa_ent)))(oa_wa u_hdip))(u_isn u_hdip))	21	12
6950	((((u_csn cp_ent)(ca_ent ca_wa)))((p_re ca_wa)(cp_ent oa_ent)))(u_isn u_hdip))(u_isn u_hdip))	29	12
6951	((((u_csn cp_ent)((ca_ent p_isn)(ca_wa p_csn))))(cp_ent oa_ent)(u_isn u_hdip)))(u_isn u_hdip))	24	12
6952	(((u_csn cp_ent)((ca_ent ca_wa) p_re))(p_isn ca_wa)))((p_csn (oa_ent u_isn))(u_hdip u_hdip)))	22	12
6953	(((((u_csn cp_ent)(p_isn ca_ent))(ca_wa p_re))(p_isn ca_wa))(cp_ent oa_ent)))(u_isn u_hdip))	23	12
6954	((((u_hdip oa_wa)((u_csn cp_ent) p_csn))(p_isn ca_wa)))((p_re ca_wa)((cp_ent oa_ent) u_hdip)))	15	12
6955	((((u_hdip u_hdip)(oa_wa (u_csn cp_ent)))((ca_ent ca_wa)))((p_re ca_wa)((cp_ent oa_ent) oa_wa)))	19	12
6956	(((((u_hdip u_hdip)(oa_wa (u_csn cp_ent))) ca_ent)((ca_wa p_re) ca_wa)))((cp_ent oa_ent) u_hdip))	24	12
6957	((((u_hdip u_hdip)(oa_wa (u_csn cp_ent))) ca_ent)((ca_wa p_re) cp_ent)((oa_ent u_isn) u_hdip)))	24	12
6958	((((u_hdip u_hdip)(oa_wa (u_csn cp_ent)))((ca_ent ca_wa)))((p_re ca_wa) oa_ent)(u_hdip u_hdip)))	18	12
6959	((((u_hdip u_hdip)(oa_wa (u_csn cp_ent))) ca_ent)(((ca_wa p_re) ca_wa) oa_ent)(u_hdip u_hdip)))	20	12
6960	((((u_hdip u_hdip)(oa_wa (u_csn cp_ent))) ca_ent)(((ca_wa p_re) cp_ent) oa_ent)(u_isn oa_wa)))	19	12
6961	((((u_hdip u_hdip)(oa_wa (u_csn cp_ent))) ca_ent)(((ca_wa p_re) cp_ent) oa_ent)(u_isn u_hdip)))	24	12
6962	((((u_hdip u_hdip)(oa_wa (u_csn cp_ent)))((p_csn (ca_ent ca_wa)))((p_re ca_wa)(cp_ent oa_ent)))	19	12
6963	((((u_hdip u_hdip)(oa_wa (u_csn cp_ent)))((p_csn ca_ent)))((ca_wa p_re) ca_wa)(oa_ent u_isn)))	17	12
6964	((((u_hdip u_hdip)(oa_wa (u_csn cp_ent)))((p_csn ca_ent)))((ca_wa p_re) cp_ent)(oa_ent u_isn)))	19	12
6965	((((u_isn cp_ent) ca_ent)((ca_wa p_re) ca_wa)))(cp_ent oa_ent)(u_isn u_hdip)))(u_isn u_hdip))	24	12
6966	(((u_isn oa_wa)((u_csn cp_ent) p_csn))(p_isn ca_ent)))((ca_wa p_re) ca_wa)(cp_ent oa_ent)))	16	12
6967	(((u_isn oa_wa)((u_csn cp_ent) p_csn))(p_isn ca_ent)))((ca_wa p_re) ca_wa)(oa_ent u_isn)))	15	12
6968	(((u_isn oa_wa)((u_csn cp_ent) p_csn))(p_isn ca_ent)))((ca_wa p_re) cp_ent)(oa_ent u_isn)))	16	12
6969	(((u_isn oa_wa)((u_csn cp_ent) p_csn))(p_isn ca_wa)))((p_re ca_wa)((cp_ent oa_ent) u_hdip)))	16	12
6970	(((u_isn oa_wa)((u_csn cp_ent) p_csn))(p_isn ca_wa)))((p_re ca_wa) oa_ent)(u_hdip u_hdip)))	16	12
6971	((((u_isn u_hdip)((u_isn oa_wa) u_csn))(cp_ent ca_ent)))((ca_wa p_re) ca_wa)(cp_ent oa_ent)))	22	12
6972	((((u_isn u_hdip)((u_isn oa_wa) u_csn))(cp_ent ca_ent)))((ca_wa p_re) cp_ent)(oa_ent u_isn)))	21	12
6973	((((u_isn u_hdip)((u_isn oa_wa) u_csn))(cp_ent ca_ent)))((ca_wa p_re) cp_ent)(oa_ent u_hdip)))	21	12
6974	((((oa_wa (u_csn cp_ent)) p_csn) ca_ent)))((ca_wa p_re) ca_wa)(cp_ent oa_ent)))(u_hdip u_hdip)))	23	12
6975	((((oa_wa (u_csn cp_ent)) p_csn)(p_isn ca_ent)))((ca_wa p_re) ca_wa)((cp_ent oa_ent) u_hdip)))	19	12
6976	((((oa_wa (u_csn cp_ent)) p_csn)(p_isn ca_ent)))((ca_wa p_re) cp_ent)((oa_ent u_isn) u_hdip)))	21	12
6977	((((oa_wa (u_csn cp_ent)) p_csn)(p_isn ca_ent)))((ca_wa p_re) cp_ent) oa_ent)(u_isn u_hdip)))	21	12
6978	((((oa_wa (u_csn cp_ent))(p_isn ca_ent)))((p_re ca_wa)((cp_ent oa_ent) oa_wa)))(u_hdip u_hdip))	17	12
6979	((((oa_wa (u_csn cp_ent))(p_isn ca_wa)))((p_re ca_wa)(cp_ent oa_ent))))((oa_wa u_hdip) oa_wa))	21	12
6980	((((oa_wa (u_csn cp_ent)))((ca_wa p_re) ca_wa)))(cp_ent oa_ent)(u_isn u_hdip)))(u_isn u_hdip))	26	12
6981	((((oa_wa (u_csn cp_ent)))((ca_wa p_re) ca_wa)))(p_csn oa_ent)(u_isn u_hdip)))(u_isn u_hdip))	22	12
6982	(((p_csn (ca_ent ca_wa)))((p_re ca_wa) p_csn)))(p_isn oa_ent)(u_isn u_hdip)))(u_isn u_hdip))	21	12
6983	(((p_csn (oa_ent u_hdip))(u_isn oa_wa))((u_csn cp_ent)))((p_csn (ca_ent ca_wa))(p_isn p_re)))	23	12
6984	((((u_csn cp_ent)(ca_ent ca_wa))(p_re p_re)))((p_csn (p_isn oa_ent)) u_hdip))(u_isn u_hdip))	19	12
6985	((((u_csn cp_ent)(ca_wa p_re))(ca_wa p_csn)))(cp_ent oa_ent)(u_isn u_hdip)))(u_isn u_hdip))	23	12
6986	((((u_csn (cp_ent ca_wa))(p_isn ca_wa)))(cp_ent oa_ent)(u_isn u_hdip))))(u_isn oa_wa) u_hdip))	22	12
6987	((((u_csn cp_ent)(p_isn ca_ent)))((ca_wa p_re) ca_wa))(cp_ent oa_ent)))(u_isn oa_wa) u_hdip))	23	12
6988	(((u_csn (u_isn cp_ent))(p_isn ca_ent)))((ca_wa p_re) ca_wa)))((cp_ent oa_ent)(u_isn u_hdip)))	22	12
6989	((((u_hdip (u_isn oa_wa)) u_csn)(cp_ent ca_ent)))((ca_wa p_re) ca_wa)))((cp_ent oa_ent) u_hdip))	26	12
6990	((((u_hdip (u_isn oa_wa)) u_csn)(ca_ent ca_wa)))((p_re ca_wa)((cp_ent oa_ent)(oa_wa u_hdip))))	21	12
6991	((((u_hdip (u_isn oa_wa)) u_csn)(ca_ent ca_wa)))((p_re ca_wa)((p_csn oa_ent)(u_isn u_hdip))))	19	12
6992	((((u_hdip (u_isn oa_wa)) u_csn)(cp_ent ca_ent)))((ca_wa p_re) ca_wa)(p_csn (oa_ent u_isn))))	20	12
6993	((((u_hdip (u_isn oa_wa)) u_csn)(ca_ent ca_wa)))((p_re ca_wa)(cp_ent oa_ent)))(oa_wa u_hdip)))	20	12
6994	((((u_hdip (u_isn oa_wa)) u_csn)((cp_ent ca_ent)((ca_wa p_re) cp_ent)))((oa_ent u_isn) u_hdip))	24	12
6995	((((u_hdip (u_isn oa_wa)) u_csn)(cp_ent ca_ent)))((ca_wa p_re) cp_ent)((oa_ent u_isn) u_hdip))	25	12
6996	((((u_hdip (u_isn oa_wa)) u_csn)(ca_ent ca_wa)))((p_re ca_wa) p_csn) oa_ent)(u_isn u_hdip)))	21	12
6997	((((u_hdip (u_isn oa_wa)) u_csn)(ca_ent ca_wa)))((p_re ca_wa)(cp_ent oa_ent)))(u_hdip u_hdip)))	24	12
6998	((((u_hdip (u_isn oa_wa)) u_csn)(ca_ent ca_wa))(p_isn p_re)))(cp_ent oa_ent)(u_isn u_hdip)))	25	12
6999	((((u_hdip (u_isn oa_wa)) u_csn)(cp_ent ca_ent)))((ca_wa p_re) ca_wa) oa_ent)(u_hdip u_hdip)))	21	12
7000	((((u_hdip (u_isn oa_wa)) u_csn)(cp_ent ca_ent)))((ca_wa p_re) ca_wa) oa_ent)(u_isn u_hdip)))	21	12
7001	((((u_hdip (u_isn oa_wa)) u_csn)(cp_ent ca_ent)))((ca_wa p_re) cp_ent) oa_ent)(u_isn oa_wa)))	20	12
7002	((((u_hdip (u_isn oa_wa)) u_csn)(cp_ent ca_ent)))((ca_wa p_re) cp_ent) oa_ent)(u_isn u_hdip)))	25	12
7003	((((u_hdip (u_isn oa_wa)) u_csn)(ca_ent ca_wa)))((p_re ca_wa) p_csn) oa_ent)(u_hdip u_hdip)))	20	12
7004	((((u_hdip (u_isn oa_wa)) u_csn)(ca_ent ca_wa)))((p_re ca_wa)(p_csn ((p_isn oa_ent) u_hdip))))	15	12
7005	((((u_hdip (u_isn oa_wa)) u_csn)(cp_ent ca_wa)))((p_re ca_wa)(cp_ent oa_ent)))(oa_wa u_hdip)))	20	12
7006	((((u_hdip (u_isn oa_wa)) u_csn)(cp_ent ca_wa)))((p_re ca_wa)(cp_ent oa_ent)))(oa_wa u_hdip)))	19	12
7007	((((u_hdip (u_isn oa_wa)) u_csn)(cp_ent ca_wa)))((p_re ca_wa)(cp_ent oa_ent)))(u_hdip u_hdip)))	20	12
7008	((((u_hdip (u_isn oa_wa)) u_csn)(cp_ent ca_wa)))((p_re ca_wa)(cp_ent oa_ent)))(u_isn u_hdip)))	20	12
7009	(((ca_ent p_isn)))((p_re ca_wa)(cp_ent oa_ent)))(u_isn u_hdip))(u_isn u_hdip)))(u_isn u_hdip))	34	12
7010	((((oa_wa u_csn) p_csn) ca_ent)))((ca_wa p_re) cp_ent)((oa_ent u_isn) u_hdip)))(u_isn u_hdip))	22	12
7011	((((oa_wa u_csn)(cp_ent ca_wa))(p_re ca_wa)))(cp_ent oa_ent)(oa_wa u_hdip)))(u_isn u_hdip))	23	12
7012	((((u_csn cp_ent)(ca_wa p_re))(p_isn ca_wa)))(cp_ent oa_ent)(u_isn u_hdip)))(u_isn u_hdip))	23	12
7013	((((u_csn cp_ent)(p_isn ca_ent)))((ca_wa p_re) ca_wa)))(cp_ent oa_ent) u_hdip))(u_isn u_hdip))	29	12
7014	((((u_csn cp_ent)(p_isn ca_ent)))((p_re ca_wa) p_csn))(oa_ent (u_isn u_hdip)))(u_isn u_hdip))	25	12
7015	((((u_csn cp_ent)(p_isn ca_wa)))(p_isn ca_wa)))(cp_ent oa_ent)(u_isn u_hdip)))(u_isn oa_wa))	23	12
7016	((((u_csn cp_ent)(p_isn ca_wa)))(p_isn ca_wa)))(cp_ent oa_ent)(u_isn u_hdip)))(u_isn u_hdip))	23	12
7017	((((u_csn cp_ent)(p_isn ca_wa))(p_isn p_re)))(cp_ent oa_ent)(u_isn u_hdip)))(u_isn oa_wa))	26	12
7018	((((u_csn cp_ent)(p_isn ca_wa))(p_isn p_re)))(cp_ent oa_ent)(u_isn u_hdip)))(u_isn u_hdip))	25	12
7019	((((u_csn cp_ent)(p_isn ca_wa)))(p_re ca_wa)))(cp_ent oa_ent)))(oa_wa u_hdip)(u_isn oa_wa)))	22	12
7020	((((u_csn cp_ent)(p_isn p_re))(ca_wa p_csn)))(cp_ent oa_ent)(u_isn u_hdip)))(u_isn u_hdip))	21	12
7021	((((u_hdip u_csn)(cp_ent ca_ent)))((ca_wa p_re) ca_wa)))(cp_ent oa_ent) u_hdip))(u_isn u_hdip))	26	12
7022	((((u_isn cp_ent)(ca_wa p_re))(ca_wa p_csn)))(cp_ent oa_ent)(u_isn u_hdip)))(u_isn u_hdip))	20	12
7023	((((u_isn cp_ent)(p_isn ca_wa))(p_re ca_wa)))(cp_ent oa_ent)(u_isn u_hdip)))(u_isn oa_wa))	18	12
7024	((((u_isn cp_ent)(p_isn ca_wa))(p_re ca_wa)))(cp_ent oa_ent)(u_isn u_hdip)))(u_isn u_hdip))	19	12
7025	((((u_isn oa_wa) u_hdip)(oa_wa u_csn))(cp_ent ca_ent)))((ca_wa p_re) ca_wa)(cp_ent oa_ent)))	21	12
7026	((((u_isn oa_wa) u_hdip)(oa_wa u_csn))(cp_ent ca_ent)))((ca_wa p_re) cp_ent)(oa_ent u_isn)))	20	12
7027	(p_csn ((p_isn ca_ent)))((ca_wa p_re) ca_wa))((cp_ent oa_ent)(u_isn u_hdip)))(u_isn u_hdip))))	20	12
7028	((ca_ent ca_wa)))(p_isn p_re)))(ca_wa p_csn)(oa_ent u_hdip))(u_isn u_hdip)))(u_isn u_hdip)))	27	12
7029	((ca_ent ca_wa)))(p_isn p_re)))(ca_wa p_csn)(oa_ent u_isn))(u_hdip u_hdip)))(u_isn u_hdip)))	26	12
7030	((ca_ent ca_wa)))(p_isn p_re)))(ca_wa p_csn)(cp_ent oa_ent))(u_isn u_hdip)))(u_isn oa_wa)))	26	12
7031	((ca_ent ca_wa)))((p_re ca_wa)((cp_ent oa_ent)(oa_wa u_hdip)))(u_isn u_hdip))(u_isn u_hdip)))	29	12
7032	((ca_ent ca_wa)))((p_isn ca_wa) p_csn) oa_ent)(u_hdip u_hdip))(u_isn u_hdip))(u_isn u_hdip)))	20	12
7033	((ca_ent ca_wa)))((p_re ca_wa)(cp_ent oa_ent))(oa_wa u_hdip))(u_isn u_hdip))(u_isn u_hdip)))	30	12
7034	((ca_ent ca_wa)))((p_re ca_wa)(cp_ent oa_ent))(u_hdip u_hdip))(u_isn u_hdip))(oa_wa u_csn)))	32	12
7035	((ca_ent ca_wa)))((p_re ca_wa)(cp_ent oa_ent))(u_hdip u_hdip))(u_isn u_hdip))(u_isn oa_wa)))	32	12
7036	((ca_ent ca_wa)))((p_re ca_wa)(cp_ent oa_ent))(u_hdip u_hdip))(u_isn u_hdip))(u_isn u_hdip)))	32	12
7037	((ca_ent ca_wa)))((p_re ca_wa)(cp_ent oa_ent))(u_isn u_hdip))(u_isn u_hdip))(u_isn oa_wa)))	32	12
7038	((ca_ent ca_wa)))((p_re ca_wa)(p_csn oa_ent))(u_hdip u_hdip))(u_isn u_hdip))(u_isn u_hdip)))	23	12
7039	((ca_ent ca_wa)))((p_re ca_wa)(p_csn oa_ent))(u_isn u_hdip))(u_isn oa_wa))(u_isn u_hdip)))	27	12
7040	((ca_ent p_isn)))((p_re (ca_wa p_csn)) oa_ent)(u_hdip u_hdip))(u_isn u_hdip))(u_isn u_hdip)))	18	12
7041	((ca_ent p_isn)))((p_re ca_wa)(cp_ent oa_ent))(u_hdip u_hdip))(u_isn u_hdip))(oa_wa u_csn)))	28	12
7042	((ca_ent p_isn)))((p_re ca_wa)(cp_ent oa_ent))(u_hdip u_hdip))(u_isn u_hdip))(u_isn oa_wa)))	28	12
7043	((ca_ent p_isn)))((p_re ca_wa)(cp_ent oa_ent))(u_hdip u_hdip))(u_isn u_hdip))(u_isn u_hdip)))	28	12
7044	((ca_ent p_isn)))((p_re ca_wa)(cp_ent oa_ent))(u_isn u_hdip))(u_isn u_hdip))(u_isn oa_wa)))	31	12
7045	((ca_ent p_isn)))((p_re ca_wa)(p_csn oa_ent))(u_hdip u_hdip))(u_isn u_hdip))(u_isn u_hdip)))	18	12
7046	((cp_ent ca_ent)))((ca_wa p_re) ca_wa))(p_csn ((p_isn oa_ent)(u_isn u_hdip)))(u_isn u_hdip))))	21	12
7047	((cp_ent ca_ent)))(p_isn p_re)))(ca_wa p_csn)(oa_ent u_hdip))(u_isn u_hdip)))(u_isn u_hdip)))	25	12
7048	((cp_ent ca_ent)))(p_isn p_re)))(ca_wa p_csn)(oa_ent u_isn))(u_hdip u_hdip)))(u_isn u_hdip)))	24	12
7049	((cp_ent ca_ent)))(p_isn p_re)))(ca_wa p_csn)(cp_ent oa_ent))(u_isn u_hdip)))(u_isn oa_wa)))	24	12
7050	((cp_ent ca_ent)))(p_isn p_re)))(ca_wa (p_csn cp_ent)))(oa_ent (u_isn u_hdip))))(u_isn oa_wa)))	19	12
7051	((cp_ent ca_ent)))((ca_wa p_re) ca_wa)(p_csn (p_isn oa_ent)))(u_isn u_hdip)))(u_isn u_hdip)))	23	12
7052	((cp_ent ca_ent)))((p_isn p_re)(ca_wa (p_csn cp_ent))))(oa_ent u_isn) u_hdip))(u_isn oa_wa)))	23	12
7053	((cp_ent ca_ent)))((ca_wa p_re) ca_wa)(p_csn (oa_ent u_isn)))(u_hdip u_hdip))(u_isn u_hdip)))	26	12
7054	(((cp_ent ca_ent)))((ca_wa p_re) ca_wa))((cp_ent oa_ent) oa_wa))(u_hdip u_hdip)))(u_isn u_hdip))	24	12
7055	((cp_ent ca_ent)))((ca_wa p_re) ca_wa))((cp_ent oa_ent) u_hdip))(u_isn u_hdip))(u_isn u_hdip)))	34	12
7056	((cp_ent ca_ent)))((ca_wa p_re) ca_wa))(p_csn oa_ent) u_hdip))(u_isn u_hdip))(u_isn u_hdip)))	27	12
7057	((cp_ent ca_ent)))((ca_wa ca_wa)(cp_ent oa_ent))(u_hdip u_hdip))(u_isn u_hdip))(oa_wa u_csn)))	22	12
7058	((cp_ent ca_ent)))((ca_wa ca_wa)(cp_ent oa_ent))(u_hdip u_hdip))(u_isn u_hdip))(u_isn oa_wa)))	23	12
7059	((cp_ent ca_ent)))((ca_wa ca_wa)(cp_ent oa_ent))(u_isn u_hdip))(u_isn u_hdip))(u_isn oa_wa)))	22	12
7060	((cp_ent ca_ent)))((p_isn ca_wa) p_csn) oa_ent)(u_hdip u_hdip))(u_isn u_hdip))(u_isn u_hdip)))	18	12
7061	((cp_ent ca_ent)))((ca_wa p_re) ca_wa)(cp_ent oa_ent))(u_isn u_hdip)))(u_isn oa_wa) u_hdip))	29	12
7062	((cp_ent ca_ent)))((ca_wa p_re) ca_wa))((cp_ent oa_ent) oa_wa))(u_hdip u_hdip))(u_isn u_hdip)))	25	12
7063	((cp_ent ca_ent)))((ca_wa p_re) ca_wa))((cp_ent oa_ent) oa_wa))(u_isn u_hdip))(u_isn u_hdip)))	27	12
7064	((cp_ent ca_ent)))((p_re ca_wa)(cp_ent oa_ent))(oa_wa u_hdip))(u_isn u_hdip))(u_isn u_hdip)))	26	12
7065	((cp_ent ca_ent)))((p_re ca_wa)(cp_ent oa_ent))(u_isn u_hdip))(u_isn u_hdip))(u_isn oa_wa)))	27	12
7066	((cp_ent ca_wa)))((p_re ca_wa)(cp_ent oa_ent))(u_hdip u_hdip))(u_isn u_hdip))(u_isn oa_wa)))	29	12
7067	((p_csn ca_ent)))((ca_wa p_re) ca_wa))(cp_ent oa_ent) u_hdip))(u_isn u_hdip))(u_isn oa_wa)))	21	12
7068	((p_isn ca_ent)))((ca_wa p_re) ca_wa))(p_csn ((p_isn oa_ent)(u_isn u_hdip)))(u_isn u_hdip))))	22	12
7069	((p_isn ca_ent)))((ca_wa p_re) ca_wa)))((p_csn cp_ent) oa_ent)(u_isn u_hdip)))(u_isn oa_wa)))	17	12
7070	((p_isn ca_ent)))(p_isn p_re)))(ca_wa p_csn)(oa_ent u_isn))(u_hdip u_hdip)))(u_isn u_hdip)))	21	12
7071	((p_isn ca_ent)))(p_isn p_re)))(ca_wa p_csn)(cp_ent oa_ent))(u_isn u_hdip)))(u_isn oa_wa)))	20	12
7072	((p_isn ca_ent)))((ca_wa p_re) ca_wa)((p_csn oa_ent) u_hdip))(u_isn u_hdip))(u_isn u_hdip)))	25	12
7073	((p_isn ca_ent)))((ca_wa p_re) ca_wa)(p_csn oa_ent))(u_isn u_hdip)))(u_isn oa_wa) u_hdip)))	24	12
7074	((p_isn ca_ent)))((ca_wa p_re) ca_wa)(p_csn (p_isn oa_ent)))(u_isn u_hdip))(u_isn u_hdip)))	24	12
7075	((p_isn ca_ent)))((ca_wa p_re)(ca_wa (p_csn cp_ent))))(oa_ent u_isn) u_hdip))(u_isn oa_wa)))	18	12
7076	((p_isn ca_ent)))(p_isn p_re)(ca_wa (p_csn cp_ent)))(oa_ent u_isn) u_hdip))(u_isn oa_wa)))	19	12
7077	((p_isn ca_ent)))((ca_wa p_re) ca_wa)(p_csn (oa_ent u_isn)))(u_hdip u_hdip))(u_isn u_hdip)))	25	12
7078	(((p_isn ca_ent)))((ca_wa p_re) ca_wa))(cp_ent oa_ent) oa_wa))(u_isn u_hdip)))(u_isn u_hdip)))	27	12
7079	(((p_isn ca_ent)))((ca_wa p_re) ca_wa)(cp_ent oa_ent))(u_isn u_hdip)))(u_isn oa_wa) u_hdip))	28	12
7080	((p_isn ca_ent)))((ca_wa p_re) ca_wa))((cp_ent oa_ent) oa_wa))(u_hdip u_hdip))(u_isn u_hdip)))	20	12
7081	((p_isn ca_ent)))((ca_wa p_re) ca_wa))(cp_ent oa_ent) oa_wa))(u_isn u_hdip))(u_isn u_hdip)))	25	12
7082	((p_isn ca_ent)))((ca_wa p_re) cp_ent))((oa_ent u_isn) u_hdip))(u_isn oa_wa))(u_isn oa_wa)))	27	12
7083	((p_isn ca_ent)))((ca_wa p_re) cp_ent))((oa_ent u_isn) u_hdip))(u_isn oa_wa))(u_isn u_hdip)))	28	12
7084	((p_isn ca_ent)))((ca_wa p_re) cp_ent))((oa_ent u_isn) u_hdip))(u_isn u_hdip))(u_isn u_hdip)))	28	12
7085	(((p_isn ca_ent)))((p_re ca_wa) p_csn) oa_ent)(u_isn u_hdip))(u_isn u_hdip)))(u_isn u_hdip))	26	12
7086	((p_isn ca_ent)))((p_re ca_wa)(cp_ent oa_ent))(oa_wa u_hdip))(u_isn u_hdip)))(u_isn u_hdip)))	24	12
7087	((p_isn ca_ent)))((p_re ca_wa)(cp_ent oa_ent))(u_isn u_hdip))(u_isn u_hdip))(u_isn oa_wa)))	23	12
7088	((p_isn ca_ent)))((p_re ca_wa)(p_csn oa_ent))(u_isn u_hdip))(u_isn oa_wa))(u_isn u_hdip)))	21	12
7089	(((p_isn ca_wa))(p_isn p_re)))(ca_wa p_csn)(oa_ent u_hdip))(u_isn u_hdip)))(u_isn u_hdip))	26	12
7090	((p_isn ca_wa)))((p_re ca_wa)((cp_ent oa_ent)(oa_wa u_hdip)))(u_isn u_hdip))(u_isn u_hdip)))	28	12
7091	((p_isn ca_wa)))((p_re ca_wa)(cp_ent oa_ent))(oa_wa u_hdip))(u_isn u_hdip))(u_isn u_hdip)))	29	12
7092	((p_isn ca_wa)))((p_re ca_wa)(cp_ent oa_ent))(u_hdip u_hdip))(u_isn u_hdip))(oa_wa u_csn)))	30	12
7093	((p_isn ca_wa)))((p_re ca_wa)(cp_ent oa_ent))(u_hdip u_hdip))(u_isn u_hdip))(u_isn oa_wa)))	30	12
7094	((p_isn ca_wa)))((p_re ca_wa)(cp_ent oa_ent))(u_hdip u_hdip))(u_isn u_hdip))(u_isn u_hdip)))	30	12
7095	((p_isn ca_wa)))((p_re ca_wa)(cp_ent oa_ent))(u_isn u_hdip))(u_isn u_hdip))(u_isn oa_wa)))	30	12
7096	((u_csn (cp_ent ca_ent)))((ca_wa p_re)(p_csn ((p_isn oa_ent)(u_isn u_hdip))))(u_isn u_hdip)))	18	12
7097	(((u_csn (cp_ent ca_ent)))((ca_wa p_re) ca_wa)(p_csn ((p_isn oa_ent) u_hdip))))(u_isn u_hdip))	19	12
7098	((u_csn (cp_ent ca_ent)))((ca_wa p_re) ca_wa)(cp_ent oa_ent))(u_isn oa_wa))(u_isn oa_wa)))	26	12
7099	((u_csn (cp_ent ca_ent)))((ca_wa p_re) ca_wa)(cp_ent oa_ent))(u_isn oa_wa))(u_isn u_hdip)))	24	12
7100	((u_csn (cp_ent ca_ent)))((ca_wa p_re) ca_wa)(cp_ent oa_ent))(u_isn u_hdip))(u_isn oa_wa)))	28	12
7101	((u_csn (cp_ent ca_ent)))((ca_wa p_re) ca_wa)(oa_ent u_isn))(u_hdip u_hdip))(u_isn u_hdip)))	22	12
7102	((u_csn (cp_ent ca_ent)))((ca_wa p_re) cp_ent)(oa_ent u_hdip))(u_isn u_hdip))(u_isn u_hdip)))	28	12
7103	(((u_csn (cp_ent ca_ent)))((ca_wa p_re) ca_wa)(cp_ent oa_ent))(u_isn u_hdip))(u_isn u_hdip))	31	12
7104	((u_csn (cp_ent ca_ent)))((ca_wa p_re) cp_ent)(oa_ent u_hdip))(u_isn oa_wa))(u_isn oa_wa)))	21	12
7105	((u_csn (cp_ent ca_ent)))((ca_wa p_re) cp_ent)(oa_ent u_hdip))(u_isn oa_wa))(u_isn u_hdip)))	22	12
7106	((u_csn (cp_ent ca_ent)))((ca_wa p_re) cp_ent)(oa_ent u_isn))(u_hdip u_hdip))(u_isn u_hdip)))	24	12
7107	(((u_csn cp_ent)(ca_ent ca_wa)))((p_re ca_wa) p_csn) oa_ent)(u_hdip u_hdip))(u_isn u_hdip)))	23	12
7108	(((u_csn cp_ent)(ca_ent ca_wa)))((p_re ca_wa) p_csn) oa_ent)(u_isn u_hdip))(u_isn u_hdip))	26	12
7109	((u_hdip u_hdip)((u_isn oa_wa))((u_csn cp_ent)(p_csn ca_ent)))((ca_wa p_re) ca_wa) oa_ent))))	17	12
7110	((u_hdip u_hdip)((u_isn oa_wa))((u_csn cp_ent) p_csn))(ca_ent ca_wa)))((p_re ca_wa) oa_ent)))	17	12
7111	((u_hdip u_hdip)((u_isn oa_wa))((u_csn cp_ent) p_csn)) ca_ent)((ca_wa p_re) ca_wa) oa_ent)))	18	12
7112	((u_isn u_hdip)((u_isn oa_wa))((u_csn cp_ent) p_csn)) ca_ent)((ca_wa p_re) ca_wa) oa_ent)))	17	12
7113	((oa_wa (u_csn (cp_ent ca_ent)))((ca_wa p_re) cp_ent) oa_ent)(u_isn oa_wa))(u_isn oa_wa)))	22	12
7114	((oa_wa (u_csn (cp_ent ca_ent)))((ca_wa p_re) cp_ent) oa_ent)(u_isn u_hdip))(oa_wa u_hdip)))	20	12
7115	((oa_wa (u_csn (cp_ent ca_ent)))((ca_wa p_re) cp_ent) oa_ent)(u_isn u_hdip))(u_isn oa_wa)))	25	12
7116	((oa_wa (u_csn (cp_ent ca_ent)))((ca_wa p_re) cp_ent) oa_ent)(u_isn u_hdip))(u_isn u_hdip)))	28	12
7117	((oa_wa (u_csn (cp_ent ca_ent)))((p_re ca_wa) p_csn) oa_ent)(u_isn u_hdip))(u_isn u_hdip)))	23	12
7118	((p_csn (p_isn ca_ent)))((ca_wa p_re) ca_wa)(cp_ent oa_ent))(u_isn u_hdip))(u_isn u_hdip)))	21	12
7119	((u_csn (cp_ent ca_wa)))((p_re ca_wa))((cp_ent oa_ent) oa_wa))(u_hdip u_hdip))(u_isn u_hdip)))	18	12
7120	((u_csn (cp_ent ca_wa)))((p_re ca_wa))((cp_ent oa_ent) u_hdip))(u_isn u_hdip))(u_isn oa_wa)))	20	12
7121	((u_csn (cp_ent ca_wa)))((p_re ca_wa))((cp_ent oa_ent) u_hdip))(u_isn u_hdip))(u_isn u_hdip)))	23	12
7122	((((u_csn cp_ent) p_csn) ca_ent)))((ca_wa p_re) ca_wa) oa_ent)(u_hdip u_hdip))(u_isn u_hdip)))	19	12
7123	((((u_csn cp_ent) p_csn) ca_ent)))((ca_wa p_re) cp_ent) oa_ent)(u_isn u_hdip))(u_isn u_hdip)))	25	12
7124	(((u_csn cp_ent)(p_isn ca_ent)))((ca_wa p_re))(p_csn (p_isn oa_ent)) u_hdip))(u_isn u_hdip)))	17	12
7125	(((u_csn (p_isn ca_ent)))((ca_wa p_re) ca_wa)(p_csn ((p_isn oa_ent) u_hdip))))(u_isn u_hdip))	17	12
7126	((u_csn (p_isn ca_ent)))((ca_wa p_re) ca_wa)(cp_ent oa_ent))(u_isn oa_wa))(u_isn oa_wa)))	20	12
7127	((u_csn (p_isn ca_ent)))((ca_wa p_re) ca_wa)(cp_ent oa_ent))(u_isn oa_wa))(u_isn u_hdip)))	20	12
7128	((u_csn (p_isn ca_ent)))((ca_wa p_re) ca_wa)(cp_ent oa_ent))(u_isn u_hdip))(u_isn oa_wa)))	22	12
7129	((u_csn (p_isn ca_ent)))((ca_wa p_re) ca_wa)(oa_ent u_isn))(u_hdip u_hdip))(u_isn u_hdip)))	19	12
7130	((u_csn (p_isn ca_ent)))((ca_wa p_re) cp_ent)(oa_ent u_hdip))(u_isn oa_wa))(u_isn oa_wa)))	18	12
7131	((u_csn (p_isn ca_ent)))((ca_wa p_re) cp_ent)(oa_ent u_hdip))(u_isn oa_wa))(u_isn u_hdip)))	19	12
7132	(((oa_wa (u_csn cp_ent)) ca_ent)))((ca_wa p_re))(p_csn (p_isn oa_ent)) u_hdip))(u_isn u_hdip)))	17	12
7133	(((oa_wa (u_csn cp_ent)) ca_ent)))((ca_wa p_re) ca_wa) oa_ent)(u_hdip u_hdip))(u_isn u_hdip)))	22	12
7134	(((oa_wa (u_csn cp_ent)) ca_ent)))((ca_wa p_re) ca_wa) oa_ent)(u_isn u_hdip))(u_isn oa_wa)))	19	12
7135	(((oa_wa (u_csn cp_ent)) ca_ent)))((ca_wa p_re) ca_wa) oa_ent)(u_isn u_hdip))(u_isn u_hdip)))	23	12
7136	(((oa_wa (u_csn cp_ent)) ca_ent)))((ca_wa p_re) cp_ent) oa_ent)(u_isn u_hdip))(u_isn oa_wa)))	26	12
7137	(((oa_wa (u_csn cp_ent)) ca_ent)))((ca_wa p_re) cp_ent) oa_ent)(u_isn u_hdip))(u_isn u_hdip)))	29	12
7138	((oa_wa u_csn)(p_isn ca_ent)))((ca_wa p_re) cp_ent) oa_ent)(u_isn oa_wa))(u_isn oa_wa)))	17	12
7139	((oa_wa u_csn)(p_isn ca_ent)))((ca_wa p_re) cp_ent) oa_ent)(u_isn u_hdip))(u_isn oa_wa)))	19	12
7140	((oa_wa u_csn)(p_isn ca_ent)))((ca_wa p_re) cp_ent) oa_ent)(u_isn u_hdip))(u_isn u_hdip)))	23	12
7141	(((u_csn cp_ent)(p_isn ca_ent)))((ca_wa p_re) ca_wa) oa_ent)(u_hdip u_hdip))(u_isn u_hdip)))	21	12
7142	(((u_csn cp_ent)(p_isn ca_ent)))((ca_wa p_re) ca_wa) oa_ent)(u_isn u_hdip))(u_isn u_hdip)))	22	12
7143	(((u_csn cp_ent)(p_isn ca_ent)))((ca_wa p_re) cp_ent) oa_ent)(u_isn oa_wa))(u_isn oa_wa)))	20	12
7144	(((u_csn cp_ent)(p_isn ca_ent)))((ca_wa p_re) cp_ent) oa_ent)(u_isn u_hdip))(u_isn oa_wa)))	23	12
7145	(((u_csn cp_ent)(p_isn ca_ent)))((ca_wa p_re) cp_ent) oa_ent)(u_isn u_hdip))(u_isn u_hdip)))	27	12
7146	(((u_hdip u_csn)(cp_ent ca_ent)))((ca_wa p_re) ca_wa) oa_ent)(u_hdip u_hdip))(u_isn u_hdip)))	20	12
7147	(((u_hdip u_csn)(cp_ent ca_ent)))((ca_wa p_re) cp_ent) oa_ent)(u_isn u_hdip))(u_isn oa_wa)))	20	12
7148	(((u_hdip u_csn)(cp_ent ca_ent)))((ca_wa p_re) cp_ent) oa_ent)(u_isn u_hdip))(u_isn u_hdip)))	24	12
7149	(((u_isn cp_ent) ca_ent)))((ca_wa p_re) ca_wa)(cp_ent oa_ent))(u_isn oa_wa))(u_isn oa_wa)))	18	12
7150	(((u_isn cp_ent) ca_ent)))((ca_wa p_re) ca_wa)(cp_ent oa_ent))(u_isn oa_wa))(u_isn u_hdip)))	19	12
7151	(((u_isn cp_ent) ca_ent)))((ca_wa p_re) ca_wa)(cp_ent oa_ent))(u_isn u_hdip))(u_isn u_hdip)))	22	12
7152	(((u_isn cp_ent)(p_isn ca_ent)))((ca_wa p_re) cp_ent) oa_ent)(u_isn oa_wa))(u_isn u_hdip)))	17	12
7153	(((u_isn cp_ent)(p_isn ca_ent)))((ca_wa p_re) cp_ent) oa_ent)(u_isn u_hdip))(u_isn u_hdip)))	20	12
7154	(((u_isn oa_wa) u_hdip)))((u_isn oa_wa))((u_csn cp_ent) p_csn)) ca_ent)((ca_wa p_re) cp_ent)))	17	12
7155	(((u_isn u_csn)(cp_ent ca_ent)))((ca_wa p_re) cp_ent) oa_ent)(u_isn u_hdip))(u_isn oa_wa)))	19	12
7156	(((u_csn (u_isn cp_ent)) ca_ent)))((ca_wa p_re) cp_ent) oa_ent)(u_isn u_hdip))(u_isn oa_wa)))	18	12
7157	(((u_csn (u_isn cp_ent)) ca_ent)))((ca_wa p_re) cp_ent) oa_ent)(u_isn u_hdip))(u_isn u_hdip)))	22	12
7158	((((oa_wa u_csn) p_csn) ca_ent)))((ca_wa p_re) cp_ent) oa_ent)(u_isn u_hdip))(u_isn u_hdip)))	21	12
7159	(((ca_ent ca_wa)))((p_re ca_wa)((cp_ent oa_ent) oa_wa))(u_isn oa_wa)))(u_hdip (u_isn u_csn)))	20	12
7160	(((ca_ent ca_wa)))((p_re ca_wa)(cp_ent oa_ent))(oa_wa u_hdip))(u_isn oa_wa)))(u_csn cp_ent))	24	12
7161	(((ca_ent ca_wa)))((p_re ca_wa)(p_csn oa_ent))(u_hdip u_hdip))(u_isn u_hdip)))(u_isn u_hdip))	25	12
7162	(((ca_ent ca_wa)))((p_re ca_wa)(p_csn oa_ent))(u_isn u_hdip))(u_isn oa_wa)))(u_isn u_hdip))	29	12
7163	(((ca_ent p_isn)))((ca_wa (p_csn cp_ent)))(oa_ent u_isn) u_hdip))(u_isn oa_wa)))(u_isn oa_wa))	19	12
7164	(((ca_ent p_isn)))((p_re ca_wa)((cp_ent oa_ent) oa_wa))(u_isn oa_wa)))(u_hdip (u_isn u_csn)))	19	12
7165	(((ca_ent p_isn)))((p_re ca_wa)(cp_ent oa_ent))(oa_wa u_hdip)))(u_isn u_hdip)))(u_isn oa_wa))	27	12
7166	(((ca_ent p_isn)))((p_re ca_wa)(cp_ent oa_ent))(oa_wa u_hdip)))(u_isn u_hdip)))(u_isn u_hdip))	30	12
7167	(((ca_ent p_isn)))((p_re ca_wa)(cp_ent oa_ent))(oa_wa u_hdip))(u_isn u_hdip)))(u_isn u_hdip))	31	12
7168	(((ca_ent p_isn)))((p_re ca_wa)(cp_ent oa_ent))(u_hdip u_hdip))(u_isn u_hdip)))(oa_wa u_csn))	31	12
7169	(((ca_ent p_isn)))((p_re ca_wa)(cp_ent oa_ent))(u_hdip u_hdip))(u_isn u_hdip)))(u_isn oa_wa))	31	12
7170	(((ca_ent p_isn)))((p_re ca_wa)(cp_ent oa_ent))(u_hdip u_hdip))(u_isn u_hdip)))(u_isn u_hdip))	31	12
7171	(((ca_ent p_isn)))((p_re ca_wa)(p_csn oa_ent))(u_hdip u_hdip))(u_isn u_hdip)))(u_isn u_hdip))	21	12
7172	(((cp_ent ca_ent)))((ca_wa p_re) ca_wa))((cp_ent oa_ent) oa_wa))(u_hdip u_hdip)))(u_isn oa_wa))	25	12
7173	(((cp_ent ca_ent)))((ca_wa p_re) ca_wa))((cp_ent oa_ent) oa_wa))(u_isn u_hdip)))(u_isn u_hdip))	29	12
7174	(((cp_ent ca_ent)))((ca_wa p_re) ca_wa))((cp_ent oa_ent) u_hdip))(u_isn u_hdip)))(u_isn oa_wa))	29	12
7175	(((cp_ent ca_ent)))((ca_wa p_re) ca_wa))(p_csn oa_ent) u_hdip))(u_isn u_hdip)))(u_isn u_hdip))	28	12
7176	(((cp_ent ca_ent)))((ca_wa ca_wa)(cp_ent oa_ent))(u_hdip u_hdip))(u_isn u_hdip)))(u_isn u_hdip))	19	12
7177	(((cp_ent ca_ent)))((ca_wa p_re) ca_wa)(cp_ent oa_ent))(u_isn u_hdip))))(u_isn oa_wa) u_hdip))	32	12
7178	(((cp_ent ca_ent)))((ca_wa p_re) ca_wa)(p_csn oa_ent))(u_isn u_hdip))))(u_isn oa_wa) u_hdip))	26	12
7179	(((cp_ent ca_ent)))((ca_wa p_re) ca_wa))((cp_ent oa_ent) oa_wa))(u_isn u_hdip)))(u_isn u_hdip))	28	12
7180	(((cp_ent ca_ent)))((ca_wa p_re) cp_ent))((oa_ent u_isn) u_hdip))(u_isn u_hdip)))(u_isn u_hdip))	31	12
7181	(((cp_ent ca_ent)))((ca_wa p_re) cp_ent))((oa_ent u_isn) u_hdip))(u_isn oa_wa)))(u_isn oa_wa))	29	12
7182	(((cp_ent ca_ent)))((ca_wa p_re) cp_ent))((oa_ent u_isn) u_hdip))(u_isn oa_wa)))(u_isn u_hdip))	29	12
7183	(((cp_ent ca_ent)))((p_re ca_wa)(cp_ent oa_ent))(oa_wa u_hdip))(u_isn u_hdip)))(u_isn oa_wa))	26	12
7184	(((cp_ent ca_wa)))((p_re ca_wa)(cp_ent oa_ent))(oa_wa u_hdip))(u_isn u_hdip)))(u_isn oa_wa))	26	12
7185	(((cp_ent ca_wa)))((p_re ca_wa)(cp_ent oa_ent))(oa_wa u_hdip))(u_isn u_hdip)))(u_isn u_hdip))	24	12
7186	(((cp_ent ca_wa)))((p_re ca_wa)(cp_ent oa_ent))(u_hdip u_hdip))(u_isn oa_wa)))(u_csn cp_ent))	24	12
7187	(((cp_ent ca_wa)))((p_re ca_wa)(cp_ent oa_ent))(u_hdip u_hdip))(u_isn u_hdip)))(u_isn u_hdip))	24	12
7188	(((cp_ent ca_wa)))((p_re ca_wa)(cp_ent oa_ent))(u_isn u_hdip))(u_isn oa_wa)))(u_csn cp_ent))	23	12
7189	((((cp_ent oa_ent) oa_wa))(u_isn u_hdip))(u_isn u_hdip))(u_isn oa_wa) u_csn))))(oa_ent oa_wa))	19	12
7190	(((p_csn ca_ent)))((ca_wa p_re) ca_wa)(cp_ent oa_ent))(u_isn u_hdip))))(u_isn oa_wa) u_hdip))	23	12
7191	(((p_isn ca_ent)))((ca_wa p_re) ca_wa))((cp_ent oa_ent) u_hdip)))(u_isn oa_wa)))(u_csn cp_ent))	24	12
7192	(((p_isn ca_ent)))((ca_wa p_re) ca_wa))((cp_ent oa_ent) oa_wa))(u_hdip u_hdip)))(u_isn oa_wa))	23	12
7193	(((p_isn ca_ent)))((ca_wa p_re) ca_wa))((p_csn oa_ent) oa_wa))(u_isn u_hdip)))(u_isn u_hdip))	23	12
7194	(((p_isn ca_ent)))((ca_wa p_re) cp_ent))((oa_ent u_isn) u_hdip)))(u_isn u_hdip)))(u_isn u_hdip))	30	12
7195	(((p_isn ca_ent)))((ca_wa p_re) cp_ent))((oa_ent oa_wa) u_hdip))(u_isn u_hdip)))(u_isn u_hdip))	18	12
7196	(((p_isn ca_ent)))((ca_wa p_re) cp_ent)(oa_ent u_hdip))(u_isn oa_wa))))(u_isn oa_wa) u_hdip))	22	12
7197	(((p_isn ca_ent)))((ca_wa p_re) cp_ent)(oa_ent (oa_wa u_hdip)))(u_isn u_hdip)))(u_isn oa_wa))	22	12
7198	(((p_isn ca_ent)))((ca_wa p_re) cp_ent)(oa_ent (oa_wa u_hdip)))(u_isn u_hdip)))(u_isn u_hdip))	19	12
7199	(((p_isn ca_ent)))((ca_wa p_re) cp_ent)(oa_ent (u_isn u_hdip)))(u_isn u_hdip)))(u_isn u_hdip))	28	12
7200	(((p_isn ca_ent)))((p_re ca_wa)(cp_ent oa_ent))(oa_wa u_hdip))(u_isn u_hdip)))(u_isn oa_wa))	24	12
7201	(((p_isn ca_ent)))((p_re ca_wa)(p_csn oa_ent))(u_isn u_hdip))(u_isn oa_wa)))(u_isn oa_wa))	21	12
7202	(((p_isn ca_wa)))((p_re ca_wa)((cp_ent oa_ent) oa_wa))(u_isn oa_wa)))(u_hdip (u_isn u_csn)))	19	12
7203	(((p_isn ca_wa)))((p_re ca_wa)(cp_ent oa_ent))(oa_wa u_hdip))(u_isn u_hdip)))(u_isn oa_wa))	26	12
7204	(((p_isn ca_wa)))((p_re ca_wa)(p_csn oa_ent))(u_isn u_hdip))(u_isn u_hdip)))(u_isn u_hdip))	30	12
7205	(((p_isn ca_wa)))((p_re ca_wa) p_csn)(oa_ent (u_isn u_hdip)))(u_isn u_hdip)))(u_isn u_hdip))	30	12
7206	(((p_isn ca_wa)))((p_re ca_wa)(cp_ent oa_ent))(oa_wa u_hdip))(u_isn u_hdip)))(u_isn oa_wa))	27	12
7207	(((p_isn ca_wa)))((p_re ca_wa)(cp_ent oa_ent))(oa_wa u_hdip))(u_isn u_hdip)))(u_isn u_hdip))	30	12
7208	(((p_isn ca_wa)))((p_re ca_wa)(cp_ent oa_ent))(u_isn u_hdip))(u_isn u_hdip)))(u_isn oa_wa))	29	12
7209	(((p_isn ca_wa)))((p_re ca_wa)(cp_ent oa_ent))(u_isn u_hdip))(u_isn u_hdip)))(u_isn u_hdip))	34	12
7210	(((p_isn ca_wa)))((p_re ca_wa)(p_csn oa_ent))(u_hdip u_hdip))(u_isn u_hdip)))(u_isn u_hdip))	23	12
7211	(((u_csn (cp_ent ca_ent)))((ca_wa p_re) ca_wa)(cp_ent oa_ent))(u_isn oa_wa)))(u_isn u_hdip))	25	12
7212	(((u_csn (cp_ent ca_ent)))((ca_wa p_re) cp_ent)(oa_ent u_isn))(u_hdip u_hdip)))(u_isn u_hdip))	25	12
7213	((((u_hdip u_hdip)((u_isn oa_wa))((u_csn cp_ent) p_csn)))(ca_ent ca_wa)))((p_re ca_wa) oa_ent))	18	12
7214	(((u_hdip u_hdip)((u_isn oa_wa))((u_csn cp_ent) p_csn)) ca_ent)))((ca_wa p_re) ca_wa) oa_ent))	19	12
7215	(((u_hdip u_hdip)((oa_wa (u_csn cp_ent)) p_csn) ca_ent)))((ca_wa p_re) ca_wa)(oa_ent u_isn)))	19	12
7216	(((u_hdip u_hdip)((oa_wa (u_csn cp_ent)) p_csn) ca_ent)))((ca_wa p_re) cp_ent)(oa_ent u_isn)))	20	12
7217	(((u_hdip u_hdip)((oa_wa (u_csn cp_ent)) p_csn)(p_isn ca_ent)))((ca_wa p_re) ca_wa) oa_ent))	15	12
7218	(((u_hdip u_hdip)((oa_wa (u_csn cp_ent)) p_csn)(p_isn ca_ent)))((ca_wa p_re) cp_ent) oa_ent))	16	12
7219	(((oa_ent (u_isn u_hdip))((u_isn u_hdip))(u_isn u_hdip))(u_isn oa_wa) u_csn))))(cp_ent oa_ent))	24	12
7220	(((oa_wa (u_csn (cp_ent ca_ent)))((p_re ca_wa) p_csn) oa_ent)(u_isn u_hdip)))(u_isn u_hdip))	24	12
7221	((((oa_wa (u_csn cp_ent)) ca_ent)))((ca_wa p_re) ca_wa) oa_ent)(u_isn u_hdip)))(u_isn u_hdip))	24	12
7222	((((oa_wa (u_csn cp_ent)) ca_ent)))((ca_wa p_re) cp_ent) oa_ent)(u_isn oa_wa)))(u_isn oa_wa))	23	12
7223	((((oa_wa u_csn) p_csn) ca_ent)))((ca_wa p_re) cp_ent) oa_ent)(u_isn u_hdip)))(u_isn u_hdip))	22	12
7224	(((p_isn p_re)))((ca_wa p_csn)(oa_ent u_isn))(u_hdip u_hdip)))(u_isn u_hdip))(u_isn u_hdip))	19	12
7225	(((p_isn p_re)))((ca_wa p_csn)(oa_ent u_hdip))(u_isn u_hdip)))(u_isn u_hdip))(u_isn u_hdip))	19	12
7226	(((ca_ent p_isn)))((p_re ca_wa)(p_csn oa_ent)(u_isn u_hdip)))(u_isn oa_wa))(u_isn u_hdip))	27	12
7227	(((ca_ent p_isn)))((p_re ca_wa)(p_csn oa_ent))(u_hdip u_hdip))(u_isn u_hdip))(u_isn u_hdip))	24	12
7228	(((ca_ent p_isn)))((p_re ca_wa)(p_csn oa_ent))(u_isn u_hdip)))(u_isn oa_wa))(u_isn u_hdip))	28	12
7229	((((cp_ent ca_ent)))((ca_wa p_re) ca_wa))((cp_ent oa_ent) oa_wa))(u_hdip u_hdip))(u_isn oa_wa)))	28	12
7230	((((cp_ent ca_ent)))((ca_wa p_re) ca_wa))((cp_ent oa_ent) oa_wa)))(u_hdip u_hdip))(u_isn u_hdip)))	26	12
7231	((((cp_ent ca_ent)((p_re ca_wa)((cp_ent oa_ent)(oa_wa u_hdip))))(u_isn u_hdip))(u_isn oa_wa)))	27	12
7232	((((cp_ent ca_ent)))((ca_wa p_re) ca_wa) oa_ent)))(oa_wa u_hdip)(u_isn oa_wa)))(u_csn cp_ent)))	22	12
7233	((((cp_ent ca_ent)))((ca_wa p_re) cp_ent) oa_ent)))(oa_wa u_hdip)(u_isn oa_wa)))(u_csn cp_ent)))	24	12
7234	((((cp_ent ca_ent)))((ca_wa p_re) cp_ent) oa_ent)(u_isn u_hdip)))(u_isn oa_wa)))(u_csn cp_ent))	26	12
7235	((((cp_ent ca_ent)))((ca_wa p_re) cp_ent)(oa_ent (oa_wa u_hdip))))(u_isn u_hdip))(u_isn oa_wa)))	25	12
7236	((((cp_ent ca_ent)))((ca_wa p_re) cp_ent)(oa_ent (oa_wa u_hdip))))(u_isn u_hdip))(u_isn u_hdip)))	25	12
7237	(((cp_ent ca_ent)))((ca_wa p_re) cp_ent)(oa_ent (u_isn u_hdip))))(u_isn oa_wa))(u_isn oa_wa)))	28	12
7238	((((cp_ent ca_ent)))((ca_wa p_re) cp_ent)(oa_ent (u_isn u_hdip))))(u_isn oa_wa))(u_isn u_hdip)))	29	12
7239	((((cp_ent ca_ent)))((ca_wa p_re) cp_ent)((oa_ent oa_wa) u_hdip)))(u_isn u_hdip))(u_isn oa_wa)))	26	12
7240	((((cp_ent ca_ent)))((ca_wa p_re) cp_ent)((oa_ent oa_wa) u_hdip)))(u_isn u_hdip))(u_isn u_hdip)))	22	12
7241	(((cp_ent ca_ent)))((ca_wa p_re) cp_ent)((oa_ent u_isn) u_hdip)))(u_isn oa_wa))(u_isn oa_wa)))	30	12
7242	((((cp_ent ca_ent)))((ca_wa p_re) cp_ent)((oa_ent u_isn) u_hdip)))(u_isn oa_wa))(u_isn u_hdip)))	31	12
7243	(((cp_ent ca_ent)))((ca_wa p_re)(p_csn oa_ent))(u_isn u_hdip)))(u_isn oa_wa))(u_isn u_hdip)))	23	12
7244	((((cp_ent ca_ent)))((p_re ca_wa)(cp_ent oa_ent))(oa_wa u_hdip)))(u_isn u_hdip))(u_isn oa_wa)))	28	12
7245	((((cp_ent ca_ent)))((p_re ca_wa)(cp_ent oa_ent))(oa_wa u_hdip)))(u_isn u_hdip))(u_isn u_hdip)))	27	12
7246	((((cp_ent ca_ent)))((p_re ca_wa)(cp_ent oa_ent))(u_hdip u_hdip)))(u_isn oa_wa))(u_csn cp_ent)))	25	12
7247	((((cp_ent ca_wa)))((p_re ca_wa)(cp_ent oa_ent))(oa_wa u_hdip)))(u_isn u_hdip))(u_isn oa_wa)))	25	12
7248	((((cp_ent ca_wa)))((p_re ca_wa)(cp_ent oa_ent))(oa_wa u_hdip)))(u_isn u_hdip))(u_isn u_hdip)))	24	12
7249	((((cp_ent ca_wa)))((p_re ca_wa)(cp_ent oa_ent))(oa_wa u_hdip)))(u_isn u_hdip))(u_isn oa_wa)))	27	12
7250	((((cp_ent ca_wa)))((p_re ca_wa)(cp_ent oa_ent))(oa_wa u_hdip)))(u_isn u_hdip))(u_isn u_hdip)))	26	12
7251	((((p_isn ca_ent)))((ca_wa ca_wa)(cp_ent oa_ent))(u_hdip u_hdip)))(u_isn u_hdip))(oa_wa u_csn)))	22	12
7252	((((p_isn ca_ent)))((ca_wa ca_wa)(cp_ent oa_ent))(u_hdip u_hdip)))(u_isn u_hdip))(u_isn oa_wa)))	21	12
7253	((((p_isn ca_ent)))((ca_wa p_re) ca_wa) oa_ent)))(oa_wa u_hdip)(u_isn oa_wa)))(u_csn cp_ent)))	17	12
7254	((((p_isn ca_ent)))((ca_wa p_re) ca_wa)(cp_ent oa_ent)))(u_isn u_hdip)))(u_isn oa_wa) u_hdip))	29	12
7255	((((p_isn ca_ent)))((ca_wa p_re) cp_ent)(oa_ent (u_isn u_hdip))))(u_isn oa_wa))(u_isn oa_wa)))	25	12
7256	((((p_isn ca_ent)))((ca_wa p_re) cp_ent)(oa_ent (u_isn u_hdip))))(u_isn oa_wa))(u_isn u_hdip)))	26	12
7257	((((p_isn ca_ent)))((p_re ca_wa)(cp_ent oa_ent))(oa_wa u_hdip)))(u_isn oa_wa))(u_csn cp_ent)))	21	12
7258	((((u_csn (cp_ent ca_ent)))((ca_wa p_re) ca_wa)(cp_ent oa_ent)))(u_isn u_hdip))(u_isn oa_wa)))	26	12
7259	((((u_csn cp_ent)))((ca_ent ca_wa) cp_ent)(oa_ent (u_isn u_hdip))))(u_isn u_hdip))(u_isn u_hdip)))	23	12
7260	(((u_isn u_hdip)((u_isn oa_wa))((u_csn cp_ent) p_csn))) ca_ent)((ca_wa p_re) ca_wa) oa_ent)))	17	12
7261	(((u_isn u_hdip)((u_isn oa_wa))((u_csn cp_ent) p_csn)))(ca_ent ca_wa)))((p_re ca_wa) oa_ent)))	17	12
7262	(((u_isn u_hdip)((u_isn u_hdip)((u_isn oa_wa) u_csn))))(u_isn oa_wa) u_hdip))(u_isn u_hdip))	22	12
7263	((((u_csn (cp_ent ca_wa)))((p_re ca_wa))((cp_ent oa_ent) u_hdip)))(u_isn u_hdip))(u_isn u_hdip)))	24	12
7264	((((u_hdip (u_csn cp_ent)))((ca_ent ca_wa))((p_isn ca_wa) p_csn)))(oa_ent u_hdip))(u_isn u_hdip)))	18	12
7265	((((u_hdip (u_isn oa_wa)))((u_csn cp_ent))((p_isn oa_ent) oa_wa)))(u_isn u_hdip))(u_isn u_hdip)))	20	12
7266	((((p_csn (oa_ent (u_isn u_hdip)))(u_isn oa_wa)))((u_csn cp_ent) p_csn)) ca_ent)))(ca_wa ca_wa)))	25	12
7267	((((p_csn (oa_ent u_hdip))(u_isn oa_wa)))((u_csn cp_ent) p_csn)) ca_ent)((ca_wa p_re) ca_wa)))	25	12
7268	((((p_csn (oa_ent (u_isn u_hdip)))((u_isn oa_wa) u_hdip)))((u_csn cp_ent) p_csn))(ca_ent p_isn)))	21	12
7269	(((p_csn ((oa_ent u_isn) u_hdip))(u_isn oa_wa))((u_csn cp_ent)))((p_csn ca_ent)(ca_wa p_re)))	23	12
7270	((((cp_ent ca_ent)((ca_wa ca_wa)(p_csn oa_ent)))((u_hdip u_hdip))(u_isn u_hdip))(u_isn u_hdip)))	19	12
7271	((((cp_ent ca_ent)((ca_wa p_re) cp_ent)) oa_ent))((oa_wa u_hdip)(u_isn oa_wa)))(u_csn cp_ent)))	25	12
7272	((((cp_ent ca_ent)((ca_wa p_re) cp_ent)))((oa_ent oa_wa))(u_isn oa_wa)))(u_hdip (u_isn u_csn)))	19	12
7273	((((cp_ent ca_ent)((ca_wa p_re) cp_ent)))((oa_ent u_isn) u_hdip))(u_isn oa_wa)))(u_csn cp_ent)))	27	12
7274	((((cp_ent ca_ent)((ca_wa p_re) cp_ent)))(oa_ent (oa_wa u_hdip)))(u_isn u_hdip))(u_isn oa_wa)))	27	12
7275	((((cp_ent ca_ent)((ca_wa p_re) cp_ent)))(oa_ent (oa_wa u_hdip)))(u_isn u_hdip))(u_isn u_hdip)))	26	12
7276	((((cp_ent ca_ent)((ca_wa p_re) cp_ent)))(oa_ent (u_isn u_hdip)))(u_isn oa_wa))(u_isn oa_wa)))	29	12
7277	((((cp_ent ca_ent)((ca_wa p_re) cp_ent)))(oa_ent (u_isn u_hdip)))(u_isn oa_wa))(u_isn u_hdip)))	30	12
7278	((((cp_ent ca_ent)((ca_wa p_re) cp_ent)))((oa_ent oa_wa) u_hdip)))(u_isn u_hdip))(u_isn u_hdip)))	23	12
7279	((((cp_ent ca_ent)((ca_wa p_re) cp_ent)))((oa_ent u_isn) u_hdip))(u_isn oa_wa))(u_isn oa_wa)))	31	12
7280	((((cp_ent ca_ent)((ca_wa p_re) cp_ent)))((oa_ent u_isn) u_hdip))(u_isn oa_wa))(u_isn u_hdip)))	32	12
7281	((((cp_ent ca_ent)((ca_wa p_re) cp_ent)))((oa_ent u_isn) u_hdip))(u_isn u_hdip))(u_isn u_hdip)))	32	12
7282	((((cp_ent ca_ent)((ca_wa p_re)(p_csn oa_ent)))((u_isn u_hdip))(u_isn oa_wa)))(u_isn u_hdip)))	24	12
7283	((((oa_wa u_csn)((cp_ent ca_wa) cp_ent))((oa_ent u_isn))(u_hdip (u_isn oa_wa)))(u_isn oa_wa)))	18	12
7284	((((p_csn (ca_ent ca_wa)))((p_re ca_wa) p_csn)))(cp_ent oa_ent))(u_isn u_hdip))(u_isn oa_wa)))	19	12
7285	((((p_csn oa_ent)((u_isn oa_wa) u_hdip))(u_isn oa_wa)))(u_csn cp_ent) p_csn))(ca_ent p_isn)))	21	12
7286	((((p_csn oa_ent)((u_isn oa_wa) u_hdip))(u_isn oa_wa)))(u_csn cp_ent) p_csn))(ca_ent ca_wa)))	21	12
7287	((((p_isn ca_ent)((ca_wa p_re))(p_csn oa_ent)))(u_isn u_hdip))(u_isn oa_wa))(u_isn u_hdip)))	22	12
7288	(((((ca_wa p_re) ca_wa))(p_csn (oa_ent u_isn)))(u_hdip u_hdip))(u_isn u_hdip))(u_isn u_hdip)))	24	12
7289	((((p_re ca_wa)((cp_ent oa_ent) oa_wa))(u_isn oa_wa))(u_hdip (u_isn u_csn))))(u_isn cp_ent)))	17	12
7290	(((((ca_wa p_re) ca_wa))((cp_ent oa_ent) u_hdip))(u_isn u_hdip))(u_isn u_hdip))(oa_wa u_csn)))	31	12
7291	(((((ca_wa p_re) ca_wa))((cp_ent oa_ent) u_hdip))(u_isn u_hdip))(u_isn u_hdip))(u_isn oa_wa)))	31	12
7292	(((((ca_wa p_re) ca_wa))((cp_ent oa_ent) u_hdip))(u_isn u_hdip))(u_isn u_hdip))(u_isn u_hdip)))	30	12
7293	((((p_re ca_wa)((cp_ent oa_ent)(oa_wa u_hdip)))(u_isn u_hdip))(u_isn u_hdip))(u_isn oa_wa)))	27	12
7294	(((((ca_wa p_re) ca_wa))(p_csn oa_ent) u_hdip))(u_isn u_hdip))(u_isn u_hdip))(u_isn u_hdip)))	23	12
7295	((((u_csn (cp_ent ca_ent)))((ca_wa p_re) ca_wa))(cp_ent oa_ent)))(u_isn oa_wa))(u_isn oa_wa)))	27	12
7296	((((u_csn (cp_ent ca_ent)))((ca_wa p_re) ca_wa))(cp_ent oa_ent)))(u_isn oa_wa))(u_isn u_hdip)))	26	12
7297	((((u_csn (cp_ent ca_ent)))((ca_wa p_re) ca_wa))(cp_ent oa_ent)))(u_isn u_hdip))(u_isn oa_wa)))	27	12
7298	((((u_csn (cp_ent ca_ent)))((ca_wa p_re) ca_wa))(cp_ent oa_ent)))(u_isn u_hdip))(u_isn u_hdip)))	32	12
7299	((((u_csn cp_ent)((ca_ent ca_wa) p_re))(p_isn ca_wa)))(cp_ent oa_ent) u_hdip))(u_isn u_hdip)))	24	12
7300	((((u_csn cp_ent)((ca_ent ca_wa) p_re))(p_isn ca_wa)))(p_csn oa_ent) u_hdip))(u_isn u_hdip)))	21	12
7301	((((p_csn (ca_ent ca_wa)))((p_re (ca_wa p_csn)))(cp_ent oa_ent))(u_isn u_hdip))(u_isn oa_wa)))	18	12
7302	((((p_csn (ca_ent ca_wa)))((p_re ca_wa) p_csn)))(cp_ent oa_ent))(u_isn u_hdip))(u_isn u_hdip)))	26	12
7303	((((p_csn (ca_ent ca_wa)))((p_re ca_wa) p_csn)))(p_isn oa_ent))(u_isn u_hdip))(u_isn u_hdip)))	22	12
7304	((((p_csn (oa_ent u_hdip))(u_isn oa_wa))((u_csn cp_ent)))((p_csn (ca_ent ca_wa)))(p_re ca_wa)))	23	12
7305	((((p_csn (oa_ent u_isn))(u_hdip u_hdip))(u_isn oa_wa)))(u_csn cp_ent) p_csn))(ca_ent p_isn)))	24	12
7306	((((p_csn (oa_ent u_isn))(u_hdip u_hdip))(u_isn oa_wa)))(u_csn cp_ent) p_csn))(ca_ent ca_wa)))	24	12
7307	(((((u_csn cp_ent)(ca_ent ca_wa))(p_isn ca_wa))(cp_ent oa_ent)))(u_isn oa_wa))(u_isn oa_wa)))	26	12
7308	(((((u_csn cp_ent)(ca_ent ca_wa))(p_isn ca_wa))(cp_ent oa_ent)))(u_isn u_hdip))(u_isn oa_wa)))	26	12
7309	((((u_csn (ca_ent ca_wa))(p_isn ca_wa)))(oa_ent u_isn) u_hdip))(u_isn u_hdip))(u_isn u_hdip)))	22	12
7310	((((u_csn (p_isn ca_ent))(ca_wa p_re))(p_isn ca_wa)))(cp_ent oa_ent) u_hdip))(u_isn u_hdip)))	22	12
7311	((((u_csn (p_isn ca_ent))(ca_wa p_re))(p_isn ca_wa)))(oa_ent u_isn) u_hdip))(u_isn u_hdip)))	22	12
7312	((((u_csn (p_isn ca_ent))(p_isn ca_wa)))(cp_ent oa_ent) u_hdip))(u_isn u_hdip))(u_isn u_hdip)))	20	12
7313	((((u_csn (u_isn cp_ent))(p_isn ca_wa))(p_re ca_wa)))(cp_ent oa_ent) oa_wa))(u_isn oa_wa)))	17	12
7314	((((u_csn (u_isn cp_ent))(p_isn ca_wa))(p_re ca_wa)))(cp_ent oa_ent) oa_wa))(u_isn u_hdip)))	18	12
7315	((((u_hdip (u_csn cp_ent))(p_isn ca_ent)))((ca_wa p_re) cp_ent))(oa_ent oa_wa))(u_isn u_hdip)))	16	12
7316	((((u_hdip (u_csn cp_ent))(p_isn ca_wa))(p_isn ca_wa)))(cp_ent oa_ent) u_hdip))(u_isn oa_wa)))	18	12
7317	((((u_hdip (u_csn cp_ent))(p_isn ca_wa))(p_isn ca_wa)))(cp_ent oa_ent) oa_wa))(u_isn u_hdip)))	18	12
7318	((((u_hdip (u_csn cp_ent))(p_isn ca_wa))(p_isn ca_wa)))(oa_ent u_isn) u_hdip))(u_isn oa_wa)))	18	12
7319	((((u_hdip (u_isn oa_wa)) u_csn)(cp_ent ca_wa)))((p_re ca_wa)(cp_ent oa_ent)))(oa_wa u_hdip)))	21	12
7320	((((u_hdip (u_isn u_csn))(cp_ent ca_ent)))((ca_wa p_re) cp_ent))(oa_ent oa_wa))(u_isn u_hdip)))	18	12
7321	(((((ca_ent ca_wa) cp_ent)((oa_ent u_isn) u_hdip))(u_isn u_hdip))(u_isn u_hdip))(u_isn oa_wa)))	29	12
7322	(((((ca_ent ca_wa) cp_ent)((oa_ent u_isn) u_hdip))(u_isn u_hdip))(u_isn u_hdip))(u_isn u_hdip)))	29	12

7323	(((((ca_ent ca_wa) p_re)(p_csn (oa_ent u_isn)))(u_hdip u_hdip))(u_isn u_hdip))(u_isn u_hdip))	20	12
7324	(((((ca_ent p_re)(cp_ent oa_ent))(oa_wa u_hdip))(u_isn u_hdip))(u_isn u_hdip))(u_isn oa_wa))	18	12
7325	(((((ca_ent p_re)(cp_ent oa_ent))(u_hdip u_hdip))(u_isn u_hdip))(u_isn oa_wa))(u_csn cp_ent))	22	12
7326	(((((ca_ent p_re)(cp_ent oa_ent))(u_hdip u_hdip))(u_isn u_hdip))(u_isn u_hdip))(u_csn cp_ent))	19	12
7327	(((((ca_ent p_re)(cp_ent oa_ent))(u_hdip u_hdip))(u_isn u_hdip))(u_isn u_hdip))(u_isn oa_wa))	19	12
7328	((((((ca_wa ca_wa)(cp_ent oa_ent))(u_hdip u_hdip))(u_isn u_hdip))(u_isn oa_wa))(u_csn cp_ent))	24	12
7329	((((((ca_wa ca_wa)(cp_ent oa_ent))(u_hdip u_hdip))(u_isn u_hdip))(u_isn u_hdip))(u_csn cp_ent))	24	12
7330	((((((ca_wa ca_wa)(cp_ent oa_ent))(u_hdip u_hdip))(u_isn u_hdip))(u_isn u_hdip))(u_isn oa_wa))	21	12
7331	((((((ca_wa ca_wa)(p_csn oa_ent))(u_hdip u_hdip))(u_isn u_hdip))(u_isn u_hdip))(u_csn cp_ent))	23	12
7332	((((((ca_wa p_re) cp_ent) oa_ent)(u_isn u_hdip))(u_isn u_hdip))(u_isn oa_wa))(u_csn cp_ent))	24	12
7333	(((((ca_wa p_re) ca_wa))(cp_ent oa_ent) oa_wa))(u_hdip u_hdip))(u_isn u_hdip))(oa_wa u_csn))	25	12
7334	(((((ca_wa p_re) ca_wa))(cp_ent oa_ent) oa_wa))(u_hdip u_hdip))(u_isn u_hdip))(u_isn oa_wa))	28	12
7335	(((((ca_wa p_re) ca_wa))(cp_ent oa_ent) oa_wa))(u_isn u_hdip))(u_isn u_hdip))(u_isn oa_wa))	26	12
7336	(((((ca_wa p_re) cp_ent))(oa_ent u_isn) u_hdip))(u_isn u_hdip))(u_isn oa_wa))(u_csn cp_ent))	25	12
7337	(((((ca_wa p_re) cp_ent) oa_ent))(oa_wa u_hdip)(u_isn oa_wa)))(u_csn cp_ent))(oa_ent u_isn))	19	12
7338	(((((ca_wa p_re) cp_ent)(oa_ent oa_wa))(u_isn oa_wa))(u_hdip (u_isn u_csn)))(u_isn cp_ent))	16	12
7339	(((((ca_wa p_re) cp_ent)(oa_ent (oa_wa u_hdip)))(u_isn u_hdip))(u_isn oa_wa))(u_csn cp_ent))	21	12
7340	(((((ca_wa p_re) cp_ent)(oa_ent (oa_wa u_hdip)))(u_isn u_hdip))(u_isn u_hdip))(u_isn oa_wa))	25	12
7341	(((((ca_wa p_re) cp_ent))(oa_ent u_isn) u_hdip))(u_isn u_hdip))(u_isn u_hdip))(u_isn u_hdip))	24	12
7342	(((((cp_ent ca_ent) cp_ent) oa_ent))(oa_wa u_hdip)(u_isn oa_wa)))(u_csn cp_ent))(oa_ent u_isn))	18	12
7343	(((((cp_ent ca_ent) cp_ent))(oa_ent oa_wa) u_hdip)))(u_isn u_hdip))(u_isn u_hdip))(u_isn oa_wa))	21	12
7344	(((((cp_ent ca_ent) cp_ent)(oa_ent (oa_wa u_hdip)))(u_isn u_hdip))(u_isn oa_wa))(u_csn cp_ent))	22	12
7345	(((((cp_ent ca_ent) cp_ent)(oa_ent (u_isn u_hdip)))(u_isn u_hdip))(u_isn u_hdip))(u_isn oa_wa))	26	12
7346	(((((cp_ent ca_ent) cp_ent)(oa_ent (u_isn u_hdip)))(u_isn u_hdip))(u_isn u_hdip))(u_isn u_hdip))	22	12
7347	(((((cp_ent ca_ent) cp_ent))(oa_ent u_isn) u_hdip))(u_isn u_hdip))(u_isn oa_wa))(u_csn cp_ent))	26	12
7348	(((((cp_ent ca_ent) cp_ent))(oa_ent u_isn) u_hdip))(u_isn u_hdip))(u_isn u_hdip))(u_isn oa_wa))	28	12
7349	(((((cp_ent ca_ent) cp_ent))(oa_ent u_isn) u_hdip))(u_isn u_hdip))(u_isn u_hdip))(u_isn u_hdip))	24	12
7350	(((((cp_ent ca_ent)(ca_wa p_re))(cp_ent oa_ent))(oa_wa u_hdip))(u_isn u_hdip))(u_isn oa_wa))	25	12
7351	(((((cp_ent ca_ent)(ca_wa p_re))(cp_ent oa_ent))(oa_wa u_hdip))(u_isn u_hdip))(u_isn u_hdip))	25	12
7352	(((((cp_ent ca_ent)(ca_wa p_re))(cp_ent oa_ent))(u_hdip u_hdip))(u_isn u_hdip))(u_isn oa_wa))	28	12
7353	(((((cp_ent ca_ent)(ca_wa p_re))(cp_ent oa_ent))(u_hdip u_hdip))(u_isn u_hdip))(u_isn u_hdip))	23	12
7354	(((((cp_ent ca_wa) cp_ent)(oa_ent (oa_wa u_hdip)))(u_isn u_hdip))(u_isn oa_wa))(u_csn cp_ent))	22	12
7355	(((((cp_ent ca_wa) cp_ent)(oa_ent (oa_wa u_hdip)))(u_isn u_hdip))(u_isn u_hdip))(u_isn oa_wa))	20	12
7356	(((((cp_ent ca_wa) cp_ent))(oa_ent u_isn) u_hdip))(u_isn u_hdip))(u_isn u_hdip))(u_isn oa_wa))	25	12
7357	(((((cp_ent ca_wa) cp_ent))(oa_ent u_isn) u_hdip))(u_isn u_hdip))(u_isn u_hdip))(u_isn u_hdip))	20	12
7358	(((((p_csn ca_ent)(ca_wa p_re))(ca_wa p_csn))(cp_ent oa_ent))(u_isn u_hdip))(u_isn u_hdip))	25	12
7359	(((((p_csn oa_ent)(u_isn u_hdip))(u_isn u_hdip))(u_isn oa_wa)))(u_csn cp_ent) p_csn)) ca_ent)	23	12
7360	(((((p_csn oa_ent)(u_isn u_hdip))(u_isn u_hdip))(u_isn u_hdip))(u_isn u_hdip))(u_csn cp_ent))	25	12
7361	(((((p_isn ca_ent)(ca_wa p_re))(cp_ent oa_ent))(u_hdip u_hdip))(u_isn u_hdip))(oa_wa u_csn))	25	12
7362	(((((p_isn ca_ent)(ca_wa p_re))(cp_ent oa_ent))(u_hdip u_hdip))(u_isn u_hdip))(u_isn oa_wa))	25	12
7363	(((((p_re ca_wa)(cp_ent oa_ent))(oa_wa u_hdip))(u_isn u_hdip))(u_isn u_hdip))(u_isn oa_wa))	27	12
7364	(((((p_re ca_wa)(cp_ent oa_ent))(u_hdip u_hdip))(u_isn u_hdip))(u_isn oa_wa))(u_csn cp_ent))	27	12
7365	(((((p_re ca_wa)(cp_ent oa_ent))(u_hdip u_hdip))(u_isn u_hdip))(u_isn u_hdip))(u_csn cp_ent))	29	12
7366	(((((p_re ca_wa)(cp_ent oa_ent))(u_hdip u_hdip))(u_isn u_hdip))(u_isn u_hdip))(u_isn oa_wa))	26	12
7367	(((((p_re ca_wa)(p_csn oa_ent))(u_hdip u_hdip))(u_isn u_hdip))(u_isn u_hdip))(u_csn cp_ent))	23	12
7368	(((((u_csn cp_ent)(p_isn ca_ent))(ca_wa p_re))(p_isn ca_wa))(cp_ent oa_ent))(u_hdip u_hdip))	23	12
7369	(((((u_csn cp_ent)(p_isn ca_wa))(p_re ca_wa))(cp_ent oa_ent))(u_isn oa_wa))(u_isn oa_wa))	25	12
7370	(((((u_csn cp_ent)(p_isn ca_wa))(p_re ca_wa))(cp_ent oa_ent))(u_isn oa_wa))(u_isn u_hdip))	25	12
7371	(p_csn ((p_isn ca_ent)((((ca_wa p_re) ca_wa)(cp_ent oa_ent))(u_isn u_hdip))(u_isn u_hdip))))	23	12
7372	(p_csn (((p_isn ca_ent)(((ca_wa p_re) ca_wa)(cp_ent oa_ent)))(u_isn u_hdip))(u_isn u_hdip)))	24	12
7373	((ca_ent ca_wa)(((((p_re ca_wa) p_csn) oa_ent)(u_hdip u_hdip))(u_isn u_hdip))(u_isn u_hdip)))	24	12
7374	((ca_ent ca_wa)(((((p_re ca_wa) p_csn) oa_ent)(u_isn u_hdip))(u_isn oa_wa))(u_isn oa_wa)))	26	12
7375	((ca_ent ca_wa)(((((p_re ca_wa) p_csn) oa_ent)(u_isn u_hdip))(u_isn oa_wa))(u_isn u_hdip)))	28	12
7376	((ca_ent p_isn)(((((p_re ca_wa) p_csn) oa_ent)(u_hdip u_hdip))(u_isn u_hdip))(u_isn u_hdip)))	20	12
7377	((ca_ent p_isn)(((((p_re ca_wa) p_csn) oa_ent)(u_isn u_hdip))(u_isn oa_wa))(u_isn oa_wa)))	24	12
7378	((ca_ent p_isn)(((((p_re ca_wa) p_csn) oa_ent)(u_isn u_hdip))(u_isn oa_wa))(u_isn u_hdip)))	26	12
7379	((cp_ent ca_ent)(((((p_re ca_wa) p_csn) oa_ent)(u_hdip u_hdip))(u_isn u_hdip))(u_isn u_hdip)))	20	12
7380	((cp_ent ca_ent)(((((p_re ca_wa) p_csn) oa_ent)(u_isn u_hdip))(u_isn oa_wa))(u_isn oa_wa)))	24	12
7381	((cp_ent ca_ent)(((((p_re ca_wa) p_csn) oa_ent)(u_isn u_hdip))(u_isn oa_wa))(u_isn u_hdip)))	24	12
7382	((p_isn ca_ent)(((((ca_wa p_re) ca_wa) oa_ent)(oa_wa u_hdip))(u_isn u_hdip))(oa_wa u_csn)))	23	12
7383	((p_isn ca_ent)(((((ca_wa p_re) ca_wa) oa_ent)(u_hdip u_hdip))(u_isn u_hdip))(u_isn oa_wa)))	23	12
7384	((p_isn ca_ent)(((((ca_wa p_re) cp_ent) oa_ent)(u_isn u_hdip))(u_isn u_hdip))(u_isn oa_wa)))	25	12
7385	((p_isn ca_ent)(((((ca_wa p_re) cp_ent) oa_ent)(u_isn u_hdip))(u_isn u_hdip))(u_isn u_hdip)))	28	12
7386	((p_isn ca_ent)(((((p_re ca_wa) p_csn) oa_ent)(u_isn u_hdip))(u_isn oa_wa))(u_isn oa_wa)))	21	12
7387	((p_isn ca_ent)(((((p_re ca_wa) p_csn) oa_ent)(u_isn u_hdip))(u_isn oa_wa))(u_isn u_hdip)))	22	12
7388	((p_isn ca_wa)(((((p_re ca_wa) p_csn) oa_ent)(u_hdip u_hdip))(u_isn u_hdip))(u_isn u_hdip)))	24	12
7389	(((ca_ent ca_wa)((((p_re ca_wa) p_csn) oa_ent)(u_hdip u_hdip))(u_isn u_hdip)))(u_isn u_hdip))	26	12
7390	(((ca_ent ca_wa)((((p_re ca_wa) p_csn) oa_ent)(u_isn u_hdip))(u_isn oa_wa)))(u_isn oa_wa))	27	12
7391	(((ca_ent ca_wa)((((p_re ca_wa) p_csn) oa_ent)(u_isn u_hdip))(u_isn oa_wa)))(u_isn u_hdip))	30	12
7392	(((ca_ent p_isn)(((ca_wa (p_csn cp_ent)) oa_ent)(u_isn u_hdip))(u_isn oa_wa)))(u_isn oa_wa))	19	12
7393	(((cp_ent ca_ent)((((ca_wa p_re) cp_ent) oa_ent)(u_isn u_hdip))(u_isn oa_wa)))(u_isn oa_wa))	29	12
7394	(((cp_ent ca_ent)((((ca_wa p_re) cp_ent) oa_ent)(u_isn u_hdip))(u_isn oa_wa)))(u_isn u_hdip))	27	12
7395	(((cp_ent ca_ent)((((ca_wa p_re) cp_ent) oa_ent)(u_isn u_hdip))(u_isn u_hdip)))(u_isn u_hdip))	30	12
7396	(((cp_ent ca_ent)((((p_re ca_wa) p_csn) oa_ent)(u_isn u_hdip))(u_isn oa_wa)))(u_isn oa_wa))	25	12
7397	(((cp_ent ca_wa)((((p_re ca_wa) p_csn) oa_ent)(u_isn u_hdip))(u_isn oa_wa)))(u_isn oa_wa))	25	12
7398	(((cp_ent ca_wa)((((p_re ca_wa) p_csn) oa_ent)(u_isn u_hdip))(u_isn oa_wa)))(u_isn u_hdip))	25	12
7399	(((p_isn ca_ent)((((ca_wa p_re) ca_wa) oa_ent)(oa_wa u_hdip))(u_isn u_hdip)))(u_isn u_hdip))	18	12
7400	(((p_isn ca_ent)((((ca_wa p_re) ca_wa) oa_ent)(u_hdip u_hdip))(u_isn u_hdip)))(u_csn cp_ent))	24	12
7401	(((p_isn ca_ent)((((ca_wa p_re) cp_ent) oa_ent)(oa_wa u_hdip))(u_isn u_hdip)))(u_isn oa_wa))	23	12
7402	(((p_isn ca_ent)((((ca_wa p_re) cp_ent) oa_ent)(oa_wa u_hdip))(u_isn u_hdip)))(u_isn u_hdip))	20	12
7403	(((p_isn ca_ent)((((ca_wa p_re) cp_ent) oa_ent)(u_isn u_hdip))(u_isn oa_wa)))(u_isn oa_wa))	27	12
7404	(((p_isn ca_ent)((((ca_wa p_re) cp_ent) oa_ent)(u_isn u_hdip))(u_isn u_hdip)))(u_isn u_hdip))	30	12
7405	(((p_isn ca_ent)((((p_re ca_wa) p_csn) oa_ent)(u_isn u_hdip))(u_isn oa_wa)))(u_isn oa_wa))	24	12
7406	(((p_isn ca_ent)((((p_re ca_wa) p_csn) oa_ent)(u_isn u_hdip))(u_isn oa_wa)))(u_isn u_hdip))	24	12
7407	(((p_isn ca_wa)((((p_re ca_wa) p_csn) oa_ent)(u_isn u_hdip))(u_isn u_hdip)))(u_isn u_hdip))	31	12
7408	((((ca_ent p_isn)(((p_re ca_wa) p_csn) oa_ent)(u_hdip u_hdip)))(u_isn u_hdip))(u_isn u_hdip))	25	12
7409	((((cp_ent ca_ent)(((ca_wa p_re) ca_wa) oa_ent)(u_hdip u_hdip)))(u_isn u_hdip))(oa_wa u_csn))	26	12
7410	((((cp_ent ca_ent)(((ca_wa p_re) ca_wa) oa_ent)(u_hdip u_hdip)))(u_isn u_hdip))(u_isn oa_wa))	26	12
7411	((((cp_ent ca_ent)(((ca_wa p_re) ca_wa) oa_ent)(u_hdip u_hdip)))(u_isn u_hdip))(u_isn u_hdip))	21	12
7412	((((cp_ent ca_ent)(((ca_wa p_re) ca_wa) oa_ent)(u_isn u_hdip)))(u_isn oa_wa))(u_csn cp_ent))	22	12
7413	((((cp_ent ca_ent)(((ca_wa p_re) cp_ent) oa_ent)(oa_wa u_hdip)))(u_isn u_hdip))(u_isn oa_wa))	27	12
7414	((((cp_ent ca_ent)(((ca_wa p_re) cp_ent) oa_ent)(oa_wa u_hdip)))(u_isn u_hdip))(u_isn u_hdip))	26	12
7415	((((cp_ent ca_ent)(((ca_wa p_re) cp_ent) oa_ent)(u_hdip u_hdip)))(u_isn u_hdip))(u_isn oa_wa))	30	12
7416	((((cp_ent ca_ent)(((ca_wa p_re) cp_ent) oa_ent)(u_hdip u_hdip)))(u_isn u_hdip))(u_isn u_hdip))	28	12
7417	((((cp_ent ca_ent)(((ca_wa p_re) cp_ent) oa_ent)(u_isn u_hdip)))(u_isn oa_wa))(u_isn oa_wa))	30	12
7418	((((cp_ent ca_ent)(((p_re ca_wa) p_csn) oa_ent)(u_hdip u_hdip)))(u_isn u_hdip))(u_isn u_hdip))	23	12
7419	((((p_isn ca_ent)(((ca_wa p_re) cp_ent) oa_ent)(oa_wa u_hdip)))(u_isn u_hdip))(u_isn oa_wa))	22	12
7420	((((p_isn ca_ent)(((ca_wa p_re) cp_ent) oa_ent)(oa_wa u_hdip)))(u_isn u_hdip))(u_isn u_hdip))	19	12
7421	((((p_isn ca_ent)(((ca_wa p_re) cp_ent) oa_ent)(u_isn oa_wa)))(u_isn oa_wa))(u_isn u_hdip))	23	12
7422	((((p_isn ca_ent)(((ca_wa p_re) cp_ent) oa_ent)(u_isn u_hdip)))(u_isn oa_wa))(u_isn oa_wa))	25	12
7423	((((p_isn ca_ent)(((ca_wa p_re) cp_ent) oa_ent)(u_isn u_hdip)))(u_isn oa_wa))(u_isn u_hdip))	24	12
7424	(((((cp_ent ca_ent)((p_re (ca_wa p_csn)) oa_ent))(u_hdip u_hdip))(u_isn u_hdip))(u_isn u_hdip))	19	12
7425	(((((cp_ent ca_ent)(((ca_wa p_re) ca_wa) oa_ent))(oa_wa u_hdip))(u_isn u_hdip))(u_isn oa_wa))	25	12
7426	(((((cp_ent ca_ent)(((ca_wa p_re) ca_wa) oa_ent))(oa_wa u_hdip))(u_isn u_hdip))(u_isn u_hdip))	21	12
7427	(((((cp_ent ca_ent)(((ca_wa p_re) ca_wa) oa_ent))(u_hdip u_hdip))(u_isn u_hdip))(oa_wa u_csn))	27	12
7428	(((((cp_ent ca_ent)(((ca_wa p_re) ca_wa) oa_ent))(u_hdip u_hdip))(u_isn u_hdip))(u_isn oa_wa))	27	12
7429	(((((cp_ent ca_ent)(((ca_wa p_re) ca_wa) oa_ent))(u_hdip u_hdip))(u_isn u_hdip))(u_isn u_hdip))	22	12
7430	(((((cp_ent ca_ent)(((ca_wa p_re) ca_wa) oa_ent))(u_isn u_hdip))(u_isn oa_wa))(u_csn cp_ent))	23	12
7431	(((((cp_ent ca_wa)(((p_re ca_wa) p_csn) oa_ent))(u_hdip u_hdip))(u_isn u_hdip))(u_isn u_hdip))	20	12
7432	(((((p_isn ca_ent)(((ca_wa p_re) cp_ent) oa_ent))(oa_wa u_hdip))(u_isn u_hdip))(u_isn oa_wa))	22	12
7433	(((((p_isn ca_ent)(((ca_wa p_re) cp_ent) oa_ent))(u_hdip u_hdip))(u_isn u_hdip))(u_isn oa_wa))	26	12
7434	(((((p_csn (oa_ent (u_isn u_hdip)))(u_isn u_hdip)))(u_csn cp_ent) p_csn)) ca_ent)(ca_wa ca_wa))	23	12
7435	(((((p_csn ((oa_ent u_isn) u_hdip))(u_isn oa_wa))(u_csn cp_ent))(p_csn ca_ent))(ca_wa ca_wa))	24	12
7436	((((((ca_ent ca_wa)((p_re ca_wa) p_csn)) oa_ent)(u_hdip u_hdip))(u_isn u_hdip))(u_isn u_hdip))	25	12
7437	((((((ca_ent ca_wa)((p_re ca_wa) p_csn)) oa_ent)(u_isn u_hdip))(u_isn oa_wa))(u_isn oa_wa))	28	12
7438	((((((ca_ent ca_wa)((p_re ca_wa) p_csn)) oa_ent)(u_isn u_hdip))(u_isn oa_wa))(u_isn u_hdip))	29	12
7439	((((((cp_ent ca_ent)((ca_wa p_re) cp_ent)) oa_ent)(oa_wa u_hdip))(u_isn u_hdip))(u_isn oa_wa))	28	12
7440	((((((cp_ent ca_ent)((ca_wa p_re) cp_ent)) oa_ent)(oa_wa u_hdip))(u_isn u_hdip))(u_isn u_hdip))	27	12
7441	((((((cp_ent ca_ent)((ca_wa p_re) cp_ent)) oa_ent)(u_hdip u_hdip))(u_isn u_hdip))(oa_wa u_csn))	29	12
7442	((((((cp_ent ca_ent)((ca_wa p_re) cp_ent)) oa_ent)(u_hdip u_hdip))(u_isn u_hdip))(u_isn oa_wa))	31	12
7443	((((((cp_ent ca_ent)((ca_wa p_re) cp_ent)) oa_ent)(u_hdip u_hdip))(u_isn u_hdip))(u_isn u_hdip))	29	12
7444	((((((ca_wa p_re) ca_wa) oa_ent)(oa_wa u_hdip))(u_isn u_hdip))(u_isn oa_wa))(u_csn cp_ent))	22	12
7445	((((((ca_wa p_re) ca_wa) oa_ent)(oa_wa u_hdip))(u_isn u_hdip))(u_isn u_hdip))(u_isn oa_wa))	24	12
7446	((((((ca_wa p_re) ca_wa) oa_ent)(u_hdip u_hdip))(u_isn u_hdip))(u_isn u_hdip))(u_csn cp_ent))	27	12
7447	((((((ca_wa p_re) ca_wa) oa_ent)(u_hdip u_hdip))(u_isn u_hdip))(u_isn u_hdip))(u_isn oa_wa))	23	12
7448	((((((ca_wa p_re) cp_ent) oa_ent)(oa_wa u_hdip))(u_isn u_hdip))(u_isn u_hdip))(u_isn oa_wa))	26	12
7449	((((((ca_wa p_re) cp_ent) oa_ent)(u_hdip u_hdip))(u_isn u_hdip))(u_isn oa_wa))(u_csn cp_ent))	26	12
7450	((((((ca_wa p_re) cp_ent) oa_ent)(u_hdip u_hdip))(u_isn u_hdip))(u_isn u_hdip))(u_csn cp_ent))	28	12
7451	((((((ca_wa p_re) cp_ent) oa_ent)(u_hdip u_hdip))(u_isn u_hdip))(u_isn u_hdip))(u_isn oa_wa))	25	12
7452	((((((cp_ent ca_ent) cp_ent) oa_ent)(u_hdip u_hdip))(u_isn u_hdip))(u_isn u_hdip))(u_csn cp_ent))	25	12
7453	((((((cp_ent ca_ent) cp_ent) oa_ent)(u_hdip u_hdip))(u_isn u_hdip))(u_isn u_hdip))(u_isn oa_wa))	24	12
7454	((((((cp_ent ca_wa) cp_ent) oa_ent)(oa_wa u_hdip))(u_isn u_hdip))(u_isn oa_wa))(u_csn cp_ent))	23	12
7455	((((((cp_ent ca_wa) cp_ent) oa_ent)(oa_wa u_hdip))(u_isn u_hdip))(u_isn u_hdip))(u_isn oa_wa))	21	12
7456	((((((cp_ent ca_wa) cp_ent) oa_ent)(u_hdip u_hdip))(u_isn u_hdip))(u_isn oa_wa))(u_csn cp_ent))	25	12
7457	((((((cp_ent ca_wa) cp_ent) oa_ent)(u_hdip u_hdip))(u_isn u_hdip))(u_isn u_hdip))(u_csn cp_ent))	25	12
7458	((((((cp_ent ca_wa) cp_ent) oa_ent)(u_hdip u_hdip))(u_isn u_hdip))(u_isn u_hdip))(u_isn oa_wa))	24	12
7459	((((((cp_ent oa_ent) oa_wa) u_csn)(u_isn cp_ent))(ca_ent ca_wa))(p_isn p_re))(ca_wa p_csn))	19	12
7460	(((oa_wa (u_csn cp_ent))(ca_ent ca_wa))(((p_re ca_wa)(cp_ent oa_ent)))(oa_wa u_hdip)(u_isn oa_wa))))	21	13
7461	((oa_wa u_hdip)(u_isn oa_wa)))(((u_csn cp_ent)(p_csn (ca_ent ca_wa)))((p_re ca_wa)(cp_ent oa_ent)))	17	13
7462	(((u_isn cp_ent)(ca_ent ca_wa))((p_isn p_re)))(ca_wa (p_csn cp_ent))(oa_ent u_hdip))(u_isn u_hdip)))	17	13
7463	(((oa_wa u_hdip)(((u_csn cp_ent) p_csn)(ca_ent ca_wa)))(((p_re ca_wa)(cp_ent oa_ent))(u_hdip u_hdip)))	20	13
7464	(((u_csn cp_ent)(p_csn (p_isn ca_ent)))((((ca_wa p_re) ca_wa)(cp_ent oa_ent) u_hdip))(u_isn u_hdip)))	18	13
7465	(((u_hdip (u_isn oa_wa)))((u_csn cp_ent) p_csn)(ca_ent ca_wa)))((p_re ca_wa)(cp_ent oa_ent) u_hdip)))	20	13
7466	(((u_hdip (u_isn oa_wa))(((u_csn cp_ent) p_csn) ca_ent)))((ca_wa p_re) cp_ent))(oa_ent u_isn) u_hdip)))	20	13
7467	((u_hdip oa_wa)(((u_csn cp_ent) p_csn) ca_ent))(((ca_wa p_re) ca_wa)(cp_ent oa_ent))(u_hdip u_hdip)))	19	13
7468	(((u_hdip u_hdip)((u_csn cp_ent)(p_csn (ca_ent ca_wa)))(((p_re ca_wa)(cp_ent oa_ent))(u_hdip u_hdip)))	17	13
7469	((oa_wa (u_csn cp_ent))(p_isn ca_ent)))(((ca_wa p_re) ca_wa))(cp_ent oa_ent) u_hdip))(u_isn u_hdip)))	22	13
7470	((((oa_wa (u_csn cp_ent))(p_isn ca_ent)))((ca_wa p_re) ca_wa))(p_csn oa_ent) u_hdip)))(u_isn u_hdip))	19	13
7471	(((oa_wa (u_csn cp_ent))(p_isn ca_wa)))((p_re ca_wa)(cp_ent oa_ent)))(oa_wa u_hdip)(u_isn oa_wa)))	18	13
7472	(((u_hdip (u_csn cp_ent))(p_csn (p_isn ca_ent))))((ca_wa p_re) cp_ent)(oa_ent u_hdip))(u_isn u_hdip)))	16	13
7473	(((u_hdip (u_csn cp_ent))(p_csn (p_isn ca_wa))))((p_re ca_wa))(cp_ent oa_ent) u_hdip))(u_isn u_hdip)))	17	13
7474	(((u_hdip (u_isn oa_wa))(u_csn (cp_ent ca_ent))))((ca_wa p_re) ca_wa)(cp_ent oa_ent))(oa_wa u_hdip)))	20	13
7475	(((u_hdip (u_isn oa_wa))(u_csn cp_ent)(ca_ent ca_wa))))((p_re ca_wa)(cp_ent oa_ent))(oa_wa u_hdip)))	19	13
7476	(((u_hdip (u_isn oa_wa))(u_csn (cp_ent ca_ent))))((ca_wa p_re) ca_wa)(cp_ent oa_ent))(u_hdip u_hdip)))	23	13
7477	((((oa_wa u_hdip)(u_isn oa_wa)))((u_csn cp_ent) p_csn) ca_ent)))((ca_wa p_re) cp_ent)(oa_ent u_isn)))	17	13
7478	((((u_csn cp_ent) p_csn)(p_isn ca_ent)))(((ca_wa p_re) ca_wa))(cp_ent oa_ent) u_hdip))(u_isn u_hdip)))	20	13
7479	(((((u_isn cp_ent)(p_isn ca_ent))((ca_wa p_re) ca_wa))(p_csn cp_ent))(oa_ent u_hdip))(u_isn u_hdip))	15	13
7480	(((u_isn oa_wa)((u_csn cp_ent) p_csn)) ca_ent))((ca_wa p_re) ca_wa)(cp_ent oa_ent))(u_hdip u_hdip)))	20	13
7481	((((u_isn oa_wa) u_hdip)(u_csn (cp_ent ca_ent)))))((ca_wa p_re) ca_wa)(cp_ent oa_ent))(u_hdip u_hdip)))	21	13
7482	((((u_isn oa_wa) u_hdip)(u_csn cp_ent)(p_csn (ca_ent ca_wa)))((p_re ca_wa)(cp_ent oa_ent) u_hdip)))	20	13
7483	((((u_isn oa_wa) u_hdip)((u_csn cp_ent)(p_csn ca_ent))))((ca_wa p_re) cp_ent)((oa_ent u_isn) u_hdip)))	20	13
7484	((oa_wa u_hdip)(((u_csn cp_ent)(p_csn (p_isn ca_ent))))((ca_wa p_re) cp_ent)((oa_ent u_isn) u_hdip)))	16	13
7485	((u_csn (cp_ent ca_ent))(((((ca_wa p_re) ca_wa)(cp_ent oa_ent))(u_isn u_hdip))((u_isn oa_wa) u_hdip)))	23	13
7486	((u_csn (cp_ent ca_ent))((((ca_wa p_re) ca_wa)(p_csn ((p_isn oa_ent)(u_isn u_hdip))))(u_isn u_hdip)))	19	13
7487	((u_hdip u_hdip)((oa_wa (u_csn cp_ent))(p_csn ca_ent)))(((ca_wa p_re) ca_wa)(cp_ent oa_ent) u_hdip)))	20	13
7488	((u_hdip u_hdip)(((u_isn oa_wa) u_csn)(cp_ent ca_ent)))(((ca_wa p_re) ca_wa)(cp_ent oa_ent) u_hdip)))	24	13
7489	((u_hdip u_hdip)(((u_isn oa_wa) u_csn)(cp_ent ca_ent)))(((ca_wa p_re) ca_wa)(cp_ent oa_ent) oa_wa)))	18	13
7490	((u_hdip u_hdip)(((u_isn oa_wa) u_csn)(ca_ent ca_wa)))(((p_re ca_wa)(cp_ent oa_ent))(oa_wa u_hdip)))	18	13
7491	((u_hdip u_hdip)(((u_isn oa_wa) u_csn)(cp_ent ca_ent)))((ca_wa p_re) cp_ent)((oa_ent u_isn) u_hdip)))	24	13
7492	((u_hdip u_hdip)(((u_isn oa_wa) u_csn)(cp_ent ca_wa)))((p_re ca_wa)(cp_ent oa_ent))(oa_wa u_hdip))))	18	13
7493	((u_hdip u_hdip)(((u_isn oa_wa) u_csn)(cp_ent ca_wa)))((p_re ca_wa)(cp_ent oa_ent))(u_hdip u_hdip))))	18	13
7494	((u_hdip u_hdip)(((u_isn oa_wa) u_csn)(cp_ent ca_wa)))((p_re ca_wa)(cp_ent oa_ent))(oa_wa u_hdip))))	17	13
7495	((u_hdip u_hdip)(((u_isn oa_wa)((u_csn cp_ent) p_csn)) ca_ent)))((ca_wa p_re) ca_wa)(cp_ent oa_ent)))	19	13
7496	((u_isn u_hdip)(((u_isn oa_wa) u_csn)(cp_ent ca_ent)))((ca_wa p_re) cp_ent)((oa_ent u_isn) u_hdip)))	20	13
7497	((u_isn u_hdip)(((u_isn oa_wa)((u_csn cp_ent) p_csn)) ca_ent)))((ca_wa p_re) ca_wa)(cp_ent oa_ent)))	18	13
7498	((oa_wa (u_csn (cp_ent ca_ent)))((((ca_wa p_re) ca_wa)(p_csn oa_ent))(u_isn u_hdip))(u_isn u_hdip)))	22	13
7499	((oa_wa (u_csn (cp_ent ca_ent)))(((ca_wa p_re) ca_wa)(p_csn oa_ent))(u_isn u_hdip)))(u_isn u_hdip)))	23	13
7500	((oa_wa (u_csn (cp_ent ca_ent)))(((ca_wa p_re) cp_ent)((oa_ent u_isn) u_hdip)))((u_isn oa_wa) u_hdip)))	22	13
7501	(((u_csn cp_ent)(p_isn ca_ent)))(((ca_wa p_re) ca_wa)(p_csn (p_isn oa_ent)) u_hdip))(u_isn u_hdip)))	18	13
7502	((u_csn (p_isn ca_ent))((((ca_wa p_re) ca_wa)(cp_ent oa_ent))(u_isn u_hdip)))((u_isn oa_wa) u_hdip)))	19	13
7503	(((u_csn cp_ent)(p_isn ca_ent))(((ca_wa p_re) cp_ent)(p_isn oa_ent)(u_isn u_hdip)))(u_isn u_hdip)))	18	13
7504	((((oa_wa (u_csn cp_ent)) ca_ent)(((ca_wa p_re) ca_wa)(p_csn (oa_ent u_isn)) u_hdip)))(u_isn u_hdip))	24	13
7505	((((oa_wa (u_csn cp_ent)) ca_ent)(((ca_wa p_re) ca_wa)(p_csn oa_ent))(u_isn u_hdip))(u_isn u_hdip)))	23	13
7506	((((oa_wa (u_csn cp_ent)) ca_ent)(((ca_wa p_re) ca_wa)(p_csn (p_isn oa_ent)) u_hdip))(u_isn u_hdip)))	18	13
7507	(((oa_wa (u_csn cp_ent)) ca_ent)((((ca_wa p_re) ca_wa)(cp_ent oa_ent))(oa_wa u_hdip))(u_isn u_hdip)))	21	13
7508	(((oa_wa (u_csn cp_ent)) ca_ent)((((ca_wa p_re) ca_wa)(cp_ent oa_ent)))(oa_wa u_hdip)(u_isn oa_wa)))	20	13
7509	(((oa_wa (u_csn cp_ent))(ca_ent ca_wa))(((p_re ca_wa) p_csn)(oa_ent (u_isn u_hdip)))(u_isn u_hdip)))	22	13
7510	(((oa_wa (u_csn cp_ent))(ca_ent ca_wa))((((p_re ca_wa)(cp_ent oa_ent))(oa_wa u_hdip))(u_isn oa_wa)))	20	13
7511	(((oa_wa (u_csn cp_ent))(ca_ent ca_wa))((((p_re ca_wa)(cp_ent oa_ent))(oa_wa u_hdip))(u_isn u_hdip)))	21	13
7512	(((oa_wa (u_csn cp_ent))(ca_ent ca_wa))((((p_re ca_wa)(cp_ent oa_ent))(u_hdip u_hdip))(u_isn oa_wa)))	20	13
7513	((((oa_wa (u_csn cp_ent)) ca_ent)((((ca_wa p_re) ca_wa)(cp_ent oa_ent))(u_hdip u_hdip)))(u_isn u_hdip))	26	13
7514	(((oa_wa (u_csn cp_ent))(ca_ent ca_wa))((((p_re ca_wa)(cp_ent oa_ent))(u_isn u_hdip))(u_isn oa_wa)))	23	13
7515	(((oa_wa (u_csn cp_ent))(ca_ent ca_wa))((((p_re ca_wa)(cp_ent oa_ent))(u_isn u_hdip))(u_isn u_hdip)))	26	13
7516	(((oa_wa (u_csn cp_ent)) ca_ent)((((ca_wa p_re) ca_wa)(p_csn oa_ent))(u_hdip u_hdip))(u_isn u_hdip)))	20	13
7517	(((oa_wa (u_csn cp_ent)) ca_ent)((((ca_wa p_re) ca_wa)(cp_ent oa_ent))(oa_wa u_hdip)))(u_isn u_hdip))	22	13
7518	((((oa_wa (u_csn cp_ent)) p_csn)(ca_ent ca_wa))(((p_re ca_wa)(cp_ent oa_ent) u_hdip))(u_isn u_hdip)))	20	13
7519	(((oa_wa (u_csn cp_ent))(p_csn ca_ent))((((ca_wa p_re) cp_ent))(oa_ent u_isn) u_hdip))(u_isn u_hdip)))	24	13
7520	((((oa_wa u_csn)(p_isn ca_ent)))((ca_wa p_re) ca_wa))(p_csn (oa_ent u_isn)) u_hdip)))(u_isn u_hdip))	18	13
7521	(((oa_wa (u_csn cp_ent))(p_isn ca_ent))((((ca_wa p_re) ca_wa)(cp_ent oa_ent)))((u_isn oa_wa) u_hdip)))	19	13
7522	(((oa_wa (u_csn cp_ent))(p_isn ca_ent))((((ca_wa p_re) cp_ent)(oa_ent u_hdip)))((u_isn oa_wa) u_hdip)))	18	13
7523	(((oa_wa u_csn)(p_isn ca_ent))((((ca_wa p_re) ca_wa)(cp_ent oa_ent))(u_isn u_hdip))(u_isn u_hdip)))	22	13
7524	((((u_csn cp_ent)(p_isn ca_ent))(((ca_wa p_re) ca_wa)(p_csn oa_ent))(u_isn u_hdip)))(u_isn u_hdip)))	21	13
7525	(((u_csn cp_ent)(p_isn ca_ent))((((ca_wa p_re) ca_wa)(p_csn oa_ent))(u_isn u_hdip))(u_isn u_hdip)))	22	13
7526	((((u_csn cp_ent)(p_isn ca_ent))(((ca_wa p_re) cp_ent)((oa_ent u_isn) u_hdip)))((u_isn oa_wa) u_hdip))	21	13
7527	(((u_csn cp_ent)(p_isn ca_ent))(((((ca_wa p_re) cp_ent) oa_ent)(u_isn u_hdip)))((u_isn oa_wa) u_hdip)))	20	13
7528	((((u_csn cp_ent)(p_isn ca_ent))((((ca_wa p_re) ca_wa)(cp_ent oa_ent))(u_isn u_hdip)))(u_isn u_hdip))	26	13
7529	(((u_csn cp_ent)(p_isn ca_ent))((((ca_wa p_re) ca_wa)(cp_ent oa_ent))(u_hdip u_hdip)))(u_isn u_hdip))	23	13
7530	(((u_hdip oa_wa) u_hdip)((((u_csn cp_ent) p_csn)(ca_ent ca_wa)))((p_re ca_wa))(cp_ent oa_ent) u_hdip)))	17	13
7531	(((u_hdip (u_csn cp_ent))(ca_ent)((((ca_wa p_re) ca_wa)(cp_ent oa_ent))(u_hdip u_hdip))(u_isn u_hdip)))	23	13
7532	(((u_isn oa_wa) u_hdip)(((u_csn cp_ent) p_csn)(ca_ent ca_wa)))((p_re ca_wa))(cp_ent oa_ent) u_hdip)))	19	13
7533	(((u_isn oa_wa) u_hdip)(((u_csn cp_ent) p_csn) ca_ent)))((ca_wa p_re) cp_ent)((oa_ent u_isn) u_hdip)))	19	13
7534	(((u_isn oa_wa) u_hdip)(((u_csn cp_ent)(p_csn (p_isn ca_ent)))((ca_wa p_re) ca_wa)(oa_ent u_isn))))	14	13
7535	(((u_isn oa_wa) u_hdip)(((u_csn cp_ent)(p_csn (p_isn ca_ent))))((ca_wa p_re) ca_wa)(cp_ent oa_ent)))	16	13
7536	(((u_isn oa_wa) u_hdip)(((u_csn cp_ent)(p_csn (p_isn ca_ent))))((ca_wa p_re) cp_ent)(oa_ent u_isn)))	16	13
7537	(((u_isn oa_wa) u_hdip)(((u_csn cp_ent)(p_csn (p_isn ca_ent))))((ca_wa p_re) cp_ent)(oa_ent u_hdip)))	16	13
7538	((((oa_wa u_csn)(p_csn (p_isn ca_ent)))(((ca_wa p_re) cp_ent)((oa_ent u_isn) u_hdip)))(u_isn u_hdip))	17	13
7539	((oa_wa u_hdip)((((u_csn cp_ent) p_csn) ca_ent))(((ca_wa p_re) ca_wa)(oa_ent u_isn))(u_hdip u_hdip)))	18	13
7540	(((oa_wa u_hdip)(((u_csn cp_ent) p_csn) ca_ent))(((ca_wa p_re) cp_ent)(oa_ent u_hdip))(u_isn u_hdip)))	20	13
7541	(((oa_wa u_hdip)(((u_csn cp_ent) p_csn) ca_ent))(((ca_wa p_re) cp_ent)(oa_ent u_isn))(u_hdip u_hdip)))	20	13
7542	((((u_csn cp_ent)(p_csn (p_isn ca_ent)))((((ca_wa p_re) cp_ent)((oa_ent u_isn) u_hdip)))(u_isn u_hdip))	20	13
7543	(((u_hdip oa_wa)((u_csn cp_ent)(ca_ent ca_wa)))((((p_re ca_wa)(cp_ent oa_ent) oa_wa))(u_hdip u_hdip)))	19	13
7544	(((u_hdip oa_wa)((u_csn cp_ent)(ca_ent ca_wa)))((((p_re ca_wa)(cp_ent oa_ent) oa_wa))(u_isn u_hdip)))	20	13
7545	(((u_hdip oa_wa)((u_csn cp_ent)(ca_ent ca_wa)))((((p_re ca_wa)(cp_ent oa_ent) u_hdip))(u_isn u_hdip)))	23	13
7546	(((u_hdip oa_wa)(u_csn (cp_ent ca_ent)))((((ca_wa p_re) cp_ent)((oa_ent u_isn) u_hdip))(u_isn u_hdip)))	24	13
7547	(((u_hdip oa_wa)(u_csn (cp_ent ca_ent)))((((ca_wa p_re) cp_ent)(oa_ent (oa_wa u_hdip))))(u_isn u_hdip)))	17	13
7548	(((u_hdip oa_wa)((u_csn cp_ent)(ca_ent ca_wa)))((((p_re ca_wa) oa_ent)(u_hdip u_hdip))(u_isn u_hdip)))	17	13
7549	(((u_hdip u_hdip)((oa_wa (u_csn cp_ent))(p_csn ca_ent)))((((ca_wa p_re) ca_wa) oa_ent)(u_hdip u_hdip)))	17	13
7550	(((u_hdip (u_isn oa_wa)))((u_csn cp_ent) p_csn) ca_ent)))(((ca_wa p_re) ca_wa) oa_ent)(u_hdip u_hdip)))	17	13
7551	((u_hdip oa_wa)((((u_csn cp_ent) p_csn) ca_ent))(((ca_wa p_re) ca_wa)(oa_ent u_isn))(u_hdip u_hdip)))	17	13
7552	(((u_hdip oa_wa)(((u_csn cp_ent) p_csn) ca_ent))(((ca_wa p_re) cp_ent)(oa_ent u_hdip))(u_isn u_hdip)))	19	13
7553	(((u_hdip oa_wa)(((u_csn cp_ent) p_csn) ca_ent))(((ca_wa p_re) cp_ent)(oa_ent u_isn))(u_hdip u_hdip)))	19	13
7554	(((u_hdip u_hdip)((oa_wa (u_csn cp_ent))(p_csn ca_ent)))((((ca_wa p_re) cp_ent) oa_ent)(u_isn u_hdip)))	20	13
7555	((u_hdip (u_isn oa_wa))((((u_csn cp_ent) p_csn) ca_ent)))((ca_wa p_re) cp_ent) oa_ent)(u_isn u_hdip)))	20	13
7556	(((u_hdip u_hdip)((u_csn cp_ent)(p_csn ca_ent)))((((ca_wa p_re) ca_wa)(oa_ent u_isn))(u_hdip u_hdip)))	16	13
7557	(((u_hdip u_hdip)((u_csn cp_ent)(p_csn ca_ent)))((((ca_wa p_re) cp_ent)(oa_ent u_hdip))(u_isn u_hdip)))	17	13
7558	(((u_hdip u_hdip)((u_csn cp_ent)(p_csn ca_ent)))((((ca_wa p_re) cp_ent)(oa_ent u_isn))(u_hdip u_hdip)))	17	13
7559	(((u_hdip u_hdip)((oa_wa (u_csn cp_ent)) ca_ent))((((ca_wa p_re) ca_wa)(cp_ent oa_ent))(oa_wa u_hdip)))	21	13
7560	(((u_hdip u_hdip)((oa_wa (u_csn cp_ent)) ca_ent))((((ca_wa p_re) ca_wa)(cp_ent oa_ent))(u_hdip u_hdip)))	24	13
7561	(((u_hdip u_hdip)((oa_wa (u_csn cp_ent)) ca_ent))((((ca_wa p_re) ca_wa)(oa_ent u_isn))(u_hdip u_hdip)))	20	13
7562	(((u_hdip u_hdip)((oa_wa (u_csn cp_ent)) ca_ent))((((ca_wa p_re) cp_ent)(oa_ent u_hdip))(u_isn u_hdip)))	24	13
7563	(((u_hdip u_hdip)((oa_wa (u_csn cp_ent)) ca_ent))((((ca_wa p_re) cp_ent)(oa_ent u_isn))(u_hdip u_hdip)))	24	13
7564	(((oa_wa (u_csn cp_ent))(p_isn ca_ent))(((ca_wa p_re))(p_csn oa_ent)(u_isn u_hdip)))(u_isn u_hdip)))	18	13
7565	(((oa_wa (u_csn cp_ent))(p_isn ca_ent))((((ca_wa p_re) ca_wa))(cp_ent oa_ent) oa_wa))(u_isn oa_wa)))	18	13
7566	(((oa_wa (u_csn cp_ent))(p_isn ca_ent))((((ca_wa p_re) ca_wa))(cp_ent oa_ent) u_hdip))(u_isn oa_wa)))	19	13
7567	((((oa_wa (u_csn cp_ent))(p_isn ca_ent)))((ca_wa p_re) cp_ent)((oa_ent u_isn) u_hdip)))(u_isn u_hdip))	24	13
7568	(((oa_wa (u_csn cp_ent))(p_isn ca_ent)))(((ca_wa p_re) cp_ent) oa_ent)((oa_wa u_hdip))(u_isn oa_wa)))	17	13
7569	(((oa_wa (u_csn cp_ent))(p_isn ca_ent))(((ca_wa p_re) cp_ent)((oa_ent u_isn) u_hdip))(u_isn oa_wa)))	20	13
7570	(((oa_wa (u_csn cp_ent))(p_isn ca_ent))(((ca_wa p_re) cp_ent)((oa_ent (u_isn u_hdip))(u_isn oa_wa)))	19	13
7571	((((oa_wa (u_csn cp_ent))(p_isn ca_ent)))((p_re ca_wa) p_csn) oa_ent)(u_isn u_hdip)))(u_isn u_hdip))	20	13
7572	(((oa_wa (u_csn cp_ent))(p_isn ca_ent))(((p_re ca_wa)(cp_ent oa_ent)))(u_isn u_hdip))(u_isn oa_wa)))	18	13
7573	(((oa_wa (u_csn cp_ent))(p_isn ca_ent))(((p_re ca_wa)(cp_ent oa_ent)))(u_isn u_hdip))(u_isn u_hdip)))	21	13
7574	(((oa_wa (u_csn cp_ent))(p_isn ca_wa))((p_re ca_wa))(cp_ent oa_ent)(oa_wa u_hdip)))(u_isn u_hdip)))	20	13
7575	(((oa_wa (u_csn cp_ent))(p_isn ca_wa))(((p_re ca_wa))(cp_ent oa_ent))(oa_wa u_hdip))(u_isn u_hdip)))	20	13
7576	(((oa_wa (u_csn cp_ent))(p_isn ca_wa))(((p_re ca_wa))(cp_ent oa_ent))(u_isn u_hdip))(u_isn oa_wa)))	20	13
7577	(((oa_wa (u_csn cp_ent))(p_csn (p_isn ca_ent)))((((ca_wa p_re) ca_wa)(cp_ent oa_ent))(u_hdip u_hdip)))	18	13
7578	((((u_csn cp_ent)(ca_ent ca_wa))(p_isn ca_wa))((p_csn ((p_isn oa_ent)(u_isn u_hdip)))(u_isn u_hdip)))	22	13
7579	((((u_csn cp_ent)(ca_ent ca_wa))(p_re p_re)))(p_csn ((p_isn oa_ent)(u_isn u_hdip)))(u_isn u_hdip)))	19	13
7580	((((u_csn cp_ent)(p_isn ca_ent))(p_re p_re)))(p_csn ((p_isn oa_ent)(u_isn u_hdip)))(u_isn u_hdip)))	18	13
7581	(((u_csn (u_isn cp_ent)) ca_ent)((((ca_wa p_re) ca_wa)(cp_ent oa_ent))(u_isn u_hdip))(u_isn u_hdip)))	21	13
7582	(((u_csn (u_isn cp_ent))(p_isn ca_ent))((((ca_wa p_re) ca_wa)(cp_ent oa_ent)))((u_isn oa_wa) u_hdip)))	17	13
7583	((((u_csn (u_isn cp_ent))(p_isn ca_ent)))((ca_wa p_re) cp_ent)((oa_ent u_isn) u_hdip)))(u_isn u_hdip))	20	13
7584	(((u_hdip (oa_wa u_csn))(cp_ent ca_ent)))((((ca_wa p_re) cp_ent)((oa_ent u_isn) u_hdip))(u_isn u_hdip)))	22	13
7585	(((u_hdip (oa_wa u_csn))(cp_ent ca_ent)))((((ca_wa p_re) cp_ent)((oa_ent u_isn) u_hdip))(u_isn oa_wa)))	18	13
7586	(((u_hdip (oa_wa u_csn))(cp_ent ca_wa)))((((p_re ca_wa)(cp_ent oa_ent))(u_hdip u_hdip))(u_isn u_hdip)))	18	13
7587	(((u_hdip (u_csn cp_ent))(p_csn (ca_ent ca_wa)))(((p_re ca_wa)(cp_ent oa_ent) u_hdip))(u_isn u_hdip)))	19	13
7588	(((u_hdip (u_csn cp_ent))(p_csn ca_ent))((((ca_wa p_re) cp_ent)((oa_ent u_isn) u_hdip))(u_isn u_hdip)))	20	13
7589	(((u_hdip (u_csn cp_ent))(p_isn ca_ent))((((ca_wa p_re) ca_wa)((cp_ent oa_ent) oa_wa))(u_isn oa_wa)))	17	13
7590	(((u_hdip (u_csn cp_ent))(p_isn ca_ent))((((ca_wa p_re) ca_wa)((cp_ent oa_ent) u_hdip))(u_isn oa_wa)))	17	13
7591	((((u_hdip (u_csn cp_ent))(p_isn ca_ent)))((ca_wa p_re) cp_ent)((oa_ent u_isn) u_hdip)))(u_isn u_hdip))	21	13
7592	(((u_hdip (u_csn cp_ent))(p_csn (p_isn ca_ent))))((((ca_wa p_re) ca_wa)(oa_ent u_isn))(u_hdip u_hdip)))	15	13
7593	(((u_hdip (u_isn oa_wa))(u_csn (cp_ent ca_ent))))((ca_wa p_re) ca_wa)(oa_ent u_isn))(u_hdip u_hdip)))	20	13
7594	(((u_hdip (u_isn oa_wa))(u_csn (cp_ent ca_ent))))((ca_wa p_re) cp_ent)(oa_ent u_hdip))(u_isn u_hdip)))	23	13
7595	(((u_hdip (u_isn oa_wa))(u_csn (cp_ent ca_ent))))((ca_wa p_re) cp_ent)(oa_ent u_isn))(u_hdip u_hdip)))	23	13
7596	(((u_hdip (u_isn u_csn))(cp_ent ca_ent)))((((ca_wa p_re) cp_ent)((oa_ent u_isn) u_hdip))(u_isn u_hdip)))	22	13
7597	(((u_hdip (u_isn u_csn))(cp_ent ca_wa)))((((p_re ca_wa)(cp_ent oa_ent))(u_hdip u_hdip))(u_isn u_hdip)))	17	13
7598	((((oa_wa u_csn) p_csn)(p_isn ca_ent)))(((ca_wa p_re) cp_ent)((oa_ent u_isn) u_hdip)))(u_isn u_hdip))	19	13
7599	((((oa_wa u_hdip) oa_wa)(u_csn (cp_ent ca_ent)))((((ca_wa p_re) ca_wa)(cp_ent oa_ent))(u_isn oa_wa)))	24	13
7600	((((p_csn (oa_ent u_isn)) u_hdip)(u_isn oa_wa)))(((u_csn cp_ent) p_csn)(ca_ent p_isn)))(p_re ca_wa)))	24	13
7601	((((u_csn cp_ent) p_csn)(p_isn ca_ent))(((ca_wa p_re) cp_ent)((oa_ent u_isn) u_hdip)))(u_isn u_hdip))	22	13
7602	((((u_hdip oa_wa) u_csn)(cp_ent ca_ent)))(((ca_wa p_re) ca_wa)(p_csn ((p_isn oa_ent)(u_isn u_hdip))))	18	13
7603	((((u_hdip oa_wa) u_csn)(cp_ent ca_ent)))(((ca_wa p_re))(p_csn oa_ent)(u_isn u_hdip)))(u_isn u_hdip))	18	13
7604	(((((u_hdip oa_wa) u_csn)(cp_ent ca_wa)))((p_re ca_wa)(cp_ent oa_ent)))(oa_wa u_hdip))(u_isn u_hdip))	19	13
7605	(((u_hdip oa_wa) u_csn)(cp_ent ca_ent)))((((ca_wa p_re) cp_ent)((oa_ent u_isn) u_hdip))(u_isn u_hdip)))	25	13
7606	((((u_hdip oa_wa) u_csn)(cp_ent ca_wa)))((((p_re ca_wa)(cp_ent oa_ent))(oa_wa u_hdip)))(u_isn u_hdip))	18	13
7607	((((u_hdip oa_wa) u_csn)(cp_ent ca_ent)))(((ca_wa p_re) cp_ent)(oa_ent (u_isn u_hdip)))(u_isn u_hdip)))	24	13
7608	((((u_hdip oa_wa) u_csn)(cp_ent ca_ent)))(((ca_wa p_re)(p_csn oa_ent))(u_isn u_hdip))(u_isn u_hdip)))	18	13
7609	((((u_hdip oa_wa) u_csn)(ca_ent ca_wa)))(((((p_re ca_wa) p_csn) oa_ent)(u_isn u_hdip))(u_isn u_hdip)))	20	13
7610	((((u_hdip oa_wa) u_csn)(cp_ent ca_wa)))((((p_re ca_wa)(cp_ent oa_ent))(oa_wa u_hdip)))(u_isn u_hdip)))	19	13
7611	((((u_hdip oa_wa) u_csn)(cp_ent ca_wa)))((((p_re ca_wa)(cp_ent oa_ent))(u_hdip u_hdip)))(u_isn u_hdip)))	21	13
7612	((((u_hdip oa_wa) u_csn)(cp_ent ca_wa)))((((p_re ca_wa)(cp_ent oa_ent))(u_isn u_hdip))(u_isn u_hdip)))	19	13
7613	((((u_isn oa_wa) u_csn)(cp_ent ca_ent)))((((ca_wa p_re) ca_wa))(cp_ent oa_ent) oa_wa))(u_hdip u_hdip)))	19	13
7614	((((u_isn oa_wa) u_csn)(cp_ent ca_ent)))((((ca_wa p_re) ca_wa))(cp_ent oa_ent) oa_wa))(u_isn u_hdip)))	20	13
7615	((((u_isn oa_wa) u_csn)(cp_ent ca_ent)))((((ca_wa p_re) ca_wa))(cp_ent oa_ent) u_hdip))(u_isn u_hdip)))	23	13
7616	((((u_isn oa_wa) u_csn)(ca_ent ca_wa)))((((p_re ca_wa)(cp_ent oa_ent))(oa_wa u_hdip))(u_isn u_hdip)))	18	13
7617	((((u_isn oa_wa) u_csn)(cp_ent ca_ent)))(((ca_wa p_re) cp_ent)((oa_ent u_isn) u_hdip)))(u_isn u_hdip))	24	13
7618	((((u_isn oa_wa) u_csn)(cp_ent ca_ent)))(((ca_wa p_re) cp_ent)(oa_ent (oa_wa u_hdip)))(u_isn u_hdip)))	17	13
7619	((((u_isn oa_wa) u_csn)(cp_ent ca_wa)))((((p_re ca_wa)(cp_ent oa_ent))(u_hdip u_hdip))(u_isn u_hdip)))	20	13
7620	(((u_isn oa_wa) u_hdip)(u_csn (cp_ent ca_ent)))((((ca_wa p_re) ca_wa)(cp_ent oa_ent) u_hdip))(u_isn u_hdip)))	21	13
7621	(((u_isn oa_wa) u_hdip)((u_csn cp_ent)(p_csn ca_ent)))((((ca_wa p_re) ca_wa) oa_ent)(u_hdip u_hdip)))	17	13
7622	(((u_isn oa_wa) u_hdip)((u_csn cp_ent)(p_csn ca_ent)))((((ca_wa p_re) cp_ent) oa_ent)(u_isn u_hdip)))	20	13
7623	(((u_isn u_hdip)(oa_wa (u_csn cp_ent))) ca_ent)((((ca_wa p_re) ca_wa)(cp_ent oa_ent))(u_hdip u_hdip)))	20	13
7624	(((u_isn u_hdip)(oa_wa (u_csn cp_ent))) ca_ent)((((ca_wa p_re) cp_ent)(oa_ent u_hdip))(u_isn u_hdip)))	20	13
7625	(((u_isn u_hdip)(oa_wa (u_csn cp_ent))) ca_ent)((((ca_wa p_re) cp_ent)(oa_ent u_isn))(u_hdip u_hdip)))	20	13
7626	(((u_isn u_hdip)(oa_wa (u_csn cp_ent)))(p_csn ca_ent))((((ca_wa p_re) cp_ent) oa_ent)(u_isn u_hdip)))	17	13
7627	(((cp_ent oa_ent)(oa_wa u_hdip)))(u_isn u_hdip))(u_isn oa_wa) u_csn)))(cp_ent oa_ent)(u_isn u_hdip)))	16	13
7628	(((u_hdip u_hdip)(((u_isn oa_wa) u_csn)(cp_ent ca_ent)))(((ca_wa p_re) ca_wa)(cp_ent oa_ent) oa_wa)))	19	13
7629	(((u_hdip u_hdip)(((u_isn oa_wa) u_csn)(ca_ent ca_wa)))(((p_re ca_wa)(cp_ent oa_ent))(oa_wa u_hdip))))	22	13
7630	(((u_hdip u_hdip)(((u_isn oa_wa) u_csn)(ca_ent ca_wa)))(((p_re ca_wa)(cp_ent oa_ent))(oa_wa u_hdip)))	19	13
7631	(((u_hdip u_hdip)(((u_isn oa_wa) u_csn)(cp_ent ca_ent)))(((ca_wa p_re) ca_wa) oa_ent)(u_hdip u_hdip)))	20	13
7632	(((u_hdip u_hdip)(((u_isn oa_wa) u_csn)(cp_ent ca_ent)))(((ca_wa p_re) cp_ent) oa_ent)(u_isn u_hdip)))	24	13
7633	(((u_isn oa_wa)(u_hdip (u_csn cp_ent))(p_csn (ca_ent ca_wa)))(((p_re ca_wa)(cp_ent oa_ent) u_hdip)))	19	13
7634	(((u_isn oa_wa)(u_hdip (u_csn cp_ent))(p_csn ca_ent)))(((ca_wa p_re) cp_ent)((oa_ent u_isn) u_hdip)))	19	13
7635	(((u_isn oa_wa)(u_hdip (u_csn cp_ent))(p_csn ca_ent)))(((ca_wa p_re) cp_ent) oa_ent)(u_isn u_hdip)))	19	13
7636	(((u_isn oa_wa)(u_hdip (u_csn cp_ent))(p_csn (p_isn ca_ent))))((ca_wa p_re) ca_wa)(cp_ent oa_ent)))	15	13
7637	(((u_isn oa_wa)(u_hdip (u_csn cp_ent))(p_csn (p_isn ca_ent))))((ca_wa p_re) cp_ent)(oa_ent u_isn)))	15	13
7638	(((u_isn oa_wa)(((u_csn cp_ent) p_csn)(p_isn ca_wa))))((p_re ca_wa)(cp_ent oa_ent))(u_hdip u_hdip)))	17	13
7639	(((u_isn u_hdip)(((u_isn oa_wa) u_csn)(cp_ent ca_ent)))(((ca_wa p_re) ca_wa) oa_ent)(u_hdip u_hdip)))	16	13
7640	(((u_isn u_hdip)(((u_isn oa_wa) u_csn)(cp_ent ca_ent)))(((ca_wa p_re) cp_ent) oa_ent)(u_isn u_hdip)))	20	13
7641	(((oa_wa (u_csn (cp_ent ca_ent)))((((ca_wa p_re) ca_wa)(cp_ent oa_ent))(u_isn u_hdip)))(u_isn u_hdip))	27	13
7642	(((oa_wa (u_csn (cp_ent ca_ent)))(((ca_wa p_re) cp_ent)((oa_ent u_isn) u_hdip)))((u_isn oa_wa) u_hdip))	23	13
7643	(((u_csn cp_ent)(p_csn (ca_ent ca_wa)))((((p_re ca_wa)(cp_ent oa_ent))(u_hdip u_hdip)))(u_isn u_hdip))	21	13
7644	((((oa_wa (u_csn cp_ent)) ca_ent)(((ca_wa p_re) ca_wa)(cp_ent oa_ent))(u_isn u_hdip)))(u_isn u_hdip))	28	13
7645	((((oa_wa (u_csn cp_ent))(ca_ent ca_wa))(((p_re ca_wa)(cp_ent oa_ent))(u_hdip u_hdip)))(u_isn u_hdip))	24	13
7646	((((oa_wa (u_csn cp_ent))(p_csn ca_ent))(((ca_wa p_re) cp_ent)((oa_ent u_isn) u_hdip)))(u_isn u_hdip))	23	13
7647	((((u_isn oa_wa) u_hdip)((u_csn (cp_ent ca_ent)))((ca_wa p_re) ca_wa)))((p_csn (oa_ent u_isn)) u_hdip))	20	13
7648	((((u_isn oa_wa)((u_csn cp_ent) p_csn)) ca_ent)((((ca_wa p_re) ca_wa)(oa_ent u_isn))(u_hdip u_hdip)))	18	13
7649	((((u_isn oa_wa)((u_csn cp_ent) p_csn)) ca_ent)((((ca_wa p_re) cp_ent)(oa_ent u_hdip))(u_isn u_hdip)))	20	13
7650	((((u_isn oa_wa)((u_csn cp_ent) p_csn)) ca_ent)((((ca_wa p_re) cp_ent)(oa_ent u_isn))(u_hdip u_hdip)))	20	13
7651	((((u_isn oa_wa) u_hdip)(((u_isn oa_wa) u_csn)(cp_ent ca_ent)))(((ca_wa p_re) ca_wa)(cp_ent oa_ent)))	20	13
7652	((((u_isn oa_wa) u_hdip)(((u_isn oa_wa) u_csn)(cp_ent ca_ent)))(((ca_wa p_re) cp_ent)(oa_ent u_isn)))	19	13
7653	((((p_csn (oa_ent (u_isn u_hdip)))(u_isn oa_wa)))((u_csn cp_ent))(p_csn (ca_ent ca_wa))))(p_re ca_wa))	22	13
7654	((((ca_ent p_isn))(p_re ca_wa)(cp_ent oa_ent)))(u_isn oa_wa))(u_hdip (u_isn u_csn))(u_isn cp_ent)))	18	13
7655	((((oa_wa oa_wa)((u_csn cp_ent) p_csn))(p_isn ca_ent))(((ca_wa p_re) cp_ent)((oa_ent u_isn) u_hdip))	14	13
7656	((((oa_wa oa_wa)((u_csn cp_ent) p_csn))(p_isn ca_ent))(((ca_wa p_re) cp_ent) oa_ent)(u_isn u_hdip))	14	13
7657	((((u_csn cp_ent)((ca_ent ca_wa) p_re))(p_isn ca_wa))(((p_csn oa_ent)(u_isn u_hdip))(u_isn u_hdip)))	21	13
7658	((((u_csn cp_ent)(p_isn ca_ent))((ca_wa p_re) ca_wa)))(cp_ent oa_ent))(u_isn u_hdip))(u_isn u_hdip))	28	13
7659	(((u_hdip oa_wa)(u_csn (cp_ent ca_ent)))((((ca_wa p_re) cp_ent)((oa_ent u_isn) u_hdip)))(u_isn u_hdip))	23	13
7660	((((u_hdip (u_isn oa_wa))((u_csn cp_ent) p_csn)(p_isn ca_wa))((p_re ca_wa)(cp_ent oa_ent) u_hdip))	15	13
7661	((((u_hdip u_hdip)(oa_wa (u_csn cp_ent)))(ca_ent ca_wa))((p_re ca_wa))(cp_ent oa_ent)(oa_wa u_hdip)))	20	13
7662	((((u_hdip u_hdip)(oa_wa (u_csn cp_ent)))(ca_ent ca_wa))((p_re ca_wa))(cp_ent oa_ent))(oa_wa u_hdip)))	19	13
7663	((((u_hdip u_hdip)(oa_wa (u_csn cp_ent)))(ca_ent ca_wa))((p_re ca_wa))(cp_ent oa_ent))(u_hdip u_hdip)))	23	13
7664	((((u_hdip u_hdip)(oa_wa (u_csn cp_ent)) ca_ent)(((ca_wa p_re) ca_wa)(oa_ent u_isn))(u_hdip u_hdip)))	19	13
7665	((((u_hdip u_hdip)(oa_wa (u_csn cp_ent)) ca_ent)(((ca_wa p_re) cp_ent)(oa_ent u_hdip))(u_isn u_hdip)))	23	13
7666	((((u_hdip u_hdip)(oa_wa (u_csn cp_ent)) ca_ent)(((ca_wa p_re) cp_ent)(oa_ent u_isn))(u_hdip u_hdip)))	23	13
7667	((((u_hdip u_hdip)(oa_wa (u_csn cp_ent)))(p_csn (ca_ent ca_wa)))((p_re ca_wa))(cp_ent oa_ent) u_hdip)))	19	13
7668	((((u_hdip u_hdip)(oa_wa (u_csn cp_ent)))(p_csn ca_ent)))(((ca_wa p_re) cp_ent)((oa_ent u_isn) u_hdip)))	19	13
7669	((((u_hdip u_hdip)(oa_wa (u_csn cp_ent)))(p_csn ca_ent)))(((ca_wa p_re) ca_wa) oa_ent)(u_hdip u_hdip)))	16	13
7670	((((u_hdip u_hdip)(oa_wa (u_csn cp_ent)))(p_csn ca_ent)))(((ca_wa p_re) cp_ent) oa_ent)(u_isn u_hdip)))	19	13
7671	((((u_isn u_hdip)(u_isn oa_wa))((u_csn cp_ent) p_csn))(ca_ent ca_wa))((p_re ca_wa)(cp_ent oa_ent)))	19	13
7672	((((u_isn oa_wa)((u_csn cp_ent) p_csn))(p_isn ca_ent)))((ca_wa p_re) ca_wa)((cp_ent oa_ent) u_hdip)))	16	13
7673	((((u_isn oa_wa)((u_csn cp_ent) p_csn))(p_isn ca_ent)))((ca_wa p_re) cp_ent)((oa_ent u_isn) u_hdip)))	16	13
7674	((((u_isn oa_wa)((u_csn cp_ent) p_csn))(p_isn ca_ent)))(((ca_wa p_re) ca_wa) oa_ent)(u_hdip u_hdip)))	14	13
7675	((((u_isn oa_wa)((u_csn cp_ent) p_csn))(p_isn ca_ent)))(((ca_wa p_re) cp_ent) oa_ent)(u_isn u_hdip)))	16	13
7676	((((u_isn u_hdip)((u_isn oa_wa) u_csn))(cp_ent ca_ent)))(((ca_wa p_re) cp_ent)((oa_ent u_isn) u_hdip)))	21	13
7677	((((u_isn u_hdip)((u_isn oa_wa) u_csn))(cp_ent ca_ent)))(((ca_wa p_re) ca_wa) oa_ent)(u_hdip u_hdip)))	17	13
7678	((((u_isn u_hdip)((u_isn oa_wa) u_csn))(cp_ent ca_ent)))(((ca_wa p_re) cp_ent) oa_ent)(u_isn u_hdip)))	21	13
7679	(((oa_wa (u_csn cp_ent)) p_csn)(p_isn ca_ent))))((ca_wa p_re) ca_wa)(cp_ent oa_ent))(u_hdip u_hdip)))	20	13
7680	((((oa_wa (u_csn cp_ent))(p_isn ca_ent)))((p_re ca_wa) p_csn)(oa_ent (u_isn u_hdip))))(u_isn u_hdip))	18	13
7681	((((oa_wa (u_csn cp_ent))(p_isn ca_wa))((p_re ca_wa))(cp_ent oa_ent)(oa_wa u_hdip))))(u_isn u_hdip))	21	13
7682	((((p_csn (oa_ent u_hdip))(u_isn oa_wa))(u_csn cp_ent)))(p_csn (ca_ent ca_wa)))((p_re ca_wa) p_csn)))	23	13
7683	(((((u_csn cp_ent)(p_isn ca_ent))((ca_wa p_re) ca_wa))(cp_ent oa_ent))(u_isn u_hdip))(u_isn u_hdip))	27	13
7684	(((((u_csn (u_isn cp_ent))(ca_ent ca_wa))(p_re ca_wa)))(cp_ent oa_ent)(u_isn u_hdip))(u_isn u_hdip))	23	13
7685	((((u_hdip (oa_wa u_csn))(cp_ent ca_wa)))((p_re ca_wa)(cp_ent oa_ent))(u_hdip u_hdip)))(u_isn u_hdip))	17	13
7686	((((u_hdip (u_csn cp_ent))(p_csn ca_ent)))((ca_wa p_re) cp_ent)((oa_ent u_isn) u_hdip)))(u_isn u_hdip))	19	13
7687	((((u_hdip (u_isn oa_wa)) u_csn)(cp_ent ca_ent)))((ca_wa p_re) ca_wa))(p_csn oa_ent)(u_isn u_hdip)))	20	13
7688	((((u_hdip (u_isn oa_wa)) u_csn)(ca_ent ca_wa)))((p_re ca_wa))(cp_ent oa_ent) oa_wa))(u_isn u_hdip)))	20	13
7689	((((u_hdip (u_isn oa_wa)) u_csn)(ca_ent ca_wa))(((p_re ca_wa) p_csn)(p_isn oa_ent))(u_isn u_hdip)))	18	13
7690	((((u_hdip (u_isn oa_wa)) u_csn)(cp_ent ca_ent)))((ca_wa p_re) ca_wa)(p_csn oa_ent)))(u_hdip u_hdip))	19	13
7691	((((u_hdip (u_isn oa_wa)) u_csn)(cp_ent ca_ent)))((ca_wa p_re)(p_csn ((p_isn oa_ent)(u_isn u_hdip))))	17	13
7692	((((u_hdip (u_isn oa_wa)) u_csn)(cp_ent ca_ent)))((ca_wa p_re) ca_wa)(p_csn (p_isn oa_ent)) u_hdip))	17	13
7693	((((u_hdip (u_isn oa_wa)) u_csn)(ca_ent ca_wa)))((p_re ca_wa)(p_csn oa_ent) u_hdip))(u_isn u_hdip)))	18	13
7694	((((u_hdip (u_isn oa_wa)) u_csn)(cp_ent ca_wa)))((p_re ca_wa)(cp_ent oa_ent) oa_wa)))(u_hdip u_hdip))	19	13
7695	((((u_hdip (u_isn oa_wa)) u_csn)(ca_ent ca_wa)))((p_re ca_wa)(cp_ent oa_ent) u_hdip))(u_isn u_hdip)))	23	13
7696	((((u_hdip (u_isn oa_wa)) u_csn)(cp_ent ca_ent)))((ca_wa p_re) ca_wa)(oa_ent u_isn))(u_hdip u_hdip)))	21	13
7697	((((u_hdip (u_isn oa_wa)) u_csn)(cp_ent ca_ent)))((ca_wa p_re) cp_ent)(oa_ent u_hdip))(u_isn u_hdip)))	24	13
7698	((((u_hdip (u_isn oa_wa)) u_csn)(cp_ent ca_ent)))((ca_wa p_re) cp_ent)(oa_ent u_isn))(u_hdip u_hdip)))	24	13
7699	((((u_hdip (u_isn oa_wa)) u_csn)(ca_ent ca_wa)))(((p_re ca_wa) p_csn)(oa_ent u_isn))(u_hdip u_hdip)))	20	13
7700	((((u_hdip (u_isn oa_wa)) u_csn)(ca_ent ca_wa)))(((p_re ca_wa) p_csn)(oa_ent u_hdip))(u_isn u_hdip)))	19	13
7701	((((u_hdip (u_isn oa_wa)) u_csn)(cp_ent ca_wa)))((p_re ca_wa))(cp_ent oa_ent) oa_wa))(u_isn u_hdip)))	19	13
7702	((((u_hdip (u_isn oa_wa)) u_csn)(cp_ent ca_wa)))((p_re ca_wa))(cp_ent oa_ent) u_hdip))(u_isn u_hdip)))	19	13
7703	(((((u_csn cp_ent) p_csn)(ca_ent ca_wa)))((p_re ca_wa)(cp_ent oa_ent))(u_hdip u_hdip)))(u_isn u_hdip))	20	13
7704	(((((u_csn cp_ent)(ca_ent ca_wa))(p_re ca_wa)))(cp_ent oa_ent) u_hdip))(u_isn oa_wa))(u_isn oa_wa))	15	13
7705	(((((u_hdip oa_wa) u_csn)(cp_ent ca_ent)))(ca_wa p_re))(p_csn oa_ent)(u_isn u_hdip)))(u_isn u_hdip))	17	13
7706	(((((u_hdip oa_wa) u_csn)(cp_ent ca_ent)))(ca_wa p_re) cp_ent)((oa_ent u_isn) u_hdip)))(u_isn u_hdip))	24	13
7707	(((((u_isn cp_ent)(p_isn ca_ent)))(ca_wa p_re) ca_wa)))(cp_ent oa_ent)(u_isn u_hdip)))(u_isn u_hdip))	22	13
7708	(((((u_isn cp_ent)(p_isn ca_wa))(p_re ca_wa)))(cp_ent oa_ent)(u_isn u_hdip)))((u_isn oa_wa) u_hdip))	16	13
7709	(((((u_isn oa_wa) u_csn)(cp_ent ca_ent)))(ca_wa p_re) cp_ent)((oa_ent u_isn) u_hdip)))(u_isn u_hdip))	23	13
7710	((cp_ent ca_ent)(((((ca_wa p_re) ca_wa))(cp_ent oa_ent)(oa_wa u_hdip)))(u_isn u_hdip))(u_isn u_hdip)))	25	13
7711	((cp_ent ca_ent)(((((ca_wa p_re) ca_wa))(cp_ent oa_ent)(u_isn u_hdip)))(u_isn u_hdip))(u_isn oa_wa)))	24	13
7712	((cp_ent ca_ent)(((((ca_wa p_re) ca_wa))(p_csn oa_ent)(u_isn u_hdip)))(u_isn oa_wa))(u_isn oa_wa)))	21	13
7713	((cp_ent ca_ent)(((((ca_wa p_re) ca_wa))(p_csn oa_ent)(u_isn u_hdip)))(u_isn oa_wa))(u_isn u_hdip)))	20	13
7714	((cp_ent ca_ent)((((((ca_wa p_re) ca_wa)(cp_ent oa_ent))(u_hdip u_hdip))(u_isn u_hdip))(u_isn oa_wa)))	28	13
7715	((cp_ent ca_ent)((((((ca_wa p_re) ca_wa)(cp_ent oa_ent))(u_hdip u_hdip))(u_isn u_hdip))(u_isn u_hdip)))	26	13
7716	((cp_ent ca_ent)((((((ca_wa p_re) ca_wa)(p_csn oa_ent))(u_hdip u_hdip))(u_isn u_hdip))(u_isn u_hdip)))	19	13
7717	((cp_ent ca_ent)((((((ca_wa p_re) ca_wa)(p_csn oa_ent))(u_isn u_hdip))(u_isn oa_wa))(u_isn u_hdip)))	23	13
7718	((oa_wa u_hdip)(((u_csn cp_ent)(p_csn (p_isn ca_ent))))((((ca_wa p_re) cp_ent) oa_ent)(u_isn u_hdip)))	16	13
7719	((p_isn ca_ent)(((((ca_wa p_re) ca_wa))(cp_ent oa_ent)(oa_wa u_hdip)))(u_isn u_hdip))(u_isn u_hdip)))	20	13
7720	((p_isn ca_ent)(((((ca_wa p_re) ca_wa))(cp_ent oa_ent)(u_isn u_hdip)))(u_isn u_hdip))(u_isn oa_wa)))	20	13
7721	((p_isn ca_ent)(((((ca_wa p_re) ca_wa))(p_csn oa_ent)(u_isn u_hdip)))(u_isn oa_wa))(u_isn oa_wa)))	18	13
7722	((p_isn ca_ent)(((((ca_wa p_re) ca_wa))(p_csn oa_ent)(u_isn u_hdip)))(u_isn oa_wa))(u_isn u_hdip)))	18	13
7723	(((p_isn ca_wa)(p_isn p_re))(((ca_wa p_csn) oa_ent)(u_isn u_hdip))(u_isn u_hdip))))(u_isn u_hdip))	26	13
7724	((u_csn (cp_ent ca_ent))((((ca_wa p_re) ca_wa)((p_csn ((p_isn oa_ent)(u_isn u_hdip)))(u_isn u_hdip)))	18	13
7725	((u_csn (cp_ent ca_ent))(((((ca_wa p_re) ca_wa)(p_csn (p_isn oa_ent)))(u_isn u_hdip))(u_isn u_hdip)))	20	13
7726	((u_csn (cp_ent ca_ent))(((((ca_wa p_re) ca_wa)(p_csn (oa_ent u_isn)))(u_hdip u_hdip))(u_isn u_hdip)))	21	13
7727	((u_csn (cp_ent ca_ent))(((((ca_wa p_re) ca_wa)(cp_ent oa_ent) u_hdip))(u_isn u_hdip))(u_isn oa_wa)))	21	13
7728	((u_csn cp_ent)(ca_ent ca_wa))(((((p_re ca_wa)(cp_ent oa_ent) u_hdip))(u_isn u_hdip))(u_isn u_hdip)))	26	13
7729	((u_csn (cp_ent ca_ent))(((((ca_wa p_re) ca_wa)(cp_ent oa_ent) u_hdip))(u_isn u_hdip))(u_isn u_hdip)))	27	13

7730	((u_csn (cp_ent ca_ent))((((ca_wa p_re) ca_wa)((p_csn oa_ent) u_hdip))(u_isn u_hdip))(u_isn u_hdip)))	21	13
7731	((u_csn (cp_ent ca_ent))(((((ca_wa p_re) ca_wa)((cp_ent oa_ent) oa_wa))(u_hdip u_hdip))(u_isn u_hdip)))	20	13
7732	((u_csn (cp_ent ca_ent))((((ca_wa p_re) cp_ent)((oa_ent u_isn) u_hdip))(u_isn oa_wa))(u_isn oa_wa)))	21	13
7733	((u_csn (cp_ent ca_ent))((((ca_wa p_re) cp_ent)((oa_ent u_isn) u_hdip))(u_isn oa_wa))(u_isn u_hdip)))	22	13
7734	((u_csn (cp_ent ca_ent))((((ca_wa p_re) cp_ent)((oa_ent u_isn) u_hdip))(u_isn u_hdip))(u_isn u_hdip)))	24	13
7735	(((u_csn cp_ent)(ca_ent ca_wa))((((p_re ca_wa) p_csn)(oa_ent u_isn))(u_hdip u_hdip))(u_isn u_hdip)))	22	13
7736	((u_hdip oa_wa)((((u_csn cp_ent) p_csn)(((ca_wa p_re) cp_ent))(oa_ent u_isn) u_hdip)))(u_isn u_hdip)))	18	13
7737	((u_hdip u_hdip)((u_isn oa_wa)(((u_csn cp_ent)(p_csn ca_ent))(((ca_wa p_re) ca_wa)(oa_ent u_isn)))))	17	13
7738	((u_hdip u_hdip)((((u_isn oa_wa) u_csn)(cp_ent ca_ent))))((ca_wa p_re) cp_ent) oa_ent)(u_isn oa_wa)))	18	13
7739	((u_hdip u_hdip))(((u_isn oa_wa) u_csn)(cp_ent ca_ent))))((ca_wa p_re) ca_wa) oa_ent)(u_hdip u_hdip))))	19	13
7740	((u_hdip u_hdip)((((u_isn oa_wa)((u_csn cp_ent) p_csn)) ca_ent)(((ca_wa p_re) ca_wa)(oa_ent u_isn))))	18	13
7741	((u_hdip u_hdip))(((u_isn oa_wa)((u_csn cp_ent) p_csn)) ca_ent))(((ca_wa p_re) cp_ent)(oa_ent u_isn))))	19	13
7742	((u_hdip u_hdip))(((u_isn oa_wa) u_csn)(cp_ent ca_wa)))((p_re ca_wa)(cp_ent oa_ent)))(oa_wa u_hdip)))	19	13
7743	((u_isn u_hdip))(((u_isn oa_wa)((u_csn cp_ent) p_csn)) ca_ent)(((ca_wa p_re) ca_wa)(oa_ent u_isn))))	17	13
7744	((u_isn u_hdip))(((u_isn oa_wa)((u_csn cp_ent) p_csn)) ca_ent)(((ca_wa p_re) cp_ent)(oa_ent u_isn))))	18	13
7745	((oa_wa (u_csn (cp_ent ca_ent)))((((ca_wa p_re) ca_wa)(cp_ent oa_ent))(u_isn oa_wa))(u_isn oa_wa)))	24	13
7746	((oa_wa (u_csn (cp_ent ca_ent)))((((ca_wa p_re) ca_wa)(cp_ent oa_ent))(u_isn oa_wa))(u_isn u_hdip)))	22	13
7747	((oa_wa (u_csn (cp_ent ca_ent)))((((ca_wa p_re) ca_wa)(cp_ent oa_ent))(u_isn u_hdip))(u_isn oa_wa)))	25	13
7748	((oa_wa (u_csn (cp_ent ca_ent)))((((ca_wa p_re) cp_ent)(oa_ent u_hdip))(u_isn oa_wa))(u_isn oa_wa)))	19	13
7749	((oa_wa (u_csn (cp_ent ca_ent)))((((ca_wa p_re) cp_ent)(oa_ent u_hdip))(u_isn oa_wa))(u_isn u_hdip)))	20	13
7750	((p_csn (ca_ent ca_wa))((((p_re ca_wa)(cp_ent oa_ent))(u_isn u_hdip))(u_isn u_hdip))(u_isn oa_wa)))	22	13
7751	((u_csn (ca_ent ca_wa))((((p_re ca_wa)(cp_ent oa_ent))(u_isn u_hdip))(u_isn u_hdip))(u_isn oa_wa)))	20	13
7752	((u_csn (cp_ent ca_wa))((((p_re ca_wa)((cp_ent oa_ent)(oa_wa u_hdip)))(u_isn u_hdip))(u_isn u_hdip)))	18	13
7753	((u_csn (cp_ent ca_wa))((((p_re ca_wa)(cp_ent oa_ent))(oa_wa u_hdip))(u_isn u_hdip))(u_isn u_hdip)))	18	13
7754	((u_csn (cp_ent ca_wa))((((p_re ca_wa)(cp_ent oa_ent))(u_hdip u_hdip))(u_isn u_hdip))(u_isn oa_wa)))	21	13
7755	((u_csn (cp_ent ca_wa))((((p_re ca_wa)(cp_ent oa_ent))(u_hdip u_hdip))(u_isn u_hdip))(u_isn u_hdip)))	20	13
7756	((u_csn (cp_ent ca_wa))((((p_re ca_wa)(cp_ent oa_ent))(u_isn u_hdip))(u_isn u_hdip))(u_isn oa_wa)))	19	13
7757	(((u_csn cp_ent)(p_csn ca_ent))((((ca_wa p_re) ca_wa)(oa_ent u_isn))(u_hdip u_hdip))(u_isn u_hdip)))	18	13
7758	((((u_csn cp_ent) p_csn) ca_ent)((((ca_wa p_re) ca_wa)(cp_ent oa_ent))(u_hdip u_hdip)))(u_isn u_hdip))	22	13
7759	(((u_csn cp_ent)(p_csn ca_ent))((((ca_wa p_re) cp_ent)(oa_ent u_hdip))(u_isn u_hdip))(u_isn u_hdip)))	22	13
7760	((((u_csn cp_ent) p_csn) ca_ent)((((ca_wa p_re) cp_ent)(oa_ent u_isn))(u_hdip u_hdip))(u_isn u_hdip)))	19	13
7761	((u_csn (p_isn ca_ent))(((ca_wa p_re) ca_wa)((p_csn ((p_isn oa_ent)(u_isn u_hdip)))(u_isn u_hdip))))	16	13
7762	(((u_csn cp_ent)(p_isn ca_ent))(((ca_wa p_re)(p_csn ((p_isn oa_ent)(u_isn u_hdip))))(u_isn u_hdip)))	16	13
7763	((u_csn (p_isn ca_ent))((((ca_wa p_re) cp_ent)((oa_ent u_isn) u_hdip))(u_isn oa_wa))(u_isn oa_wa)))	18	13
7764	((u_csn (p_isn ca_ent))((((ca_wa p_re) cp_ent)((oa_ent u_isn) u_hdip))(u_isn oa_wa))(u_isn u_hdip)))	19	13
7765	((u_csn (p_isn ca_ent))((((ca_wa p_re) cp_ent)((oa_ent u_isn) u_hdip))(u_isn u_hdip))(u_isn u_hdip)))	20	13
7766	((u_csn (u_isn cp_ent))((p_isn ca_ent))(((ca_wa p_re) ca_wa)(cp_ent oa_ent)))((u_isn oa_wa) u_hdip)))	19	13
7767	(((oa_wa (u_csn cp_ent)) ca_ent))(((ca_wa p_re)(p_csn ((p_isn oa_ent)(u_isn u_hdip))))(u_isn u_hdip)))	17	13
7768	(((oa_wa (u_csn cp_ent)) ca_ent))(((ca_wa p_re) ca_wa)(p_csn ((p_isn oa_ent) u_hdip)))(u_isn u_hdip)))	17	13
7769	(((oa_wa (u_csn cp_ent)) ca_ent))((((ca_wa p_re) ca_wa)(cp_ent oa_ent))(u_isn u_hdip))(u_isn oa_wa)))	26	13
7770	(((oa_wa (u_csn cp_ent)) ca_ent))((((ca_wa p_re) ca_wa)(oa_ent u_isn))(u_hdip u_hdip))(u_isn u_hdip)))	20	13
7771	(((oa_wa (u_csn cp_ent)) ca_ent))((((ca_wa p_re) cp_ent)(oa_ent u_hdip))(u_isn u_hdip))(u_isn u_hdip)))	26	13
7772	(((oa_wa (u_csn cp_ent)) ca_ent))((((ca_wa p_re) cp_ent)(oa_ent u_isn))(u_hdip u_hdip))(u_isn u_hdip)))	22	13
7773	(((oa_wa (u_csn cp_ent))(ca_ent ca_wa))((((p_re ca_wa) p_csn) oa_ent)(u_hdip u_hdip))(u_isn u_hdip)))	21	13
7774	(((oa_wa (u_csn cp_ent))(ca_ent ca_wa))((((p_re ca_wa) p_csn) oa_ent)(u_isn u_hdip))(u_isn u_hdip)))	24	13
7775	(((oa_wa u_csn)(cp_ent ca_wa))((((p_re ca_wa)((cp_ent oa_ent) oa_wa))(u_isn u_hdip))(u_isn u_hdip)))	22	13
7776	(((oa_wa u_csn)(cp_ent ca_wa))((((p_re ca_wa)((cp_ent oa_ent) u_hdip))(u_isn u_hdip))(u_isn oa_wa)))	19	13
7777	(((oa_wa u_csn)(cp_ent ca_wa))((((p_re ca_wa)((cp_ent oa_ent) u_hdip))(u_isn u_hdip))(u_isn u_hdip)))	23	13
7778	(((oa_wa (u_csn cp_ent))(p_csn ca_ent))((((ca_wa p_re) ca_wa) oa_ent)(u_hdip u_hdip))(u_isn u_hdip)))	17	13
7779	(((oa_wa (u_csn cp_ent))(p_csn ca_ent))((((ca_wa p_re) cp_ent) oa_ent)(u_isn u_hdip))(u_isn u_hdip)))	23	13
7780	(((oa_wa u_csn)(p_isn ca_ent))((((ca_wa p_re) ca_wa)(cp_ent oa_ent))(u_isn u_hdip))(u_isn oa_wa)))	19	13
7781	(((oa_wa u_csn)(p_isn ca_ent))((((ca_wa p_re) cp_ent)(oa_ent u_hdip))(u_isn oa_wa))(u_isn u_hdip)))	17	13
7782	(((u_csn cp_ent)(p_isn ca_ent))((((ca_wa p_re) ca_wa)(cp_ent oa_ent))(u_isn oa_wa))(u_isn oa_wa)))	21	13
7783	(((u_csn cp_ent)(p_isn ca_ent))((((ca_wa p_re) ca_wa)(cp_ent oa_ent))(u_isn oa_wa))(u_isn u_hdip)))	21	13
7784	(((u_csn cp_ent)(p_isn ca_ent))((((ca_wa p_re) ca_wa)(cp_ent oa_ent))(u_isn u_hdip))(u_isn oa_wa)))	23	13
7785	(((u_csn cp_ent)(p_isn ca_ent))((((ca_wa p_re) ca_wa)(oa_ent u_isn))(u_hdip u_hdip))(u_isn u_hdip)))	20	13
7786	(((u_csn cp_ent)(p_isn ca_ent))((((ca_wa p_re) cp_ent)(oa_ent u_hdip))(u_isn oa_wa))(u_isn oa_wa)))	19	13
7787	(((u_csn cp_ent)(p_isn ca_ent))((((ca_wa p_re) cp_ent)(oa_ent u_hdip))(u_isn oa_wa))(u_isn u_hdip)))	20	13
7788	(((u_csn cp_ent)(p_isn ca_wa))((((p_re ca_wa)((cp_ent oa_ent) oa_wa))(u_hdip u_hdip))(u_isn u_hdip)))	17	13
7789	(((u_csn cp_ent)(p_isn ca_wa))((((p_re ca_wa)((cp_ent oa_ent) u_hdip))(u_isn u_hdip))(u_isn oa_wa)))	19	13
7790	(((u_csn cp_ent)(p_isn ca_wa))((((p_re ca_wa)((cp_ent oa_ent) u_hdip))(u_isn u_hdip))(u_isn u_hdip)))	22	13
7791	(((u_hdip u_csn)(cp_ent ca_ent))((((ca_wa p_re) ca_wa)(oa_ent u_isn))(u_hdip u_hdip))(u_isn u_hdip)))	18	13
7792	(((u_hdip u_csn)(cp_ent ca_ent))((((ca_wa p_re) cp_ent)(oa_ent u_hdip))(u_isn u_hdip))(u_isn u_hdip)))	23	13
7793	(((u_hdip u_csn)(cp_ent ca_ent))((((ca_wa p_re) cp_ent)(oa_ent u_isn))(u_hdip u_hdip))(u_isn u_hdip)))	21	13
7794	(((u_hdip u_csn)(cp_ent ca_wa))((((p_re ca_wa)((cp_ent oa_ent) u_hdip))(u_isn u_hdip))(u_isn u_hdip)))	20	13
7795	(((u_isn cp_ent)(p_isn ca_ent))((((ca_wa p_re) ca_wa)(cp_ent oa_ent))(u_isn oa_wa))(u_isn u_hdip)))	18	13
7796	(((u_isn cp_ent)(p_isn ca_ent))((((ca_wa p_re) ca_wa)(cp_ent oa_ent))(u_isn u_hdip))(u_isn u_hdip)))	20	13
7797	(((u_isn oa_wa) u_hdip)((((u_csn cp_ent) p_csn) ca_ent))(((ca_wa p_re) cp_ent) oa_ent)(u_isn u_hdip))))	19	13
7798	(((u_isn u_csn)(cp_ent ca_ent))((((ca_wa p_re) cp_ent)(oa_ent u_hdip))(u_isn u_hdip))(u_isn u_hdip)))	19	13
7799	(((u_isn u_csn)(cp_ent ca_ent))((((ca_wa p_re) cp_ent)(oa_ent u_isn))(u_hdip u_hdip))(u_isn u_hdip)))	18	13
7800	(((u_isn u_csn)(cp_ent ca_wa))((((p_re ca_wa)((cp_ent oa_ent) oa_wa))(u_hdip u_hdip))(u_isn u_hdip)))	15	13
7801	(((oa_wa u_csn)(p_csn (p_isn ca_ent)))((((ca_wa p_re) cp_ent) oa_ent)(u_isn u_hdip))(u_isn u_hdip)))	17	13
7802	(((u_csn cp_ent)(p_csn (p_isn ca_ent)))((((ca_wa p_re) cp_ent) oa_ent)(u_isn u_hdip))(u_isn u_hdip)))	20	13
7803	(((u_hdip oa_wa)(u_csn (cp_ent ca_ent)))((((ca_wa p_re) ca_wa) oa_ent)(u_hdip u_hdip))(u_isn u_hdip)))	19	13
7804	(((u_hdip oa_wa)(u_csn (cp_ent ca_ent)))((((ca_wa p_re) cp_ent) oa_ent)(u_isn u_hdip))(u_isn u_hdip)))	23	13
7805	(((oa_wa (u_csn cp_ent))(p_isn ca_ent))(((ca_wa p_re)((p_csn (p_isn oa_ent)) u_hdip))(u_isn u_hdip)))	15	13
7806	(((oa_wa (u_csn cp_ent))(p_isn ca_ent))((((ca_wa p_re) ca_wa) oa_ent)(u_hdip u_hdip))(u_isn u_hdip)))	18	13
7807	(((oa_wa (u_csn cp_ent))(p_isn ca_ent))((((ca_wa p_re) ca_wa) oa_ent)(u_isn u_hdip))(u_isn u_hdip)))	19	13
7808	(((oa_wa (u_csn cp_ent))(p_isn ca_ent))((((ca_wa p_re) cp_ent) oa_ent)(u_isn u_hdip))(u_isn oa_wa)))	20	13
7809	(((oa_wa (u_csn cp_ent))(p_isn ca_ent))((((ca_wa p_re) cp_ent) oa_ent)(u_isn u_hdip))(u_isn u_hdip)))	24	13
7810	(((u_csn (u_isn cp_ent)) ca_ent))((((ca_wa p_re) ca_wa)(cp_ent oa_ent))(u_isn oa_wa))(u_isn oa_wa)))	17	13
7811	(((u_csn (u_isn cp_ent)) ca_ent))((((ca_wa p_re) ca_wa)(cp_ent oa_ent))(u_isn oa_wa))(u_isn u_hdip)))	18	13
7812	(((u_csn (u_isn cp_ent))(p_isn ca_ent))((((ca_wa p_re) cp_ent) oa_ent)(u_isn u_hdip))(u_isn u_hdip)))	20	13
7813	(((u_hdip (oa_wa u_csn))(cp_ent ca_ent))((((ca_wa p_re) cp_ent) oa_ent)(u_isn u_hdip))(u_isn oa_wa)))	18	13
7814	(((u_hdip (u_csn cp_ent)) ca_ent))((((ca_wa p_re) cp_ent)(oa_ent u_isn))(u_hdip u_hdip))(u_isn u_hdip)))	22	13
7815	(((u_hdip (u_csn cp_ent))(p_csn ca_ent))((((ca_wa p_re) ca_wa) oa_ent)(u_hdip u_hdip))(u_isn u_hdip)))	16	13
7816	(((u_hdip (u_csn cp_ent))(p_csn ca_ent))((((ca_wa p_re) cp_ent) oa_ent)(u_isn u_hdip))(u_isn u_hdip)))	19	13
7817	(((u_hdip (u_csn cp_ent))(p_isn ca_ent))((((ca_wa p_re) ca_wa) oa_ent)(u_hdip u_hdip))(u_isn u_hdip)))	17	13
7818	(((u_hdip (u_csn cp_ent))(p_isn ca_ent))((((ca_wa p_re) cp_ent) oa_ent)(u_isn u_hdip))(u_isn u_hdip)))	21	13
7819	((((oa_wa u_csn) p_csn)(p_isn ca_ent))((((ca_wa p_re) cp_ent) oa_ent)(u_isn u_hdip))(u_isn u_hdip)))	19	13
7820	((((u_csn cp_ent) p_csn) ca_ent)((((ca_wa p_re) cp_ent)(oa_ent u_hdip))(u_isn u_hdip))(u_isn u_hdip)))	21	13
7821	((((u_csn cp_ent) p_csn)(p_isn ca_ent))((((ca_wa p_re) cp_ent) oa_ent)(u_isn u_hdip))(u_isn u_hdip)))	22	13
7822	((((u_hdip oa_wa) u_csn)(cp_ent ca_ent))(((ca_wa p_re)((p_csn (p_isn oa_ent)) u_hdip))(u_isn u_hdip)))	16	13
7823	((((u_hdip oa_wa) u_csn)(cp_ent ca_ent))((((ca_wa p_re) ca_wa) oa_ent)(u_hdip u_hdip))(u_isn u_hdip)))	20	13
7824	((((u_hdip oa_wa) u_csn)(cp_ent ca_ent))((((ca_wa p_re) ca_wa) oa_ent)(u_isn u_hdip))(u_isn u_hdip)))	21	13
7825	((((u_hdip oa_wa) u_csn)(cp_ent ca_ent))((((ca_wa p_re) cp_ent) oa_ent)(u_isn u_hdip))(u_isn u_hdip)))	24	13
7826	((((u_hdip oa_wa) u_csn)(ca_ent ca_wa))((((p_re ca_wa) p_csn) oa_ent)(u_hdip u_hdip))(u_isn u_hdip)))	19	13
7827	((((u_isn oa_wa) u_csn)(cp_ent ca_ent))((((ca_wa p_re) ca_wa) oa_ent)(u_hdip u_hdip))(u_isn u_hdip)))	19	13
7828	((((u_isn oa_wa) u_csn)(cp_ent ca_ent))((((ca_wa p_re) cp_ent) oa_ent)(u_isn u_hdip))(u_isn u_hdip)))	23	13
7829	((((oa_wa (u_csn cp_ent)) p_csn) ca_ent)((((ca_wa p_re) cp_ent) oa_ent)(u_isn u_hdip))(u_isn u_hdip)))	22	13
7830	((((cp_ent ca_ent))((ca_wa p_re) ca_wa)((cp_ent oa_ent) oa_wa)))(u_isn oa_wa))(u_hdip (u_isn u_csn)))	19	13
7831	(((cp_ent ca_ent))(((ca_wa p_re) ca_wa)((cp_ent oa_ent)(oa_wa u_hdip)))(u_isn u_hdip)))(u_isn oa_wa))	25	13
7832	(((cp_ent ca_ent))(((ca_wa p_re) ca_wa)(cp_ent oa_ent))(u_isn u_hdip)))((oa_wa u_hdip)(u_isn oa_wa)))	27	13
7833	(((cp_ent ca_ent))(((ca_wa p_re) ca_wa)((cp_ent oa_ent) oa_wa))(u_isn oa_wa)))(u_hdip (u_isn u_csn)))	18	13
7834	(((oa_ent oa_wa)((u_isn u_hdip)((u_isn u_hdip)((u_isn oa_wa) u_csn))))(cp_ent oa_ent)(oa_wa u_hdip)))	15	13
7835	(((oa_ent oa_wa)((u_isn u_hdip)((u_isn u_hdip)((u_isn oa_wa) u_csn))))(cp_ent oa_ent)(u_isn u_hdip)))	15	13
7836	((((p_isn ca_ent))(((ca_wa p_re) ca_wa)(cp_ent oa_ent)))(oa_wa u_hdip))(u_isn u_hdip))(u_isn u_hdip))	20	13
7837	(((p_isn ca_ent))(((ca_wa p_re) ca_wa)((cp_ent oa_ent)(oa_wa u_hdip)))(u_isn u_hdip)))(u_isn oa_wa))	23	13
7838	(((p_isn ca_ent))(((ca_wa p_re) ca_wa)((cp_ent oa_ent)(oa_wa u_hdip)))(u_isn u_hdip)))(u_isn u_hdip))	23	13
7839	(((u_csn (cp_ent ca_ent))((((ca_wa p_re) cp_ent)((oa_ent u_isn) u_hdip))(u_isn u_hdip)))(u_isn u_hdip))	25	13
7840	(((u_csn (cp_ent ca_ent))((((ca_wa p_re) cp_ent)(oa_ent (u_isn u_hdip)))(u_isn u_hdip)))(u_isn u_hdip))	26	13
7841	(((u_hdip u_hdip)((u_isn oa_wa)((u_csn cp_ent) p_csn)))((ca_ent ca_wa)((p_re ca_wa)(cp_ent oa_ent)))	20	13
7842	(((u_hdip u_hdip)((u_isn oa_wa)((u_csn cp_ent) p_csn)) ca_ent))(((ca_wa p_re) ca_wa)(oa_ent u_isn)))	19	13
7843	(((u_hdip u_hdip)((u_isn oa_wa)((u_csn cp_ent) p_csn)) ca_ent))(((ca_wa p_re) cp_ent)(oa_ent u_isn)))	20	13
7844	(((u_hdip u_hdip)(((oa_wa (u_csn cp_ent)) p_csn) ca_ent))(((ca_wa p_re) cp_ent)((oa_ent u_isn) u_hdip)))	20	13
7845	(((u_hdip u_hdip)(((oa_wa (u_csn cp_ent)) p_csn)(p_isn ca_ent)))(((ca_wa p_re) ca_wa)(oa_ent u_isn)))	15	13
7846	(((u_hdip u_hdip)(((oa_wa (u_csn cp_ent)) p_csn)(p_isn ca_ent)))(((ca_wa p_re) cp_ent)(oa_ent u_isn)))	16	13
7847	(((oa_wa (u_csn (cp_ent ca_ent)))((((ca_wa p_re) ca_wa)(cp_ent oa_ent))(u_isn oa_wa)))(u_isn u_hdip))	23	13
7848	(((oa_wa (u_csn (cp_ent ca_ent)))((((ca_wa p_re) cp_ent)(oa_ent u_hdip))(u_isn oa_wa)))(u_isn u_hdip))	21	13
7849	(((u_csn (cp_ent ca_wa))((((p_re ca_wa)(cp_ent oa_ent))(u_hdip u_hdip))(u_isn u_hdip)))(u_isn oa_wa))	22	13
7850	((((u_csn (p_isn ca_ent))(((ca_wa p_re) cp_ent)((oa_ent u_isn) u_hdip)))(u_isn u_hdip)))(u_isn u_hdip))	22	13
7851	((((oa_wa (u_csn cp_ent)) ca_ent))(((ca_wa p_re) cp_ent)(oa_ent u_isn))(u_hdip u_hdip)))(u_isn u_hdip))	25	13
7852	((((oa_wa (u_csn cp_ent)) ca_ent))(((ca_wa p_re) cp_ent)(oa_ent oa_wa))(u_isn u_hdip)))(u_isn u_hdip))	20	13
7853	((((oa_wa (u_csn cp_ent))(ca_ent ca_wa))(((p_re ca_wa) p_csn) oa_ent)(u_hdip u_hdip)))(u_isn u_hdip))	22	13
7854	((((oa_wa (u_csn cp_ent))(ca_ent ca_wa))(((p_re ca_wa) p_csn) oa_ent)(u_isn u_hdip)))(u_isn u_hdip))	25	13
7855	((((oa_wa u_csn)(cp_ent ca_wa))(((p_re ca_wa)((cp_ent oa_ent) oa_wa))(u_hdip u_hdip)))(u_isn oa_wa))	20	13
7856	((((oa_wa u_csn)(cp_ent ca_wa))(((p_re ca_wa)((cp_ent oa_ent) oa_wa))(u_hdip u_hdip)))(u_isn u_hdip))	21	13
7857	((((oa_wa u_csn)(cp_ent ca_wa))(((p_re ca_wa)((cp_ent oa_ent) oa_wa))(u_isn u_hdip)))(u_isn oa_wa))	21	13
7858	((((oa_wa u_csn)(cp_ent ca_wa))(((p_re ca_wa)((cp_ent oa_ent) u_hdip))(u_isn u_hdip)))(u_isn u_hdip))	22	13
7859	((((u_csn cp_ent)(ca_ent ca_wa))(((p_re ca_wa)((cp_ent oa_ent) u_hdip))(u_isn u_hdip)))(u_isn u_hdip))	24	13
7860	((((u_hdip u_csn)(cp_ent ca_ent))(((ca_wa p_re) cp_ent)(oa_ent u_hdip))(u_isn u_hdip)))(u_isn u_hdip))	22	13
7861	((((u_isn oa_wa) u_hdip)(((u_isn oa_wa) u_csn)(cp_ent ca_ent)))((ca_wa p_re) cp_ent)))(oa_wa u_hdip))	17	13
7862	((((u_isn u_csn)(cp_ent ca_ent))(((ca_wa p_re) ca_wa)(cp_ent oa_ent))(u_isn oa_wa)))(u_isn u_hdip))	17	13
7863	((((u_isn u_csn)(cp_ent ca_ent))(((ca_wa p_re) cp_ent)(oa_ent u_isn))(u_hdip u_hdip)))(u_isn u_hdip))	16	13
7864	((((cp_ent ca_ent))(((ca_wa p_re) ca_wa)((cp_ent oa_ent)(oa_wa u_hdip))))(u_isn u_hdip))(u_isn u_hdip))	26	13
7865	((((cp_ent ca_ent))(((ca_wa p_re) ca_wa)(cp_ent oa_ent)))((oa_wa u_hdip)(u_isn oa_wa)))(u_csn cp_ent))	25	13
7866	((((cp_ent ca_ent))(((ca_wa p_re) ca_wa)((cp_ent oa_ent)(oa_wa u_hdip))))(u_isn u_hdip))(u_isn oa_wa))	26	13
7867	((((cp_ent ca_ent))(((ca_wa p_re) ca_wa)((p_csn oa_ent)(u_isn u_hdip))))(u_isn oa_wa))(u_isn oa_wa))	24	13
7868	((((cp_ent ca_ent))(((ca_wa p_re) ca_wa)((p_csn oa_ent)(u_isn u_hdip))))(u_isn oa_wa))(u_isn u_hdip))	22	13
7869	((((p_isn ca_ent))(((ca_wa p_re) ca_wa)((cp_ent oa_ent)(oa_wa u_hdip))))(u_isn u_hdip))(u_isn oa_wa))	24	13
7870	((((p_isn ca_ent))(((ca_wa p_re) ca_wa)((cp_ent oa_ent)(oa_wa u_hdip))))(u_isn u_hdip))(u_isn u_hdip))	24	13
7871	((((p_isn ca_ent))(((ca_wa p_re) ca_wa) oa_ent)))(oa_wa u_hdip)(u_isn oa_wa)))((u_csn cp_ent) oa_ent))	19	13
7872	((((p_isn ca_ent))(((ca_wa p_re) ca_wa)(cp_ent oa_ent)))(u_isn u_hdip))((oa_wa u_hdip)(u_isn oa_wa)))	25	13
7873	((((p_isn ca_ent))(((ca_wa p_re) cp_ent) oa_ent))((oa_wa u_hdip)(u_isn oa_wa)))((u_csn cp_ent) oa_ent))	18	13
7874	((((p_isn ca_ent))(((ca_wa p_re) cp_ent)(oa_ent (u_isn u_hdip))))(u_isn oa_wa))((u_isn oa_wa) u_hdip))	22	13
7875	((((p_isn ca_ent))((p_re ca_wa)(cp_ent oa_ent))(oa_wa u_hdip))((u_isn oa_wa))((u_csn cp_ent) oa_ent))	20	13
7876	((((u_csn (cp_ent ca_ent))(((ca_wa p_re) cp_ent)((oa_ent u_isn) u_hdip)))(u_isn u_hdip))(u_isn u_hdip))	26	13
7877	((((u_isn u_hdip)((u_isn oa_wa)((u_csn cp_ent) p_csn))) ca_ent))(((ca_wa p_re) ca_wa)(oa_ent u_isn)))	17	13
7878	((((u_isn u_hdip)((u_isn oa_wa)((u_csn cp_ent) p_csn))) ca_ent))(((ca_wa p_re) cp_ent)(oa_ent u_isn)))	19	13
7879	((((u_hdip (u_csn cp_ent))((ca_ent ca_wa)((p_isn ca_wa) p_csn)))((oa_ent u_isn) u_hdip))(u_isn u_hdip))	18	13
7880	((((p_csn (oa_ent (u_isn u_hdip)))(u_isn oa_wa)))(((u_csn cp_ent) p_csn) ca_ent))((ca_wa p_re) ca_wa))	25	13
7881	((((p_csn ((oa_ent u_isn) u_hdip))(u_isn oa_wa))(u_csn cp_ent)))(p_csn (ca_ent ca_wa))(p_isn p_re)))	23	13
7882	(((((ca_ent ca_wa)(p_re ca_wa)(cp_ent oa_ent)))(u_isn oa_wa))(u_hdip (u_isn u_csn)))(u_isn cp_ent))	19	13
7883	(((((p_isn ca_ent)(p_re ca_wa)(cp_ent oa_ent)))(u_isn u_hdip))(u_isn oa_wa))(u_hdip (u_isn u_csn)))	19	13
7884	(((((ca_wa p_re) ca_wa)((cp_ent oa_ent) oa_wa))(u_isn oa_wa))(u_hdip (u_isn u_csn)))(u_isn cp_ent))	17	13
7885	(((((ca_wa p_re) ca_wa)((cp_ent oa_ent)(oa_wa u_hdip)))(u_isn u_hdip))(u_isn u_hdip))(u_isn oa_wa))	27	13
7886	((((u_csn (cp_ent ca_ent))((ca_wa p_re) ca_wa))((oa_ent u_isn) u_hdip))(u_isn u_hdip))(u_isn u_hdip))	21	13
7887	((((u_csn cp_ent)((ca_ent ca_wa) p_re))((p_isn ca_wa)))(cp_ent oa_ent)(u_isn u_hdip)))(u_isn u_hdip))	24	13
7888	(((((u_csn cp_ent)(ca_ent ca_wa))(p_isn p_re))((cp_ent oa_ent) u_hdip))(u_isn u_hdip))(u_isn u_hdip))	25	13
7889	((((u_csn cp_ent)((p_isn ca_ent)(ca_wa p_re)))(p_isn ca_wa)))(cp_ent oa_ent) u_hdip))(u_isn u_hdip))	22	13
7890	((((oa_wa (u_csn cp_ent))(p_isn ca_wa))((p_re ca_wa)(cp_ent oa_ent)))(oa_wa u_hdip))(u_isn u_hdip))	22	13
7891	(((((u_csn cp_ent)(p_isn ca_wa))((p_isn ca_wa))((oa_ent u_isn) u_hdip))(u_isn u_hdip))(u_isn u_hdip))	22	13
7892	(((((u_csn cp_ent)(p_isn ca_ent))((ca_wa p_re) ca_wa))(cp_ent oa_ent))(u_hdip u_hdip))(u_isn u_hdip))	18	13
7893	((((u_csn (p_isn ca_ent))(ca_wa p_re))(p_isn ca_wa)))(cp_ent oa_ent)(u_isn u_hdip)))(u_isn u_hdip))	21	13
7894	((((u_csn (u_isn cp_ent))(ca_wa p_re))(ca_wa p_csn)))(cp_ent oa_ent)(u_isn u_hdip)))(u_isn u_hdip))	19	13
7895	((((u_csn (u_isn cp_ent))(p_isn ca_wa))(p_re ca_wa)))(cp_ent oa_ent)(u_isn u_hdip)))(u_isn oa_wa))	17	13
7896	((((u_csn (u_isn cp_ent))(p_isn ca_wa))(p_re ca_wa)))(cp_ent oa_ent)(u_isn u_hdip)))(u_isn u_hdip))	18	13
7897	((ca_ent ca_wa)((((p_re ca_wa)((cp_ent oa_ent) oa_wa))(u_hdip u_hdip))(u_isn u_hdip))(u_isn oa_wa)))	26	13
7898	(((((ca_ent ca_wa) cp_ent)((p_isn oa_ent)(u_isn u_hdip)))(u_isn u_hdip))(u_isn u_hdip))(oa_wa u_csn))	22	13
7899	(((((ca_ent p_isn))((p_re ca_wa) p_csn)(cp_ent oa_ent)))(u_isn oa_wa))(u_isn oa_wa))(u_isn u_hdip))	21	13
7900	((ca_ent ca_wa)((((p_re ca_wa) p_csn)(oa_ent u_isn))(u_hdip u_hdip))(u_isn u_hdip))(u_isn u_hdip)))	23	13
7901	(((cp_ent ca_ent))((((ca_wa p_re) ca_wa)(cp_ent oa_ent))(u_hdip u_hdip))(u_isn u_hdip)))(u_isn oa_wa))	29	13
7902	(((((p_csn oa_ent)(u_isn u_hdip))(u_isn u_hdip))(u_isn oa_wa))((u_csn cp_ent) p_csn))(ca_ent p_isn))	23	13
7903	(((((p_csn oa_ent)(u_isn u_hdip))(u_isn u_hdip))(u_isn oa_wa))((u_csn cp_ent) p_csn))(ca_ent ca_wa))	23	13
7904	(((((u_csn cp_ent)(p_isn ca_ent))(ca_wa p_re))(p_isn ca_wa)))(oa_ent u_isn) u_hdip))(u_isn u_hdip))	23	13
7905	(((((u_csn cp_ent)(p_isn ca_wa))(p_isn p_re))((cp_ent oa_ent) u_hdip))(u_isn u_hdip))(u_isn u_hdip))	21	13
7906	(((((u_hdip u_csn)(ca_ent ca_wa))(p_isn ca_wa))((cp_ent oa_ent) u_hdip))(u_isn u_hdip))(u_isn u_hdip))	19	13
7907	(((((u_hdip u_csn)(cp_ent ca_ent))((ca_wa p_re) ca_wa))(cp_ent oa_ent))(u_hdip u_hdip))(u_isn u_hdip))	22	13
7908	((ca_ent ca_wa)((((p_re ca_wa)(p_csn (oa_ent u_isn)))(u_hdip u_hdip))(u_isn u_hdip))(u_isn u_hdip)))	22	13
7909	((ca_ent ca_wa)((((p_re ca_wa)((cp_ent oa_ent) oa_wa))(u_isn u_hdip))(u_isn u_hdip))(u_isn oa_wa)))	27	13
7910	((ca_ent ca_wa)((((p_re ca_wa)((cp_ent oa_ent) oa_wa))(u_isn u_hdip))(u_isn u_hdip))(u_isn u_hdip)))	27	13
7911	((ca_ent ca_wa)((((p_re ca_wa)((cp_ent oa_ent) u_hdip))(u_isn u_hdip))(u_isn u_hdip))(oa_wa u_csn)))	31	13
7912	((ca_ent ca_wa)((((p_re ca_wa)((cp_ent oa_ent) u_hdip))(u_isn u_hdip))(u_isn u_hdip))(u_isn oa_wa)))	31	13
7913	((ca_ent ca_wa)((((p_re ca_wa)((cp_ent oa_ent) u_hdip))(u_isn u_hdip))(u_isn u_hdip))(u_isn u_hdip)))	29	13
7914	((ca_ent ca_wa)((((p_re ca_wa)((p_csn oa_ent) u_hdip))(u_isn u_hdip))(u_isn u_hdip))(u_isn u_hdip)))	22	13
7915	((ca_ent p_isn)((((p_re ca_wa)(p_csn (oa_ent u_isn)))(u_hdip u_hdip))(u_isn u_hdip))(u_isn u_hdip)))	17	13
7916	((ca_ent p_isn)((((p_re ca_wa)((cp_ent oa_ent) oa_wa))(u_hdip u_hdip))(u_isn u_hdip))(u_isn oa_wa)))	22	13
7917	((ca_ent p_isn)((((p_re ca_wa)((cp_ent oa_ent) oa_wa))(u_isn u_hdip))(u_isn u_hdip))(u_isn oa_wa)))	25	13
7918	((ca_ent p_isn)((((p_re ca_wa)((cp_ent oa_ent) oa_wa))(u_isn u_hdip))(u_isn u_hdip))(u_isn u_hdip)))	25	13
7919	((ca_ent p_isn)((((p_re ca_wa)((cp_ent oa_ent) u_hdip))(u_isn u_hdip))(u_isn u_hdip))(oa_wa u_csn)))	30	13
7920	((ca_ent p_isn)((((p_re ca_wa)((cp_ent oa_ent) u_hdip))(u_isn u_hdip))(u_isn u_hdip))(u_isn oa_wa)))	30	13
7921	((ca_ent p_isn)((((p_re ca_wa)((cp_ent oa_ent) u_hdip))(u_isn u_hdip))(u_isn u_hdip))(u_isn u_hdip)))	27	13
7922	((ca_ent p_isn)((((p_re ca_wa)((p_csn oa_ent) u_hdip))(u_isn u_hdip))(u_isn u_hdip))(u_isn u_hdip)))	17	13
7923	((ca_ent p_isn)((((p_re ca_wa) p_csn)(oa_ent u_isn))(u_hdip u_hdip))(u_isn u_hdip))(u_isn u_hdip)))	18	13
7924	((cp_ent ca_ent)((((p_re ca_wa)((cp_ent oa_ent) oa_wa))(u_hdip u_hdip))(u_isn u_hdip))(u_isn oa_wa)))	22	13
7925	((cp_ent ca_ent)((((p_re ca_wa)((cp_ent oa_ent) oa_wa))(u_isn u_hdip))(u_isn u_hdip))(u_isn oa_wa)))	25	13
7926	((cp_ent ca_ent)((((p_re ca_wa)((cp_ent oa_ent) oa_wa))(u_isn u_hdip))(u_isn u_hdip))(u_isn u_hdip)))	23	13
7927	((cp_ent ca_ent)((((ca_wa p_re) ca_wa)(cp_ent oa_ent))(oa_wa u_hdip))(u_isn u_hdip))(u_isn u_hdip)))	26	13
7928	((cp_ent ca_ent)((((ca_wa p_re) ca_wa)(cp_ent oa_ent))(u_hdip u_hdip))(u_isn oa_wa))(u_csn cp_ent)))	22	13
7929	((cp_ent ca_ent)((((ca_wa p_re) ca_wa)(cp_ent oa_ent))(u_isn u_hdip))(u_isn oa_wa))(u_isn oa_wa)))	28	13
7930	((cp_ent ca_ent)((((ca_wa p_re) ca_wa)(cp_ent oa_ent))(u_isn u_hdip))(u_isn oa_wa))(u_isn u_hdip)))	27	13
7931	((cp_ent ca_ent)((((p_re ca_wa) p_csn)(oa_ent u_isn))(u_hdip u_hdip))(u_isn u_hdip))(u_isn u_hdip)))	19	13
7932	((cp_ent ca_wa)((((p_re ca_wa)((cp_ent oa_ent) u_hdip))(u_isn u_hdip))(u_isn u_hdip))(u_isn oa_wa)))	28	13
7933	((p_isn ca_ent)((((ca_wa p_re) ca_wa)(p_csn oa_ent))(u_isn u_hdip))(u_isn oa_wa))(u_isn u_hdip)))	23	13
7934	((p_isn ca_ent)((((p_re ca_wa)((cp_ent oa_ent) oa_wa))(u_hdip u_hdip))(u_isn u_hdip))(u_isn oa_wa)))	17	13
7935	((p_isn ca_ent)((((p_re ca_wa)((cp_ent oa_ent) oa_wa))(u_isn u_hdip))(u_isn u_hdip))(u_isn oa_wa)))	20	13
7936	((p_isn ca_ent)((((p_re ca_wa)((cp_ent oa_ent) oa_wa))(u_isn u_hdip))(u_isn u_hdip))(u_isn u_hdip)))	22	13
7937	((p_isn ca_ent)((((ca_wa p_re) ca_wa) oa_ent)(u_hdip u_hdip))(u_isn u_hdip))(oa_wa (u_csn cp_ent))))	23	13
7938	((p_isn ca_ent)((((ca_wa p_re) ca_wa)(cp_ent oa_ent))(oa_wa u_hdip))(u_isn u_hdip))(u_isn u_hdip)))	24	13
7939	((p_isn ca_ent)((((ca_wa p_re) ca_wa)(cp_ent oa_ent))(u_hdip u_hdip))(u_isn oa_wa))(u_csn cp_ent)))	22	13
7940	((p_isn ca_ent)((((ca_wa p_re) ca_wa)(cp_ent oa_ent))(u_isn u_hdip))(u_isn oa_wa))(u_isn oa_wa)))	27	13
7941	((p_isn ca_ent)((((ca_wa p_re) ca_wa)(cp_ent oa_ent))(u_isn u_hdip))(u_isn oa_wa))(u_isn u_hdip)))	27	13
7942	((p_isn ca_ent)((((ca_wa p_re) ca_wa)(oa_ent u_isn))(u_hdip u_hdip))(u_isn u_hdip))(oa_wa u_csn)))	24	13
7943	((p_isn ca_ent)((((ca_wa p_re) ca_wa)(oa_ent u_isn))(u_hdip u_hdip))(u_isn u_hdip))(u_isn oa_wa)))	23	13
7944	((p_isn ca_ent)((((ca_wa p_re) cp_ent)(oa_ent u_hdip))(u_isn u_hdip))(u_isn u_hdip))(u_isn oa_wa)))	25	13
7945	((p_isn ca_ent)((((ca_wa p_re) cp_ent)(oa_ent u_isn))(u_hdip u_hdip))(u_isn u_hdip))(u_isn oa_wa)))	23	13
7946	((p_isn ca_wa)(((p_isn p_re)(((ca_wa p_csn) oa_ent)(u_hdip u_hdip)))(u_isn u_hdip))(u_isn u_hdip)))	19	13
7947	((p_isn ca_wa)((((p_re ca_wa)(p_csn (oa_ent u_isn)))(u_hdip u_hdip))(u_isn u_hdip))(u_isn u_hdip)))	23	13
7948	((p_isn ca_wa)((((p_re ca_wa)((cp_ent oa_ent) oa_wa))(u_hdip u_hdip))(u_isn u_hdip))(u_isn oa_wa)))	26	13
7949	((p_isn ca_wa)((((p_re ca_wa)((cp_ent oa_ent) oa_wa))(u_isn u_hdip))(u_isn u_hdip))(u_isn oa_wa)))	26	13
7950	((p_isn ca_wa)((((p_re ca_wa)((cp_ent oa_ent) oa_wa))(u_isn u_hdip))(u_isn u_hdip))(u_isn u_hdip)))	27	13
7951	((p_isn ca_wa)((((p_re ca_wa)((cp_ent oa_ent) u_hdip))(u_isn u_hdip))(u_isn u_hdip))(oa_wa u_csn)))	29	13
7952	((p_isn ca_wa)((((p_re ca_wa)((cp_ent oa_ent) u_hdip))(u_isn u_hdip))(u_isn u_hdip))(u_isn oa_wa)))	29	13
7953	((p_isn ca_wa)((((p_re ca_wa)((cp_ent oa_ent) u_hdip))(u_isn u_hdip))(u_isn u_hdip))(u_isn u_hdip)))	29	13
7954	((p_isn ca_wa)((((p_re ca_wa)((p_csn oa_ent) u_hdip))(u_isn u_hdip))(u_isn u_hdip))(u_isn u_hdip)))	22	13
7955	((p_isn ca_wa)((((p_re ca_wa) p_csn)(oa_ent u_isn))(u_hdip u_hdip))(u_isn u_hdip))(u_isn u_hdip)))	24	13
7956	((u_csn (cp_ent ca_ent))((((ca_wa p_re) ca_wa) oa_ent)(u_hdip u_hdip))(u_isn u_hdip))(u_isn oa_wa)))	19	13
7957	((u_csn (cp_ent ca_ent))((((ca_wa p_re) cp_ent) oa_ent)(u_isn u_hdip))(u_isn u_hdip))(u_isn u_hdip)))	23	13
7958	((u_isn cp_ent)((p_isn ca_ent))(((ca_wa p_re) ca_wa)(cp_ent oa_ent))(u_isn oa_wa))(u_isn u_hdip))))	19	13
7959	((u_csn (p_isn ca_ent))((((ca_wa p_re) cp_ent) oa_ent)(u_isn u_hdip))(u_isn u_hdip))(u_isn u_hdip)))	20	13
7960	((u_csn (u_isn cp_ent))((p_isn ca_ent))(((ca_wa p_re) cp_ent) oa_ent)(u_isn u_hdip)))(u_isn oa_wa)))	18	13
7961	(((ca_ent ca_wa))(((p_re ca_wa)(p_csn (oa_ent u_isn)))(u_hdip u_hdip))(u_isn u_hdip)))(u_isn u_hdip))	24	13
7962	(((ca_ent ca_wa))(((p_re ca_wa)((cp_ent oa_ent) oa_wa))(u_hdip u_hdip))(u_isn u_hdip)))(u_isn oa_wa))	27	13
7963	(((ca_ent ca_wa))(((p_re ca_wa)((cp_ent oa_ent) oa_wa))(u_isn u_hdip))(u_isn u_hdip)))(u_isn oa_wa))	28	13
7964	(((ca_ent ca_wa))(((p_re ca_wa)((cp_ent oa_ent) u_hdip))(u_isn u_hdip))(u_isn u_hdip)))(u_isn u_hdip))	30	13
7965	(((ca_ent ca_wa))(((p_re ca_wa)((p_csn oa_ent) u_hdip))(u_isn u_hdip))(u_isn u_hdip)))(u_isn u_hdip))	23	13
7966	(((ca_ent ca_wa))(((p_re ca_wa) p_csn)(oa_ent u_isn))(u_hdip u_hdip))(u_isn u_hdip)))(u_isn u_hdip))	25	13
7967	(((((ca_wa p_re) ca_wa)(cp_ent oa_ent))(u_hdip u_hdip))(u_isn u_hdip))(u_isn oa_wa))(u_csn cp_ent))	27	13
7968	(((((ca_wa p_re) ca_wa)(cp_ent oa_ent))(u_hdip u_hdip))(u_isn u_hdip))(u_isn u_hdip))(u_csn cp_ent))	29	13
7969	(((((ca_wa p_re) ca_wa)(cp_ent oa_ent))(u_hdip u_hdip))(u_isn u_hdip))(u_isn u_hdip))(u_isn oa_wa))	26	13
7970	(((ca_ent p_isn))(((p_re ca_wa)(p_csn (oa_ent u_isn)))(u_hdip u_hdip))(u_isn u_hdip)))(u_isn u_hdip))	20	13
7971	(((ca_ent p_isn))(((p_re ca_wa)((cp_ent oa_ent) oa_wa))(u_hdip u_hdip))(u_isn u_hdip)))(u_isn oa_wa))	24	13
7972	(((ca_ent p_isn))(((p_re ca_wa) p_csn)(oa_ent u_isn))(u_hdip u_hdip))(u_isn u_hdip)))(u_isn u_hdip))	21	13
7973	(((cp_ent ca_ent))(((p_re ca_wa)((cp_ent oa_ent) oa_wa))(u_isn u_hdip))(u_isn u_hdip)))(u_isn u_hdip))	21	13
7974	(((cp_ent ca_ent))(((p_re ca_wa)((cp_ent oa_ent) u_hdip))(u_isn u_hdip))(u_isn u_hdip)))(u_isn oa_wa))	30	13
7975	(((cp_ent ca_ent))(((p_re ca_wa)((cp_ent oa_ent) u_hdip))(u_isn u_hdip))(u_isn u_hdip)))(u_isn u_hdip))	24	13
7976	(((cp_ent ca_ent))((((ca_wa p_re) ca_wa)(cp_ent oa_ent))(oa_wa u_hdip))(u_isn u_hdip)))(u_isn oa_wa))	26	13
7977	(((cp_ent ca_ent))((((ca_wa p_re) ca_wa)(cp_ent oa_ent))(u_hdip u_hdip))(u_isn u_hdip)))(u_isn u_hdip))	24	13
7978	(((cp_ent ca_ent))((((ca_wa p_re) ca_wa)(cp_ent oa_ent))(u_isn u_hdip))(u_isn oa_wa)))(u_isn oa_wa))	29	13
7979	(((cp_ent ca_ent))((((ca_wa p_re) ca_wa)(cp_ent oa_ent))(u_isn u_hdip))(u_isn oa_wa)))(u_isn u_hdip))	28	13
7980	(((cp_ent ca_wa))(((p_re ca_wa)((cp_ent oa_ent) u_hdip))(u_isn u_hdip))(u_isn u_hdip)))(u_isn u_hdip))	22	13
7981	(((p_csn ca_ent))((((ca_wa p_re) ca_wa)(cp_ent oa_ent))(u_isn u_hdip))(u_isn oa_wa)))(u_isn u_hdip))	19	13
7982	(((p_isn ca_ent))((((ca_wa p_re) ca_wa)(p_csn oa_ent))(u_isn u_hdip))(u_isn oa_wa)))(u_isn oa_wa))	23	13
7983	(((p_isn ca_ent))((((ca_wa p_re) ca_wa)(cp_ent oa_ent))(oa_wa u_hdip))(u_isn u_hdip)))(u_isn oa_wa))	24	13
7984	(((p_isn ca_ent))((((ca_wa p_re) ca_wa)(cp_ent oa_ent))(u_isn oa_wa))(u_isn oa_wa)))(u_isn u_hdip))	23	13
7985	(((p_isn ca_ent))((((ca_wa p_re) ca_wa)(cp_ent oa_ent))(u_isn u_hdip))(u_isn u_hdip)))(u_isn u_hdip))	29	13
7986	(((p_isn ca_ent))((((ca_wa p_re) ca_wa)(oa_ent u_isn))(u_hdip u_hdip))(u_isn u_hdip)))(u_csn cp_ent))	24	13
7987	(((p_isn ca_ent))((((ca_wa p_re) cp_ent)(oa_ent u_hdip))(u_isn oa_wa))(u_isn oa_wa)))(u_isn u_hdip))	22	13
7988	((((ca_ent p_isn))((p_re ca_wa)(p_csn (oa_ent u_isn)))(u_hdip u_hdip)))(u_isn u_hdip))(u_isn u_hdip))	23	13
7989	((((ca_ent p_isn))((p_re ca_wa) p_csn)(oa_ent u_isn))(u_hdip u_hdip)))(u_isn u_hdip))(u_isn u_hdip))	24	13
7990	(((u_hdip u_hdip)(((u_isn oa_wa)((u_csn cp_ent) p_csn)) ca_ent))((ca_wa p_re) cp_ent)))(oa_ent u_hdip))	21	13
7991	(((u_csn (p_isn ca_ent))((((ca_wa p_re) cp_ent) oa_ent)(u_isn u_hdip))(u_isn u_hdip)))(u_isn u_hdip))	22	13
7992	(((((ca_wa p_re) ca_wa)(p_csn oa_ent))(u_hdip u_hdip))(u_isn u_hdip))(u_isn u_hdip))(u_csn cp_ent))	23	13
7993	((((ca_ent p_isn))((p_re ca_wa)((p_csn (oa_ent u_isn))(u_hdip u_hdip)))(u_isn u_hdip))(u_isn u_hdip))	22	13
7994	((((p_isn ca_wa))((p_re ca_wa)((p_csn (oa_ent u_isn))(u_hdip u_hdip)))(u_isn u_hdip))(u_isn u_hdip))	22	13
7995	((((ca_ent p_isn))((p_re ca_wa)((cp_ent oa_ent) oa_wa))(u_hdip u_hdip)))(u_isn u_hdip))(oa_wa u_csn))	25	13
7996	((((ca_ent p_isn))((p_re ca_wa)((cp_ent oa_ent) oa_wa))(u_hdip u_hdip)))(u_isn u_hdip))(u_isn oa_wa))	28	13
7997	((((cp_ent ca_ent))((ca_wa p_re) ca_wa)((p_csn (oa_ent u_isn)) u_hdip)))(u_isn oa_wa))(u_isn u_hdip))	23	13
7998	((((cp_ent ca_ent))((p_re ca_wa)((cp_ent oa_ent) u_hdip))(u_isn u_hdip)))(u_isn oa_wa))(u_csn cp_ent))	24	13
7999	((((cp_ent ca_ent))(((ca_wa p_re) ca_wa)(cp_ent oa_ent))(oa_wa u_hdip)))(u_isn u_hdip))(u_isn oa_wa))	28	13
8000	((((cp_ent ca_ent))(((ca_wa p_re) ca_wa)(cp_ent oa_ent))(oa_wa u_hdip)))(u_isn u_hdip))(u_isn u_hdip))	27	13
8001	((((cp_ent ca_ent))(((ca_wa p_re) ca_wa)(cp_ent oa_ent))(u_isn u_hdip)))(u_isn oa_wa))(u_isn oa_wa))	30	13
8002	((((cp_ent ca_ent))(((ca_wa p_re) ca_wa)(cp_ent oa_ent))(u_isn u_hdip)))(u_isn oa_wa))(u_isn u_hdip))	30	13
8003	((((cp_ent ca_ent))(((ca_wa p_re) ca_wa)(oa_ent u_isn))(u_hdip u_hdip)))(u_isn u_hdip))(u_isn oa_wa))	25	13
8004	((((cp_ent ca_ent))(((ca_wa p_re) ca_wa)(p_csn oa_ent))(u_isn u_hdip)))(u_isn oa_wa))(u_isn oa_wa))	25	13
8005	((((cp_ent ca_ent))(((ca_wa p_re) ca_wa)(p_csn oa_ent))(u_isn u_hdip)))(u_isn oa_wa))(u_isn u_hdip))	24	13
8006	((((cp_ent ca_ent))(((ca_wa p_re) cp_ent)(oa_ent u_hdip))(u_isn u_hdip)))(u_isn u_hdip))(u_isn oa_wa))	29	13
8007	((((cp_ent ca_ent))(((ca_wa p_re) cp_ent)(oa_ent u_hdip))(u_isn u_hdip)))(u_isn u_hdip))(u_isn u_hdip))	24	13
8008	((((cp_ent ca_ent))(((ca_wa p_re) cp_ent)(oa_ent u_isn))(u_hdip u_hdip)))(u_isn u_hdip))(u_isn oa_wa))	26	13
8009	((((cp_ent ca_ent))(((ca_wa p_re) cp_ent)(oa_ent u_isn))(u_hdip u_hdip)))(u_isn u_hdip))(u_isn u_hdip))	21	13
8010	((((cp_ent ca_ent))(((ca_wa p_re) cp_ent)(oa_ent oa_wa))(u_isn u_hdip)))(u_isn u_hdip))(u_isn oa_wa))	25	13
8011	((((cp_ent ca_wa))((p_re ca_wa)((cp_ent oa_ent) oa_wa))(u_hdip u_hdip)))(u_isn oa_wa))(u_csn cp_ent))	21	13
8012	((((cp_ent ca_wa))((p_re ca_wa)((cp_ent oa_ent) oa_wa))(u_hdip u_hdip)))(u_isn u_hdip))(u_isn oa_wa))	20	13
8013	((((cp_ent ca_wa))((p_re ca_wa)((cp_ent oa_ent) oa_wa))(u_isn u_hdip)))(u_isn u_hdip))(u_isn oa_wa))	23	13
8014	((((cp_ent ca_wa))((p_re ca_wa)((cp_ent oa_ent) u_hdip))(u_isn u_hdip)))(u_isn u_hdip))(u_isn oa_wa))	25	13
8015	((((p_csn ca_ent))(((ca_wa p_re) ca_wa)(cp_ent oa_ent))(u_isn u_hdip)))(u_isn oa_wa))(u_isn u_hdip))	20	13
8016	((((p_isn ca_ent))(((ca_wa p_re) cp_ent)(oa_ent oa_wa))(u_hdip u_hdip)))(u_isn oa_wa))(u_csn cp_ent))	19	13
8017	((((p_isn ca_wa))((p_re ca_wa)((cp_ent oa_ent) oa_wa))(u_hdip u_hdip)))(u_isn u_hdip))(u_isn oa_wa))	27	13
8018	((((p_isn ca_wa))((p_re ca_wa) p_csn)(oa_ent u_hdip))(u_isn u_hdip)))(u_isn u_hdip))(u_isn u_hdip))	23	13
8019	((((ca_ent ca_wa))((p_re ca_wa) p_csn)(cp_ent oa_ent)))(u_isn oa_wa))(u_isn oa_wa))(u_isn u_hdip))	22	13
8020	((((ca_ent p_isn))((p_re ca_wa)((p_csn oa_ent) oa_wa))(u_isn u_hdip))(u_isn u_hdip))(u_isn u_hdip))	24	13
8021	((((ca_wa ca_wa)((p_csn (oa_ent u_isn))(u_hdip u_hdip)))(u_isn u_hdip))(u_isn u_hdip))(u_csn cp_ent))	21	13
8022	((((cp_ent ca_ent))((ca_wa p_re) ca_wa)(cp_ent oa_ent)))(oa_wa u_hdip))(u_isn u_hdip))(u_isn oa_wa))	27	13
8023	((((cp_ent ca_ent))((ca_wa p_re) ca_wa)(cp_ent oa_ent)))(oa_wa u_hdip))(u_isn u_hdip))(u_isn u_hdip))	26	13
8024	((((cp_ent ca_ent))((ca_wa p_re) ca_wa)(oa_ent u_isn))(u_hdip u_hdip))(u_isn u_hdip))(oa_wa u_csn))	25	13
8025	((((cp_ent ca_ent))((ca_wa p_re) ca_wa)(oa_ent u_isn))(u_hdip u_hdip))(u_isn u_hdip))(u_isn oa_wa))	26	13
8026	((((cp_ent ca_ent))((ca_wa p_re) cp_ent)(oa_ent oa_wa)))(u_hdip u_hdip))(u_isn u_hdip))(u_isn oa_wa))	21	13
8027	((((cp_ent ca_ent))((ca_wa p_re) cp_ent)(oa_ent u_hdip)))(u_isn u_hdip))(u_isn u_hdip))(u_isn u_hdip))	23	13
8028	((((cp_ent ca_wa)((p_re ca_wa)((cp_ent oa_ent) oa_wa)))(u_isn u_hdip))(u_isn u_hdip))(u_isn oa_wa))	25	13
8029	((((cp_ent ca_wa)((p_re ca_wa)((cp_ent oa_ent) oa_wa)))(u_isn u_hdip))(u_isn u_hdip))(u_isn u_hdip))	20	13
8030	((((p_isn ca_ent)((p_re ca_wa)((cp_ent oa_ent) u_hdip)))(u_isn u_hdip))(u_isn u_hdip))(u_isn oa_wa))	24	13
8031	((((p_isn ca_ent))((ca_wa p_re) ca_wa)(cp_ent oa_ent)))(oa_wa u_hdip))(u_isn u_hdip))(u_isn oa_wa))	24	13
8032	((((p_isn ca_ent))((ca_wa p_re) ca_wa)(cp_ent oa_ent)))(u_isn u_hdip))(u_isn oa_wa))(u_isn oa_wa))	28	13
8033	((((p_isn ca_ent))((ca_wa p_re) ca_wa)(cp_ent oa_ent)))(u_isn u_hdip))(u_isn oa_wa))(u_isn u_hdip))	28	13
8034	((((p_isn ca_ent))((ca_wa p_re) ca_wa)(cp_ent oa_ent)))(u_isn u_hdip))(u_isn u_hdip))(u_isn u_hdip))	30	13
8035	((((p_isn ca_ent))((ca_wa p_re) cp_ent)(oa_ent u_isn)))(u_hdip u_hdip))(u_isn u_hdip))(oa_wa u_csn))	26	13
8036	((((p_isn ca_ent))((ca_wa p_re) cp_ent)(oa_ent u_isn)))(u_hdip u_hdip))(u_isn u_hdip))(u_isn oa_wa))	26	13
8037	((((u_csn cp_ent))((ca_ent ca_wa) cp_ent)(oa_ent u_hdip)))(u_isn u_hdip))(u_isn u_hdip))(u_isn oa_wa))	21	13
8038	((((p_csn (oa_ent (u_isn u_hdip)))(u_isn oa_wa) u_hdip))((u_csn cp_ent) p_csn)) ca_ent)(ca_wa ca_wa))	21	13
8039	((((p_csn ((oa_ent u_isn) u_hdip))(u_isn oa_wa))(u_csn cp_ent))(p_csn (ca_ent ca_wa)))(p_re ca_wa))	23	13
8040	((((ca_ent ca_wa)((p_re ca_wa) p_csn))(cp_ent oa_ent))(u_isn u_hdip))(u_isn oa_wa))(u_isn oa_wa))	24	13
8041	((((ca_ent ca_wa)((p_re ca_wa) p_csn))(oa_ent u_isn))(u_hdip u_hdip))(u_isn u_hdip))(u_isn u_hdip))	24	13
8042	(((((cp_ent ca_ent)((ca_wa p_re) cp_ent)) oa_ent))(oa_wa u_hdip)(u_isn oa_wa)))((u_csn cp_ent)) oa_ent)	19	13
8043	(((((cp_ent ca_ent)((ca_wa p_re) cp_ent))(oa_ent oa_wa))(u_hdip u_hdip))(u_isn oa_wa))(u_csn cp_ent))	23	13
8044	(((((cp_ent ca_ent)((ca_wa p_re) cp_ent))(oa_ent oa_wa))(u_hdip u_hdip))(u_isn u_hdip))(u_isn oa_wa))	24	13
8045	(((((cp_ent ca_ent)((ca_wa p_re) cp_ent))(oa_ent oa_wa))(u_isn u_hdip))(u_isn u_hdip))(u_isn oa_wa))	26	13
8046	(((((cp_ent ca_ent)((ca_wa p_re) cp_ent))(oa_ent u_hdip))(u_isn u_hdip))(u_isn u_hdip))(u_isn oa_wa))	30	13
8047	(((((cp_ent ca_ent)((ca_wa p_re) cp_ent))(oa_ent u_hdip))(u_isn u_hdip))(u_isn u_hdip))(u_isn u_hdip))	25	13
8048	(((((cp_ent ca_ent)((ca_wa p_re) cp_ent))(oa_ent u_isn))(u_hdip u_hdip))(u_isn u_hdip))(u_isn oa_wa))	27	13
8049	(((((cp_ent ca_ent)((ca_wa p_re) cp_ent))(oa_ent u_isn))(u_hdip u_hdip))(u_isn u_hdip))(u_isn u_hdip))	22	13
8050	(((((p_csn oa_ent)((u_isn oa_wa) u_hdip))(u_isn oa_wa))((u_csn cp_ent) p_csn)) ca_ent)(ca_wa ca_wa))	20	13
8051	(((((p_re ca_wa)(p_csn (oa_ent u_isn)))(u_hdip u_hdip))(u_isn u_hdip))(u_isn u_hdip))(u_csn cp_ent))	22	13
8052	(((((p_re ca_wa)((cp_ent oa_ent) oa_wa))(u_isn u_hdip))(u_isn u_hdip))(u_isn oa_wa))(u_csn cp_ent))	22	13
8053	(((((p_re ca_wa)((cp_ent oa_ent) oa_wa))(u_isn u_hdip))(u_isn u_hdip))(u_isn u_hdip))(u_isn oa_wa))	25	13
8054	(((((p_re ca_wa)((cp_ent oa_ent) u_hdip))(u_isn u_hdip))(u_isn u_hdip))(u_isn oa_wa))(u_csn cp_ent))	26	13
8055	(((((p_re ca_wa)((cp_ent oa_ent) u_hdip))(u_isn u_hdip))(u_isn u_hdip))(u_isn u_hdip))(u_csn cp_ent))	27	13
8056	(((((p_re ca_wa)((cp_ent oa_ent) u_hdip))(u_isn u_hdip))(u_isn u_hdip))(u_isn u_hdip))(u_isn oa_wa))	25	13
8057	(((((p_csn (oa_ent u_isn))(u_hdip u_hdip))(u_isn oa_wa))((u_csn cp_ent) p_csn)) ca_ent)(ca_wa ca_wa))	23	13
8058	(((((u_hdip (u_csn cp_ent))(p_isn ca_wa))(p_isn ca_wa))(cp_ent oa_ent))(u_hdip u_hdip))(u_isn u_hdip))	19	13
8059	((((((ca_ent ca_wa) p_csn)(oa_ent u_isn))(u_hdip u_hdip))(u_isn u_hdip))(u_isn u_hdip))(u_isn u_csn))	19	13
8060	((((((ca_ent ca_wa) p_re)(cp_ent oa_ent))(oa_wa u_hdip))(u_isn u_hdip))(u_isn u_hdip))(u_isn oa_wa))	24	13
8061	((((((ca_ent ca_wa) p_re)(cp_ent oa_ent))(u_hdip u_hdip))(u_isn u_hdip))(u_isn oa_wa))(u_csn cp_ent))	24	13
8062	((((((ca_ent ca_wa) p_re)(cp_ent oa_ent))(u_hdip u_hdip))(u_isn u_hdip))(u_isn u_hdip))(u_isn oa_wa))	22	13
8063	((((((ca_wa p_re) cp_ent)(oa_ent u_hdip))(u_isn u_hdip))(u_isn u_hdip))(u_isn oa_wa))(u_csn cp_ent))	25	13
8064	((((((ca_wa p_re) ca_wa)(cp_ent oa_ent))(u_isn u_hdip))(u_isn oa_wa))(u_isn oa_wa))(u_isn u_hdip))	24	13
8065	((((((ca_wa p_re) ca_wa)(oa_ent u_isn))(u_hdip u_hdip))(u_isn u_hdip))(u_isn oa_wa))(u_csn cp_ent))	24	13
8066	((((((ca_wa p_re) ca_wa)(oa_ent u_isn))(u_hdip u_hdip))(u_isn u_hdip))(u_isn u_hdip))(u_csn cp_ent))	22	13
8067	((((((ca_wa p_re) ca_wa)(oa_ent u_isn))(u_hdip u_hdip))(u_isn u_hdip))(u_isn u_hdip))(u_isn oa_wa))	20	13
8068	((((((ca_wa p_re) cp_ent)(oa_ent oa_wa))(u_hdip u_hdip))(u_isn u_hdip))(u_isn oa_wa))(u_csn cp_ent))	21	13
8069	((((((ca_wa p_re) cp_ent)(oa_ent u_hdip))(u_isn u_hdip))(u_isn u_hdip))(u_isn u_hdip))(u_isn oa_wa))	24	13
8070	((((((ca_wa p_re) cp_ent)(oa_ent u_isn))(u_hdip u_hdip))(u_isn u_hdip))(u_isn oa_wa))(u_csn cp_ent))	21	13
8071	((((((cp_ent ca_ent) cp_ent)(oa_ent u_isn))(u_hdip u_hdip))(u_isn u_hdip))(u_isn u_hdip))(u_isn oa_wa))	19	13
8072	((((((cp_ent ca_wa) cp_ent)(oa_ent oa_wa))(u_isn u_hdip))(u_isn u_hdip))(u_isn oa_wa))(u_csn cp_ent))	21	13
8073	((((((cp_ent ca_wa) cp_ent)(oa_ent u_hdip))(u_isn u_hdip))(u_isn u_hdip))(u_isn oa_wa))(u_csn cp_ent))	24	13
8074	((((((cp_ent ca_wa) cp_ent)(oa_ent u_hdip))(u_isn u_hdip))(u_isn u_hdip))(u_isn u_hdip))(u_isn oa_wa))	21	13
8075	((((((u_isn oa_wa) u_hdip)(oa_wa u_csn)) oa_ent)((oa_wa u_hdip) oa_wa))(cp_ent ca_ent))(ca_wa ca_wa))	19	13
8076	(((u_isn cp_ent)(ca_ent ca_wa))(p_isn p_re)))((ca_wa (p_csn cp_ent)) oa_ent)(u_isn u_hdip))(u_isn u_hdip)))	17	14
8077	((u_hdip (u_isn oa_wa))(((u_csn cp_ent) p_csn)(ca_ent ca_wa)))((p_re ca_wa)(cp_ent oa_ent))(u_hdip u_hdip)))	19	14
8078	(((((u_isn cp_ent)(p_isn ca_ent))((ca_wa p_re) ca_wa))(p_csn cp_ent))(oa_ent u_isn) u_hdip))(u_isn u_hdip))	15	14
8079	((u_isn oa_wa) u_hdip)((((u_csn cp_ent) p_csn) ca_ent)(((ca_wa p_re) ca_wa)(cp_ent oa_ent))(u_hdip u_hdip)))	19	14
8080	((u_hdip u_hdip)((oa_wa (u_csn cp_ent))(p_csn ca_ent)))(((ca_wa p_re) ca_wa)(cp_ent oa_ent))(u_hdip u_hdip)))	19	14
8081	((u_hdip u_hdip)((((u_isn oa_wa) u_csn)(cp_ent ca_ent)))(((ca_wa p_re) ca_wa)(cp_ent oa_ent))(oa_wa u_hdip)))	19	14
8082	((u_hdip u_hdip)((((u_isn oa_wa) u_csn)(cp_ent ca_ent)))(((ca_wa p_re) ca_wa)(cp_ent oa_ent))(u_hdip u_hdip)))	23	14
8083	((u_hdip u_hdip)(((u_isn oa_wa)((u_csn cp_ent) p_csn)) ca_ent))(((ca_wa p_re) ca_wa)((cp_ent oa_ent) u_hdip)))	19	14
8084	((u_isn u_hdip)((((u_isn oa_wa) u_csn)(cp_ent ca_ent)))(((ca_wa p_re) ca_wa)(cp_ent oa_ent))(u_hdip u_hdip)))	19	14
8085	((u_isn u_hdip)(((u_isn oa_wa)((u_csn cp_ent) p_csn)) ca_ent))(((ca_wa p_re) ca_wa)((cp_ent oa_ent) u_hdip)))	18	14
8086	(((oa_wa (u_csn cp_ent)) ca_ent))((((ca_wa p_re) ca_wa)(cp_ent oa_ent))(u_isn u_hdip)))((u_isn oa_wa) u_hdip)))	21	14
8087	((oa_wa (u_csn (cp_ent ca_ent)))((((ca_wa p_re) cp_ent)((oa_ent u_isn) u_hdip))((oa_wa u_hdip)(u_isn oa_wa)))	18	14
8088	(((u_csn cp_ent)(p_isn ca_ent))((((ca_wa p_re) ca_wa)(p_csn ((p_isn oa_ent)(u_isn u_hdip))))(u_isn u_hdip)))	17	14
8089	(((oa_wa (u_csn cp_ent)) ca_ent))(((ca_wa p_re) ca_wa)(p_csn ((p_isn oa_ent)(u_isn u_hdip))))(u_isn u_hdip)))	18	14
8090	((((oa_wa (u_csn cp_ent)) p_csn) ca_ent))(((ca_wa p_re) ca_wa)(cp_ent oa_ent))(u_hdip u_hdip)))(u_isn u_hdip))	19	14
8091	(((oa_wa (u_csn cp_ent))(p_isn ca_ent))((((ca_wa p_re) cp_ent)((oa_ent u_isn) u_hdip)))((u_isn oa_wa) u_hdip)))	18	14
8092	(((oa_wa u_csn)(p_isn ca_ent))((((ca_wa p_re) ca_wa)(cp_ent oa_ent))(u_isn u_hdip)))((u_isn oa_wa) u_hdip)))	17	14
8093	(((u_hdip oa_wa) u_hdip)((((u_csn cp_ent) p_csn) ca_ent))(((ca_wa p_re) ca_wa)(cp_ent oa_ent))(u_hdip u_hdip))))	17	14
8094	(((u_isn oa_wa) u_hdip)(((u_csn cp_ent)(p_csn (p_isn ca_ent))))(((ca_wa p_re) cp_ent)((oa_ent u_isn) u_hdip))))	16	14
8095	(((oa_wa u_hdip)(((u_csn cp_ent) p_csn)(ca_ent ca_wa)))(((p_re ca_wa)((cp_ent oa_ent) u_hdip))(u_isn u_hdip)))	19	14
8096	(((oa_wa u_hdip)(((u_csn cp_ent) p_csn) ca_ent)))(((ca_wa p_re) cp_ent)((oa_ent u_isn) u_hdip))(u_isn u_hdip)))	20	14
8097	(((oa_wa u_hdip)(((u_csn cp_ent) p_csn) ca_ent)))(((ca_wa p_re) cp_ent) oa_ent)(u_hdip u_hdip))(u_isn u_hdip)))	18	14
8098	(((u_hdip oa_wa)(u_csn (cp_ent ca_ent)))((((ca_wa p_re) ca_wa)(cp_ent oa_ent))(oa_wa u_hdip))(u_isn u_hdip)))	18	14
8099	(((u_hdip oa_wa)((u_csn cp_ent)(ca_ent ca_wa)))((((p_re ca_wa)(cp_ent oa_ent))(oa_wa u_hdip))(u_isn u_hdip)))	18	14
8100	(((u_hdip oa_wa)(u_csn (cp_ent ca_ent)))((((ca_wa p_re) ca_wa)(cp_ent oa_ent))(u_hdip u_hdip))(u_isn u_hdip)))	22	14
8101	(((u_hdip oa_wa)(((u_csn cp_ent) p_csn)(ca_ent ca_wa)))(((p_re ca_wa)((cp_ent oa_ent) u_hdip))(u_isn u_hdip)))	18	14
8102	(((u_hdip u_hdip)((oa_wa (u_csn cp_ent))(p_csn ca_ent)))((((ca_wa p_re) ca_wa)(oa_ent u_isn))(u_hdip u_hdip)))	17	14
8103	((u_hdip (u_isn oa_wa))((((u_csn cp_ent) p_csn) ca_ent))(((ca_wa p_re) ca_wa)(oa_ent u_isn))(u_hdip u_hdip)))	17	14
8104	(((u_hdip oa_wa)(((u_csn cp_ent) p_csn) ca_ent)))(((ca_wa p_re) cp_ent)((oa_ent u_isn) u_hdip))(u_isn u_hdip)))	19	14
8105	(((u_hdip u_hdip)((oa_wa (u_csn cp_ent))(p_csn ca_ent)))((((ca_wa p_re) cp_ent)(oa_ent u_hdip))(u_isn u_hdip)))	19	14
8106	(((u_hdip u_hdip)((oa_wa (u_csn cp_ent))(p_csn ca_ent)))((((ca_wa p_re) cp_ent)(oa_ent u_isn))(u_hdip u_hdip)))	19	14
8107	(((u_hdip (u_isn oa_wa))(((u_csn cp_ent) p_csn) ca_ent))((((ca_wa p_re) cp_ent)(oa_ent u_hdip))(u_isn u_hdip)))	19	14
8108	(((u_hdip (u_isn oa_wa))(((u_csn cp_ent) p_csn) ca_ent))((((ca_wa p_re) cp_ent)(oa_ent u_isn))(u_hdip u_hdip)))	19	14
8109	(((u_hdip oa_wa)(((u_csn cp_ent) p_csn) ca_ent)))((((ca_wa p_re) cp_ent) oa_ent)(u_hdip u_hdip))(u_isn u_hdip)))	17	14
8110	(((u_hdip u_hdip)((u_csn cp_ent)(p_csn (ca_ent ca_wa))))((((p_re ca_wa)(cp_ent oa_ent) u_hdip))(u_isn u_hdip)))	16	14
8111	(((u_hdip u_hdip)((u_csn cp_ent)(p_csn ca_ent)))((((ca_wa p_re) cp_ent)((oa_ent u_isn) u_hdip))(u_isn u_hdip)))	17	14
8112	(((u_hdip u_hdip)((oa_wa (u_csn cp_ent))(ca_ent ca_wa)))(((p_re ca_wa)((cp_ent oa_ent) oa_wa))(u_isn u_hdip)))	20	14
8113	(((u_hdip u_hdip)((oa_wa (u_csn cp_ent))(ca_ent ca_wa)))(((p_re ca_wa)((cp_ent oa_ent) u_hdip))(u_isn u_hdip)))	23	14
8114	(((u_hdip u_hdip)((oa_wa (u_csn cp_ent)) ca_ent))((((ca_wa p_re) cp_ent)((oa_ent u_isn) u_hdip))(u_isn u_hdip)))	24	14
8115	(((oa_wa (u_csn cp_ent))(p_isn ca_ent))((((ca_wa p_re) ca_wa)(p_csn oa_ent))(u_isn u_hdip))(u_isn u_hdip)))	19	14
8116	(((oa_wa (u_csn cp_ent))(p_isn ca_ent))((((ca_wa p_re) ca_wa)((p_csn (p_isn oa_ent)) u_hdip))(u_isn u_hdip)))	16	14
8117	((((oa_wa (u_csn cp_ent))(p_isn ca_ent))(((ca_wa p_re) ca_wa)(cp_ent oa_ent))(u_isn u_hdip)))(u_isn u_hdip)))	23	14
8118	(((oa_wa (u_csn cp_ent))(p_isn ca_ent))((((ca_wa p_re) cp_ent)(oa_ent u_hdip))(u_isn u_hdip))(u_isn u_hdip)))	20	14
8119	(((oa_wa (u_csn cp_ent))(p_isn ca_wa))((((p_re ca_wa)((cp_ent oa_ent) u_hdip))(u_isn u_hdip))(u_isn oa_wa)))	18	14
8120	(((oa_wa (u_csn cp_ent))(p_isn ca_wa))((((p_re ca_wa)((cp_ent oa_ent) u_hdip))(u_isn u_hdip))(u_isn u_hdip)))	21	14
8121	(((oa_wa (u_csn cp_ent))(p_csn (p_isn ca_ent)))((((ca_wa p_re) ca_wa)((cp_ent oa_ent) u_hdip))(u_isn u_hdip)))	16	14
8122	(((oa_wa (u_csn cp_ent))(p_csn (p_isn ca_ent)))((((ca_wa p_re) cp_ent)((oa_ent u_isn) u_hdip)))(u_isn u_hdip))	18	14
8123	(((u_csn (u_isn cp_ent))(p_isn ca_ent))((((ca_wa p_re) ca_wa)(cp_ent oa_ent))(u_isn u_hdip))(u_isn u_hdip)))	19	14
8124	(((u_hdip (oa_wa u_csn))(cp_ent ca_ent))((((ca_wa p_re) ca_wa)(cp_ent oa_ent))(u_hdip u_hdip))(u_isn u_hdip)))	20	14
8125	(((u_hdip (oa_wa u_csn))(p_csn (ca_ent ca_wa)))((((p_re ca_wa)(cp_ent oa_ent))(u_hdip u_hdip))(u_isn u_hdip)))	15	14
8126	(((u_hdip (u_csn cp_ent))(p_csn ca_ent))((((ca_wa p_re) ca_wa)(cp_ent oa_ent))(u_hdip u_hdip))(u_isn u_hdip)))	18	14
8127	(((u_hdip (u_csn cp_ent))(p_isn ca_ent))((((ca_wa p_re) cp_ent)(oa_ent u_hdip))(u_isn u_hdip))(u_isn u_hdip)))	19	14
8128	(((u_hdip (u_csn cp_ent))(p_isn ca_wa))((((p_re ca_wa)((cp_ent oa_ent) u_hdip))(u_isn u_hdip))(u_isn u_hdip)))	20	14
8129	((((u_hdip (u_csn cp_ent))(p_csn (p_isn ca_ent)))(((ca_wa p_re) cp_ent)((oa_ent u_isn) u_hdip)))(u_isn u_hdip))	16	14
8130	(((u_hdip (u_isn oa_wa))((u_csn cp_ent)(ca_ent ca_wa)))((((p_re ca_wa)((cp_ent oa_ent) oa_wa))(u_hdip u_hdip)))	18	14
8131	(((u_hdip (u_isn oa_wa))((u_csn cp_ent)(ca_ent ca_wa)))((((p_re ca_wa)((cp_ent oa_ent) oa_wa))(u_isn u_hdip)))	19	14
8132	(((u_hdip (u_isn oa_wa))((u_csn cp_ent)(ca_ent ca_wa)))((((p_re ca_wa)((cp_ent oa_ent) u_hdip))(u_isn u_hdip)))	22	14
8133	(((u_hdip (u_isn oa_wa))(u_csn (cp_ent ca_ent)))((((ca_wa p_re) cp_ent)((oa_ent u_isn) u_hdip))(u_isn u_hdip)))	23	14
8134	(((u_hdip (u_isn oa_wa))(u_csn (cp_ent ca_ent)))((((p_re ca_wa)(cp_ent oa_ent))(oa_wa u_hdip))(u_isn u_hdip)))	17	14
8135	((((u_hdip (u_isn u_csn))(cp_ent ca_ent))((((ca_wa p_re) ca_wa)(cp_ent oa_ent))(u_hdip u_hdip)))(u_isn u_hdip)))	19	14
8136	((((u_hdip oa_wa) u_csn)(cp_ent ca_ent))((((ca_wa p_re) ca_wa)(p_csn oa_ent))(u_isn u_hdip))(u_isn u_hdip)))	19	14

8137	((((u_hdip oa_wa) u_csn)(cp_ent ca_ent))))(ca_wa p_re) ca_wa)((p_csn (p_isn oa_ent)) u_hdip)))(u_isn u_hdip))	17	14
8138	((((u_hdip oa_wa) u_csn)(cp_ent ca_ent))))(ca_wa p_re) ca_wa)(cp_ent oa_ent))(u_hdip u_hdip)))(u_isn u_hdip)))	23	14
8139	((((u_hdip oa_wa) u_csn)(cp_ent ca_ent))))(ca_wa p_re) ca_wa)(cp_ent oa_ent))(u_isn u_hdip))(u_isn u_hdip)))	23	14
8140	((((u_hdip oa_wa) u_csn)(cp_ent ca_ent))))(ca_wa p_re) ca_wa)(p_csn oa_ent))(u_hdip u_hdip))(u_isn u_hdip)))	18	14
8141	((((u_isn oa_wa) u_csn)(cp_ent ca_ent))))(ca_wa p_re) ca_wa)(cp_ent oa_ent))(oa_wa u_hdip))(u_isn u_hdip)))	18	14
8142	((((u_isn oa_wa) u_csn)(cp_ent ca_ent))))(ca_wa p_re) ca_wa)(cp_ent oa_ent))(u_hdip u_hdip))(u_isn u_hdip)))	22	14
8143	((((u_isn oa_wa)((u_csn cp_ent) p_csn))(ca_ent ca_wa))))(p_re ca_wa)((cp_ent oa_ent) u_hdip))(u_isn u_hdip)))	19	14
8144	((((u_isn oa_wa) u_hdip)(u_csn (cp_ent ca_ent))))(ca_wa p_re) ca_wa)((cp_ent oa_ent) u_hdip))(u_isn u_hdip)))	20	14
8145	((((u_isn oa_wa) u_hdip)((u_csn cp_ent)(p_csn ca_ent))))(ca_wa p_re) cp_ent)(oa_ent u_hdip))(u_isn u_hdip)))	19	14
8146	((((u_isn u_hdip)(oa_wa (u_csn cp_ent))) ca_ent))))(ca_wa p_re) cp_ent)(oa_ent u_isn) u_hdip))(u_isn u_hdip)))	20	14
8147	((((u_isn u_hdip)(oa_wa (u_csn cp_ent)))(p_csn ca_ent))))(ca_wa p_re) ca_wa)(cp_ent oa_ent))(u_hdip u_hdip)))	17	14
8148	((((u_isn u_hdip)(oa_wa (u_csn cp_ent)))(p_csn ca_ent))))(ca_wa p_re) cp_ent)(oa_ent u_hdip))(u_isn u_hdip)))	17	14
8149	((((u_isn u_hdip)(oa_wa (u_csn cp_ent)))(p_csn ca_ent))))(ca_wa p_re) cp_ent)(oa_ent u_isn))(u_hdip u_hdip)))	17	14
8150	((((cp_ent ca_wa))((p_re ca_wa)(cp_ent oa_ent))(oa_wa u_hdip))((u_isn u_hdip))(u_isn oa_wa))(u_csn cp_ent))	22	14
8151	((((u_hdip u_hdip)((u_isn oa_wa))(u_csn cp_ent) p_csn))) ca_ent)))(ca_wa p_re) cp_ent)(oa_ent u_isn) u_hdip)))	20	14
8152	((((u_hdip u_hdip)((u_isn oa_wa) u_csn)(cp_ent ca_ent))))(ca_wa p_re) ca_wa)(cp_ent oa_ent))(oa_wa u_hdip)))	20	14
8153	(((u_hdip u_hdip)((u_isn oa_wa) u_csn)(cp_ent ca_ent))))(ca_wa p_re) ca_wa)(oa_ent u_isn))(u_hdip u_hdip)))	20	14
8154	(((u_hdip u_hdip)((u_isn oa_wa) u_csn)(cp_ent ca_ent))))(ca_wa p_re) cp_ent)(oa_ent u_hdip))(u_isn u_hdip)))	23	14
8155	(((u_hdip u_hdip)((u_isn oa_wa) u_csn)(cp_ent ca_ent))))(ca_wa p_re) cp_ent)(oa_ent u_isn))(u_hdip u_hdip)))	23	14
8156	(((u_hdip u_hdip)((u_isn oa_wa) u_csn)(cp_ent ca_wa))))(p_re ca_wa)((cp_ent oa_ent) oa_wa))(u_isn u_hdip)))	17	14
8157	(((u_hdip u_hdip)((u_isn oa_wa) u_csn)(cp_ent ca_wa))))(p_re ca_wa)((cp_ent oa_ent) u_hdip))(u_isn u_hdip)))	17	14
8158	(((u_isn oa_wa)((u_csn cp_ent)(p_csn (p_isn ca_wa))))(p_re ca_wa)((cp_ent oa_ent) u_hdip))(u_isn u_hdip)))	16	14
8159	(((u_isn oa_wa)((u_hdip (u_csn cp_ent))(p_csn (p_isn ca_ent))))(ca_wa p_re) ca_wa)((cp_ent oa_ent) u_hdip)))	15	14
8160	(((u_isn oa_wa)((u_hdip (u_csn cp_ent))(p_csn (p_isn ca_ent))))(ca_wa p_re) cp_ent) oa_ent)(u_isn u_hdip)))	15	14
8161	(((u_isn u_hdip)(((u_isn oa_wa) u_csn)(cp_ent ca_ent))))(ca_wa p_re) cp_ent)(oa_ent u_hdip))(u_isn u_hdip)))	19	14
8162	(((u_isn u_hdip)(((u_isn oa_wa) u_csn)(cp_ent ca_ent))))(ca_wa p_re) cp_ent)(oa_ent u_isn))(u_hdip u_hdip)))	19	14
8163	(((u_isn oa_wa)((u_csn cp_ent) p_csn)) ca_ent)))(ca_wa p_re) cp_ent)(oa_ent u_isn) u_hdip))(u_isn u_hdip)))	20	14
8164	(((u_isn oa_wa) u_hdip))((u_csn cp_ent) p_csn)(p_isn ca_ent))))(ca_wa p_re) ca_wa)((cp_ent oa_ent) u_hdip)))	14	14
8165	(((u_isn oa_wa) u_hdip))((u_csn cp_ent) p_csn)(p_isn ca_ent))))(ca_wa p_re) cp_ent) oa_ent)(u_isn u_hdip)))	14	14
8166	(((p_isn ca_ent)((p_re ca_wa) oa_ent))(oa_wa u_hdip)(u_isn oa_wa))((u_csn cp_ent) oa_ent)(oa_wa u_hdip)))	18	14
8167	(((u_csn cp_ent)((ca_ent ca_wa) p_re))(p_isn ca_wa))(p_csn ((p_isn oa_ent)(u_isn u_hdip)))(u_isn u_hdip)))	18	14
8168	(((u_csn cp_ent)((p_isn ca_ent)(ca_wa p_re)))(p_isn ca_wa))((p_csn oa_ent)(u_isn u_hdip)))(u_isn u_hdip)))	19	14
8169	(((u_hdip oa_wa)((u_csn cp_ent) p_csn))(p_isn ca_wa))))(p_re ca_wa)((cp_ent oa_ent) u_hdip))(u_isn u_hdip)))	15	14
8170	(((u_hdip u_hdip)(oa_wa (u_csn cp_ent)))(ca_ent ca_wa))))(p_re ca_wa)((cp_ent oa_ent) oa_wa))(u_isn u_hdip)))	19	14
8171	(((u_hdip u_hdip)(oa_wa (u_csn cp_ent)))(ca_ent ca_wa))))(p_re ca_wa)((cp_ent oa_ent) u_hdip))(u_isn oa_wa)))	19	14
8172	(((u_hdip u_hdip)(oa_wa (u_csn cp_ent)))(ca_ent ca_wa))))(p_re ca_wa)((cp_ent oa_ent) u_hdip))(u_isn u_hdip)))	22	14
8173	(((u_hdip u_hdip)(oa_wa (u_csn cp_ent))) ca_ent))))(ca_wa p_re) cp_ent)(oa_ent u_isn) u_hdip))(u_isn u_hdip)))	23	14
8174	(((u_hdip u_hdip)(oa_wa (u_csn cp_ent))) ca_ent))))(ca_wa p_re) cp_ent)(oa_ent u_isn) u_hdip))(u_isn oa_wa)))	19	14
8175	(((u_hdip u_hdip)(oa_wa (u_csn cp_ent)))(p_csn (ca_ent ca_wa))))(p_re ca_wa)(cp_ent oa_ent))(u_hdip u_hdip)))	18	14
8176	(((u_hdip u_hdip)(oa_wa (u_csn cp_ent)))(p_csn ca_ent))))(ca_wa p_re) ca_wa)(oa_ent u_isn))(u_hdip u_hdip)))	16	14
8177	(((u_hdip u_hdip)(oa_wa (u_csn cp_ent)))(p_csn ca_ent))))(ca_wa p_re) cp_ent)(oa_ent u_hdip))(u_isn u_hdip)))	18	14
8178	(((u_hdip u_hdip)(oa_wa (u_csn cp_ent)))(p_csn ca_ent))))(ca_wa p_re) cp_ent)(oa_ent u_isn))(u_hdip u_hdip)))	18	14
8179	(((u_isn u_hdip)((u_isn oa_wa))(u_csn cp_ent) p_csn)))(ca_ent ca_wa))(p_re ca_wa)((cp_ent oa_ent) u_hdip)))	19	14
8180	(((u_isn oa_wa)((u_csn cp_ent) p_csn))(p_isn ca_ent))))(ca_wa p_re) cp_ent)(oa_ent u_hdip))(u_isn u_hdip)))	15	14
8181	(((u_isn oa_wa)((u_csn cp_ent) p_csn))(p_isn ca_ent))))(ca_wa p_re) ca_wa)(oa_ent u_isn))(u_hdip u_hdip)))	14	14
8182	(((u_isn u_hdip)((u_isn oa_wa) u_csn))(cp_ent ca_ent))))(ca_wa p_re) ca_wa)(cp_ent oa_ent))(u_hdip u_hdip)))	20	14
8183	(((u_isn u_hdip)((u_isn oa_wa) u_csn))(cp_ent ca_ent))))(ca_wa p_re) cp_ent)(oa_ent u_hdip))(u_isn u_hdip)))	20	14
8184	(((u_isn u_hdip)((u_isn oa_wa) u_csn))(cp_ent ca_ent))))(ca_wa p_re) cp_ent)(oa_ent u_isn))(u_hdip u_hdip)))	20	14
8185	(((oa_wa (u_csn cp_ent)) p_csn)(p_isn ca_ent))))(ca_wa p_re) ca_wa)((cp_ent oa_ent) u_hdip))(u_isn u_hdip)))	18	14
8186	(((oa_wa (u_csn cp_ent)) p_csn)(p_isn ca_ent))))(ca_wa p_re) cp_ent)(oa_ent u_isn) u_hdip)))(u_isn u_hdip))	20	14
8187	(((oa_wa (u_csn cp_ent))(p_isn ca_ent))))(ca_wa p_re) ca_wa)((cp_ent oa_ent)(oa_wa u_hdip)))(u_isn u_hdip))	17	14
8188	(((p_csn (oa_ent u_hdip))(u_isn oa_wa))(u_csn cp_ent))))(p_csn (ca_ent ca_wa))(p_isn p_re))(ca_wa p_csn)))	21	14
8189	(((u_hdip (u_isn oa_wa)) u_csn)(cp_ent ca_ent))))(ca_wa p_re) ca_wa)(p_csn (oa_ent u_isn)))(u_hdip u_hdip))	19	14
8190	(((u_hdip (u_isn oa_wa)) u_csn)(ca_ent ca_wa))((p_re ca_wa)((p_csn oa_ent)(u_isn u_hdip))))(u_isn u_hdip))	18	14
8191	(((u_hdip (u_isn oa_wa)) u_csn)(cp_ent ca_ent))))(ca_wa p_re) ca_wa)(p_csn ((p_isn oa_ent)(u_isn u_hdip))))	17	14
8192	(((u_hdip (u_isn oa_wa)) u_csn)(ca_ent ca_wa))))(p_re ca_wa) p_csn))(p_isn oa_ent) u_hdip)))(u_isn u_hdip))	16	14
8193	((((u_hdip (u_isn oa_wa)) u_csn)(cp_ent ca_wa))((p_re ca_wa)(cp_ent oa_ent)))(oa_wa u_hdip))(u_isn u_hdip))	18	14
8194	(((u_hdip (u_isn oa_wa)) u_csn)(cp_ent ca_ent))))(ca_wa p_re) cp_ent)(oa_ent u_isn) u_hdip))(u_isn u_hdip)))	24	14
8195	((((u_hdip (u_isn oa_wa)) u_csn)(cp_ent ca_wa))((p_re ca_wa)(cp_ent oa_ent))(oa_wa u_hdip)))(u_isn u_hdip))	17	14
8196	(((u_hdip (u_isn oa_wa)) u_csn)(cp_ent ca_ent))))(ca_wa p_re) cp_ent)(oa_ent (u_isn u_hdip)))(u_isn u_hdip)))	23	14
8197	(((u_hdip (u_isn oa_wa)) u_csn)(ca_ent ca_wa))))(p_re ca_wa) p_csn) oa_ent)(u_isn u_hdip))(u_isn u_hdip)))	19	14
8198	((((u_hdip (u_isn oa_wa)) u_csn)(cp_ent ca_wa))))(p_re ca_wa)(cp_ent oa_ent))(oa_wa u_hdip))(u_isn u_hdip)))	18	14
8199	(((u_hdip (u_isn oa_wa)) u_csn)(ca_ent ca_wa))))(p_re ca_wa)(p_csn oa_ent))(u_hdip u_hdip))(u_isn u_hdip)))	17	14
8200	(((u_hdip (u_isn oa_wa)) u_csn)(cp_ent ca_wa))))(p_re ca_wa)(cp_ent oa_ent))(u_hdip u_hdip))(u_isn u_hdip)))	20	14
8201	(((u_hdip (u_isn oa_wa)) u_csn)(cp_ent ca_wa))))(p_re ca_wa)(cp_ent oa_ent))(u_isn u_hdip))(u_isn u_hdip)))	18	14
8202	(((u_hdip (u_isn oa_wa)) u_csn (cp_ent ca_ent))))(ca_wa p_re) cp_ent)(oa_ent u_isn) u_hdip)))(u_isn u_hdip))	22	14
8203	((cp_ent ca_ent))))(ca_wa p_re) ca_wa)((cp_ent oa_ent) u_hdip))(u_isn u_hdip))(u_isn u_hdip))(oa_wa u_csn)))	26	14
8204	((oa_wa u_hdip))((u_csn cp_ent)(p_csn (p_isn ca_ent))))(ca_wa p_re) ca_wa)(cp_ent oa_ent))(u_hdip u_hdip)))	16	14
8205	((p_isn ca_ent))))(ca_wa p_re) ca_wa)((cp_ent oa_ent) u_hdip))(u_isn u_hdip))(u_isn u_hdip))(oa_wa u_csn)))	26	14
8206	((p_isn ca_ent))))(ca_wa p_re) ca_wa)((cp_ent oa_ent) u_hdip))(u_isn u_hdip))(u_isn u_hdip))(u_isn oa_wa)))	26	14
8207	(((p_isn ca_wa))))(p_re ca_wa)(cp_ent oa_ent))(oa_wa u_hdip))(u_isn u_hdip)))(u_isn oa_wa))(u_csn cp_ent))	21	14
8208	((u_csn (cp_ent ca_ent))))(ca_wa p_re) ca_wa)(cp_ent oa_ent))(u_isn u_hdip))(u_isn oa_wa))(u_isn u_hdip)))	21	14
8209	((u_csn (cp_ent ca_ent))))(ca_wa p_re) ca_wa)(cp_ent oa_ent))(u_hdip u_hdip))(u_isn u_hdip))(u_isn oa_wa)))	22	14
8210	((u_csn (cp_ent ca_ent))))(ca_wa p_re) ca_wa)(cp_ent oa_ent))(u_hdip u_hdip))(u_isn u_hdip))(u_isn u_hdip)))	21	14
8211	((u_hdip u_hdip))((u_isn oa_wa) u_csn)(cp_ent ca_ent))))(ca_wa p_re) ca_wa)(oa_ent u_isn))(u_hdip u_hdip)))	19	14
8212	((u_hdip u_hdip))((u_isn oa_wa)(u_csn cp_ent) p_csn)) ca_ent))(ca_wa p_re) cp_ent)((oa_ent u_isn) u_hdip)))	19	14
8213	((u_hdip u_hdip))((u_isn oa_wa)(u_csn cp_ent) p_csn)) ca_ent))(ca_wa p_re) cp_ent) oa_ent)(u_isn u_hdip)))	19	14
8214	((u_hdip u_hdip))((u_isn oa_wa)(u_csn cp_ent) p_csn)) ca_ent))(ca_wa p_re) ca_wa) oa_ent)(u_hdip u_hdip)))	16	14
8215	((u_isn u_hdip))((u_isn oa_wa)((u_csn cp_ent) p_csn)) ca_ent))(ca_wa p_re) cp_ent)((oa_ent u_isn) u_hdip)))	18	14
8216	((u_isn u_hdip))((u_isn oa_wa)((u_csn cp_ent) p_csn)) ca_ent))(ca_wa p_re) cp_ent) oa_ent)(u_isn u_hdip)))	18	14
8217	((oa_wa (u_csn (cp_ent ca_ent))))(ca_wa p_re) cp_ent)(oa_ent u_isn) u_hdip))(u_isn oa_wa))(u_isn oa_wa)))	19	14
8218	((oa_wa (u_csn (cp_ent ca_ent))))(ca_wa p_re) cp_ent)(oa_ent u_isn) u_hdip))(u_isn oa_wa))(u_isn u_hdip)))	20	14
8219	(((u_csn cp_ent) p_csn)(ca_ent ca_wa))))(p_re ca_wa)((cp_ent oa_ent) u_hdip))(u_isn u_hdip))(u_isn u_hdip)))	20	14
8220	(((u_csn cp_ent) p_csn) ca_ent))))(ca_wa p_re) cp_ent)(oa_ent u_isn) u_hdip))(u_isn u_hdip))(u_isn u_hdip)))	19	14
8221	((u_csn cp_ent)(p_isn ca_ent))))(ca_wa p_re) ca_wa)(p_csn (p_isn oa_ent)))(u_isn u_hdip))(u_isn u_hdip)))	18	14
8222	(((oa_wa (u_csn cp_ent)) ca_ent))))(ca_wa p_re) ca_wa)(p_csn ((p_isn oa_ent)(u_isn u_hdip)))(u_isn u_hdip)))	17	14
8223	(((oa_wa (u_csn cp_ent)) ca_ent))))(ca_wa p_re) ca_wa)(p_csn (oa_ent u_isn)))(u_hdip u_hdip))(u_isn u_hdip)))	19	14
8224	(((oa_wa (u_csn cp_ent))(ca_ent ca_wa))))(p_re ca_wa)((cp_ent oa_ent) oa_wa))(u_isn u_hdip))(u_isn u_hdip)))	21	14
8225	(((oa_wa (u_csn cp_ent))(ca_ent ca_wa))))(p_re ca_wa)((cp_ent oa_ent) u_hdip))(u_isn u_hdip))(u_isn u_hdip)))	24	14
8226	(((oa_wa (u_csn cp_ent)) ca_ent))))(ca_wa p_re) ca_wa)((cp_ent oa_ent) u_hdip))(u_isn u_hdip))(u_isn u_hdip)))	25	14
8227	(((oa_wa (u_csn cp_ent)) ca_ent))))(ca_wa p_re) ca_wa)(p_csn oa_ent) u_hdip))(u_isn u_hdip))(u_isn u_hdip)))	19	14
8228	(((oa_wa (u_csn cp_ent)) ca_ent))))(ca_wa p_re) ca_wa)((cp_ent oa_ent) oa_wa))(u_hdip u_hdip))(u_isn u_hdip)))	19	14
8229	(((oa_wa (u_csn cp_ent)) ca_ent))))(ca_wa p_re) ca_wa)(cp_ent oa_ent) oa_wa))(u_isn u_hdip))(u_isn u_hdip)))	19	14
8230	(((oa_wa (u_csn cp_ent)) ca_ent))))(ca_wa p_re) cp_ent)(oa_ent u_isn) u_hdip))(u_isn u_hdip))(u_isn u_hdip)))	22	14
8231	(((oa_wa (u_csn cp_ent))(ca_ent ca_wa))))(p_re ca_wa) p_csn)(oa_ent u_isn))(u_hdip u_hdip))(u_isn u_hdip)))	20	14
8232	(((oa_wa (u_csn cp_ent))(ca_ent ca_wa))))(p_re ca_wa)((cp_ent oa_ent) oa_wa))(u_hdip u_hdip))(u_isn u_hdip)))	18	14
8233	(((oa_wa u_csn)(cp_ent ca_wa))))(p_re ca_wa)(cp_ent oa_ent))(u_isn u_hdip))(u_isn u_hdip))(u_isn oa_wa)))	18	14
8234	(((oa_wa (u_csn cp_ent))(p_csn ca_ent))))(ca_wa p_re) cp_ent)(oa_ent u_hdip))(u_isn u_hdip))(u_isn u_hdip)))	19	14
8235	(((oa_wa (u_csn cp_ent))(p_csn ca_ent))))(ca_wa p_re) cp_ent)(oa_ent u_isn))(u_hdip u_hdip))(u_isn u_hdip)))	17	14
8236	(((oa_wa (u_csn cp_ent))(p_isn ca_ent))))(ca_wa p_re) ca_wa)(cp_ent oa_ent))(u_isn oa_wa))(u_isn oa_wa)))	18	14
8237	(((oa_wa (u_csn cp_ent))(p_isn ca_ent))))(ca_wa p_re) ca_wa)(cp_ent oa_ent))(u_isn oa_wa))(u_isn u_hdip)))	18	14
8238	(((oa_wa u_csn)(p_isn ca_ent))))(ca_wa p_re) cp_ent)((oa_ent u_isn) u_hdip))(u_isn oa_wa))(u_isn u_hdip)))	17	14
8239	(((u_csn cp_ent)(p_isn ca_ent))))(ca_wa p_re) cp_ent)((oa_ent u_isn) u_hdip))(u_isn oa_wa))(u_isn oa_wa)))	19	14
8240	(((u_csn cp_ent)(p_isn ca_ent))))(ca_wa p_re) cp_ent)((oa_ent u_isn) u_hdip))(u_isn oa_wa))(u_isn u_hdip)))	20	14
8241	(((u_csn cp_ent)(p_isn ca_ent))))(ca_wa p_re) cp_ent)((oa_ent u_isn) u_hdip))(u_isn u_hdip))(u_isn u_hdip)))	21	14
8242	(((u_csn cp_ent)(p_isn ca_wa))))(p_re ca_wa)(cp_ent oa_ent)(oa_wa u_hdip)))(u_isn u_hdip))(u_isn u_hdip)))	17	14
8243	(((u_csn cp_ent)(p_isn ca_wa))))(p_re ca_wa)(cp_ent oa_ent))(u_hdip u_hdip))(u_isn u_hdip))(u_isn oa_wa)))	20	14
8244	(((u_csn cp_ent)(p_isn ca_wa))))(p_re ca_wa)(cp_ent oa_ent))(u_hdip u_hdip))(u_isn u_hdip))(u_isn u_hdip)))	19	14
8245	(((u_csn cp_ent)(p_isn ca_wa))))(p_re ca_wa)(cp_ent oa_ent))(u_isn u_hdip))(u_isn u_hdip))(u_isn oa_wa)))	18	14
8246	(((u_hdip oa_wa) u_hdip))((u_csn cp_ent) p_csn) ca_ent))))(ca_wa p_re) cp_ent)(oa_ent u_hdip))(u_isn u_hdip)))	17	14
8247	(((u_hdip oa_wa) u_hdip))((u_csn cp_ent) p_csn) ca_ent))))(ca_wa p_re) cp_ent)(oa_ent u_isn))(u_hdip u_hdip)))	17	14
8248	(((u_hdip oa_wa) u_hdip))((u_csn cp_ent) p_csn)))(ca_wa p_re) cp_ent)((oa_ent u_isn) u_hdip))(u_isn u_hdip)))	16	14
8249	(((u_hdip (u_csn cp_ent))(ca_ent ca_wa))))(p_re ca_wa)((cp_ent oa_ent) oa_wa))(u_hdip u_hdip))(u_isn u_hdip)))	17	14
8250	(((u_hdip (u_csn cp_ent))(ca_ent ca_wa))))(p_re ca_wa)((cp_ent oa_ent) u_hdip))(u_isn u_hdip))(u_isn u_hdip)))	22	14
8251	(((u_hdip u_csn)(cp_ent ca_ent))))(ca_wa p_re) ca_wa)((cp_ent oa_ent) oa_wa))(u_hdip u_hdip))(u_isn u_hdip)))	18	14
8252	(((u_hdip u_csn)(cp_ent ca_ent))))(ca_wa p_re) cp_ent)(oa_ent u_isn) u_hdip))(u_isn u_hdip))(u_isn u_hdip)))	21	14
8253	(((u_isn oa_wa) u_hdip))((u_csn cp_ent)(p_csn (p_isn ca_ent))))(ca_wa p_re) cp_ent) oa_ent)(u_isn u_hdip)))	16	14
8254	(((u_isn u_csn)(cp_ent ca_ent))))(ca_wa p_re) cp_ent)(oa_ent u_isn) u_hdip))(u_isn u_hdip))(u_isn u_hdip)))	18	14
8255	(((oa_wa u_hdip))((u_csn cp_ent) p_csn) ca_ent))))(ca_wa p_re) ca_wa) oa_ent)(u_hdip u_hdip))(u_isn u_hdip)))	16	14
8256	(((oa_wa u_hdip))((u_csn cp_ent) p_csn) ca_ent))))(ca_wa p_re) cp_ent) oa_ent)(u_isn u_hdip))(u_isn u_hdip)))	19	14
8257	(((u_csn cp_ent)(p_csn (p_isn ca_ent))))(ca_wa p_re) ca_wa)(oa_ent u_isn))(u_hdip u_hdip))(u_isn u_hdip)))	15	14
8258	(((u_hdip oa_wa)(u_csn (cp_ent ca_ent))))(ca_wa p_re) ca_wa)(oa_ent u_isn))(u_hdip u_hdip))(u_isn u_hdip)))	17	14
8259	(((u_hdip oa_wa)(u_csn (cp_ent ca_ent))))(ca_wa p_re) cp_ent)(oa_ent u_hdip))(u_isn u_hdip))(u_isn u_hdip)))	22	14
8260	(((u_hdip oa_wa)(u_csn (cp_ent ca_ent))))(ca_wa p_re) cp_ent)(oa_ent u_isn))(u_hdip u_hdip))(u_isn u_hdip)))	20	14
8261	(((u_hdip oa_wa))((u_csn cp_ent)(p_csn ca_ent))))(ca_wa p_re) ca_wa) oa_ent)(u_hdip u_hdip))(u_isn u_hdip)))	15	14
8262	(((u_hdip oa_wa))((u_csn cp_ent) p_csn) ca_ent))))(ca_wa p_re) cp_ent) oa_ent)(u_isn u_hdip))(u_isn u_hdip)))	18	14
8263	(((u_hdip u_hdip))((u_csn cp_ent)(p_csn ca_ent))))(ca_wa p_re) cp_ent) oa_ent)(u_isn u_hdip))(u_isn u_hdip)))	16	14
8264	(((u_hdip u_hdip)((oa_wa (u_csn cp_ent)) ca_ent))))(ca_wa p_re) cp_ent) oa_ent)(u_isn u_hdip))(u_isn u_hdip)))	23	14
8265	(((oa_wa (u_csn cp_ent))(p_isn ca_ent))))(ca_wa p_re)(p_csn ((p_isn oa_ent)(u_isn u_hdip))))(u_isn u_hdip)))	15	14
8266	(((oa_wa (u_csn cp_ent))(p_isn ca_ent))))(ca_wa p_re) ca_wa)(p_csn ((p_isn oa_ent) u_hdip)))(u_isn u_hdip)))	15	14
8267	(((oa_wa (u_csn cp_ent))(p_isn ca_ent))))(ca_wa p_re) ca_wa)(cp_ent oa_ent))(u_isn u_hdip))(u_isn oa_wa)))	20	14
8268	(((oa_wa (u_csn cp_ent))(p_isn ca_ent))))(ca_wa p_re) ca_wa)(oa_ent u_isn))(u_hdip u_hdip))(u_isn u_hdip)))	17	14
8269	(((oa_wa (u_csn cp_ent))(p_isn ca_wa))))(p_re ca_wa)((cp_ent oa_ent) oa_wa))(u_hdip u_hdip))(u_isn u_hdip)))	16	14
8270	(((oa_wa (u_csn cp_ent))(p_isn ca_wa))))(p_re ca_wa)((cp_ent oa_ent) oa_wa))(u_isn u_hdip))(u_isn u_hdip)))	20	14
8271	(((oa_wa (u_csn cp_ent))(p_csn (p_isn ca_ent))))(ca_wa p_re) cp_ent) oa_ent)(u_isn u_hdip))(u_isn u_hdip)))	18	14
8272	(((u_csn (u_isn cp_ent))(p_isn ca_ent))))(ca_wa p_re) ca_wa)(cp_ent oa_ent))(u_isn oa_wa))(u_isn u_hdip)))	17	14
8273	(((u_hdip (oa_wa u_csn))(cp_ent ca_ent))))(ca_wa p_re) cp_ent)(oa_ent u_hdip))(u_isn u_hdip))(u_isn u_hdip)))	20	14
8274	(((u_hdip (oa_wa u_csn))(cp_ent ca_ent))))(ca_wa p_re) cp_ent)(oa_ent u_isn))(u_hdip u_hdip))(u_isn u_hdip)))	18	14
8275	(((u_hdip (oa_wa u_csn))(ca_ent ca_wa))))(p_re ca_wa)((cp_ent oa_ent) oa_wa))(u_hdip u_hdip))(u_isn u_hdip)))	15	14
8276	(((u_hdip (u_csn cp_ent)) ca_ent))))(ca_wa p_re) cp_ent)(oa_ent u_isn) u_hdip))(u_isn u_hdip))(u_isn u_hdip)))	22	14
8277	(((u_hdip (u_csn cp_ent)) ca_ent))))(ca_wa p_re) cp_ent)(oa_ent (oa_wa u_hdip)))(u_isn u_hdip))(u_isn u_hdip)))	16	14
8278	(((u_hdip (u_csn cp_ent))(p_csn ca_ent))))(ca_wa p_re) ca_wa)(oa_ent u_isn))(u_hdip u_hdip))(u_isn u_hdip)))	15	14
8279	(((u_hdip (u_csn cp_ent))(p_csn ca_ent))))(ca_wa p_re) cp_ent)(oa_ent u_hdip))(u_isn u_hdip))(u_isn u_hdip)))	18	14
8280	(((u_hdip (u_csn cp_ent))(p_csn ca_ent))))(ca_wa p_re) cp_ent)(oa_ent u_isn))(u_hdip u_hdip))(u_isn u_hdip)))	16	14
8281	(((u_hdip (u_csn cp_ent))(p_isn ca_ent))))(ca_wa p_re) ca_wa)(oa_ent u_isn))(u_hdip u_hdip))(u_isn u_hdip)))	16	14
8282	(((u_hdip (u_csn cp_ent))(p_isn ca_wa))))(p_re ca_wa)((cp_ent oa_ent) oa_wa))(u_hdip u_hdip))(u_isn u_hdip)))	16	14
8283	(((u_hdip (u_csn cp_ent))(p_csn (p_isn ca_ent))))(ca_wa p_re) cp_ent) oa_ent)(u_isn u_hdip))(u_isn u_hdip)))	16	14
8284	(((u_hdip (u_isn oa_wa))(u_csn (cp_ent ca_ent))))(ca_wa p_re) ca_wa) oa_ent)(u_hdip u_hdip))(u_isn u_hdip)))	18	14
8285	(((u_hdip (u_isn oa_wa))(u_csn (cp_ent ca_ent))))(ca_wa p_re) cp_ent) oa_ent)(u_isn u_hdip))(u_isn u_hdip)))	22	14
8286	(((u_hdip (u_isn u_csn))(cp_ent ca_ent))))(ca_wa p_re) cp_ent)(oa_ent u_hdip))(u_isn u_hdip))(u_isn u_hdip)))	19	14
8287	(((u_hdip (u_isn u_csn))(cp_ent ca_ent))))(ca_wa p_re) cp_ent)(oa_ent u_isn))(u_hdip u_hdip))(u_isn u_hdip)))	18	14
8288	(((u_hdip (u_isn u_csn))(cp_ent ca_wa))))(p_re ca_wa)((cp_ent oa_ent) oa_wa))(u_hdip u_hdip))(u_isn u_hdip)))	15	14
8289	(((u_csn cp_ent) p_csn)(p_isn ca_ent))))(ca_wa p_re) ca_wa)(oa_ent u_isn))(u_hdip u_hdip))(u_isn u_hdip)))	17	14
8290	(((u_hdip oa_wa) u_csn)(ca_ent ca_wa))((p_re ca_wa)((p_csn ((p_isn oa_ent)(u_isn u_hdip)))(u_isn u_hdip))))	16	14
8291	(((u_hdip oa_wa) u_csn)(cp_ent ca_ent))))(ca_wa p_re)(p_csn ((p_isn oa_ent)(u_isn u_hdip))))(u_isn u_hdip)))	16	14
8292	(((u_hdip oa_wa) u_csn)(cp_ent ca_ent))))(ca_wa p_re) ca_wa)(p_csn ((p_isn oa_ent) u_hdip))))(u_isn u_hdip))	16	14
8293	((((u_hdip oa_wa) u_csn)(cp_ent ca_wa))))(p_re ca_wa)((cp_ent oa_ent) oa_wa))(u_isn u_hdip))(u_isn u_hdip)))	19	14
8294	(((u_hdip oa_wa) u_csn)(ca_ent ca_wa))))(p_re ca_wa)((cp_ent oa_ent) u_hdip))(u_isn u_hdip))(u_isn u_hdip)))	22	14
8295	(((u_hdip oa_wa) u_csn)(cp_ent ca_ent))))(ca_wa p_re) ca_wa)(oa_ent u_isn))(u_hdip u_hdip))(u_isn u_hdip)))	18	14
8296	(((u_hdip oa_wa) u_csn)(cp_ent ca_ent))))(ca_wa p_re) cp_ent)(oa_ent u_hdip))(u_isn u_hdip))(u_isn u_hdip)))	23	14
8297	(((u_hdip oa_wa) u_csn)(ca_ent ca_wa))))(p_re ca_wa) p_csn)(oa_ent u_isn))(u_hdip u_hdip))(u_isn u_hdip)))	18	14
8298	(((u_hdip oa_wa) u_csn)(cp_ent ca_wa))))(p_re ca_wa)((cp_ent oa_ent) u_hdip))(u_isn u_hdip))(u_isn u_hdip)))	20	14
8299	((((u_isn oa_wa) u_csn)(ca_ent ca_wa))))(p_re ca_wa)((cp_ent oa_ent) oa_wa))(u_hdip u_hdip))(u_isn u_hdip)))	17	14
8300	((((u_isn oa_wa) u_csn)(ca_ent ca_wa))))(p_re ca_wa)((cp_ent oa_ent) oa_wa))(u_isn u_hdip))(u_isn u_hdip)))	18	14
8301	((((u_isn oa_wa) u_csn)(cp_ent ca_ent))))(ca_wa p_re) ca_wa)(oa_ent u_isn))(u_hdip u_hdip))(u_isn u_hdip)))	17	14
8302	(((u_isn oa_wa) u_csn)(cp_ent ca_ent))))(ca_wa p_re) cp_ent)(oa_ent u_hdip))(u_isn u_hdip))(u_isn u_hdip)))	22	14
8303	(((u_isn oa_wa) u_csn)(cp_ent ca_ent))))(ca_wa p_re) cp_ent)(oa_ent u_isn))(u_hdip u_hdip))(u_isn u_hdip)))	20	14
8304	((((u_isn oa_wa) u_csn)(cp_ent ca_wa))))(p_re ca_wa)((cp_ent oa_ent) u_hdip))(u_isn u_hdip))(u_isn u_hdip)))	19	14
8305	(((u_isn oa_wa) u_hdip)(u_csn (cp_ent ca_ent))))(ca_wa p_re) ca_wa) oa_ent)(u_hdip u_hdip))(u_isn u_hdip)))	16	14
8306	(((u_isn oa_wa)((u_csn cp_ent) p_csn)) ca_ent))))(ca_wa p_re) ca_wa) oa_ent)(u_hdip u_hdip))(u_isn u_hdip)))	16	14
8307	(((u_isn oa_wa)((u_csn cp_ent) p_csn)) ca_ent))))(ca_wa p_re) cp_ent) oa_ent)(u_isn u_hdip))(u_isn u_hdip)))	19	14
8308	(((u_hdip u_hdip)(oa_wa (u_csn cp_ent))) ca_ent))))(ca_wa p_re) cp_ent) oa_ent)(u_isn u_hdip))(u_isn oa_wa)))	19	14
8309	(((u_hdip u_hdip)(oa_wa (u_csn cp_ent))) ca_ent))))(ca_wa p_re) cp_ent) oa_ent)(u_isn u_hdip))(u_isn u_hdip)))	22	14
8310	(((oa_wa (u_csn cp_ent)) p_csn)(p_isn ca_ent))))(ca_wa p_re) cp_ent) oa_ent)(u_isn u_hdip))(u_isn u_hdip)))	20	14
8311	(((u_hdip (u_isn oa_wa)) u_csn)(cp_ent ca_ent))))(ca_wa p_re) ca_wa) oa_ent)(u_hdip u_hdip))(u_isn u_hdip)))	19	14
8312	((((u_hdip (u_isn oa_wa)) u_csn)(cp_ent ca_ent))))(ca_wa p_re) ca_wa) oa_ent)(u_isn u_hdip))(u_isn u_hdip)))	20	14
8313	(((u_hdip (u_isn oa_wa)) u_csn)(cp_ent ca_ent))))(ca_wa p_re) cp_ent) oa_ent)(u_isn u_hdip))(u_isn u_hdip)))	23	14
8314	(((u_hdip (u_isn oa_wa)) u_csn)(ca_ent ca_wa))))(p_re ca_wa) p_csn) oa_ent)(u_hdip u_hdip))(u_isn u_hdip)))	18	14
8315	(((cp_ent oa_ent) oa_wa))(u_isn u_hdip)(u_isn u_hdip))(u_isn oa_wa) u_csn))))(cp_ent oa_ent)(oa_wa u_hdip)))	15	14
8316	(((cp_ent oa_ent) oa_wa))(u_isn u_hdip)(u_isn u_hdip))(u_isn oa_wa) u_csn))))(cp_ent oa_ent)(u_isn u_hdip)))	15	14
8317	(((u_hdip u_hdip)((u_isn oa_wa)((u_csn cp_ent) p_csn)))(ca_ent ca_wa))(p_re ca_wa)((cp_ent oa_ent) u_hdip)))	20	14
8318	(((u_hdip u_hdip)((u_isn oa_wa)((u_csn cp_ent) p_csn)) ca_ent))))(ca_wa p_re) ca_wa) oa_ent)(u_hdip u_hdip)))	17	14
8319	(((u_hdip u_hdip)((u_isn oa_wa)(u_csn cp_ent) p_csn)) ca_ent))))(ca_wa p_re) cp_ent) oa_ent)(u_isn u_hdip)))	20	14
8320	(((u_hdip u_hdip)((oa_wa (u_csn cp_ent)) p_csn)(p_isn ca_ent))))(ca_wa p_re) ca_wa)((cp_ent oa_ent) u_hdip)))	16	14
8321	(((u_hdip u_hdip)((oa_wa (u_csn cp_ent)) p_csn)(p_isn ca_ent))))(ca_wa p_re) cp_ent)(oa_ent u_isn) u_hdip)))	16	14
8322	(((u_hdip u_hdip)((oa_wa (u_csn cp_ent)) p_csn)(p_isn ca_ent))))(ca_wa p_re) cp_ent) oa_ent)(u_isn u_hdip)))	16	14
8323	(((u_hdip u_hdip))((u_isn oa_wa) u_csn)(cp_ent ca_wa))((p_re ca_wa)((cp_ent oa_ent) oa_wa)))(u_isn u_hdip)))	18	14
8324	(((oa_wa (u_csn (cp_ent ca_ent))))(ca_wa p_re) cp_ent)(oa_ent u_isn) u_hdip))(u_isn oa_wa)))(u_isn u_hdip))	21	14
8325	((((u_csn cp_ent) p_csn)(ca_ent ca_wa))))(p_re ca_wa)((cp_ent oa_ent) u_hdip))(u_isn u_hdip)))(u_isn u_hdip))	19	14
8326	((((u_csn cp_ent)(p_csn ca_ent))))(ca_wa p_re) ca_wa)((cp_ent oa_ent) u_hdip))(u_isn u_hdip)))(u_isn u_hdip))	20	14
8327	(((oa_wa (u_csn cp_ent))(ca_ent ca_wa))))(p_re ca_wa)((cp_ent oa_ent) u_hdip))(u_isn u_hdip)))(u_isn u_hdip))	23	14
8328	(((oa_wa (u_csn cp_ent)) ca_ent))))(ca_wa p_re) cp_ent)(oa_ent u_isn) u_hdip))(u_isn u_hdip)))(u_isn u_hdip))	25	14
8329	(((oa_wa (u_csn cp_ent)) ca_ent))))(ca_wa p_re) cp_ent)(oa_ent (u_isn u_hdip)))(u_isn u_hdip)))(u_isn u_hdip))	24	14
8330	((((oa_wa u_csn)(cp_ent ca_wa))))(p_re ca_wa)(cp_ent oa_ent))(oa_wa u_hdip))(u_isn u_hdip)))(u_isn u_hdip)))	19	14
8331	((((oa_wa u_csn)(cp_ent ca_wa))))(p_re ca_wa)(cp_ent oa_ent))(u_hdip u_hdip))(u_isn u_hdip))(u_isn oa_wa)))	21	14
8332	(((oa_wa (u_csn cp_ent))(p_csn ca_ent))))(ca_wa p_re) ca_wa)(cp_ent oa_ent))(u_hdip u_hdip)))(u_isn u_hdip))	20	14
8333	(((oa_wa (u_csn cp_ent))(p_csn ca_ent))))(ca_wa p_re) cp_ent)(oa_ent u_hdip))(u_isn u_hdip)))(u_isn u_hdip))	20	14
8334	(((oa_wa u_csn)(p_isn ca_ent))))(ca_wa p_re) cp_ent)(oa_ent (u_isn u_hdip)))(u_isn oa_wa)))(u_isn u_hdip))	16	14
8335	((((u_csn cp_ent)(p_isn ca_ent))))(ca_wa p_re) cp_ent)((oa_ent u_isn) u_hdip)))(u_isn u_hdip)))(u_isn u_hdip))	23	14
8336	(((u_csn cp_ent)(p_isn ca_wa))))(p_re ca_wa)(cp_ent oa_ent))(u_isn u_hdip))(u_isn u_hdip)))(u_isn u_hdip))	21	14
8337	(((u_hdip (u_csn cp_ent))(ca_ent ca_wa))))(p_re ca_wa)((cp_ent oa_ent) u_hdip))(u_isn u_hdip)))(u_isn u_hdip))	21	14
8338	(((u_isn u_csn)(cp_ent ca_ent))))(ca_wa p_re) cp_ent)((oa_ent u_isn) u_hdip))(u_isn u_hdip)))(u_isn u_hdip))	16	14
8339	(((u_hdip oa_wa)(u_csn (cp_ent ca_ent))))(ca_wa p_re) cp_ent)(oa_ent u_hdip))(u_isn u_hdip)))(u_isn u_hdip))	21	14
8340	(((u_hdip oa_wa)(u_csn (cp_ent ca_ent))))(ca_wa p_re) cp_ent)(oa_ent u_isn))(u_hdip u_hdip)))(u_isn u_hdip))	18	14
8341	(((u_hdip oa_wa)(u_csn (cp_ent ca_ent))))(ca_wa p_re) ca_wa)(cp_ent oa_ent))(u_hdip u_hdip)))(u_isn u_hdip))	21	14
8342	(((oa_wa (u_csn cp_ent))(p_isn ca_ent))))(p_re ca_wa)((cp_ent oa_ent) oa_wa))(u_isn u_hdip)))(u_isn u_hdip))	15	14
8343	(((u_hdip (oa_wa u_csn))(cp_ent ca_ent))))(ca_wa p_re) cp_ent)(oa_ent u_hdip))(u_isn u_hdip)))(u_isn u_hdip))	19	14
8344	(((u_hdip (oa_wa u_csn))(cp_ent ca_ent))))(ca_wa p_re) cp_ent)(oa_ent u_isn))(u_hdip u_hdip)))(u_isn u_hdip))	16	14
8345	(((u_hdip (u_csn cp_ent))(p_isn ca_wa))))(p_re ca_wa)((cp_ent oa_ent) u_hdip))(u_isn u_hdip)))(u_isn u_hdip))	19	14
8346	(((u_hdip (u_isn u_csn))(cp_ent ca_ent))))(ca_wa p_re) cp_ent)(oa_ent u_isn))(u_hdip u_hdip)))(u_isn u_hdip))	16	14
8347	((((u_hdip oa_wa) u_csn)(ca_ent ca_wa))))(p_re ca_wa)((cp_ent oa_ent) u_hdip))(u_isn u_hdip)))(u_isn u_hdip))	18	14
8348	((((u_isn oa_wa) u_csn)(cp_ent ca_ent))))(ca_wa p_re) cp_ent)(oa_ent u_hdip))(u_isn u_hdip)))(u_isn u_hdip))	21	14
8349	((((u_isn oa_wa) u_csn)(cp_ent ca_ent))))(ca_wa p_re) cp_ent)(oa_ent u_isn))(u_hdip u_hdip)))(u_isn u_hdip))	18	14
8350	(((u_isn u_hdip)((u_isn oa_wa)((u_csn cp_ent) p_csn)) ca_ent))(ca_wa p_re) cp_ent)((oa_ent u_isn) u_hdip)))	19	14
8351	(((u_isn u_hdip)((u_isn oa_wa)((u_csn cp_ent) p_csn)) ca_ent))(ca_wa p_re) ca_wa) oa_ent)(u_hdip u_hdip)))	16	14
8352	(((u_isn u_hdip)((u_isn oa_wa)((u_csn cp_ent) p_csn)) ca_ent))(ca_wa p_re) cp_ent) oa_ent)(u_isn u_hdip)))	19	14
8353	(((oa_wa (u_csn (cp_ent ca_ent))))(ca_wa p_re) ca_wa)(cp_ent oa_ent))(u_hdip (u_isn oa_wa)))(u_isn oa_wa))	15	14
8354	(((u_hdip (u_csn cp_ent))((p_isn ca_ent)((p_isn ca_wa) p_csn))))(p_isn oa_ent)(u_isn u_hdip))(u_isn u_hdip))	14	14
8355	((((oa_wa (u_csn cp_ent)) ca_ent))(ca_wa p_re) cp_ent)(oa_ent u_isn) u_hdip)))(u_isn u_hdip))(u_isn u_hdip))	24	14
8356	((((oa_wa u_csn)(cp_ent ca_wa))((p_re ca_wa)((cp_ent oa_ent)(oa_wa u_hdip)))(u_isn u_hdip))(u_isn oa_wa))	20	14
8357	((((oa_wa u_csn)(cp_ent ca_wa))((p_re ca_wa)((cp_ent oa_ent)(oa_wa u_hdip)))(u_isn u_hdip))(u_isn u_hdip))	20	14
8358	((((u_hdip u_csn)(cp_ent ca_ent))((ca_wa p_re) cp_ent)((oa_ent u_isn) u_hdip)))(u_isn u_hdip))(u_isn u_hdip))	18	14
8359	((((u_isn oa_wa)(u_csn cp_ent) p_csn)) ca_ent))(ca_wa p_re) cp_ent)(oa_ent u_isn) u_hdip)))(u_isn u_hdip))	19	14
8360	(((p_csn ((oa_ent u_isn) u_hdip))(u_isn oa_wa))(u_csn cp_ent)))(p_csn (ca_ent ca_wa))((p_re ca_wa) p_csn)))	23	14
8361	((((u_csn cp_ent)(ca_ent ca_wa))((p_re ca_wa)(cp_ent oa_ent))(oa_wa u_hdip))(u_isn u_hdip))(u_isn u_hdip))	19	14
8362	((((u_csn cp_ent)(ca_ent ca_wa))((p_re ca_wa)(cp_ent oa_ent)))(u_isn u_hdip))(u_isn u_hdip))(u_isn u_hdip))	24	14
8363	((((u_csn cp_ent)(p_isn ca_ent))(ca_wa p_re)(p_isn ca_wa))(cp_ent oa_ent))(u_isn u_hdip))(u_isn u_hdip))	22	14
8364	((((u_hdip u_hdip)(oa_wa (u_csn cp_ent))) ca_ent)))(ca_wa p_re) cp_ent)(oa_ent u_isn) u_hdip)))(u_isn u_hdip))	22	14
8365	((((u_csn (u_isn cp_ent))(p_isn ca_ent))(ca_wa p_re) ca_wa))(cp_ent oa_ent)(u_isn u_hdip))(u_isn u_hdip))	21	14
8366	((((u_csn (u_isn cp_ent))(p_isn ca_wa))(p_re ca_wa))(cp_ent oa_ent)(u_isn u_hdip)))(u_isn oa_wa) u_hdip))	15	14
8367	(((u_hdip (u_isn oa_wa)) u_csn)(cp_ent ca_ent))))(ca_wa p_re) cp_ent)(oa_ent u_isn) u_hdip)))(u_isn u_hdip))	23	14
8368	((((u_hdip (u_isn oa_wa)) u_csn)(ca_ent ca_wa))((p_re ca_wa)(cp_ent oa_ent))(u_hdip u_hdip)))(u_isn u_hdip))	18	14
8369	(((cp_ent ca_ent))))(ca_wa p_re) ca_wa)(cp_ent oa_ent) u_hdip))(u_isn u_hdip))(u_isn u_hdip)))(u_isn oa_wa))	29	14
8370	((((oa_wa u_csn)(cp_ent ca_wa))(p_re ca_wa)))(cp_ent oa_ent)(oa_wa u_hdip)))(u_isn u_hdip))(u_isn oa_wa))	20	14
8371	((((oa_wa u_csn)(cp_ent ca_wa))(p_re ca_wa)))(cp_ent oa_ent)(oa_wa u_hdip))(u_isn u_hdip))(u_isn u_hdip))	20	14
8372	((((u_csn cp_ent)(p_isn ca_ent))(ca_wa p_re) ca_wa))(cp_ent oa_ent) u_hdip))(u_isn u_hdip))(u_isn u_hdip))	19	14
8373	((((u_isn cp_ent)(p_isn ca_wa))(p_re ca_wa))(cp_ent oa_ent)(u_isn u_hdip)))(u_isn oa_wa))(u_isn u_hdip))	16	14
8374	((ca_ent ca_wa))((p_isn p_re))((ca_wa p_csn)(oa_ent u_isn))(u_hdip u_hdip)))(u_isn u_hdip))(u_isn u_hdip)))	19	14
8375	((ca_ent ca_wa))((p_isn p_re))((ca_wa p_csn)(oa_ent u_hdip))(u_isn u_hdip)))(u_isn u_hdip))(u_isn u_hdip)))	19	14
8376	((ca_ent ca_wa))))(p_re ca_wa)((cp_ent oa_ent)(oa_wa u_hdip)))(u_isn u_hdip))(u_isn u_hdip))(u_isn oa_wa)))	27	14
8377	((ca_ent ca_wa))))(p_re ca_wa)(cp_ent oa_ent))(oa_wa u_hdip))(u_isn u_hdip))(u_isn u_hdip))(u_isn oa_wa)))	27	14
8378	((ca_ent ca_wa))))(p_re ca_wa)(cp_ent oa_ent))(u_hdip u_hdip))(u_isn u_hdip))(u_isn u_hdip))(u_csn cp_ent)))	27	14
8379	((ca_ent ca_wa))))(p_re ca_wa)(cp_ent oa_ent))(u_hdip u_hdip))(u_isn u_hdip))(u_isn u_hdip))(u_isn oa_wa)))	25	14
8380	((ca_ent ca_wa))))(p_re ca_wa)(p_csn oa_ent))(u_hdip u_hdip))(u_isn u_hdip))(u_isn u_hdip))(u_csn cp_ent)))	21	14
8381	((ca_ent p_isn))))(p_re ca_wa)((cp_ent oa_ent)(oa_wa u_hdip)))(u_isn u_hdip))(u_isn u_hdip))(u_isn oa_wa)))	25	14
8382	((ca_ent p_isn))))(p_re ca_wa)(cp_ent oa_ent))(oa_wa u_hdip))(u_isn u_hdip))(u_isn u_hdip))(u_isn oa_wa)))	25	14
8383	((ca_ent p_isn))))(p_re ca_wa)(cp_ent oa_ent))(u_hdip u_hdip))(u_isn u_hdip))(u_isn u_hdip))(u_csn cp_ent)))	23	14
8384	((ca_ent p_isn))))(p_re ca_wa)(cp_ent oa_ent))(u_hdip u_hdip))(u_isn u_hdip))(u_isn u_hdip))(u_isn oa_wa)))	23	14
8385	((ca_ent p_isn))))(p_re ca_wa)(p_csn oa_ent))(u_hdip u_hdip))(u_isn u_hdip))(u_isn u_hdip))(u_csn cp_ent)))	17	14
8386	((cp_ent ca_ent))((p_isn p_re))((ca_wa p_csn)(oa_ent u_isn))(u_hdip u_hdip)))(u_isn u_hdip))(u_isn u_hdip)))	17	14
8387	((cp_ent ca_ent))((p_isn p_re))((ca_wa p_csn)(oa_ent u_hdip))(u_isn u_hdip)))(u_isn u_hdip))(u_isn u_hdip)))	17	14
8388	((cp_ent ca_ent))))(ca_wa p_re) ca_wa)(p_csn (oa_ent u_isn)))(u_hdip u_hdip))(u_isn u_hdip))(u_isn u_hdip)))	18	14
8389	((cp_ent ca_ent))))(ca_wa p_re) ca_wa)(cp_ent oa_ent) u_hdip))(u_isn u_hdip))(u_isn u_hdip))(u_isn oa_wa)))	28	14
8390	((cp_ent ca_ent))))(ca_wa p_re) ca_wa)(cp_ent oa_ent) u_hdip))(u_isn u_hdip))(u_isn u_hdip))(u_isn u_hdip)))	25	14
8391	((cp_ent ca_ent))))(ca_wa p_re) ca_wa)(p_csn oa_ent) u_hdip))(u_isn u_hdip))(u_isn u_hdip))(u_isn u_hdip)))	18	14
8392	((cp_ent ca_ent))))(ca_wa ca_wa)(cp_ent oa_ent))(u_hdip u_hdip))(u_isn u_hdip))(u_isn oa_wa))(u_csn cp_ent)))	20	14
8393	((cp_ent ca_ent))))(ca_wa ca_wa)(cp_ent oa_ent))(u_hdip u_hdip))(u_isn u_hdip))(u_isn u_hdip))(u_isn oa_wa)))	18	14
8394	((cp_ent ca_ent))))(ca_wa p_re) ca_wa)((cp_ent oa_ent) oa_wa))(u_hdip u_hdip))(u_isn u_hdip))(oa_wa u_csn)))	20	14
8395	((cp_ent ca_ent))))(ca_wa p_re) ca_wa)((cp_ent oa_ent) oa_wa))(u_hdip u_hdip))(u_isn u_hdip))(u_isn oa_wa)))	23	14
8396	((cp_ent ca_ent))))(ca_wa p_re) ca_wa)((cp_ent oa_ent) oa_wa))(u_isn u_hdip))(u_isn u_hdip))(u_isn oa_wa)))	23	14
8397	(((p_isn ca_ent))))(ca_wa p_re) cp_ent) oa_ent)(u_isn u_hdip))(u_isn u_hdip))(u_isn u_hdip))(oa_wa u_csn))	25	14
8398	(((p_isn ca_ent))))(ca_wa p_re) cp_ent) oa_ent)(u_isn u_hdip))(u_isn u_hdip))(u_isn oa_wa)))(u_csn cp_ent))	22	14
8399	((p_isn ca_ent))))(ca_wa p_re) ca_wa)((cp_ent oa_ent) oa_wa))(u_hdip u_hdip))(u_isn u_hdip))(oa_wa u_csn)))	17	14
8400	((p_isn ca_ent))))(ca_wa p_re) ca_wa)((cp_ent oa_ent) oa_wa))(u_hdip u_hdip))(u_isn u_hdip))(u_isn oa_wa)))	18	14
8401	((p_isn ca_ent))))(ca_wa p_re) ca_wa)((cp_ent oa_ent) oa_wa))(u_isn u_hdip))(u_isn u_hdip))(u_isn oa_wa)))	18	14
8402	((p_isn ca_ent))))(p_re ca_wa)(cp_ent oa_ent))(u_hdip u_hdip))(u_isn u_hdip))(u_isn oa_wa))(u_csn cp_ent)))	20	14
8403	((p_isn ca_wa))))(p_re ca_wa)((cp_ent oa_ent)(oa_wa u_hdip)))(u_isn u_hdip))(u_isn u_hdip))(u_isn oa_wa)))	26	14
8404	((p_isn ca_wa))))(p_re ca_wa)(cp_ent oa_ent))(oa_wa u_hdip))(u_isn u_hdip))(u_isn u_hdip))(u_isn oa_wa)))	26	14
8405	((p_isn ca_wa))))(p_re ca_wa)(cp_ent oa_ent))(u_hdip u_hdip))(u_isn u_hdip))(u_isn oa_wa))(u_csn cp_ent)))	25	14
8406	((p_isn ca_wa))))(p_re ca_wa)(cp_ent oa_ent))(u_hdip u_hdip))(u_isn u_hdip))(u_isn u_hdip))(u_csn cp_ent)))	27	14
8407	((u_csn (cp_ent ca_ent))))(ca_wa p_re) ca_wa)(cp_ent oa_ent))(u_isn u_hdip))(u_isn oa_wa))(u_isn oa_wa)))	21	14
8408	((u_csn (cp_ent ca_ent))))(ca_wa p_re) cp_ent)(oa_ent u_hdip))(u_isn u_hdip))(u_isn u_hdip))(u_isn u_hdip)))	20	14
8409	((u_csn (cp_ent ca_ent))))(ca_wa p_re) cp_ent)(oa_ent u_isn))(u_hdip u_hdip))(u_isn u_hdip))(u_isn oa_wa)))	19	14
8410	((u_csn (ca_ent ca_wa))))(p_re ca_wa)((cp_ent oa_ent) oa_wa))(u_isn u_hdip))(u_isn u_hdip))(u_isn oa_wa)))	19	14
8411	((u_csn (cp_ent ca_wa))))(p_re ca_wa)((cp_ent oa_ent) oa_wa))(u_isn u_hdip))(u_isn u_hdip))(u_isn oa_wa)))	18	14
8412	((u_csn (cp_ent ca_wa))))(p_re ca_wa)((cp_ent oa_ent) u_hdip))(u_isn u_hdip))(u_isn u_hdip))(u_isn oa_wa)))	20	14
8413	(((u_csn cp_ent)(p_csn ca_ent))))(ca_wa p_re) cp_ent) oa_ent)(u_isn u_hdip))(u_isn u_hdip))(u_isn u_hdip)))	18	14
8414	((u_csn (p_isn ca_ent))))(ca_wa p_re) ca_wa)(cp_ent oa_ent))(u_isn u_hdip))(u_isn oa_wa))(u_isn oa_wa)))	18	14
8415	((u_csn (u_isn cp_ent))(p_isn ca_ent))))(ca_wa p_re) ca_wa)(cp_ent oa_ent))(u_isn oa_wa))(u_isn u_hdip)))	19	14
8416	(((oa_wa (u_csn cp_ent)) ca_ent))))(ca_wa p_re) cp_ent) oa_ent)(u_isn u_hdip))(u_isn u_hdip))(u_isn u_hdip)))	21	14
8417	(((oa_wa u_csn)(p_isn ca_ent))))(ca_wa p_re) cp_ent) oa_ent)(u_isn oa_wa))(u_isn oa_wa))(u_isn u_hdip)))	15	14
8418	(((u_csn cp_ent)(p_isn ca_ent))))(ca_wa p_re) cp_ent) oa_ent)(u_isn u_hdip))(u_isn u_hdip))(u_isn u_hdip)))	21	14
8419	(((u_hdip u_csn)(cp_ent ca_ent))))(ca_wa p_re) cp_ent) oa_ent)(u_isn u_hdip))(u_isn u_hdip))(u_isn u_hdip)))	20	14
8420	(((u_hdip (u_csn cp_ent)) ca_ent))))(ca_wa p_re) cp_ent) oa_ent)(u_isn u_hdip))(u_isn u_hdip))(u_isn u_hdip)))	21	14
8421	(((ca_ent ca_wa))))(p_re ca_wa)(cp_ent oa_ent))(u_hdip u_hdip))(u_isn u_hdip))(u_isn oa_wa)))(u_csn cp_ent))	27	14
8422	(((ca_ent ca_wa))))(p_re ca_wa)(cp_ent oa_ent))(u_hdip u_hdip))(u_isn u_hdip))(u_isn u_hdip)))(u_csn cp_ent))	29	14
8423	(((ca_ent ca_wa))))(p_re ca_wa)(cp_ent oa_ent))(u_hdip u_hdip))(u_isn u_hdip))(u_isn u_hdip))(u_isn oa_wa))	26	14
8424	((((cp_ent ca_ent)(ca_wa p_re))))(ca_wa (p_csn cp_ent)) oa_ent)(u_isn u_hdip)(u_isn oa_wa)))(u_isn oa_wa))	19	14
8425	(((ca_ent p_isn))))(p_re ca_wa)(cp_ent oa_ent))(u_hdip u_hdip))(u_isn u_hdip))(u_isn u_hdip)))(u_csn cp_ent))	25	14
8426	(((cp_ent ca_ent)((p_isn p_re))(ca_wa p_csn)(cp_ent oa_ent))(u_isn u_hdip)))(u_isn oa_wa)))(u_isn oa_wa))	22	14
8427	(((cp_ent ca_ent)((p_isn p_re))(ca_wa (p_csn cp_ent))(oa_ent (u_isn u_hdip)))(u_isn oa_wa)))(u_isn oa_wa))	18	14
8428	(((cp_ent ca_ent)((p_isn p_re)(ca_wa (p_csn cp_ent)))(oa_ent u_isn) u_hdip))(u_isn oa_wa)))(u_isn oa_wa))	21	14
8429	(((cp_ent ca_ent))))(ca_wa p_re) ca_wa)(cp_ent oa_ent) u_hdip))(u_isn u_hdip))(u_isn u_hdip)))(u_isn u_hdip))	23	14
8430	(((cp_ent ca_wa))))(p_re ca_wa)(cp_ent oa_ent))(u_hdip u_hdip))(u_isn u_hdip))(u_isn oa_wa)))(u_csn cp_ent))	24	14
8431	(((p_isn ca_ent))((p_isn p_re))(ca_wa p_csn)(cp_ent oa_ent))(u_isn u_hdip))(u_isn oa_wa)))(u_isn oa_wa))	19	14
8432	(((p_isn ca_ent))((ca_wa p_re)(ca_wa (p_csn cp_ent)))(oa_ent u_isn) u_hdip))(u_isn oa_wa)))(u_isn oa_wa))	17	14
8433	(((p_isn ca_ent))((p_isn p_re)(ca_wa (p_csn cp_ent)))(oa_ent u_isn) u_hdip))(u_isn oa_wa)))(u_isn oa_wa))	18	14
8434	(((p_isn ca_ent))))(ca_wa p_re) ca_wa)((cp_ent oa_ent) oa_wa))(u_isn u_hdip)))(u_isn u_hdip))(u_isn oa_wa))	23	14
8435	(((p_isn ca_ent))))(ca_wa p_re) ca_wa)((cp_ent oa_ent) oa_wa))(u_isn u_hdip)))(u_isn u_hdip))(u_isn u_hdip))	20	14
8436	(((p_isn ca_ent))))(ca_wa p_re) ca_wa)(cp_ent oa_ent))(u_isn u_hdip))(u_isn oa_wa)))(u_isn oa_wa) u_hdip))	23	14
8437	(((p_isn ca_ent))))(ca_wa p_re) cp_ent)((oa_ent u_isn) u_hdip))(u_isn oa_wa))(u_isn oa_wa)))(u_isn u_hdip))	22	14
8438	(((p_isn ca_ent))))(p_re ca_wa)(cp_ent oa_ent))(oa_wa u_hdip))(u_isn u_hdip))(u_isn u_hdip)))(u_isn oa_wa))	21	14
8439	(((p_isn ca_wa))))(p_re ca_wa)(cp_ent oa_ent))(u_hdip u_hdip))(u_isn u_hdip))(oa_wa u_csn)))(u_isn cp_ent))	24	14
8440	((((u_csn (cp_ent ca_ent))((ca_wa p_re) ca_wa)(cp_ent oa_ent)))(u_isn u_hdip))(u_isn u_hdip))(u_isn u_hdip))	26	14
8441	((((oa_wa (u_csn cp_ent)) ca_ent))))(ca_wa p_re) cp_ent) oa_ent)(u_isn u_hdip))(u_isn u_hdip)))(u_isn u_hdip))	24	14
8442	(((u_csn cp_ent)(p_isn ca_ent))))(ca_wa p_re) cp_ent) oa_ent)(u_isn u_hdip))(u_isn u_hdip)))(u_isn u_hdip))	23	14
8443	(((u_hdip u_csn)(cp_ent ca_ent))))(ca_wa p_re) cp_ent) oa_ent)(u_isn u_hdip))(u_isn u_hdip)))(u_isn u_hdip))	18	14
8444	((((ca_ent ca_wa))((p_re ca_wa))(cp_ent oa_ent) oa_wa))(u_isn oa_wa)))(u_hdip (u_isn u_csn))(u_isn cp_ent))	17	14
8445	((((ca_ent ca_wa))((p_re ca_wa)(p_csn oa_ent))(u_hdip u_hdip))(u_isn u_hdip)))(u_isn u_hdip))(u_csn cp_ent))	23	14
8446	((((ca_ent p_isn))((p_re ca_wa)((cp_ent oa_ent)(oa_wa u_hdip)))(u_isn u_hdip))(u_isn u_hdip))(u_isn oa_wa))	28	14
8447	((((ca_ent p_isn))((p_re ca_wa)(cp_ent oa_ent))(oa_wa u_hdip))(u_isn u_hdip))(u_isn u_hdip))(u_isn oa_wa))	28	14
8448	((((ca_ent p_isn))((p_re ca_wa)(cp_ent oa_ent))(u_hdip u_hdip))(u_isn u_hdip)))(u_isn oa_wa))(u_csn cp_ent))	26	14
8449	((((ca_ent p_isn))((p_re ca_wa)(cp_ent oa_ent))(u_hdip u_hdip))(u_isn u_hdip))(u_isn u_hdip))(u_csn cp_ent))	28	14
8450	((((cp_ent ca_ent))((ca_wa p_re) ca_wa)((cp_ent oa_ent) oa_wa)))(u_isn u_hdip))(u_isn u_hdip))(u_isn oa_wa))	25	14
8451	((((cp_ent ca_ent))((ca_wa p_re) ca_wa)((cp_ent oa_ent) u_hdip)))(u_isn u_hdip))(u_isn oa_wa))(u_csn cp_ent))	23	14
8452	((((cp_ent ca_ent))((ca_wa p_re) ca_wa)((cp_ent oa_ent) oa_wa))(u_isn u_hdip))(u_isn u_hdip))(u_isn oa_wa))	24	14
8453	((((cp_ent ca_ent))((ca_wa p_re) cp_ent)((oa_ent u_isn) u_hdip))(u_isn u_hdip)))(u_isn u_hdip))(u_isn oa_wa))	25	14
8454	((((cp_ent ca_ent))((ca_wa p_re) cp_ent)((oa_ent u_isn) u_hdip))(u_isn u_hdip)))(u_isn u_hdip))(u_isn u_hdip))	21	14
8455	((((cp_ent ca_wa))((p_re ca_wa)(cp_ent oa_ent))(oa_wa u_hdip))(u_isn u_hdip)))(u_isn oa_wa))(u_csn cp_ent))	21	14
8456	((((cp_ent ca_wa))((p_re ca_wa)(cp_ent oa_ent))(oa_wa u_hdip))(u_isn u_hdip))(u_isn u_hdip))(u_isn oa_wa))	22	14
8457	((((cp_ent ca_wa))((p_re ca_wa)(cp_ent oa_ent))(u_hdip u_hdip))(u_isn u_hdip)))(u_isn u_hdip))(u_csn cp_ent))	21	14
8458	((((cp_ent ca_wa))((p_re ca_wa)(cp_ent oa_ent))(u_hdip u_hdip))(u_isn u_hdip)))(u_isn u_hdip))(u_isn oa_wa))	21	14
8459	((((p_isn ca_ent))((ca_wa p_re) ca_wa)((cp_ent oa_ent) oa_wa))(u_hdip u_hdip))(u_isn oa_wa))(u_csn cp_ent))	19	14
8460	(((p_isn ca_ent))((ca_wa p_re) ca_wa)((p_csn oa_ent) oa_wa))(u_isn u_hdip)))(u_isn u_hdip))(u_isn u_hdip))	18	14
8461	(((p_isn ca_ent))((ca_wa p_re) cp_ent) oa_ent)(u_isn u_hdip))(u_isn u_hdip)))(u_isn u_hdip))(u_isn oa_wa))	25	14
8462	((((p_isn ca_ent))((ca_wa p_re) cp_ent)(oa_ent (oa_wa u_hdip)))(u_isn u_hdip)))(u_isn oa_wa))(u_csn cp_ent))	18	14
8463	((((p_isn ca_ent))((ca_wa p_re) cp_ent)(oa_ent (oa_wa u_hdip)))(u_isn u_hdip)))(u_isn u_hdip))(u_isn oa_wa))	17	14
8464	((((p_isn ca_ent))((ca_wa p_re) cp_ent)(oa_ent (u_isn u_hdip)))(u_isn u_hdip)))(u_isn u_hdip))(u_isn oa_wa))	23	14
8465	((((p_isn ca_ent))((p_re ca_wa)(cp_ent oa_ent))(oa_wa u_hdip))(u_isn u_hdip)))(u_isn oa_wa))(u_csn cp_ent))	20	14
8466	(((p_isn ca_wa))((p_re ca_wa)(p_csn oa_ent))(u_isn u_hdip))(u_isn u_hdip)))(u_isn u_hdip))(u_isn u_hdip))	22	14
8467	(((p_isn ca_wa))((p_re ca_wa) p_csn)(oa_ent (u_isn u_hdip)))(u_isn u_hdip)))(u_isn u_hdip))(u_isn u_hdip))	22	14
8468	(((p_isn ca_wa))((p_re ca_wa)(cp_ent oa_ent))(oa_wa u_hdip))(u_isn u_hdip))(u_isn u_hdip))(u_isn oa_wa))	27	14
8469	(((p_isn ca_wa))((p_re ca_wa)(cp_ent oa_ent))(oa_wa u_hdip))(u_isn u_hdip))(u_isn u_hdip))(u_isn u_hdip))	19	14
8470	(((ca_ent p_isn))((p_re ca_wa)(cp_ent oa_ent))(u_isn u_hdip))(u_isn u_hdip)))(u_isn u_hdip))(u_isn oa_wa))	27	14
8471	(((p_isn ca_wa))((p_re ca_wa)(cp_ent oa_ent))(u_isn u_hdip))(u_isn u_hdip))(u_isn u_hdip))(u_isn oa_wa))	27	14
8472	(((ca_ent p_isn))((p_re ca_wa)(cp_ent oa_ent))(u_isn u_hdip))(u_isn u_hdip)))(u_isn u_hdip))(u_isn u_hdip))	24	14
8473	(((p_isn ca_wa))((p_re ca_wa)(cp_ent oa_ent))(u_isn u_hdip))(u_isn u_hdip))(u_isn u_hdip))(u_isn u_hdip))	24	14
8474	((((p_isn p_re))((ca_wa p_csn)(oa_ent u_isn))(u_hdip u_hdip)))(u_isn u_hdip))(u_isn u_hdip))(u_isn u_csn))	17	14
8475	((((cp_ent ca_ent))((ca_wa p_re) ca_wa)((cp_ent oa_ent) oa_wa)))(u_hdip u_hdip))(u_isn oa_wa))(u_csn cp_ent))	22	14
8476	((((cp_ent ca_ent))((ca_wa p_re) ca_wa)((cp_ent oa_ent) oa_wa))(u_hdip u_hdip))(u_isn u_hdip))(u_isn oa_wa))	24	14
8477	((((cp_ent ca_ent))((ca_wa p_re) ca_wa)(cp_ent oa_ent)))((oa_wa u_hdip)(u_isn oa_wa)))(u_csn cp_ent)) oa_ent)	18	14
8478	((((cp_ent ca_ent))((ca_wa p_re) cp_ent)(oa_ent (oa_wa u_hdip))))(u_isn u_hdip))(u_isn u_hdip))(u_isn oa_wa))	22	14
8479	((((cp_ent ca_ent))((ca_wa p_re) cp_ent)(oa_ent (u_isn u_hdip))))(u_isn oa_wa))(u_isn oa_wa))(u_isn u_hdip))	22	14
8480	((((cp_ent ca_ent))((ca_wa p_re) cp_ent)((oa_ent oa_wa) u_hdip)))(u_isn u_hdip))(u_isn u_hdip))(u_isn oa_wa))	20	14
8481	((((cp_ent ca_ent))((p_re ca_wa)(cp_ent oa_ent))(oa_wa u_hdip)))(u_isn u_hdip))(u_isn oa_wa))(u_csn cp_ent))	22	14
8482	((((cp_ent ca_ent))((p_re ca_wa)(cp_ent oa_ent))(oa_wa u_hdip)))(u_isn u_hdip))(u_isn u_hdip))(u_isn oa_wa))	24	14
8483	((((cp_ent ca_wa))((p_re ca_wa)((cp_ent oa_ent)(oa_wa u_hdip))))(u_isn u_hdip))(u_isn u_hdip))(u_isn oa_wa))	22	14
8484	((((p_isn ca_ent))((ca_wa ca_wa)(cp_ent oa_ent))(u_hdip u_hdip)))(u_isn u_hdip))(u_isn oa_wa))(u_csn cp_ent))	20	14
8485	((((p_isn ca_ent))((ca_wa p_re) ca_wa)(cp_ent oa_ent))(u_isn u_hdip)(u_isn oa_wa)))(u_isn oa_wa) u_hdip))	24	14
8486	((((u_csn cp_ent))((ca_ent ca_wa) cp_ent)(oa_ent (u_isn u_hdip))))(u_isn u_hdip))(u_isn u_hdip))(u_isn oa_wa))	20	14
8487	((((u_csn (cp_ent ca_wa))(p_re ca_wa)((cp_ent oa_ent) u_hdip)))(u_isn u_hdip))(u_isn u_hdip))(u_isn oa_wa))	21	14
8488	((((p_csn (oa_ent (u_isn u_hdip)))((u_isn oa_wa) u_hdip))((u_csn cp_ent) p_csn))(ca_ent ca_wa))(p_re ca_wa))	21	14
8489	((((cp_ent ca_ent))((ca_wa p_re) cp_ent)) oa_ent)((oa_wa u_hdip)(u_isn oa_wa))(u_csn cp_ent))(oa_ent u_isn))	19	14
8490	((((cp_ent ca_ent))((ca_wa p_re) cp_ent))(oa_ent oa_wa))(u_isn oa_wa))(u_hdip (u_isn u_csn))(u_isn cp_ent))	16	14
8491	((((cp_ent ca_ent))((ca_wa p_re) cp_ent))(oa_ent (oa_wa u_hdip)))(u_isn u_hdip))(u_isn oa_wa))(u_csn cp_ent))	21	14
8492	((((cp_ent ca_ent))((ca_wa p_re) cp_ent))(oa_ent (oa_wa u_hdip)))(u_isn u_hdip))(u_isn u_hdip))(u_isn oa_wa))	23	14
8493	((((cp_ent ca_ent))((ca_wa p_re) cp_ent))(oa_ent (u_isn u_hdip)))(u_isn oa_wa))(u_isn oa_wa))(u_isn u_hdip))	23	14
8494	((((cp_ent ca_ent))((ca_wa p_re) cp_ent))((oa_ent oa_wa) u_hdip))(u_isn u_hdip))(u_isn u_hdip))(u_isn oa_wa))	21	14
8495	((((cp_ent ca_ent))((ca_wa p_re) cp_ent))((oa_ent u_isn) u_hdip))(u_isn u_hdip))(u_isn u_hdip))(u_isn oa_wa))	26	14
8496	((((cp_ent ca_ent))((ca_wa p_re) cp_ent))((oa_ent u_isn) u_hdip))(u_isn u_hdip))(u_isn u_hdip))(u_isn u_hdip))	22	14
8497	((((((ca_wa p_re) ca_wa)(p_csn (oa_ent u_isn)))(u_hdip u_hdip))(u_isn u_hdip))(u_isn u_hdip))(u_csn cp_ent))	22	14
8498	((((((ca_wa p_re) ca_wa)((cp_ent oa_ent) u_hdip))(u_isn u_hdip))(u_isn u_hdip))(u_isn oa_wa))(u_csn cp_ent))	26	14
8499	((((((ca_wa p_re) ca_wa)((cp_ent oa_ent) u_hdip))(u_isn u_hdip))(u_isn u_hdip))(u_isn u_hdip))(u_csn cp_ent))	27	14
8500	((((((ca_wa p_re) ca_wa)((cp_ent oa_ent) u_hdip))(u_isn u_hdip))(u_isn u_hdip))(u_isn u_hdip))(u_isn oa_wa))	25	14
8501	(((((u_csn (cp_ent ca_ent))((ca_wa p_re) ca_wa))(cp_ent oa_ent))(u_isn u_hdip))(u_isn u_hdip))(u_isn u_hdip))	21	14
8502	(((((u_csn (p_isn ca_ent))(ca_wa p_re))(p_isn ca_wa)))(cp_ent oa_ent) u_hdip))(u_isn u_hdip))(u_isn u_hdip))	13	14
8503	(((((u_hdip (u_csn cp_ent))(p_isn ca_wa))(p_isn ca_wa)))(oa_ent u_isn) u_hdip))(u_isn u_hdip))(u_isn u_hdip))	19	14
8504	(((((ca_ent ca_wa) cp_ent))(oa_ent u_isn) u_hdip))(u_isn u_hdip))(u_isn u_hdip))(u_isn oa_wa))(u_csn cp_ent))	24	14
8505	(((((ca_ent p_re)(cp_ent oa_ent))(u_hdip u_hdip))(u_isn u_hdip))(u_isn u_hdip))(u_isn oa_wa))(u_csn cp_ent))	17	14
8506	(((((ca_wa ca_wa)(cp_ent oa_ent))(u_hdip u_hdip))(u_isn u_hdip))(u_isn u_hdip))(u_isn oa_wa))(u_csn cp_ent))	20	14
8507	(((((ca_wa p_re) ca_wa)((cp_ent oa_ent) oa_wa))(u_hdip u_hdip))(u_isn u_hdip))(u_isn oa_wa))(u_csn cp_ent))	22	14
8508	(((((cp_ent ca_ent) cp_ent)(oa_ent (u_isn u_hdip)))(u_isn u_hdip))(u_isn u_hdip))(u_isn u_hdip))(u_isn oa_wa))	19	14
8509	(((((cp_ent ca_ent) cp_ent)((oa_ent u_isn) u_hdip))(u_isn u_hdip))(u_isn u_hdip))(u_isn u_hdip))(u_csn cp_ent))	21	14
8510	(((((cp_ent ca_ent)(ca_wa p_re))(cp_ent oa_ent))(oa_wa u_hdip))(u_isn u_hdip))(u_isn u_hdip))(u_isn oa_wa))	22	14
8511	(((((cp_ent ca_ent)(ca_wa p_re))(cp_ent oa_ent))(u_hdip u_hdip))(u_isn u_hdip))(u_isn u_hdip))(u_isn oa_wa))	21	14
8512	(((((cp_ent ca_wa) cp_ent)((oa_ent u_isn) u_hdip))(u_isn u_hdip))(u_isn u_hdip))(u_isn oa_wa))(u_csn cp_ent))	20	14
8513	(((((cp_ent ca_wa) cp_ent)((oa_ent u_isn) u_hdip))(u_isn u_hdip))(u_isn u_hdip))(u_isn u_hdip))(u_isn oa_wa))	18	14
8514	(((((p_csn oa_ent)(u_isn u_hdip))(u_isn u_hdip))(u_isn oa_wa)))(u_csn cp_ent) p_csn)) ca_ent)(ca_wa ca_wa))	22	14
8515	(((((p_isn ca_ent)(ca_wa p_re))(cp_ent oa_ent))(u_hdip u_hdip))(u_isn u_hdip))(u_isn oa_wa))(u_csn cp_ent))	22	14
8516	(((((p_re ca_wa)(cp_ent oa_ent))(oa_wa u_hdip))(u_isn u_hdip))(u_isn u_hdip))(u_isn oa_wa))(u_csn cp_ent))	21	14
8517	(((((p_re ca_wa)(cp_ent oa_ent))(u_hdip u_hdip))(u_isn u_hdip))(u_isn u_hdip))(u_isn oa_wa))(u_csn cp_ent))	23	14
8518	(((((u_isn oa_wa) u_hdip)(oa_wa u_csn)) oa_ent))(oa_wa u_hdip) oa_wa))(cp_ent ca_ent)))(ca_wa p_re) cp_ent))	19	14
8519	((((p_isn ca_ent))))(ca_wa p_re) ca_wa) oa_ent)(oa_wa u_hdip))(u_isn u_hdip)))(u_isn u_hdip))(oa_wa u_csn))	17	14
8520	((((p_isn ca_ent))))(ca_wa p_re) cp_ent) oa_ent)(oa_wa u_hdip))(u_isn u_hdip)))(u_isn u_hdip))(u_isn oa_wa))	18	14
8521	((((p_isn ca_wa))))(p_re ca_wa) p_csn) oa_ent)(u_isn u_hdip))(u_isn u_hdip)))(u_isn u_hdip))(u_isn u_hdip))	23	14
8522	((((cp_ent ca_ent))))(ca_wa p_re) ca_wa) oa_ent)(u_hdip u_hdip)))(u_isn u_hdip))(u_isn oa_wa))(u_csn cp_ent))	23	14
8523	((((cp_ent ca_ent))))(ca_wa p_re) ca_wa) oa_ent)(u_hdip u_hdip)))(u_isn u_hdip))(u_isn u_hdip))(u_csn cp_ent))	20	14
8524	((((cp_ent ca_ent))))(ca_wa p_re) ca_wa) oa_ent)(u_hdip u_hdip)))(u_isn u_hdip))(u_isn u_hdip))(u_isn oa_wa))	19	14
8525	((((cp_ent ca_ent))))(ca_wa p_re) cp_ent) oa_ent)(oa_wa u_hdip)))(u_isn u_hdip))(u_isn u_hdip))(u_isn oa_wa))	23	14
8526	((((cp_ent ca_ent))))(ca_wa p_re) cp_ent) oa_ent)(u_hdip u_hdip)))(u_isn u_hdip))(u_isn oa_wa))(u_csn cp_ent))	25	14
8527	((((cp_ent ca_ent))))(ca_wa p_re) cp_ent) oa_ent)(u_hdip u_hdip)))(u_isn u_hdip))(u_isn u_hdip))(u_csn cp_ent))	25	14
8528	((((cp_ent ca_ent))))(ca_wa p_re) cp_ent) oa_ent)(u_hdip u_hdip)))(u_isn u_hdip))(u_isn u_hdip))(u_isn oa_wa))	24	14
8529	((((p_isn ca_ent))))(ca_wa p_re) cp_ent) oa_ent)(oa_wa u_hdip)))(u_isn u_hdip))(u_isn oa_wa))(u_csn cp_ent))	19	14
8530	((((p_isn ca_ent))))(ca_wa p_re) cp_ent) oa_ent)(oa_wa u_hdip))(u_isn u_hdip))(u_isn u_hdip))(u_isn oa_wa))	17	14
8531	((((p_isn ca_ent))))(ca_wa p_re) cp_ent) oa_ent)(u_isn u_hdip)))(u_isn oa_wa))(u_isn oa_wa))(u_isn u_hdip))	20	14
8532	((((cp_ent ca_ent))))(ca_wa p_re) ca_wa) oa_ent))(oa_wa u_hdip))(u_isn u_hdip))(u_isn u_hdip))(u_isn oa_wa))	19	14
8533	((((cp_ent ca_ent))))(ca_wa p_re) ca_wa) oa_ent)(u_hdip u_hdip)))(u_isn u_hdip))(u_isn u_hdip))(u_csn cp_ent))	21	14
8534	((((cp_ent ca_ent))))(ca_wa p_re) ca_wa) oa_ent))(u_hdip u_hdip))(u_isn u_hdip))(u_isn u_hdip))(u_isn oa_wa))	20	14
8535	((((p_isn ca_ent))))(ca_wa p_re) cp_ent) oa_ent))(oa_wa u_hdip))(u_isn u_hdip))(u_isn oa_wa))(u_csn cp_ent))	19	14
8536	(((((cp_ent ca_ent))((ca_wa p_re) cp_ent)) oa_ent)(oa_wa u_hdip))(u_isn u_hdip))(u_isn u_hdip))(u_isn oa_wa))	24	14
8537	(((((cp_ent ca_ent))((ca_wa p_re) cp_ent)) oa_ent)(u_hdip u_hdip))(u_isn u_hdip))(u_isn oa_wa))(u_csn cp_ent))	26	14
8538	(((((cp_ent ca_ent))((ca_wa p_re) cp_ent)) oa_ent)(u_hdip u_hdip))(u_isn u_hdip))(u_isn u_hdip))(u_csn cp_ent))	26	14
8539	(((((cp_ent ca_ent))((ca_wa p_re) cp_ent)) oa_ent)(u_hdip u_hdip))(u_isn u_hdip))(u_isn u_hdip))(u_isn oa_wa))	25	14
8540	((((((ca_wa p_re) ca_wa) oa_ent)(oa_wa u_hdip))(u_isn u_hdip))(u_isn u_hdip))(u_isn oa_wa))(u_csn cp_ent))	21	14
8541	((((((ca_wa p_re) ca_wa) oa_ent)(u_hdip u_hdip))(u_isn u_hdip))(u_isn u_hdip))(u_isn oa_wa))(u_csn cp_ent))	22	14
8542	((((((cp_ent ca_ent) cp_ent) oa_ent)(u_hdip u_hdip))(u_isn u_hdip))(u_isn u_hdip))(u_isn oa_wa))(u_csn cp_ent))	21	14
8543	((((((cp_ent ca_wa) cp_ent) oa_ent)(u_hdip u_hdip))(u_isn u_hdip))(u_isn u_hdip))(u_isn oa_wa))(u_csn cp_ent))	21	14

8544	((u_hdip u_hdip))(((u_isn oa_wa))(u_csn cp_ent) p_csn)) ca_ent))((((ca_wa p_re) ca_wa)(cp_ent oa_ent))(u_hdip u_hdip)))	18	15
8545	((u_isn u_hdip))(((u_isn oa_wa))(u_csn cp_ent) p_csn)) ca_ent))(((ca_wa p_re) ca_wa)(cp_ent oa_ent))(u_hdip u_hdip))))	17	15
8546	(((u_hdip u_hdip))(oa_wa (u_csn cp_ent)) p_csn)(ca_ent ca_wa))))((p_re ca_wa)((cp_ent oa_ent) u_hdip))(u_isn u_hdip)))	18	15
8547	(((u_hdip (u_isn oa_wa))((u_csn cp_ent) p_csn)(ca_ent ca_wa))))((p_re ca_wa)((cp_ent oa_ent) u_hdip))(u_isn u_hdip)))	18	15
8548	(((u_hdip u_hdip)(oa_wa (u_csn cp_ent))(p_csn ca_ent)))((((ca_wa p_re) cp_ent)(oa_ent u_isn) u_hdip))(u_isn u_hdip)))	19	15
8549	(((u_hdip (u_isn oa_wa))(((u_csn cp_ent) p_csn) ca_ent))))((ca_wa p_re) cp_ent)(oa_ent u_isn) u_hdip))(u_isn u_hdip)))	19	15
8550	(((u_hdip (u_isn oa_wa))(((u_csn cp_ent) p_csn) ca_ent)))))(ca_wa p_re) cp_ent) oa_ent)(u_hdip u_hdip))(u_isn u_hdip)))	17	15
8551	((((oa_wa (u_csn cp_ent))(p_isn ca_ent))((ca_wa p_re) ca_wa)(p_csn ((p_isn oa_ent)(u_isn u_hdip))))(u_isn u_hdip))	16	15
8552	(((u_hdip (u_isn oa_wa))(u_csn (cp_ent ca_ent))(((((ca_wa p_re) ca_wa)(cp_ent oa_ent))(oa_wa u_hdip))(u_isn u_hdip)))	17	15
8553	(((u_hdip (u_isn oa_wa))(u_csn (cp_ent ca_ent)))((((ca_wa p_re) ca_wa)(cp_ent oa_ent))(u_hdip u_hdip))(u_isn u_hdip)))	21	15
8554	((((u_hdip oa_wa) u_csn)(cp_ent ca_ent))((ca_wa p_re) ca_wa)(p_csn ((p_isn oa_ent)(u_isn u_hdip))))(u_isn u_hdip))	17	15
8555	(((u_isn oa_wa)((u_csn cp_ent) p_csn)) ca_ent))))(ca_wa p_re) ca_wa)(cp_ent oa_ent))(u_hdip u_hdip))(u_isn u_hdip)))	18	15
8556	((((u_isn oa_wa) u_hdip)(u_csn (cp_ent ca_ent)))))(ca_wa p_re) ca_wa)(cp_ent oa_ent))(u_hdip u_hdip))(u_isn u_hdip)))	19	15
8557	(((u_isn oa_wa) u_hdip)(((u_csn cp_ent)(p_csn ca_ent)))))(ca_wa p_re) ca_wa)((cp_ent oa_ent) u_hdip))(u_isn u_hdip)))	18	15
8558	(((u_isn u_hdip)(oa_wa (u_csn cp_ent))(p_csn ca_ent))))(ca_wa p_re) cp_ent)((oa_ent u_isn) u_hdip))(u_isn u_hdip)))	17	15
8559	(((p_isn ca_ent))((p_re ca_wa)(cp_ent oa_ent))(oa_wa u_hdip))(u_isn u_hdip)))(u_isn oa_wa))(u_csn cp_ent) oa_ent))	19	15
8560	(((u_hdip u_hdip))((u_isn oa_wa) u_csn)(cp_ent ca_ent))(((((ca_wa p_re) ca_wa)((cp_ent oa_ent) oa_wa))(u_isn u_hdip)))	19	15
8561	(((u_hdip u_hdip))((u_isn oa_wa) u_csn)(cp_ent ca_ent))))((ca_wa p_re) ca_wa))(cp_ent oa_ent) u_hdip))(u_isn u_hdip)))	22	15
8562	(((u_hdip u_hdip))((u_isn oa_wa) u_csn)(cp_ent ca_ent))))((ca_wa p_re) cp_ent))(oa_ent u_isn) u_hdip))(u_isn u_hdip)))	23	15
8563	(((u_hdip u_hdip))((u_isn oa_wa) u_csn)(p_csn (ca_ent ca_wa))))((p_re ca_wa))(cp_ent oa_ent) u_hdip))(u_isn u_hdip)))	17	15
8564	(((u_isn oa_wa) u_hdip))((u_csn cp_ent) p_csn)(p_isn ca_ent))))((ca_wa p_re) cp_ent)(oa_ent u_hdip))(u_isn u_hdip)))	14	15
8565	((u_isn u_hdip))((u_isn oa_wa) u_csn)(cp_ent ca_ent)))))(ca_wa p_re) ca_wa)(cp_ent oa_ent) u_hdip))(u_isn u_hdip)))	18	15
8566	((u_isn u_hdip))((u_isn oa_wa) u_csn)(cp_ent ca_ent))))((ca_wa p_re) cp_ent)((oa_ent u_isn) u_hdip))(u_isn u_hdip)))	19	15
8567	(((u_hdip (u_isn oa_wa))((u_csn cp_ent) p_csn)(p_isn ca_wa)))((p_re ca_wa)(cp_ent oa_ent) u_hdip))(u_isn u_hdip)))	15	15
8568	(((u_hdip u_hdip)(oa_wa (u_csn cp_ent)))(p_csn (ca_ent ca_wa))))((p_re ca_wa)((cp_ent oa_ent) u_hdip))(u_isn u_hdip)))	17	15
8569	(((u_hdip u_hdip)(oa_wa (u_csn cp_ent)))(p_csn ca_ent)))((ca_wa p_re) cp_ent)(oa_ent u_isn) u_hdip))(u_isn u_hdip)))	18	15
8570	(((u_isn u_hdip)(u_isn oa_wa))(u_csn cp_ent) p_csn)))(ca_ent ca_wa))))(p_re ca_wa)(cp_ent oa_ent))(u_hdip u_hdip)))	18	15
8571	(((u_hdip u_hdip)(oa_wa (u_csn cp_ent)))(p_csn (p_isn ca_wa))))((p_re ca_wa)(cp_ent oa_ent) u_hdip))(u_isn u_hdip)))	14	15
8572	((((u_isn oa_wa))(u_csn cp_ent) p_csn))(p_isn ca_ent))))(ca_wa p_re) cp_ent)(oa_ent u_isn) u_hdip)))(u_isn u_hdip))	15	15
8573	(((u_isn u_hdip)(u_isn oa_wa) u_csn))(cp_ent ca_ent))))(ca_wa p_re) ca_wa)(cp_ent oa_ent) u_hdip))(u_isn u_hdip)))	19	15
8574	(((u_isn u_hdip)(u_isn oa_wa) u_csn))(cp_ent ca_ent))))(ca_wa p_re) cp_ent)(oa_ent u_isn) u_hdip))(u_isn u_hdip)))	20	15
8575	((((u_hdip (u_isn oa_wa)) u_csn)(ca_ent ca_wa)))))(p_re ca_wa) p_csn)(p_isn oa_ent))(u_isn u_hdip))(u_isn u_hdip)))	16	15
8576	(((u_hdip (u_isn oa_wa)) u_csn)(cp_ent ca_ent)))))(ca_wa p_re) ca_wa)(cp_ent oa_ent))(u_hdip u_hdip))(u_isn u_hdip)))	22	15
8577	(((u_hdip (u_isn oa_wa)) u_csn)(cp_ent ca_ent))(((ca_wa p_re) ca_wa)(cp_ent oa_ent))(u_isn u_hdip))(u_isn u_hdip)))	22	15
8578	((oa_wa u_hdip))((u_csn cp_ent)(p_csn (p_isn ca_ent))))(ca_wa p_re) cp_ent)(oa_ent u_isn) u_hdip))(u_isn u_hdip)))	16	15
8579	((oa_wa u_hdip))((u_isn oa_wa))(u_csn cp_ent) p_csn)(p_isn ca_wa)))((p_re ca_wa)(cp_ent oa_ent))(u_hdip u_hdip)))	14	15
8580	((u_hdip oa_wa))(((u_csn cp_ent) p_csn)(ca_ent ca_wa))))((p_re ca_wa)(cp_ent oa_ent))(u_hdip u_hdip))(u_isn u_hdip)))	16	15
8581	((u_hdip u_hdip))((u_isn oa_wa) u_csn)(cp_ent ca_ent))))(ca_wa p_re) ca_wa)(cp_ent oa_ent) oa_wa))(u_isn u_hdip)))	18	15
8582	((u_hdip u_hdip))((u_isn oa_wa) u_csn)(cp_ent ca_ent))))(ca_wa p_re) ca_wa)(cp_ent oa_ent) u_hdip))(u_isn u_hdip)))	21	15
8583	((u_hdip u_hdip))(((u_isn oa_wa) u_csn)(cp_ent ca_ent))))(ca_wa p_re) cp_ent)(oa_ent u_isn) u_hdip))(u_isn u_hdip)))	21	15
8584	((u_hdip u_hdip))(((u_isn oa_wa))(u_csn cp_ent) p_csn) ca_ent))((ca_wa p_re) ca_wa)(oa_ent u_isn))(u_hdip u_hdip)))	16	15
8585	((u_hdip u_hdip))(((u_isn oa_wa))(u_csn cp_ent) p_csn)) ca_ent))))(ca_wa p_re) cp_ent)(oa_ent u_hdip))(u_isn u_hdip)))	18	15
8586	((u_hdip u_hdip))(((u_isn oa_wa))(u_csn cp_ent) p_csn)) ca_ent))))(ca_wa p_re) cp_ent)(oa_ent u_isn))(u_hdip u_hdip)))	18	15
8587	((u_isn oa_wa))(((u_csn cp_ent) p_csn)(ca_ent ca_wa))))(p_re ca_wa)(cp_ent oa_ent))(u_hdip u_hdip))(u_isn u_hdip)))	17	15
8588	((u_isn u_hdip))(((u_isn oa_wa) u_csn)(cp_ent ca_ent))))(ca_wa p_re) cp_ent)(oa_ent u_isn) u_hdip))(u_isn u_hdip)))	18	15
8589	((u_isn u_hdip))(((u_isn oa_wa))(u_csn cp_ent) p_csn)) ca_ent))))(ca_wa p_re) cp_ent)(oa_ent u_hdip))(u_isn u_hdip)))	17	15
8590	((u_isn u_hdip))(((u_isn oa_wa))(u_csn cp_ent) p_csn)) ca_ent))))(ca_wa p_re) cp_ent)(oa_ent u_isn))(u_hdip u_hdip)))	17	15
8591	((oa_wa (u_csn (cp_ent ca_ent)))))))(ca_wa p_re) ca_wa)(cp_ent oa_ent))(u_isn u_hdip))(u_isn oa_wa))(u_isn u_hdip)))	19	15
8592	((u_hdip (u_isn oa_wa))))(u_csn cp_ent) p_csn) ca_ent)))(ca_wa p_re) cp_ent)(oa_ent u_isn) u_hdip))(u_isn u_hdip)))	18	15
8593	(((oa_wa (u_csn cp_ent)) ca_ent))))(ca_wa p_re) ca_wa)(cp_ent oa_ent)(oa_wa u_hdip))(u_isn u_hdip))(u_isn u_hdip)))	18	15
8594	(((oa_wa (u_csn cp_ent)) ca_ent))))(ca_wa p_re) ca_wa)(cp_ent oa_ent))(u_hdip u_hdip))(u_isn u_hdip))(u_isn oa_wa)))	20	15
8595	(((oa_wa (u_csn cp_ent)) ca_ent))))(ca_wa p_re) ca_wa)(cp_ent oa_ent))(u_hdip u_hdip))(u_isn u_hdip))(u_isn u_hdip)))	19	15
8596	(((oa_wa (u_csn cp_ent))(ca_ent ca_wa))))(p_re ca_wa)(cp_ent oa_ent))(oa_wa u_hdip))(u_isn u_hdip))(u_isn u_hdip)))	18	15
8597	(((oa_wa (u_csn cp_ent))(p_csn ca_ent))))(ca_wa p_re) ca_wa)(cp_ent oa_ent) u_hdip))(u_isn u_hdip))(u_isn u_hdip)))	18	15
8598	(((oa_wa (u_csn cp_ent))(p_csn ca_ent))))(ca_wa p_re) cp_ent)(oa_ent u_isn) u_hdip))(u_isn u_hdip))(u_isn u_hdip)))	17	15
8599	(((u_hdip oa_wa) u_hdip))((u_csn cp_ent)(p_csn ca_ent))))(ca_wa p_re) ca_wa)(cp_ent oa_ent) u_hdip))(u_isn u_hdip)))	16	15
8600	(((u_hdip oa_wa) u_hdip))((u_csn cp_ent) p_csn) ca_ent))((ca_wa p_re) cp_ent)(oa_ent u_isn) u_hdip))(u_isn u_hdip)))	17	15
8601	(((u_hdip (u_csn cp_ent)) ca_ent))))(ca_wa p_re) ca_wa)(cp_ent oa_ent))(oa_wa u_hdip))(u_isn u_hdip))(u_isn u_hdip)))	17	15
8602	(((u_hdip (u_csn cp_ent))(ca_ent ca_wa))))(p_re ca_wa)(cp_ent oa_ent))(oa_wa u_hdip))(u_isn u_hdip))(u_isn u_hdip)))	17	15
8603	(((u_hdip (u_csn cp_ent)) ca_ent))))(ca_wa p_re) ca_wa)(cp_ent oa_ent))(u_hdip u_hdip))(u_isn u_hdip))(u_isn u_hdip)))	18	15
8604	(((oa_wa u_hdip))((u_csn cp_ent) p_csn) ca_ent)))))(ca_wa p_re) ca_wa)(oa_ent u_isn))(u_hdip u_hdip))(u_isn u_hdip)))	15	15
8605	(((oa_wa u_hdip))((u_csn cp_ent) p_csn) ca_ent)))))(ca_wa p_re) cp_ent)(oa_ent u_hdip))(u_isn u_hdip))(u_isn u_hdip)))	18	15
8606	(((oa_wa u_hdip))((u_csn cp_ent) p_csn) ca_ent)))))(ca_wa p_re) cp_ent)(oa_ent u_isn))(u_hdip u_hdip))(u_isn u_hdip)))	16	15
8607	(((u_csn cp_ent)(p_csn (p_isn ca_ent)))))(ca_wa p_re) ca_wa)((cp_ent oa_ent) u_hdip))(u_isn u_hdip))(u_isn u_hdip)))	16	15
8608	(((u_hdip oa_wa))(u_csn cp_ent)(ca_ent ca_wa))))((p_re ca_wa)(cp_ent oa_ent) oa_wa))(u_hdip u_hdip))(u_isn u_hdip)))	17	15
8609	(((u_hdip oa_wa))(u_csn cp_ent)(ca_ent ca_wa))))((p_re ca_wa)((cp_ent oa_ent) oa_wa))(u_isn u_hdip))(u_isn u_hdip)))	18	15
8610	(((u_hdip oa_wa))(u_csn cp_ent)(ca_ent ca_wa))))((p_re ca_wa)((cp_ent oa_ent) u_hdip))(u_isn u_hdip))(u_isn u_hdip)))	21	15
8611	(((u_hdip oa_wa)(u_csn (cp_ent ca_ent)))))(ca_wa p_re) ca_wa)(cp_ent oa_ent) oa_wa))(u_hdip u_hdip))(u_isn u_hdip)))	18	15
8612	(((u_hdip oa_wa)(u_csn (cp_ent ca_ent)))))(ca_wa p_re) ca_wa)((cp_ent oa_ent) oa_wa))(u_isn u_hdip))(u_isn u_hdip)))	17	15
8613	(((u_hdip oa_wa)(u_csn (cp_ent ca_ent)))))(ca_wa p_re) cp_ent)(oa_ent u_isn) u_hdip))(u_isn u_hdip))(u_isn u_hdip)))	20	15
8614	(((u_hdip oa_wa)(u_csn (cp_ent ca_ent)))))(ca_wa p_re) cp_ent)(oa_ent (oa_wa u_hdip)))(u_isn u_hdip))(u_isn u_hdip)))	16	15
8615	(((u_hdip (u_isn oa_wa))((u_csn cp_ent)(p_csn ca_ent)))))(ca_wa p_re) ca_wa) oa_ent)(u_hdip u_hdip))(u_isn u_hdip)))	15	15
8616	((u_hdip oa_wa))(((u_csn cp_ent) p_csn) ca_ent))))(ca_wa p_re) cp_ent)(oa_ent u_hdip))(u_isn u_hdip))(u_isn u_hdip)))	17	15
8617	(((u_hdip u_hdip)(oa_wa (u_csn cp_ent)(p_csn ca_ent)))))(ca_wa p_re) cp_ent) oa_ent)(u_isn u_hdip))(u_isn u_hdip)))	18	15
8618	(((u_hdip (u_isn oa_wa))(((u_csn cp_ent) p_csn) ca_ent)))))(ca_wa p_re) cp_ent) oa_ent)(u_isn u_hdip))(u_isn u_hdip)))	18	15
8619	(((oa_wa (u_csn cp_ent))(p_isn ca_ent)))(ca_wa p_re) ca_wa)(p_csn ((p_isn oa_ent)(u_isn u_hdip))))(u_isn u_hdip)))	15	15
8620	(((oa_wa (u_csn cp_ent))(p_isn ca_ent))))(ca_wa p_re) cp_ent)(oa_ent u_isn) u_hdip))(u_isn u_hdip))(u_isn u_hdip)))	18	15
8621	(((oa_wa (u_csn cp_ent))(p_isn ca_wa))))(p_re ca_wa)(cp_ent oa_ent)(oa_wa u_hdip)))(u_isn u_hdip))(u_isn u_hdip)))	17	15
8622	(((oa_wa (u_csn cp_ent))(p_isn ca_wa))))(p_re ca_wa)(cp_ent oa_ent))(oa_wa u_hdip))(u_isn u_hdip))(u_isn u_hdip)))	17	15
8623	(((oa_wa (u_csn cp_ent))(p_isn ca_wa))))(p_re ca_wa)(cp_ent oa_ent))(u_isn u_hdip))(u_isn u_hdip))(u_isn oa_wa)))	17	15
8624	(((u_hdip (oa_wa u_csn))(cp_ent ca_ent)))))(ca_wa p_re) ca_wa)(cp_ent oa_ent) oa_wa))(u_hdip u_hdip))(u_isn u_hdip)))	16	15
8625	(((u_hdip (oa_wa u_csn))(cp_ent ca_ent)))))(ca_wa p_re) cp_ent)(oa_ent u_isn) u_hdip))(u_isn u_hdip))(u_isn u_hdip)))	18	15
8626	(((u_hdip (u_csn cp_ent))(p_csn (ca_ent ca_wa))))(p_re ca_wa)(cp_ent oa_ent) u_hdip))(u_isn u_hdip))(u_isn u_hdip)))	17	15
8627	(((u_hdip (u_csn cp_ent))(p_csn ca_ent))))(ca_wa p_re) cp_ent)(oa_ent u_isn) u_hdip))(u_isn u_hdip))(u_isn u_hdip)))	16	15
8628	(((u_hdip (u_csn cp_ent))(p_isn ca_ent)))))(ca_wa p_re) ca_wa)(cp_ent oa_ent) oa_wa))(u_hdip u_hdip))(u_isn u_hdip)))	15	15
8629	(((u_hdip (u_csn cp_ent))(p_isn ca_ent))))(ca_wa p_re) cp_ent)(oa_ent u_isn) u_hdip))(u_isn u_hdip))(u_isn u_hdip)))	18	15
8630	(((u_hdip (u_csn cp_ent))(p_isn ca_ent)))))(p_re ca_wa)(cp_ent oa_ent))(oa_wa u_hdip))(u_isn u_hdip))(u_isn u_hdip)))	15	15
8631	(((u_hdip (u_csn cp_ent))(p_isn ca_wa))))(p_re ca_wa)(cp_ent oa_ent)(oa_wa u_hdip)))(u_isn u_hdip))(u_isn u_hdip)))	17	15
8632	(((u_hdip (u_csn cp_ent))(p_isn ca_wa))))(p_re ca_wa)(cp_ent oa_ent))(oa_wa u_hdip))(u_isn u_hdip))(u_isn u_hdip)))	17	15
8633	(((u_hdip (u_csn cp_ent))(p_csn (p_isn ca_ent)))))(ca_wa p_re) ca_wa)(oa_ent u_isn))(u_hdip u_hdip))(u_isn u_hdip)))	13	15
8634	(((u_hdip (u_isn oa_wa))(u_csn (cp_ent ca_ent)))))(ca_wa p_re) cp_ent)(oa_ent u_hdip))(u_isn u_hdip))(u_isn u_hdip)))	21	15
8635	(((u_hdip (u_isn oa_wa))(u_csn (cp_ent ca_ent)))))(ca_wa p_re) cp_ent)(oa_ent u_isn))(u_hdip u_hdip))(u_isn u_hdip)))	19	15
8636	(((u_hdip (u_isn u_csn))(cp_ent ca_ent))))(ca_wa p_re) cp_ent)(oa_ent u_isn) u_hdip))(u_isn u_hdip))(u_isn u_hdip)))	18	15
8637	(((u_hdip (u_isn u_csn))(cp_ent ca_wa))))(p_re ca_wa)(cp_ent oa_ent))(u_hdip u_hdip))(u_isn u_hdip))(u_isn u_hdip)))	15	15
8638	(((u_csn cp_ent) p_csn)(p_isn ca_ent))))(ca_wa p_re) ca_wa)((cp_ent oa_ent) u_hdip))(u_isn u_hdip))(u_isn u_hdip)))	18	15
8639	(((u_hdip oa_wa) u_csn)(cp_ent ca_ent))))(ca_wa p_re) ca_wa)(p_csn (oa_ent u_isn))(u_hdip u_hdip))(u_isn u_hdip)))	17	15
8640	(((u_hdip oa_wa) u_csn)(cp_ent ca_ent))))(ca_wa p_re) ca_wa)((p_csn oa_ent) u_hdip))(u_isn u_hdip))(u_isn u_hdip)))	17	15
8641	(((u_isn oa_wa) u_csn)(cp_ent ca_ent)))))(ca_wa p_re) ca_wa)((cp_ent oa_ent) u_hdip))(u_isn u_hdip))(u_isn u_hdip)))	21	15
8642	(((u_isn oa_wa) u_csn)(ca_ent ca_wa))))(p_re ca_wa)(cp_ent oa_ent))(oa_wa u_hdip))(u_isn u_hdip))(u_isn u_hdip)))	17	15
8643	(((u_isn oa_wa) u_csn)(cp_ent ca_ent)))))(ca_wa p_re) ca_wa)((cp_ent oa_ent) oa_wa))(u_hdip u_hdip))(u_isn u_hdip)))	18	15
8644	(((u_isn oa_wa) u_csn)(cp_ent ca_ent)))))(ca_wa p_re) ca_wa)((cp_ent oa_ent) oa_wa))(u_isn u_hdip))(u_isn u_hdip)))	17	15
8645	(((u_isn oa_wa) u_csn)(cp_ent ca_ent)))))(ca_wa p_re) cp_ent)(oa_ent u_isn) u_hdip))(u_isn u_hdip))(u_isn u_hdip)))	20	15
8646	(((u_isn oa_wa) u_csn)(cp_ent ca_ent)))))(ca_wa p_re) cp_ent)(oa_ent (oa_wa u_hdip)))(u_isn u_hdip))(u_isn u_hdip)))	16	15
8647	(((u_isn oa_wa) u_hdip)(u_csn (cp_ent ca_ent)))))(ca_wa p_re) cp_ent)(oa_ent u_isn))(u_hdip u_hdip))(u_isn u_hdip)))	17	15
8648	(((u_isn oa_wa) u_hdip)((u_csn cp_ent)(p_csn ca_ent)))))(ca_wa p_re) ca_wa) oa_ent)(u_hdip u_hdip))(u_isn u_hdip)))	15	15
8649	(((u_hdip u_hdip))((u_isn oa_wa) u_csn)(cp_ent ca_ent)))))(ca_wa p_re) cp_ent) oa_ent)(u_isn u_hdip))(u_isn u_hdip)))	22	15
8650	((u_isn u_hdip))((u_isn oa_wa) u_csn)(cp_ent ca_ent)))))(ca_wa p_re) cp_ent) oa_ent)(u_isn u_hdip))(u_isn u_hdip)))	18	15
8651	(((u_isn oa_wa))(u_csn cp_ent) p_csn)) ca_ent))))(ca_wa p_re) cp_ent)(oa_ent u_hdip))(u_isn u_hdip))(u_isn u_hdip)))	18	15
8652	(((u_isn oa_wa))(u_csn cp_ent) p_csn)) ca_ent))))(ca_wa p_re) cp_ent)(oa_ent u_isn))(u_hdip u_hdip))(u_isn u_hdip)))	16	15
8653	(((u_hdip u_hdip)(oa_wa (u_csn cp_ent))(p_csn ca_ent)))))(ca_wa p_re) cp_ent) oa_ent)(u_isn u_hdip))(u_isn u_hdip)))	17	15
8654	((((u_isn oa_wa))(u_csn cp_ent) p_csn))(p_isn ca_ent)))))(ca_wa p_re) cp_ent) oa_ent)(u_isn u_hdip))(u_isn u_hdip)))	15	15
8655	(((u_isn u_hdip))(u_isn oa_wa) u_csn))(cp_ent ca_ent))))(ca_wa p_re) cp_ent) oa_ent)(u_isn u_hdip))(u_isn u_hdip)))	19	15
8656	((((u_hdip (u_isn oa_wa)) u_csn)(ca_ent ca_wa))(p_re ca_wa)(p_csn ((p_isn oa_ent)(u_isn u_hdip))))(u_isn u_hdip)))	15	15
8657	((((u_hdip (u_isn oa_wa)) u_csn)(cp_ent ca_ent))))(ca_wa p_re)(p_csn ((p_isn oa_ent)(u_isn u_hdip))))(u_isn u_hdip)))	16	15
8658	((((u_hdip (u_isn oa_wa)) u_csn)(cp_ent ca_ent))))(ca_wa p_re) ca_wa)(p_csn ((p_isn oa_ent) u_hdip))(u_isn u_hdip)))	15	15
8659	(((u_hdip (u_isn oa_wa)) u_csn)(cp_ent ca_wa))))(p_re ca_wa)(cp_ent oa_ent) oa_wa))(u_isn u_hdip))(u_isn u_hdip)))	18	15
8660	(((u_hdip (u_isn oa_wa)) u_csn)(ca_ent ca_wa))))(p_re ca_wa)((cp_ent oa_ent) u_hdip))(u_isn u_hdip))(u_isn u_hdip)))	21	15
8661	(((u_hdip (u_isn oa_wa)) u_csn)(cp_ent ca_ent))))(ca_wa p_re) ca_wa)(oa_ent u_isn))(u_hdip u_hdip))(u_isn u_hdip)))	17	15
8662	(((u_hdip (u_isn oa_wa)) u_csn)(cp_ent ca_ent)))))(ca_wa p_re) cp_ent)(oa_ent u_hdip))(u_isn u_hdip))(u_isn u_hdip)))	22	15
8663	(((u_hdip (u_isn oa_wa)) u_csn)(ca_ent ca_wa))))(p_re ca_wa) p_csn)(oa_ent u_isn))(u_hdip u_hdip))(u_isn u_hdip)))	17	15
8664	(((u_hdip (u_isn oa_wa)) u_csn)(cp_ent ca_wa))))(p_re ca_wa)((cp_ent oa_ent) u_hdip))(u_isn u_hdip))(u_isn u_hdip)))	19	15
8665	(((oa_wa u_hdip))((u_csn cp_ent)(p_csn (p_isn ca_ent))))(ca_wa p_re) cp_ent)(oa_ent u_isn) u_hdip)))(u_isn u_hdip)))	15	15
8666	((((u_hdip u_hdip)(u_isn oa_wa))(u_csn cp_ent) p_csn)))(ca_ent ca_wa)))(p_re ca_wa)(cp_ent oa_ent))(u_hdip u_hdip)))	19	15
8667	(((u_hdip u_hdip))((u_isn oa_wa))(u_csn cp_ent) p_csn)) ca_ent))((ca_wa p_re) ca_wa)(oa_ent u_isn))(u_hdip u_hdip)))	17	15
8668	(((u_hdip u_hdip))((u_isn oa_wa))(u_csn cp_ent) p_csn)) ca_ent))))(ca_wa p_re) cp_ent)(oa_ent u_hdip))(u_isn u_hdip)))	19	15
8669	(((u_hdip u_hdip))((u_isn oa_wa))(u_csn cp_ent) p_csn)) ca_ent))))(ca_wa p_re) cp_ent)(oa_ent u_isn))(u_hdip u_hdip)))	19	15
8670	(((u_hdip u_hdip))((oa_wa (u_csn cp_ent)) p_csn)(p_isn ca_ent))))(ca_wa p_re) ca_wa)(oa_ent u_isn))(u_hdip u_hdip)))	14	15
8671	(((u_hdip (u_isn oa_wa))(u_csn (cp_ent ca_ent))))(ca_wa p_re) ca_wa)(cp_ent oa_ent))(u_hdip u_hdip))(u_isn u_hdip)))	20	15
8672	((u_hdip u_hdip))(((u_isn oa_wa) u_csn)(cp_ent ca_ent))))(ca_wa p_re) cp_ent)(oa_ent u_isn) u_hdip))(u_isn u_hdip)))	22	15
8673	(((oa_wa (u_csn cp_ent))(ca_ent ca_wa))))(p_re ca_wa)(cp_ent oa_ent))(oa_wa u_hdip))(u_isn u_hdip))(u_isn u_hdip)))	19	15
8674	(((u_hdip oa_wa))(u_csn cp_ent)(ca_ent ca_wa))))(p_re ca_wa)((cp_ent oa_ent) u_hdip))(u_isn u_hdip))(u_isn u_hdip)))	20	15
8675	(((u_hdip oa_wa))(u_csn (cp_ent ca_ent))))(ca_wa p_re) cp_ent)(oa_ent u_isn) u_hdip))(u_isn u_hdip))(u_isn u_hdip)))	18	15
8676	(((oa_wa (u_csn cp_ent))(p_isn ca_wa))))(p_re ca_wa)(cp_ent oa_ent))(u_isn u_hdip))(u_isn u_hdip))(u_isn u_hdip)))	20	15
8677	(((u_hdip (oa_wa u_csn))(cp_ent ca_ent))))(ca_wa p_re) cp_ent)(oa_ent u_isn) u_hdip))(u_isn u_hdip))(u_isn u_hdip)))	16	15
8678	((((u_hdip (u_csn cp_ent))(p_isn ca_ent))))(ca_wa p_re) cp_ent)(oa_ent u_isn) u_hdip))(u_isn u_hdip))(u_isn u_hdip)))	16	15
8679	(((u_hdip (u_isn oa_wa))(u_csn (cp_ent ca_ent)))))(ca_wa p_re) cp_ent)(oa_ent u_hdip))(u_isn u_hdip))(u_isn u_hdip)))	20	15
8680	(((u_hdip (u_isn oa_wa))(u_csn (cp_ent ca_ent)))))(ca_wa p_re) cp_ent)(oa_ent u_isn))(u_hdip u_hdip))(u_isn u_hdip)))	17	15
8681	(((u_hdip (u_isn u_csn))(cp_ent ca_ent))))(ca_wa p_re) cp_ent)(oa_ent u_isn) u_hdip))(u_isn u_hdip))(u_isn u_hdip)))	16	15
8682	(((u_isn oa_wa) u_csn)(cp_ent ca_ent)))))(ca_wa p_re) ca_wa)((cp_ent oa_ent) u_hdip))(u_isn u_hdip))(u_isn u_hdip)))	20	15
8683	((((u_isn oa_wa) u_csn)(cp_ent ca_ent)))))(ca_wa p_re) cp_ent)(oa_ent u_isn) u_hdip))(u_isn u_hdip))(u_isn u_hdip)))	18	15
8684	(((p_isn ca_ent))((ca_wa p_re) ca_wa) oa_ent))(oa_wa u_hdip)(u_isn oa_wa))))(u_csn cp_ent) oa_ent)(oa_wa u_hdip)))	19	15
8685	(((p_isn ca_ent))((ca_wa p_re) cp_ent) oa_ent))(oa_wa u_hdip)(u_isn oa_wa))))(u_csn cp_ent) oa_ent)(oa_wa u_hdip)))	18	15
8686	(((p_isn ca_ent))((ca_wa p_re) cp_ent) oa_ent))(oa_wa u_hdip)(u_isn oa_wa))))(u_csn cp_ent) oa_ent)(u_isn u_hdip)))	18	15
8687	(((u_isn u_hdip)(u_isn oa_wa))(u_csn cp_ent) p_csn))) ca_ent))))(ca_wa p_re) ca_wa)(oa_ent u_isn))(u_hdip u_hdip)))	16	15
8688	(((u_isn u_hdip)(u_isn oa_wa))(u_csn cp_ent) p_csn))) ca_ent))))(ca_wa p_re) cp_ent)(oa_ent u_hdip))(u_isn u_hdip)))	18	15
8689	(((u_isn u_hdip)(u_isn oa_wa))(u_csn cp_ent) p_csn))) ca_ent))))(ca_wa p_re) cp_ent)(oa_ent u_isn))(u_hdip u_hdip)))	18	15
8690	((((p_csn (oa_ent u_isn)) u_hdip)(u_isn oa_wa))(u_csn cp_ent))))(p_csn (ca_ent ca_wa))(p_isn p_re))(ca_wa p_csn)))	21	15
8691	(((p_isn ca_ent))(p_re ca_wa) oa_ent))(oa_wa u_hdip)(u_isn oa_wa))))(u_csn cp_ent) oa_ent))(u_hdip (u_isn oa_wa)))	16	15
8692	(((u_hdip oa_wa)(u_csn (cp_ent ca_ent))))(ca_wa p_re) cp_ent)(oa_ent u_isn) u_hdip)))(u_isn u_hdip))(u_isn u_hdip)))	17	15
8693	((((u_isn u_hdip))(u_isn oa_wa) u_csn))(cp_ent ca_ent))))(ca_wa p_re) cp_ent)(oa_ent u_isn) u_hdip)))(u_isn u_hdip)))	19	15
8694	(((oa_wa (u_csn cp_ent))(p_isn ca_wa))(p_re ca_wa)(cp_ent oa_ent)(oa_wa u_hdip))))(u_isn u_hdip))(u_isn u_hdip)))	19	15
8695	(((u_hdip (u_isn oa_wa)) u_csn)(ca_ent ca_wa))))(p_re ca_wa)((cp_ent oa_ent) u_hdip))(u_isn u_hdip))(u_isn u_hdip)))	17	15
8696	(((u_hdip (u_isn oa_wa)) u_csn)(cp_ent ca_ent))))(ca_wa p_re) cp_ent)(oa_ent u_hdip))(u_isn u_hdip)))(u_isn u_hdip)))	18	15
8697	(((((cp_ent ca_ent))(ca_wa p_re) ca_wa)(cp_ent oa_ent)))(oa_wa u_hdip))(u_isn u_hdip))(u_isn u_hdip))(u_isn oa_wa)))	23	15
8698	((((u_isn oa_wa) u_csn)(cp_ent ca_ent))))(ca_wa p_re) cp_ent)((oa_ent u_isn) u_hdip)))(u_isn u_hdip))(u_isn u_hdip)))	17	15
8699	((ca_ent p_isn))))(p_re ca_wa)((cp_ent oa_ent) u_hdip))(u_isn u_hdip))(u_isn u_hdip))(u_isn oa_wa))(u_csn cp_ent)))	25	15
8700	((ca_ent p_isn))))(p_re ca_wa)((cp_ent oa_ent) u_hdip))(u_isn u_hdip))(u_isn u_hdip))(u_isn oa_wa))(u_csn cp_ent)))	23	15
8701	(((cp_ent ca_ent))))(ca_wa p_re) ca_wa)(cp_ent oa_ent))(oa_wa u_hdip))(u_isn u_hdip))(u_isn u_hdip))(u_isn oa_wa)))	23	15
8702	((cp_ent ca_ent)))))(ca_wa p_re) ca_wa)(cp_ent oa_ent))(u_hdip u_hdip))(u_isn u_hdip))(u_isn u_hdip))(u_csn cp_ent)))	23	15
8703	((cp_ent ca_ent)))))(ca_wa p_re) ca_wa)(cp_ent oa_ent))(u_hdip u_hdip))(u_isn u_hdip))(u_isn u_hdip))(u_isn oa_wa)))	23	15
8704	((cp_ent ca_ent)))))(ca_wa p_re) ca_wa)(p_csn oa_ent))(u_hdip u_hdip))(u_isn u_hdip))(u_isn u_hdip))(u_csn cp_ent)))	17	15
8705	((oa_wa u_hdip))((u_csn cp_ent)(p_csn (p_isn ca_ent))))(ca_wa p_re) cp_ent) oa_ent)(u_isn u_hdip))(u_isn u_hdip)))	16	15
8706	((p_isn ca_ent)))))(ca_wa p_re) ca_wa)((cp_ent oa_ent)(oa_wa u_hdip))(u_isn u_hdip))(u_isn u_hdip))(u_isn oa_wa)))	18	15
8707	((p_isn ca_wa))))(p_re ca_wa)((cp_ent oa_ent) u_hdip))(u_isn u_hdip))(u_isn u_hdip))(u_isn u_hdip))(u_isn oa_wa)))	24	15
8708	((u_csn cp_ent)(ca_ent ca_wa))))(p_re ca_wa)((cp_ent oa_ent) u_hdip))(u_isn u_hdip))(u_isn u_hdip))(u_isn oa_wa)))	21	15
8709	((u_hdip u_hdip))((u_isn oa_wa) u_csn)(cp_ent ca_ent))))(ca_wa p_re) cp_ent) oa_ent)(u_isn u_hdip))(u_isn u_hdip)))	21	15
8710	((oa_wa (u_csn (cp_ent ca_ent)))))(ca_wa p_re) ca_wa)(cp_ent oa_ent))(u_isn u_hdip))(u_isn oa_wa))(u_isn oa_wa)))	19	15
8711	((u_csn cp_ent)(p_isn ca_wa))))(p_re ca_wa)((cp_ent oa_ent) oa_wa))(u_isn u_hdip))(u_isn u_hdip))(u_isn u_hdip)))	17	15
8712	((u_csn cp_ent)(p_isn ca_wa))))(p_re ca_wa)((cp_ent oa_ent) u_hdip))(u_isn u_hdip))(u_isn u_hdip))(u_isn u_hdip)))	18	15
8713	(((oa_wa u_csn)(cp_ent ca_wa))))(p_re ca_wa)((cp_ent oa_ent) oa_wa))(u_hdip u_hdip))(u_isn u_hdip))(u_isn oa_wa)))	16	15
8714	((u_csn cp_ent)(p_isn ca_ent)))))(ca_wa p_re) ca_wa)(cp_ent oa_ent))(u_isn u_hdip))(u_isn oa_wa))(u_isn oa_wa)))	19	15
8715	((u_csn cp_ent)(p_isn ca_wa))))(p_re ca_wa)((cp_ent oa_ent) oa_wa))(u_isn u_hdip))(u_isn u_hdip))(u_isn oa_wa)))	17	15
8716	((u_csn cp_ent)(p_isn ca_wa))))(p_re ca_wa)((cp_ent oa_ent) u_hdip))(u_isn u_hdip))(u_isn u_hdip))(u_isn oa_wa)))	19	15
8717	(((u_hdip u_csn)(cp_ent ca_wa))))(p_re ca_wa)((cp_ent oa_ent) oa_wa))(u_hdip u_hdip))(u_isn u_hdip))(u_isn oa_wa)))	15	15
8718	((u_csn cp_ent)(p_csn (p_isn ca_ent)))))(ca_wa p_re) cp_ent) oa_ent)(u_isn u_hdip))(u_isn u_hdip))(u_isn u_hdip)))	15	15
8719	(((u_hdip oa_wa)(u_csn (cp_ent ca_ent)))))(ca_wa p_re) cp_ent) oa_ent)(u_isn u_hdip))(u_isn u_hdip))(u_isn u_hdip)))	19	15
8720	(((oa_wa (u_csn cp_ent))(p_isn ca_ent)))))(ca_wa p_re) cp_ent) oa_ent)(u_isn u_hdip))(u_isn u_hdip))(u_isn u_hdip)))	18	15
8721	(((u_hdip (u_csn cp_ent))(p_csn ca_ent)))))(ca_wa p_re) cp_ent) oa_ent)(u_isn u_hdip))(u_isn u_hdip))(u_isn u_hdip)))	15	15
8722	(((u_hdip (u_csn cp_ent))(p_isn ca_ent)))))(ca_wa p_re) cp_ent) oa_ent)(u_isn u_hdip))(u_isn u_hdip))(u_isn u_hdip)))	18	15
8723	(((u_csn cp_ent) p_csn)(p_isn ca_ent)))))(ca_wa p_re) cp_ent) oa_ent)(u_isn u_hdip))(u_isn u_hdip))(u_isn u_hdip)))	17	15
8724	(((u_hdip oa_wa) u_csn)(cp_ent ca_ent)))))(ca_wa p_re) ca_wa) oa_ent)(u_hdip u_hdip))(u_isn u_hdip))(u_isn u_hdip)))	16	15
8725	(((u_isn oa_wa) u_csn)(cp_ent ca_ent)))))(ca_wa p_re) cp_ent) oa_ent)(u_isn u_hdip))(u_isn u_hdip))(u_isn u_hdip)))	19	15
8726	(((p_isn ca_wa))(p_isn p_re))((ca_wa p_csn) oa_ent)(u_isn u_hdip))(u_isn u_hdip))))(u_isn u_hdip))(u_isn u_hdip)))	19	15
8727	(((oa_wa u_hdip))((u_csn cp_ent)(p_csn (p_isn ca_ent))))(ca_wa p_re) cp_ent) oa_ent)(u_isn u_hdip)))(u_isn u_hdip)))	15	15
8728	(((u_csn (cp_ent ca_ent)))))(ca_wa p_re) ca_wa)((cp_ent oa_ent) oa_wa))(u_hdip u_hdip))(u_isn u_hdip))(u_isn oa_wa)))	18	15
8729	(((oa_wa u_csn)(cp_ent ca_wa))))(p_re ca_wa)((cp_ent oa_ent) u_hdip))(u_isn u_hdip))(u_isn u_hdip))(u_isn oa_wa)))	20	15
8730	(((u_hdip oa_wa)(u_csn (cp_ent ca_ent)))))(ca_wa p_re) cp_ent) oa_ent)(u_isn u_hdip))(u_isn u_hdip))(u_isn u_hdip)))	17	15
8731	((((u_hdip (u_csn cp_ent))(p_isn ca_ent)))))(ca_wa p_re) cp_ent) oa_ent)(u_isn u_hdip))(u_isn u_hdip))(u_isn u_hdip)))	16	15
8732	((((u_isn oa_wa) u_csn)(cp_ent ca_ent)))))(ca_wa p_re) cp_ent) oa_ent)(u_isn u_hdip))(u_isn u_hdip))(u_isn u_hdip)))	17	15
8733	(((cp_ent ca_ent))(ca_wa p_re) ca_wa)((cp_ent oa_ent) oa_wa))(u_isn oa_wa))(u_hdip (u_isn u_csn)))(u_isn cp_ent)))	16	15
8734	((((p_isn ca_ent))(ca_wa p_re) ca_wa)(cp_ent oa_ent)))(oa_wa u_hdip))(u_isn u_hdip))(u_isn u_hdip))(u_isn oa_wa)))	18	15
8735	((p_isn ca_ent)))))(ca_wa p_re) ca_wa)(cp_ent oa_ent))(oa_wa u_hdip))(u_isn u_hdip))(u_isn u_hdip))(u_isn oa_wa)))	21	15
8736	(((p_isn ca_ent))((ca_wa p_re) ca_wa)((cp_ent oa_ent) oa_wa))(u_hdip u_hdip)))(u_isn oa_wa))(u_csn cp_ent) oa_ent)))	18	15
8737	(((p_isn ca_ent))((ca_wa p_re) cp_ent)(oa_ent (oa_wa u_hdip)))(u_isn u_hdip)))(u_isn oa_wa))(u_csn cp_ent) oa_ent)))	18	15
8738	(((oa_wa u_csn)(cp_ent ca_wa))))(p_re ca_wa)((cp_ent oa_ent) oa_wa))(u_hdip u_hdip)))(u_isn u_hdip))(u_isn oa_wa)))	19	15
8739	((((cp_ent ca_ent))(ca_wa p_re) ca_wa)((cp_ent oa_ent) oa_wa)))(u_hdip u_hdip))(u_isn oa_wa))(u_hdip (u_isn u_csn)))	16	15
8740	((((cp_ent ca_ent)))(ca_wa p_re) ca_wa)((cp_ent oa_ent))(oa_wa u_hdip)))(u_isn u_hdip))(u_isn u_hdip))(u_isn oa_wa)))	24	15
8741	((((cp_ent ca_ent))(ca_wa p_re) ca_wa)(cp_ent oa_ent))))(oa_wa u_hdip)(u_isn oa_wa)))(u_csn cp_ent)(oa_ent u_isn)))	18	15
8742	((((p_isn ca_ent)))(ca_wa p_re) ca_wa)((cp_ent oa_ent)(oa_wa u_hdip))))(u_isn u_hdip))(u_isn oa_wa))(u_csn cp_ent)))	20	15
8743	((((p_isn ca_ent))(ca_wa p_re) ca_wa)((cp_ent oa_ent)(oa_wa u_hdip))))(u_isn u_hdip))(u_isn u_hdip))(u_isn oa_wa)))	22	15
8744	((((((u_csn cp_ent)(p_isn ca_ent))(ca_wa p_re))(p_isn ca_wa))((oa_ent u_isn) u_hdip))(u_isn u_hdip))(u_isn u_hdip)))	14	15
8745	(((((u_csn (u_isn cp_ent))(p_isn ca_wa))(p_re ca_wa))(cp_ent oa_ent)(u_isn u_hdip)))(u_isn oa_wa))(u_isn u_hdip)))	15	15
8746	(((ca_ent ca_wa))))(p_re ca_wa)((cp_ent oa_ent) u_hdip))(u_isn u_hdip))(u_isn u_hdip))(u_isn u_hdip)))(u_csn cp_ent)))	26	15
8747	(((ca_ent ca_wa))))(p_re ca_wa)((cp_ent oa_ent) u_hdip))(u_isn u_hdip))(u_isn u_hdip))(u_isn u_hdip))(u_isn oa_wa)))	24	15
8748	(((((cp_ent ca_ent))(ca_wa p_re) cp_ent))(oa_ent u_isn))(u_hdip u_hdip))(u_isn u_hdip))(u_isn oa_wa))(u_csn cp_ent)))	21	15
8749	((ca_ent ca_wa))))(p_re ca_wa)(p_csn (oa_ent u_isn)))(u_hdip u_hdip))(u_isn u_hdip))(u_isn u_hdip))(u_csn cp_ent)))	20	15
8750	((ca_ent ca_wa))))(p_re ca_wa)((cp_ent oa_ent) oa_wa))(u_isn u_hdip))(u_isn u_hdip))(u_isn oa_wa))(u_csn cp_ent)))	21	15
8751	((ca_ent ca_wa))))(p_re ca_wa)((cp_ent oa_ent) oa_wa))(u_isn u_hdip))(u_isn u_hdip))(u_isn u_hdip))(u_isn oa_wa)))	25	15
8752	((ca_ent p_isn))))(p_re ca_wa)(p_csn (oa_ent u_isn)))(u_hdip u_hdip))(u_isn u_hdip))(u_isn u_hdip))(u_csn cp_ent)))	16	15
8753	((ca_ent p_isn))))(p_re ca_wa)((cp_ent oa_ent) oa_wa))(u_isn u_hdip))(u_isn u_hdip))(u_isn oa_wa))(u_csn cp_ent)))	19	15
8754	((ca_ent p_isn))))(p_re ca_wa)((cp_ent oa_ent) oa_wa))(u_isn u_hdip))(u_isn u_hdip))(u_isn u_hdip))(u_isn oa_wa)))	23	15
8755	((ca_ent p_isn))))(p_re ca_wa)((cp_ent oa_ent) u_hdip))(u_isn u_hdip))(u_isn u_hdip))(u_isn u_hdip))(u_csn cp_ent)))	22	15
8756	((ca_ent p_isn))))(p_re ca_wa)((cp_ent oa_ent) u_hdip))(u_isn u_hdip))(u_isn u_hdip))(u_isn u_hdip))(u_isn oa_wa)))	22	15
8757	((cp_ent ca_ent))))(p_re ca_wa)((cp_ent oa_ent) oa_wa))(u_isn u_hdip))(u_isn u_hdip))(u_isn oa_wa))(u_csn cp_ent)))	19	15
8758	((cp_ent ca_ent))))(p_re ca_wa)((cp_ent oa_ent) oa_wa))(u_isn u_hdip))(u_isn u_hdip))(u_isn u_hdip))(u_isn oa_wa)))	21	15
8759	((cp_ent ca_ent)))))(ca_wa p_re) ca_wa)(cp_ent oa_ent))(u_isn u_hdip))(u_isn oa_wa))(u_isn oa_wa))(u_isn u_hdip)))	21	15
8760	((p_isn ca_wa))))(p_re ca_wa)((cp_ent oa_ent) oa_wa))(u_isn u_hdip))(u_isn u_hdip))(u_isn u_hdip))(u_isn oa_wa)))	18	15
8761	((p_isn ca_ent)))))(ca_wa p_re) ca_wa)(oa_ent u_isn))(u_hdip u_hdip))(u_isn u_hdip))(u_isn oa_wa))(u_csn cp_ent)))	22	15
8762	((p_isn ca_ent)))))(ca_wa p_re) ca_wa)((cp_ent oa_ent))(u_isn u_hdip))(u_isn oa_wa))(u_isn oa_wa))(u_isn u_hdip)))	23	15
8763	((p_isn ca_wa))))(p_re ca_wa)((cp_ent oa_ent) oa_wa))(u_isn u_hdip))(u_isn u_hdip))(u_isn oa_wa))(u_csn cp_ent)))	20	15
8764	((p_isn ca_wa))))(p_re ca_wa)((cp_ent oa_ent) oa_wa))(u_isn u_hdip))(u_isn u_hdip))(u_isn u_hdip))(u_isn oa_wa)))	25	15
8765	((p_isn ca_wa))))(p_re ca_wa)((cp_ent oa_ent) u_hdip))(u_isn u_hdip))(u_isn u_hdip))(u_isn oa_wa))(u_csn cp_ent)))	24	15
8766	((p_isn ca_wa))))(p_re ca_wa)((cp_ent oa_ent) u_hdip))(u_isn u_hdip))(u_isn u_hdip))(u_isn u_hdip))(u_csn cp_ent)))	26	15
8767	(((p_isn ca_wa))(p_isn p_re))((ca_wa p_csn) oa_ent)(u_hdip u_hdip)))(u_isn u_hdip))(u_isn u_hdip)))(u_isn u_csn)))	17	15
8768	((ca_ent ca_wa))))(p_re ca_wa)((cp_ent oa_ent) u_hdip))(u_isn u_hdip))(u_isn u_hdip))(u_isn oa_wa)))(u_csn cp_ent)))	26	15
8769	((cp_ent ca_wa))))(p_re ca_wa)((cp_ent oa_ent) u_hdip))(u_isn u_hdip))(u_isn u_hdip))(u_isn oa_wa))(u_csn cp_ent)))	23	15
8770	((p_isn ca_ent))))(p_re ca_wa)((cp_ent oa_ent) oa_wa))(u_isn u_hdip))(u_isn u_hdip))(u_isn u_hdip))(u_isn oa_wa)))	20	15
8771	((p_isn ca_ent))))(ca_wa p_re) cp_ent)(oa_ent u_isn)))(u_hdip u_hdip))(u_isn u_hdip))(u_isn oa_wa))(u_csn cp_ent)))	20	15
8772	(((p_isn ca_wa))))(p_re ca_wa)(p_csn (oa_ent u_isn)))(u_hdip u_hdip))(u_isn u_hdip))(u_isn u_hdip))(u_csn cp_ent)))	21	15
8773	((((ca_ent p_isn))(p_re ca_wa)(p_csn (oa_ent u_isn)))(u_hdip u_hdip))(u_isn u_hdip))(u_isn u_hdip))(u_csn cp_ent)))	21	15
8774	(((ca_ent ca_wa))))(p_re ca_wa)(p_csn (oa_ent u_isn)))(u_hdip u_hdip))(u_isn u_hdip)))(u_isn u_hdip))(u_csn cp_ent)))	22	15
8775	(((ca_ent ca_wa))))(p_re ca_wa)((cp_ent oa_ent) oa_wa))(u_isn u_hdip))(u_isn u_hdip)))(u_isn oa_wa))(u_csn cp_ent)))	22	15
8776	(((ca_ent ca_wa))))(p_re ca_wa)((cp_ent oa_ent) u_hdip))(u_isn u_hdip))(u_isn u_hdip)))(u_isn u_hdip))(u_csn cp_ent)))	27	15
8777	(((ca_ent ca_wa))))(p_re ca_wa)((cp_ent oa_ent) u_hdip))(u_isn u_hdip))(u_isn u_hdip)))(u_isn u_hdip))(u_isn oa_wa)))	25	15
8778	(((((ca_wa p_re) ca_wa)(cp_ent oa_ent))(u_hdip u_hdip))(u_isn u_hdip))(u_isn u_hdip))(u_isn oa_wa))(u_csn cp_ent)))	23	15
8779	(((cp_ent ca_ent))))(p_re ca_wa)((cp_ent oa_ent) oa_wa))(u_isn u_hdip))(u_isn u_hdip)))(u_isn u_hdip))(u_isn oa_wa)))	19	15
8780	(((cp_ent ca_ent))))(p_re ca_wa)((cp_ent oa_ent) u_hdip))(u_isn u_hdip))(u_isn u_hdip))(u_isn oa_wa))(u_csn cp_ent)))	25	15
8781	(((cp_ent ca_ent))))(p_re ca_wa)((cp_ent oa_ent) u_hdip))(u_isn u_hdip))(u_isn u_hdip))(u_isn u_hdip))(u_isn oa_wa)))	22	15
8782	(((cp_ent ca_ent))))(ca_wa p_re) ca_wa)(cp_ent oa_ent))(u_hdip u_hdip))(u_isn u_hdip)))(u_isn u_hdip))(u_isn oa_wa)))	22	15
8783	(((cp_ent ca_wa))))(p_re ca_wa)((cp_ent oa_ent) u_hdip))(u_isn u_hdip))(u_isn u_hdip)))(u_isn u_hdip))(u_isn oa_wa)))	20	15
8784	(((p_isn ca_ent)))))(ca_wa p_re) ca_wa)(cp_ent oa_ent))(oa_wa u_hdip))(u_isn u_hdip))(u_isn oa_wa))(u_csn cp_ent)))	20	15
8785	(((p_isn ca_ent)))))(ca_wa p_re) ca_wa)(cp_ent oa_ent))(u_isn u_hdip))(u_isn u_hdip)))(u_isn u_hdip))(u_isn oa_wa)))	24	15
8786	(((ca_ent p_isn))))(p_re ca_wa)((cp_ent oa_ent) oa_wa))(u_hdip u_hdip))(u_isn u_hdip))(u_isn oa_wa))(u_csn cp_ent)))	22	15
8787	(((cp_ent ca_ent))))(ca_wa p_re) ca_wa)(oa_ent u_isn))(u_hdip u_hdip))(u_isn u_hdip))(u_isn oa_wa))(u_csn cp_ent)))	22	15
8788	(((((cp_ent ca_ent))((ca_wa p_re) cp_ent)(oa_ent u_hdip))(u_isn u_hdip)))(u_isn u_hdip))(u_isn oa_wa))(u_csn cp_ent)))	24	15
8789	(((((cp_ent ca_ent))((ca_wa p_re) cp_ent)(oa_ent u_hdip))(u_isn u_hdip))(u_isn u_hdip))(u_isn u_hdip))(u_isn oa_wa)))	21	15
8790	(((cp_ent ca_wa))))(p_re ca_wa)((cp_ent oa_ent) u_hdip))(u_isn u_hdip))(u_isn u_hdip))(u_isn oa_wa))(u_csn cp_ent)))	20	15
8791	(((p_isn ca_wa))))(p_re ca_wa)((cp_ent oa_ent) oa_wa))(u_hdip u_hdip)))(u_isn u_hdip))(u_isn oa_wa))(u_csn cp_ent)))	21	15
8792	(((((cp_ent ca_ent))(ca_wa p_re) ca_wa)(cp_ent oa_ent)))(oa_wa u_hdip))(u_isn u_hdip))(u_isn oa_wa))(u_csn cp_ent)))	21	15
8793	(((((cp_ent ca_ent))(ca_wa p_re) ca_wa)(oa_ent u_isn)))(u_hdip u_hdip))(u_isn u_hdip))(u_isn oa_wa))(u_csn cp_ent)))	23	15
8794	(((((cp_ent ca_wa))(p_re ca_wa)((cp_ent oa_ent) oa_wa)))(u_isn u_hdip))(u_isn u_hdip))(u_isn oa_wa))(u_csn cp_ent)))	20	15
8795	(((((cp_ent ca_wa))(p_re ca_wa)((cp_ent oa_ent) oa_wa)))(u_isn u_hdip))(u_isn u_hdip))(u_isn u_hdip))(u_isn oa_wa)))	18	15
8796	(((((p_isn ca_ent))(p_re ca_wa)((cp_ent oa_ent) u_hdip))(u_isn u_hdip))(u_isn u_hdip))(u_isn oa_wa))(u_csn cp_ent)))	21	15
8797	(((((p_isn ca_ent))((ca_wa p_re) ca_wa)(cp_ent oa_ent)))(oa_wa u_hdip))(u_isn u_hdip))(u_isn oa_wa))(u_csn cp_ent)))	20	15
8798	(((((p_isn ca_ent))(ca_wa p_re) ca_wa)(cp_ent oa_ent))(u_isn u_hdip))(u_isn oa_wa))(u_isn oa_wa))(u_isn u_hdip)))	24	15
8799	(((((p_isn ca_ent))(ca_wa p_re) ca_wa)(cp_ent oa_ent))(u_isn u_hdip))(u_isn u_hdip))(u_isn u_hdip))(oa_wa u_csn)))	25	15
8800	(((((p_isn ca_ent))(ca_wa p_re) ca_wa)(cp_ent oa_ent))(u_isn u_hdip))(u_isn u_hdip))(u_isn u_hdip))(u_isn oa_wa)))	25	15
8801	(((((p_isn ca_ent))(ca_wa p_re) cp_ent)(oa_ent u_isn))(u_hdip u_hdip))(u_isn u_hdip))(u_isn oa_wa))(u_csn cp_ent)))	23	15
8802	((((((cp_ent ca_ent))(ca_wa p_re) cp_ent))(oa_ent u_hdip))(u_isn u_hdip))(u_isn u_hdip))(u_isn oa_wa))(u_csn cp_ent)))	25	15
8803	(((((cp_ent ca_ent))(ca_wa p_re) cp_ent))(oa_ent u_hdip))(u_isn u_hdip))(u_isn u_hdip))(u_isn u_hdip))(u_isn oa_wa)))	22	15
8804	(((((p_re ca_wa)(cp_ent oa_ent) u_hdip))(u_isn u_hdip))(u_isn u_hdip))(u_isn u_hdip))(u_isn oa_wa))(u_csn cp_ent)))	22	15
8805	((((((ca_wa p_re) ca_wa)(oa_ent u_isn))(u_hdip u_hdip))(u_isn u_hdip))(u_isn u_hdip))(u_isn oa_wa))(u_csn cp_ent)))	19	15
8806	((((((ca_wa p_re) cp_ent)(oa_ent u_hdip))(u_isn u_hdip))(u_isn u_hdip))(u_isn u_hdip))(u_isn oa_wa))(u_csn cp_ent)))	21	15
8807	(((u_isn oa_wa) u_hdip)))(u_csn cp_ent)(p_csn (p_isn ca_ent))(((ca_wa p_re) ca_wa)((cp_ent oa_ent) u_hdip))(u_isn u_hdip)))	15	16
8808	(((u_isn oa_wa) u_hdip))((u_csn cp_ent)(p_csn ca_ent)))))(ca_wa p_re) ca_wa)(cp_ent oa_ent))(u_hdip u_hdip))(u_isn u_hdip)))	17	16
8809	((((u_isn oa_wa) u_hdip))((u_csn cp_ent) p_csn)(p_isn ca_ent))))(ca_wa p_re) cp_ent)(oa_ent u_isn) u_hdip)))(u_isn u_hdip))	14	16
8810	(((u_isn oa_wa))(u_hdip (u_csn cp_ent))(p_csn (p_isn ca_ent))))(ca_wa p_re) cp_ent)(oa_ent u_isn) u_hdip)))(u_isn u_hdip))	15	16
8811	(((u_isn u_hdip))(u_isn oa_wa))(u_csn cp_ent) p_csn))(ca_ent ca_wa))))(p_re ca_wa)((cp_ent oa_ent) u_hdip))(u_isn u_hdip)))	16	16
8812	(((p_isn ca_ent))(p_re ca_wa) oa_ent))(oa_wa u_hdip)(u_isn oa_wa))))(u_csn cp_ent) oa_ent)(u_isn u_hdip))(u_isn u_hdip)))	17	16
8813	(((u_isn u_hdip))(u_isn oa_wa))(u_csn cp_ent) p_csn)))(ca_ent ca_wa))))(p_re ca_wa)((cp_ent oa_ent) u_hdip))(u_isn u_hdip)))	17	16
8814	((u_hdip u_hdip))(((u_isn oa_wa))(u_csn cp_ent) p_csn)) ca_ent))((ca_wa p_re) cp_ent)(oa_ent u_isn) u_hdip))(u_isn u_hdip)))	18	16
8815	((u_isn u_hdip))(((u_isn oa_wa))(u_csn cp_ent) p_csn)) ca_ent))((ca_wa p_re) cp_ent)(oa_ent u_isn) u_hdip))(u_isn u_hdip)))	17	16
8816	((u_hdip (u_isn oa_wa))))((u_csn cp_ent) p_csn)(ca_ent ca_wa))))(p_re ca_wa)(cp_ent oa_ent))(u_hdip u_hdip))(u_isn u_hdip)))	16	16
8817	(((u_hdip oa_wa) u_hdip))))(u_csn cp_ent) p_csn)(ca_ent ca_wa))))(p_re ca_wa)(cp_ent oa_ent))(u_hdip u_hdip)))(u_isn u_hdip)))	15	16
8818	(((u_isn oa_wa) u_hdip)))(u_isn oa_wa)))(u_csn cp_ent) p_csn)(p_isn ca_wa))))(p_re ca_wa)(cp_ent oa_ent))(u_hdip u_hdip)))	14	16
8819	((u_isn oa_wa) u_hdip))))(u_csn cp_ent) p_csn)(ca_ent ca_wa))))(p_re ca_wa)(cp_ent oa_ent))(u_hdip u_hdip))(u_isn u_hdip)))	16	16
8820	(((oa_wa u_hdip))((u_csn cp_ent) p_csn)(ca_ent ca_wa))))(p_re ca_wa)((cp_ent oa_ent) u_hdip))(u_isn u_hdip))(u_isn u_hdip)))	17	16
8821	(((oa_wa u_hdip))((u_csn cp_ent) p_csn) ca_ent))))(ca_wa p_re) cp_ent)(oa_ent u_isn) u_hdip))(u_isn u_hdip))(u_isn u_hdip)))	16	16
8822	(((u_hdip oa_wa)(u_csn (cp_ent ca_ent)))))(ca_wa p_re) ca_wa)(cp_ent oa_ent))(oa_wa u_hdip))(u_isn u_hdip))(u_isn u_hdip)))	17	16
8823	(((u_hdip oa_wa))(u_csn cp_ent)(ca_ent ca_wa))))(p_re ca_wa)(cp_ent oa_ent))(oa_wa u_hdip))(u_isn u_hdip))(u_isn u_hdip)))	17	16
8824	(((u_hdip oa_wa)(u_csn (cp_ent ca_ent)))))(ca_wa p_re) ca_wa)(cp_ent oa_ent))(u_hdip u_hdip))(u_isn u_hdip))(u_isn u_hdip)))	18	16
8825	(((u_hdip oa_wa))((u_csn cp_ent) p_csn)(ca_ent ca_wa))))(p_re ca_wa)((cp_ent oa_ent) u_hdip))(u_isn u_hdip))(u_isn u_hdip)))	16	16
8826	((u_hdip (u_isn oa_wa))))(u_csn cp_ent) p_csn) ca_ent))))(ca_wa p_re) cp_ent)(oa_ent u_hdip))(u_isn u_hdip))(u_isn u_hdip)))	17	16
8827	((u_hdip (u_isn oa_wa))))(u_csn cp_ent) p_csn) ca_ent))))(ca_wa p_re) cp_ent)(oa_ent u_isn))(u_hdip u_hdip))(u_isn u_hdip)))	15	16
8828	(((u_hdip u_hdip))(oa_wa (u_csn cp_ent))(ca_ent ca_wa))))(p_re ca_wa)((cp_ent oa_ent) oa_wa))(u_hdip u_hdip))(u_isn u_hdip)))	17	16
8829	(((u_hdip u_hdip))(oa_wa (u_csn cp_ent)) ca_ent))))(ca_wa p_re) ca_wa)((cp_ent oa_ent) oa_wa))(u_hdip u_hdip))(u_isn u_hdip)))	18	16
8830	(((u_hdip u_hdip))(oa_wa (u_csn cp_ent)) ca_ent))))(ca_wa p_re) cp_ent)(oa_ent (oa_wa u_hdip)))(u_isn u_hdip))(u_isn u_hdip)))	16	16
8831	(((oa_wa (u_csn cp_ent))(p_isn ca_wa))))(p_re ca_wa)((cp_ent oa_ent) u_hdip))(u_isn u_hdip))(u_isn u_hdip))(u_isn oa_wa)))	18	16
8832	(((oa_wa (u_csn cp_ent))(p_isn ca_wa))))(p_re ca_wa)((cp_ent oa_ent) u_hdip))(u_isn u_hdip))(u_isn u_hdip))(u_isn u_hdip)))	17	16
8833	(((u_hdip (oa_wa u_csn))(cp_ent ca_ent)))))(ca_wa p_re) ca_wa)(cp_ent oa_ent))(u_hdip u_hdip))(u_isn u_hdip))(u_isn u_hdip)))	16	16
8834	(((u_hdip (u_csn cp_ent))(p_isn ca_ent)))))(ca_wa p_re) ca_wa)(cp_ent oa_ent))(oa_wa u_hdip))(u_isn u_hdip))(u_isn u_hdip)))	15	16
8835	(((u_hdip (u_csn cp_ent))(p_isn ca_wa))))(p_re ca_wa)((cp_ent oa_ent) u_hdip))(u_isn u_hdip))(u_isn u_hdip))(u_isn u_hdip)))	17	16
8836	(((u_hdip (u_csn cp_ent))(p_csn (p_isn ca_ent)))))(ca_wa p_re) ca_wa)((cp_ent oa_ent) u_hdip))(u_isn u_hdip))(u_isn u_hdip)))	14	16
8837	(((u_hdip (u_csn cp_ent))(p_csn (p_isn ca_ent)))))(ca_wa p_re) cp_ent)(oa_ent u_isn) u_hdip))(u_isn u_hdip))(u_isn u_hdip)))	13	16
8838	(((u_hdip (u_isn oa_wa))((u_csn cp_ent)(ca_ent ca_wa))))(p_re ca_wa)((cp_ent oa_ent) oa_wa))(u_hdip u_hdip))(u_isn u_hdip)))	16	16
8839	(((u_hdip (u_isn oa_wa))((u_csn cp_ent)(ca_ent ca_wa))))(p_re ca_wa)((cp_ent oa_ent) oa_wa))(u_isn u_hdip))(u_isn u_hdip)))	17	16
8840	(((u_hdip (u_isn oa_wa))((u_csn cp_ent)(ca_ent ca_wa))))(p_re ca_wa)((cp_ent oa_ent) u_hdip))(u_isn u_hdip))(u_isn u_hdip)))	20	16
8841	(((u_hdip (u_isn oa_wa))(u_csn (cp_ent ca_ent)))))(ca_wa p_re) ca_wa)(cp_ent oa_ent) oa_wa))(u_hdip u_hdip))(u_isn u_hdip)))	17	16
8842	(((u_hdip (u_isn oa_wa))(u_csn (cp_ent ca_ent)))))(ca_wa p_re) ca_wa)((cp_ent oa_ent) oa_wa))(u_isn u_hdip))(u_isn u_hdip)))	16	16
8843	(((u_hdip (u_isn oa_wa))(u_csn (cp_ent ca_ent)))))(ca_wa p_re) cp_ent)(oa_ent u_isn) u_hdip))(u_isn u_hdip))(u_isn u_hdip)))	19	16
8844	(((u_hdip oa_wa) u_csn)(cp_ent ca_ent)))))(ca_wa p_re) ca_wa)(cp_ent oa_ent))(u_hdip u_hdip))(u_isn u_hdip))(u_isn u_hdip)))	19	16
8845	(((u_isn oa_wa) u_csn)(cp_ent ca_ent)))))(ca_wa p_re) ca_wa)(cp_ent oa_ent))(oa_wa u_hdip))(u_isn u_hdip))(u_isn u_hdip)))	17	16
8846	(((u_isn oa_wa) u_csn)(cp_ent ca_ent)))))(ca_wa p_re) ca_wa)(cp_ent oa_ent))(u_hdip u_hdip))(u_isn u_hdip))(u_isn u_hdip)))	17	16
8847	(((u_isn oa_wa))(u_csn cp_ent) p_csn))(ca_ent ca_wa))))(p_re ca_wa)((cp_ent oa_ent) u_hdip))(u_isn u_hdip))(u_isn u_hdip)))	17	16
8848	(((u_isn oa_wa) u_hdip)(u_csn (cp_ent ca_ent)))))(ca_wa p_re) cp_ent)(oa_ent u_isn) u_hdip))(u_isn u_hdip))(u_isn u_hdip)))	17	16
8849	(((u_isn oa_wa) u_hdip))((u_csn cp_ent)(p_csn ca_ent)))))(ca_wa p_re) cp_ent)(oa_ent u_isn))(u_hdip u_hdip))(u_isn u_hdip)))	15	16
8850	(((u_hdip u_hdip)(u_isn oa_wa))(u_csn cp_ent) p_csn)) ca_ent))))(ca_wa p_re) cp_ent) oa_ent)(u_isn u_hdip))(u_isn u_hdip)))	18	16
8851	(((u_hdip u_hdip))((u_isn oa_wa) u_csn)(ca_ent ca_wa))))(p_re ca_wa)((cp_ent oa_ent) oa_wa))(u_hdip u_hdip))(u_isn u_hdip)))	16	16
8852	(((u_hdip u_hdip))((u_isn oa_wa) u_csn)(cp_ent ca_wa))))(p_re ca_wa)((cp_ent oa_ent) oa_wa))(u_hdip u_hdip))(u_isn u_hdip)))	15	16
8853	(((u_isn oa_wa))(u_hdip (u_csn cp_ent))(p_csn (p_isn ca_ent))))(ca_wa p_re) cp_ent) oa_ent)(u_isn u_hdip))(u_isn u_hdip)))	15	16
8854	(((u_isn oa_wa))(u_csn cp_ent) p_csn)) ca_ent))))(ca_wa p_re) cp_ent)(oa_ent u_isn) u_hdip))(u_isn u_hdip))(u_isn u_hdip)))	16	16
8855	(((u_isn oa_wa) u_hdip))((u_csn cp_ent) p_csn)(p_isn ca_ent))))(ca_wa p_re) ca_wa) oa_ent)(u_hdip u_hdip))(u_isn u_hdip)))	12	16
8856	(((u_isn oa_wa) u_hdip))((u_csn cp_ent) p_csn)(p_isn ca_ent))))(ca_wa p_re) cp_ent) oa_ent)(u_isn u_hdip))(u_isn u_hdip)))	14	16
8857	(((u_hdip u_hdip)(oa_wa (u_csn cp_ent))(ca_ent ca_wa))))(p_re ca_wa)((cp_ent oa_ent) oa_wa))(u_hdip u_hdip))(u_isn u_hdip)))	16	16
8858	(((u_hdip u_hdip)(oa_wa (u_csn cp_ent)) ca_ent))))(ca_wa p_re) ca_wa)((cp_ent oa_ent) oa_wa))(u_hdip u_hdip))(u_isn u_hdip)))	17	16
8859	(((u_hdip u_hdip)(oa_wa (u_csn cp_ent)) ca_ent))))(ca_wa p_re) cp_ent)(oa_ent (oa_wa u_hdip)))(u_isn u_hdip))(u_isn u_hdip)))	15	16
8860	(((u_hdip u_hdip)(u_isn oa_wa))(u_csn cp_ent) p_csn)))(ca_ent ca_wa))))(p_re ca_wa)((cp_ent oa_ent) u_hdip))(u_isn u_hdip)))	18	16
8861	(((u_hdip u_hdip)(u_isn oa_wa))(u_csn cp_ent) p_csn)) ca_ent))))(ca_wa p_re) cp_ent)(oa_ent u_isn) u_hdip))(u_isn u_hdip)))	19	16
8862	(((u_hdip u_hdip))(oa_wa (u_csn cp_ent)) p_csn)(p_isn ca_ent))))(ca_wa p_re) ca_wa)((cp_ent oa_ent) u_hdip))(u_isn u_hdip)))	15	16
8863	(((u_hdip u_hdip))(oa_wa (u_csn cp_ent)) p_csn)(p_isn ca_ent))))(ca_wa p_re) cp_ent)(oa_ent u_isn) u_hdip))(u_isn u_hdip)))	15	16
8864	(((u_hdip u_hdip))(oa_wa (u_csn cp_ent)) p_csn)(p_isn ca_ent))))(ca_wa p_re) cp_ent) oa_ent)(u_isn u_hdip))(u_isn u_hdip)))	15	16
8865	(((u_hdip (u_isn oa_wa))((u_csn cp_ent)(ca_ent ca_wa))))(p_re ca_wa)((cp_ent oa_ent) u_hdip))(u_isn u_hdip))(u_isn u_hdip)))	19	16
8866	(((u_hdip (u_isn oa_wa))(u_csn (cp_ent ca_ent)))))(ca_wa p_re) cp_ent)(oa_ent u_isn) u_hdip))(u_isn u_hdip))(u_isn u_hdip))	17	16
8867	(((u_isn u_hdip)(u_isn oa_wa))(u_csn cp_ent) p_csn)) ca_ent))))(ca_wa p_re) cp_ent)(oa_ent u_isn) u_hdip))(u_isn u_hdip)))	18	16
8868	(((u_isn u_hdip)(u_isn oa_wa))(u_csn cp_ent) p_csn)) ca_ent))))(ca_wa p_re) cp_ent) oa_ent)(u_isn u_hdip))(u_isn u_hdip)))	17	16
8869	(((u_isn u_hdip)(u_isn oa_wa))(u_csn cp_ent) p_csn))(p_isn ca_wa))))(p_re ca_wa)(cp_ent oa_ent) u_hdip))(u_isn u_hdip)))	14	16
8870	((((p_isn ca_ent))(p_re ca_wa) oa_ent))(oa_wa u_hdip)(u_isn oa_wa))))(u_csn cp_ent) oa_ent)(u_isn u_hdip))(u_isn oa_wa))	18	16
8871	((((u_hdip (u_isn oa_wa))(u_csn (cp_ent ca_ent))))(ca_wa p_re) cp_ent)(oa_ent u_isn) u_hdip)))(u_isn u_hdip))(u_isn u_hdip))	16	16
8872	((cp_ent ca_ent)))))(ca_wa p_re) ca_wa)((cp_ent oa_ent) u_hdip))(u_isn u_hdip))(u_isn u_hdip))(u_isn oa_wa))(u_csn cp_ent)))	23	16
8873	((u_hdip (u_isn oa_wa))))(u_csn cp_ent)(p_csn ca_ent)))))(ca_wa p_re) ca_wa)(oa_ent u_isn))(u_hdip u_hdip))(u_isn u_hdip)))	14	16
8874	((u_hdip u_hdip))(((u_isn oa_wa))(u_csn cp_ent) p_csn) ca_ent))))(ca_wa p_re) cp_ent) oa_ent)(u_isn u_hdip))(u_isn u_hdip)))	17	16
8875	((u_hdip u_hdip))(((u_isn oa_wa))(u_csn cp_ent) p_csn) ca_ent))((ca_wa p_re) cp_ent)(oa_ent u_isn) u_hdip)))(u_isn u_hdip)))	17	16
8876	(((oa_wa (u_csn cp_ent))(ca_ent ca_wa))))(p_re ca_wa)((cp_ent oa_ent) oa_wa))(u_isn u_hdip))(u_isn u_hdip))(u_isn oa_wa)))	18	16
8877	(((oa_wa (u_csn cp_ent))(ca_ent ca_wa))))(p_re ca_wa)((cp_ent oa_ent) u_hdip))(u_isn u_hdip))(u_isn u_hdip))(u_isn oa_wa)))	19	16
8878	(((oa_wa (u_csn cp_ent))(p_isn ca_wa))))(p_re ca_wa)((cp_ent oa_ent) oa_wa))(u_isn u_hdip))(u_isn u_hdip))(u_isn u_hdip)))	17	16
8879	(((oa_wa u_csn)(cp_ent ca_wa))))(p_re ca_wa)((cp_ent oa_ent)(oa_wa u_hdip)))(u_isn u_hdip))(u_isn u_hdip))(u_isn oa_wa)))	16	16
8880	(((oa_wa u_csn)(cp_ent ca_wa))))(p_re ca_wa)((cp_ent oa_ent))(oa_wa u_hdip))(u_isn u_hdip))(u_isn u_hdip))(u_isn oa_wa)))	16	16
8881	(((oa_wa u_csn)(cp_ent ca_wa))))(p_re ca_wa)((cp_ent oa_ent))(u_hdip u_hdip))(u_isn u_hdip))(u_isn u_hdip))(u_isn oa_wa)))	16	16
8882	(((u_hdip oa_wa) u_hdip))))(u_csn cp_ent) p_csn) ca_ent)))))(ca_wa p_re) cp_ent)(oa_ent u_isn))(u_hdip u_hdip))(u_isn u_hdip)))	14	16
8883	(((u_hdip oa_wa) u_hdip))))(u_csn cp_ent) p_csn) ca_ent)))))(ca_wa p_re) cp_ent)(oa_ent u_hdip))(u_isn u_hdip))(u_isn u_hdip)))	15	16
8884	(((u_hdip u_csn)(cp_ent ca_wa))))(p_re ca_wa)((cp_ent oa_ent)(oa_wa u_hdip)))(u_isn u_hdip))(u_isn u_hdip))(u_isn oa_wa)))	15	16
8885	(((u_hdip u_csn)(cp_ent ca_wa))))(p_re ca_wa)((cp_ent oa_ent))(oa_wa u_hdip))(u_isn u_hdip))(u_isn u_hdip))(u_isn oa_wa)))	15	16
8886	(((u_isn oa_wa) u_hdip)))(u_csn cp_ent)(p_csn ca_ent)))))(ca_wa p_re) ca_wa)(oa_ent u_isn))(u_hdip u_hdip))(u_isn u_hdip)))	14	16
8887	(((oa_wa u_hdip)))(u_csn cp_ent) p_csn) ca_ent)))))(ca_wa p_re) cp_ent) oa_ent)(u_isn u_hdip))(u_isn u_hdip))(u_isn u_hdip)))	15	16
8888	(((u_hdip oa_wa)(u_csn (cp_ent ca_ent)))))(ca_wa p_re) cp_ent)(oa_ent u_hdip))(u_isn u_hdip))(u_isn u_hdip))(u_isn u_hdip)))	17	16
8889	(((oa_wa (u_csn cp_ent))(p_isn ca_wa))))(p_re ca_wa)((cp_ent oa_ent) oa_wa))(u_isn u_hdip))(u_isn u_hdip))(u_isn oa_wa)))	17	16
8890	(((u_hdip (oa_wa u_csn))(cp_ent ca_wa))))(p_re ca_wa)((cp_ent oa_ent) oa_wa))(u_hdip u_hdip))(u_isn u_hdip))(u_isn oa_wa)))	13	16
8891	(((u_hdip (u_csn cp_ent)) ca_ent))))(ca_wa p_re) ca_wa)((cp_ent oa_ent) oa_wa))(u_hdip u_hdip))(u_isn u_hdip))(u_isn oa_wa)))	16	16
8892	(((u_hdip (u_csn cp_ent))(p_isn ca_ent)))))(ca_wa p_re) cp_ent)(oa_ent u_hdip))(u_isn u_hdip))(u_isn u_hdip))(u_isn oa_wa)))	15	16
8893	(((u_hdip (u_csn cp_ent))(p_csn (p_isn ca_ent)))))(ca_wa p_re) cp_ent) oa_ent)(u_isn u_hdip))(u_isn u_hdip))(u_isn u_hdip)))	13	16
8894	(((u_hdip (u_isn oa_wa))(u_csn (cp_ent ca_ent)))))(ca_wa p_re) cp_ent) oa_ent)(u_isn u_hdip))(u_isn u_hdip))(u_isn u_hdip)))	18	16
8895	(((u_hdip oa_wa) u_csn)(cp_ent ca_wa))))(p_re ca_wa)((cp_ent oa_ent) oa_wa))(u_isn u_hdip))(u_isn u_hdip))(u_isn u_hdip)))	16	16
8896	(((u_hdip oa_wa) u_csn)(cp_ent ca_wa))))(p_re ca_wa)((cp_ent oa_ent) u_hdip))(u_isn u_hdip))(u_isn u_hdip))(u_isn u_hdip)))	17	16
8897	(((u_hdip oa_wa) u_csn)(cp_ent ca_ent)))))(ca_wa p_re) cp_ent)(oa_ent u_hdip))(u_isn u_hdip))(u_isn u_hdip))(u_isn u_hdip)))	18	16
8898	(((u_isn oa_wa) u_csn)(ca_ent ca_wa))))(p_re ca_wa)((cp_ent oa_ent) oa_wa))(u_hdip u_hdip))(u_isn u_hdip))(u_isn oa_wa)))	16	16
8899	(((u_isn oa_wa) u_csn)(cp_ent ca_ent)))))(ca_wa p_re) cp_ent)(oa_ent u_hdip))(u_isn u_hdip))(u_isn u_hdip))(u_isn u_hdip)))	16	16
8900	(((u_isn oa_wa) u_csn)(cp_ent ca_wa))))(p_re ca_wa)((cp_ent oa_ent) u_hdip))(u_isn u_hdip))(u_isn u_hdip))(u_isn u_hdip)))	15	16
8901	(((u_isn oa_wa) u_hdip)(u_csn (cp_ent ca_ent)))))(ca_wa p_re) cp_ent) oa_ent)(u_isn u_hdip))(u_isn u_hdip))(u_isn u_hdip)))	16	16
8902	(((oa_wa (u_csn cp_ent)) ca_ent))))(ca_wa p_re) ca_wa)((cp_ent oa_ent) oa_wa))(u_hdip u_hdip))(u_isn u_hdip)))(u_isn oa_wa)))	17	16
8903	(((u_hdip (u_isn oa_wa))(u_csn (cp_ent ca_ent)))))(ca_wa p_re) cp_ent) oa_ent)(u_isn u_hdip))(u_isn u_hdip))(u_isn u_hdip))	16	16
8904	((((oa_wa u_csn)(cp_ent ca_wa))))(p_re ca_wa)((cp_ent oa_ent))(oa_wa u_hdip))(u_isn u_hdip)))(u_isn u_hdip))(u_isn oa_wa)))	17	16
8905	((((p_isn ca_ent))((ca_wa p_re) ca_wa) oa_ent))(oa_wa u_hdip)(u_isn oa_wa))))(u_csn cp_ent) oa_ent))(u_hdip (u_isn oa_wa)))	19	16
8906	((((p_isn ca_ent))((ca_wa p_re) ca_wa) oa_ent))(oa_wa u_hdip)(u_isn oa_wa))))(u_csn cp_ent) oa_ent))(oa_wa u_hdip) oa_wa))	17	16
8907	((((p_isn ca_ent))((ca_wa p_re) cp_ent) oa_ent))(oa_wa u_hdip)(u_isn oa_wa))))(u_csn cp_ent) oa_ent))(u_hdip (u_isn oa_wa)))	17	16
8908	(((((oa_wa u_csn)(cp_ent ca_wa))(p_re ca_wa)((cp_ent oa_ent)(oa_wa u_hdip))))(u_isn u_hdip))(u_isn u_hdip))(u_isn oa_wa))	18	16
8909	(((((u_csn cp_ent)(ca_ent ca_wa))(p_re ca_wa)((cp_ent oa_ent))(oa_wa u_hdip))(u_isn u_hdip))(u_isn u_hdip))(u_isn oa_wa))	17	16
8910	(((cp_ent ca_ent))))(ca_wa p_re) ca_wa)((cp_ent oa_ent) u_hdip))(u_isn u_hdip))(u_isn u_hdip)))(u_isn u_hdip))(u_isn oa_wa))	21	16
8911	((ca_ent ca_wa))))(p_isn p_re))((ca_wa p_csn)(oa_ent u_isn))(u_hdip u_hdip)))(u_isn u_hdip))(u_isn u_hdip))(u_isn u_csn)))	17	16
8912	((ca_ent ca_wa))))(p_re ca_wa)(cp_ent oa_ent))(oa_wa u_hdip))(u_isn u_hdip))(u_isn u_hdip))(u_isn oa_wa))(u_csn cp_ent)))	21	16
8913	((ca_ent ca_wa))))(p_re ca_wa)(cp_ent oa_ent))(u_hdip u_hdip))(u_isn u_hdip))(u_isn u_hdip))(u_isn oa_wa))(u_csn cp_ent)))	22	16
8914	((ca_ent p_isn))))(p_re ca_wa)(cp_ent oa_ent))(oa_wa u_hdip))(u_isn u_hdip))(u_isn u_hdip))(u_isn oa_wa))(u_csn cp_ent)))	17	16
8915	((ca_ent p_isn))))(p_re ca_wa)(cp_ent oa_ent))(u_hdip u_hdip))(u_isn u_hdip))(u_isn u_hdip))(u_isn oa_wa))(u_csn cp_ent)))	20	16
8916	((cp_ent ca_ent))))(p_isn p_re))((ca_wa p_csn)(oa_ent u_isn))(u_hdip u_hdip)))(u_isn u_hdip))(u_isn u_hdip))(u_isn u_csn)))	15	16
8917	((cp_ent ca_ent)))))(ca_wa p_re) ca_wa)(p_csn (oa_ent u_isn)))(u_hdip u_hdip))(u_isn u_hdip))(u_isn u_hdip))(u_csn cp_ent)))	16	16
8918	((cp_ent ca_ent)))))(ca_wa p_re) ca_wa)((cp_ent oa_ent) u_hdip))(u_isn u_hdip))(u_isn u_hdip))(u_isn u_hdip))(u_csn cp_ent)))	22	16
8919	((cp_ent ca_ent)))))(ca_wa p_re) ca_wa)((cp_ent oa_ent) u_hdip))(u_isn u_hdip))(u_isn u_hdip))(u_isn u_hdip))(u_isn oa_wa)))	22	16
8920	((cp_ent ca_ent)))))(ca_wa p_re) ca_wa)((cp_ent oa_ent) oa_wa))(u_hdip u_hdip))(u_isn u_hdip))(u_isn oa_wa))(u_csn cp_ent)))	17	16
8921	((p_isn ca_ent)))))(ca_wa p_re) ca_wa)((cp_ent oa_ent) u_hdip))(u_isn u_hdip))(u_isn u_hdip))(u_isn oa_wa))(u_csn cp_ent)))	23	16
8922	((p_isn ca_wa))))(p_re ca_wa)(cp_ent oa_ent))(oa_wa u_hdip))(u_isn u_hdip))(u_isn u_hdip))(u_isn oa_wa))(u_csn cp_ent)))	20	16
8923	(((cp_ent ca_ent)))))(ca_wa ca_wa)(cp_ent oa_ent))(u_hdip u_hdip))(u_isn u_hdip))(u_isn u_hdip))(u_isn oa_wa)))(u_csn cp_ent)))	17	16
8924	(((ca_ent ca_wa))))(p_re ca_wa)(cp_ent oa_ent))(u_hdip u_hdip))(u_isn u_hdip))(u_isn u_hdip)))(u_isn oa_wa))(u_csn cp_ent)))	23	16
8925	(((p_isn ca_ent))))(ca_wa p_re) ca_wa)((cp_ent oa_ent) oa_wa))(u_isn u_hdip))(u_isn u_hdip))(u_isn oa_wa))(u_csn cp_ent)))	20	16
8926	(((p_isn ca_ent))))(ca_wa p_re) ca_wa)((cp_ent oa_ent) oa_wa))(u_isn u_hdip))(u_isn u_hdip))(u_isn u_hdip))(u_isn oa_wa))	18	16
8927	(((p_isn ca_ent))))(ca_wa p_re) cp_ent) oa_ent)(oa_wa u_hdip))(u_isn u_hdip)))(u_isn oa_wa))(u_csn cp_ent)(oa_ent oa_wa)))	18	16
8928	(((ca_ent p_isn))))(p_re ca_wa)(cp_ent oa_ent))(oa_wa u_hdip))(u_isn u_hdip))(u_isn u_hdip))(u_isn oa_wa))(u_csn cp_ent))	22	16
8929	(((cp_ent ca_wa))))(p_re ca_wa)(cp_ent oa_ent))(u_hdip u_hdip))(u_isn u_hdip))(u_isn u_hdip))(u_isn oa_wa))(u_csn cp_ent)))	18	16
8930	(((p_isn ca_ent))))(ca_wa p_re) cp_ent)(oa_ent (u_isn u_hdip)))(u_isn u_hdip)))(u_isn u_hdip))(u_isn oa_wa))(u_csn cp_ent))	20	16
8931	(((p_isn ca_wa))))(p_re ca_wa)(cp_ent oa_ent))(oa_wa u_hdip))(u_isn u_hdip)))(u_isn u_hdip))(u_isn oa_wa))(u_csn cp_ent))	21	16
8932	(((p_isn ca_wa))))(p_re ca_wa)(cp_ent oa_ent))(oa_wa u_hdip))(u_isn u_hdip)))(u_isn u_hdip))(u_isn u_hdip))(u_isn u_csn))	17	16
8933	((((p_isn ca_ent))(ca_wa p_re) ca_wa)(cp_ent oa_ent)))(oa_wa u_hdip))(u_isn u_hdip))(u_isn oa_wa)))(u_csn cp_ent) oa_ent))	19	16
8934	(((((cp_ent ca_ent))(ca_wa p_re) cp_ent)))(oa_ent u_isn) u_hdip))(u_isn u_hdip))(u_isn u_hdip))(u_isn u_hdip))(u_isn oa_wa))	19	16
8935	((((((ca_wa p_re) ca_wa))(cp_ent oa_ent) u_hdip))(u_isn u_hdip))(u_isn u_hdip))(u_isn u_hdip))(u_isn oa_wa))(u_csn cp_ent))	22	16
8936	((((cp_ent ca_ent)))(ca_wa p_re) ca_wa) oa_ent)(u_hdip u_hdip)))(u_isn u_hdip))(u_isn u_hdip))(u_isn oa_wa))(u_csn cp_ent))	18	16
8937	(((((cp_ent ca_ent)))(ca_wa p_re) cp_ent) oa_ent)(u_hdip u_hdip)))(u_isn u_hdip))(u_isn u_hdip))(u_isn oa_wa))(u_csn cp_ent))	21	16
8938	(((((cp_ent ca_ent)))(ca_wa p_re) ca_wa) oa_ent))(u_hdip u_hdip))(u_isn u_hdip))(u_isn u_hdip))(u_isn oa_wa))(u_csn cp_ent))	19	16
8939	((((((cp_ent ca_ent))(ca_wa p_re) cp_ent)) oa_ent)(u_hdip u_hdip))(u_isn u_hdip))(u_isn u_hdip))(u_isn oa_wa))(u_csn cp_ent))	22	16
8940	(((u_hdip (u_isn oa_wa))((u_csn cp_ent) p_csn)(ca_ent ca_wa))))(p_re ca_wa)((cp_ent oa_ent) u_hdip))(u_isn u_hdip))(u_isn u_hdip)))	16	17
8941	((u_hdip (u_isn oa_wa))))(u_csn cp_ent) p_csn) ca_ent))))(ca_wa p_re) cp_ent)(oa_ent u_isn) u_hdip))(u_isn u_hdip))(u_isn u_hdip)))	15	17
8942	(((u_hdip u_hdip))(oa_wa (u_csn cp_ent)) ca_ent))))(ca_wa p_re) ca_wa)(cp_ent oa_ent))(oa_wa u_hdip))(u_isn u_hdip))(u_isn u_hdip)))	17	17
8943	(((u_hdip (u_isn oa_wa))(u_csn (cp_ent ca_ent)))))(ca_wa p_re) ca_wa)(cp_ent oa_ent))(oa_wa u_hdip))(u_isn u_hdip))(u_isn u_hdip)))	16	17
8944	(((u_hdip (u_isn oa_wa))(u_csn cp_ent)(ca_ent ca_wa))))(p_re ca_wa)(cp_ent oa_ent))(oa_wa u_hdip))(u_isn u_hdip))(u_isn u_hdip)))	16	17
8945	(((u_hdip (u_isn oa_wa))(u_csn (cp_ent ca_ent)))))(ca_wa p_re) ca_wa)(cp_ent oa_ent))(u_hdip u_hdip))(u_isn u_hdip))(u_isn u_hdip)))	17	17
8946	(((u_isn oa_wa) u_hdip))(u_csn cp_ent)(p_csn ca_ent)))))(ca_wa p_re) cp_ent)(oa_ent u_isn) u_hdip))(u_isn u_hdip))(u_isn u_hdip)))	15	17
8947	(((u_hdip u_hdip))((u_isn oa_wa) u_csn)(cp_ent ca_ent)))))(ca_wa p_re) ca_wa)((cp_ent oa_ent) oa_wa))(u_hdip u_hdip))(u_isn u_hdip)))	17	17
8948	(((u_hdip u_hdip))((u_isn oa_wa) u_csn)(cp_ent ca_ent))))(ca_wa p_re) cp_ent)(oa_ent (oa_wa u_hdip)))(u_isn u_hdip))(u_isn u_hdip)))	15	17
8949	(((u_hdip u_hdip))((u_isn oa_wa) u_csn)(cp_ent ca_wa))))(p_re ca_wa)(cp_ent oa_ent)(oa_wa u_hdip)))(u_isn u_hdip))(u_isn u_hdip)))	15	17
8950	(((u_isn oa_wa) u_hdip))((u_csn cp_ent) p_csn)(p_isn ca_ent)))))(ca_wa p_re) ca_wa)(oa_ent u_isn))(u_hdip u_hdip))(u_isn u_hdip)))	12	17

8951	((((u_hdip u_hdip)(oa_wa (u_csn cp_ent))(ca_ent)))))((ca_wa p_re)ca_wa)(cp_ent oa_ent))(oa_wa u_hdip))(u_isn u_hdip))(u_isn u_hdip)))	16	17
8952	((((u_hdip (u_isn oa_wa))u_csn)(cp_ent ca_ent)))))((ca_wa p_re)ca_wa)(cp_ent oa_ent))(u_hdip u_hdip))(u_isn u_hdip))(u_isn u_hdip)))	18	17
8953	((((p_isn ca_ent)))((ca_wa p_re)ca_wa)oa_ent))((oa_wa u_hdip)(u_isn oa_wa)))((((u_csn cp_ent)oa_ent)(u_isn u_hdip))(u_isn u_hdip)))	18	17
8954	((u_hdip u_hdip)((((u_isn oa_wa)u_csn)(cp_ent ca_ent))))((ca_wa p_re)ca_wa)((cp_ent oa_ent)oa_wa))(u_hdip u_hdip))(u_isn u_hdip))))	16	17
8955	((u_hdip u_hdip))(((u_isn oa_wa)u_csn)(cp_ent ca_ent)))))((ca_wa p_re)cp_ent)(oa_ent (oa_wa u_hdip)))(u_isn u_hdip))(u_isn u_hdip))))	14	17
8956	(((u_hdip oa_wa)u_hdip)(((u_csn cp_ent)p_csn)ca_ent)((((ca_wa p_re)cp_ent))(oa_ent u_isn)u_hdip))(u_isn u_hdip))(u_isn u_hdip))))	14	17
8957	(((u_hdip oa_wa)u_hdip))(((u_csn cp_ent)p_csn)(ca_ent ca_wa))))((p_re ca_wa)((cp_ent oa_ent)u_hdip))(u_isn u_hdip))(u_isn u_hdip)))	14	17
8958	(((u_hdip oa_wa)(u_csn (cp_ent ca_ent)))))))((ca_wa p_re)ca_wa)((cp_ent oa_ent)u_hdip))(u_isn u_hdip))(u_isn u_hdip))(u_isn u_hdip)))	16	17
8959	(((oa_wa (u_csn cp_ent))(p_isn ca_wa))))))((p_re ca_wa)((cp_ent oa_ent)(oa_wa u_hdip)))(u_isn u_hdip))(u_isn u_hdip))(u_isn oa_wa)))	15	17
8960	(((oa_wa (u_csn cp_ent))(p_isn ca_wa))))))((p_re ca_wa)(cp_ent oa_ent))(oa_wa u_hdip))(u_isn u_hdip))(u_isn u_hdip))(u_isn oa_wa)))	15	17
8961	(((u_hdip (u_csn cp_ent))(p_isn ca_wa))))))((p_re ca_wa)((cp_ent oa_ent)(oa_wa u_hdip)))(u_isn u_hdip))(u_isn u_hdip))(u_isn oa_wa)))	14	17
8962	(((u_hdip (u_csn cp_ent))(p_isn ca_wa))))))(p_re ca_wa)(cp_ent oa_ent))(oa_wa u_hdip))(u_isn u_hdip))(u_isn u_hdip))(u_isn oa_wa)))	14	17
8963	(((u_hdip (u_isn oa_wa))(u_csn (cp_ent ca_ent)))))))((ca_wa p_re)cp_ent)(oa_ent u_hdip))(u_isn u_hdip))(u_isn u_hdip))(u_isn u_hdip)))	16	17
8964	((((u_isn oa_wa)u_csn)(cp_ent ca_ent))))))(ca_wa p_re)cp_ent)(oa_ent (oa_wa u_hdip)))(u_isn u_hdip))(u_isn u_hdip))(u_isn oa_wa)))	15	17
8965	((((u_hdip (u_isn oa_wa))u_csn)(cp_ent ca_wa))))((p_re ca_wa)((cp_ent oa_ent)u_hdip))(u_isn u_hdip))(u_isn u_hdip))(u_isn u_hdip)))	16	17
8966	((((u_hdip (u_isn oa_wa))u_csn)(cp_ent ca_ent)))))((ca_wa p_re)cp_ent)(oa_ent u_hdip))(u_isn u_hdip))(u_isn u_hdip))(u_isn u_hdip)))	17	17
8967	((((u_hdip (u_isn oa_wa))u_csn)(ca_ent ca_wa)))))((p_re ca_wa)((cp_ent oa_ent)oa_wa))(u_isn u_hdip))(u_isn u_hdip))(u_isn u_hdip)))	15	17
8968	(((((oa_wa (u_csn cp_ent))(ca_ent ca_wa))((((p_re ca_wa)(cp_ent oa_ent))(oa_wa u_hdip))(u_isn u_hdip)))(u_isn u_hdip))(u_isn oa_wa))	17	17
8969	(((((p_isn ca_ent)((((ca_wa p_re)ca_wa)oa_ent))((oa_wa u_hdip)(u_isn oa_wa))))(u_csn cp_ent)oa_ent))((oa_wa u_hdip)(u_isn oa_wa)))	18	17
8970	(((((p_isn ca_ent)))((ca_wa p_re)ca_wa)oa_ent))((oa_wa u_hdip)(u_isn oa_wa)))((u_csn cp_ent)oa_ent)(u_isn u_hdip)))(u_isn oa_wa))	19	17
8971	(((((p_isn ca_ent)))((ca_wa p_re)cp_ent)oa_ent))((oa_wa u_hdip)(u_isn oa_wa)))((u_csn cp_ent)oa_ent)(u_isn u_hdip)))(u_isn u_hdip))	16	17
8972	(((((p_isn ca_ent)))((p_re ca_wa)((cp_ent oa_ent)oa_wa))(u_isn u_hdip)))(u_isn oa_wa))((u_csn cp_ent)oa_ent))(u_hdip (u_isn oa_wa)))	19	17
8973	((((((oa_wa (u_csn cp_ent))(p_isn ca_wa)))((p_re ca_wa)((cp_ent oa_ent)(oa_wa u_hdip)))(u_isn u_hdip))(u_isn u_hdip))(u_isn oa_wa))	17	17
8974	((cp_ent ca_ent))))))((ca_wa p_re)ca_wa)(cp_ent oa_ent))(u_hdip u_hdip))(u_isn u_hdip))(u_isn u_hdip))(u_isn oa_wa))(u_csn cp_ent)))	20	17
8975	((p_isn ca_wa))))))((p_re ca_wa)((cp_ent oa_ent)u_hdip))(u_isn u_hdip))(u_isn u_hdip))(u_isn u_hdip))(u_isn oa_wa))(u_csn cp_ent)))	21	17
8976	((u_hdip (u_isn oa_wa))(((u_csn cp_ent)(p_csn ca_ent)))))((ca_wa p_re)cp_ent)oa_ent)(u_isn u_hdip))(u_isn u_hdip))(u_isn u_hdip))))	14	17
8977	(((u_hdip oa_wa)u_hdip)(((u_csn cp_ent)(p_csn ca_ent)))))((ca_wa p_re)cp_ent)oa_ent)(u_isn u_hdip))(u_isn u_hdip))(u_isn u_hdip))))	13	17
8978	(((((cp_ent ca_ent)))((ca_wa p_re)ca_wa)((cp_ent oa_ent)oa_wa)))(u_hdip u_hdip))(u_isn oa_wa))(u_hdip (u_isn u_csn)))(u_isn cp_ent))	14	17
8979	((((((p_isn ca_ent)))((ca_wa p_re)ca_wa)((cp_ent oa_ent)(oa_wa u_hdip)))(u_isn u_hdip))(u_isn u_hdip))(u_isn oa_wa))(u_csn cp_ent))	18	17
8980	(((((((cp_ent ca_ent))((ca_wa p_re)cp_ent))(oa_ent u_hdip))(u_isn u_hdip))(u_isn u_hdip))(u_isn u_hdip))(u_isn oa_wa))(u_csn cp_ent))	18	17
8981	((ca_ent ca_wa))))))((p_re ca_wa)((cp_ent oa_ent)u_hdip))(u_isn u_hdip))(u_isn u_hdip))(u_isn u_hdip))(u_isn oa_wa))(u_csn cp_ent)))	21	17
8982	((ca_ent p_isn))))))((p_re ca_wa)((cp_ent oa_ent)u_hdip))(u_isn u_hdip))(u_isn u_hdip))(u_isn u_hdip))(u_isn oa_wa))(u_csn cp_ent)))	19	17
8983	(((((ca_ent ca_wa))))((p_re ca_wa)((cp_ent oa_ent)u_hdip))(u_isn u_hdip))(u_isn u_hdip)))(u_isn u_hdip))(u_isn oa_wa))(u_csn cp_ent))	22	17
8984	(((((cp_ent ca_wa))))((p_re ca_wa)((cp_ent oa_ent)u_hdip))(u_isn u_hdip))(u_isn u_hdip)))(u_isn u_hdip))(u_isn oa_wa))(u_csn cp_ent))	17	17
8985	((((((p_isn ca_ent)))))((ca_wa p_re)ca_wa)(cp_ent oa_ent))(u_isn u_hdip))(u_isn u_hdip)))(u_isn u_hdip))(u_isn oa_wa))(u_csn cp_ent))	21	17
8986	(((((((p_isn ca_ent)))((ca_wa p_re)ca_wa)(cp_ent oa_ent)))(u_isn u_hdip))(u_isn u_hdip))(u_isn u_hdip))(u_isn oa_wa))(u_csn cp_ent))	22	17
8987	(((u_hdip u_hdip))((u_isn oa_wa)u_csn)(cp_ent ca_ent)))))))((ca_wa p_re)ca_wa)(cp_ent oa_ent))(oa_wa u_hdip))(u_isn u_hdip))(u_isn u_hdip)))	16	18
8988	(((u_isn oa_wa))((u_hdip (u_csn cp_ent))(p_csn (p_isn ca_ent)))))))((ca_wa p_re)ca_wa)((cp_ent oa_ent)u_hdip))(u_isn u_hdip))(u_isn u_hdip))	13	18
8989	(((((p_isn ca_ent))((((p_re ca_wa)(cp_ent oa_ent))(oa_wa u_hdip))(u_isn u_hdip)))(u_isn oa_wa)))(u_csn cp_ent)oa_ent))(u_hdip (u_isn oa_wa)))	18	18
8990	(((oa_wa (u_csn cp_ent))(p_isn ca_wa))))))((p_re ca_wa)((cp_ent oa_ent)u_hdip))(u_isn u_hdip))(u_isn u_hdip))(u_isn u_hdip))(u_isn oa_wa)))	15	18
8991	((((u_isn oa_wa)u_csn)(cp_ent ca_ent))))))((ca_wa p_re)ca_wa))(cp_ent oa_ent)(oa_wa u_hdip)))(u_isn u_hdip))(u_isn u_hdip))(u_isn oa_wa)))	16	18
8992	(((u_hdip u_hdip)(((u_isn oa_wa)u_csn)(cp_ent ca_wa))))))((p_re ca_wa)((cp_ent oa_ent)oa_wa))(u_hdip u_hdip))(u_isn u_hdip))(u_isn oa_wa)))	14	18
8993	(((oa_wa (u_csn cp_ent))(p_isn ca_wa))))))((p_re ca_wa)((cp_ent oa_ent)oa_wa))(u_isn u_hdip))(u_isn u_hdip))(u_isn u_hdip))(u_isn oa_wa)))	15	18
8994	((cp_ent ca_ent))))))((ca_wa p_re)ca_wa)((cp_ent oa_ent)u_hdip))(u_isn u_hdip))(u_isn u_hdip))(u_isn u_hdip))(u_isn oa_wa))(u_csn cp_ent)))	19	18
8995	(((((p_isn ca_ent))((((ca_wa p_re)cp_ent)oa_ent)(oa_wa u_hdip))(u_isn u_hdip)))(u_isn oa_wa)))(u_csn cp_ent)(oa_ent oa_wa)))(u_isn u_hdip))	14	18
8996	(((u_hdip u_hdip))((u_isn oa_wa)u_csn)(cp_ent ca_wa))))))((p_re ca_wa)((cp_ent oa_ent)(oa_wa u_hdip)))(u_isn u_hdip))(u_isn u_hdip))(u_isn oa_wa)))	14	19
8997	((u_hdip u_hdip)((((u_isn oa_wa)u_csn)(cp_ent ca_ent))))))((ca_wa p_re)cp_ent)(oa_ent (oa_wa u_hdip)))(u_isn u_hdip))(u_isn u_hdip))(u_isn oa_wa)))	13	19
8998	(((((p_isn ca_ent)((((ca_wa p_re)ca_wa)((cp_ent oa_ent)oa_wa))(u_isn u_hdip)))(u_isn u_hdip))(u_isn oa_wa))((((u_csn cp_ent)oa_ent)(u_isn u_hdip))(u_isn u_hdip)))	17	21
8999	(((p_isn ca_ent)((((p_re ca_wa)((cp_ent oa_ent)oa_wa))(u_isn u_hdip))(u_isn u_hdip))(u_isn oa_wa))(u_csn cp_ent)))((ca_ent ca_wa)((p_isn ca_wa)(p_csn p_csn)))	16	21
9000	(((p_isn ca_ent))))))(ca_wa p_re)ca_wa)((cp_ent oa_ent)oa_wa))(u_hdip u_hdip))(u_isn u_hdip))(u_isn oa_wa))(u_csn cp_ent)))((ca_ent ca_wa)((p_isn ca_wa)(p_csn p_csn)))	14	22

Supplementary Figure 7. The length distribution of T-patterns (that is, the number of T-patterns of different lengths detected for each group) in EPM.
